# Development of a Flame Retardant and an Organohalogen Flame Retardant Chemical Inventory

**DOI:** 10.1038/s41597-022-01351-0

**Published:** 2022-06-13

**Authors:** Charles Bevington, Antony J. Williams, Colin Guider, Nancy C. Baker, Brian Meyer, Michael A. Babich, Sayon Robinson, Ann Jones, Katherine A. Phillips

**Affiliations:** 1 Directorate for Health Sciences, U.S. Consumer Product Safety Commission, Rockville, MD USA; 2 Center for Computational Toxicology and Exposure, Office of Research and Development, U.S. Environmental Protection Agency (U.S. EPA), Research Triangle Park, North Carolina USA; 3 Oak Ridge Associated Universities (ORAU), Oak Ridge, TN USA; 4 Leidos, Research Triangle Park, NC USA; 5 Senior Environmental Employment Program, U.S. Environmental Protection Agency, Research Triangle Park, Durham, North Carolina 27711 United States; 6 Directorate for Laboratory Sciences, U.S. Consumer Product Safety Commission, Rockville, MD USA; 7 Industrial Economics, Inc., 2067 Massachusetts Avenue, Cambridge, MA 02140 USA

**Keywords:** Chemical safety, Cheminformatics

## Abstract

There have been many attempts to compile comprehensive lists of flame retardants. However, this goal has proven challenging due to the heterogeneity of compounds that can be used as flame retardants coupled with changes in formulation chemistry over time. Flame retardants have been the focus of many recent existing hazard, exposure, and risk assessments. These assessments have been class-based or for individual chemical substances. Here, diverse sets of publicly available data sources from governmental organizations and the open literature were compiled to develop an inventory of chemicals used as flame retardants and organohalogen flame retardants. The chemical substances from these data sources were mapped to appropriate chemical identifiers via manual curation and deduplicated. Despite different data sources containing a large number of overlapping chemical substances, compiling information from multiple data sources was found to increase the breadth of potential flame retardant chemistries. The flame retardant and organohalogen flame retardant inventories were developed as a resource for scientists interested in better understanding properties of flame retardant and organohalogen flame retardant classes.

## Abstract (summary)

**Table:** 

Measurement(s)	chemical use information
Technology Type(s)	manual and automated literature collection

## Background & Summary

Flame retardants (FRs) have received greater attention over time due to their occurrence in environmental media and biological matrices^1,2^. Certain FRs have been classified as persistent, bioaccumulative, and toxic^3^. Up to the present, organizations have assessed and regulated FRs as single substances (e.g., decabromodiphenyl ether) and classes (e.g., Cyclic Aliphatic Bromides)^4–6^. FRs can be organized into groups or classes in different ways^7–9^. Common descriptions include organic vs. inorganic, halogenated vs. non-halogenated, and polymeric vs. non-polymeric^10^. Organohalogen flame retardants (OFRs) have received additional attention as a class, and can also be subcategorized, based on expected performance, chemical structure similarity, or biological similarity^9^. The U.S. Consumer Product Safety Commission (CPSC) is presently assessing OFRs.

After granting a petition in 2015, which requested that CPSC ban all OFRs as a class in certain consumer product categories, CPSC sought advice from the National Academies of Science, Engineering and Medicine (NASEM) on the best approach to implement a class-based assessment of OFRs. As part of their 2019 report, NASEM identified 161 OFR chemicals and 1,073 analogs (i.e., substances structurally similar to known flame retardants) and recommended a class-approach to assess hazards for 14 classes of OFRs based on chemical and biological similarity rather than an approach for one monolithic OFR class^9^. In 2020, CPSC staff issued a project plan that describes building out a comprehensive OFR inventory as a foundational activity that is expected to inform scoping of multiple class-based risk assessments^11^. The efforts to compile this inventory are described here. In addition to an OFR inventory, a broader inventory of FRs with any classification has been assembled and included here. This broader list will benefit organizations that require characterization of chemical uses to scope their chemical hazard, exposure, or risk assessments. The accurate identification of chemicals is an important and necessary step that informs source characterization and scoping.

The FR and OFR inventories described here expand on the 161 substances identified in the 2019 NASEM report and other recent compilations of FR and OFR chemistries^7,9^. Chemical inventories, by nature, are dynamic and the uses and applications of chemical substances are also dynamic. As such, this inventory represents a snapshot in time and reflects the compilation of data sources that were used during curation. A curated and accurate chemical list that has been attributed to reliable sources is a valuable resource for future assessments of flame retardant classes.

## Methods

**Fig. 1:**
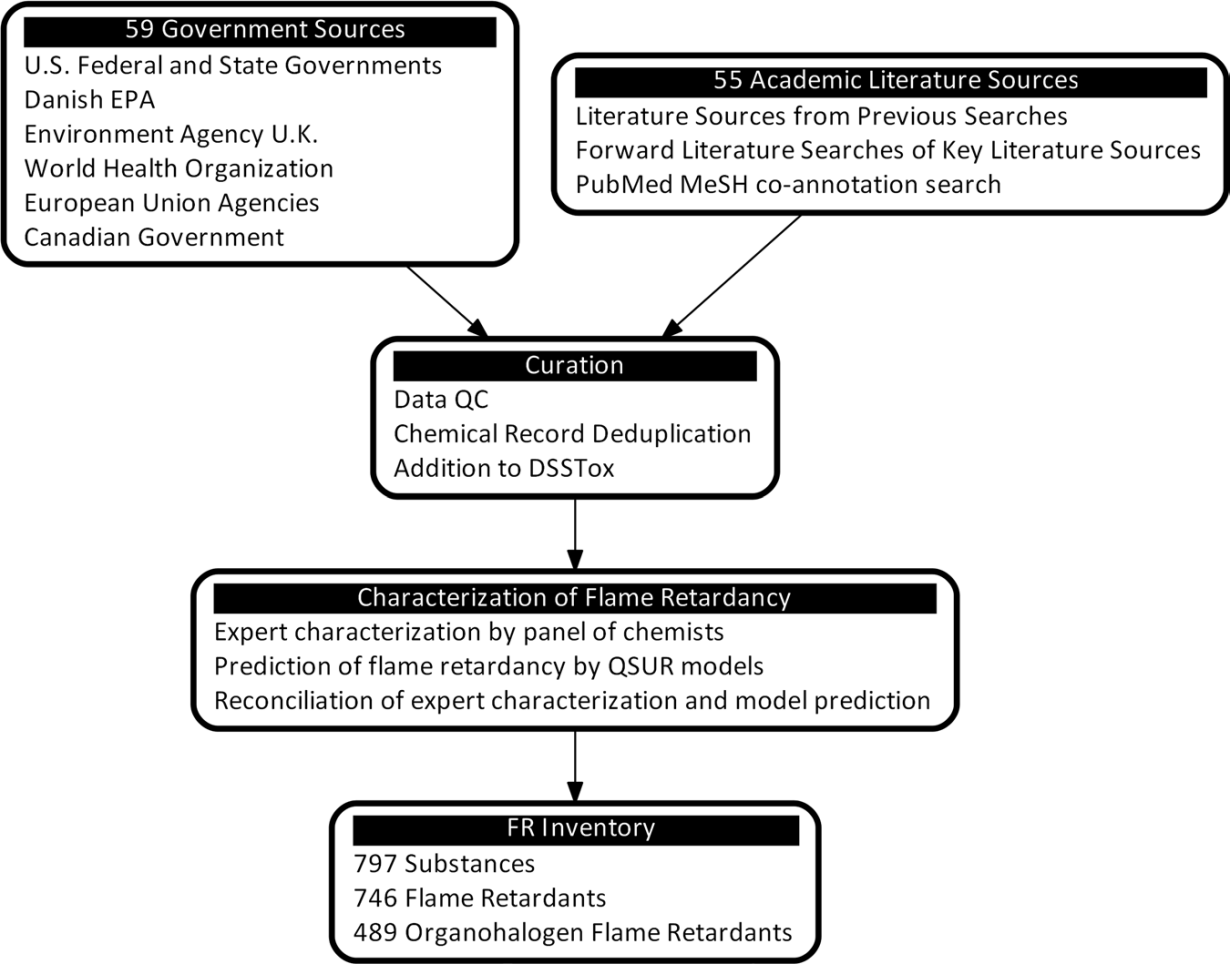
Process used to identify and curate flame retardant inventory.

Multiple approaches were used to identify and aggregate lists of FR substances, and to discern which of those were also OFR substances. The workflow used to collect, curate, and compile these FR substances into a single inventory is shown in Fig. 1. First, existing publicly available data sources from United States, Canadian, and European government organizations were compiled. Next, literature searches based on data from government sources, forward searching and PubMed Medical Subject Heading (MeSH) terms were used to identify data sources with curated FR or OFR lists in the open literature. Finally, manual deduplication and quality assurance processes were used to remove duplicate chemistries and chemicals that were incorrectly identified as FR chemistries.

Government organizations that have published data containing information on FR substances were: U.S. Consumer Product Safety Commission^9,12–23^, U.S. Environmental Protection Agency (EPA) Office of Pollution Prevention and Toxics (OPPT)^8,24–34^, U.S. EPA Office of Research and Development (ORD)^35–41^ Danish EPA^7,42–45^, Environment Agency UK^46^, World Health Organization (WHO) Internal Program of Chemical Safety (IPCS)^47–53^ European Food Safety Agency (EFSA)^54–59^, regulatory data sources from the European Union (EU)^60–62^, State of California health agencies^63–65^, Health Canada^66–68^, and the Commission for Environmental Cooperation^69^. One component of the U.S. EPA ORD data source was a literature search that queried MeSH terms extracted from PubMed’s MEDLINE. For each PubMed annotation of “flame retardant”, the co-occurring chemical MeSH identifiers were extracted from the database. One hundred forty-eight chemical MeSH terms were extracted from 1,551 PubMed citations about flame retardants and placed in an Excel worksheet for curation.

When more than one data source was available from a government agency, those data sources were compiled into one aggregated data source for simpler presentation. In total, 59 data sources were compiled from across these government organizations into ten aggregated data sources.

The open literature includes lists of FR substances identified in the context of source characterization (e.g., uses, applications, physicochemical properties), hazard identification, exposure assessment, or risk assessment. Included in these sources were three literature sources of which the authors were aware that focused on non-halogenated and inorganic chemistries^70–72^. These sources were included as most other literature sources focused on organic and halogenated substances. A manuscript published by Bergman *et al*. in 2012 was included as it contains multiple tables of potential FR substances aggregated from literature sources^73^. Finally, other literature sources were obtained by using Bergman *et al*., 2012 as a starting point and forward searching literature that cited this paper^73^. A total of 355 open access articles were found to cite Bergman *et al*. (Google Scholar, December 2020). One hundred thirty-nine of these literature sources contained the phrase “flame retardant*” and “CAS”, where the asterisk (*) denotes a wildcard search; 52 of those included a chemical list with >10 chemical names and/or identifiers. Chemical name and identifier information from these 52 articles were extracted and subsequently included as data sources for the FR inventory^1–3,10,74–121^. The decision to limit extracted information from this forward search to references with at least 10 unique FRs was intended to capture a large quantity of FR substances while reducing the initial burden for extracting and curating the inventory. It is recognized that this limit may exclude references where one or a few novel FRs are identified. For example, of the 87 articles that had fewer than 10 chemicals, only 17 manuscripts contained keywords such as “new”, “emerging”, “novel”, and/or “newly discovered”; upon further investigation only one of these references identified a possible compound that was not already included in the inventory by another source^122^. Further review of this one compound, indicated use as a flame retardant was not likely. In addition, inclusion of the PubMed citations in U.S. EPA ORD’s aggregated data source should provide coverage of publications discussing identification of novel FRs. Future updates of the inventory may include revisiting and extracting all or some of the remaining 303 references identified in this search as well as identifying new references based on references in the inventory. All 55 open literature data sources met a two-part *a priori* screening criteria: 1) chemical name and identifier information for ten or more flame retardant chemical substances and 2) the data source had been published within the past ten years. The ten-year timeframe was chosen because it has been approximately ten years since Bergman *et al*., was published. The ten chemical threshold was selected to prioritize references that compiled information on multiple flame retardants.

Chemical identifiers, typically chemical name and Chemical Abstract Services Registry Number (CASRN), were extracted from relevant tables within each data source. Through manual quality control (QC), CASRNs were corrected when deleted or incorrect CASRNs were originally assigned to chemical names, and duplicated substances were then removed. Incorrect chemical identifiers were corrected by mapping substances to DTXSIDs, which are unique substance identifiers from U.S. EPA’s Distributed Structure-Searchable Toxicity (DSSTox) database^123^. Substances that could not be mapped to an existing DTXSID record were queued for curation, added to the chemical registration database, and assigned new DTXSIDs. In total there were 797 registered substances in the FR/OFR inventory which can be freely accessed via U.S. EPA’s CompTox Chemicals Dashboard, henceforth referred to as the Dashboard, (https://comptox.epa.gov/dashboard).

After initial mapping of extracted chemical identifiers to DTXSIDs, chemicals were manually reviewed and curated by chemists with knowledge of flame retardants at both US CPSC and US EPA to characterize the likelihood of flame retardancy and identify chemicals that were unlikely to be FRs. For example, fluorinated substances used as fire suppressants or fire extinguishers were labeled as non-flame retardants as fire suppression agents are defined by the Organisation for Economic Co-operation and Development (OECD) as “chemical substances that aid to slow down combustion once started” while flame retardants are “chemical substances that alter the normal degradation or combustion processes of plastics, rubber, textiles, papers and woods, etc.”^124^. Physicochemical properties were also considered to identify substances that were gases at room temperature, were soluble in water, and likely polymeric; these chemicals were unlikely to be FRs. The physicochemical properties used to screen for these criteria were boiling points of <25 degrees, a log K_ow_ of <1, water solubility >100 mg/L, vapor pressure of >10 torr, or were otherwise highly reactive, flammable, or pyrophoric^125^. While chemists performing review of the flame retardancy of these substances referenced these physical properties, their expert judgement was ultimately used to make an initial decision as to whether a substance should be considered a flame retardant. Further technical validation of the flame retardancy of the inventory is described below in the Technical Validation section.

**Fig. 2:**
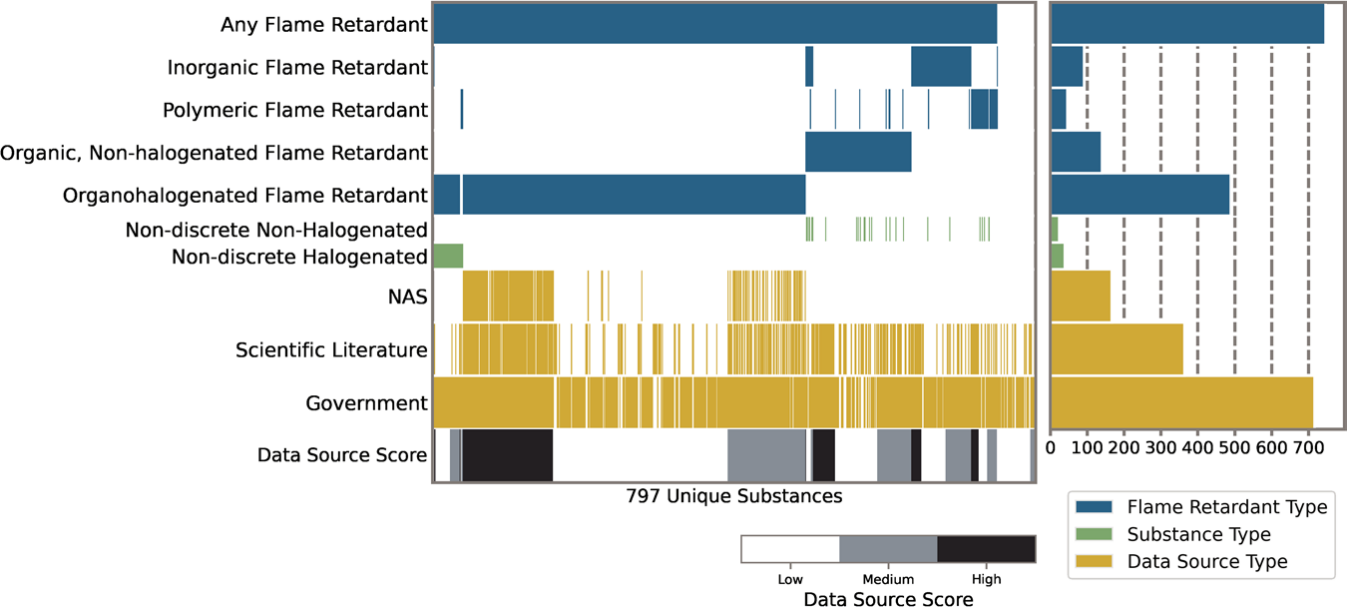
Flame retardant data inventory for the 797 compounds included in the inventory. The classifications of flame retardant types are shown by blue, non-discrete substance classifications are show in green, and the type of literature each substance was found in is shown in yellow.

Chemicals that were considered likely to be FRs from the identified data sources were then further classified into the following flame retardant categories: organohalogen flame retardants (substances whose structure contained at least one carbon-halogen bond), organic non-halogenated flame retardants (substances that contained carbon-carbon bonds, but no halogens), inorganic flame retardants (substances that contained no carbon-carbon bonds), and polymeric flame retardants (substances that contained repeating monomer units). Non-discrete substances (i.e., mixtures of two or more chemical substances and cation-anion pairs), were placed into two categories: one where at least one component contained a carbon-halogen bond and one where no component contained a carbon-halogen bond. Chemical mixtures can include different combinations of congeners or isomers and combinations of either halogenated or non-halogenated substances. Chemical mixtures and generic chemical substances are retained in the inventory, although they need to be treated differently in future work related to assessing hazards, exposures, or risks from chemical substances. The summary of 797 substances in the inventory and their curated FR classifications are shown in Fig. 2; this shows substances that were curated to be any type of flame retardant and those that were listed in sources but were not determined to be likely flame retardants.

All thirteen aggregated data sources were used to calculate a categorical data source score. If only one data source identified a chemical as an FR, it was assigned a low data source score. If two or three data sources identified a chemical as an FR, it was assigned a medium data source score. If more than three data sources identified a chemical as an FR, it was assigned a high data source score.

## Data Records

The 59 data sources obtained from governmental data sources were grouped into their source governmental organizations as previously described. The 55 data sources from open literature were also grouped into their aggregate data sources as previously described. Each chemical on the inventory can be attributed to at least one of the 13 aggregated data sources. A data file of this inventory is publicly available on EPA’s figshare site with a separate worksheet for each aggregated data source^126^. Compiling data from multiple sources increases the confidence that a wider breadth of FR chemistries has been identified. However, due to the diverse nature of these data sources, additional screening and QC were necessary for compilation into a uniform FR and OFR list. Curation notes are included in inventory^126^.

In addition, the final lists of substances on the FR inventory from this work are available as three lists on EPA’s CompTox Chemistry Dashboard: one for the entire inventory (n = 797 substances), one for the likely flame retardants in the inventory (n = 746), and one for the likely organohalogen flame retardants in the inventory (n = 489). These lists are accessible via https://comptox.epa.gov/dashboard/chemical-lists/FRFULLLIST. The Dashboard gives researchers the capability to view and download structures, known biological activity, and other chemical attributes including physicochemical properties and *in vivo* hazard data.

Organohalogen flame retardants in Table 1 are defined as chemicals that are non-polymeric or non-UVCB (unknown or variable composition, complex reaction products, or biological materials), that contain a carbon-halogen bond, and that are not inorganic. For Table 2, mixtures and cation-anion pairs that contain a carbon-halogen bond were retained. In addition, >100 reported PBDE (polybrominated diphenyl ether) or PBB (polybrominated biphenyl) congeners with only one reported data source were retained. The impact of retaining or removing these chemicals in an organohalogen flame retardant inventory list can be seen in Table 2.

**Table 1:** Number of unique substances categorized as an FR and the subset of those that were classified as an OFR in each Aggregate Data Source.

Aggregate Data Source	FR Substances	OFR Substances
US CPSC^9,12–23^	220	177
US EPA OPPT^8,24–34^	130	70
US EPA ORD^35–41^	437	316
Danish EPA^7,42–45^	106	87
Environment Agency UK^46^	133	52
WHO IPCS^47–53^	217	124
EFSA^54–59^	94	94
EU^60–62^	104	64
Biomonitoring California^63–65^	54	54
Health Canada^66–68^ Commission for Environmental Cooperation^69^	68	41
Bergman *et al*.^73^	87	72
Forward Search of Bergman *et al*.^1–3,10,74–121^	319	216
Non-Halogenated and Inorganic^70–72^	93	44
TOTAL	746	489

**Table 2:** Number of unique OFR substances present in each NAS OFR category found in the NAS report, the FR Inventory, and substances in the OFR Inventory without mixtures or congeners with only one reported data source.

NAS OFR Category	OFR Inventory	No mixtures or congeners
Polyhalogenated Alicycles	22	22
Polyhalogenated Aliphatic carboxylates	3	3
Polyhalogenated Aliphatic chains	47	20
Polyhalogenated Benzene Alicycles	4	4
Polyhalogenated Benzene Aliphatics and Functionalized	20	20
Polyhalogenated Benzenes	50	37
Polyhalogenated Bisphenol Aliphatics and Functionalized	14	14
Polyhalogenated Carbocycles	20	20
Polyhalogenated Diphenyl Ethers	223	59
Polyhalogenated Organophosphates	42	42
Polyhalogenated Phenol Derivatives	8	8
Polyhalogenated Phenol Aliphatic Ethers	11	11
Polyhalogenated Phthalates/Benzoates/Imides	19	17
Polyhalogenated Triazines	6	6
Sum of Categories	489	278

## Technical Validation

Each chemical identifier (both name and CASRN) was curated according to established curation procedures adopted by the US EPA in the construction of the DSSTox database that underpins the Dashboard^123^.

To further bolster classification of a substance as a Flame Retardant beyond the initial characterization described in the Methods section, Quantitative Structure-Use Relationship (QSUR) models were employed on the inventory^127^. These models take a substance’s structure as input and return a probability between 0 and 1 to indicate the likelihood of a substance serving a given functional role (in this case, the functional role would be flame retardant). A lower probability implies a lower likelihood of serving as a flame retardant. Of the 797 substances, only 6 substances had no known structure. These substances could not be predicted by the QSUR models. Of the remaining 792 substances which did have a known structure, QSUR models predicted 657 of them to be flame retardants. For substances where the QSUR predictions agreed with initial characterization efforts, no further curation of flame retardancy was performed. However, if there was disagreement between the initial characterization and QSUR prediction, further review was undertaken. One of two cases required further review 1) cases in which a substance was predicted to be a flame retardant (i.e., its QSUR probability was greater than or equal to 0.5) but had been determined to not be a flame retardant by earlier curation efforts and 2) cases in which a substance was not predicted to be a flame retardant (i.e., its QSUR probability was less 0.5) but had been identified as a potential flame retardant by at least one data source in the inventory.

There were 19 substances that met the criteria for the first case and 89 substances that met the criteria for the second case. For these substances, if their physicochemical properties were within the ranges specified in Methods, then the substance was classified as a likely flame retardant, otherwise the substance went on to further expert review. For the second case, if the chemical’s physicochemical properties were outside the physicochemical property ranges it was marked as not a likely flame retardant, otherwise it went on to further expert review.

Further expert review consisted of a series of meetings held by chemists from US CPSC and US EPA with considerable knowledge of fire chemistry. Before each meeting experts independently considered the structure of the substance, physicochemical properties, QSUR prediction, and gathered market use information for substances to be further reviewed. At each meeting the team reviewed the initial characterization, QSUR prediction, and new characterization. Consensus was reached for each of the 108 substances and this consensus became the final characterization for each of these substances.

The final determination for all substances in the of the inventory yielded 746 likely flame retardant substances. It should be noted that the QSUR predictions which were used to quickly screen a substance for flame retardancy obtained were able to correctly predict 85% (676) of the likely flame retardants in the inventory. While, not a perfect predictor, using this method substantially reduced the number of substances required for manual review by an expert panel.

For completeness, all 797 potential FRs have been included in the inventory, however the substances that are likely FRs from the technical validation described above are indicated as such in the inventory by a binary flag (is_fr) where a 0 indicates the substance was found to be an unlikely FR and a 1 indicates the substance was found to be a likely FR. Further, any relevant information that was used to make a determination of the flame retardancy of substances in the inventory are provided in the “integrated_comments”^126^.

## Usage Notes

The composition and nature of FR chemistries has changed over time and continues to evolve. Formulation of FRs into various materials and applications is defined by performance, however there are increasing restrictions on chemistries that can be used especially as there are many approaches to reduce flammability hazards (e.g., barrier technology, use of non-flammable, fire resistant materials). Over time, more non-halogenated and polymeric FRs have been introduced into the market. In addition, material scientists and formulation chemists have considered how non-halogenated FRs, polymeric FRs, and reactive FRs can be used as replacements for halogenated FRs. Due to the evolving nature of FR chemistries, this inventory only provides a snapshot of the FR chemical universe and includes chemistries that have been used in the past 10 years. Additional market-use research is needed to characterize trends associated with manufacturing, production, and use of diverse FR chemistries over time. For example, market-use research describing trends in manufacturing, processing, uses, and applications of OFRs across their lifecycle was completed in coordination with development of this inventory was recently completed^128^. These changes as well as the potential for new information to become publicly available over will necessitate updates to this inventory. Collection and curation of new information could be performed and released in new versions of this inventory as well as on EPA’s CompTox Chemicals Dashboard.

As government entities consider regulation of flame retardant chemical substances, this FR Inventory will aid in identifying which chemicals are known and likely flame retardants, and to which classes and subclasses of FRs the substances belong. This inventory also provides a well curated training set for more refined QSUR models that can aid assessors by predicting flame retardancy in substances that are not in the inventory. For example, chemicals that have a very high QSUR prediction value (e.g., 0.95), but were not identified by one of the data sources described here, could be considered as potential flame retardants in future work.

### Disclaimer

The views expressed in this publication are those of the authors and do not necessarily represent the views of the United States Environmental Protection Agency nor the United States Consumer Product Safety Commission.

## Supplementary information

PUBMED Mesh Terms Search

Main FR | OFR Inventories

## Data Availability

Two literature tools have been made available in the Supplemental Information. The file FlameRetardantsFromMeSHMining.xlsm is the chemical – Flame Retardant co-annotation output from the process briefly described in the Methods. The second tool is a version of the PubMed Abstract Sifter populated with the resulting Flame Retardant Inventory^129^. The Abstract Sifter is tool for easy querying of PubMed with a list of chemicals. Both tools have ReadMe sheets with instructions for usage. An additional user guide is provided for the Abstract Sifter.
