# Supplementary material for: Development of a Flame Retardant and an Organohalogen Flame Retardant Chemical Inventory
Source: Sci Data. 2022 Jun 13;9:295. doi: 10.1038/s41597-022-01351-0 (PMC9192637; doi:10.1038/s41597-022-01351-0)
Supplement: Supplementary file 1 — PUBMED Mesh Terms Search [file 41597_2022_1351_MOESM1_ESM.pdf]

Abstract Sifter User Guide, Version 6.0

Availability: The Abstract Sifter and documentation is freely available for download at https://gaftp.epa.gov/COMPTOX/Sustainable_Chemistry_Data/Chemistry_Dashboard/Abstract_Sifter/AbstractSifter.zip

This beta version of 6.0 not yet available through Dashboard download!

Contact: Nancy Baker at baker.nancy@epa.gov

Disclaimer: The views expressed in this user guide are those of the authors and do not necessarily represent the views or policies of the U.S. Environmental Protection Agency.

Abstract Sifter User Guide – Version 6.0

This user guide describes the functionality of the PubMed Abstract Sifter. The reader is invited to download the tool from the freely accessible ftp site and follow along: https://gaftp.epa.gov/COMPTOX/Sustainable_Chemistry_Data/Chemistry_Dashboard/Abstract_Sifter/AbstractSifter.zip Version 6.0 not publicly available!

This document provides guidance on the use of the Abstract Sifter through a series of screen shots showing the most common tasks in the tool followed by some helpful tips.

What's new in version 6?

Wait a minute … read this first:

As the Abstract Sifter grows it may get more sheets. If this is confusing or irritating, please feel free to hide the sheets you don't use or don't use often. This might simplify your life, which is a good thing. To hide sheets, right click on the sheet tab you want to hide at the bottom and when the menu pops up, click on Hide. To unhide, click on any sheet tab and then Unhide where you'll be given a selection of hidden sheets to unhide.

Version 6 has one new major function and a few smaller nice-to-have things.

- Term Expansion – this feature allows the end user to compile a list of terms to be mapped to or expanded by (however you want to look at it) another term. This is an opportunity to use an ontology or just a set of desired terms and see how often they appear in your corpus and to find them quickly. This feature is learned best by examples so check out the section below.
- If two notes are taken on the same citation and the yes / no / maybe designation are not the same, the Highlight Noted PMIDs function will color the PMID purple. This feature is useful if you are combining Notes from different researchers and your team wants to find and resolve differences.

In case you didn't know, Version 5.6 had features that facilitate larger, more complex projects.

- The Log sheet now allows the user to select rows and click on Run in Batch. When Batch is selected, each selected query will be run in turn and the results appended.
- The Landscape sheet has a new feature that allows the user to select cells and then click on More Stuff, then Send to Log. On the Log sheet the corresponding queries will be seen in italics (meaning they have not yet been run) and from there the rows can be selected and run in batch. The preferred name value (column B) will be put into column F where it is called the Batch Tag. When a query on the Log sheet is run in batch, the Batch Tag will be appended to the rows retrieved on the Main sheet. The More Things button on the Main sheet has the option to Summarize the row counts by this Batch Tag.

Let's start!

First open the Abstract Sifter file AbstractSifter_v5.7.xlsm. A security warning may appear. If so, be sure to enable content as shown in Figure 1.

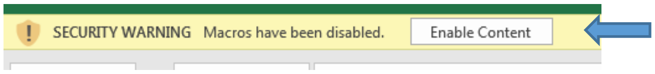

Figure 1. Enable macros upon opening

Once open you will see that the Abstract Sifter Excel file consists of 11 sheets. Each sheet is described briefly in the table below.

**Sheet name**	**Sheet Function**
ReadMe	Basic information on the sifter with links to more documentation
Main	Starting point for PubMed queries and for sifting
Abstract	The sheet where the citation abstract is shown

Notes	Notes and tags are inserted here
Log	Log of every query run on Main sheet
PathwayQueries	Repository of sample queries to use in research disease or treatment pathways (e.g., AOPs)
SampleQueries	Repository of sample queries to use as starting points
Landscape	High level view of literature for entities
CuratedLists	A place to keep lists of chemicals or other entities
TermExpand	Mapping terms to higher level concepts

The Abstract Sifter is growing. If you find the number of sheets onerous, hide (or even delete) the ones you don't use. For instance, the sheet CuratedLists is pretty specialized. Feel free to hide it and unclutter your workspace. To hide a sheet, right click on the tab at the bottom, then click on Hide. To unhide, click on any sheet, right click and then click on Unhide. You'll be give a list of hidden sheets to unhide. If you don't think you'll use the MeSH Mine function, delete the hidden sheet MappingsHide.

You can also freely change the order of the sheets.

The Abstract Sifter you open is likely to have rows in it from previous use. These are left as examples, but they may be deleted. In the Sifter, it is generally best to select an entire row and then click on the delete button in the Excel toolbar or right-click and then delete.

Main Sheet

The Main sheet is where the basic functionality of Abstract Sifter occurs, including functions we call "sifting". To begin using the Abstract Sifter, the end-user clicks on the *Query PubMed* button at the top of the screen in the Main sheet. A form is displayed in which the end-user types a PubMed query of interest (Figure 2). In the example, we are showing a very simple query: "chlorpyrifos", but these queries can be more complex. The text that the user enters into the box is sent to PubMed, so all

PubMed syntax rules apply.   (For a review of this syntax, visit https://www.ncbi.nlm.nih.gov/books/NBK3827/) NOTE: NLM changed PubMed early 2020 and reprogrammed the search algorithms. Since then, the search algorithms used by the web services do not return the exact counts returned by the PubMed web site.

Note also, that the end-user can optionally append the result of a query to the records currently on the Main sheet.

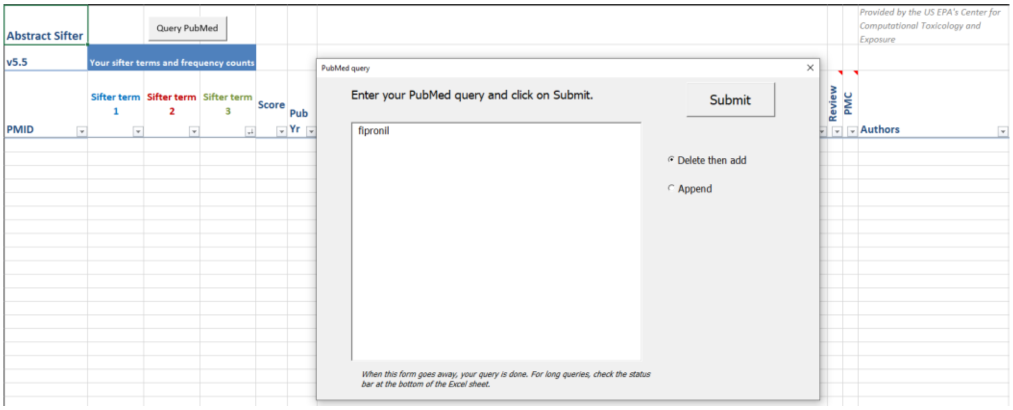

Figure 2. Running a PubMed query

When finished entering the query, the user clicks on *Submit* and the query is packaged by a Visual Basic Application (VBA) into an e-utility command that is passed to the NCBI (National Center for Biotechnology Information) web services. (Note that using Sifter Query PubMed capability requires internet access.) The first response returned by the utility is the number of articles found. (Figure 3) This number is displayed, and the user is asked if he/she want to continue. If the number of articles is over 10,000, the query will not be run and the user is encouraged to refine the query to return fewer records.

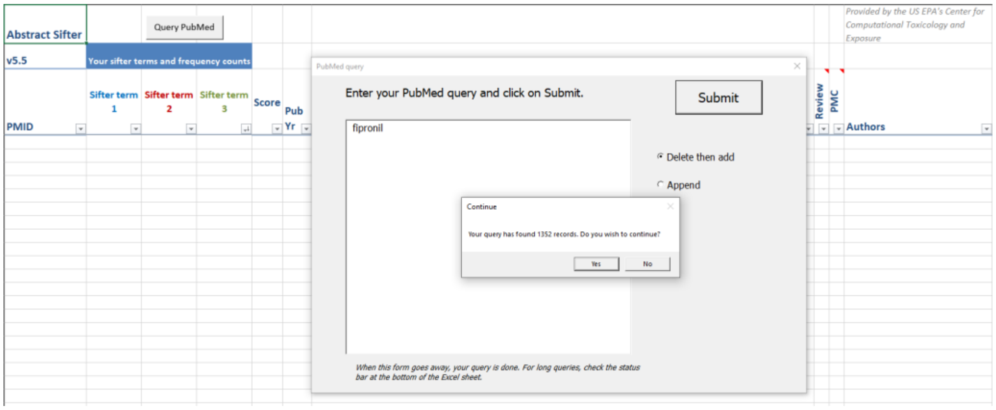

Figure 3. Responding to PubMed

If the returned results are fewer than 10,000 and the user indicates he/she wants to continue, the articles are downloaded from NCBI by Excel, and regular expressions are used to parse the citations for title, abstract, authors, publication year, journal, and PubMed identifier. Each record returned is inserted into a row in the Main sheet. Any rows in the Main sheet from a previous query are deleted unless the end-user chose the Append option. The Append option adds the new results to the end of the Main sheet. You can watch the status bar at the bottom of Excel to see how far along the retrieval process is. For longer queries, a cancel form will appear and the user can use it to cancel the process.

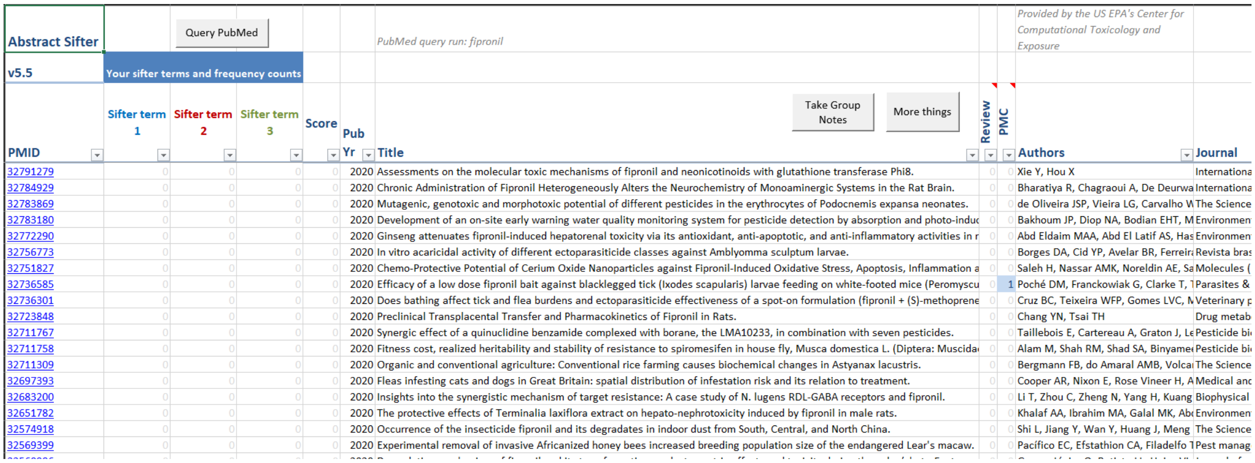

Figure 4.Results from PubMed query - before sifting

At this point the results of the query are stored in the Main sheet and can be browsed like any other data in a spreadsheet (Figure 4); however, the most effective way to find articles of interest is to use the

innovative sifter functionality. To demonstrate this functionality, we will continue to use our example of fipronil.

Let us suppose at this point that we are looking for dose-response toxicity data for fipronil. We type the term “fipronil” in cell B3, “toxic” in C3, and “mg/kg” in D3. As we finish typing and move to the next cell, the Abstract Sifter will count the occurrences of the terms in the title, abstract, and key words combined. The citations can then be sorted by these counts, either individually or by the total. Figure 5 shows what the Sifter looks like when these terms have been entered into cells B3, C3, and D3 and then the entries sorted by occurrence counts of “fipronil” in descending order. PubMed 12442503 has 26 occurrences of “fipronil”, 13 of “toxic”, and four of “mg/kg”. This article indeed describes a toxic doses of the chemical in various animal species.

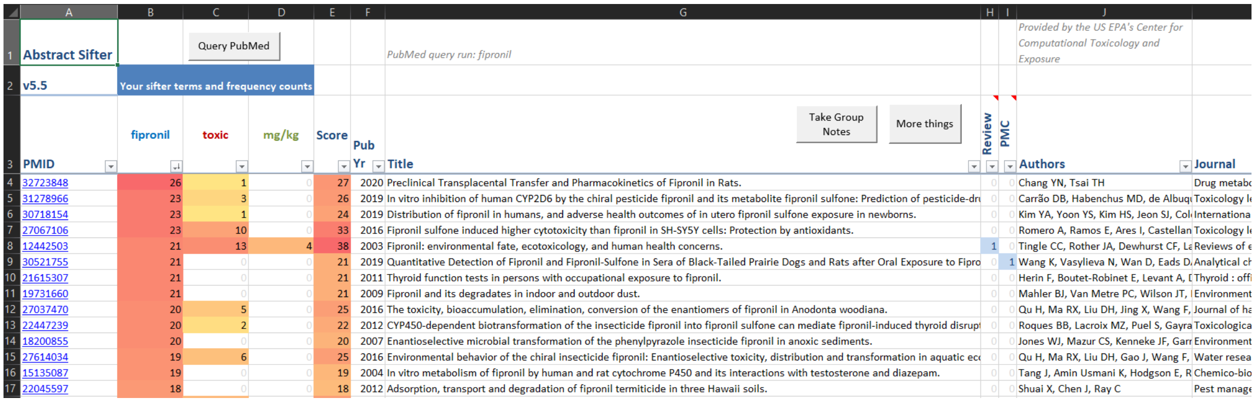

	A	B	C	D	E	F	G	H	I	J	
1	Abstract Sifter		Query PubMed				PubMed query run: fipronil			Provided by the US EPA's Center for Computational Toxicology and Exposure	
2	v5.5	Your sifter terms and frequency counts									
3	PMID	fipronil	toxic	mg/kg	Score	Pub Yr	Title (Take Group Notes / More things)	Review	PMC	Authors	Journal
4	32723848	26	1	0	27	2020	Preclinical Transplacental Transfer and Pharmacokinetics of Fipronil in Rats.	0	0	Chang YN, Tsai TH	Drug metabo
5	31278966	23	3	0	26	2019	In vitro inhibition of human CYP2D6 by the chiral pesticide fipronil and its metabolite fipronil sulfone: Prediction of pesticide-dru	0	0	Carrão DB, Habenchus MD, de Albuqu	Toxicology le
6	30718154	23	1	0	24	2019	Distribution of fipronil in humans, and adverse health outcomes of in utero fipronil sulfone exposure in newborns.	0	0	Kim YA, Yoon YS, Kim HS, Jeon SJ, Col	Internationa
7	27067106	23	10	0	33	2016	Fipronil sulfone induced higher cytotoxicity than fipronil in SH-SY5Y cells: Protection by antioxidants.	0	0	Romero A, Ramos E, Ares I, Castellan	Toxicology le
8	12442503	21	13	4	38	2003	Fipronil: environmental fate, ecotoxicology, and human health concerns.	1	0	Tingle CC, Rother JA, Dewhurst CF, La	Reviews of e
9	30521755	21	0	0	21	2019	Quantitative Detection of Fipronil and Fipronil-Sulfone in Sera of Black-Tailed Prairie Dogs and Rats after Oral Exposure to Fipro	0	1	Wang K, Vasylieva N, Wan D, Eads D.	Analytical ch
10	21615307	21	0	0	21	2011	Thyroid function tests in persons with occupational exposure to fipronil.	0	0	Herin F, Boutet-Robinet E, Levant A, D	Thyroid : offi
11	19731660	21	0	0	21	2009	Fipronil and its degradates in indoor and outdoor dust.	0	0	Mahler BJ, Van Metre PC, Wilson JT, I	Environment
12	27037470	20	5	0	25	2016	The toxicity, bioaccumulation, elimination, conversion of the enantiomers of fipronil in Anodonta woodiana.	0	0	Qu H, Ma RX, Liu DH, Jing X, Wang F,	Journal of ha
13	22447239	20	2	0	22	2012	CYP450-dependent biotransformation of the insecticide fipronil into fipronil sulfone can mediate fipronil-induced thyroid disrupt	0	0	Roques BB, Lacroix MZ, Puel S, Gayra	Toxicologica
14	18200855	20	0	0	20	2007	Enantioselective microbial transformation of the phenylpyrazole insecticide fipronil in anoxic sediments.	0	0	Jones WJ, Mazur CS, Kenneke JF, Garr	Environment
15	27614034	19	6	0	25	2016	Environmental behavior of the chiral insecticide fipronil: Enantioselective toxicity, distribution and transformation in aquatic eco	0	0	Qu H, Ma RX, Liu DH, Gao J, Wang F,	Water resea
16	15135087	19	0	0	19	2004	In vitro metabolism of fipronil by human and rat cytochrome P450 and its interactions with testosterone and diazepam.	0	0	Tang J, Amin Usmani K, Hodgson E, R	Chemico-bio
17	22045597	18	0	0	18	2012	Adsorption, transport and degradation of fipronil termiticide in three Hawaii soils.	0	0	Shuai X, Chen J, Ray C	Pest manage

Figure 5. After sifter terms were entered into cells B3, C3, D3 and sorting on B3

Abstract Sheet

To see the abstract for any of the retrieved articles, we can either click on the PubMed ID hyperlink to be taken to PubMed, or we can double-click on any other cell in the row for this article. This action brings us to the Abstract sheet where the abstract is displayed along with other article meta-data like title and authors (Figure 6).

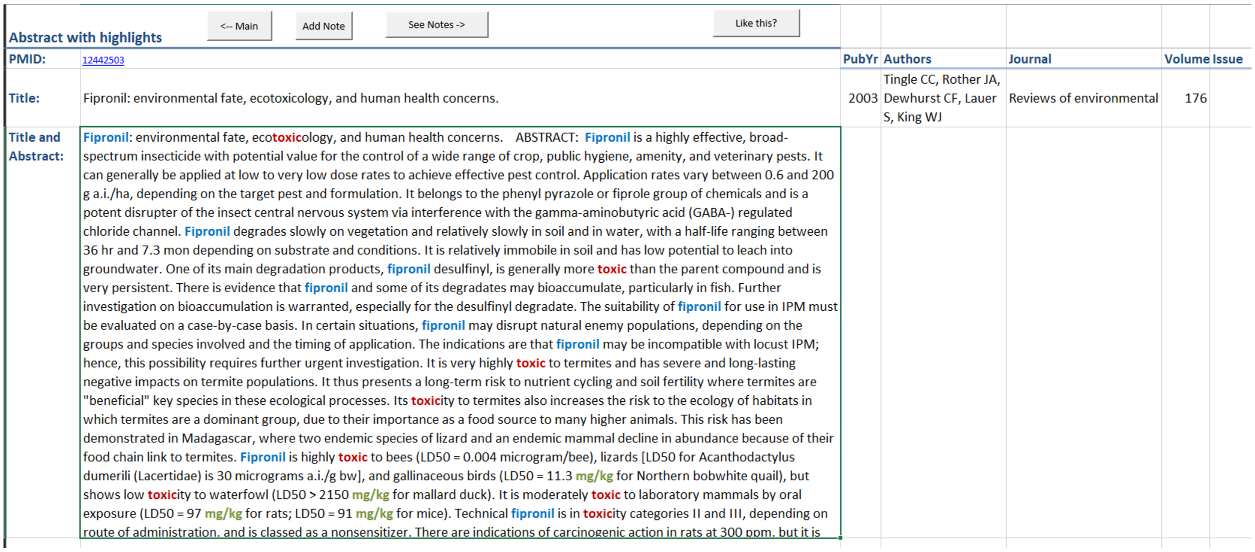

Figure 6a. An abstract with highlighted sifter terms.

There are several aspects of the Abstract sheet that are important to note. First, the sifter terms in the abstract are highlighted. The font colors reflect the colors of the fonts in cells B3, C3, and D3 entered into the Main Sheet. This highlighting makes the reading the abstract easier by drawing attention to sentences that might be of more interest.

With version 3 of the Abstract Sifter, the *Like this?* Button was added to the top of the Abstract Sheet. Click on this button allows the user to find articles in PubMed that are similar to the article on the Abstract sheet or find articles in PubMed Central that cite that article in question. (Figure 6b.) These functionalities are likely familiar as they are offered on the PubMed Entrez web site. The results can be appended to results already on the Main sheet. A colorization feature has been added to make the titles either yellow or green. This way the user can see which rows were from the original query and which were retrieved from the Like this functionality.

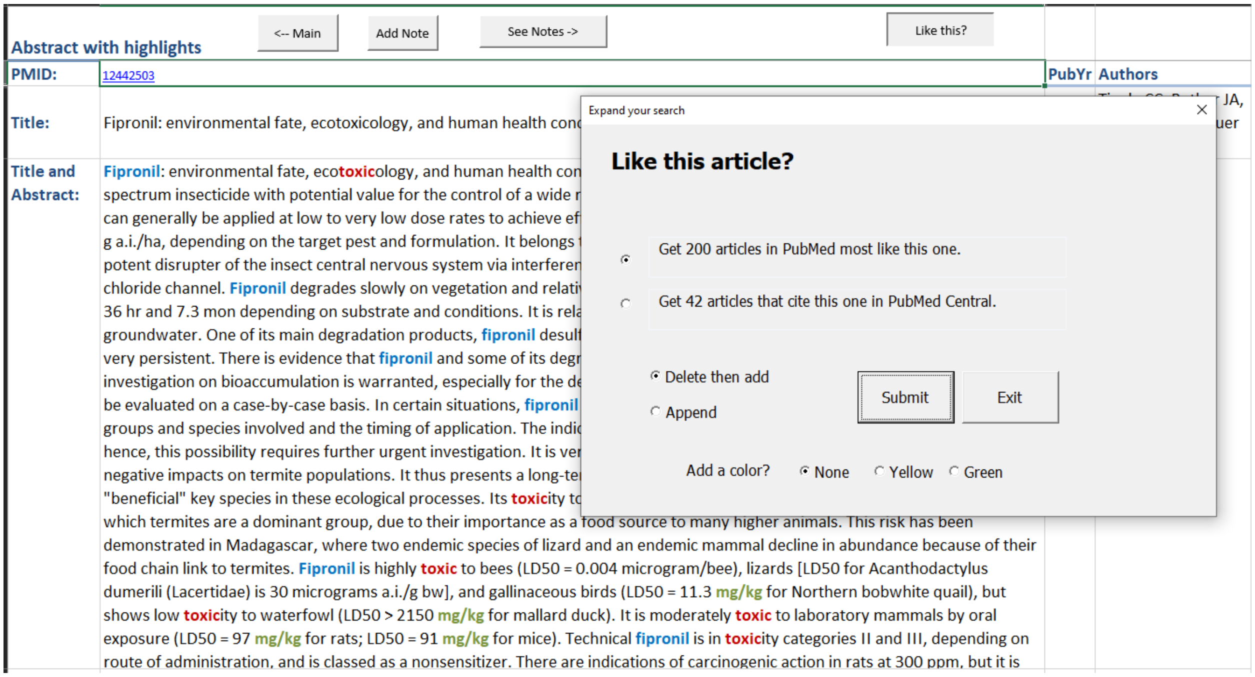

Figure 6b. Clicking on the Like this? Button on the Abstract Sheet.

Sifting the results through specifying sifter terms in B3, C3, and D3 can be repeated as many times as the user wishes. Similarly, new PubMed queries can be run, altered, rerun. There are no restrictions on either of these activities other than the 10000 record return limit.

Notes Sheet

Given the dynamic nature of the Sifter, many users find it helpful to be able to make notes on articles that they want to track. There are two ways using the Sifter to take notes: one way is through the Main sheet, and the other way starts with the Abstract sheet. To return to our case study, let us say that we have found a set of articles on the Main sheet that we know we need to read in depth. We can select these articles and then click on the *Take Group Notes* button. A form appears where we can enter information into fields called Tag and Notes. These elements are self-defined. We can also click on *yes*, *no*, or *maybe*. The note-taker can enter her/his initials or name in the Who field. This set of variables is a quick way to associate articles with a note. Notice that these choices each come with a color (yes-green, no-red, and maybe-yellow). Entering any of these fields is optional. (Figures 7 and 8.) When we

click on the *OK* button, each article selected will be inserted into the Notes page with the corresponding information (Figure 9) and the PubMed ID (PMID) on the Main sheet will be colorized.

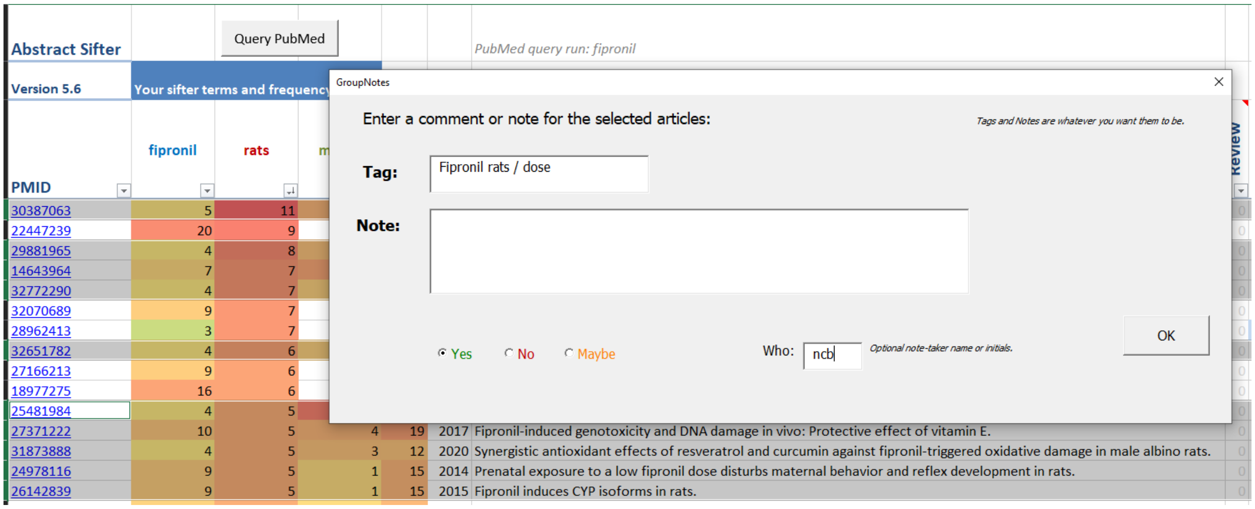

Figure 7. Taking group notes.

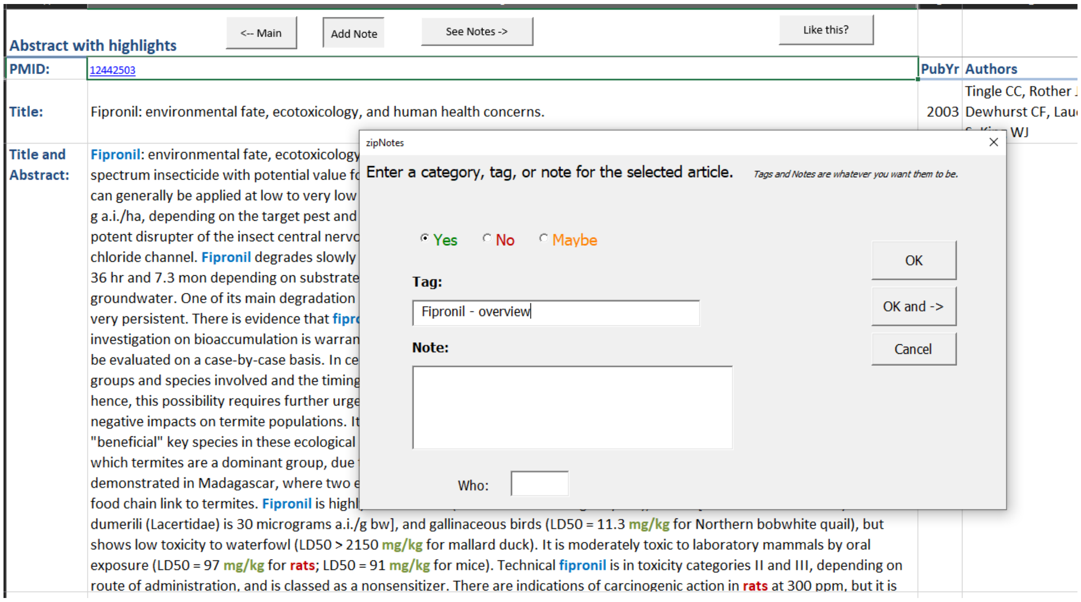

Figure 8. Taking single notes on the Abstract sheet.

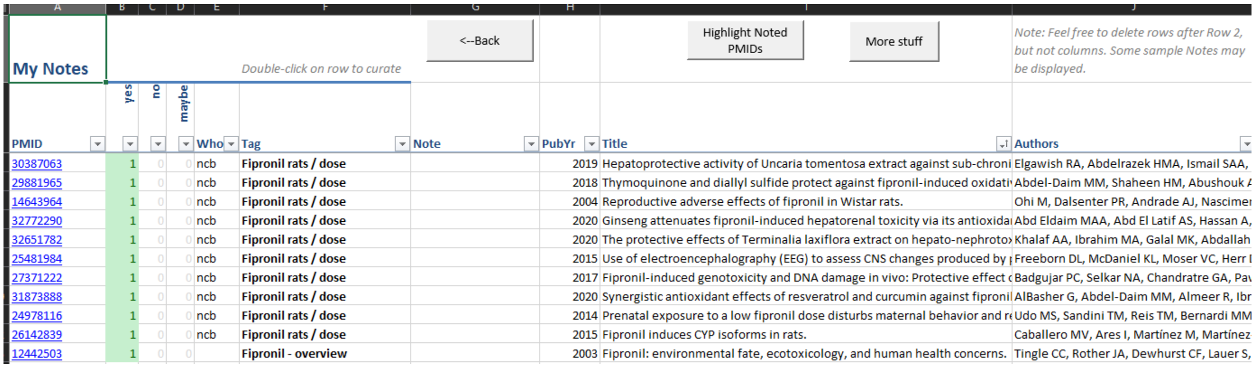

Figure 9. The Notes page. Remember to save your workbook.

The second option for note taking starts with the Abstract Sheet. (Figure 8) The "Add Note" button in the top row allows notes to be inserted into the Notes Sheet using the same form used to add Group Notes described above.

The note-taking can be used to help keep track of which citations have been read and evaluated and which have not. On the Main sheet the PMIDs can be sorted by the noted color using the built-in Excel sorting functionality. (Figure 10) The More Things button on the Main sheet will allow the end-user to delete duplicates and highlight noted citations with colorization.

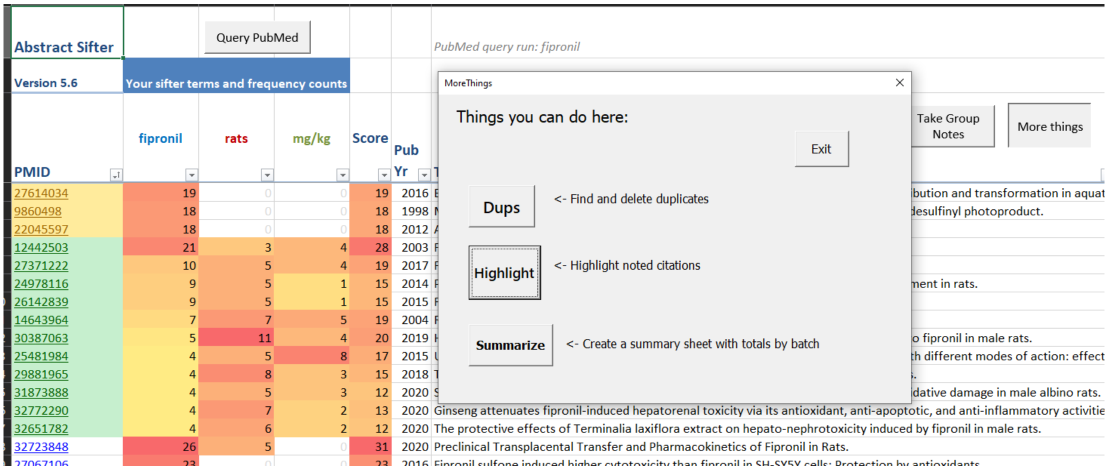

Figure 10. After clicking on More things, then and then sorting by color

The user can make changes to the Notes sheet by editing, adding or deleting rows below row 2. In version 4 of the Abstract Sifter, double-clicking on a row in the Notes sheet brings up a curation form (Figure 10a). Here the end-user can enter and update tags, notes. Text in the large box can be selected and dragged to any of the smaller tag and notes boxes. The Refresh button brings the title and abstract back in and refreshes the view. The purpose of this form is to allow quick easy extraction of information from the abstract into separate fields. The copy button sends the elements of the citation and your notes to the clipboard; then you can open up Word and paste the clipboard contents there. The Notes sheet can be copied, printed, or the Notes can be exported to a text file for further import into other tools like Word.

New in this version is the capability to attach a pdf. Before you can attach a pdf, the file must reside on your computer. Click the Attach Local PDF button and find the pdf. You can open the pdf by clicking on the Open button or on the pdf hyperlink in column U. Note that while Abstract Sifter files can be shared with colleagues, the pdfs are local and specific to one machine.

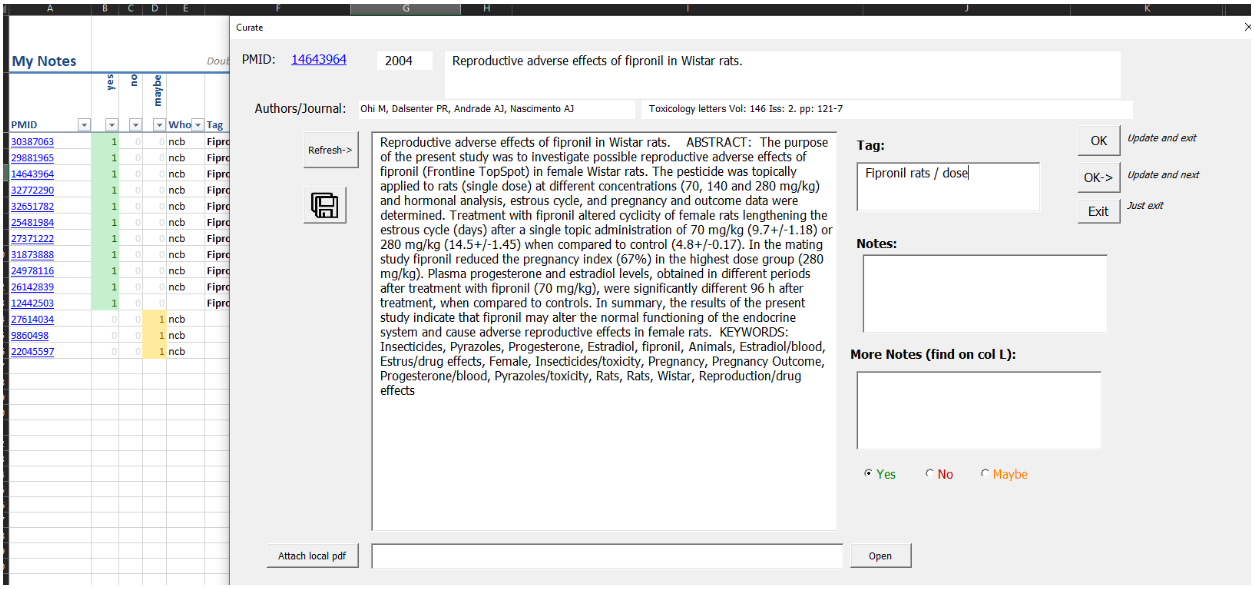

Figure 10a. Double-clicking on a row in the Notes sheet brings up a curation form.

Log / Batch sheet

The Log sheet keeps track of the queries you have run. The Abstract Sifter routines insert a row into the sheet every time you complete a query. These queries can be viewed and rerun. To rerun a query, simply double-click on it. (Figure 11.) Starting with version 5.6 a new feature called Batch Run has been implemented to allow the user to run multiple queries and append the results from each on the Main sheet. To run in batch, select rows and click on the Run in Batch button. A Batch Tag can be added or modified on the Log sheet. This tag will be added to the Main sheet results and used to help summarize the results of multiple runs. This summary functionality is accessible on the Main sheet through the More things button.

Delete any or all rows after Row 2 if you want to clear old entries.

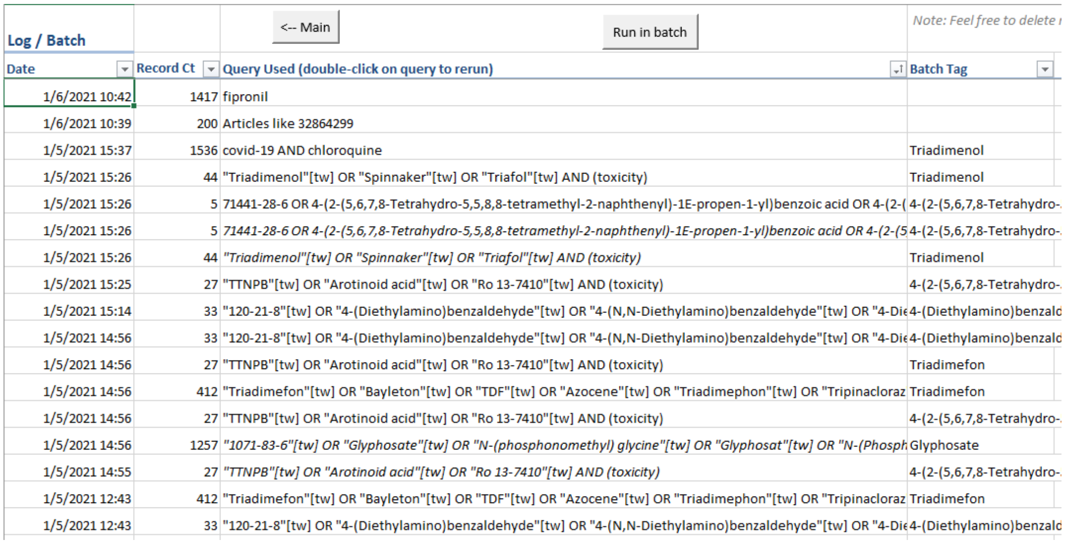

Date	Record Ct	Query Used (double-click on query to rerun)	Batch Tag
1/6/2021 10:42	1417	fipronil	
1/6/2021 10:39	200	Articles like 32864299	
1/5/2021 15:37	1536	covid-19 AND chloroquine	Triadimenol
1/5/2021 15:26	44	"Triadimenol"[tw] OR "Spinnaker"[tw] OR "Triafol"[tw] AND (toxicity)	Triadimenol
1/5/2021 15:26	5	71441-28-6 OR 4-(2-(5,6,7,8-Tetrahydro-5,5,8,8-tetramethyl-2-naphthenyl)-1E-propen-1-yl)benzoic acid OR 4-(2-(	4-(2-(5,6,7,8-Tetrahydro-.
1/5/2021 15:26	5	71441-28-6 OR 4-(2-(5,6,7,8-Tetrahydro-5,5,8,8-tetramethyl-2-naphthenyl)-1E-propen-1-yl)benzoic acid OR 4-(2-(5	4-(2-(5,6,7,8-Tetrahydro-.
1/5/2021 15:26	44	"Triadimenol"[tw] OR "Spinnaker"[tw] OR "Triafol"[tw] AND (toxicity)	Triadimenol
1/5/2021 15:25	27	"TTNPB"[tw] OR "Arotinoid acid"[tw] OR "Ro 13-7410"[tw] AND (toxicity)	4-(2-(5,6,7,8-Tetrahydro-.
1/5/2021 15:14	33	"120-21-8"[tw] OR "4-(Diethylamino)benzaldehyde"[tw] OR "4-(N,N-Diethylamino)benzaldehyde"[tw] OR "4-Die	4-(Diethylamino)benzald
1/5/2021 14:56	33	"120-21-8"[tw] OR "4-(Diethylamino)benzaldehyde"[tw] OR "4-(N,N-Diethylamino)benzaldehyde"[tw] OR "4-Die	4-(Diethylamino)benzald
1/5/2021 14:56	27	"TTNPB"[tw] OR "Arotinoid acid"[tw] OR "Ro 13-7410"[tw] AND (toxicity)	Triadimefon
1/5/2021 14:56	412	"Triadimefon"[tw] OR "Bayleton"[tw] OR "TDF"[tw] OR "Azocene"[tw] OR "Triadimephon"[tw] OR "Tripinacloraz	Triadimefon
1/5/2021 14:56	27	"TTNPB"[tw] OR "Arotinoid acid"[tw] OR "Ro 13-7410"[tw] AND (toxicity)	4-(2-(5,6,7,8-Tetrahydro-.
1/5/2021 14:56	1257	"1071-83-6"[tw] OR "Glyphosate"[tw] OR "N-(phosphonomethyl) glycine"[tw] OR "Glyphosat"[tw] OR "N-(Phosph	Glyphosate
1/5/2021 14:55	27	"TTNPB"[tw] OR "Arotinoid acid"[tw] OR "Ro 13-7410"[tw] AND (toxicity)	4-(2-(5,6,7,8-Tetrahydro-.
1/5/2021 12:43	412	"Triadimefon"[tw] OR "Bayleton"[tw] OR "TDF"[tw] OR "Azocene"[tw] OR "Triadimephon"[tw] OR "Tripinacloraz	Triadimefon
1/5/2021 12:43	33	"120-21-8"[tw] OR "4-(Diethylamino)benzaldehyde"[tw] OR "4-(N,N-Diethylamino)benzaldehyde"[tw] OR "4-Die	4-(Diethylamino)benzald

Figure 11. View of the Log sheet

Landscape sheet

The Landscape sheet provides an overview of the literature to the user for a set of entities, for example, a list of chemicals or genes. Figure 12 shows an example of a Landscape sheet built by a researcher interested in the toxicity of a particular set of chemicals. Let's take a look at that first. Queries designed to find the chemicals of interest are entered into Column C and in this case, a short version of the chemical name is in Column B. The queries in Row 3 are typical ones used in searching for articles about different kinds of chemical toxicity. We will refer to these queries as subject matter queries. (Note: Column A on the Landscape sheet is often hidden. Go ahead and unhide it and use it when you have a DSSTox chemical identifier.)

The premise behind the design of the Landscape sheet is very simple: PubMed queries will be built by taking the values in Column C (in this example chemical names and corresponding CAS numbers) and appending this query text to the subject matter query text in Row 3 with an " AND " in between the two query parts.

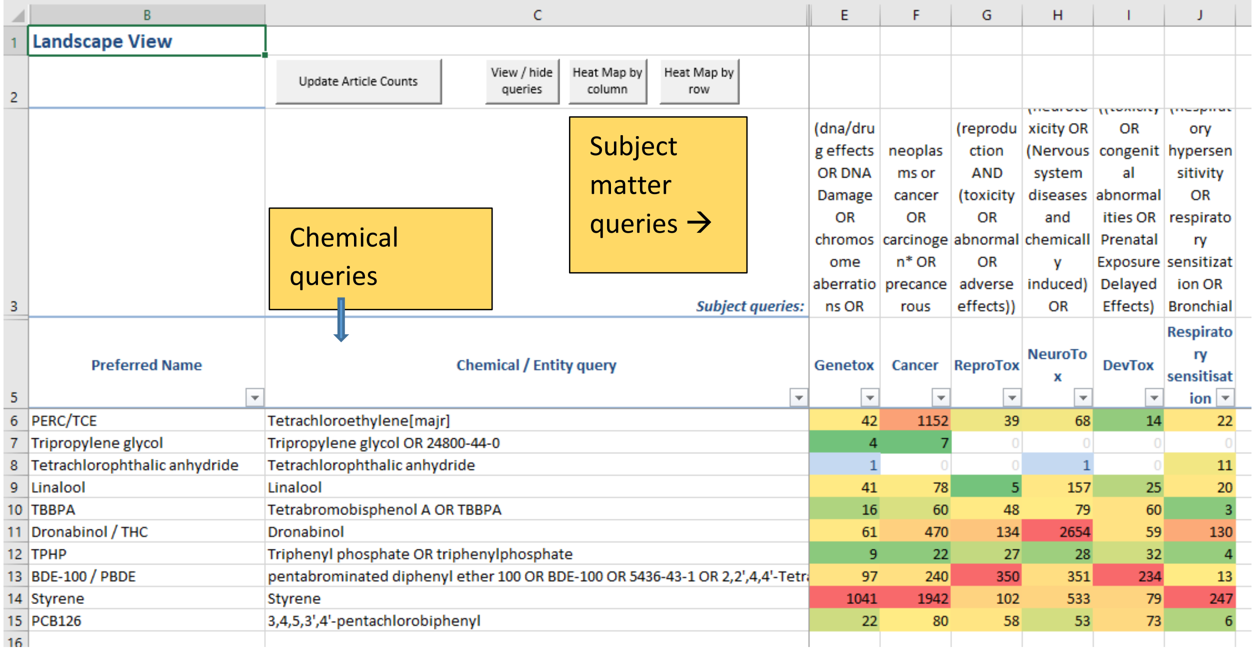

Figure 12. Example of Landscape sheet use

To illustrate, we will double-click on the cell with the arrow pointer in Figure 13. When we double-click on this cell this tells the Abstract Sifter to take the query text in Column C about Linalool and append it to query text designed to find citations about reproductive toxicity. Figure 14 shows the constructed query. We can then click on *Submit* and the query gets sent to PubMed and we can then see the results on the Main sheet. The number of articles retrieved from PubMed is 5. That count is placed in the corresponding Landscape cell that we just clicked on.

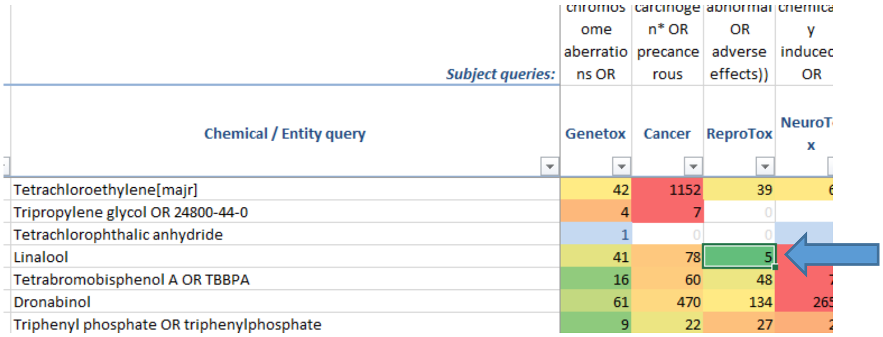

Figure 13. Double-click on article count cells

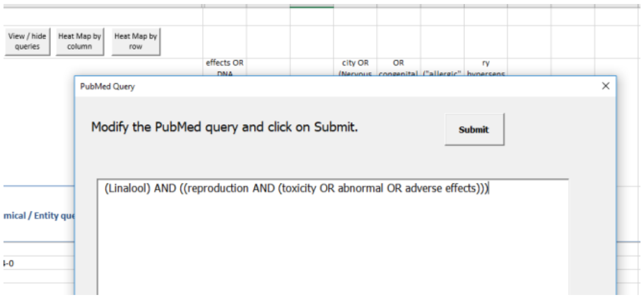

Figure 14. Constructed query

Now let's add to the Landscape sheet. Figure 15 shows how we added a new chemical to the list: aspirin. To find out the article counts for aspirin, select empty cells on the same row as aspirin, then click on *Update Article Counts* button. Excel will build each query from the aspirin part and the subject matter part and send each query to PubMed to find out how many citations satisfy the query. The article

counts are placed in the corresponding cells. To run the query and retrieve the results, just double-click on any of the article count cells.

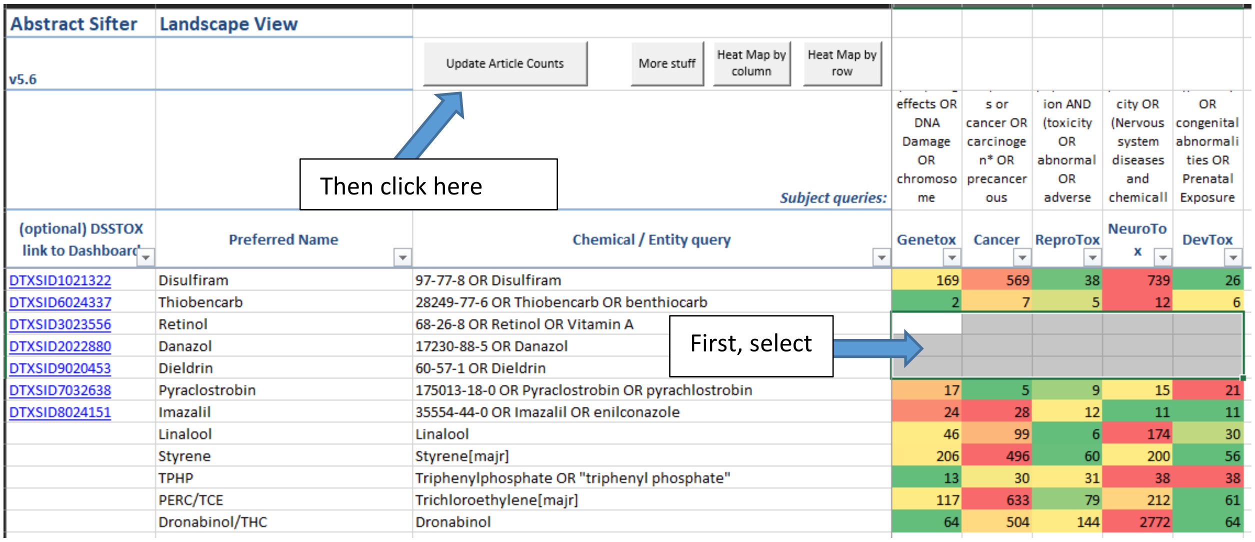

Figure 15. Adding rows to the Landscape sheet

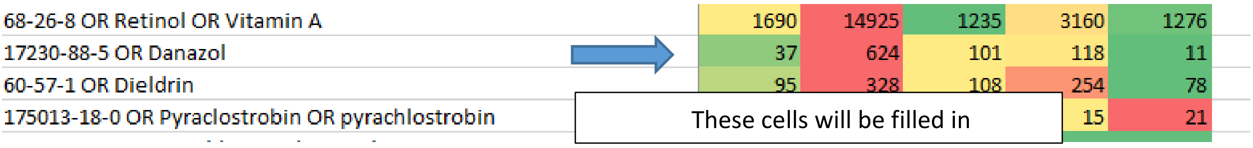

Figure 16. After clicking on Update Article Counts

New subject matter queries can be entered as well. The query part goes into Row 3 and a heading (of your choice) goes into Row 5. See the example below where the PubMed query part: skin OR dermatitis is entered with the heading skin. Next highlight the cells underneath and click on the *Update Article Counts* button. The counts of articles satisfying the queries are placed in the cells. What's happening behind the scenes? For each cell, a query is being built by the Abstract Sifter and sent to PubMed to retrieve a record count. That record count is then inserted into the corresponding cell. (Figure 17.)

Keep in mind that our examples revolve around chemicals, but that does not mean one is limited to chemicals. The entries in Column C and in Row 3 can be whatever you the end-user want them to be: genes, proteins, diseases, authors …

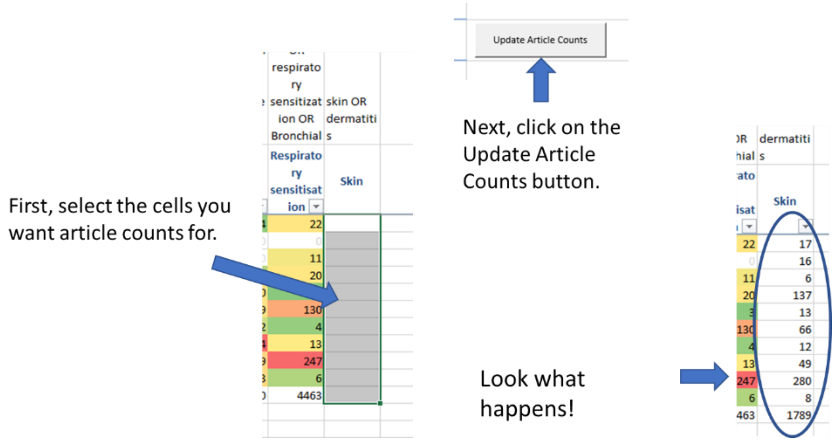

Figure 17. Steps for retrieving counts

Making things look good

The Landscape sheet has three buttons that make formatting easy (Figure 18). The heat map buttons will quickly apply heat map coloring to the cells with article counts either by column or by row. Try them out!

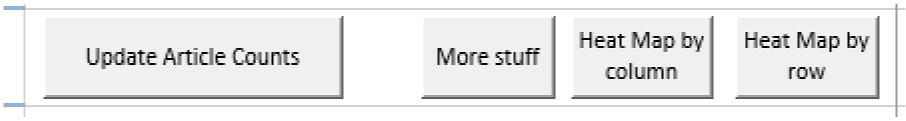

Figure 18. Buttons on the Landscape page include formatting actions.

Sample_Queries and Pathway_Queries Sheets

These two sheets function in a very similar way. We will use the Sample_queries sheet as an example in this user guide. Both sheets contain a number of sample subject matter queries that the end user can use as a starting point for building a Landscape view of a set of entities. Let's see how. First, we will clean off the old subject matter queries by deleting columns E-L on the Landscape sheet. (You can let the previous work stay if you wish.) Next, on the Sample_Queries sheet we will select rows with queries of interest then we click on the button *Send Queries to Landscape* (Figure 19).

Sample Queries | *Note: these are starting points ... please expand and customize* | Send queries to Landscape

Category	Heading	Query (double-click to see how the query looks to PubMed)
Methods	In vitro	In Vitro Techniques[mh] OR cell culture or "in vitro"
Mixtures	Mixtures	(Drug synergism[mh] OR cocarcinogenesis OR pesticide synergists[mh] OR mixture[tiab] OR mixtures[tiab] OR Drug Antagonism[n
Medicine	Clinical trials	((clinical[Title/Abstract] AND trial[Title/Abstract]) OR clinical trial[Publication Type])
Medicine	Clinical trials in children	((children OR child OR infants) AND human) AND ((clinical[Title/Abstract] AND trial[Title/Abstract]) OR clinical trial[Publication Typ
Medicine	Obesity	(obesity OR obese OR adipose OR overweight Or adipogenesis OR adipose tissue)
Toxicity	Genetox	(dna/drug effects OR DNA Damage OR chromosome aberrations OR genotoxicity OR micronucleus OR DNA Repair OR mutagenicity
Toxicity	Cancer	neoplasms or cancer OR carcinogen* OR precancerous
Toxicity	ReproTox	(reproduction AND (toxicity OR abnormal OR adverse effects))
Toxicity	NeuroTox	(neurotoxicity OR (Nervous system diseases and chemically induced) OR ((neurons OR brain OR behavior) AND drug effects)
Toxicity	DevTox	((toxicity OR congenital abnormalities OR Prenatal Exposure Delayed Effects) AND (fetus OR embryo OR embryonic development C
Toxicity	Skin sensitization	("allergic" AND "contact" And dermatitis) OR Dermatitis, Allergic Contact[mh]
Toxicity	Respiratory sensitisation	(Respiratory hypersensitivity OR respiratory sensitization OR Bronchial Hyperreactivity OR Respiration Disorders OR Respiratory Tra
Toxicity	DNT	((Brain OR central nervous system OR "CNS" OR "neural tube" OR spinal cord OR spina bifida OR Nervous System Diseases or Neura
Use	Pharmaceutical	"therapeutic use" OR "therapeutic use"[subheading]" OR pharmacologic actions[mh] OR drug therapy
Use	Pesticide	pesticide OR insecticide OR rodenticide OR fungicide
Use	Cosmetics	cosmetics OR beauty
Use	Explosive Agents	Explosive Agents OR explosive OR explosives
Use	Food	food OR diet OR beverage OR nutrition OR eating
Use	Surface-acting	Antifoaming OR Anti-foaming OR detergent OR detergents OR soap OR detergent OR surfactant
Use	Dye/coloring	dye OR "coloring agent" OR pigment OR pigments
Use	Fertilizer	fertilizer OR fertilize
[illegible]	[illegible]	[illegible]

Figure 19. Selecting rows with queries of interest

Our Landscape sheet then looks like Figure 20.

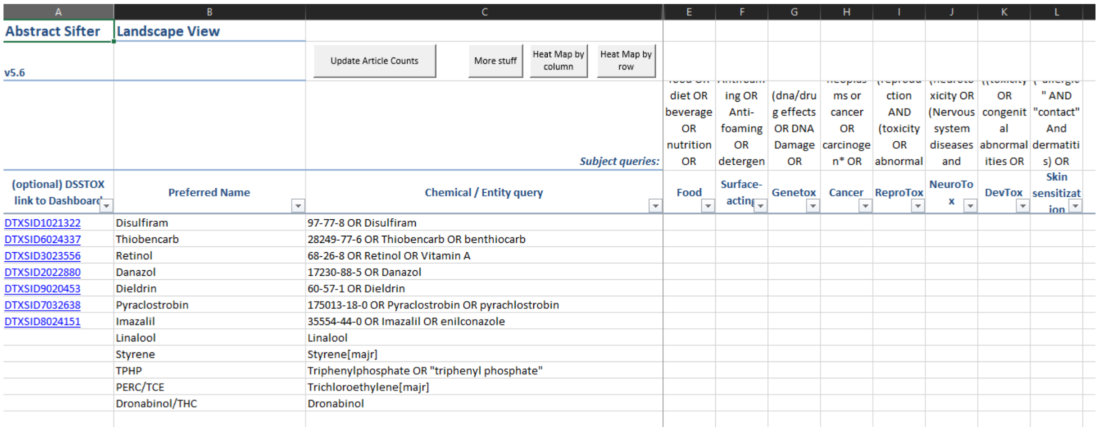

A	B	C	E	F	G	H	I	J	K	L
Abstract Sifter	Landscape View									
v5.6		Update Article Counts / More stuff / Heat Map by column / Heat Map by row								
		Subject queries:	diet OR beverage OR nutrition OR	ing OR Anti-foaming OR detergen	(dna/dru g effects OR DNA Damage OR	ms or cancer OR carcinoge n* OR	ction AND (toxicity OR abnormal	xicity OR (Nervous system diseases and	OR congenit al abnormal ities OR	" AND "contact" And dermatiti s) OR
(optional) DSSTOX link to Dashboard	Preferred Name	Chemical / Entity query	Food	Surface-acting	Genetox	Cancer	ReproTox	NeuroTox	DevTox	Skin sensitization
DTXSID1021322	Disulfiram	97-77-8 OR Disulfiram								
DTXSID6024337	Thiobencarb	28249-77-6 OR Thiobencarb OR benthiocarb								
DTXSID3023556	Retinol	68-26-8 OR Retinol OR Vitamin A								
DTXSID2022880	Danazol	17230-88-5 OR Danazol								
DTXSID9020453	Dieldrin	60-57-1 OR Dieldrin								
DTXSID7032638	Pyraclostrobin	175013-18-0 OR Pyraclostrobin OR pyrachlostrobin								
DTXSID8024151	Imazalil	35554-44-0 OR Imazalil OR enilconazole								
	Linalool	Linalool								
	Styrene	Styrene[majr]								
	TPHP	Triphenylphosphate OR "triphenyl phosphate"								
	PERC/TCE	Trichloroethylene[majr]								
	Dronabinol/THC	Dronabinol								

Figure 20. New queries on Landscape sheet

Next, we select the article count area and then click on *Update Article Counts*.

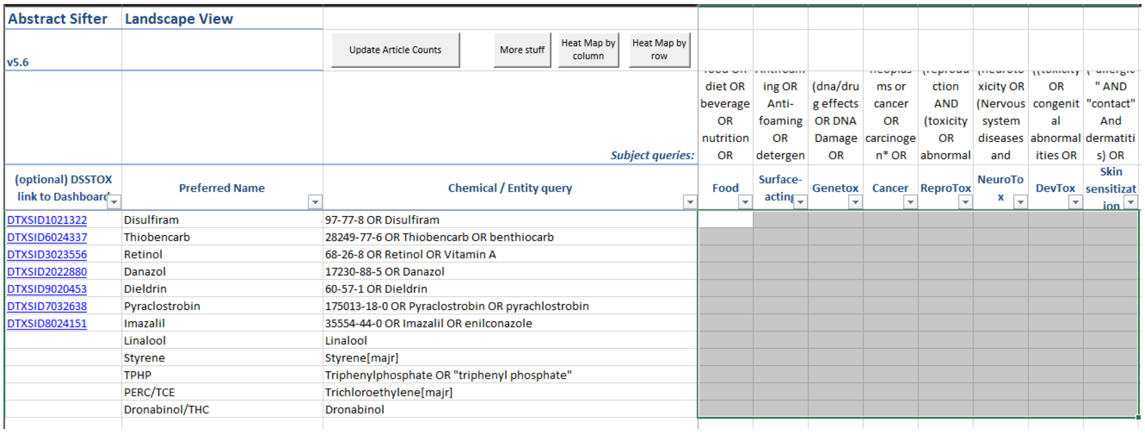

Abstract Sifter	Landscape View									
v5.6		Update Article Counts / More stuff / Heat Map by column / Heat Map by row								
		Subject queries:	diet OR beverage OR nutrition OR	ing OR Anti-foaming OR detergen	(dna/dru g effects OR DNA Damage OR	ms or cancer OR carcinoge n* OR	ction AND (toxicity OR abnormal	xicity OR (Nervous system diseases and	OR congenit al abnormal ities OR	" AND "contact" And dermatiti s) OR
(optional) DSSTOX link to Dashboard	Preferred Name	Chemical / Entity query	Food	Surface-acting	Genetox	Cancer	ReproTox	NeuroTox	DevTox	Skin sensitization
DTXSID1021322	Disulfiram	97-77-8 OR Disulfiram								
DTXSID6024337	Thiobencarb	28249-77-6 OR Thiobencarb OR benthiocarb								
DTXSID3023556	Retinol	68-26-8 OR Retinol OR Vitamin A								
DTXSID2022880	Danazol	17230-88-5 OR Danazol								
DTXSID9020453	Dieldrin	60-57-1 OR Dieldrin								
DTXSID7032638	Pyraclostrobin	175013-18-0 OR Pyraclostrobin OR pyrachlostrobin								
DTXSID8024151	Imazalil	35554-44-0 OR Imazalil OR enilconazole								
	Linalool	Linalool								
	Styrene	Styrene[majr]								
	TPHP	Triphenylphosphate OR "triphenyl phosphate"								
	PERC/TCE	Trichloroethylene[majr]								
	Dronabinol/THC	Dronabinol								

Figure 21. Selecting the cells for article counts

Once the article counts are populated, we click on Heat Map by Row and then on Hide queries. Our resulting Landscape view looks like Figure 22. To run the query and retrieve the results, just double-click on any of the article count cells.

Note that the Pathway_queries sheet works the same way: select the rows of interest and click on the button *Send queries to Landscape*. To add your own queries to either the pathway or sample queries sheets, just enter in new rows. Use the existing rows as a template to indicate where headers go and where the query text goes. Be mindful of parentheses and be sure to test the queries.

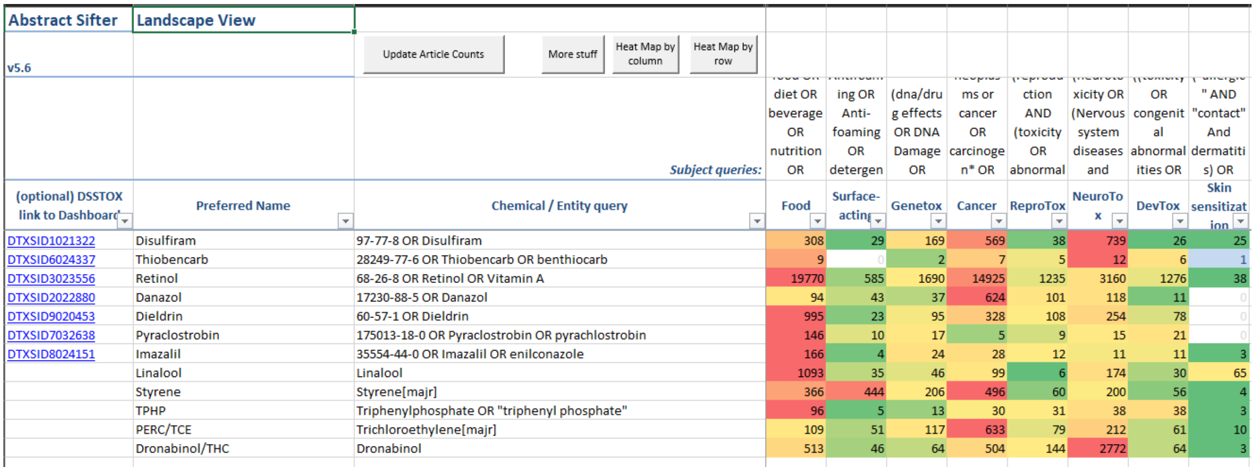

Abstract Sifter	Landscape View									
v5.6		Update Article Counts / More stuff / Heat Map by column / Heat Map by row								
		Subject queries:	diet OR beverage OR nutrition OR	ing OR Anti-foaming OR detergen	(dna/drug effects OR DNA Damage OR	ms or cancer OR carcinogen* OR	ction AND (toxicity OR abnormal	xicity OR (Nervous system diseases and	OR congenital abnormalities OR	" AND "contact" And dermatitis) OR
(optional) DSSTOX link to Dashboard	Preferred Name	Chemical / Entity query	Food	Surface-acting	Genetox	Cancer	ReproTox	NeuroTox	DevTox	Skin sensitization
DTXSID1021322	Disulfiram	97-77-8 OR Disulfiram	308	29	169	569	38	739	26	25
DTXSID6024337	Thiobencarb	28249-77-6 OR Thiobencarb OR benthiocarb	9	0	2	7	5	12	6	1
DTXSID3023556	Retinol	68-26-8 OR Retinol OR Vitamin A	19770	585	1690	14925	1235	3160	1276	38
DTXSID2022880	Danazol	17230-88-5 OR Danazol	94	43	37	624	101	118	11	0
DTXSID9020453	Dieldrin	60-57-1 OR Dieldrin	995	23	95	328	108	254	78	0
DTXSID7032638	Pyraclostrobin	175013-18-0 OR Pyraclostrobin OR pyrachlostrobin	146	10	17	5	9	15	21	0
DTXSID8024151	Imazalil	35554-44-0 OR Imazalil OR enilconazole	166	4	24	28	12	11	11	3
	Linalool	Linalool	1093	35	46	99	6	174	30	65
	Styrene	Styrene[majr]	366	444	206	496	60	200	56	4
	TPHP	Triphenylphosphate OR "triphenyl phosphate"	96	5	13	30	31	38	38	3
	PERC/TCE	Trichloroethylene[majr]	109	51	117	633	79	212	61	10
	Dronabinol/THC	Dronabinol	513	46	64	504	144	2772	64	3

Figure 22. New Landscape view

TermMap Sheet

Term mapping is a new functionality that is motivated by the following questions that some users have asked.

- There is a set of terms I always sift on to find articles relevant to my work. Can I somehow sift on them all at once?
- I am doing research on ocean warming and its effect on fish. I have a list of over 100 fish species I am interested in. Can I find the citations quickly that have any of these fish? And because my species are in a family ontology, can I sift on the family name, too?
- Can I map genes of interest to a specialized ontology?

The Term Map has built in examples. Let's walk through one of them here.

On the Main sheet, run the query: coral AND (ocean warming OR climate change) This query will return approximately 1750 records. Then go to the Term Map sheet and click on More. (The TermMap sheet may have stuff on it – just ignore.) A form is displayed that looks like the one in Figure 23. Click on the Coral Ontology button. (Check out the other examples later!)

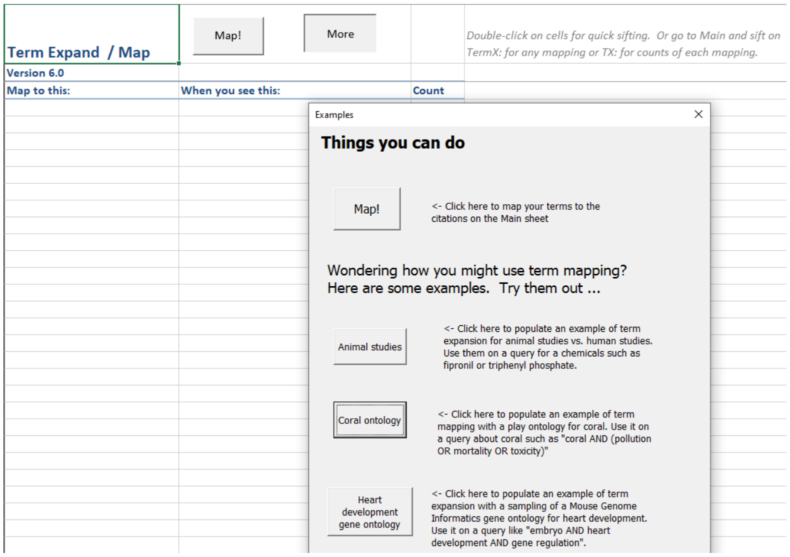

Figure 23. TermMap sheet More button actions

The TermMap sheet is then populated with what might be a coral ontology. Please note – this is NOT a real ontology. It is made up to be an illustration of what you can do. The coral names may be real, but the color categories are completely made up. Next, click on the Map! Button. After a few seconds you will see in numbers appear in Column C. (Figure 24.) These are counts of the number of citations from Main that contain the term in Column B. The term *zoanthid* is in 2 articles, *Acropora millepora* in 45.

Term Expand / Map Map! More

Version 6.0

**Map to this:**	**When you see this:**	**Count**
Coral	Anthozoa	923
Coral:red	Pocillopora damicornis	54
Coral:red	Acropora millepora	46
Coral:red	Stylophora pistillata	39
Coral:red	zoanthid	2
Coral:red	M. annularis	2
Coral:red	Acropora muricata	12
Coral:red	Porites lutea	8
Coral:red	Colpophyllia natans	2
Coral:red	Seriatopora hystrix	9
Coral:red	Anthropocene	23
Coral:red	brain coral	4
Coral:red	Orbicella annularis	3
Coral:red	A. muricata	4
Coral:red	Meandrina meandrites	1
Coral:red	Diploria labyrinthiformis	4
Coral:red	Pseudodiploria strigosa	10
Coral:red	Agaricia agaricites	1
Coral:red	Porites porites	1
Coral:blue	P. verrucosa	1
Coral:blue	Ctenactis echinata	1
Coral:blue	P. eydouxi	0
Coral:blue	Eusmilia fastigiata	1
Coral:blue	Isopora palifera	2
Coral:pink	Dichocoenia stokesi	1

Figure 24. TermMap sheet after Map! Action has counted occurrences of the terms.

The Abstract Sifter did more than just count the articles where it found the terms. The Sifter changed the abstract for each citation in which at least one of the terms was found. (Of course, the abstract is changed only in the Sifter, not at PubMed. Rerunning the query will refresh the abstracts back to their original state.)

Here's how the abstract is modified: if any of the column B terms was found in the title and abstract, then "TERMX:" was appended to the abstract. For each column B term found, "TX: " and the column A value plus the Column B value were stuck on the end of the abstract.

To see an example that will make this clearer … see Figure 25 below of an abstract on the Abstract Sheet. At the end you see "TERMX: |TX:Coral:Anthozoa |TX:Coral:red:Seriatopora hystrix"

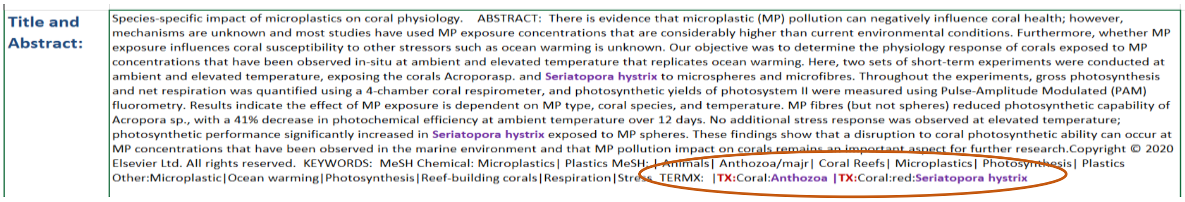

Title and Abstract:	Species-specific impact of microplastics on coral physiology. ABSTRACT: There is evidence that microplastic (MP) pollution can negatively influence coral health; however, mechanisms are unknown and most studies have used MP exposure concentrations that are considerably higher than current environmental conditions. Furthermore, whether MP exposure influences coral susceptibility to other stressors such as ocean warming is unknown. Our objective was to determine the physiology response of corals exposed to MP concentrations that have been observed in-situ at ambient and elevated temperature that replicates ocean warming. Here, two sets of short-term experiments were conducted at ambient and elevated temperature, exposing the corals Acroporasp. and **Seriatopora hystrix** to microspheres and microfibres. Throughout the experiments, gross photosynthesis and net respiration was quantified using a 4-chamber coral respirometer, and photosynthetic yields of photosystem II were measured using Pulse-Amplitude Modulated (PAM) fluorometry. Results indicate the effect of MP exposure is dependent on MP type, coral species, and temperature. MP fibres (but not spheres) reduced photosynthetic capability of Acropora sp., with a 41% decrease in photochemical efficiency at ambient temperature over 12 days. No additional stress response was observed at elevated temperature; photosynthetic performance significantly increased in **Seriatopora hystrix** exposed to MP spheres. These findings show that a disruption to coral photosynthetic ability can occur at MP concentrations that have been observed in the marine environment and that MP pollution impact on corals remains an important aspect for further research.Copyright © 2020 Elsevier Ltd. All rights reserved. KEYWORDS: MeSH Chemical: Microplastics| Plastics MeSH: | Animals| Anthozoa/majr| Coral Reefs| Microplastics| Photosynthesis| Plastics Other:Microplastic|Ocean warming|Photosynthesis|Reef-building corals|Respiration|Stress TERMX: |**TX:**Coral:**Anthozoa** |**TX:**Coral:red:**Seriatopora hystrix**

Figure 25. How term mapping changes the abstract. See the circled area.

Two of the coral column B terms were found and appended along with their Column A values Coral and Coral:red.

In the Abstract Sifter, if text exists in the abstract column that means it can be sifted on! There are three new ways to sift if you map terms.

1. On the Main sheet, in cell B3, C3, or D3 - Sift on *TERMX:* … this will show you any citation that has a least one of the column B terms.
2. On the Main sheet, cell B3, C3, or D3 - Sift on *TX:* … this will count the number of terms from column B found in the citation.
3. Sift on a value in column A or column B. To do this quickly, stay on the TermMap sheet and double-click on a term in either A or B and see what happens. (What does happen? The Sifter copies the term to the Main sheet cell B3, lets the Main sheet sift the results, and then sorts by B3. It happens fast, but it is very simple.)

Exporting to other applications from the Notes sheet

The Abstract Sifter allows the user to export articles from the Notes sheet to outside applications. On the Notes sheet there is a button labeled *Export*. By clicking on this button, the form in Figure 26 appears. The first set of radio buttons allows the user to select what data is exported and how the records should be formatted. Next the user can choose to export all entries / rows on the Notes sheet or just selected rows. If the end-user selects PMIDs to be exported, the PMIDs will be formatted in the box. In this case, clicking on Next Step will copy the formatted PMIDs to the clipboard, ready to be pasted to the next application. They should then be pasted into the destination. In the case of PubMed, they should be pasted into the query box in PubMed Entrez. (Figure 27) From PubMed, the citations can be downloaded in a variety of formats, including a format that can be imported into citation management software (Figures 28 and 29).

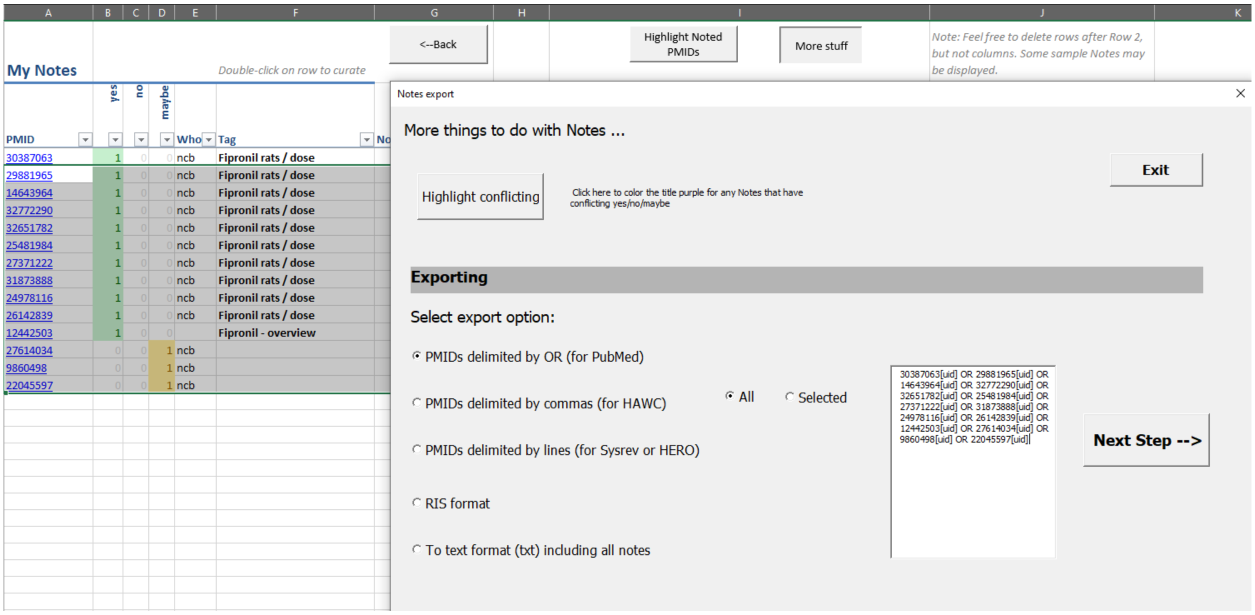

Figure 23. Form that appears after clicking on Export button

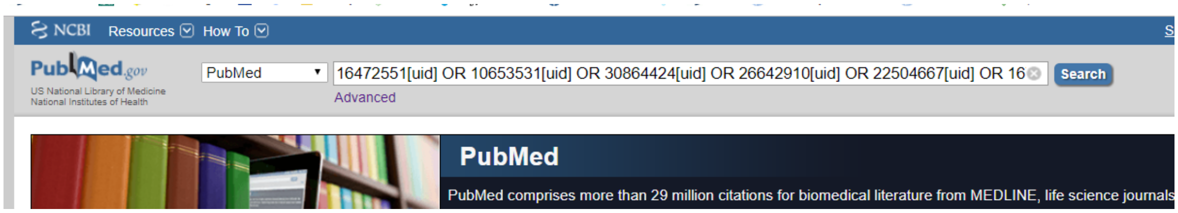

Figure 24. Paste PMIDs in the query box to retrieve the records.

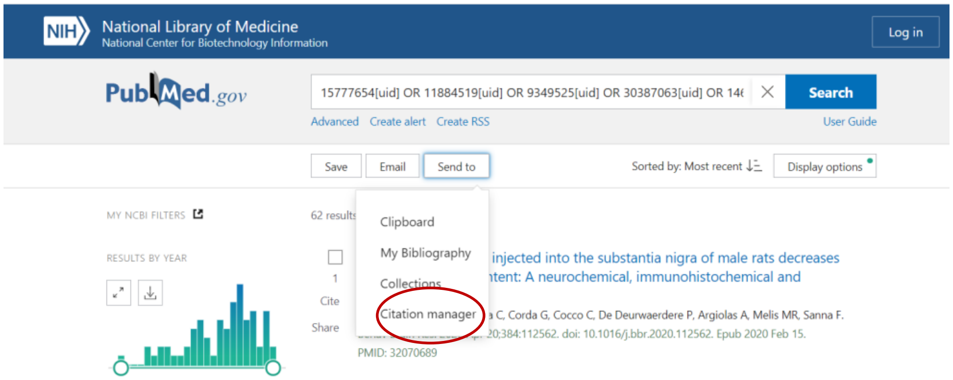

Figure 24. In PubMed, click on Send to

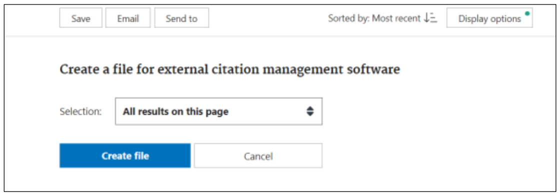

Figure 25. Dialog box for exporting to citation manager

When the user clicks on the Create File button, a file is created and downloaded in nbib format and can be imported into most common reference manager software.

If the end-user specifies RIS format or text format, files will be created. The user will specify the location and name of the files. The text file option includes the abstract, tags, and notes.

Helpful Tips and Guidelines

Tip 1 – checking quality of results

The Landscape sheet is a great way to explore a set of chemicals, but some chemical names are long, complex, and a challenge to PubMed. If you copy and paste a chemical name from another source, make sure it does not have any special characters. Non printing escape characters make the web service calls give unexpected results, but the PubMed web site knows how to ignore them.

For other types of errors or strange results, it's a good idea to check it in PubMed. You can take any query generated by the Abstract Sifter and copy and paste it into PubMed using Ctl-C to copy and Ctl-V to paste. For example, the query in the box shown in Figure 29 is selected and copied (with Ctl-C). Then in PubMed the query is pasted into the query line at the top as shown in Figure 30. On the right side of the page is a box entitled *Search Details*. Click on the *See More* … link to expand this box. Figure 31 shows the information provided by PubMed about how it expands the query. If you need to learn more about PubMed queries, click on *Help* on the PubMed home page.

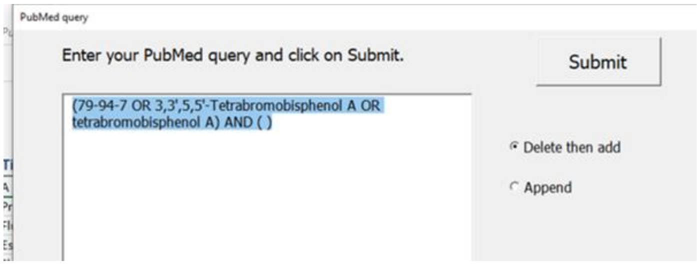

Figure 29. Select and Ctl-C to copy

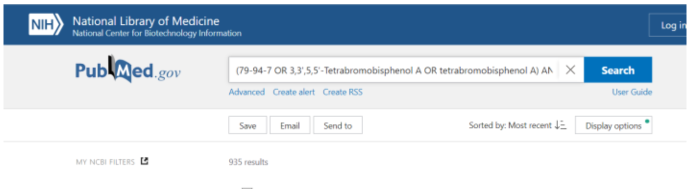

Figure 30. Ctl-V to paste in PubMed then search

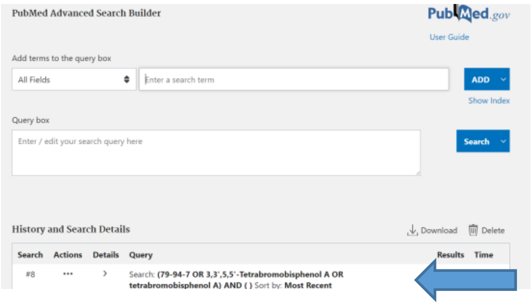

Figure 26. See what PubMed does to expand your search

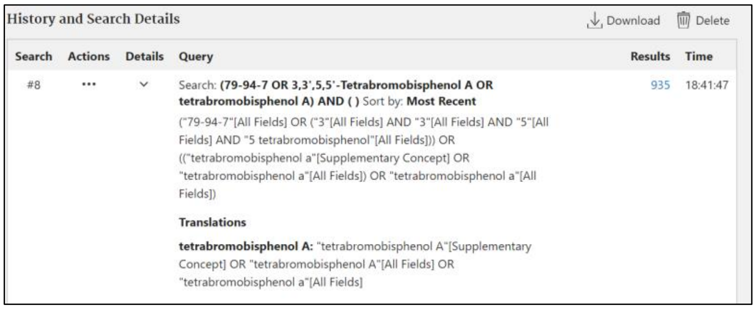

Figure 32. PubMed query breakdown and expansion

Note that after PubMed rolled out its new version in 2020, not just the looks of the site changed. They redid the search algorithms. As of this writing, the web service had not changed to use the new search methods – that means for the first time we are noticing small differences in the returned record counts.

Tip 2 – Sifting the chemical literature

It can be very helpful in chemical research to include the chemical name in the sifting process. This is because a chemical can be mentioned in an abstract even in cases where the article is not really about the chemical and will be retrieved in the PubMed query (depending on how the query is worded). Counting the occurrences of the chemical name in the abstract through the sifting process can help the user discriminate between articles mentioning a chemical or those that are actually about the chemical.

Tip 3 – cleanup and customization

The Abstract Sifter can be cleaned up by deleting rows and columns from previous work, but the Abstract Sifter programming requires certain columns and rows to be in certain places. To learn how to clean up your sifter without disrupting the behind-the-scenes coding, consult the table below.

**Sheet name**	**Advice for cleaning**
Main	Do not add columns. Rows will be added and deleted by the Sifter.
Abstract	Do not add or delete rows or columns. The Sifter software updates this sheet.
Notes	Delete any unwanted rows after Row 2. Do not add or delete columns.
Log	Delete any unwanted rows after Row 2. Do not delete columns. Add columns after G if desired.
Sample_Queries	Delete unwanted rows after Row 3. Modify and add rows as desired, following the pattern of current rows. (That is, keep the heading in column B and the query text in column C.) *Hint: use this sheet to keep queries important to your organization.*
Pathway_queries	Delete unwanted rows after Row 3. Modify and add rows as desired, following the pattern of current rows.
Landscape	Delete or modify rows after Row 4 and columns after Column D.

CuratedLists	Delete or modify rows after Row 3 and columns after Column C.
TermMap	Delete or modify rows after Row 3.

Keep in mind that the Abstract Sifter is an Excel file. You can rename it, mail it, and of course, if you want to keep your Log, Notes, and Landscape entries, you should save it. The Sample_queries sheet provides an opportunity for you and your organization to start collecting and organizing queries that you have found useful. As mentioned earlier, feel free to hide any sheets you don't use to keep your workplace streamlined.

Tip 4 – Collaborative literature review tips

Sometimes more than one person will want to work together on evaluating a set of articles. The Abstract Sifter has some features to make this easier. So, let's say Mary and Joe each retrieve, sift, and take notes on their own Abstract Sifter files. Mary can copy Joe's notes to her version of the Sifter and then she has both sets. (Or they can mail the Sifter back and forth …)

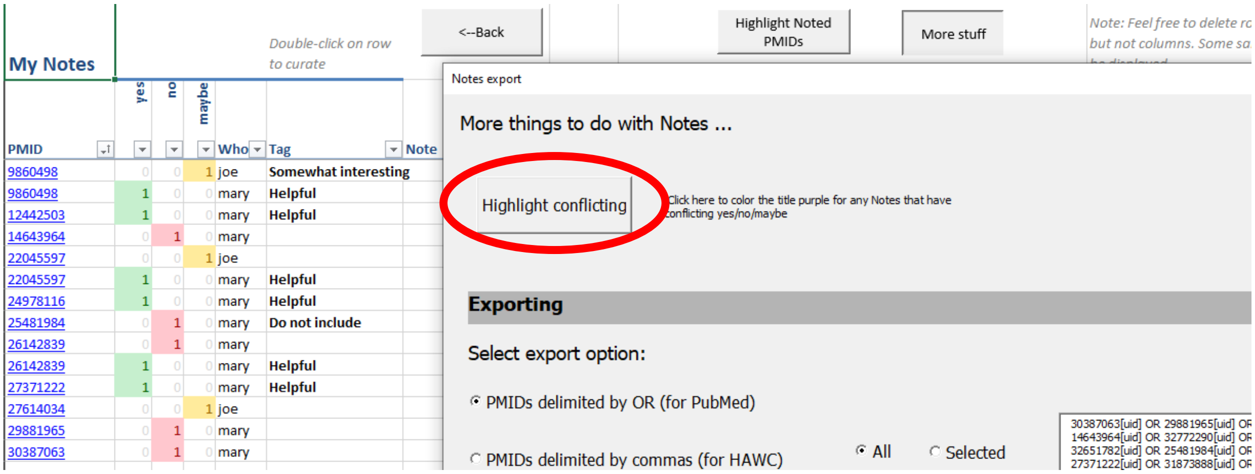

Figure 33. Notes from two reviewers combined on the Notes sheet.

To see if she and Joe disagreed on any record, she can click on More Stuff then Highlight Conflicting. The titles of Notes with different yes/no/maybe designations are colorized in purple. Resolving the conflicts and re-clicking on the button will cause the purple to disappear.

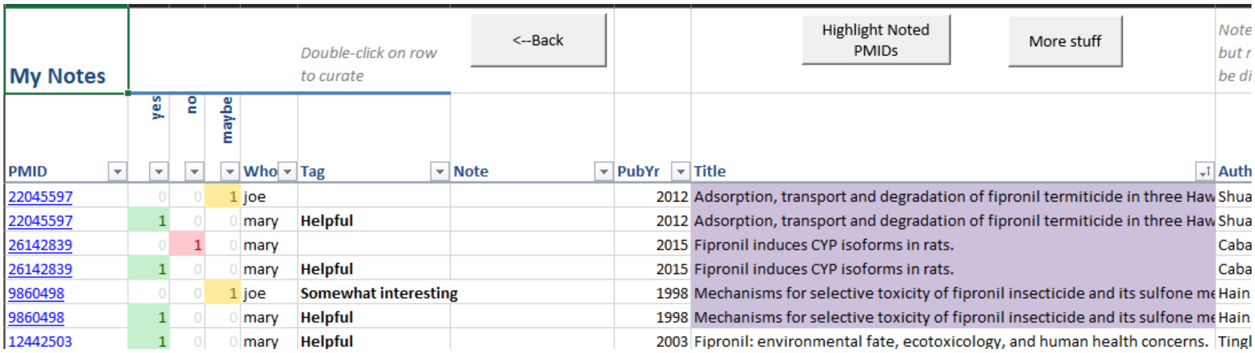

PMID	yes	no	maybe	Who	Tag	Note	PubYr	Title	Auth
22045597	0	0	1	joe			2012	Adsorption, transport and degradation of fipronil termiticide in three Haw	Shua
22045597	1	0	0	mary	Helpful		2012	Adsorption, transport and degradation of fipronil termiticide in three Haw	Shua
26142839	0	1	0	mary			2015	Fipronil induces CYP isoforms in rats.	Caba
26142839	1	0	0	mary	Helpful		2015	Fipronil induces CYP isoforms in rats.	Caba
9860498	0	0	1	joe	Somewhat interesting		1998	Mechanisms for selective toxicity of fipronil insecticide and its sulfone me	Hain
9860498	1	0	0	mary	Helpful		1998	Mechanisms for selective toxicity of fipronil insecticide and its sulfone me	Hain
12442503	1	0	0	mary	Helpful		2003	Fipronil: environmental fate, ecotoxicology, and human health concerns.	Tingl

Figure 34. Purple highlighting on title of conflicting notes.

Tip 5 – Connections to the EPA Chemicals Dashboard

The Environmental Protection Agency's Chemicals Dashboard is a great place to find chemical information to enhance your chemical search queries with synonyms and CAS numbers. Future releases of the Dashboard will offer opportunities to download a list of chemicals formatted for easy insertion into the Landscape sheet. You'll find the Chemistry Dashboard here: https://comptox.epa.gov/dashboard.

The EPA Chemicals Dashboard also contains its own (slightly different) version of the Abstract Sifter. It works on the same basic premise as the Excel version, but has some interesting differences. To see it, start with a chemical search. Let's look at the chemical fipronil by entering the name in the search box and clicking on the search icon (magnifying glass) (Figure 35).

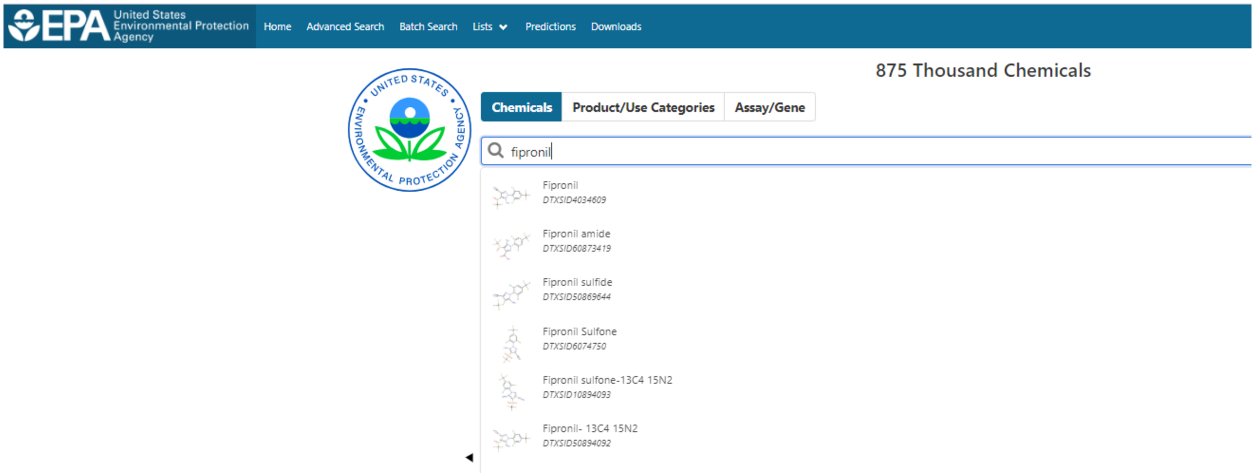

Figure 35. Searching for fipronil on the EPA's Chemistry Dashboard entry form

The main page for fipronil is displayed with the structure diagram and a selection of tabs below that lead to other information about the chemical. Click on the Literature tab as shown in Figure 33.

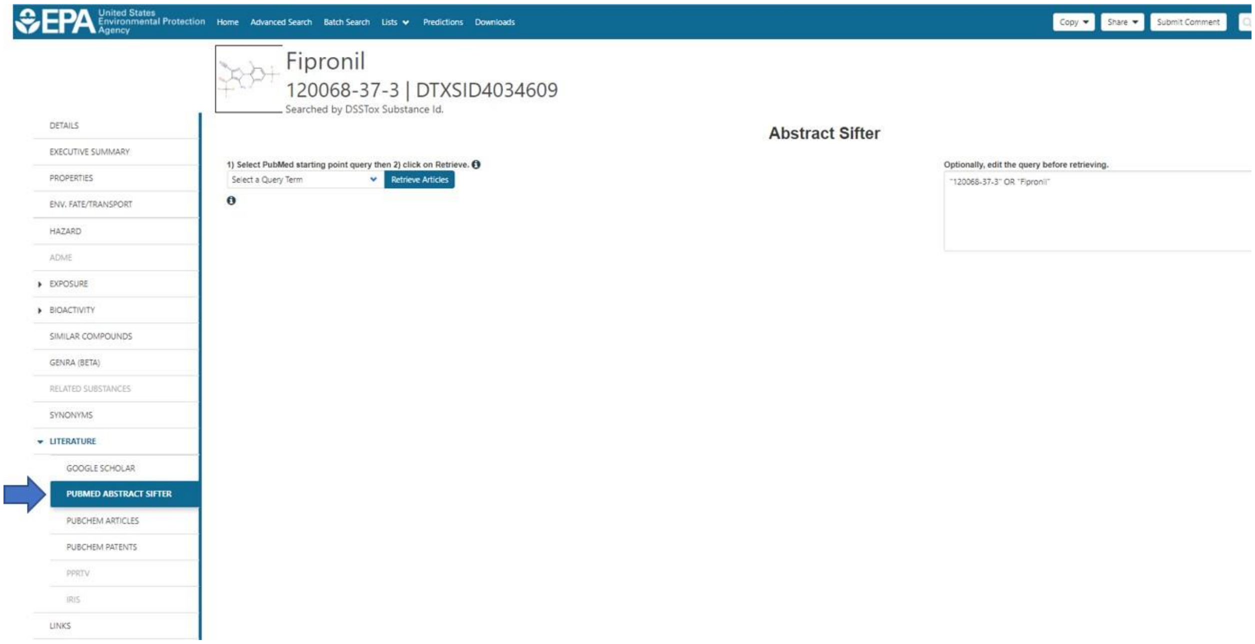

Figure 36. Select the Literature tab then on PubMed Abstract Sifter (see below).

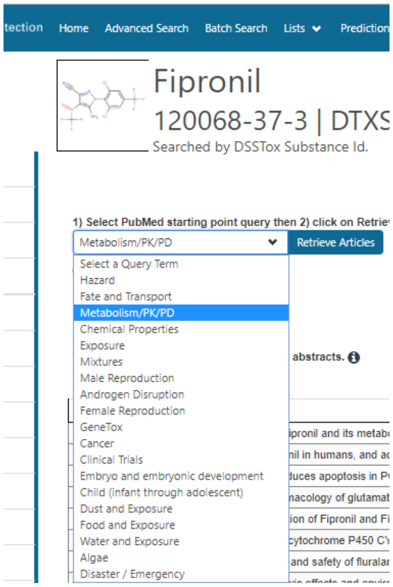

Figure 37. How to select prepared queries.

Select PubMed Abstract Sifter on the left set of buttons. The Dashboard helps you to build queries for this chemical. The chemical identifier part of the query is prepopulated on the right with name and CAS number. The subject matter part of the query is determined by selecting a topic area in the pull-down box in the center of the form. The user has several pre-composed queries to choose from. When one of them is chosen, the query is modified by appending the subject matter text. Figure 37 shows that when Metabolism/PK/PD is chosen, the text (metabolism OR metabolite OR tissue distribution OR pharmacokinetics OR pharmacodynamics) is appended to the chemical identifiers. The query can be modified manually as well. When ready, the user clicks on Retrieve Articles.

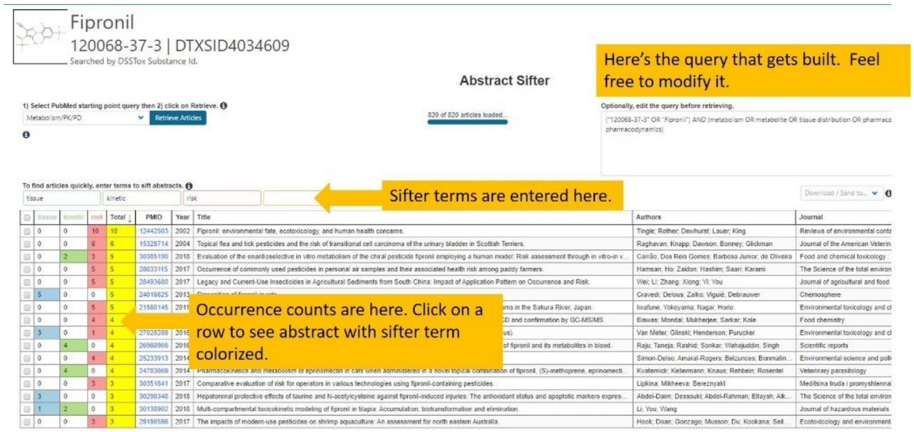

Figure 38. Sifting on the EPA Chemicals Dashboard's PubMed Abstract Sifter.

After the user clicks on Retrieve Articles, the article information is retrieved from PubMed and inserted into the results table. The articles can be sifted by entering terms into the boxes shown. In the example in Figure 38, the user has entered tissue into one box and kinetic into the other. The occurrences of these terms are counted for each PubMed citation and displayed. The table can be sorted on these values. Clicking on a row tells the Dashboard Sifter to display the title and abstract below the table with the sifter terms highlighted.

A check box on the left of the table provides a way to select citation rows. Selected rows can be downloaded or sent to PubMed by clicking on the pull-down box to the right.

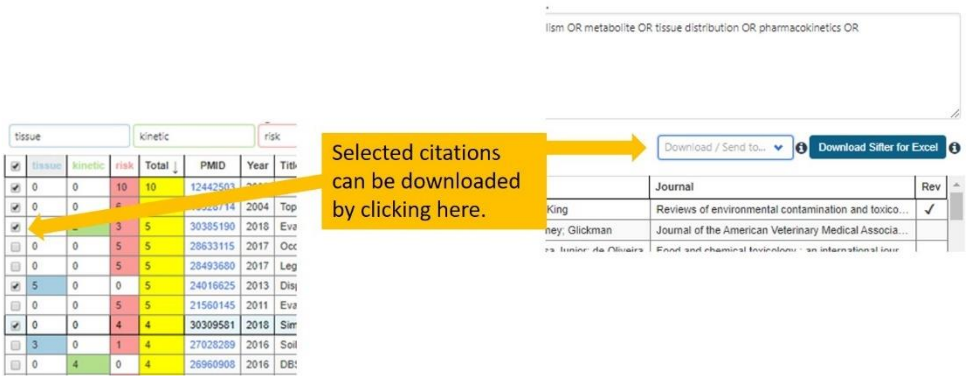

Figure 39. How to select and download citations.

Notice, too, that the blue button above lets the user download the Excel version of the Abstract Sifter. This button will always download the most up-to-date version. Check back periodically with the EPA's Chemicals Dashboard to learn about enhancements to PubMed Abstract Sifter.

Populating the Excel Abstract Sifter from the EPA CompTox Chemicals Dashboard

The Chemicals Dashboard can download chemicals in a variety of formats. One of those formats make it easy to use in the Excel Abstract Sifter. Here's an example to get you started. On the home page of the Dashboard, click on Lists, then chemical (Figure 40.)

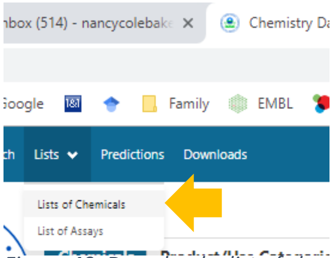

Figure 40. Download a list of chemicals.

Pick a list. We'll pick Algal Toxins as a sample. Click on the name, then, when the chemicals appear, click on Send to Batch Search. (Figure 41.)

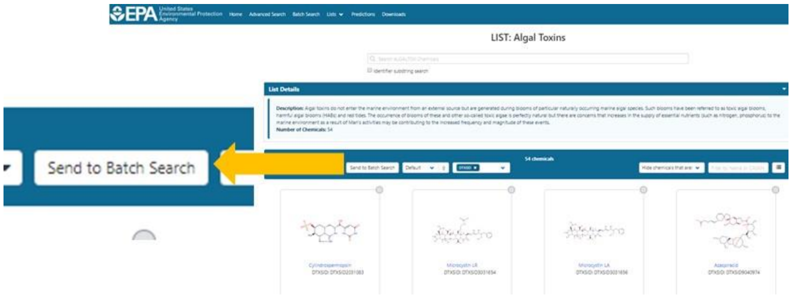

Figure 41. Send a list of chemicals to Batch Search.

The batch search page looks like Figure 41.

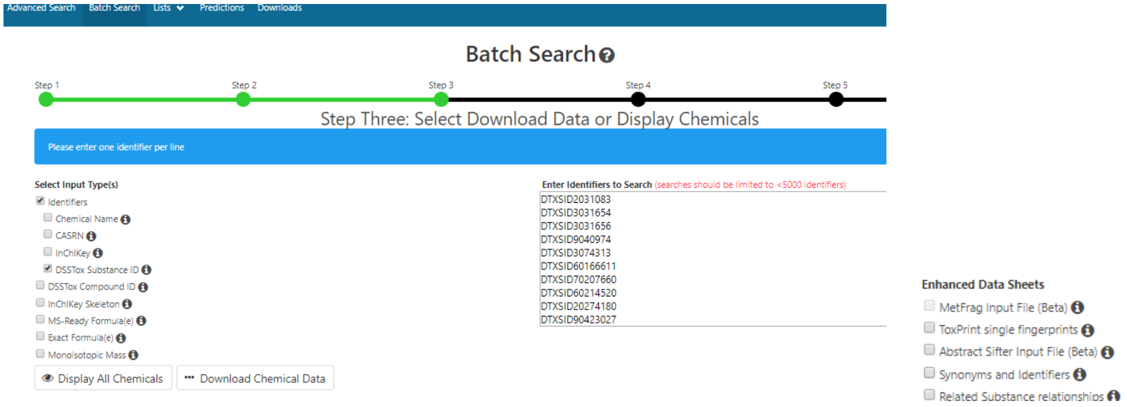

Figure 42. Send a list of chemicals to Batch Search.

Click on the following: Download Chemical Data, then Download as Excel, then Abstract Sifter Input File (Beta), then (finally) the Download bar. This action will download the chemicals to Excel. Open that file. It will have 2 sheets. Open the one that is called Abstract Sifter. It looks like Figure 43. On the Abstract Sifter Landscape sheet, unhide column A. This is done by clicking on the left border of Column B, then right-clicking to see the menu where you can click on *Unhide*. Paste rows from the downloaded spreadsheet onto the Landscape sheet as in Figure 45.

	A	B	C
1	DSSTOX_LINK	PREFERRED_NAME	CHEMICAL/ENTITY_QUERY
2	DTXSID2031083	Cylindrospermopsin	143545-90-8 OR Cylindrospermopsin
3	DTXSID3031654	Microcystin LR	101043-37-2 OR Microcystin LR OR cyanoginosin LR
4	DTXSID3031656	Microcystin LA	96180-79-9 OR Microcystin LA OR cyanoginosin-LA
5	DTXSID9040974	Azaspiracid	214899-21-5 OR Azaspiracid
6	DTXSID3074313	Saxitoxin	35523-89-8 OR Saxitoxin
7	DTXSID60166611	beta-N-Methylamino-L-alanine	15920-93-1 OR beta-N-Methylamino-L-alanine
8	DTXSID70207660	Decarbamylsaxitoxin	58911-04-9 OR Decarbamylsaxitoxin
9	DTXSID60214520	Gonyautoxin V	64296-25-9 OR Gonyautoxin V
10	DTXSID20274180	L-Domoic acid	14277-97-5 OR L-Domoic acid OR domoic acid
11	DTXSID90423027	palytoxin	77734-91-9 OR palytoxin
12	DTXSID50867064	Anatoxin a	64285-06-9 OR Anatoxin a OR anatoxin I
13	DTXSID60879996	Brevetoxin A	98112-41-5 OR Brevetoxin A
14	DTXSID20879997	Brevetoxin 2	79580-28-2 OR Brevetoxin 2
15	DTXSID40879999	Brevetoxin C	98225-48-0 OR Brevetoxin C
16	DTXSID40880000	Ciguatoxin 1	11050-21-8 OR Ciguatoxin 1
17	DTXSID00880001	Dinophysistoxin 1	81720-10-7 OR Dinophysistoxin 1
18	DTXSID60880002	Okadaic acid	78111-17-8 OR Okadaic acid
19	DTXSID10880012	Maitotoxin	59392-53-9 OR Maitotoxin
20	DTXSID90880015	Lyngbyatoxin-a	70497-14-2 OR Lyngbyatoxin-a
21	DTXSID10880017	Euglenophycin	1219817-69-2 OR Euglenophycin
22	DTXSID60880022	Nodularin	118399-22-7 OR Nodularin
23	DTXSID20880023	Yessotoxin	112514-54-2 OR Yessotoxin
24	DTXSID80880024	Azaspiracid 4	344422-49-7 OR Azaspiracid 4
25	DTXSID60880082	Aplysiatoxin	52659-57-1 OR Aplysiatoxin

Worksheet1 | Abstract Sifter

Figure 43. Excel view of downloaded chemicals on Abstract Sifter sheet.

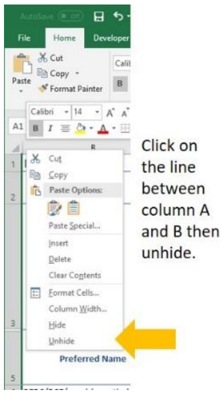

Click on the line between column A and B then unhide.

Figure 44. Unhide column A on the Landscape sheet in order to paste the DSSTox number there.

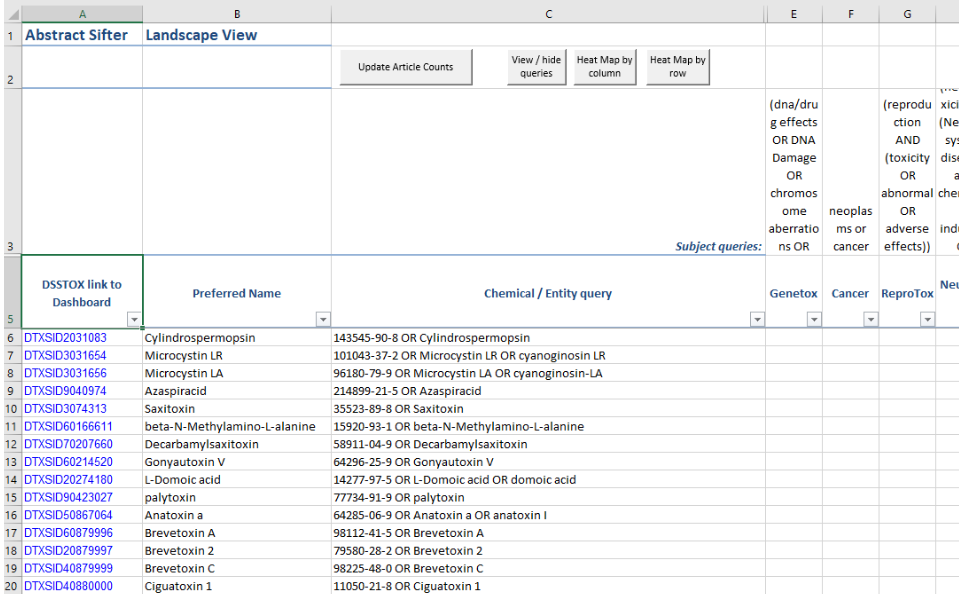

	A	B	C	E	F	G
1	Abstract Sifter	Landscape View				
2			Update Article Counts / View / hide queries / Heat Map by column / Heat Map by row			
3			Subject queries:	(dna/drug effects OR DNA Damage OR chromosome aberrations OR	neoplasms or cancer	(reproduction AND (toxicity OR abnormal OR adverse effects))
5	DSSTOX link to Dashboard	Preferred Name	Chemical / Entity query	Genetox	Cancer	ReproTox
6	DTXSID2031083	Cylindrospermopsin	143545-90-8 OR Cylindrospermopsin			
7	DTXSID3031654	Microcystin LR	101043-37-2 OR Microcystin LR OR cyanoginosin LR			
8	DTXSID3031656	Microcystin LA	96180-79-9 OR Microcystin LA OR cyanoginosin-LA			
9	DTXSID9040974	Azaspiracid	214899-21-5 OR Azaspiracid			
10	DTXSID3074313	Saxitoxin	35523-89-8 OR Saxitoxin			
11	DTXSID60166611	beta-N-Methylamino-L-alanine	15920-93-1 OR beta-N-Methylamino-L-alanine			
12	DTXSID70207660	Decarbamylsaxitoxin	58911-04-9 OR Decarbamylsaxitoxin			
13	DTXSID60214520	Gonyautoxin V	64296-25-9 OR Gonyautoxin V			
14	DTXSID20274180	L-Domoic acid	14277-97-5 OR L-Domoic acid OR domoic acid			
15	DTXSID90423027	palytoxin	77734-91-9 OR palytoxin			
16	DTXSID50867064	Anatoxin a	64285-06-9 OR Anatoxin a OR anatoxin I			
17	DTXSID60879996	Brevetoxin A	98112-41-5 OR Brevetoxin A			
18	DTXSID20879997	Brevetoxin 2	79580-28-2 OR Brevetoxin 2			
19	DTXSID40879999	Brevetoxin C	98225-48-0 OR Brevetoxin C			
20	DTXSID40880000	Ciguatoxin 1	11050-21-8 OR Ciguatoxin 1			

Figure 45. This is what the sheet will look like after unhiding Column A and pasting the chemicals downloaded from the Dashboard.

Now, enter subject matter queries, or, if you already have queries in place, select the intersecting cells and click on *Update Article Counts*. Click on one of the Heat Map buttons to make it pretty.

	A	B	C	E	F	G
1	Abstract Sifter	Landscape View				
2			Update Article Counts · View / hide queries · Heat Map by column · Heat Map by row			
3			*Subject queries:*	(dna/drug effects OR DNA Damage OR chromosome aberrations OR	neoplasms or cancer	(reproduction AND (toxicity OR abnormal OR adverse effects))
5	DSSTOX link to Dashboard	Preferred Name	Chemical / Entity query	Genetox	Cancer	ReproTox
6	DTXSID2031083	Cylindrospermopsin	143545-90-8 OR Cylindrospermopsin	48	15	8
7	DTXSID3031654	Microcystin LR	101043-37-2 OR Microcystin LR OR cyanoginosin LR	89	129	74
8	DTXSID3031656	Microcystin LA	96180-79-9 OR Microcystin LA OR cyanoginosin-LA	1	1	0
9	DTXSID9040974	Azaspiracid	214899-21-5 OR Azaspiracid	2	12	1
10	DTXSID3074313	Saxitoxin	35523-89-8 OR Saxitoxin	20	51	5
11	DTXSID60166611	beta-N-Methylamino-L-alanine	15920-93-1 OR beta-N-Methylamino-L-alanine	13	13	10
12	DTXSID70207660	Decarbamylsaxitoxin	58911-04-9 OR Decarbamylsaxitoxin	0	0	0
13	DTXSID60214520	Gonyautoxin V	64296-25-9 OR Gonyautoxin V	0	0	0
14	DTXSID20274180	L-Domoic acid	14277-97-5 OR L-Domoic acid OR domoic acid	78154	279707	12022
15	DTXSID90423027	palytoxin	77734-91-9 OR palytoxin	6	30	2
16	DTXSID50867064	Anatoxin a	64285-06-9 OR Anatoxin a OR anatoxin I	6	4	6
17	DTXSID60879996	Brevetoxin A	98112-41-5 OR Brevetoxin A	1	2	0
18	DTXSID20879997	Brevetoxin 2	79580-28-2 OR Brevetoxin 2	4	3	1
19	DTXSID40879999	Brevetoxin C	98225-48-0 OR Brevetoxin C	6	14	4
20	DTXSID40880000	Ciguatoxin 1	11050-21-8 OR Ciguatoxin 1	2	30	2

Figure 46. Downloaded chemicals and queries with subject matter queries.

Now you have an overview of your chemicals and what literature is out in PubMed for them. Take advantage of the iterative nature of the Abstract Sifter to query, sift, read, note as much as you need.

Contact:

Contact Nancy Baker at baker.nancy@epa.gov with issues, ideas, and feedback.

Read Me

Note: because of changes expected to PubMed web services, it is above) of the Abstract Sifter. In the future, you can find new versi https://gaftp.epa.gov/COMPTOX/Sustainable_Chemistry_Data/Cl actSifter.zip

Abstract Sifter v6

This is a special version of the Abstract Sifter populated with chemicals from the manuscript: Develo Flame Retardant and an Organohalogen Flame Retardant Chemical Inventory

The Abstract Sifter user guide has detailed instruction on how to use the tool for investigating literat of chemicals.

This special Abstract Sifter has two sheets with results from the manuscript. See FRUniverse and fromBergmanChems for chemicals lists to query.

Important notes about customizing and cleanup:

1. On the Main, Abstract, Notes, or Log sheets - do NOT delete or add columns.
2. On the Landscape sheet, delete and add query columns after column D. Feel free to delete or add rows
3. Feel free to delete your notes on the Notes sheet. Select rows and click on the delete button above.
4. Delete log entries that you no longer need.
5. On the Pathway_queries sheet and the Sample_queries sheet, feel free to delete rows after 3.

For best results, always delete rows and columns by selecting the row selector and column selector (the nur

Note: this file may come with some sample data. Feel free to delete it according to the cleanup ir

Recent enhancements:

Filters and Notes work.

You can now select many rows and apply the same Note to all of them.

Notes now include tags and yes/no/maybe.

Blank sifter terms won't cause error.

Double-clicking on an article in Notes will bring user to the abstract page.

09/07/2017 - Landscape page col A value changed.
09/07/2017 - Can select notes to send to PubMed (instead of all).
09/11/2017 - Fixed formatting on Landscape
10/20/2017 - Some cleanup; changed yes/no/maybe on Notes to string; No author to blanks.
10/23/2017 - reformatting
11/2/2017 - toggle for viewing queries on Landscape
11/12/2017 - Review column formatting fix; loop structure on heatmap; form renames
11/14/2017 - Error control on Landscape sheet and entity query expansion
04/24/2018 - Added wrap text to query on row 3, Landscape sheet
04/24/2018 - Row counts on Main changed to avoid 0 in column headers
04/24/2018 - Now records query counts for not-run queries (0 recs and >5000) on Log sheet
05/02/2018 - added occupational exposure to sample queries
05/30/2018 check on blank sifter terms added
09/20/2018 Lots of changes to the export function and AOP queries
9/26/2018 On Abstract sheet, added button and fuctionality for finding like articles or finding articles citing th
09/27/2018 Send queries to landscape simplified.
10/17/2018 Error control on PubMed retrieval (0 out of headers from bad PubMed … like this over 300?)
10/19/2018 Like this limited to 200 pmids in order to bypass error
10/30/2018 Added superheading to Pathway_queries and Sample_queries sheets; colorization transfers to La
11/05/2018 modified formatting on Sample_queries to Landscape;
11/06/2018 query run updated only if query is run to completion (2 changes); autofilter corrections on Lands
11/09/2018 set title and abstract to null before iteration
11/19/2018 added a timer to slow down Landscape retrieval to come under NLM limits
11/21/2018 note on ReadMe pointing to ftp site
12/19/2018 Landscape page fix - select area for update counts button checked

Version 3

01/29/2019 Colorize PMIDs immediately when taking notes / 2
02/01/2019 PubMed DOM rewrite. Added keywords and PMC flag.
02/05/ 2019 works fast - can't add perc notes … like not fixed
02/06/2019 trim abstract AND clean up formatting on Notes sheet
02/08/2019 Fixed log entries for like/citing
02/08/2019 Added nowrap on authors (example 25905296) and citation to ReadMe
02/12/2019 Formatting on pathway queries; font size on query boxes reduced.
02/21/2019 Fixed problem with pubyr like Spring 2019 and unwrap Journal title: 24943998[uid] OR 2813912[illegible]
02/21/2019 Started reworking pathway_queries to have KE by number when possible
03/06/2019 Pathway queries send col A and B to landscape, not C.
07/08/2019 Landscape sheet - no error msg if blank chem/subject cells selected
07/08/2019 Pathway and Sample queries - send only selected AND visible rows to Landscape
08/19/2019 Fixed problem with copy to clipboard from Notes sheet
09/05/2019 Enhanced some sample_queries

Version 4

09/12/2019 Allows appending on Main sheet
09/19/2019 Buttons on Main sheet changed. More things button now has delete dups and highlight noted
09/19/2019 From Landscape appending allowed. Volume, issue, pages, doi, pubtype added to Main and Not[illegible]
10/11/2019 Working on RIS export
10/14/2019 Txt exporting
10/18/2019 Added initials to author names; modified RIS export and export form; some reformatting of Note[illegible]

11/01/2019 Added error control to Log sheet and Notes sheet
11/05/2019 Fixed changes from 11/01 that were wrong, including Notes from Abstract sheet. Took title term
11/05/2019 Added append option to Get Like
11/06/2019 Added scroll bar to curation form
11/07/2019 Fixed pmid hyperlink on Notes coming from Abstract; Landscape Col A - unhidden
11/08/2019 PMID colorization on notes from Abstract fixed
11/25/2019 Fixed pmid hyperlink on Notes when inserting from Abstract sheet
02/2020 More improvements to curation form
03/06/2020 Encoding ampersands in queries
03/13/2020   Fixed eutil/http problem giving error "The download of the specified resource has failed." Hype

Version 5

needs some fixes
04/23/2020 PMID link on Curate form fixed; working on pdf column and curation change for pdf link
06/03/2020 Fixes: export to text
06/12/2020 wait on pmid counts extended; encoding of ampersands and quotation marks added
07/10/2020 PubMed xml call error check and redo
07/10/2020 CuratedLists code - activesheet

Version 5.5

08/2020 Speed improvements
08/2020 Cancel button added
08/2020 Curate form - new copy feature and pdf attach
08/2020 Notes functionality improved; attach pdf
08/2020 Trim added to Landscape query entity only; tab on notes form modified
09/2020 Changes to source check on Log
09/28/2020 Row number consistency improved
10/01/2020 If no good, bad, maybe style, then added
10/08/2020 Styles eliminated. Old notes code removed.
10/19/2020 Error control if trying to open blank pdf

Version 5.6

12/04/2020 Adding batch run capability; PubMed module renamed
12/22/2020 Added colors to Likethis; update and next option to curation form
12/28/2020 Conflicting yes/no/maybe on Notes highlight option in More stuff form
12/28/2020 Notetaker field added to Notes form
01/27/2021 Running 0 record queries in batch … fixed

Version 5.7

03/31/2021 Curate form copy function output format modified
03/31/2021 If Notes with conflicting yes/no/maybe then colorize purple
04/09/2021 Next/previous buttons on Abstract sheet

Version 6.0

05/15/2021 TermMap added
05/18/2021 Messages on Main fixed
06/01/2021 Adjustments made to Term Mapping (lcase and TX appended on double-click)
06/2021 fixed RIS export of keywords
07/07/2021 Backed out MeSH extract; added copy to Main from Notes More Stuff
07/08/2021 Fixed colorization of mapped terms on Abstract sheet; column formatting changed for batch
07/20/2021 Fixed dialog box on Main
07/21/2021 Added msgbox to TermMap for continue decision

important to use a newer version (v3 or
ons here:
hemistry_Dashboard/Abstract_Sifter/Abstr

pment of a

ture for a set

The Abst
and run

Because

How to u
hyperlin

Abstract
https://y

Abstract
https://y

Abstract
https://y

ASCCT w
https://w

This tool
RIS code

AFTER row 5.

mbers or letters).

nstructions above.

tract Sifter is a tool that helps readers and researchers find PubMed articles quickly a
a PubMed query and 2) Sift and sort the results of that query and 3) make notes on a

the Abstract Sifter talks directly to PubMed, you must be connected to the internet

use the Abstract Sifter: the best way is to watch someone else use it. Here are links
ks don't work, copy and paste into your browser.)

Sifter tutorial part one: The basics
outu.be/B7-pMGpY_Us

Sifter tutorial part two: Taking notes
outu.be/mYxG8RhDgHE

Sifter tutorial part three: Landscape view
outu.be/s64jTt9U57I

ebinar recording from 2020
www.ascctox.org/webinar/69

was created by Nancy Baker, Leidos, contractor to US EPA. Email : baker.nancy@epa.gov
contributed by Tom Transue, GDIT, contractor to US EPA.

ınd easily. The tool has three major functions: 1) build
ırticles of interest.

to use it. Be sure to enable content / macros!

to the currently available video tutorials: (If the

Abstract Sifter

Version 6.0 (beta)

Your sifter terms and frequency counts

PMID	dechlor	water	organo	Score	Pub Yr
26313934	15	0	0	15	2015
28110879	14	2	5	21	2017
22695503	14	0	0	14	2012
26854703	13	0	0	13	2016
30159835	12	12	0	24	2018
26241843	12	2	0	14	2015
21351765	12	11	0	23	2011
20681512	12	0	0	12	2010
26572321	11	5	0	16	2015
24835686	11	0	0	11	2014
24440800	11	0	1	12	2014
23183230	11	0	0	11	2013
22913883	11	2	0	13	2012
31276863	10	0	0	10	2019
26476047	10	0	0	10	2016
26386769	10	0	0	10	2016
24997969	10	4	0	14	2014
21222481	10	0	0	10	2011
32801111	9	0	0	9	2020
27989386	9	0	0	9	2017
24702310	9	2	0	11	2014
21434636	9	4	0	13	2011
21133428	9	3	0	12	2011
28593372	8	0	0	8	2017
26766358	8	3	0	11	2016
26042896	8	4	0	12	2015
25184555	8	2	0	10	2014
24996336	8	2	0	10	2014
22985592	8	3	0	11	2013
22718042	8	0	0	8	2012
21956524	8	3	0	11	2011
33743437	7	2	1	10	2021
33466958	7	0	0	7	2021
29407832	7	2	0	9	2018
28672702	7	2	1	10	2017
22863566	7	4	0	11	2012

31129551	6	2	0	8	2019
30077101	6	0	0	6	2018
29659266	6	2	0	8	2018
29294456	6	1	0	7	2018
27654949	6	0	0	6	2016
26453819	6	0	0	6	2015
25662232	6	3	0	9	2015
25113208	6	0	0	6	2014
22899248	6	0	0	6	2012
21864960	6	0	0	6	2011
21615082	6	0	0	6	2011
33087285	5	2	1	8	2021
31627044	5	0	0	5	2020
31141740	5	0	0	5	2019
30347870	5	0	0	5	2018
29803891	5	2	0	7	2018
29078185	5	0	0	5	2018
28214714	5	0	0	5	2017
26760718	5	0	0	5	2016
25542638	5	0	0	5	2015
25463253	5	4	0	9	2015
25410309	5	2	1	8	2015
25079277	5	0	0	5	2014
24295901	5	2	0	7	2014
24290300	5	1	0	6	2014
23992842	5	0	0	5	2013
23747460	5	2	0	7	2013
23202648	5	0	0	5	2013
22727895	5	0	0	5	2012
22695692	5	2	0	7	2012
21647944	5	0	0	5	2011
21324409	5	0	0	5	2011
20728200	5	1	0	6	2010
20000816	5	2	0	7	2010
20000543	5	0	0	5	2009
34082369	4	0	0	4	2021
33660181	4	0	1	5	2021
33310198	4	9	0	13	2021
33068787	4	0	0	4	2020
32864714	4	0	0	4	2020
32380452	4	0	0	4	2020
32209501	4	3	0	7	2020
31865567	4	2	0	6	2020
31628060	4	0	0	4	2020
31154201	4	2	0	6	2019
30852890	4	0	0	4	2019
30708317	4	0	0	4	2019

30681328	4	0	0	4	2019
30384072	4	1	0	5	2019
30321706	4	0	0	4	2019
30316096	4	2	0	6	2019
30223335	4	4	0	8	2018
29727946	4	0	0	4	2018
29709809	4	0	0	4	2018
29459350	4	0	0	4	2018
29102191	4	2	0	6	2018
28954370	4	0	0	4	2018
28651087	4	2	0	6	2017
28551155	4	0	0	4	2017
28441609	4	2	0	6	2017
28397063	4	0	0	4	2018
28288352	4	1	0	5	2017
27572532	4	3	0	7	2016
27421724	4	2	0	6	2016
26735721	4	4	0	8	2016
26651433	4	0	0	4	2016
26619313	4	0	0	4	2016
26502059	4	0	1	5	2015
25950133	4	0	0	4	2015
25666277	4	0	0	4	2015
25585867	4	0	0	4	2015
24793840	4	12	0	16	2014
24764193	4	2	0	6	2014
24751489	4	0	0	4	2014
24737022	4	0	0	4	2015
24594740	4	2	0	6	2014
24568839	4	6	0	10	2014
24534698	4	2	0	6	2014
24509140	4	0	0	4	2014
24463492	4	2	0	6	2014
24433788	4	2	0	6	2014
24295775	4	1	0	5	2014
24261747	4	0	0	4	2014
24211159	4	0	0	4	2014
24120307	4	0	0	4	2013
22982220	4	0	0	4	2012
21751774	4	5	0	9	2011
21621844	4	0	0	4	2011
21376363	4	4	0	8	2011
21105700	4	0	0	4	2010
18754476	4	3	0	7	2008
18350871	4	0	0	4	2008
18284131	4	3	0	7	2008
33728497	3	0	0	3	2021

33170516	3	0	0	3	2021
32806402	3	0	0	3	2020
32805504	3	0	0	3	2020
32721756	3	0	0	3	2020
32556108	3	0	0	3	2020
32354043	3	0	0	3	2020
32252527	3	0	0	3	2020
31826665	3	0	0	3	2019
30595018	3	0	0	3	2019
29960221	3	0	0	3	2018
29717277	3	0	0	3	2019
29625318	3	0	0	3	2018
29236134	3	0	0	3	2018
29149662	3	0	2	5	2018
28511039	3	0	0	3	2017
28214009	3	2	0	5	2017
27814535	3	0	0	3	2016
27608428	3	0	0	3	2016
26884244	3	0	0	3	2016
26829245	3	0	0	3	2016
26452921	3	0	0	3	2015
26098022	3	0	2	5	2015
25463724	3	0	0	3	2015
25454220	3	0	0	3	2015
25149073	3	8	0	11	2015
24710727	3	0	0	3	2014
24021719	3	0	0	3	2013
23919519	3	0	0	3	2013
23801340	3	0	0	3	2013
23745424	3	0	0	3	2013
23542572	3	0	0	3	2013
23500052	3	2	0	5	2013
23440862	3	0	0	3	2013
23427074	3	3	0	6	2013
23416272	3	0	0	3	2013
23354373	3	0	0	3	2013
23246747	3	0	0	3	2013
23245762	3	0	0	3	2013
23016984	3	2	0	5	2012
22913625	3	0	0	3	2012
22698370	3	1	0	4	2012
22376139	3	2	0	5	2012
22060819	3	2	0	5	2011
22032166	3	0	0	3	2011
21705082	3	4	0	7	2011
21574656	3	0	0	3	2011
21561692	3	0	0	3	2011

21539933	3	2	0	5	2011
21529947	3	0	0	3	2011
21213363	3	1	0	4	2011
21128659	3	4	0	7	2011
21122888	3	4	0	7	2011
21047104	3	5	0	8	2010
20959615	3	0	0	3	2010
20673687	3	5	0	8	2011
20630636	3	0	0	3	2010
20621327	3	0	0	3	2010
20303139	3	2	0	5	2010
20210356	3	11	0	14	2010
20025285	3	4	0	7	2010
19994895	3	5	0	8	2010
19108864	3	0	0	3	2009
18800517	3	0	0	3	2008
18075076	3	2	0	5	2007
16572773	3	2	0	5	2006
34134417	2	6	0	8	2021
34023657	2	0	0	2	2021
32750573	2	0	2	4	2020
32146393	2	0	4	6	2020
31926415	2	0	2	4	2020
31891828	2	0	0	2	2020
31864027	2	0	0	2	2020
31260929	2	1	0	3	2019
31228838	2	2	0	4	2019
30995592	2	0	0	2	2019
30844700	2	1	4	7	2019
30830665	2	0	0	2	2019
30390529	2	0	4	6	2019
30086519	2	0	0	2	2018
30029314	2	7	0	9	2018
29908512	2	0	0	2	2018
29879556	2	0	0	2	2018
29751406	2	0	0	2	2018
29461545	2	1	0	3	2018
29289283	2	4	0	6	2017
29235599	2	2	0	4	2018
29216545	2	0	0	2	2018
29192494	2	0	0	2	2018
29107904	2	0	0	2	2018
28696837	2	0	0	2	2017
28550783	2	4	1	7	2017
28545217	2	0	0	2	2017
28419962	2	2	0	4	2017
28292528	2	2	0	4	2017

28254720	2	0	0	2	2017
28144875	2	0	3	5	2017
27993042	2	7	0	9	2016
27900722	2	0	0	2	2017
27788366	2	0	0	2	2017
27728888	2	0	1	3	2017
27639615	2	2	0	4	2017
27453143	2	2	2	6	2016
27346442	2	0	3	5	2016
27149147	2	0	0	2	2016
26780041	2	0	0	2	2016
26724461	2	0	1	3	2016
26363723	2	0	0	2	2016
26209127	2	2	0	4	2015
26111846	2	0	0	2	2015
25795070	2	0	0	2	2015
25380095	2	0	0	2	2014
25303654	2	2	3	7	2015
25286358	2	7	0	9	2014
25010345	2	0	2	4	2014
24206837	2	0	0	2	2014
23000968	2	3	1	6	2013
22845168	2	0	0	2	2012
22208742	2	0	0	2	2012
21742626	2	0	0	2	2011
18284135	2	0	0	2	2008
17937275	2	3	0	5	2007
17438771	2	4	0	6	2007
33754718	1	0	1	2	2021
33540273	1	0	0	1	2021
33352370	1	0	0	1	2021
33321435	1	0	2	3	2021
33068585	1	0	0	1	2021
33010546	1	0	5	6	2021
32758856	1	0	1	2	2020
32305741	1	0	2	3	2020
32251911	1	0	0	1	2020
32220776	1	0	0	1	2020
32163809	1	0	0	1	2020
32112953	1	0	1	2	2020
32041014	1	0	0	1	2020
32006868	1	0	4	5	2020
31869616	1	0	2	3	2020
31820947	1	0	4	5	2020
31600649	1	0	0	1	2019
31520824	1	0	1	2	2019
31349194	1	1	0	2	2019

30901092	1	2	0	3	2019
30761900	1	0	1	2	2019
30677996	1	1	0	2	2019
30665116	1	2	0	3	2019
30567666	1	0	1	2	2019
30524917	1	1	0	2	2018
30502730	1	0	0	1	2019
30366322	1	8	4	13	2019
30308922	1	0	0	1	2019
30235595	1	0	0	1	2019
30122736	1	0	0	1	2018
30089276	1	0	1	2	2019
30005248	1	4	2	7	2018
29990753	1	2	0	3	2018
29316461	1	0	1	2	2018
29134799	1	0	0	1	2017
29107777	1	4	3	8	2018
29054623	1	5	0	6	2018
28724249	1	0	0	1	2017
28609849	1	0	0	1	2017
28593369	1	0	2	3	2017
28448930	1	3	0	4	2017
28406624	1	0	0	1	2017
28384585	1	0	0	1	2017
28258858	1	0	0	1	2017
27741446	1	8	1	10	2017
27539248	1	0	1	2	2016
27513551	1	0	0	1	2016
27387797	1	0	3	4	2016
29964460	1	4	0	5	2016
27239689	1	0	3	4	2016
27177141	1	12	1	14	2016
27160856	1	0	0	1	2016
27088732	1	2	0	3	2016
26843139	1	0	0	1	2016
26821261	1	2	0	3	2016
26807939	1	2	0	3	2016
26552538	1	1	0	2	2016
26520266	1	0	8	9	2016
26406977	1	0	1	2	2015
26318119	1	2	0	3	2015
26078122	1	2	0	3	2015
26050713	1	0	3	4	2015
25974192	1	1	0	2	2015
25863512	1	9	1	11	2015
25769908	1	3	0	4	2015
25728907	1	0	4	5	2015

25677852	1	0	2	3	2015
25661400	1	1	0	2	2015
25629888	1	0	0	1	2015
25306095	1	0	0	1	2015
25096494	1	3	0	4	2015
25084546	1	0	0	1	2014
24997943	1	0	0	1	2014
24992563	1	0	0	1	2014
24927135	1	0	0	1	2014
24888473	1	1	0	2	2014
24859045	1	2	1	4	2014
24848787	1	0	4	5	2014
24845805	1	2	2	5	2014
24657373	1	0	1	2	2014
24583391	1	0	3	4	2014
24530800	1	2	0	3	2014
24400732	1	2	0	3	2014
24385192	1	0	1	2	2014
24059974	1	0	0	1	2013
23830888	1	0	3	4	2013
23809805	1	4	0	5	2013
23473389	1	0	0	1	2013
23408421	1	0	0	1	2013
23262072	1	0	0	1	2013
23231623	1	0	0	1	2013
23185960	1	0	3	4	2012
22930373	1	0	0	1	2012
22766500	1	0	0	1	2012
22609680	1	0	0	1	2012
22579795	1	1	0	2	2012
22280921	1	3	0	4	2012
22279897	1	5	0	6	2011
22230080	1	0	0	1	2012
21542623	1	0	0	1	2011
21500827	1	0	0	1	2011
20821514	1	2	0	3	2010
20704217	1	0	1	2	2010
20594579	1	7	0	8	2010
20579684	1	0	2	3	2010
20486701	1	1	2	4	2010
20025286	1	0	0	1	2010
19161661	1	2	0	3	2009
19049258	1	3	0	4	2009
18678000	1	0	0	1	2008
18350866	1	0	0	1	2008
17695897	1	1	0	2	2007

PubMed query run: (Dechlorane Plus)

Title

Ultra-trace measurement of Dechloranes to investigate food as a route of human exposure.
Bioaccumulation of Dechloranes, organophosphate esters, and other flame retardants in Great Lakes fish.
Dechlorane Plus and related compounds in aquatic and terrestrial biota: a review.
Occurrence of Dechlorane compounds and polybrominated diphenyl ethers (PBDEs) in the Korean general popula
Distributions, influencing factors, and risk assessment of Dechlorane Plus and related compounds in surficial wate
Determination of dechlorane flame retardants in soil and fish at Guiyu, an electronic waste recycling site in south
Concentration and bioaccumulation of dechlorane compounds in coastal environment of northern China.
An Asia-specific source of dechlorane plus: concentration, isomer profiles, and other related compounds.
Evidence for Anaerobic Dechlorination of Dechlorane Plus in Sewage Sludge.
Assessment of Dechlorane compounds in foodstuffs obtained from retail markets and estimates of dietary intake
Levels of dechloranes and polybrominated diphenyl ethers (PBDEs) in human serum from France.
Levels and distribution of Dechlorane Plus and related compounds in surficial sediments of the Qiantang River in e
Tissue distribution, maternal transfer, and age-related accumulation of dechloranes in Chinese sturgeon.
Assessment of Dechlorane Plus and related compounds in foodstuffs and estimates of daily intake from Lebanese
Determination of Dechlorane Plus and related compounds (dechlorane 602, 603 and 604) in fish and vegetable oil
Dechloranes in lichens from the southeast Tibetan Plateau: Evidence of long-range atmospheric transport.
Kinetics of stereoselective enrichment of Dechlorane Plus in Ulva Pertusa.
Dechlorane plus and related compounds in peregrine falcon (Falco peregrinus) eggs from Canada and Spain.
Time trend of exposure to dechloranes: Plasma samples of German young adults from the environmental specime
Halogenated flame retardants in bobcats from the midwestern United States.
Trophic transfer of dechloranes in the marine food web of Liaodong Bay, north China.
Historic trends of dechloranes 602, 603, 604, dechlorane plus and other norbornene derivatives and their bioaccu
Dechloranes 602, 603, 604, Dechlorane Plus, and Chlordene Plus, a newly detected analogue, in tributary sedimer
Simultaneous determination of dechloranes, polybrominated diphenyl ethers and novel brominated flame retarda
The occurrence of Dechlorane Plus and related norbornene-based flame retardants in Baltic wild salmon (Salmo s
Maternal transfer of emerging brominated and chlorinated flame retardants in European eels.
Identification and occurrence of analogues of dechlorane 604 in Lake Ontario sediment and their accumulation in
Optimisation of matrix solid-phase dispersion for the determination of Dechlorane compounds in marketed fish.
Brominated flame retardants and dechloranes in eels from German Rivers.
Identification and determination of the dechlorination products of Dechlorane 602 in Great Lakes fish and Arctic b
Analysis and occurrence of emerging chlorinated and brominated flame retardants in surficial sediment of the Dal
Occurrence of Dechlorane series flame retardants in sediments from the Pearl River Delta, South China.
Dechlorane Plus and Related Compounds in Food-A Review.
Emerging brominated flame retardants and dechlorane-related compounds in European eels (Anguilla anguilla) fr
Spatial distribution and implications to sources of halogenated flame retardants in riverine sediments of Taizhou,
Dechloranes in a river in northeastern China: spatial trends in multi-matrices and bioaccumulation in fish (Enchely

Occurrence of legacy and emerging organic pollutants in whitemouth croakers from Southeastern Brazil.
Dechlorane Plus flame retardant in a contaminated frog species: Biomagnification and isomer-specific transfer fro
Dechlorinated Analogues of Dechlorane Plus.
Occurrence and distribution of old and new halogenated flame retardants in mosses and lichens from the South S
Measurement of emerging dechloranes in human serum using modulated gas chromatography coupled to electro
Persistent and emerging pollutants in the blood of German adults: Occurrence of dechloranes, polychlorinated na
Distribution characteristics and indicator significance of Dechloranes in multi-matrices at Ny-Ålesund in the Arctic.
Sources, gastrointestinal absorption and stereo-selective and tissue-specific accumulation of Dechlorane Plus (DP
Dechlorane Plus in eggs of two gull species (Larus michahellis and Larus audouinii) from the southwestern Medite
Tissue distribution of Dechlorane Plus and its dechlorinated analogs in contaminated fish: high affinity to the brair
Historically and currently used Dechloranes in the sediments of the Great Lakes.
Occurrence, distribution, and bioaccumulation of new and legacy persistent organic pollutants in an ecosystem or
Bioaccumulation and transfer characteristics of dechlorane plus in human adipose tissue and blood stream and th
Correlations between dechlorane plus concentrations in paired hair and indoor dust samples and differences betv
Distribution Characteristics and Source of Dechloranes in Soil and Lichen of the Fildes Peninsula (Antarctica).
Occurrence of Dechlorane Plus and related compounds in catfish (Silurus spp.) from rivers in France.
Development and optimization of gas chromatography coupled to high resolution mass spectrometry based meth
Current halogenated flame retardant concentrations in serum from residents of Shandong Province, China, and te
Sources and environmental behaviors of Dechlorane Plus and related compounds - A review.
Dechlorane Plus in paired hair and serum samples from e-waste workers: correlation and differences.
Trophic magnification of chlorinated flame retardants and their dechlorinated analogs in a fresh water food web.
Levels and distribution of dechloranes in sediments of Lake Taihu, China.
Bioavailability and tissue distribution of Dechloranes in wild frogs (Rana limnocharis) from an e-waste recycling ar
Occurrence of classic and emerging halogenated flame retardants in sediment and sludge from Ebro and Llobrega
Brominated flame retardants and Dechloranes in European and American eels from glass to silver life stages.
Determination of emerging halogenated flame retardants and polybrominated diphenyl ethers in serum by gas ch
Photodegradation of Dechlorane Plus in n-nonane under the irradiation of xenon lamp.
Dechlorane Plus and its dechlorinated analogs from an e-waste recycling center in maternal serum and breast mil
Species- and tissue-specific accumulation of Dechlorane Plus in three terrestrial passerine bird species from the P
Analytical method for the determination of halogenated norbornene flame retardants in environmental and biota
Dechlorane Plus in house dust from E-waste recycling and urban areas in South China: sources, degradation, and h
Dechlorane Plus (DP) in air and plants at an electronic waste (e-waste) site in South China.
Mechanochemical destruction of Dechlorane Plus with calcium oxide.
Identification and screening analysis of halogenated norbornene flame retardants in the Laurentian Great Lakes: D
Determination of Dechlorane Plus in serum from electronics dismantling workers in South China.
A review on the analytical procedures of halogenated flame retardants by gas chromatography coupled with singl
Mechanisms of emerging pollutant Dechlorane Plus on the production of short-chain fatty acids from sludge anae
Source, fate and budget of Dechlorane Plus (DP) in a typical semi-closed sea, China.
Polybrominated diphenyl ethers, decabromodiphenyl ethane and dechlorane plus in aquatic products from the Ye
Dechlorane Plus as an emerging environmental pollutant in Asia: a review.
Intergenerational transfer of Dechlorane Plus and the associated long-term effects on the structure and function o
Toxic effects of dechlorane plus on the common carp (Cyprinus carpio) embryonic development.
Uptake, depuration, bioaccumulation, and selective enrichment of dechlorane plus in common carp (Cyprinus car
Halogenated flame retardants in sediments from the Upper Laurentian Great Lakes: Implications to long-range tra
Neurodevelopmental toxicity assessments of alkyl phenanthrene and Dechlorane Plus co-exposure in zebrafish.
Novel Dechlorane Analogues and Possible Sources in Peregrine Falcon Eggs and Shark Livers from the Western No
Comparative study of dechlorane plus (DP) in adult chickens and developing embryos: Stereo-selective bioaccumu

Spatial and Temporal Trends (2004-2016) of Selected Alternative Flame Retardants in Fish of the Laurentian Great
Dechlorane plus in greenhouse and conventional vegetables: Uptake, translocation, dissipation and human dietar
Is the urban-adapted ring-billed gull a biovector for flame retardants?
Bioaccumulation and cycling of polybrominated diphenyl ethers (PBDEs) and dechlorane plus (DP) in three natura
New brominated flame retardants and dechlorane plus in the Arctic: Local sources and bioaccumulation potential
Levels, occurrence and human exposure to novel brominated flame retardants (NBFRs) and Dechlorane Plus (DP)
Enrichment and physiological responses of dechlorane plus on juvenile marine macroalgae (Ulva pertusa).
PBDEs and Dechlorane Plus in the environment of Guiyu, Southeast China: A historical location for E-waste recycli
Stereoselective bioaccumulation of syn- and anti-Dechlorane plus isomers in different tissues of common carp (Cy
Brominated flame retardant (BFRs) and Dechlorane Plus (DP) in paired human serum and segmented hair.
Effects of terrestrial and marine organic matters on deposition of dechlorane plus (DP) in marine sediments from
Coupling of supercritical fluid chromatography to mass spectrometry for the analysis of Dechlorane Plus: Examina
Dechlorane Plus induces oxidative stress and decreases cyclooxygenase activity in the blue mussel.
Brominated flame retardants and dechlorane plus on a remote high mountain of the eastern Tibetan Plateau: imp
Effects of Dechlorane Plus exposure on axonal growth, musculature and motor behavior in embryo-larval zebrafis
Distribution and region-specific sources of Dechlorane Plus in marine sediments from the coastal East China Sea.
Spatial and Vertical Distribution of Dechlorane Plus in Mangrove Sediments of the Pearl River Estuary, South China
Toxicological responses following short-term exposure through gavage feeding or water-borne exposure to Dechl
Maternal transfer of dechloranes and their distribution among tissues in contaminated ducks.
Ecotoxicological effects of earthworm following long-term Dechlorane Plus exposure.
Occurrence of Legacy and New Persistent Organic Pollutants in Avian Tissues from King George Island, Antarctica.
Occurrence, behavior and human health risk assessment of dechlorane plus and related compounds in indoor dus
Accumulation of Dechlorane Plus flame retardant in terrestrial passerines from a nature reserve in South China: th
Potential genotoxicity and risk assessment of a chlorinated flame retardant, Dechlorane Plus.
Polybrominated diphenyl ethers (PBDEs) and dechlorane plus (DP) in a conventional wastewater treatment plant
Polybrominated diphenyl ethers, dechlorane plus, and polychlorinated biphenyls in tree bark near the upper Yello
Short-term effects of Dechlorane Plus on the earthworm Eisenia fetida determined by a systems biology approach
Dechlorane Plus and decabromodiphenyl ether in atmospheric particles of northeast Asian cities.
Investigating Dechlorane Plus (DP) distribution and isomer specific adsorption behavior in size fractionated marine
Isomers of Dechlorane Plus in an aquatic environment in a highly industrialized area in Southern China: spatial and
Novel brominated flame retardants and dechloranes in three fish species from the St. Lawrence River, Canada.
Hexachloronorbornene-based flame retardants in humans: levels in maternal serum and milk.
Effects of dechlorane plus on the hepatic proteome of juvenile Chinese sturgeon (Acipenser sinensis).
Gastrointestinal absorption, dynamic tissue-specific accumulation, and isomer composition of dechlorane plus and
Effects of zero-valent metals together with quartz sand on the mechanochemical destruction of dechlorane plus c
Placental transfer of dechlorane plus in mother-infant pairs in an e-waste recycling area (Wenling, China).
Inhalation and dietary exposure to Dechlorane Plus and polybrominated diphenyl ethers in Osaka, Japan.
Levels, profile and distribution of Dechloran Plus (DP) and Polybrominated Diphenyl Ethers (PBDEs) in the environ
Dechlorane Plus in serum from e-waste recycling workers: influence of gender and potential isomer-specific meta
Polybrominated diphenyl ethers vs alternate brominated flame retardants and Dechloranes from East Asia to the
Sources and environmental behavior of dechlorane plus--a review.
Levels and distribution of Dechlorane Plus in coastal sediments of the Yellow Sea, North China.
Dechlorane Plus in human hair from an e-waste recycling area in South China: comparison with dust.
Examination of isomer specific bioaccumulation parameters and potential in vivo hepatic metabolites of syn- and
Dechlorane plus and other flame retardants in tree bark from the northeastern United States.
Dechlorane plus levels in sediment of the lower Great Lakes.
Analysis of Dechlorane Plus and related compounds in gull eggs by GC-HRMS using a novel atmospheric pressure [illegible]

Dechlorane Plus Biomagnification and Transmission through Prairie Food Webs in Inner Mongolia, China.
Transfer of dechlorane plus between human breast milk and adipose tissue and comparison with legacy lipophilic
Effects of dechlorane plus on intestinal barrier function and intestinal microbiota of Cyprinus carpio L.
Global emissions of Dechlorane Plus.
Exposure to Low Doses of Dechlorane Plus Promotes Adipose Tissue Dysfunction and Glucose Intolerance in Male
Levels of Octachlorostyrene in Mothers' Milk and Potential Exposure Among Infants in Sendai City, Japan 2012.
Fractions Transformation and Dissipation Mechanism of Dechlorane Plus in the Rhizosphere of the Soil-Plant Syste
Effects of dechlorane plus on oxidative stress, inflammatory response, and cell apoptosis in Cyprinus carpio.
Validation and Application of a 3-Step Sequential Extraction Method to Investigate the Fraction Transformation of
Integrating population connectivity into pollution assessment: Overwintering mixing reveals flame retardant cont
Dechlorane Plus increases adipogenesis in 3T3-L1 and human primary preadipocytes independent of peroxisome
Halogenated flame retardants in building and decoration materials in China: Implications for human exposure via
Determination of halogenated flame retardants by GC-API-MS/MS and GC-EI-MS: a multi-compound multi-matrix
In ovo transformation of two emerging flame retardants in Japanese quail (Coturnix japonica).
Occurrence, composition, source, and regional distribution of halogenated flame retardants and polybrominated
Trophic magnification of Dechlorane Plus in the marine food webs of Fildes Peninsula in Antarctica.
Species-specific accumulation of polybrominated diphenyl ethers (PBDEs) and other emerging flame retardants in
Measurement and health risk assessment of PM2.5, flame retardants, carbonyls and black carbon in indoor and o
Airborne polybrominated diphenyl ethers (PBDEs), polybrominated dibenzo-p-dioxins/furans (PBDD/Fs), and dech
Evaluation of the Genotoxic and Physiological Effects of Decabromodiphenyl Ether (BDE-209) and Dechlorane Plus
Simultaneous determination of three alternative flame retardants (dechlorane plus, 1,2-bis(2,4,6-tribromophenox
Multiyear Measurements of Flame Retardants and Organochlorine Pesticides in Air in Canada's Western Sub-Arcti
Novel brominated flame retardants and dechlorane plus in Greenland air and biota.
Comparing human exposure to emerging and legacy flame retardants from the indoor environment and diet with
Distribution, congener profile, and risk of polybrominated diphenyl ethers and dechlorane plus in water and sedin
Dechlorane Plus in surface soil of North China: levels, isomer profiles, and spatial distribution.
Alternative flame retardants, Dechlorane Plus and BDEs in the blubber of harbour porpoises (Phocoena phocoena
Assessment on the occupational exposure of manufacturing workers to Dechlorane Plus through blood and hair a
Levels and profiles of Dechlorane Plus in a major E-waste dismantling area in China.
[Levels and sources of decabromodiphenyl ether and dechlorane plus in Xining and Tianjun, Qinghai Province, Chi
In vitro biotransformation of decabromodiphenyl ether (BDE-209) and Dechlorane Plus flame retardants: a case st
Sex-dependent accumulation and maternal transfer of Dechlorane Plus flame retardant in fish from an electronic
Accumulation and effects of 90-day oral exposure to Dechlorane Plus in quail (Coturnix coturnix).
Dechlorane plus monoadducts in a Lake Ontario (Canada) food web and biotransformation by lake trout (Salvelinu
Dechlorane Plus flame retardant in terrestrial raptors from northern China.
Human health risk assessment of occupational and residential exposures to dechlorane plus in the manufacturing
Dechlorane Plus flame retardant in kingfishers (Alcedo atthis) from an electronic waste recycling site and a refere
Accumulation pattern of Dechlorane Plus and associated biological effects on rats after 90 d of exposure.
Dechlorane-related compounds in franciscana dolphin (Pontoporia blainvillei) from southeastern and southern co
Responses of mouse liver to dechlorane plus exposure by integrative transcriptomic and metabonomic studies.
Detection of Dechlorane Plus and brominated flame retardants in marketed fish in Japan.
Brominated flame retardants and dechlorane plus in the marine atmosphere from Southeast Asia toward Antarcti
Dechlorane plus in the atmosphere and precipitation near the Great Lakes.
[Determination of polybrominated diphenyl ethers and dechlorane plus in fish and fish oil supplements by gel per
Biota-sediment accumulation factors for Dechlorane Plus in bottom fish from an electronic waste recycling site, S
Dechlorane plus and related compounds in the environment: a review.
Particle-bound Dechlorane Plus and polybrominated diphenyl ethers in ambient air around Shanghai, China.

The effects of Dechlorane Plus on toxicity and mRNA expression in chicken embryos: a comparison of in vitro and
Dechlorane plus and possible degradation products in white stork eggs from Spain.
Liquid chromatography/atmospheric pressure photoionization tandem mass spectrometry for analysis of Dechlor
Bioaccumulation of several brominated flame retardants and dechlorane plus in waterbirds from an e-waste recyc
Concentrations and sources of Dechlorane Plus in sewage sludge.
Large-scale distribution of dechlorane plus in air and seawater from the Arctic to Antarctica.
Oral repeat dose and reproductive toxicity of the chlorinated flame retardant Dechlorane Plus.
Dechlorane plus in multimedia in northeastern Chinese urban region.
Levels and isomer profiles of Dechlorane Plus in the surface soils from e-waste recycling areas and industrial areas
Dechlorane Plus pollution and inventory in soil of Huai'an City, China.
Detection of Dechlorane Plus in fish from urban-industrial rivers.
Dechlorane Plus in surficial water and sediment in a northeastern Chinese river.
Compounds structurally related to Dechlorane Plus in sediment and biota from Lake Ontario (Canada).
Isomer-specific bioaccumulation and trophic transfer of Dechlorane Plus in the freshwater food web from a highly
Isomers of Dechlorane Plus flame retardant in the eggs of herring gulls (Larus argentatus) from the Laurentian Gre
Levels and isomer profiles of dechlorane plus in Chinese air.
Detection of dechlorane plus in residential indoor dust in the city of Ottawa, Canada.
Dechlorane plus, a chlorinated flame retardant, in the Great Lakes.
Severe contamination and time trends of legacy and novel halogenated flame retardants in multiple environment
Long-term trends of airborne halogenated flame retardants (HFRs) by means of tree leaf and shoot analyses.
Investigating the presence of emerging and legacy POPs in European domestic air.
Measuring exposure of e-waste dismantlers in Dhaka Bangladesh to organophosphate esters and halogenated fla
Assessment of persistent organic pollutants (POPs) in sediments of the Eastern Indian Ocean.
Alternative halogenated flame retardants (AHFRs) in green mussels from the south China sea.
Changes in plasma biochemistry in breeding ring-billed gulls: Effects of anthropogenic habitat use and contaminar
Spatial distribution and hazard of halogenated flame retardants and polychlorinated biphenyls to common kingfis
Halogenated flame retardants in the sediments of the Chinese Yellow Sea and East China Sea.
Halogenated flame retardants in mangrove sediments from the Pearl River Estuary, South China: Comparison with
Flame retardant concentrations and profiles in wild birds associated with landfill: A critical review.
Pine needles as biomonitors of polybrominated diphenyl ethers and emerging flame retardants in the atmosphere
Measurement of legacy and emerging flame retardants in indoor dust from a rural village (Kopawa) in Nepal: Impl
Amplification effect of haze on human exposure to halogenated flame retardants in atmospheric particulate matt
Halogenated organic pollutants in aquatic, amphibious, and terrestrial organisms from an e-waste site: Habitat-de
Temporal trends of Dechlorane Plus in air and precipitation around the North American Great Lakes.
Halogenated flame retardants in tree samples applied as bioindicators for atmospheric pollution.
High levels of medium-chain chlorinated paraffins and polybrominated diphenyl ethers on the inside of several ho
Temporal trends of halogenated flame retardants in the atmosphere of the Canadian Great Lakes Basin (2005-201
The presence and partitioning behavior of flame retardants in waste, leachate, and air particles from Norwegian w
Modelling oral up-take of hydrophobic and super-hydrophobic chemicals in fish.
Occurrence of selected halogenated flame retardants in Belgian foodstuff.
Regulated and Unregulated Halogenated Flame Retardants in Peregrine Falcon Eggs from Greenland.
Environmental concentration and atmospheric deposition of halogenated flame retardants in soil from Nepal: Sou
Japanese quail (Coturnix japonica) liver and thyroid gland histopathology as a result of in ovo exposure to the flam
Occurrence, distribution and bioaccumulation behaviour of hydrophobic organic contaminants in a large-scale con
A miniature bird-borne passive air sampler for monitoring halogenated flame retardants.
Temporal trends of PBDEs and emerging flame retardants in belugas from the St. Lawrence Estuary (Canada) and
Halogenated organic pollutants in marine biota from the Xuande Atoll, South China Sea: Levels, biomagnification a

Organic contaminants and heavy metals in indoor dust from e-waste recycling, rural, and urban areas in South Chi
Development of plastic disks containing flame retardants for elucidating changes in their concentrations due to si
Identification of Marbon in the Indiana Harbor and Ship Canal.
Atmospheric deposition of PBDEs and DPs in Dongjiang River Basin, South China.
Estimation of human exposure to halogenated flame retardants through dermal adsorption by skin wipe.
Concentrations of polybrominated diphenyl ethers and alternative flame retardants in surface soils and river sedi
Occurrence of PBDEs and alternative halogenated flame retardants in sewage sludge from the industrial city of Gu
Relationship between legacy and emerging organic pollutants in Antarctic seabirds and their foraging ecology as s
New insight into the levels, distribution and health risk diagnosis of indoor and outdoor dust-bound FRs in colder,
Plant selective uptake of halogenated flame retardants at an e-waste recycling site in southern China.
Non-PBDE halogenated flame retardants in Canadian indoor house dust: sampling, analysis, and occurrence.
Short-term fasts increase levels of halogenated flame retardants in tissues of a wild incubating bird.
Five-year trends of selected halogenated flame retardants in the atmosphere of Northeast China.
Bioaccumulation and biomagnification of halogenated organic pollutants in mangrove biota from the Pearl River E
Temporal trends in classical and alternative flame retardants in bird eggs from Doñana Natural Space and surroun
Assessing the combined influence of TOC and black carbon in soil-air partitioning of PBDEs and DPs from the Indu
Particle size distribution of halogenated flame retardants and implications for atmospheric deposition and transp
The spatial distribution of organochlorine pesticides and halogenated flame retardants in the surface sediments o
Current levels and composition profiles of emerging halogenated flame retardants and dehalogenated products ir
Distribution patterns of brominated, chlorinated, and phosphorus flame retardants with particle size in indoor and
Species-specific accumulation of halogenated flame retardants in eggs of terrestrial birds from an ecological static
Primary investigation on contamination pattern of legacy and emerging halogenated organic pollutions in freshwa
Novel flame retardants in urban-feeding ring-billed gulls from the St. Lawrence River, Canada.
Levels of dechlorane plus and polybrominated diphenylethers in human milk in two Canadian cities.
Exposure to flame retardants in electronics recycling sites.
Identification and determination of hexachlorocyclopentadienyl-dibromocyclooctane (HCDBCO) in residential ind
Dechlorane plus and other flame retardants in a sediment core from Lake Ontario.
Isomers of dechlorane plus in Lake Winnipeg and Lake Ontario food webs.
Probing Legacy and Alternative Flame Retardants in the Air of Chinese Cities.
Halogenated flame retardants in surface sediments from fourteen estuaries, South China.
Chlorinated and brominated persistent compounds in hard coral, soft coral, and parrotfish from remote Mascarer
Effect of laying sequence and selection of maternal tissues in assessment of maternal transfer of organohalogena
Legacy and novel flame retardants from indoor dust in Antarctica: Sources and human exposure.
Human exposure to halogenated and organophosphate flame retardants through informal e-waste handling activ
Legacy and emerging flame retardants (FRs) in the urban atmosphere of Pakistan: Diurnal variations, gas-particle p
Halogenated and organophosphorous flame retardants in surface soils from an e-waste dismantling park and its s
Evidence for complex sources of persistent halogenated compounds in birds from the south China sea.
Uptake of halogenated organic compounds (HOCs) into peanut and corn during the whole life cycle grown in an a
Distribution behaviour in body compartments and in ovo transfer of flame retardants in North American Great La
Human exposure to legacy and emerging flame retardants in indoor dust: A multiple-exposure assessment of PBD
Polybrominated diphenyl ethers and alternative halogenated flame retardants in mangrove plants from Futian Na
Airborne brominated, chlorinated and organophosphate ester flame retardants inside the buildings of the Indian s
Occurrence of organic pollutants in plastics on beach: Stranded foams can be sources of pollutants in islands.
Association between Thyroid Function and Exposures to Brominated and Organophosphate Flame Retardants in R
Persistent organic pollutants (POPs) in oriental magpie-robins from e-waste, urban, and rural sites: Site-specific bi
Integrated exposure assessment of northern goshawk (Accipiter gentilis) nestlings to legacy and emerging organic
Recent findings of halogenated flame retardants (HFR) in the German and Polar environment.

Bioaccumulation of Selected Halogenated Organic Flame Retardants in Lake Ontario.
Legacy and Currently Used Organic Contaminants in Human Hair and Hand Wipes of Female E-Waste Dismantling
Legacy PBDEs and NBFRs in sediments of the tidal River Thames using liquid chromatography coupled to a high re
Halogenated organic pollutants in sediments and organisms from mangrove wetlands of the Jiulong River Estuary,
A clean-up method for determination of multi-classes of persistent organic pollutants in sediment and biota samp
(Persistent) Organic pollutants in Germany: results from a pilot study within the 2015 moss survey.
Legacy and alternative flame retardants in house dust and hand wipes from South China.
Characterization of brominated, chlorinated, and phosphate flame retardants in San Francisco Bay, an urban estua
Particle-phase concentrations and sources of legacy and novel flame retardants in outdoor and indoor environme
The non-negligible environmental risk of recycling halogenated flame retardants associated with plastic regenerat
Effect of Long-time Heating for Elements from Flame Retardants in Acrylonitrile Butadiene Styrene and Polycarbo
White-tailed eagle (Haliaeetus albicilla) feathers from Norway are suitable for monitoring of legacy, but not emerg
Assessing bioaccumulation behaviour of hydrophobic organic contaminants in a tropical urban catchment.
Environmental risk assessment of perfluoroalkyl substances and halogenated flame retardants released from bios
Impact of particle size on distribution and human exposure of flame retardants in indoor dust.
E-Waste Driven Pollution in Pakistan: The First Evidence of Environmental and Human Exposure to Flame Retarda
Legacy and emerging organohalogenated contaminants in wild edible aquatic organisms: Implications for bioaccu
From headwaters to estuary: Distribution and fate of halogenated flame retardants (HFRs) in a river basin near th
Occurrence and source apportionment of atmospheric halogenated flame retardants in Lhasa City in the Tibetan P
Legacy and emerging halogenated flame retardants in the middle and lower stream of the Yellow River.
Miniaturised sample preparation method for the multiresidual determination of regulated organohalogenated po
Determining equilibrium partition coefficients between lipid/protein and polydimethylsiloxane for highly hydroph
Flame Retardant Chemicals in College Dormitories: Flammability Standards Influence Dust Concentrations.
Polyhalogenated compounds (chlorinated paraffins, novel and classic flame retardants, POPs) in dishcloths after t
New halogenated flame retardants in the atmosphere of nine urban areas in China: Pollution characteristics, sour
Legacy and emerging flame retardants (FRs) in the freshwater ecosystem: A review.
Occurrence and Concentrations of Halogenated Flame Retardants in the Atmospheric Fine Particles in Chinese Cit
Dermal uptake and percutaneous penetration of ten flame retardants in a human skin ex vivo model.
Organohalogen pollutants in surface particulates from workshop floors of four major e-waste recycling sites in Ch
[Concentrations and Partitioning of Halogenated Flame Retardants in Industrial Water of Dongjiang River].
Habitat- and species-dependent accumulation of organohalogen pollutants in home-produced eggs from an electr
First insight into the levels and distribution of flame retardants in potable water in Pakistan: An underestimated p
Trends in the levels of halogenated flame retardants in the Great Lakes atmosphere over the period 2005-2013.
Latitudinal exposure to DDTs, HCB, PCBs, PBDEs and DP in giant petrels (Macronectes spp.) across the Southern O
Emerging halogenated flame retardants and hexabromocyclododecanes in food samples from an e-waste process
Persistent halogenated compounds in fish from rivers in the Pearl River Delta, South China: Geographical pattern
Concentrations of legacy and emerging flame retardants in air and soil on a transect in the UK West Midlands.
Distributions and compositions of old and emerging flame retardants in the rhizosphere and non-rhizosphere soil
A review on current knowledge and future prospects of organohalogen contaminants (OHCs) in Asian birds.
Spatial and temporal comparisons of legacy and emerging flame retardants in herring gull eggs from colonies spar
Legacy and emerging halogenated organic pollutants in marine organisms from the Pearl River Estuary, South Chin
Co-extraction and simultaneous determination of multi-class hydrophobic organic contaminants in marine sedime
Halogenated flame retardants in the Great Lakes environment.
Bioaccumulation and translocation of polyhalogenated compounds in rice (Oryza sativa L.) planted in paddy soil co
Multi-residue analysis of legacy POPs and emerging organic contaminants in Singapore's coastal waters using gas
An eight year (2005-2013) temporal trend of halogenated organic pollutants in fish from the Pearl River Estuary, S
A broad cocktail of environmental pollutants found in eggs of three seabird species from remote colonies in Norw

Flame retardants and organochlorines in indoor dust from several e-waste recycling sites in South China: composi
Spatial distribution of old and emerging flame retardants in Chinese forest soils: sources, trends and processes.
Locating POPs Sources with Tree Bark.
Levels of polybrominated diphenyl ethers and novel flame retardants in microenvironment dust from Egypt: an as
Global trends of research on emerging contaminants in the environment and humans: a literature assimilation.
Halogenated flame retardants in baby food from the United States and from China and the estimated dietary inta
A review of new and current-use contaminants in the Arctic environment: evidence of long-range transport and ir
Health risk characterization for resident inhalation exposure to particle-bound halogenated flame retardants in a t
Brominated flame retardants in matched serum samples from Swedish first-time mothers and their toddlers.
Halogenated flame retardants during egg formation and chicken embryo development: maternal transfer, possibl
Species-specific bioaccumulation of halogenated organic pollutants and their metabolites in fish serum from an e-
Organophosphate and halogenated flame retardants in atmospheric particles from a European Arctic site.
Bioaccumulation of highly hydrophobic organohalogen flame retardants from sediments: application of toxicokine
Concentrations and relationships between classes of persistent halogenated organic compounds in pooled humar
Organohalogen contamination in passerine birds from three metropolises in China: geographical variation and its
Changes of accumulation profiles from PBDEs to brominated and chlorinated alternatives in marine mammals fro
Polybrominated diphenyl ethers and alternative flame retardants in air and precipitation samples from the northe
Atmospheric deposition of persistent organic pollutants and chemicals of emerging concern at two sites in northe
European starlings (Sturnus vulgaris) suggest that landfills are an important source of bioaccumulative flame reta
Occurrence and biomagnification of organohalogen pollutants in two terrestrial predatory food chains.
Matrix solid-phase dispersion combined with gas chromatography-mass spectrometry for the determination of fif
Reactive oxygen species alteration of immune cells in local residents at an electronic waste recycling site in northe
Concentrations and trends of halogenated flame retardants in the pooled serum of residents of Laizhou Bay, Chin
Bioaccumulation of polybrominated diphenyl ethers and several alternative halogenated flame retardants in a sm
Brominated and chlorinated flame retardants in tree bark from around the globe.
After the PBDE phase-out: a broad suite of flame retardants in repeat house dust samples from California.
Flame retardants in eggs of American kestrels and European starlings from southern Lake Ontario region (North A
Brominated and chlorinated flame retardants in San Francisco Bay sediments and wildlife.
Halogenated flame retardants in home-produced eggs from an electronic waste recycling region in South China: le
Flame retardants in eggs of four gull species (Laridae) from breeding sites spanning Atlantic to Pacific Canada.
Measurement of flame retardants and triclosan in municipal sewage sludge and biosolids.
[Concentration and emission fluxes of halogenated flame retardants in sewage from sewage outlet in Dongjiang R
Atmospheric concentrations of halogenated flame retardants at two remote locations: the Canadian High Arctic a
Atmospheric deposition of halogenated flame retardants at urban, e-waste, and rural locations in southern China.
Flame retardants in the serum of pet dogs and in their food.
Contaminant pattern and bioaccumulation of legacy and emerging organhalogen pollutants in the aquatic biota fr
Evaluation of tree bark as a passive atmospheric sampler for flame retardants, PCBs, and organochlorine pesticide
Polybrominated diphenyl ethers (PBDEs) and other flame retardants in the atmosphere and water from Taihu Lak
Flame retardants and organochlorine pollutants in bald eagle plasma from the Great Lakes region.
Flame-retardants and other organohalogens detected in sewage sludge by electron capture negative ion mass spe
Flame retardants are the suspected source of a new compound in the environment.
The analysis of halogenated flame retardants by GC-HRMS in environmental samples.
Brominated and chlorinated flame retardants in Lake Ontario, Canada, lake trout (Salvelinus namaycush) betweer
Flame retardants in the atmosphere near the Great Lakes.
New data on a widely used flame retardant.
Current-use flame retardants in the eggs of herring gulls (Larus argentatus) from the Laurentian Great Lakes.

Provided by the US EPA's Center for Computational Toxicology and Exposure

Review	PMC	Authors	Journal
0	0	L'Homme B, Calaprice C, Calvano CD, Za	Chemosphere
0	0	Guo J, Venier M, Salamova A, Hites RA	The Science of the total environment
1	0	Feo ML, Barón E, Eljarrat E, Barceló D	Analytical and bioanalytical chemistry
0	0	Kim J, Son MH, Shin ES, Choi SD, Chang	Environmental pollution (Barking, Essex : 1987
0	0	Chen X, Zhu Y, Huang Q, Liu J, Liu B, Zha	Environmental science and pollution research
0	0	Tao W, Zhou Z, Shen L, Zhao B	Environmental pollution (Barking, Essex : 1987
0	0	Jia H, Sun Y, Liu X, Yang M, Wang D, Qi H	Environmental science & technology
0	0	Wang DG, Yang M, Qi H, Sverko E, Ma V	Environmental science & technology
0	0	Sverko E, McCarry B, McCrindle R, Braze	Environmental science & technology
0	0	Kim J, Son MH, Kim J, Suh J, Kang Y, Cha	Journal of hazardous materials
0	0	Brasseur C, Pirard C, Scholl G, De Pauw	Environment international
0	0	Sun J, Zhang A, Fang L, Wang J, Liu W	The Science of the total environment
0	0	Peng H, Zhang K, Wan Y, Hu J	Environmental science & technology
0	0	Abdel Malak I, Cariou R, Guiffard I, Véni	Chemosphere
0	0	Von Eyken A, Pijuan L, Martí R, Blanco M	Chemosphere
0	0	Yang R, Zhang S, Li X, Luo D, Jing C	Chemosphere
0	0	Zhao L, Gong N, Mi D, Luan C, Shao K, Ji	Chemosphere
0	0	Guerra P, Fernie K, Jiménez B, Pacepavi	Environmental science & technology
0	0	Fromme H, Thomsen C, Aschenbrenner	International journal of hygiene and environm
0	0	Boyles E, Tan H, Wu Y, Nielsen CK, Shen	Environmental pollution (Barking, Essex : 1987
0	0	Peng H, Wan Y, Zhang K, Sun J, Hu J	Environmental science & technology
0	0	Shen L, Reiner EJ, Helm PA, Marvin CH,	Environmental science & technology
0	0	Shen L, Reiner EJ, MacPherson KA, Kolic	Environmental science & technology
0	0	Sales C, Poma G, Malarvannan G, Porto	Analytical and bioanalytical chemistry
0	0	Rjabova J, Bartkevics V, Zacs D	Chemosphere
0	0	Sühring R, Freese M, Schneider M, Schu	The Science of the total environment
0	0	Shen L, Jobst KJ, Reiner EJ, Helm PA, Mc	Environmental science & technology
0	0	Chen CL, Tsai DY, Ding WH	Food chemistry
0	0	Sühring R, Möller A, Freese M, Pohlmar	Chemosphere
0	0	Shen L, Jobst KJ, Helm PA, Reiner EJ, Mc	Analytical and bioanalytical chemistry
0	0	Wang DG, Alaee M, Sverko E, Li YF, Reir	Journal of environmental monitoring : JEM
0	0	Li H, Song A, Liu H, Li Y, Liu M, Sheng G,	Environmental pollution (Barking, Essex : 1987
1	1	Ghelli E, Cariou R, Dervilly G, Pagliuca G	International journal of environmental researc
0	0	Zacs D, Ikkere LE, Bartkevics V	Chemosphere
0	0	Zhou S, Fu J, He H, Fu J, Tang Q, Dong M	Chemosphere
0	0	Wang L, Jia H, Liu X, Yang M, Hong W, S	Ecotoxicology and environmental safety

0	0	Pizzochero AC, de la Torre A, Sanz P, Na	The Science of the total environment
0	0	Wu JP, Chen XY, Si-Kang W, Sun Y, Feng	Chemosphere
0	0	Brazeau AL, Pena-Abaurrea M, Shen L, F	Environmental science & technology
0	0	Kim JT, Choi YJ, Barghi M, Yoon YJ, Kim .	Environmental pollution (Barking, Essex : 1987
0	0	Brasseur C, Pirard C, L'homme B, De Pa	Rapid communications in mass spectrometry
0	0	Fromme H, Cequier E, Kim JT, Hanssen I	Environment international
0	0	Na G, Wei W, Zhou S, Gao H, Ma X, Qiu	Journal of environmental sciences (China)
0	0	Zheng XB, Luo XJ, Zeng YH, Wu JP, Mai E	Chemosphere
0	0	Muñoz-Arnanz J, Roscales JL, Vicente A,	Analytical and bioanalytical chemistry
0	0	Zhang Y, Wu JP, Luo XJ, Wang J, Chen SJ	Environmental pollution (Barking, Essex : 1987
0	0	Yang R, Wei H, Guo J, McLeod C, Li A, St	Environmental science & technology
0	0	Kim JT, Choi YJ, Barghi M, Kim JH, Jung J	Journal of hazardous materials
0	0	Yin JF, Li JF, Li XH, Yang YL, Qin ZF	The Science of the total environment
0	0	Chen W, Li J, Dong Z, Bao J, Zhang A, Sh	Chemosphere
0	1	Gao H, Na G, Yao Y, Li R, Gao Y, Zhang Z	International journal of environmental researc
0	0	Abdel Malak I, Cariou R, Vénisseau A, D	Chemosphere
0	0	Rjabova J, Viksna A, Zacs D	Chemosphere
0	0	Ma Y, Li P, Jin J, Wang Y, Wang Q	Environmental research
1	0	Wang P, Zhang Q, Zhang H, Wang T, Sur	Environment international
0	0	Chen K, Zheng J, Yan X, Yu L, Luo X, Pen	Chemosphere
0	0	Wang DG, Guo MX, Pei W, Byer JD, Wa	Chemosphere
0	0	Yu D, Yang J, Li T, Feng J, Xian Q, Zhu J	Environmental science and pollution research
0	0	Li L, Wang W, Lv Q, Ben Y, Li X	Journal of environmental sciences (China)
0	0	Barón E, Santín G, Eljarrat E, Barceló D	Journal of hazardous materials
0	0	Sühring R, Byer J, Freese M, Pohlmann J	Chemosphere
0	0	Cequier E, Marcé RM, Becher G, Thoms	Journal of chromatography. A
0	0	Wang S, Huang J, Yang Y, Yu G, Deng S,	Journal of hazardous materials
0	0	Ben YJ, Li XH, Yang YL, Li L, Di JP, Wang	Environmental pollution (Barking, Essex : 1987
0	0	Sun Y, Luo X, Wu J, Mo L, Chen S, Zhang	Chemosphere
0	0	Barón E, Eljarrat E, Barceló D	Journal of chromatography. A
0	0	Wang J, Tian M, Chen SJ, Zheng J, Luo X	Environmental toxicology and chemistry
0	0	Chen SJ, Tian M, Wang J, Shi T, Luo Y, Lu	Environmental pollution (Barking, Essex : 1987
0	0	Zhang W, Huang J, Yu G, Deng S, Zhu W	Chemosphere
0	0	Shen L, Reiner EJ, MacPherson KA, Kolic	Environmental science & technology
0	0	Ren G, Yu Z, Ma S, Li H, Peng P, Sheng G	Environmental science & technology
1	0	Martinez G, Niu J, Takser L, Bellenger JP	Environmental pollution (Barking, Essex : 1987
0	0	Zhang J, Zhao J, Sun Y, Xin M, Zhang D, I	Environmental science and pollution research
0	0	Zhen X, Li Y, Wang X, Liu L, Li Y, Tian C, F	Environmental pollution (Barking, Essex : 1987
0	0	Zhang Z, Tong X, Xing Y, Ma J, Jiang R, S	Marine pollution bulletin
1	0	Zafar MI, Kali S, Ali M, Riaz MA, Naz T, I	Environmental science and pollution research
0	0	Zhang G, Ren Q, Ma S, Wu J, Yang X, Yu	Environment international
0	0	Li B, Chen J, Du Q, Wang B, Qu Y, Chang	Chemosphere
0	0	Wang D, Jia H, Hong WJ, Xue X, Sun Y, L	Environmental science and pollution research
0	0	Guo J, Li Z, Ranasinghe P, Rockne KJ, Stu	Journal of hazardous materials
0	0	Chen X, Chen Y, Huang C, Dong Q, Rope	Ecotoxicology and environmental safety
0	0	Liu X, Wu Y, Zhang X, Shen L, Brazeau Al	Environmental science & technology
0	0	Li ZR, Luo XJ, Luo YL, Zeng YH, Mai BX	Environmental pollution (Barking, Essex : 1987

0	0	Wu Y, Tan H, Zhou C, Crimmins BS, Hols	Environmental science & technology
0	0	Sun J, Wu Y, Tao N, Lv L, Yu X, Zhang A,	Environmental pollution (Barking, Essex : 1987
0	0	Desjardins CF, Mazerolle MJ, Verreault .	Environmental pollution (Barking, Essex : 1987
0	0	Qiu YW, Qiu HL, Zhang G, Li J	The Science of the total environment
0	0	Carlsson P, Vrana B, Sobotka J, Borgå K,	Chemosphere
0	0	Sun J, Xu Y, Zhou H, Zhang A, Qi H	The Science of the total environment
0	0	Gong N, Shao K, Han X, Zhang Y, Sun Y	Chemosphere
0	0	Li N, Chen XW, Deng WJ, Giesy JP, Zhen	Chemosphere
0	0	Tang B, Luo XJ, Huang CC, Sun RX, Wang	The Science of the total environment
0	0	Qiao L, Zheng XB, Yan X, Wang MH, Zhe	Ecotoxicology and environmental safety
0	0	Wang G, Peng J, Hao T, Feng L, Liu Q, Li	Environmental pollution (Barking, Essex : 1987
0	0	Riddell N, van Bavel B, Ericson Jogsten I	Talanta
0	0	Gagné PL, Fortier M, Fraser M, Parent L	Aquatic toxicology (Amsterdam, Netherlands)
0	0	Liu X, Bing H, Chen Y, Li J, Wu Y, Zhang (	Environmental geochemistry and health
0	0	Chen X, Dong Q, Chen Y, Zhang Z, Huang	Environmental pollution (Barking, Essex : 1987
0	0	Wang G, Peng J, Hao T, Liu Y, Zhang D, L	The Science of the total environment
0	0	Sun YX, Zhang ZW, Xu XR, Hao QW, Hu Y	Archives of environmental contamination and
0	0	Kang H, Moon HB, Choi K	Chemosphere
0	0	Wu PF, Yu LL, Li L, Zhang Y, Li XH	Chemosphere
0	0	Yang Y, Ji F, Cui Y, Li M	Chemosphere
0	0	Kim JT, Son MH, Kang JH, Kim JH, Jung J	Environmental science & technology
0	0	Li WL, Qi H, Ma WL, Liu LY, Zhang Z, Zhu	Chemosphere
0	0	Peng Y, Wu JP, Tao L, Mo L, Zheng XB, T	The Science of the total environment
0	0	Dou J, Jin Y, Li Y, Wu B, Li M	Chemosphere
0	0	Xiang N, Chen L, Meng XZ, Li YL, Liu Z, W	The Science of the total environment
0	0	He C, Jin J, Wang Y, Ma Z, He S, Li M	Environmental toxicology and chemistry
0	0	Zhang L, Ji F, Li M, Cui Y, Wu B	Journal of hazardous materials
0	0	Kakimoto K, Nagayoshi H, Akutsu K, Kor	Environmental science and pollution research
0	0	Fang M, Kim JC, Chang YS	The Science of the total environment
0	0	He MJ, Luo XJ, Wu JP, Chen SJ, Wei SQ,	The Science of the total environment
0	0	Houde M, Berryman D, de Lafontaine Y,	The Science of the total environment
0	0	Zhou SN, Siddique S, Lavoie L, Takser L,	Environment international
0	0	Liang X, Li W, Martyniuk CJ, Zha J, Wang	Aquatic toxicology (Amsterdam, Netherlands)
0	0	Zeng YH, Luo XJ, Tang B, Zheng XB, Mai	Ecotoxicology and environmental safety
0	0	Wang H, Huang J, Zhang K, Yu Y, Liu K, Y	Journal of hazardous materials
0	0	Ben YJ, Li XH, Yang YL, Li L, Zheng MY, W	Environmental science & technology
0	0	Kakimoto K, Nagayoshi H, Takagi S, Aku	Ecotoxicology and environmental safety
0	0	Syed JH, Malik RN, Li J, Wang Y, Xu Y, Zh	Chemosphere
0	0	Yan X, Zheng J, Chen KH, Yang J, Luo XJ,	Environment international
0	0	Möller A, Xie Z, Cai M, Zhong G, Huang I	Environmental science & technology
1	0	Xian Q, Siddique S, Li T, Feng YL, Takser	Environment international
0	0	Zhao Z, Zhong G, Möller A, Xie Z, Sturm	Chemosphere
0	0	Zheng J, Wang J, Luo XJ, Tian M, He LY, Y	Environmental science & technology
0	0	Tomy GT, Thomas CR, Zidane TM, Muris	Environmental science & technology
0	0	Qiu X, Hites RA	Environmental science & technology
0	0	Sverko E, Tomy GT, Marvin CH, Zaruk D,	Environmental science & technology
0	0	Ayala-Cabrera JF, Lacorte S, Moyano E,	Analytical and bioanalytical chemistry

0	0	Chen W, Bao J, Bu T, Jin H, Liu Y, Li T, W	Environmental toxicology and chemistry
0	0	Pan HY, Li JF, Li XH, Yang YL, Qin ZF, Li JI	Environmental pollution (Barking, Essex : 1987
0	0	Li B, Chen J, Wang S, Qi P, Chang X, Cha	Ecotoxicology and environmental safety
0	0	Hansen KM, Fauser P, Vorkamp K, Chris	The Science of the total environment
0	0	Peshdary V, Styles G, Rigden M, Caldwe	Endocrinology
0	1	Soleman SR, Fujitani T, Fujii Y, Harada K	International journal of environmental researc
0	0	Cheng Y, Ding J, Liang X, Ji X, Xu L, Xie X,	Environmental science & technology
0	0	Li B, Qi P, Qu Y, Wang B, Chen J, Chang X	Drug and chemical toxicology
0	0	Cheng Y, Ding J, Xie X, Ji X, Zhang Y	Environmental science & technology
0	0	Blanco G, Sergio F, Frías Ó, Salinas P, Ta	Environmental research
0	0	Peshdary V, Calzadilla G, Landry A, Soris	International journal of obesity (2005)
0	0	Hou M, Wang Y, Zhao H, Zhang Q, Xie Q	Chemosphere
0	0	Neugebauer F, Dreyer A, Lohmann N, Ko	Analytical and bioanalytical chemistry
0	0	Briels N, Løseth ME, Ciesielski TM, Mala	Ecotoxicology and environmental safety
0	0	Xu P, Tao B, Zhou Z, Fan S, Zhang T, Liu A	Environmental pollution (Barking, Essex : 1987
0	0	Na G, Yao Y, Gao H, Li R, Ge L, Titaley IA	Marine pollution bulletin
0	0	Jin X, Lee S, Jeong Y, Yu JP, Baek WK, Sh	Environmental pollution (Barking, Essex : 1987
0	0	Deng WJ, Zheng HL, Tsui AK, Chen XW	Environment international
0	0	Li H, Liu H, Mo L, Sheng G, Fu J, Peng P	Environmental science and pollution research
0	0	Barón E, Dissanayake A, Vilà-Cano J, Crc	Environmental science & technology
0	0	Xu P, Tao B, Ye Z, Qi L, Ren Y, Zhou Z, Li	Talanta
0	0	Yu Y, Hung H, Alexandrou N, Roach P, N	Environmental science & technology
0	0	Vorkamp K, Bossi R, Riget FF, Skov H, Sc	Environmental pollution (Barking, Essex : 1987
0	0	Cequier E, Marcé RM, Becher G, Thoms	Environment international
0	0	Mahmood A, Malik RN, Li J, Zhang G	Archives of environmental contamination and
0	0	Ma J, Qiu X, Liu D, Zhao Y, Yang Q, Fang	Environmental science and pollution research
0	0	Law RJ, Losada S, Barber JL, Bersuder P,	Environment international
0	0	Zhang H, Wang P, Li Y, Shang H, Wang Y	Environmental science & technology
0	0	Xiao K, Wang P, Zhang H, Shang H, Li Y,	Environmental geochemistry and health
0	0	He C, Jin J, Ma ZH, Wang Y, Zhaxi ZM, M	Huan jing ke xue= Huanjing kexue
0	0	Chabot-Giguère B, Letcher RJ, Verreault	Environment international
0	0	Wu JP, She YZ, Zhang Y, Peng Y, Mo L, Lu	Environmental pollution (Barking, Essex : 1987
0	0	Li Y, Yu L, Zhu Z, Dai J, Mai B, Wu J, War	Environmental toxicology and chemistry
0	0	Tomy GT, Sverko E, Palace V, Rosenberg	Environmental toxicology and chemistry
0	0	Chen D, Wang Y, Yu L, Luo X, Mai B, Li S	Environmental pollution (Barking, Essex : 1987
0	0	Wang DG, Alaee M, Byer JD, Brimble S,	The Science of the total environment
0	0	Mo L, Wu JP, Luo XJ, Sun YX, Zheng XB,	Environmental pollution (Barking, Essex : 1987
0	0	Li Y, Yu L, Wang J, Wu J, Mai B, Dai J	Chemosphere
0	0	de la Torre A, Alonso MB, Martínez MA,	Environmental science & technology
0	0	Wu B, Liu S, Guo X, Zhang Y, Zhang X, Li	Environmental science & technology
0	0	Kakimoto K, Nagayoshi H, Yoshida J, Aku	Chemosphere
0	0	Möller A, Xie Z, Cai M, Sturm R, Ebingha	Environmental science & technology
0	0	Salamova A, Hites RA	Environmental science & technology
0	0	Shi Z, Wang Y, Feng J, Huang P, Wu Y	Se pu = Chinese journal of chromatography
0	0	Zhang Y, Wu JP, Luo XJ, Sun YX, Mo L, Cl	Environment international
1	0	Sverko E, Tomy GT, Reiner EJ, Li YF, McC	Environmental science & technology
0	0	Yu Z, Liao R, Li H, Mo L, Zeng X, Sheng G	Environmental pollution (Barking, Essex : 1987

0	0	Crump D, Chiu S, Gauthier LT, Hickey NJ	Comparative biochemistry and physiology. To
0	0	Muñoz-Arnanz J, Sáez M, Hiraldo F, Bao	Environment international
0	0	Zhou SN, Reiner EJ, Marvin CH, Helm PA	Rapid communications in mass spectrometry
0	0	Zhang XL, Luo XJ, Liu HY, Yu LH, Chen SJ	Environmental science & technology
0	0	de la Torre A, Sverko E, Alaee M, Martín	Chemosphere
0	0	Möller A, Xie Z, Sturm R, Ebinghaus R	Environmental science & technology
0	0	Brock WJ, Schroeder RE, McKnight CA, V	International journal of toxicology
0	0	Ma WL, Liu LY, Qi H, Sun DZ, Shen JM, W	Environment international
0	0	Yu Z, Lu S, Gao S, Wang J, Li H, Zeng X, S	Environmental pollution (Barking, Essex : 1987
0	0	Wang B, Iino F, Huang J, Lu Y, Yu G, Mor	Chemosphere
0	0	Kang JH, Kim JC, Jin GZ, Park H, Baek SY,	Chemosphere
0	0	Qi H, Liu L, Jia H, Li YF, Ren NQ, You H, S	Environmental science & technology
0	0	Sverko E, Reiner EJ, Tomy GT, McCrindle	Environmental science & technology
0	0	Wu JP, Zhang Y, Luo XJ, Wang J, Chen SJ	Environmental science & technology
0	0	Gauthier LT, Letcher RJ	Chemosphere
0	0	Ren N, Sverko E, Li YF, Zhang Z, Harner T	Environmental science & technology
0	0	Zhu J, Feng YL, Shoeib M	Environmental science & technology
0	0	Hoh E, Zhu L, Hites RA	Environmental science & technology
0	0	Lee S, Ra K, Moon HB	Chemosphere
0	0	Dreyer A, Neugebauer F, Lohmann N, R	Environmental pollution (Barking, Essex : 1987
0	0	de la Torre A, Sanz P, Navarro I, Martíne	The Science of the total environment
0	0	Wang Y, Peris A, Rifat MR, Ahmed SI, Ai	The Science of the total environment
0	0	Qiu YW, Wang DX, Zhang G	The Science of the total environment
0	0	Sun R, Pan C, Peng F, Wu Y, Chen X, Ma	Environmental research
0	0	Marteinson SC, Verreault J	Environment international
0	0	Peng Y, Wu J, Luo X, Zhang X, Giesy JP, M	Environment international
0	0	Li Y, Zhen X, Liu L, Tian C, Pan X, Tang J	Chemosphere
0	0	Hu Y, Pei N, Sun Y, Xu X, Zhang Z, Li H, W	Chemosphere
1	0	Tongue ADW, Reynolds SJ, Fernie KJ, Ha	Environmental pollution (Barking, Essex : 1987
0	0	Jia HH, Wang XT, Cheng HX, Zhou Y, Fu L	Environmental science and pollution research
0	0	Yadav IC, Devi NL, Singh VK, Li J, Zhang G	Ecotoxicology and environmental safety
0	0	Cao Z, Zhao L, Meng X, Liu X, Wu P, Fan	Journal of hazardous materials
0	0	Liu Y, Luo XJ, Huang LQ, Tao L, Zeng YH,	Environmental pollution (Barking, Essex : 1987
0	0	Olukunle OI, Lehman DC, Salamova A, V	The Science of the total environment
0	0	Dreyer A, Neugebauer F, Rüdel H, Klein	Chemosphere
0	0	Gallistl C, Sprengel J, Vetter W	The Science of the total environment
0	0	Shunthirasingham C, Alexandrou N, Bric	Environmental science. Processes & impacts
0	0	Morin NAO, Andersson PL, Hale SE, Arp	Journal of environmental sciences (China)
0	0	Larisch W, Goss KU	Environmental science. Processes & impacts
0	0	Poma G, Malysheva SV, Goscinny S, Ma	Chemosphere
0	0	Vorkamp K, Falk K, Møller S, Rigét FF, Sø	Environmental science & technology
0	0	Yadav IC, Devi NL, Li J, Zhang G	Environmental pollution (Barking, Essex : 1987
0	0	Jacobsen ML, Jaspers VLB, Ciesielski TM	Journal of toxicology and environmental healt
0	0	Wang Q, Kelly BC	Chemosphere
0	0	Sorais M, Rezaei A, Okeme JO, Diamond	The Science of the total environment
0	0	Simond AE, Houde M, Lesage V, Verreau	Environmental research
0	0	Sun YX, Hu YX, Zhang ZW, Xu XR, Li HX, Z	Marine pollution bulletin

0	0	He CT, Zheng XB, Yan X, Zheng J, Wang I	Ecotoxicology and environmental safety
0	0	Hanari N, Otake T, Itoh N, Wada A, Oha	Environmental monitoring and assessment
0	0	Guo J, Venier M, Romanak K, Westenbr	Environmental science & technology
0	0	Wu X, Wang Y, Hou M, Luo C, Zhao H, Z	Environmental science and pollution research
0	0	Liu X, Yu G, Cao Z, Wang B, Huang J, Der	Chemosphere
0	0	Matsukami H, Suzuki G, Someya M, Uch	Chemosphere
0	0	Wu Q, Li H, Kuo DTF, Chen S, Mai B, Li H	Environmental pollution (Barking, Essex : 1987
0	0	Mello FV, Roscales JL, Guida YS, Meneze	The Science of the total environment
0	0	Khan MU, Li J, Zhang G, Malik RN	Environmental pollution (Barking, Essex : 1987
0	0	Wang S, Wang Y, Luo C, Li J, Yin H, Zhan	Environmental pollution (Barking, Essex : 1987
0	0	Fan X, Kubwabo C, Rasmussen PE, Wu F	Environmental science and pollution research
0	0	Marteinson SC, Drouillard KG, Verreault	Environmental research
0	0	Li WL, Liu LY, Song WW, Zhang ZF, Qiao	The Science of the total environment
0	0	Sun YX, Zhang ZW, Xu XR, Hu YX, Luo XJ	Marine pollution bulletin
0	0	Barón E, Bosch C, Máñez M, Andreu A, S	Chemosphere
0	0	Ali U, Mahmood A, Syed JH, Li J, Zhang (	Environmental pollution (Barking, Essex : 1987
0	0	Okonski K, Degrendele C, Melymuk L, La	Environmental science & technology
0	0	Ma Y, Xie Z, Halsall C, Möller A, Yang H,	Chemosphere
0	0	Zeng L, Yang R, Zhang Q, Zhang H, Xiao I	Environmental science & technology
0	0	Cao Z, Xu F, Covaci A, Wu M, Wang H, Y	Environmental science & technology
0	0	Sun YX, Xu XR, Hao Q, Luo XJ, Ruan W, Z	Chemosphere
0	0	Ren G, Wang Z, Yu Z, Wang Y, Ma S, Wu	Environmental pollution (Barking, Essex : 1987
0	0	Gentes ML, Letcher RJ, Caron-Beaudoin	Environmental science & technology
0	0	Siddique S, Xian Q, Abdelouahab N, Tak	Environment international
0	0	Rosenberg C, Hämeilä M, Tornaeus J, Sä	The Annals of occupational hygiene
0	0	Zhu J, Hou Y, Feng YL, Shoeib M, Harner	Environmental science & technology
0	0	Qiu X, Marvin CH, Hites RA	Environmental science & technology
0	0	Tomy GT, Pleskach K, Ismail N, Whittle I	Environmental science & technology
0	0	Zhao S, Tian L, Zou Z, Liu X, Zhong G, Mo	Environmental science & technology
0	0	Xie J, Sun Y, Cheng Y, Chen Y, Chen L, Xi	Marine pollution bulletin
0	0	van der Schyff V, du Preez M, Blom K, K	Chemosphere
0	0	Li ZR, Luo XJ, Lin L, Zeng YH, Mai BX	Environmental pollution (Barking, Essex : 1987
0	0	Corsolini S, Metzdorff A, Baroni D, Rosc	Environmental research
1	0	Ma Y, Stubbings WA, Cline-Cole R, Harra	Environmental pollution (Barking, Essex : 1987
0	0	Syed JH, Iqbal M, Breivik K, Chaudhry M	The Science of the total environment
0	0	Ge X, Ma S, Zhang X, Yang Y, Li G, Yu Y	Environment international
0	0	Zhu C, Sun Y, Li D, Zheng X, Peng X, Zhu	Environmental research
0	0	Fan Y, Chen SJ, Li QQ, Zeng Y, Yan X, Ma	Environmental pollution (Barking, Essex : 1987
0	0	Smythe TA, Mattioli LC, Letcher RJ	Environmental pollution (Barking, Essex : 1987
0	0	Lee HK, Kang H, Lee S, Kim S, Choi K, Mo	The Science of the total environment
0	0	Hu Y, Sun Y, Pei N, Zhang Z, Li H, Wang \	Environmental pollution (Barking, Essex : 1987
0	0	Yadav IC, Devi NL, Kumar A, Li J, Zhang (	Ecotoxicology and environmental safety
0	0	Cao X, Wang L, Zhang Y, Li Y, Zhu C, Zhe	The Science of the total environment
0	0	Wang S, Romanak KA, Hendryx M, Salar	Environmental science & technology
0	0	Mo L, Zheng X, Zhu C, Sun Y, Yu L, Luo X	Ecotoxicology and environmental safety
0	0	Briels N, Torgersen LN, Castaño-Ortiz JN	Environmental research
0	0	Dreyer A, Neugebauer F, Lohmann N, Ru	Environmental pollution (Barking, Essex : 1987

0	0	Kurt-Karakus PB, Muir DCG, de Jourdan	Environmental toxicology and chemistry
0	0	Qiao L, Zheng XB, Zheng J, Chen SJ, Zhoı	Environmental science & technology
0	0	Ganci AP, Vane CH, Abdallah MA, Moeh	The Science of the total environment
0	0	Zhang Z, Pei N, Sun Y, Li J, Li X, Yu S, Xu	Environmental research
0	0	Zhao X, Cui T, Guo R, Liu Y, Wang X, An	Analytica chimica acta
0	1	Dreyer A, Nickel S, Schröder W	Environmental sciences Europe
0	0	Tang S, Tan H, Liu X, Chen D	The Science of the total environment
0	0	Sutton R, Chen D, Sun J, Greig DJ, Wu Y	The Science of the total environment
0	0	Reche C, Viana M, Querol X, Corcellas C	The Science of the total environment
0	0	Cao Z, Chen Q, Li X, Zhang Y, Ren M, Suı	The Science of the total environment
0	0	Ohata M, Kidokoro T, Otake T, Itoh N, H	Analytical sciences : the international journal (
0	0	Løseth ME, Briels N, Flo J, Malarvannan	The Science of the total environment
0	0	Wang Q, Kelly BC	Journal of hazardous materials
0	0	Navarro I, de la Torre A, Sanz P, Fernánc	Chemosphere
0	0	He RW, Li YZ, Xiang P, Li C, Cui XY, Ma L	Environmental research
0	0	Iqbal M, Syed JH, Breivik K, Chaudhry M	Environmental science & technology
0	0	Sun R, Luo X, Li QX, Wang T, Zheng X, Pe	The Science of the total environment
0	0	Zhen X, Tang J, Liu L, Wang X, Li Y, Xie Z	The Science of the total environment
0	0	Ma WL, Li WL, Zhang ZF, Liu LY, Song W	The Science of the total environment
0	0	Su X, Li Q, Feng J, Guo L, Sun J	The Science of the total environment
0	0	Roscales JL, Vicente A, Ramos L, Jiméne	Analytical and bioanalytical chemistry
0	0	Pei Y, Li H, You J	The Science of the total environment
0	0	Dodson RE, Rodgers KM, Carey G, Cede	Environmental science & technology
0	0	Gallistl C, Lok B, Schlienz A, Vetter W	The Science of the total environment
0	0	Li Q, Yang K, Li K, Liu X, Chen D, Li J, Zha	Environmental pollution (Barking, Essex : 1987
1	0	Iqbal M, Syed JH, Katsoyiannis A, Malik	Environmental research
0	0	Liu D, Lin T, Shen K, Li J, Yu Z, Zhang G	Environmental science & technology
0	0	Frederiksen M, Vorkamp K, Jensen NM,	Chemosphere
0	0	Zeng YH, Tang B, Luo XJ, Zheng XB, Peng	The Science of the total environment
0	0	He MJ, Li Q, Zhao JY, Wang DX	Huan jing ke xue= Huanjing kexue
0	0	Zeng YH, Luo XJ, Tang B, Mai BX	Environmental pollution (Barking, Essex : 1987
0	0	Khan MU, Li J, Zhang G, Malik RN	The Science of the total environment
0	0	Liu LY, Salamova A, Venier M, Hites RA	Environment international
0	0	Roscales JL, González-Solís J, Zango L, R	Environmental research
0	0	Tao F, Matsukami H, Suzuki G, Tue NM,	Environmental science. Processes & impacts
0	0	Sun R, Luo X, Tang B, Li Z, Wang T, Tao I	Environmental research
0	0	Drage DS, Newton S, de Wit CA, Harrad	Chemosphere
0	0	Wang S, Wang Y, Song M, Luo C, Li J, Zh	Environmental pollution (Barking, Essex : 1987
1	0	Abbasi NA, Malik RN, Frantz A, Jaspers \	The Science of the total environment
0	0	Su G, Letcher RJ, Moore JN, Williams LL,	Environmental research
0	0	Sun RX, Luo XJ, Tan XX, Tang B, Li ZR, M	Chemosphere
0	0	Zhang H, Bayen S, Kelly BC	Talanta
0	0	Venier M, Salamova A, Hites RA	Accounts of chemical research
0	0	Zhang Y, Luo XJ, Mo L, Wu JP, Mai BX, P	Chemosphere
0	0	Zhang H, Bayen S, Kelly BC	The Science of the total environment
0	0	Sun RX, Luo XJ, Tan XX, Tang B, Li ZR, M	Marine pollution bulletin
0	0	Huber S, Warner NA, Nygård T, Rember	Environmental toxicology and chemistry

0	0	Zheng X, Xu F, Chen K, Zeng Y, Luo X, Ch	Environment international
0	0	Zheng Q, Nizzetto L, Li J, Mulder MD, Sá	Environmental science & technology
0	0	Peverly AA, Salamova A, Hites RA	Environmental science & technology
0	0	Hassan Y, Shoeib T	The Science of the total environment
1	0	Bao LJ, Wei YL, Yao Y, Ruan QQ, Zeng EY	Environmental science and pollution research
0	0	Liu LY, Salamova A, Hites RA	Environmental science & technology
1	0	Vorkamp K, Rigét FF	Chemosphere
0	0	Luo P, Bao LJ, Wu FC, Li SM, Zeng EY	Environmental science & technology
0	0	Sahlström LM, Sellström U, de Wit CA, I	Environmental science & technology
0	0	Zheng XB, Luo XJ, Zeng YH, Wu JP, Chen	Environmental toxicology and chemistry
0	0	Zeng YH, Luo XJ, Zheng XB, Tang B, Wu .	Archives of environmental contamination and
0	0	Salamova A, Hermanson MH, Hites RA	Environmental science & technology
0	0	Li H, Zhang B, Wei Y, Wang F, Lydy MJ, Y	Environmental science & technology
0	0	Wang Y, Xu M, Jin J, He S, Li M, Sun Y	The Science of the total environment
0	0	Yu LH, Luo XJ, Liu HY, Zeng YH, Zheng XE	Environmental pollution (Barking, Essex : 1987
0	0	Zhu B, Lai NL, Wai TC, Chan LL, Lam JC, I	Environment international
0	0	Arinaitwe K, Muir DC, Kiremire BT, Fellir	Environmental science & technology
0	0	Newton S, Bidleman T, Bergknut M, Rac	Environmental science. Processes & impacts
0	0	Chen D, Martin P, Burgess NM, Champc	Environmental science & technology
0	0	Yu L, Luo X, Zheng X, Zeng Y, Chen D, W	Chemosphere
0	0	Villaverde-de-Sáa E, Valls-Cantenys C, Q	Journal of chromatography. A
0	0	Li R, Yang Q, Qiu X, Li K, Li G, Zhu P, Zhu	Environmental science & technology
0	0	He S, Li M, Jin J, Wang Y, Bu Y, Xu M, Ya	Environmental toxicology and chemistry
0	0	She YZ, Wu JP, Zhang Y, Peng Y, Mo L, Lu	Environmental pollution (Barking, Essex : 1987
0	0	Salamova A, Hites RA	Environmental science & technology
0	1	Dodson RE, Perovich LJ, Covaci A, Van d	Environmental science & technology
0	0	Chen D, Letcher RJ, Martin P	Journal of environmental monitoring : JEM
0	0	Klosterhaus SL, Stapleton HM, La Guard	Environment international
0	0	Zheng XB, Wu JP, Luo XJ, Zeng YH, She Y	Environment international
1	0	Chen D, Letcher RJ, Burgess NM, Champ	Environmental pollution (Barking, Essex : 1987
0	0	Davis EF, Klosterhaus SL, Stapleton HM	Environment international
0	0	Zeng YH, Luo XJ, Sun YX, Yu LH, Chen SJ,	Huan jing ke xue= Huanjing kexue
0	0	Xiao H, Shen L, Su Y, Barresi E, Dejong M	Environmental pollution (Barking, Essex : 1987
0	0	Tian M, Chen SJ, Wang J, Shi T, Luo XJ, M	Environmental science & technology
0	0	Venier M, Hites RA	Environmental science & technology
0	0	Zhang Y, Luo XJ, Wu JP, Liu J, Wang J, Ch	Environmental toxicology and chemistry
0	0	Salamova A, Hites RA	Environmental science & technology
0	0	Qiu X, Zhu T, Hu J	Chemosphere
0	0	Venier M, Wierda M, Bowerman WW, H	Chemosphere
0	0	La Guardia MJ, Hale RC, Harvey E, Chen	Environmental science & technology
0	0	Betts K	Environmental science & technology
0	0	Kolic TM, Shen L, Macpherson K, Fayez	Journal of chromatographic science
0	0	Ismail N, Gewurtz SB, Pleskach K, Whitt	Environmental toxicology and chemistry
0	0	Venier M, Hites RA	Environmental science & technology
0	0	Betts K	Environmental science & technology
0	0	Gauthier LT, Hebert CE, Weseloh DV, Le	Environmental science & technology

DOI	Volume	Issue	Pages	Pub Type	Batch Tag
10.1016/j.chemosphere.2015.07.043	139		525-33	jour	
10.1016/j.scitotenv.2016.11.063	583		1-9	jour	
10.1007/s00216-012-6161-x	404	9	2625-37	jour	
10.1016/j.envpol.2016.01.085	212		330-336	jour	
10.1007/s11356-018-2874-5	25	30	292-30300	jour	
10.1016/j.envpol.2015.07.043	206		361-8	jour	
10.1021/es103723h	45	7	2613-8	jour	
10.1021/es101224y	44	17	6608-13	jour	
10.1021/acs.est.5b03550	49	23	13862-7	jour	
10.1016/j.jhazmat.2014.04.032	275		19-25	jour	
10.1016/j.envint.2013.12.014	65		33-40	jour	
10.1016/j.scitotenv.2012.10.096	443		194-9	jour	
10.1021/es3025879	46	18	9907-13	jour	
10.1016/j.chemosphere.2019.06.148	235		492-497	jour	
10.1016/j.chemosphere.2015.10.001	144		1256-63	jour	
10.1016/j.chemosphere.2015.09.011	144		446-51	jour	
10.1016/j.chemosphere.2014.04.099	111		580-6	jour	
10.1021/es103333j	45	4	1284-90	jour	
10.1016/j.ijheh.2020.113593	229		113593	jour	
10.1016/j.envpol.2016.11.063	221		191-198	jour	
10.1021/es500229y	48	10	5458-66	jour	
10.1021/es104328r	45	8	3333-40	jour	
10.1021/es1027844	45	2	693-9	jour	
10.1007/s00216-017-0411-x	409	19	4507-4515	jour	
10.1016/j.chemosphere.2015.12.122	147		210-7	jour	
10.1016/j.scitotenv.2015.05.094	530-531		209-218	jour	
10.1021/es503089c	48	19	11170-7	jour	
10.1016/j.foodchem.2014.05.035	164		286-92	jour	
10.1016/j.chemosphere.2012.08.016	90	1	118-24	jour	
10.1007/s00216-012-6164-7	404	9	2737-48	jour	
10.1039/c1em10241a	13	11	3104-10	jour	
10.1016/j.envpol.2021.116902	279		116902	jour	
10.3390/ijerph18020690	18	2		jour	
10.1016/j.chemosphere.2018.01.105	197		680-690	jour	
10.1016/j.chemosphere.2017.06.104	184		1202-1208	jour	
10.1016/j.ecoenv.2012.07.018	84		262-7	jour	

10.1016/j.scitotenv.2019.05.213	682		719-728	jour
10.1016/j.chemosphere.2018.07.146	211		218-225	jour
10.1021/acs.est.8b00545	52	10	5619-5624	jour
10.1016/j.envpol.2017.12.080	235		302-311	jour
10.1002/rcm.7745	30	23	2545-2554	jour
10.1016/j.envint.2015.09.002	85		292-8	jour
10.1016/j.jes.2014.07.019	28		8-13	jour
10.1016/j.chemosphere.2014.04.104	114		241-6	jour
10.1007/s00216-012-6326-7	404	9	2765-73	jour
10.1016/j.envpol.2011.07.026	159	12	3647-52	jour
10.1021/es201019m	45	12	5156-63	jour
10.1016/j.jhazmat.2020.124141	405		124141	jour
10.1016/j.scitotenv.2019.134391	700		134391	jour
10.1016/j.chemosphere.2019.05.155	231		378-384	jour
10.3390/ijerph15102312	15	10		jour
10.1016/j.chemosphere.2018.05.101	207		413-420	jour
10.1016/j.chemosphere.2017.10.095	191		597-606	jour
10.1016/j.envres.2017.02.010	155		116-122	jour
10.1016/j.envint.2015.12.026	88		206-220	jour
10.1016/j.chemosphere.2014.11.058	123		43-7	jour
10.1016/j.chemosphere.2014.09.057	118		293-300	jour
10.1007/s11356-014-3794-7	22	9	6601-9	jour
10.1016/S1001-0742(13)60447-7	26	3	636-42	jour
10.1016/j.jhazmat.2013.10.069	265		288-95	jour
10.1016/j.chemosphere.2013.10.096	116		104-11	jour
10.1016/j.chroma.2013.08.067	1310		126-32	jour
10.1016/j.jhazmat.2013.04.034	260		16-23	jour
10.1016/j.envpol.2012.09.028	173		176-81	jour
10.1016/j.chemosphere.2012.05.089	89	4	445-51	jour
10.1016/j.chroma.2012.05.079	1248		154-60	jour
10.1002/etc.587	30	9	1965-72	jour
10.1016/j.envpol.2011.01.026	159	5	1290-6	jour
10.1016/j.chemosphere.2010.07.025	81	3	345-50	jour
10.1021/es902482b	44	2	760-6	jour
10.1021/es901672m	43	24	9453-7	jour
10.1016/j.envpol.2021.117476	285		117476	jour
10.1007/s11356-021-13101-7	28	26	902-34912	jour
10.1016/j.envpol.2020.116214	269		116214	jour
10.1016/j.marpolbul.2020.111733	161	Pt A	111733	jour
10.1007/s11356-020-10609-2	27	34	369-42389	jour
10.1016/j.envint.2020.105770	141		105770	jour
10.1016/j.chemosphere.2020.12648	249		126481	jour
10.1007/s11356-019-07239-8	27	6	5269-6277	jour
10.1016/j.jhazmat.2019.121346	384		121346	jour
10.1016/j.ecoenv.2019.05.066	180		762-769	jour
10.1021/acs.est.8b06214	53	7	3419-3428	jour
10.1016/j.envpol.2019.01.092	247		550-555	jour

10.1021/acs.est.8b05300	53	4	1786-1796	jour
10.1016/j.envpol.2018.10.094	244		667-674	jour
10.1016/j.envpol.2018.10.003	244		109-117	jour
10.1016/j.scitotenv.2018.10.055	651	Pt 2	1788-1795	jour
10.1016/j.chemosphere.2018.07.158	211		1193-1202	jour
10.1016/j.scitotenv.2018.03.135	631-632		1212-1220	jour
10.1016/j.chemosphere.2018.04.137	205		594-600	jour
10.1016/j.chemosphere.2018.02.041	199		603-611	jour
10.1016/j.scitotenv.2017.10.183	616-617		1339-1346	jour
10.1016/j.ecoenv.2017.09.047	147		803-808	jour
10.1016/j.envpol.2017.06.061	230		153-162	jour
10.1016/j.talanta.2017.04.066	171		68-73	jour
10.1016/j.aquatox.2017.04.009	188		26-32	jour
10.1007/s10653-017-9938-1	40	5	1887-1897	jour
10.1016/j.envpol.2017.03.011	224		7-15	jour
10.1016/j.scitotenv.2016.08.090	573		389-396	jour
10.1007/s00244-016-0296-2	71	3	359-64	jour
10.1016/j.chemosphere.2015.12.024	146		226-32	jour
10.1016/j.chemosphere.2015.11.008	150		514-519	jour
10.1016/j.chemosphere.2015.11.023	144		2476-81	jour
10.1021/acs.est.5b03181	49	22	13628-38	jour
10.1016/j.chemosphere.2015.04.024	134		166-71	jour
10.1016/j.scitotenv.2015.01.095	514		77-82	jour
10.1016/j.chemosphere.2014.12.066	135		462-6	jour
10.1016/j.scitotenv.2014.04.014	487		342-9	jour
10.1002/etc.2621	33	8	1732-8	jour
10.1016/j.jhazmat.2014.03.018	273		239-46	jour
10.1007/s11356-014-2861-4	22	19	14600-5	jour
10.1016/j.scitotenv.2014.01.082	481		114-20	jour
10.1016/j.scitotenv.2014.02.025	481		1-6	jour
10.1016/j.scitotenv.2014.01.105	479-480		48-56	jour
10.1016/j.envint.2014.01.010	66		11-7	jour
10.1016/j.aquatox.2014.01.003	148		83-91	jour
10.1016/j.ecoenv.2013.11.021	100		32-8	jour
10.1016/j.jhazmat.2013.10.075	264		230-5	jour
10.1021/es404106b	48	9	5187-93	jour
10.1016/j.ecoenv.2013.10.023	99		69-73	jour
10.1016/j.chemosphere.2013.08.054	93	8	1646-53	jour
10.1016/j.envint.2012.08.011	49		31-7	jour
10.1021/es201850n	45	16	6793-9	jour
10.1016/j.envint.2011.04.016	37	7	1273-84	jour
10.1016/j.chemosphere.2011.02.011	83	7	984-90	jour
10.1021/es103105x	44	24	9298-303	jour
10.1021/es800220y	42	15	5562-7	jour
10.1021/es072039a	42	1	31-6	jour
10.1021/es0710104	42	2	361-6	jour
10.1007/s00216-021-03286-8	413	13	3421-3431	jour

10.1002/etc.4923	40	2	413-421	jour
10.1016/j.envpol.2020.115096	265	Pt A	115096	jour
10.1016/j.ecoenv.2020.111124	204		111124	jour
10.1016/j.scitotenv.2020.140677	742		140677	jour
10.1210/endocr/bqaa096	161	8		jour
10.3390/ijerph17093064	17	9		jour
10.1021/acs.est.9b06748	54	11	5610-6620	jour
10.1080/01480545.2019.1701001			1-9	jour
10.1021/acs.est.8b06201	53	3	1325-1333	jour
10.1016/j.envres.2018.06.037	166		553-561	jour
10.1038/s41366-018-0072-7	43	3	545-555	jour
10.1016/j.chemosphere.2018.03.182	203		291-299	jour
10.1007/s00216-017-0784-x	410	4	1375-1387	jour
10.1016/j.ecoenv.2017.10.069	149		51-57	jour
10.1016/j.envpol.2017.05.024	228		61-71	jour
10.1016/j.marpolbul.2017.01.049	117	1-2	456-461	jour
10.1016/j.envpol.2016.10.040	219		191-200	jour
10.1016/j.envint.2016.08.013	96		65-74	jour
10.1007/s11356-016-6216-1	23	11	702-10713	jour
10.1021/acs.est.5b05814	50	5	2700-8	jour
10.1016/j.talanta.2015.07.031	144		1014-20	jour
10.1021/acs.est.5b01996	49	14	8623-30	jour
10.1016/j.envpol.2014.10.007	196		284-91	jour
10.1016/j.envint.2014.10.003	74		54-9	jour
10.1007/s00244-014-0074-y	68	1	83-91	jour
10.1007/s11356-014-2823-x	21	14	8870-7	jour
10.1016/j.envint.2013.08.009	60		81-8	jour
10.1021/es401144c	47	18	10567-73	jour
10.1007/s10653-013-9545-8	35	5	625-31	jour
	34	3	1129-35	jour
10.1016/j.envint.2013.02.008	55		101-8	jour
10.1016/j.envpol.2013.02.012	177		150-5	jour
10.1002/etc.2202	32	7	1649-54	jour
10.1002/etc.2199	32	6	1376-81	jour
10.1016/j.envpol.2013.01.015	176		80-6	jour
10.1016/j.scitotenv.2012.12.059	445-446		329-36	jour
10.1016/j.envpol.2012.11.007	174		57-62	jour
10.1016/j.chemosphere.2012.10.106	90	7	2149-56	jour
10.1021/es302934p	46	22	12364-72	jour
10.1021/es301804t	46	19	10758-64	jour
10.1016/j.chemosphere.2012.05.072	89	4	416-9	jour
10.1021/es300138q	46	6	3141-8	jour
10.1021/es202762n	45	23	9924-30	jour
10.3724/sp.j.1123.2011.00543	29	6	543-8	jour
10.1016/j.envint.2011.06.005	37	8	1357-61	jour
10.1021/es2003028	45	12	5088-98	jour
10.1016/j.envpol.2011.04.024	159	10	2982-8	jour

10.1016/j.cbpc.2011.04.005	154	2	129-34	jour
10.1016/j.envint.2011.03.025	37	7	1164-8	jour
10.1002/rcm.4874	25	3	436-42	jour
10.1021/es102251s	45	2	400-5	jour
10.1016/j.chemosphere.2010.10.097	82	5	692-7	jour
10.1021/es103047n	44	23	8977-82	jour
10.1177/1091581810384154	29	6	582-93	jour
10.1016/j.envint.2010.07.002	37	1	66-70	jour
10.1016/j.envpol.2010.06.003	158	9	2920-5	jour
10.1016/j.chemosphere.2010.06.05[illegible]	80	11	1285-90	jour
10.1016/j.chemosphere.2010.02.05[illegible]	79	8	850-4	jour
10.1021/es9027106	44	7	2305-8	jour
10.1021/es9025535	44	2	574-9	jour
10.1021/es902744b	44	2	606-11	jour
10.1016/j.chemosphere.2008.11.03[illegible]	75	1	115-20	jour
10.1021/es800479c	42	17	6476-80	jour
10.1021/es071716y	41	22	7694-8	jour
10.1021/es051911h	40	4	1184-9	jour
10.1016/j.chemosphere.2021.13062	279		130620	jour
10.1016/j.envpol.2021.117342	287		117342	jour
10.1016/j.scitotenv.2020.141348	746		141348	jour
10.1016/j.scitotenv.2020.137480	720		137480	jour
10.1016/j.scitotenv.2019.136335	710		136335	jour
10.1016/j.envres.2019.109082	182		109082	jour
10.1016/j.envint.2019.105416	135		105416	jour
10.1016/j.envint.2019.104952	130		104952	jour
10.1016/j.chemosphere.2019.06.11[illegible]	234		365-372	jour
10.1016/j.chemosphere.2019.04.07[illegible]	227		315-322	jour
10.1016/j.envpol.2019.01.103	248		646-658	jour
10.1007/s11356-019-04558-8	26	12	171-12180	jour
10.1016/j.ecoenv.2018.10.089	168		304-314	jour
10.1016/j.jhazmat.2018.07.109	359		491-499	jour
10.1016/j.envpol.2018.06.038	241		1063-1070	jour
10.1016/j.scitotenv.2018.05.268	642		537-542	jour
10.1016/j.chemosphere.2018.05.03[illegible]	208		233-240	jour
10.1016/j.scitotenv.2017.09.112	615		1019-1027	jour
10.1039/c7em00549k	20	3	469-479	jour
10.1016/j.jes.2017.09.005	62		115-132	jour
10.1039/c7em00495h	20	1	98-104	jour
10.1016/j.chemosphere.2017.11.17[illegible]	194		256-265	jour
10.1021/acs.est.7b04866	52	2	474-483	jour
10.1016/j.envpol.2017.10.104	233		642-654	jour
10.1080/15287394.2017.1336414	80	9	525-531	jour
10.1016/j.chemosphere.2017.05.11[illegible]	183		257-265	jour
10.1016/j.scitotenv.2017.04.246	599-600		1903-1911	jour
10.1016/j.envres.2017.03.058	156		494-504	jour
10.1016/j.marpolbul.2017.03.009	118	1-2	413-419	jour

10.1016/j.ecoenv.2017.02.041	140		109-115	jour
10.1007/s10661-017-5808-7	189	2	92	jour
10.1021/acs.est.6b04646	50	24	232-13238	jour
10.1007/s11356-016-8082-2	24	4	3882-3889	jour
10.1016/j.chemosphere.2016.10.01	168		272-278	jour
10.1016/j.chemosphere.2016.09.14	167		291-299	jour
10.1016/j.envpol.2016.09.023	220	Pt A	63-71	jour
10.1016/j.scitotenv.2016.07.080	573		1380-1389	jour
10.1016/j.envpol.2016.06.030	216		662-674	jour
10.1016/j.envpol.2016.04.071	214		705-712	jour
10.1007/s11356-015-5956-7	23	8	7998-8007	jour
10.1016/j.envres.2015.12.004	146		73-84	jour
10.1016/j.scitotenv.2015.09.001	539		286-293	jour
10.1016/j.marpolbul.2015.07.041	99	1-2	150-6	jour
10.1016/j.chemosphere.2015.06.01	138		316-23	jour
10.1016/j.envpol.2015.02.035	201		131-40	jour
10.1021/es5044547	48	24	14426-34	jour
10.1016/j.chemosphere.2014.09.01	119		953-960	jour
10.1021/es503510q	48	21	12586-94	jour
10.1021/es501224b	48	15	8839-46	jour
10.1016/j.chemosphere.2013.09.09	95		442-7	jour
10.1016/j.envpol.2012.08.012	172		94-9	jour
10.1021/es302099f	46	17	9735-44	jour
10.1016/j.envint.2011.09.010	39	1	50-5	jour
10.1093/annhyg/mer033	55	6	658-65	jour
10.1021/es702272s	42	2	386-91	jour
10.1021/es070810b	41	17	6014-9	jour
10.1021/es062781v	41	7	2249-54	jour
10.1021/acs.est.0c07367	55	14	9450-9459	jour
10.1016/j.marpolbul.2021.112099	164		112099	jour
10.1016/j.chemosphere.2020.12931	267		129316	jour
10.1016/j.envpol.2020.116157	270		116157	jour
10.1016/j.envres.2020.110344	196		110344	jour
10.1016/j.envpol.2020.115727	268	Pt A	115727	jour
10.1016/j.scitotenv.2020.140874	743		140874	jour
10.1016/j.envint.2020.105741	139		105741	jour
10.1016/j.envres.2020.109462	185		109462	jour
10.1016/j.envpol.2020.114400	263	Pt A	114400	jour
10.1016/j.envpol.2020.114306	262		114306	jour
10.1016/j.scitotenv.2020.137386	719		137386	jour
10.1016/j.envpol.2020.114087	260		114087	jour
10.1016/j.ecoenv.2020.110212	191		110212	jour
10.1016/j.scitotenv.2019.136119	707		136119	jour
10.1021/acs.est.9b04892	54	1	325-334	jour
10.1016/j.ecoenv.2019.109758	186		109758	jour
10.1016/j.envres.2019.108678	178		108678	jour
10.1016/j.envpol.2019.07.070	253		850-863	jour

10.1002/etc.4413	38	6	1198-1210	jour
10.1021/acs.est.8b05503	53	5	2820-2829	jour
10.1016/j.scitotenv.2018.12.268	658		1355-1366	jour
10.1016/j.envres.2019.01.028	171		145-152	jour
10.1016/j.aca.2018.10.011	1047		71-80	jour
10.1186/s12302-018-0172-y	30	1	43	jour
10.1016/j.scitotenv.2018.11.369	656		1-8	jour
10.1016/j.scitotenv.2018.10.096	652		212-223	jour
10.1016/j.scitotenv.2018.08.408	649		1541-1552	jour
10.1016/j.scitotenv.2018.07.373	646		1090-1096	jour
10.2116/analsci.18P221	34	12	1365-1371	jour
10.1016/j.scitotenv.2018.07.333	647		525-533	jour
10.1016/j.jhazmat.2018.06.070	358		366-375	jour
10.1016/j.chemosphere.2018.07.007	210		147-155	jour
10.1016/j.envres.2017.12.014	162		166-172	jour
10.1021/acs.est.7b03159	51	23	395-13905	jour
10.1016/j.scitotenv.2017.10.296	616-617		38-45	jour
10.1016/j.scitotenv.2017.10.091	621		1370-1377	jour
10.1016/j.scitotenv.2017.07.112	607-608		1109-1116	jour
10.1016/j.scitotenv.2017.06.045	601-602		1619-1627	jour
10.1007/s00216-017-0432-5	409	20	4905-4913	jour
10.1016/j.scitotenv.2017.04.123	598		385-392	jour
10.1021/acs.est.7b00429	51	9	4860-4869	jour
10.1016/j.scitotenv.2017.03.217	595		303-314	jour
10.1016/j.envpol.2017.02.052	224		679-688	jour
10.1016/j.envres.2016.09.024	152		26-42	jour
10.1021/acs.est.6b01685	50	18	9846-54	jour
10.1016/j.chemosphere.2016.07.100	162		308-14	jour
10.1016/j.scitotenv.2016.06.053	569-570		982-989	jour
10.13227/j.hjkx.2016.07.016	37	7	2539-2546	jour
10.1016/j.envpol.2016.05.039	216		64-70	jour
10.1016/j.scitotenv.2016.04.173	565		346-359	jour
10.1016/j.envint.2016.04.025	92-93		442-9	jour
10.1016/j.envres.2016.04.005	148		285-294	jour
10.1039/c5em00593k	18	3	361-70	jour
10.1016/j.envres.2016.01.021	146		371-8	jour
10.1016/j.chemosphere.2016.01.034	148		195-203	jour
10.1016/j.envpol.2015.10.038	208	Pt B	619-25	jour
10.1016/j.scitotenv.2015.10.088	542	Pt A	411-26	jour
10.1016/j.envres.2015.08.018	142		720-30	jour
10.1016/j.chemosphere.2015.07.044	139		565-71	jour
10.1016/j.talanta.2015.04.084	143		7-18	jour
10.1021/acs.accounts.5b00180	48	7	1853-61	jour
10.1016/j.chemosphere.2015.04.029	137		25-32	jour
10.1016/j.scitotenv.2015.04.012	523		219-32	jour
10.1016/j.marpolbul.2015.02.014	93	1-2	61-7	jour
10.1002/etc.2956	34	6	1296-308	jour

10.1016/j.envint.2015.02.006	78		1-7 jour
10.1021/es505876k	49	5	2904-11 jour
10.1021/es505394y	49	23	13743-8 jour
10.1016/j.scitotenv.2014.09.080	505		47-55 jour
10.1007/s11356-014-3404-8	22	3	1635-43 jour
10.1021/es502743q	48	16	9812-8 jour
10.1016/j.chemosphere.2014.04.019	111		379-95 jour
10.1021/es501973d	48	15	8815-22 jour
10.1021/es501139d	48	13	7584-92 jour
10.1002/etc.2588	33	8	1712-9 jour
10.1007/s00244-014-0040-8	67	3	348-57 jour
10.1021/es500911d	48	11	6133-40 jour
10.1021/es5011708	48	12	6957-64 jour
10.1016/j.scitotenv.2014.03.008	482-483		276-82 jour
10.1016/j.envpol.2014.01.023	188		118-23 jour
10.1016/j.envint.2014.01.023	66		65-70 jour
10.1021/es403600a	48	3	1458-66 jour
10.1039/c3em00590a	16	2	298-305 jour
10.1021/es403383e	47	21	12238-47 jour
10.1016/j.chemosphere.2013.06.023	93	3	506-11 jour
10.1016/j.chroma.2013.05.064	1300		85-94 jour
10.1021/es400027v	47	7	3344-52 jour
10.1002/etc.2172	32	6	1242-7 jour
10.1016/j.envpol.2012.11.024	174		164-70 jour
10.1021/es303393z	47	1	349-54 jour
10.1021/es303879n	46	24	13056-66 jour
10.1039/c2em30472d	14	11	2870-6 jour
10.1016/j.envint.2012.06.005	47		56-65 jour
10.1016/j.envint.2012.04.006	45		122-8 jour
10.1016/j.envpol.2012.03.040	168		1-9 jour
10.1016/j.envint.2011.11.008	40		1-7 jour
	32	10	2891-5 jour
10.1016/j.envpol.2011.09.041	161		154-61 jour
10.1021/es200112m	45	11	4696-701 jour
10.1021/es1043529	45	10	4602-8 jour
10.1002/etc.122	29	4	852-9 jour
10.1021/es101599h	44	16	6196-201 jour
10.1016/j.chemosphere.2010.06.013	80	10	1207-12 jour
10.1016/j.chemosphere.2010.05.043	80	10	1234-40 jour
10.1021/es9039264	44	12	4658-64 jour
10.1021/es903688s	44	2	546-7 jour
10.1093/chromsci/47.1.83	47	1	83-91 jour
10.1897/08-162.1	28	5	910-20 jour
10.1021/es800313z	42	13	4745-51 jour
	42	1	5-6 jour
10.1021/es0630487	41	13	4561-7 jour

Log / Batch

Date	Record Ct
7/21/2021 15:30	364
7/21/2021 15:23	2795
7/21/2021 15:19	287
7/21/2021 15:11	105
7/21/2021 15:11	105
7/21/2021 14:56	1
7/21/2021 14:23	42
7/20/2021 18:42	76

Query Used (double-click on query to rerun)

(Dechlorane Plus)

oceans AND coral

(Dechlorane Plus) AND (flame retardants)

(Pentabromodiphenyl ether) AND (environmental exposure AND (water OR groundwater OR drinking water))

(Pentabromodiphenyl ether) AND (environmental exposure AND (water OR groundwater OR drinking water))

22982223[uid]

23185960[uid] OR 23724807[uid] OR 24016281[uid] OR 24493336[uid] OR 24656972[uid] OR 24846325[uid] OR 2

(3194-55-6 OR 1,2,5,6,9,10-Hexabromocyclododecane OR hexabromocyclododecane) AND (environmental exposu

Note: Feel free to delete rows, but not columns. Some queries are displayed here as samples.

Batch Tag

25240235[uid] OR 25493574[uid] OR 25929991[uid] OR 25933174[uid] OR 26099790[uid] OR 26134984[uid] OR 26[illegible]

[illegible]ure AND dust)

363216[uid] OR 26613357[uid] OR 26725304[uid] OR 26742016[uid] OR 27144674[uid] OR 27309668[uid] OR

27623734[uid] OR 27741446[uid] OR 27934115[uid] OR 28034506[uid] OR 28338271[uid] OR 28556503[uid] [illegible]

OR 28869101[uid] OR 28910084[uid] OR 29320633[uid] OR 29734094[uid] OR 30196219[uid] OR 30223335[ui

id] OR 30448504[uid] OR 31081616[uid] OR 31276863[uid] OR 31603658[uid] OR 32100996[uid] OR 32417508

3[uid] OR 32460157[uid] OR 32854002[uid] OR 33026620[uid] OR 33466958[uid] OR 33736204[uid] OR 339694

458[uid]

Abstract Sifter Landscape View

Version 6.0 (beta)

(optional) DSSTOX link to Dashboard	Preferred Name
DTXSID001014486	Calcium molybdate(VI)
DTXSID0024553	Azodicarbonamide
DTXSID0026252	Triphenyl phosphite
DTXSID6021959	2,4,6-Tribromophenol
DTXSID2024246	Pentabromodiphenyl ether
DTXSID2052732	1,1'-Ethane-1,2-diylbis(pentabromobenze
DTXSID4052710	2,4,4'-Tribromodiphenyl ether
DTXSID4052712	Tripropyl phosphate
DTXSID7027750	Dechlorane Plus

Subject queries:	environmental exposure AND dust	environmental exposure AND food	environmental exposure AND (water OR groundwa
Summary heading	Exposure	Exposure	Exposure
**Chemical / Entity query**	**Dust exposure**	**Food exposure**	**Water exposure**
7789-82-4 OR Calcium molybdate(VI)	0	0	0
123-77-3 OR Azodicarbonamide OR 1,1-azobisformamide	1	1	0
101-02-0 OR Triphenyl phosphite	2	0	0
2,4,6-Tribromophenol	6	17	22
Pentabromodiphenyl ether	44	93	105
1,1'-Ethane-1,2-diylbis(pentabromobenzene)	0	0	0
41318-75-6 OR 2,4,4'-Tribromodiphenyl ether OR tribromodiphenyl ether 28		10	12
513-08-6 OR Tripropyl phosphate	1	0	6
Dechlorane Plus	31	69	120

(Occupati onal exposure OR Air Pollutants , Exposure	Chemistr y Techniqu es, Analytical " OR Methods	toxicity	flame retardant s
**Occupati onal**	**Analytical chemistry**	**toxicity**	**flame retardant s**
0	0	2	0
14	3	20	1
2	1	31	1
14	19	41	66
47	23	235	279
0	0	0	0
6	2	8	24
0	0	2	17
18	24	34	364

Term Expand / Map

Version 6.0 (beta)

Map to this:	When you see this:	Count
Include	control	
Include	dose	
Include	mg	
Include	OECD	
Include	EPA	
Include	NTP	
Include	guideline	
Include	mice	
Include	rats	
Exclude	worker	
Exclude	occupation	
Exclude	patient	
Exclude	children	
Exclude	women	
Exclude	men	
Exclude	human	
Exclude	person	

Double-click on cells for quick sifting. Or go to Main and sift on TermX: for any mapping or TX: for counts of each mapping.

My Notes

Double-click on row to curate

PMID	yes	no	maybe	Who	Tag	Note	PubYr
23185960	1	0	0		**Bergman search forward publications**		2012
23724807	1	0	0		**Bergman search forward publications**		2014
24016281	1	0	0		**Bergman search forward publications**		2013
24493336	1	0	0		**Bergman search forward publications**		2014
24656972	1	0	0		**Bergman search forward publications**		2014
24846325	1	0	0		**Bergman search forward publications**		2014
25240235	1	0	0		**Bergman search forward publications**		2014
25493574	1	0	0		**Bergman search forward publications**		2015
25929991	1	0	0		**Bergman search forward publications**		2016
25933174	1	0	0		**Bergman search forward publications**		2015
26099790	1	0	0		**Bergman search forward publications**		2015
26134984	1	0	0		**Bergman search forward publications**		2015
26363216	1	0	0		**Bergman search forward publications**		2015
26613357	1	0	0		**Bergman search forward publications**		2016
26725304	1	0	0		**Bergman search forward publications**		2016
26742016	1	0	0		**Bergman search forward publications**		2016
27144674	1	0	0		**Bergman search forward publications**		2016
27309668	1	0	0		**Bergman search forward publications**		2016
27623734	1	0	0		**Bergman search forward publications**		2016
27741446	1	0	0		**Bergman search forward publications**		2017
27934115	1	0	0		**Bergman search forward publications**		2016
28034506	1	0	0		**Bergman search forward publications**		2017
28338271	1	0	0		**Bergman search forward publications**		2015
28556503	1	0	0		**Bergman search forward publications**		2017
28869101	1	0	0		**Bergman search forward publications**		2017
28910084	1	0	0		**Bergman search forward publications**		2017
29320633	1	0	0		**Bergman search forward publications**		2018
29734094	1	0	0		**Bergman search forward publications**		2018
30196219	1	0	0		**Bergman search forward publications**		2019
30223335	1	0	0		**Bergman search forward publications**		2018
30448504	1	0	0		**Bergman search forward publications**		2019
31081616	1	0	0		**Bergman search forward publications**		2019
31276863	1	0	0		**Bergman search forward publications**		2019
31603658	1	0	0		**Bergman search forward publications**		2019
32100996	1	0	0		**Bergman search forward publications**		2020

32417508	1	0	0	**Bergman search forward publications**	2020
32460157	1	0	0	**Bergman search forward publications**	2020
32854002	1	0	0	**Bergman search forward publications**	2021
33026620	1	0	0	**Bergman search forward publications**	2020
33466958	1	0	0	**Bergman search forward publications**	2021
33736204	1	0	0	**Bergman search forward publications**	2021
33969458	1	0	0	**Bergman search forward publications**	2021
22982223	1	0	0	**Bergman**	2012

Title
After the PBDE phase-out: a broad suite of flame retardants in repeat house dust
Phosphorus flame retardants in indoor dust and their relation to asthma and aller
Novel analytical methods for flame retardants and plasticizers based on gas chron
Direct probe atmospheric pressure photoionization/atmospheric pressure chemic
Assessing the persistence, bioaccumulation potential and toxicity of brominated f
Occurrence of a broad range of legacy and emerging flame retardants in indoor er
Ecotoxicity and biodegradability of new brominated flame retardants: a review.
Feasibility study of feces for noninvasive biomonitoring of brominated flame retar
Organophosphate and phthalate esters in settled dust from apartment buildings i
Physical-chemical properties and evaluative fate modelling of 'emerging' and 'nov
Organophosphorus flame retardants (PFRs) and plasticisers in harbour porpoises (
Interlaboratory study of novel halogenated flame retardants: INTERFLAB.
Neurotoxicity and risk assessment of brominated and alternative flame retardants
Novel flame retardants: Estimating the physical-chemical properties and environn
Brominated flame retardants (BFRs): A review on environmental contamination ir
Are some "safer alternatives" hazardous as PBTs? The case study of new flame ret
Distribution of Organophosphate Esters between the Gas and Particle Phase-Mod
Organophosphate Esters in Canadian Arctic Air: Occurrence, Levels and Trends.
Consumer Product Chemicals in Indoor Dust: A Quantitative Meta-analysis of U.S.
Legacy and emerging flame retardants (FRs) in the freshwater ecosystem: A reviev
Determination of Halogenated Flame Retardants Using Gas Chromatography with
Development and comparison of gas chromatography-mass spectrometry technic
Identification strategies for flame retardants employing time-of-flight mass spectr
Occurrence and human exposure assessment of organophosphate flame retardan
Recycling of plastic waste: Screening for brominated flame retardants (BFRs).
Changes in Flame Retardant and Legacy Contaminant Concentrations in Indoor Ai
Bioaccumulation of Polybrominated Diphenyl Ethers and Alternative Halogenated
Small-scale spatial variability of flame retardants in indoor dust and implications f
Organophosphate esters in house dust: A comparative study between Canada, Tu
New brominated flame retardants and dechlorane plus in the Arctic: Local sources
Distributions of organophosphate flame retardants (OPFRs) in three dust size frac
Combining in Silico Tools with Multicriteria Analysis for Alternatives Assessment o
Assessment of Dechlorane Plus and related compounds in foodstuffs and estimate
Emissions and Occupational Exposure Risk of Halogenated Flame Retardants from
Functional Group-Dependent Screening of Organophosphate Esters (OPEs) and Di

Novel brominated flame retardants - A review of their occurrence in indoor air, du
The influence of an upgrade on the reduction of organophosphate flame retardan
Organophosphate ester flame retardants have antiandrogenic potential and affec
A comprehensive evaluation of two sample treatment procedures for the determi
Dechlorane Plus and Related Compounds in Food-A Review.
Temporal trends of legacy and novel brominated flame retardants in sediments al
Neurodevelopmental toxicity assessment of flame retardants using a human DNT
A novel abbreviation standard for organobromine, organochlorine and organopho

Note: Feel free to delete rows after Row 2, but not columns. Some sample Notes may be displayed.

Authors	Journal	Other Note
Dodson RE, Perovich LJ, Covaci A, Van den Eede	Environmental science & technology	
Araki A, Saito I, Kanazawa A, Morimoto K, Nakay	Indoor air	
Ballesteros-Gómez A, de Boer J, Leonards PE	Analytical chemistry	
Ballesteros-Gómez A, Brandsma SH, de Boer J, L	Analytical and bioanalytical chemistry	
Stieger G, Scheringer M, Ng CA, Hungerbühler K	Chemosphere	
Cequier E, Ionas AC, Covaci A, Marcé RM, Beche	Environmental science & technology	
Ezechiáš M, Covino S, Cajthaml T	Ecotoxicology and environmental safety	
Sahlström LM, Sellström U, de Wit CA, Lignell S,	Environmental science & technology	
Luongo G, Östman C	Indoor air	
Liagkouridis I, Cousins AP, Cousins IT	The Science of the total environment	
Papachlimitzou A, Barber JL, Losada S, Bersuder	Marine pollution bulletin	
Melymuk L, Goosey E, Riddell N, Diamond ML	Analytical and bioanalytical chemistry	
Hendriks HS, Westerink RH	Neurotoxicology and teratology	
Zhang X, Sühring R, Serodio D, Bonnell M, Sundi	Chemosphere	
Yu G, Bu Q, Cao Z, Du X, Xia J, Wu M, Huang J	Chemosphere	
Gramatica P, Cassani S, Sangion A	Journal of hazardous materials	
Sühring R, Wolschke H, Diamond ML, Jantunen L	Environmental science & technology	
Sühring R, Diamond ML, Scheringer M, Wong F,	Environmental science & technology	
Mitro SD, Dodson RE, Singla V, Adamkiewicz G, E	Environmental science & technology	
Iqbal M, Syed JH, Katsoyiannis A, Malik RN, Faro	Environmental research	
Megson D, Robson M, Jobst KJ, Helm PA, Reiner	Analytical chemistry	
Gustavsson J, Ahrens L, Nguyen MA, Josefsson S	Journal of chromatography. A	
Ionas AC, Ballesteros Gómez A, Leonards PE, Co	Journal of mass spectrometry : JMS	
Zhou L, Hiltscher M, Püttmann W	Indoor air	
Pivnenko K, Granby K, Eriksson E, Astrup TF	Waste management (New York, N.Y.)	
Vojta Š, Melymuk L, Klánová J	Environmental science & technology	
Morris AD, Muir DCG, Solomon KR, Teixeira CF, I	Environmental science & technology	
Jílková S, Melymuk L, Vojta Š, Vykoukalová M, B	Chemosphere	
Shoeib T, Webster GM, Hassan Y, Tepe S, Yalcin	The Science of the total environment	
Carlsson P, Vrana B, Sobotka J, Borgå K, Bohlin N	Chemosphere	
Zhou L, Püttmann W	Environmental pollution (Barking, Essex : 1987)	
Zheng Z, Peters GM, Arp HPH, Andersson PL	Environmental science & technology	
Abdel Malak I, Cariou R, Guiffard I, Vénisseau A,	Chemosphere	
Li TY, Ge JL, Pei J, Bao LJ, Wu CC, Zeng EY	Environmental science & technology	
Meng W, Li J, Shen J, Deng Y, Letcher RJ, Su G	Environmental science & technology	

Zuiderveen EAR, Slootweg JC, de Boer J	Chemosphere
Zhang D, Li S, Zhu F, Li C, Xu Y, Qing D, Wang J	Chemosphere
Rosenmai AK, Winge SB, Möller M, Lundqvist J, \	Chemosphere
Tolosa I, Huertas D, Choyke S, Sander S, Aminot	Environmental science and pollution research i
Ghelli E, Cariou R, Dervilly G, Pagliuca G, Gazzott	International journal of environmental researcl
Vauclin S, Mourier B, Dendievel AM, Marchand I	Chemosphere
Klose J, Pahl M, Bartmann K, Bendt F, Blum J, Dc	Cell biology and toxicology
Bergman A, Rydén A, Law RJ, de Boer J, Covaci A	Environment international

When Noted	DOI	Volume	Issue	Pages	PubType	pmc	pdflink	BatchTag
7/21/2021 14:22	10.1021/e	46	24	13056-66	jour	1		
7/21/2021 14:22	10.1111/ir	24	1	3-15	jour	0		
7/21/2021 14:22	10.1021/a	85	20	9572-80	jour	0		
7/21/2021 14:22	10.1007/s(	406	11	2503-12	jour	0		
7/21/2021 14:22	10.1016/j.	116		118-23	jour	0		
7/21/2021 14:22	10.1021/e	48	12	6827-35	jour	0		
7/21/2021 14:22	10.1016/j.	110		153-67	jour	0		
7/21/2021 14:22	10.1021/e	49	1	606-15	jour	0		
7/21/2021 14:22	10.1111/ir	26	3	414-25	jour	0		
7/21/2021 14:22	10.1016/j.	524-525		416-26	jour	0		
7/21/2021 14:22	10.1016/j.	98	1-2	328-34	jour	0		
7/21/2021 14:22	10.1007/s(	407	22	6759-69	jour	0		
7/21/2021 14:22	10.1016/j.	52	Pt B	248-69	jour	0		
7/21/2021 14:22	10.1016/j.	144		2401-7	jour	0		
7/21/2021 14:22	10.1016/j.	150		479-490	jour	0		
7/21/2021 14:22	10.1016/j.	306		237-246	jour	0		
7/21/2021 14:22	10.1021/a	50	13	6644-51	jour	0		
7/21/2021 14:22	10.1021/a	50	14	7409-15	jour	0		
7/21/2021 14:22	10.1021/a	50	19	561-10672	jour	1		
7/21/2021 14:22	10.1016/j.	152		26-42	jour	0		
7/21/2021 14:22	10.1021/a	88	23	406-11411	jour	0		
7/21/2021 14:22	10.1016/j.	1481		116-126	jour	0		
7/21/2021 14:22	10.1002/jr	50	8	1031-1038	jour	0		
7/21/2021 14:22	10.1111/ir	27	6	1113-1127	jour	0		
7/21/2021 14:22	10.1016/j.	69		101-109	jour	0		
7/21/2021 14:22	10.1021/a	51	20	891-11899	jour	0		
7/21/2021 14:22	10.1021/a	52	5	3136-3145	jour	0		
7/21/2021 14:22	10.1016/j.	206		132-141	jour	0		
7/21/2021 14:22	10.1016/j.	650	Pt 1	193-201	jour	0		
7/21/2021 14:22	10.1016/j.	211		1193-1202	jour	0		
7/21/2021 14:22	10.1016/j.	245		343-352	jour	0		
7/21/2021 14:22	10.1021/a	53	11	6341-6351	jour	0		
7/21/2021 14:22	10.1016/j.	235		492-497	jour	0		
7/21/2021 14:22	10.1021/a	53	21	495-12505	jour	0		
7/21/2021 14:22	10.1021/a	54	7	4455-4464	jour	0		

7/21/2021 14:22	10.1016/j.	255		126816	jour	0
7/21/2021 14:22	10.1016/j.	256		126895	jour	0
7/21/2021 14:22	10.1016/j.	263		127703	jour	0
7/21/2021 14:22	10.1007/s:				jour	0
7/21/2021 14:22	10.3390/ij	18	2		jour	1
7/21/2021 14:22	10.1016/j.	271		129889	jour	0
7/21/2021 14:22	10.1007/s:				jour	0
7/21/2021 14:56	10.1016/j.	49		57-82	jour	1

Abstract wit

PMID:

Title:

Title and Abstract:

33001521

Cryptic lineages respond differently to coral bleaching.

Cryptic lineages respond differently to coral bleaching. ABSTRACT: Coral cover is decreasing worldwide largely as a result of a rise in sea**water** temperatures that triggers coral bleaching and induces coral mortality. How coral reefs will respond to climate change will be a function of genetic variation and how it is partitioned within and among species. A critical initial step is to accurately delineate species and quantify their physiological potential to cope with heat stress. Cryptic species are morphologically similar but genetically distinct and may respond physiologically differently to climate change. A dominant Caribbean reef builder severely affected by climate change is the **mountainous star** coral, **Orbicella faveolata**. Recently in this journal, Dziedzic et al. reported quantitative genetic variation in the physiological response to thermal stress in a single population of this species, suggesting that variation within populations will allow these corals to adapt to rising ocean temperatures. We reanalysed their data and found multiple cryptic lineages rather than a single panmictic population, with one of the lineages being heat-intolerant. While different cryptic lineages co-occur in certain locations, there is at least one lineage that occurs only in a single location. Our finding of hidden lineages within a threatened species highlights the varying extinction risks faced by these independently evolving groups, especially when the prospects of survival under warmer oceans seem favourable for only some of them.© 2020 John Wiley & Sons Ltd. KEYWORDS: MeSH: | Animals| **Anthozoa**/majr/genetics| Caribbean Region| Coral Reefs| Genomics| Oceans and Seas| Temperature Other:coral bleaching|cryptic species|genomics|global warming TERMX: |TX:Coral:**Anthozoa** |**TX:Coral:orange:spikey:Mountainous Star** |**TX:Coral:orange:spikey**:**Orbicella faveolata**

4	PubYr	Authors	Journal	Volume
	2020	Gómez-Corrales M, Prada C	Molecular ecology	29

Issue	Pages	DOI	PMC
22	65-4273	10.1111/mec	0

`

Curated FR Universe

DSSToxID
DTXSID0027147
DTXSID0029189
DTXSID0041226
DTXSID1021374
DTXSID2027044
DTXSID2062325
DTXSID3043811
DTXSID3051543
DTXSID30872414
DTXSID40174330
DTXSID4021426
DTXSID4023888
DTXSID4074932
DTXSID40889331
DTXSID5024267
DTXSID5025940
DTXSID5026415
DTXSID50931491
DTXSID60174332
DTXSID6023028
DTXSID6030732
DTXSID6052823
DTXSID60894063
DTXSID7049631
DTXSID7052737
DTXSID801017084
DTXSID8021432
DTXSID8031865
DTXSID8034902
DTXSID8052641
DTXSID8052699
DTXSID8058821
DTXSID8065660
DTXSID80988973
DTXSID9021392
DTXSID9021847
DTXSID9027233
DTXSID9029647
DTXSID9041861
DTXSID9050484
DTXSID9060449
DTXSID001002372
DTXSID001010941

DTXSID001014486
DTXSID001016668
DTXSID001016682
DTXSID001016694
DTXSID001016701
DTXSID001016751
DTXSID001017111
DTXSID001018214
DTXSID00103429
DTXSID00108393
DTXSID00164338
DTXSID00181969
DTXSID00196255
DTXSID00199847
DTXSID0020078
DTXSID0020494
DTXSID00207815
DTXSID0021173
DTXSID0021331
DTXSID0021414
DTXSID00214802
DTXSID00219337
DTXSID0024553
DTXSID0026252
DTXSID0026258
DTXSID0028903
DTXSID0029557
DTXSID00477016
DTXSID0049814
DTXSID0050471
DTXSID0052679
DTXSID0052700
DTXSID0052702
DTXSID0052708
DTXSID0052750
DTXSID0058300
DTXSID00583560
DTXSID0058691
DTXSID0058695
DTXSID0060561
DTXSID0066406
DTXSID0075108
DTXSID00872668
DTXSID00872789
DTXSID00872800
DTXSID00873771
DTXSID00879854

DTXSID00879859
DTXSID00879874
DTXSID00879879
DTXSID00879894
DTXSID00879899
DTXSID00879910
DTXSID00879915
DTXSID00879930
DTXSID00879935
DTXSID00879950
DTXSID00879955
DTXSID00879970
DTXSID00879975
DTXSID00889812
DTXSID00893979
DTXSID00894087
DTXSID00904130
DTXSID00955980
DTXSID00956119
DTXSID00957040
DTXSID00959387
DTXSID00971221
DTXSID101000529
DTXSID101014603
DTXSID101016697
DTXSID101016704
DTXSID101017114
DTXSID101018500
DTXSID10107312
DTXSID1020142
DTXSID10201783
DTXSID1020194
DTXSID10208772
DTXSID1021403
DTXSID10218114
DTXSID1021952
DTXSID10229705
DTXSID1024128
DTXSID1024382
DTXSID1024627
DTXSID1025017
DTXSID1025300
DTXSID1026081
DTXSID1026083
DTXSID1027134
DTXSID10274134
DTXSID1029049

DTXSID1029095
DTXSID1029677
DTXSID1029704
DTXSID1033325
DTXSID1034347
DTXSID1034391
DTXSID1044699
DTXSID1048207
DTXSID1049697
DTXSID1051802
DTXSID1052290
DTXSID1052531
DTXSID10577713
DTXSID1059072
DTXSID1060558
DTXSID1061946
DTXSID1062623
DTXSID1064746
DTXSID1068720
DTXSID1068859
DTXSID1069455
DTXSID10704805
DTXSID1073157
DTXSID1074252
DTXSID1074597
DTXSID10850114
DTXSID10855048
DTXSID10865889
DTXSID10872316
DTXSID10872553
DTXSID10872775
DTXSID10872795
DTXSID10873929
DTXSID10879860
DTXSID10879865
DTXSID10879880
DTXSID10879885
DTXSID10879901
DTXSID10879906
DTXSID10879921
DTXSID10879926
DTXSID10879941
DTXSID10879946
DTXSID10879961
DTXSID10879966
DTXSID10879981
DTXSID10886791

DTXSID10892231
DTXSID10893960
DTXSID1091555
DTXSID10923559
DTXSID10987252
DTXSID201009900
DTXSID201016652
DTXSID201016676
DTXSID201016688
DTXSID201016690
DTXSID201016707
DTXSID201016864
DTXSID201017117
DTXSID20108653
DTXSID20108830
DTXSID20152373
DTXSID2020268
DTXSID2020426
DTXSID2020688
DTXSID20210629
DTXSID2021238
DTXSID20219339
DTXSID2022121
DTXSID2024246
DTXSID2024951
DTXSID2026941
DTXSID2026943
DTXSID2029117
DTXSID2029242
DTXSID2029327
DTXSID2029458
DTXSID2034384
DTXSID2034388
DTXSID2035013
DTXSID2036405
DTXSID20400487
DTXSID2040313
DTXSID2041593
DTXSID20439075
DTXSID20452860
DTXSID20477018
DTXSID2049634
DTXSID2052732
DTXSID2052738
DTXSID20545726
DTXSID2061149
DTXSID2069365

DTXSID2071867
DTXSID2074110
DTXSID20783707
DTXSID20786910
DTXSID20858924
DTXSID20863371
DTXSID20872423
DTXSID20872645
DTXSID20872665
DTXSID20872766
DTXSID20872781
DTXSID20872786
DTXSID20872802
DTXSID20873773
DTXSID20879851
DTXSID20879856
DTXSID20879871
DTXSID20879876
DTXSID20879891
DTXSID20879896
DTXSID20879912
DTXSID20879917
DTXSID20879932
DTXSID20879937
DTXSID20879952
DTXSID20879957
DTXSID20879972
DTXSID20879977
DTXSID20891513
DTXSID20893653
DTXSID20894307
DTXSID20894685
DTXSID2094005
DTXSID20960293
DTXSID20960334
DTXSID301012772
DTXSID301016681
DTXSID301016693
DTXSID301016700
DTXSID301018499
DTXSID30105330
DTXSID30108225
DTXSID30109297
DTXSID30195216
DTXSID3020174
DTXSID30209584
DTXSID3021770

DTXSID30218116
DTXSID30219340
DTXSID3021986
DTXSID3024233
DTXSID3024861
DTXSID3025382
DTXSID3025465
DTXSID3025833
DTXSID3027530
DTXSID3027615
DTXSID3028269
DTXSID3029813
DTXSID3030056
DTXSID3032129
DTXSID3040306
DTXSID3043786
DTXSID3044918
DTXSID30451985
DTXSID30461246
DTXSID30467127
DTXSID3051206
DTXSID3051250
DTXSID3051466
DTXSID3052307
DTXSID3052692
DTXSID3052698
DTXSID30573656
DTXSID3060950
DTXSID3067992
DTXSID3068043
DTXSID30708183
DTXSID3074789
DTXSID30785353
DTXSID30785570
DTXSID30858238
DTXSID30860639
DTXSID30864056
DTXSID30866010
DTXSID30872671
DTXSID30872797
DTXSID30879862
DTXSID30879867
DTXSID30879882
DTXSID30879887
DTXSID30879903
DTXSID30879908
DTXSID30879923

DTXSID30879928
DTXSID30879943
DTXSID30879948
DTXSID30879963
DTXSID30879968
DTXSID30879983
DTXSID30879988
DTXSID30881107
DTXSID30885564
DTXSID30892415
DTXSID30949579
DTXSID30958080
DTXSID30972327
DTXSID3097842
DTXSID30992969
DTXSID401010943
DTXSID401012399
DTXSID401012987
DTXSID401016672
DTXSID401016696
DTXSID401016703
DTXSID401017113
DTXSID40103802
DTXSID40105260
DTXSID40106732
DTXSID40109400
DTXSID40152375
DTXSID40160258
DTXSID40164493
DTXSID40185608
DTXSID4021341
DTXSID4021391
DTXSID4021420
DTXSID4023880
DTXSID4024640
DTXSID4025828
DTXSID4026216
DTXSID4026264
DTXSID4026266
DTXSID4027527
DTXSID4028880
DTXSID4030047
DTXSID4038922
DTXSID40412148
DTXSID4044161
DTXSID40477015
DTXSID4049660

DTXSID4049662
DTXSID4051661
DTXSID4051665
DTXSID4052685
DTXSID4052687
DTXSID4052689
DTXSID4052710
DTXSID4052712
DTXSID4052716
DTXSID40556652
DTXSID4061412
DTXSID40616285
DTXSID4063290
DTXSID4072154
DTXSID40724016
DTXSID4074770
DTXSID4074774
DTXSID4074776
DTXSID40858926
DTXSID40864926
DTXSID40872667
DTXSID40872703
DTXSID40872768
DTXSID40872788
DTXSID40873533
DTXSID40873972
DTXSID40879853
DTXSID40879858
DTXSID40879873
DTXSID40879878
DTXSID40879893
DTXSID40879898
DTXSID40879914
DTXSID40879919
DTXSID40879934
DTXSID40879939
DTXSID40879954
DTXSID40879959
DTXSID40879974
DTXSID40879979
DTXSID40885873
DTXSID40890902
DTXSID40893054
DTXSID40925980
DTXSID40929084
DTXSID40949863
DTXSID501000614

DTXSID501016675
DTXSID501016699
DTXSID501016706
DTXSID501016863
DTXSID501017085
DTXSID501017116
DTXSID50107134
DTXSID50192686
DTXSID5020235
DTXSID5020493
DTXSID5021174
DTXSID5021330
DTXSID5021411
DTXSID5021413
DTXSID5021758
DTXSID50218113
DTXSID5023875
DTXSID5024348
DTXSID5024639
DTXSID5025071
DTXSID5025150
DTXSID5026259
DTXSID5027980
DTXSID5028873
DTXSID5028902
DTXSID5029186
DTXSID5029689
DTXSID5034690
DTXSID50448655
DTXSID5044942
DTXSID5049817
DTXSID5051317
DTXSID5051733
DTXSID5052676
DTXSID5052705
DTXSID50573491
DTXSID50577712
DTXSID5063071
DTXSID5065196
DTXSID50702527
DTXSID5074769
DTXSID50858795
DTXSID50858856
DTXSID50872597
DTXSID50873428
DTXSID50873928
DTXSID50879864

DTXSID50879869
DTXSID50879884
DTXSID50879889
DTXSID50879900
DTXSID50879905
DTXSID50879920
DTXSID50879925
DTXSID50879940
DTXSID50879945
DTXSID50879960
DTXSID50879965
DTXSID50879980
DTXSID50879985
DTXSID50881109
DTXSID50894315
DTXSID50950561
DTXSID50957474
DTXSID50988682
DTXSID601004590
DTXSID601009714
DTXSID601010963
DTXSID601015670
DTXSID601016680
DTXSID601016692
DTXSID601017119
DTXSID601018498
DTXSID60107625
DTXSID60108450
DTXSID60108955
DTXSID60109402
DTXSID60152372
DTXSID60162597
DTXSID60199202
DTXSID6020802
DTXSID60219338
DTXSID6021959
DTXSID60229856
DTXSID60232825
DTXSID6024129
DTXSID6024626
DTXSID6024701
DTXSID6025484
DTXSID6025800
DTXSID6026084
DTXSID6026246
DTXSID6029121
DTXSID6029705

DTXSID6030782
DTXSID6032192
DTXSID6044563
DTXSID6044939
DTXSID60477017
DTXSID6050122
DTXSID6050467
DTXSID6051770
DTXSID6052168
DTXSID60550652
DTXSID60583561
DTXSID60591606
DTXSID6060896
DTXSID6064793
DTXSID6068854
DTXSID60783706
DTXSID60864948
DTXSID60872265
DTXSID60872422
DTXSID60872629
DTXSID60872669
DTXSID60872765
DTXSID60872780
DTXSID60872801
DTXSID60873772
DTXSID60879850
DTXSID60879855
DTXSID60879870
DTXSID60879875
DTXSID60879890
DTXSID60879895
DTXSID60879911
DTXSID60879916
DTXSID60879931
DTXSID60879936
DTXSID60879951
DTXSID60879956
DTXSID60879971
DTXSID60879976
DTXSID60886428
DTXSID6091554
DTXSID60919462
DTXSID6094435
DTXSID60970533
DTXSID60974814
DTXSID60975467
DTXSID6098467

DTXSID60988350
DTXSID701010942
DTXSID701014956
DTXSID701016671
DTXSID701016683
DTXSID701016695
DTXSID701016702
DTXSID701017112
DTXSID70107934
DTXSID70180181
DTXSID70190063
DTXSID70195215
DTXSID7020182
DTXSID7020895
DTXSID7020899
DTXSID7021782
DTXSID70218014
DTXSID70218115
DTXSID7021817
DTXSID7023805
DTXSID7024823
DTXSID7024873
DTXSID7024950
DTXSID7025051
DTXSID7026102
DTXSID70274039
DTXSID70274251
DTXSID70275889
DTXSID7027750
DTXSID7027887
DTXSID7028061
DTXSID7028695
DTXSID7029740
DTXSID7032262
DTXSID7034387
DTXSID7034389
DTXSID7034410
DTXSID7035016
DTXSID7039672
DTXSID7040154
DTXSID7042518
DTXSID7047489
DTXSID7047514
DTXSID7052735
DTXSID70563233
DTXSID70565813
DTXSID7059432

DTXSID7060910
DTXSID7065912
DTXSID70675901
DTXSID70728087
DTXSID7073486
DTXSID7074749
DTXSID7075212
DTXSID70760588
DTXSID70785695
DTXSID70849024
DTXSID70858838
DTXSID70867243
DTXSID70872670
DTXSID70872771
DTXSID70872776
DTXSID70872791
DTXSID70872796
DTXSID70879861
DTXSID70879866
DTXSID70879881
DTXSID70879886
DTXSID70879902
DTXSID70879907
DTXSID70879922
DTXSID70879927
DTXSID70879942
DTXSID70879947
DTXSID70879962
DTXSID70879967
DTXSID70879982
DTXSID70880073
DTXSID70881106
DTXSID70885508
DTXSID70928904
DTXSID70956080
DTXSID70965470
DTXSID70983452
DTXSID801016698
DTXSID801016705
DTXSID801017115
DTXSID80107228
DTXSID80107622
DTXSID80149507
DTXSID80151024
DTXSID80152374
DTXSID80201295
DTXSID80212649

DTXSID80220168
DTXSID8023927
DTXSID8024236
DTXSID8024319
DTXSID8024947
DTXSID8025383
DTXSID8025830
DTXSID8026228
DTXSID8027618
DTXSID8028000
DTXSID8029363
DTXSID8035681
DTXSID8040698
DTXSID8042476
DTXSID8043781
DTXSID8047507
DTXSID8052693
DTXSID8052697
DTXSID8052720
DTXSID80548894
DTXSID8065824
DTXSID8069197
DTXSID8073471

DTXSID8073471
DTXSID8074287
DTXSID80772293
DTXSID80776039
DTXSID80858783
DTXSID80858925
DTXSID80862522
DTXSID80863938
DTXSID80866227
DTXSID80872267
DTXSID80872666
DTXSID80872782
DTXSID80872787
DTXSID80873971
DTXSID80877030
DTXSID80879852
DTXSID80879857
DTXSID80879872
DTXSID80879877
DTXSID80879892
DTXSID80879897
DTXSID80879913
DTXSID80879918

DTXSID80879933
DTXSID80879938
DTXSID80879953
DTXSID80879958
DTXSID80879973
DTXSID80879978
DTXSID80893715
DTXSID80894308
DTXSID80904259
DTXSID901010948
DTXSID901016653
DTXSID901016677
DTXSID901016689
DTXSID901016691
DTXSID901017087
DTXSID901017118
DTXSID901018497
DTXSID90179830
DTXSID90192685
DTXSID90199295
DTXSID9020164
DTXSID9020376
DTXSID90209646
DTXSID9022079
DTXSID90233264
DTXSID9024015
DTXSID9024641
DTXSID9025326
DTXSID9026261
DTXSID9026267
DTXSID90274233
DTXSID9028912
DTXSID9029855
DTXSID9030048
DTXSID9040213
DTXSID9040215
DTXSID9047548
DTXSID9047754
DTXSID9050488
DTXSID9051589
DTXSID9051957
DTXSID9052686
DTXSID9052688
DTXSID9052711
DTXSID9052713
DTXSID9052715
DTXSID9052719

DTXSID90534622
DTXSID90573490
DTXSID90577711
DTXSID9061677
DTXSID9064639
DTXSID9065572
DTXSID9066752
DTXSID9068328
DTXSID9068407
DTXSID90700608
DTXSID9070406
DTXSID90724142
DTXSID9074771
DTXSID9074773
DTXSID9074775
DTXSID90785354
DTXSID90785697
DTXSID90785733
DTXSID90866551
DTXSID90872773
DTXSID90872778
DTXSID90872793
DTXSID90872798
DTXSID90873922
DTXSID90873927
DTXSID90879863
DTXSID90879868
DTXSID90879883
DTXSID90879888
DTXSID90879904
DTXSID90879909
DTXSID90879924
DTXSID90879929
DTXSID90879944
DTXSID90879949
DTXSID90879964
DTXSID90879969
DTXSID90879984
DTXSID90881108
DTXSID90884159
DTXSID90889689
DTXSID90894430
DTXSID90953470
DTXSID90963559
DTXSID401029839
DTXSID701029840

Preferrred Name
Methane, bromochlorodifluoro-
Guanidine carbonate
1,2-Dibromotetrafluoroethane
Bromoform
Sodium acetate
Trifluoroiodomethane
Guanidine carboxamide
4-Bromophenol
Antimony & Antimony Compounds
4-Bromostyrene
Urea
Asbestos
Perfluoro-2-methyl-3-pentanone
myo-Inositol hexakisphosphate
Polychlorinated biphenyls
Polyvinyl chloride
Bromotrifluoromethane
2-Bromopropenal
3-Bromostyrene
Etidronic acid
Antimony trisulfide colloid
Fumes, silica
Ammonium iodide
Fiberglass
Methyl bromoacetate
polychlorodibenzo-4-dioxin
Vinyl bromide
Perfluorooctanoic acid
Sodium bisulfite
2-Bromophenol
4-Bromobutyric acid
Ethene, 1,1,2,2-tetrabromo-
Ammonium fluoroborate
Tetrakis(hydroxymethyl)phosphanium iodide
Triethanolamine
Diphenyl oxide
2-Bromopropionic acid
Sodium silicate
Diethyl phosphite
Calcium
Phenol, 3-bromo-
Bis[(pentabromophenyl)methyl] 3,4,5,6-tetrabromobenzene-1,2-dicarboxylate
Hexacosane, chloro derivs.

Calcium molybdate(VI)
3,3',5,5'-Tetrabromobisphenol A bispropionate
dimethyl {[(4,6-dichloro-1,3,5-triazin-2-yl)oxy]methyl}phosphonate
1-(2,3-dibromopropyl)-3,5-di(prop-2-en-1-yl)-1,3,5-triazinane-2,4,6-trione
1,3-bis(2,3-dibromopropyl)-5-(prop-2-en-1-yl)-1,3,5-triazinane-2,4,6-trione
Octabromobiphenyl
End capped and partially end capped brominated epoxy oligomer
Aluminium sodium tetrahydroxide
Phosphonic acid, P-methyl-, dimethyl ester, polymer with oxirane and phosphorus oxi
Polyphosphoric acids, compds. with pyrolyzed melamine
4,4',6,6'-Tetrabromo-2,2'-biphenyldiol
Ethanol, 2-bromo-, phosphate (3:1)
2,2',3,4,4',5,6,6'-Octabromodiphenyl ether
2,4-Dichlorodiphenyl ether
Ammonium chloride
Dimethyl methylphosphonate
2,3,5,6,2',3',5',6'-Octabromobiphenyl
Firemaster FF-1
Tetrakis(hydroxymethyl)phosphonium sulfate
Tris(2-ethylhexyl) phosphate
Tris[2-(propan-2-yl)phenyl] phosphate
1,1'-Biphenyl, 2,2',3,4',5',6-hexabromo-
Azodicarbonamide
Triphenyl phosphite
Tris(2-chloroethyl) phosphite
Tar acids, cresylic, Ph phosphates
Bis(2-chloro-1-methylethyl) 2-chloropropyl phosphate
Benzene, 1,3-dibromo-2-phenoxy-
Vinyl acetate ethylene copolymer
Barium sulfate
Aluminium hydroxide oxide
2,2'-[(1-Methylethylidene)bis[(2,6-dibromo-4,1-phenylene)oxymethylene]]bis[oxirane
1,2,3,4,6,7,8,9,10,10,11,11-dodecachloro-1,4,4a,5a,6,9,9a,9b-octahydro-1,4:6,9-dime
4,4'-Sulphonylbis[2,6-dibromophenol]
1,2,5,6-Tetrabromocyclooctane
Zinc borate (ZnO 3 2B2O3)
1,3-Dibromo-5-(3-bromophenoxy)benzene
Tris(tribromoneopentyl)phosphate
2,4,6-Tris-(2,4,6-tribromophenoxy)-1,3,5-triazine
Phenol, 2,6-dibromo-
Dimethyl propylphosphonate
1,1'-Biphenyl, 3,3',5,5'-tetrabromo-
Alkanes, C16-35, chloro
Aluminum diethylphosphinate
Phenol, 4,4'-(1-methylethylidene)bis[2,6-dibromo-, polymer with carbonic dichloride,
(+/-)-α-Hexabromocyclododecane
BDE-19

BDE-24
BDE-50
BDE-57
BDE-74
BDE-81
BDE-95
BDE-103
BDE-122
BDE-129
BDE-147
BDE-152
BDE-172
BDE-178
Benzenesulfonic acid, 3,3'-sulfonylbis-, potassium salt (1:2)
Dipotassium hexafluorozirconate(2-)
Bromkal 70
2-bromoallyl 2,4,6-tribromophenyl ethe
Bis(2,3-dibromopropyl) hydrogen phosphate--ammonia (1/1)
Tetrakis(1-chloropropan-2-yl) ethane-1,2-diyl bis(phosphate)
Tris(2-bromo-4-methylphenyl) phosphate
1,2,3,4,5-Pentabromo-6-(bromomethyl)benzene
Tetrakis(hydroxymethyl)phosphanium phosphate acetate (4/1/1)
Tetrakis(hydroxymethyl)phosphanium naphthalene-2-sulfonate
Crude natural boric acid
Poly-(m-phenylene methylphosphonate)
bis(2,3-dibromopropyl) 3,4,5,6-tetrabromobenzene-1,2-dicarboxylate
Boric acid (H3BO3), compound with 1,3,5-triazine-2,4,6-triamine
Alkanes, C20-24, chloro
1,3-Propanediamine, N1,N1'-1,2-ethanediylbis-, reaction products with cyclohexane a
6-Phenyl-1,3,5-triazine-2,4-diamine
Diphenyl ether, dibromo-
Boric acid (H3BO3)
3,3',4,4',5,5'-Hexabromobiphenyl
Trimethyl phosphate
2,4,5,3',4'-Pentabromobiphenyl
Triphenyl phosphate
Phosphonium, tetrakis(hydroxymethyl)-, 1-naphthalenesulfonate (salt)
Hexabromobenzene
Phosphorus
1,2-Bis(2,4,6-tribromophenoxy)ethane
Dicumyl peroxide
2-Ethylhexyl diphenyl phosphate
3,3',5,5'-Tetrabromobisphenol A
1,1,2,2-Tetrabromoethane
Bis(2-ethylhexyl) phosphate
Tris(3,5-xylenyl)phosphate
Tetrabromophthalic acid mixed esters with diethylene glycol and propylene glycol

Phosphoric acid, mixed 3-bromo-2,2-dimethylpropyl and 2-bromoethyl and 2-chloroe
Silica
Ammonium sulfate
Chlorinated paraffins
Barium metaborate
Disodium octaborate tetrahydrate
Diethyl hydrogen phosphate
Diphenyl phosphate
o-Terphenyl
Dibutyl chlorendate
2,4-Dibromophenol
Calcium phosphinate
2,4-Dibromo-1-(3-bromophenoxy)benzene
1,1'-Biphenyl, 4,4'-dibromo-
2,4,6-Tribromoanisole
Phosphonic acid, P-phenyl-, dioctyl ester
Butyl diphenyl phosphate
Phosphonium, tetrakis(hydroxymethyl)-, acetate (salt)
Phosphonium, tetrakis(hydroxymethyl)-, ethanedioate (2:1) (salt)
Propanenitrile, 3,3',3'',3'''-[(2,3,5,6-tetramethyl-1,4-phenylene)bis(methylenephosphi
2,4,8,10-Tetraoxa-3,9-diphosphaspiro[5.5]undecane, 3,9-bis[3-bromo-2,2-bis(bromor
1,2,3,5-Tetrabromo-4-(3,4,5-tribromophenoxy)benzene
Bis(p-tert-butylphenyl) phenyl phosphate
1-Propanol, 3,3'-oxybis[2,2-bis(bromomethyl)-
Phosphoric acid, trihexyl ester
Pentabromophenyl benzoate
1,3,5-Tribromo-2-(2,3-dibromophenoxy)benzene
Tetrabromobisphenol A dimethyl ether
C10-12 chloroalkanes
Boron phosphate (B(PO4))
Brominated epoxy resin end-capped with tribromophenol
Dimelamine monophosphate
2,2',4,4',6,6'-Hexabromodiphenyl Ether
BDE-29
BDE-38
BDE-58
BDE-63
BDE-84
1,2,5-Tribromo-3-(2,4-dibromophenoxy)benzene
BDE-109
BDE-114
BDE-136
BDE-143
BDE-162
BDE-167
BDE-193
Diphosphoric acid, compd. with piperazine (1:1)

3,5,3',5'-Tetrabromobisphenol A, epichlorohydrin polymer
Zinc molybdate(VI) (ZnMoO4)
Calcium borate
Bromine--methane (1/16)
2,3,4,5,2',3',4',5'-Octabromobiphenyl
2,2',5,5'-Tetrabromobiphenyl
Tris(chloropropyl)phosphate
(-)-beta-Hexabromocyclododecane
Piperazine, phosphate (1:?)
2,3,4,5-Tetrabromobenzoic Acid
(rel)-(1R,2S,3S,4S)-1,2,3,9-tetrabromo-1,2,3,4-tetrahydro-1,4-methanonaphthalene
anti-Dechlorane Plus
Tetrakis(hydroxymethyl)phosphonium p-toluenesulfonate
Benzene, ethenyl-, polymer with 1,3-butadiene, brominated
1H-Indene, 2,3-dihydro-1,1,3-trimethyl-3-phenyl-, octabromo deriv.
2,2',3,3',4,5',6,6'-Octabromobiphenyl
Chlorendic acid
Dicloran
Hexachlorocyclopentadiene
Dioctyl phenyl phosphate
Resorcinol
1,1'-Biphenyl, 2,2',3,3',4,4',5,6'-octabromo-
Triphenylphosphine oxide
Pentabromodiphenyl ether
2,3-Dibromopropylmethacrylate
Chlorendic anhydride
Pentaerythritol
1,1':3',1''-Terphenyl
Diethyl (N,N-bis(2-hydroxyethyl)amino)methanephosphonate
Tetrabromobisphenol A diallyl ether
Bromo chloro C12-30 a-alkenes
Borax (B4Na2O7.10H2O)
Sodium tetraborate (Na2B4O7)
Zinc chloride
Aluminum hydroxide
diethylphosphinic acid
Cercelor S 52
Alumina
Tribromostyrene
1,3,5-Triazine, 2,4,6-tris(2,3-dibromopropoxy)-
Benzene, 1,2,3-tribromo-4-(2,3-dibromophenoxy)-
Graphite
1,1'-Ethane-1,2-diylbis(pentabromobenzene)
Tetraethyl ethylenebisphosphonate
2,3,4,5-Tetrabromophenol
Tributylphosphine oxide
1H-Pyrrole-2,5-dione, 1-(2,4,6-tribromophenyl)-

Diphenyl ether, heptabromo derivative
2-tert-Butylphenyl diphenyl phosphate
1,3-Dibromo-2-(3-bromophenoxy)benzene
1,2,3,4-Tetrabromo-5-(3,4-dibromophenoxy)benzene
Alkanes, C18-20, chloro
Phosphonic acid, P-[1-[[(2-chloroethoxy)(2-chloroethyl)phosphinyl]oxy]ethyl]-, 1-[bis(
Medium-chain chlorinated paraffins (C14-17)
Triphenyl phosphates tert-butylated
Alkanes, C10-21, chloro
Ethylenebis[tris(2-cyanoethyl)phosphonium bromide]
Tetrakis(hydroxymethyl)phosphonium tetraphenylborate-tetraacetate
1,2-Dibromo-4,5,6,7,8,8-hexachloro-2,3,3a,4,7,7a-hexahydro-4,7-methano-1H-indene
Aluminum hypophosphite
(+/-)-gamma-Hexabromocyclododecane
BDE-6
BDE-21
BDE-45
BDE-53
BDE-70
BDE-76
BDE-97
BDE-105
BDE-124
BDE-132
BDE-149
BDE-158
BDE-175
BDE-182
1,3,5,7,9,11-Hexabromocyclododecane
2,2-Bis(chloromethyl)-1,3-propanediyl tetrakis(1-chloro-2-propanyl) bis(phosphate)
Alkanes, C22-30-branched and linear, chloro
Phosphoric acid, bis(methylphenyl) phenyl ester
Aluminum phosphate (Al(H2PO4)3)
Tetrakis(hydroxymethyl)phosphanium 2-hydroxypropanoate
Tetrakis(hydroxymethyl)phosphanium 3-carboxy-3-hydroxypropanoate
Carbonic acid, diphenyl ester, polymer with diphenyl P-methylphosphonate and 4,4'-(
diethyl (4,6-dichloro-1,3,5-triazin-2-yl)phosphonate
Poly(tribromostyrene)
Dechloran 604
Alkanes, C20-28, chloro
Benzene, ethenyl-, homopolymer, brominated
1,4:5,8:9,10-Trimethanoanthracene, 1,2,3,4,5,6,7,8,12,12,13,13-dodecachloro-1,4,4a
Paraffins (petroleum), normal C>10, chloro
1,1':3',1''-Terphenyl, 2,2',2'',3,3'',4,4',4'',5,5',5'',6,6',6''-tetradecachloro-
Bis(2,3-dibromopropyl) phosphate, magnesium salt
Isopropyl diphenyl phosphate
2,2',6,6'-Tetrachlorobisphenol A

2,3',4,4'5,5'-Hexabromobiphenyl
2,2',3,3',4,4',5,5',6-Nonabromo-1,1'-biphenyl
Tributyl phosphate
Nonabromodiphenyl ether
Cresyl diphenyl phosphate
Hexabromobiphenyl
Isodecyl diphenyl phosphate
Pentabromotoluene
Trimethylolpropane trimethacrylate
Tris(2-chloropropyl) phosphate
Bis(tert-butylphenyl)phenyl phosphate
Chlorinated paraffin oils
2,2',4,4'-Tetrabromodiphenyl ether
Tetrabromobisphenol A-bis(2,3-dibromopropyl ether)
Chlorendate dimethyl
Tetrakis(hydroxymethyl)phosphonium
1,3,5-Tribromo-2-(prop-2-en-1-yloxy)benzene
BDE-207
3-[Hydroxy(phenyl)phosphoryl]propanoic acid
(+)-beta-Hexabromocyclododecane
Sodium aluminate
Polyvinylidene chloride
Tris(4-tert-butylphenyl) phosphate
1,3,5-Tribromobenzene
2,2',4,4',5,6'-Hexabromodiphenyl ether
Tripentyl phosphate
1,1'-[Oxybis(methylene)]bis(pentabromobenzene)
1,3,5-Triazin-2(1H)-one, 4,6-diamino-
2-Butene, 1,1,2,3,4,4-hexabromo-
1,3,5-Triazine-2,4,6(1H,3H,5H)-trione, compd. with 1,3,5-triazine-2,4,6-triamine (1:1)
1,1'-Oxybis(3,5-dibromobenzene)
BDE-196
1,2,3,5-Tetrabromo-4-(2,4-dibromophenoxy)benzene
1,2,4-Tribromo-5-(3,5-dibromophenoxy)benzene
4,5,6,7-Tetrabromo-1,1,3-trimethyl-3-(2,3,4,5-tetrabromophenyl)-2,3-dihydro-1H-ind
2,3,4,5,6,8-Hexachlorodecane
1,2-Benzenedicarboxylic acid, 1,2-bis(2,3-dibromopropyl) ester
4,7-Methano-1H-isoindole-1,3(2H)-dione, 5,6-dibromohexahydro-2-phenyl-
Alkanes, C12-14, chloro
Potassium 3-(phenylsulfonyl)benzenesulfonate
BDE-34
BDE-41
BDE-60
BDE-65
BDE-87
BDE-92
BDE-111

BDE-117
BDE-140
BDE-145
BDE-164
BDE-169
BDE-200
BDE-198
1,2,3,4,5-Pentabromo-6-(2,3,4,5-tetrabromophenoxy)benzene
Phenol, 4,4'-(1-methylethylidene)bis[2,6-dibromo-, 1,1'-diacetate
Sodium hexahydroxyantimonate(1-)
1,2,3-Tribromo-4-[(prop-2-en-1-yl)oxy]benzene
Ethenyl dihydrogen phosphate
1,2,3,4,7,7-Hexachloro-5-(2,4,6-tribromophenyl)bicyclo[2.2.1]hept-2-ene
Ammonium polyphosphates
Bis(1,3-dichloropropan-2-yl) hydrogen phosphate
Octadecane, chloro derivs.
2-Butyne-1,4-diol, polymer with 2-(chloromethyl)oxirane, brominated, dehydrochlori
Carbonic dichloride, polymer with 4,4'-(1-methylethylidene)bis[2,6-dibromophenol] a
(-)-alpha-Hexabromocyclododecane
Tribromostyrene (mixed isomers)
Hexabromohexane
Huntite
Alkanes, C12-13, chloro
C10-13 chloro alkanes
Benzene, ethenyl-, ar-bromo derivs., homopolymers
Phosphinic acid, P,P-diethyl-, zinc salt (2:1)
2,2',3,3',4,5,5',6,6'-Nonobromobiphenyl
Zirconium phosphate
Melem
p-Tetradecachloroterphenyl
4,4'-Thiobis(6-tert-butyl-m-cresol)
Tris(methylphenyl) phosphate
Sodium tungsten oxide (Na2WO4)
Antimony trioxide
4-Bromobiphenyl
Pentabromochlorocyclohexane
Tri-m-tolyl phosphate
Tris(2-ethylhexyl) phosphite
Tris(2-hydroxyethyl) isocyanurate
1,2,5,6,9,10-Hexabromocyclododecane
Triphenyl phosphates isopropylated
2,2',4,4',5,5'-Hexabromodiphenyl ether
Tetrabromobisphenol A bis(2-hydroxyethyl) ether
Benzene, pentabromo(2,3-dibromopropoxy)-
Antimony trichloride
3,4-Dibromodiphenyl ether
Magnesium carbonate (1:1)

Magnesium hydroxide
Boehmite
2-Biphenylyl diphenyl phosphate
2,2',3,4,4'-Pentabromodiphenyl ether
Benzene hexabromide
2,2',4,4',6-Pentabromodiphenyl ether
2,4,4'-Tribromodiphenyl ether
Tripropyl phosphate
Tri(aziridin-1-yl)phosphine oxide
2,2',3,3',4,5,5',6,6'-nonabromodiphenyl ether
1,3,5-Triazine-2,4,6-triyltriiminotrimethanol
1,3-Dibromo-2-(2,4-dibromophenoxy)benzene
1,3,2-Dioxaphosphorinane, 2,2'-oxybis[5,5-dimethyl-, 2,2'-disulfide
1,3,5-Triazine-2,4,6-triamine, compd. with 3,9-dihydroxy-2,4,8,10-tetraoxa-3,9-diphos
1,2,4-Tribromo-5-(3,4-dibromophenoxy)benzene
PBB 077
PBDE 195
PBDE 205
2,2',3,3',4,4'-Hexabromo-1,1'-biphenyl
2,4-Dibromophenyl glycidyl ether
Alkanes, C16-27, chloro
2,2',4-Tribromodiphenyl ether
Tetraammonium octamolybdate
Melapur 200
2,2',3,3',4,4',5,6,6'-Nonabromo-1,1'-biphenyl
BDE-180
BDE-18
BDE-23
BDE-48
BDE-56
BDE-73
BDE-79
BDE-102
BDE-107
BDE-127
BDE-134
BDE-151
BDE-160
BDE-177
BDE-187
1,2-Benzenedicarboxylic acid, 3,4,5,6-tetrabromo-, 1-butyl ester
Calcium borate silicate
Tribromoneopentyl alcohol
Sulfuric acid--methane (1/1)
1,2,3,4,5,6,7,8,10,10,11,11-Dodecachloro-4,4a,4b,5,8,8a,9,9a-octahydro-1H-1,4:5,8-c
Tetrakis(hydroxymethyl)phosphanium carbamimidate--hydrogen chloride (1/1/1)
1~2~,1~3~,1~4~,1~5~,1~6~,2~3~,2~4~,3~2~,3~3~,3~4~,3~5~,3~6~-Dodecabromo-1~

(+)-alpha-Hexabromocyclododecane
1,1'-sulfonylbis(3,5-dibromo-4-methoxybenzene)
(rel)-(1R,2R,3S,4S)-1,2,3,9-tetrabromo-1,2,3,4-tetrahydro-1,4-methanonaphthalene
syn-Dechlorane Plus
2,3-Dibromopropylphosphate
Phosphonium, tetrakis(hydroxymethyl)-, salt with cellulose carboxymethyl ether
Benzene, ethenyl-, ar-bromo derivs., polymers with propene, graft
2,3,4,5-Tetrabromo-6-chlorotoluene
Calcium chloride
Dimethyl phosphonate
Polybrominated biphenyls (PBB)
Tetramethylolphosphonium chloride
Tris(2-chloroethyl) phosphate
Tris(2,3-dibromopropyl) phosphate
Tris(2-butoxyethyl) phosphate
1,1'-Biphenyl, 2,2',4,5,5'-pentabromo-
Ammonium sulfamate
2',3,4-Tribromodiphenyl ether
3-Bromobiphenyl
1,2-Dihydro-2,2,4-trimethylquinoline, polymer
Dimethyl phosphate
Tris(2-chloroisopropyl)phosphate
Phosphoric acid, 1,2-ethanediyl tetrakis(2-chloroethyl) ester
Alkanes, C6-18, chloro
Tar acids, cresylic, C8-rich, phosphates
6-Methyl-1,3,5-triazine-2,4-diamine
Ammonium phosphate
Magnesium chloride
Benzene, 1,2,3,5-tetrabromo-4-(2,4,6-tribromophenoxy)-
1,1'-Sulfonylbis[3,5-dibromo-4-(2,3-dibromopropoxy)benzene]
Zinc carbonate
Diammonium tetraborate
2,4,6-Tribromoaniline
Tris(4-methylphenyl) phosphate
1,3,5-Tribromo-2-(2,3-dibromopropoxy)benzene
1,2,3-Tribromo-5-(3,4-dibromophenoxy)benzene
1-Bromo-2-(4-bromophenoxy)benzene
Allyl pentabromophenyl ether
Tin zinc oxide (SnZnO3)
1,2,4,5-Tetrabromo-3-(2,4,6-tribromophenoxy)benzene
PBB 053
Tris(isopropylphenyl) phosphate
Disodium stannate
Tin zinc hydroxide (ZnSn(OH)6)
Dibutyl phenyl phosphate
2,3',4,4',6-Pentabromodiphenyl Ether
BDE-37

BDE-43
BDE-62
2,4-Dibromo-1-(3,5-dibromophenoxy)benzene
BDE-83
BDE-89
BDE-108
BDE-113
BDE-135
BDE-142
BDE-161
BDE-166
BDE-189
BDE-202
Tribromodiphenyl ether
Alkanes, C21-34-branched and linear, chloro
Tris(2,4-dibromophenyl) phosphate
1,3,5-Tribromo-2-(2,3-dibromo-2-methylpropoxy)benzene
1-Bromo-4-chlorodecane
2-Bromoethyl 5-bromopentyl 2-chloroethyl phosphate
1,3,5-Triazine-2,4,6-triamine, polymer with formaldehyde
Slack Wax (petroleum), chloro
Carbonic dichloride, polymer with 4,4'-(1-methylethylidene)bis[2,6-dibromophenol] a
2,4,5,6,7-pentabromo-1,1,3-trimethyl-3-(2,4,6-tribromophenyl)-2,3-dihydro-1H-inder
dimethyl 3,4,5,6-tetrabromobenzene-1,2-dicarboxylate
Tetrakis(hydroxymethyl)phosphonium acetate-phosphate (1:1)
Alkanes, C22-30, chloro
Antiblaze 78
Phosphoric acid, P,P'-1,3-phenylene P,P,P',P'-tetrakis(2,6-dimethylphenyl) ester
Polyphosphoric acids, compds. with piperazine
Phosphonium, tetrakis(hydroxymethyl)-, chloride (1:1), reaction products with 1-tetra
1,1'-Biphenyl, 2,2',3,3',4,4',6,6'-octabromo-
2,3,4,6-Tetrabromophenol
s-Triazine, 2,2'-O-phenylene-bis(4,6-diamino-
Melamine
2,2',3,3',4,4',5-Heptabromobiphenyl
2,4,6-Tribromophenol
3,5-Dibromo-2-(2,4-dibromophenoxy)phenol
2,2',3,3',5,5',6,6'-Octabromo-4-phenoxy-1,1'-biphenyl
Hexabromodiphenyl ether
1,2-Bis(tetrabromophthalimido)ethane
tert-Butylphenyl diphenyl phosphate
Isopropyl phenyl diphenyl phosphate
1,2,3,4,5,6,7,8-Octachloronaphthalene
4,5,6,7-Tetrabromo-1,3-Isobenzofurandione
Trioctyl phosphate
1,1':4',1''-Terphenyl
Diammonium hydrogen phosphate

Bentonite
Tri-o-cresyl phosphate
6H-Dibenzo[c,e][1,2]oxaphosphinine 6-oxide
1,3,5-Tribromo-2-methoxy-4-methylbenzene
2,4,6-Tribromodiphenyl ether
Phosphoric acid, ammonium salt (1:x)
Antimony pentoxide
Bis(2-ethylhexyl) phenyl phosphate
N,N'-(Ethylene)bis[4,5-dibromohexahydro-3,6-methanophthalimide]
1,1'-Oxybis(2,3,4-tribromobenzene)
1,2,3,4,5-Pentabromo-6-(2,4-dibromophenoxy)benzene
1,3-Dibromo-5-phenoxybenzene
Tetrachlorophthalic acid
Magnesium carbonate hydroxide (Mg5(CO3)4(OH)2)
Diethylene glycol bis[bis(2-chloroethyl)phosphate]
1,4-Dibromo-2-(3-bromophenoxy)benzene
2-(2-Hydroxyethoxy)ethyl 2-hydroxypropyl 3,4,5,6-tetrabromophthalate
2,2',3,4,4',5'-Hexabromodiphenyl Ether
Long-chain chlorinated paraffins (C18-20)
C18-28 Chloroalkanes
Alkanes, C10-26, chloro
Mixed esters of phosphoric acid with [1,1'-biphenyl]-4,4'-diol and phenol
Sodium antimonate
2,2-Bis(3,5-dibromo-4-hydroxyphenyl)propane-phosgene copolymer
(+/-)-beta-Hexabromocyclododecane
BDE-4
BDE-20
BDE-44
BDE-52
BDE-69
BDE-75
BDE-96
BDE-104
BDE-123
BDE-131
BDE-148
BDE-157
BDE-174
BDE-179
Benzene, 1,1'-[1,2-ethanediylbis(oxy)]bis[2,3,4,5,6-pentabromo-
Zinc borate
Tris(3-methylbutyl) phosphate
Ethanol, 2-chloro-, phosphate (3:1), homopolymer
(Propane-2,2-diyl)bis(2,6-dibromo-4,1-phenylene) diprop-2-enoate
Tetrakis(hydroxymethyl)phosphanium bromide
2,2',4,5-Tetrabromo-1,1'-biphenyl
1,3-Butadiene, homopolymer, brominated

Diphenyl methylphosphonate--4,4'-(propane-2,2-diyl)diphenol (1/1)
Octacosane, chloro derivs.
4,4'-(1-Methylethylidene)bisphenol tetrabromo deriv.
(+)-gamma-Hexabromocyclododecane
2,4,6-tribromo-3-(tetrabromopentadecyl)-phenol
Bromkal 80
N-2,3-Dibromopropyl-4,5-dibromohexahydrophthalimide
Fyrquel GT
Phosphoric acid, triethyl ester, polymer with oxirane and phosphorus oxide (P2O5)
Cyclodecane, hexabromo
Bis(thiopyrophosphoric acid)
1,1':2',1''-Terphenyl, 2,2'',3,3',3'',4,4',4'',5,5',5'',6,6',6''-tetradecachloro-
Bisphenol A
Mirex
Molybdenum trioxide
2,3,4,5,6-Pentabromoethylbenzene
1,1'-Biphenyl, 2,2',3,4,4',5,5'-heptabromo-
2,2',3,4,4',5-Hexabromobiphenyl
2,3-Dibromopropanol
Tris(2,3-dichloropropyl)phosphate
Chloro C22-26 alkanes
Cyanuric acid
2,3-Dibromopropyl acrylate
Diethyl ethylphosphonate
Tetrachlorophthalic anhydride
Diphenyl 4-tolyl phosphate
(2-Chloro-1-methylethyl) bis(2-chloropropyl) phosphate
Bis(2,3-dibromopropyl) hydrogen phosphate
Dechlorane Plus
Bis(2-ethylhexyl) tetrabromophthalate
Chloroalkanes
Alkenes, C12-24, chloro
Dipotassium hexafluorotitanate
Tungsten trioxide
Boric oxide
Boron sodium oxide pentahydrate
Calcium hydroxide
Zinc oxide
Phosphate
N-(3-Dimethylaminopropyl)methacrylamide
Zinc sulfide
Vermiculite
Calcium sulfate dihydrate
1,2-dibromo(phenyl)ethane
1,2,3,4-Tetrabromo-2,3-dimethylbutane
2,4-Dibromo-1-phenoxybenzene
Phosphoric acid, methyl diphenyl ester

1,2,4,5-Tetrabromobenzene
Diphosphoric acid, compd. with 1,3,5-triazine-2,4,6-triamine (1:?)
4,5,6,7,13,14,15,16,19,19,20,20-Dodecachloroheptacyclo[9.6.1.1~4,7~.1~13,16~.0~2,
1,2,3,4,5-Pentabromo-6-(chloromethyl)benzene
Benzene, bromophenoxy-
BDE-203
Phosphoric acid, dimethylphenyl diphenyl ester
4-Bromo-2-chlorobutyl 3-bromo-2,2-dimethylpropyl phosphate
1,3-Dibromo-5-(4-bromophenoxy)benzene
Bis[(pentabromophenyl)methyl] benzene-1,4-dicarboxylate
2,2',4,4',5,5'-Hexabromobiphenyl
Bis(p-acryloxyethoxy)tetrabromobisphenol A
Chloroalkanes, C10-14
Melamine-(aluminium phosphate)
Zinc Borate 2335
Bisphenol A-epichlorohydrin-2,2',6,6'-tetrabromobisphenol A copolymer
Poly(pentabromobenzyl acrylate)
BDE-32
BDE-40
BDE-59
BDE-64
BDE-86
BDE-91
BDE-110
BDE-115
BDE-137
BDE-144
BDE-163
BDE-168
BDE-199
Firemaster 550
1,2,3-Tribromo-4-(2,3,5-tribromophenoxy)benzene
1-Pentanone, 2,3,4,5-tetrabromo-1,5-diphenyl-
Diphosphoric acid--1,3,5-triazinane-2,4,6-triimine (1/2)
1,2,3,4,7,7-Hexachloro-5-(pentabromophenyl)bicyclo[2.2.1]hept-2-ene
Tetrakis(hydroxymethyl)phosphanium hydroxide
Sodium bis(2,3-dibromopropyl) phosphate
potassium bis(2,3-dibromopropyl) phosphate
Bis(polyoxyethylene) methylphosphonate
Cellulose, 6-carboxy, ion (neg.), tetrakis(hydroxymethyl)phosphonium
Phosphoric trichloride, reaction products with bisphenol A and phenol
Antiblaze 19
Antimonic acid, sodium salt
1,1'-Biphenyl, 2,4,5-tribromo-
2,2',3,4,4',5,6,6'-Octabromobiphenyl
Phosphonium, tetrakis(hydroxymethyl)-, ethanedioate (1:1) (salt)
Trimethylphosphoramide

2,2',3,3',4,5,5',6'-Octabromobiphenyl
p-Bromodiphenyl ether
Octabromodiphenyl ether
Tetrabromodiphenyl ether
1,2-Dibromo-4-(1,2-dibromoethyl)cyclohexane
Hexabromocyclododecane
Pentabromoethane
Triethyl phosphate
Bis(2-chloroethyl) 2-chloroethylphosphonate
Phosphoric acid, 2,2-bis(chloromethyl)-1,3-propanediyl tetrakis(2-chloroethyl) ester
(5-Ethyl-2-methyl-1,3,2-dioxaphosphorinan-5-yl)methyl dimethyl phosphonate P-oxid
Ammonium bromide
Triisobutyl phosphate
Titanium tetrachloride
Tetrabromophthalic acid
Perbromo-1,4-diphenoxybenzene
2,2',3,4,4',5',6-Heptabromodiphenyl ether
2,3,5,6-Tetrabromo-p-xylene
Phosphoric acid, P,P'-[(1-methylethylidene)di-4,1-phenylene] P,P,P',P'-tetraphenyl est
1,2,3,4,5-Pentabromo-6-phenoxybenzene
1,2-Ethanediamine, phosphate
Tetraphenyl m-phenylene bis(phosphate)
Benzene, dibromoethenyl-

Benzene, dibromoethenyl-
Octyl diphenyl phosphate
1,2-Dibromo-3-(2-bromophenoxy)benzene
(1R,2S,5S,6S,9S,10R)-1,2,5,6,9,10-Hexabromocyclododecane
Tris(4-isopropylphenyl) phosphate
Alkanes, C22-40, chloro
Tris(3-chloropropyl)phosphate
Tris(1,3-dichloropropan-2-yl) phosphite
7,8-Dibromo-1,2,3,4,11,11-hexachloro-1,4,4a,5,6,7,8,9,10,10a-decahydro-1,4-methar
2,3,3',4,4',5,6-Heptabromodiphenyl Ether
Alkanes, C10-32, chloro
4,4'-(1-Methylethylidene)bis[2,6-dibromophenol] polymer with (chloromethyl)oxiran
1,3,5-Triazine-2,4,6-triamine monophosphate
BDE-171
BDE-77
BDE-9
BDE-22
BDE-46
BDE-54
BDE-72
BDE-78
BDE-101
BDE-106

BDE-125
BDE-133
BDE-150
BDE-159
BDE-176
BDE-186
1,1'-(Isopropylidene)bis(3,5-dibromo-4-(2,3-dibromo-2-methylpropoxy)benzene)
Alkanes, C24-28, chloro
4,4'-(propane-2,2-diyl)bis(2-bromophenol)
Tetradecane, chloro derivs.
Tris(chloroethyl) phosphate
4,5,6,7-tetrabromo-1,1,3-trimethyl-3-(2,3,4,6-tetrabromophenyl)-2,3-dihydro-1H-ind
Tris(dichloropropyl) phosphate
1,1'-{[(2Z)-2,3-dibromobut-2-ene-1,4-diyl]bis(oxy)}bis(2,4,6-tribromobenzene)
pentabromocyclododecene
Torflam
Alkanes, C14-16, chloro
Phosphonium, tetrakis(hydroxymethyl)-, formate (salt)
3,6-Bis(bromomethyl)-1,2,4,5-tetrabromobenzene
Triisopropyl phosphate
Pentaerythritol dibromide
1,1'-Oxybis[2,3,4,5,6-pentabromobenzene]
Benzene, pentabromo-
Pentabromophenol
1,1'-Biphenyl, 2,3',4,4',5',6-hexabromo-
4,4'-Dibromodiphenyl ether
3-Bromo-2,2-bis(bromomethyl)propanol
Ferrocene
Tris(1,3-dichloro-2-propyl) phosphate
Trixylyl phosphate
2,3-Dibromo-2-butene-1,4-diol
Quaternary ammonium compounds, bis(hydrogenated tallow alkyl)dimethyl, salts wit
Phosphoric trichloride, polymer with 1,3-benzenediol, phenyl ester
2,2',4,4',5-Pentabromodiphenyl ether
Tris(hydroxymethyl)phosphine oxide
Dimethyl (3-((hydroxymethyl)amino)-3-oxopropyl)phosphonate
1,1'-Oxybis(2,3,4,5-tetrabromobenzene)
Phosphorus pentoxide
Calcium magnesium hydroxide oxide
Bis(2-chloroethyl) vinylphosphonate
Bis(isopropylphenyl) phenyl phosphate
2-Ethylhexyl 2,3,4,5-tetrabromobenzoate
2,3',4,4'-Tetrabromodiphenyl ether
Tris(dibromophenyl) phosphate
1,3,5-Tris(2,3-dibromopropyl)-1,3,5-triazine-2,4,6(1H,3H,5H)-trione
Methyl 2-bromohexanoate
(Pentabromophenyl)methyl acrylate

1,1'-Oxybis(3-bromobenzene)
1,4-Dibromo-2-(4-bromophenoxy)benzene
1-Bromo-3-(4-bromophenoxy)benzene
Antimony oxide (Sb2O4)
Tribromophenoxyethyl acrylate
1,1'-Biphenyl, 2,2',3,3',4,4',5,5',6,6'-decabromo-
Phosphonium, tetrakis(hydroxymethyl)-, phosphate (3:1) (salt)
1,3,5-Triazine-2,4,6-triamine, phosphate
Bis((5-ethyl-2-methyl-1,3,2-dioxaphosphorinan-5-yl)methyl) methyl phosphonate P,P'
1,1,2,2-Tetrabromocyclododecane
2,2-Bis(bromomethyl)-3-chloropropyl bis[2-chloro-1-(chloromethyl)ethyl] phosphate
Tris(2,4,6-tribromophenyl) phosphate
PBB 103
PBDE 002
BDE-197
1,2,4,5-Tetrabromo-3-(2-bromophenoxy)benzene
1,2,3,4,5-Pentabromo-6-(3,5-dibromophenoxy)benzene
1,2,3,4,5-Pentabromo-6-(2,5-dibromophenoxy)benzene
3,3',4,4',5-Pentabromo-1,1'-biphenyl
Diphosphoric acid, zinc salt, compd. with 1,3,5-triazine-2,4,6-triamine (1:1:2)
3,3'-[(2-Methylpropyl)phosphoryl]di(propan-1-ol)
2,2'-[(1-Methylethylidene)bis[(dibromo-4,1-phenylene)oxymethylene]]bis[oxirane]-4,
2,4-(or 2,6)-Dibromophenol, homopolymer
2,3',4',6-Tetrabromodiphenyl Ether
2,2',4,5'-Tetrabromodiphenyl Ether
BDE-35
BDE-42
BDE-61
BDE-67
BDE-88
BDE-94
BDE-112
BDE-121
BDE-141
BDE-146
BDE-165
BDE-170
BDE-201
1,2,3-Tribromo-4-(3-bromophenoxy)benzene
Propanoic acid, 2-bromo-, methyl ester
1,2-Benzenedicarboxylic acid, 3,4,5,6-tetrabromo-, sodium salt (1:2)
Molybdenum zinc oxide
1,3,5,7-Tetrabromocyclooctane
Phenyl propan-2-yl hydrogen phosphate
Tribromotrichlorocyclohexane
Tetrabromo trichloromethyl benzene

Query	CAS
353-59-3 OR Methane, bromochlorodifluoro- OR bromochlorodifluoromethane	353593
593-85-1 OR Guanidine carbonate	593851
124-73-2 OR 1,2-Dibromotetrafluoroethane	124732
75-25-2 OR Bromoform	75252
127-09-3 OR Sodium acetate	127093
2314-97-8 OR Trifluoroiodomethane	2314978
141-83-3 OR Guanidine carboxamide	141833
106-41-2 OR 4-Bromophenol	106412
Antimony & Antimony Compounds	NOCAS_872414
2039-82-9 OR 4-Bromostyrene	2039829
57-13-6 OR Urea	57136
1332-21-4 OR Asbestos	1332214
756-13-8 OR Perfluoro-2-methyl-3-pentanone	756138
83-86-3 OR myo-Inositol hexakisphosphate	83863
1336-36-3 OR Polychlorinated biphenyls	1336363
9002-86-2 OR Polyvinyl chloride	9002862
75-63-8 OR Bromotrifluoromethane	75638
14925-39-4 OR 2-Bromopropenal	14925394
2039-86-3 OR 3-Bromostyrene	2039863
2809-21-4 OR Etidronic acid	2809214
1345-04-6 OR Antimony trisulfide colloid OR antimony trisulfide	1345046
69012-64-2 OR Fumes, silica	69012642
12027-06-4 OR Ammonium iodide	12027064
65997-17-3 OR Fiberglass	65997173
96-32-2 OR Methyl bromoacetate	96322
35656-51-0 OR polychlorodibenzo-4-dioxin	35656510
593-60-2 OR Vinyl bromide	593602
335-67-1 OR Perfluorooctanoic acid	335671
7631-90-5 OR Sodium bisulfite	7631905
95-56-7 OR 2-Bromophenol	95567
2623-87-2 OR 4-Bromobutyric acid	2623872
79-28-7 OR Ethene, 1,1,2,2-tetrabromo-	79287
13826-83-0 OR Ammonium fluoroborate	13826830
69248-12-0 OR Tetrakis(hydroxymethyl)phosphanium iodide	69248120
102-71-6 OR Triethanolamine	102716
101-84-8 OR Diphenyl oxide OR phenyl ether	101848
598-72-1 OR 2-Bromopropionic acid	598721
1344-09-8 OR Sodium silicate	1344098
762-04-9 OR Diethyl phosphite	762049
7440-70-2 OR Calcium	7440702
591-20-8 OR Phenol, 3-bromo- OR 3-bromophenol	591208
82001-21-6 OR Bis[(pentabromophenyl)methyl] 3,4,5,6-tetrabromobenzene-1,2-dicarb	82001216
2097144-46-0 OR Hexacosane, chloro derivs.	2097144460

7789-82-4 OR Calcium molybdate(VI)	7789824
37419-42-4 OR 3,3',5,5'-Tetrabromobisphenol A bispropionate	37419424
1373346-90-7 OR dimethyl {[(4,6-dichloro-1,3,5-triazin-2-yl)oxy]methyl}phosphonate	1373346907
57829-89-7 OR 1-(2,3-dibromopropyl)-3,5-di(prop-2-en-1-yl)-1,3,5-triazinane-2,4,6-trio	57829897
75795-16-3 OR 1,3-bis(2,3-dibromopropyl)-5-(prop-2-en-1-yl)-1,3,5-triazinane-2,4,6-tri	75795163
27858-07-7 OR Octabromobiphenyl	27858077
139638-58-7 OR End capped and partially end capped brominated epoxy oligomer	139638587
12251-53-5 OR Aluminium sodium tetrahydroxide	12251535
70715-06-9 OR Phosphonic acid, P-methyl-, dimethyl ester, polymer with oxirane and p	70715069
880647-20-1 OR Polyphosphoric acids, compds. with pyrolyzed melamine	880647201
14957-65-4 OR 4,4',6,6'-Tetrabromo-2,2'-biphenyldiol	14957654
27568-90-7 OR Ethanol, 2-bromo-, phosphate (3:1)	27568907
446255-54-5 OR 2,2',3,4,4',5,6,6'-Octabromodiphenyl ether	446255545
51892-26-3 OR 2,4-Dichlorodiphenyl ether	51892263
12125-02-9 OR Ammonium chloride	12125029
756-79-6 OR Dimethyl methylphosphonate	756796
59080-41-0 OR 2,3,5,6,2',3',5',6'-Octabromobiphenyl	59080410
67774-32-7 OR Firemaster FF-1	67774327
55566-30-8 OR Tetrakis(hydroxymethyl)phosphonium sulfate OR tetramethylolphosph	55566308
78-42-2 OR Tris(2-ethylhexyl) phosphate OR tris(2-ethylhexyl)phosphate	78422
64532-95-2 OR Tris[2-(propan-2-yl)phenyl] phosphate	64532952
69278-59-7 OR 1,1'-Biphenyl, 2,2',3,4',5',6-hexabromo- OR 2,2',3,4',5',6-hexabromobip	69278597
123-77-3 OR Azodicarbonamide OR 1,1-azobisformamide	123773
101-02-0 OR Triphenyl phosphite	101020
140-08-9 OR Tris(2-chloroethyl) phosphite	140089
68952-35-2 OR Tar acids, cresylic, Ph phosphates	68952352
76025-08-6 OR Bis(2-chloro-1-methylethyl) 2-chloropropyl phosphate	76025086
51930-04-2 OR Benzene, 1,3-dibromo-2-phenoxy-	51930042
24937-78-8 OR Vinyl acetate ethylene copolymer OR ethylenevinylacetate copolymer	24937788
7727-43-7 OR Barium sulfate	7727437
24623-77-6 OR Aluminium hydroxide oxide OR aluminum oxide hydroxide	24623776
3072-84-2 OR 2,2'-[(1-Methylethylidene)bis[(2,6-dibromo-4,1-phenylene)oxymethylen	3072842
31107-44-5 OR 1,2,3,4,6,7,8,9,10,10,11,11-dodecachloro-1,4,4a,5a,6,9,9a,9b-octahydr	31107445
39635-79-5 OR 4,4'-Sulphonylbis[2,6-dibromophenol]	39635795
3194-57-8 OR 1,2,5,6-Tetrabromocyclooctane	3194578
12767-90-7 OR Zinc borate (ZnO 3 2B2O3)	12767907
147217-79-6 OR 1,3-Dibromo-5-(3-bromophenoxy)benzene	147217796
19186-97-1 OR Tris(tribromoneopentyl)phosphate	19186971
25713-60-4 OR 2,4,6-Tris-(2,4,6-tribromophenoxy)-1,3,5-triazine OR 2,4,6-tris(2,4,6-trib	25713604
608-33-3 OR Phenol, 2,6-dibromo- OR 2,6-dibromophenol	608333
18755-43-6 OR Dimethyl propylphosphonate	18755436
16400-50-3 OR 1,1'-Biphenyl, 3,3',5,5'-tetrabromo-	16400503
85049-26-9 OR Alkanes, C16-35, chloro	85049269
225789-38-8 OR Aluminum diethylphosphinate	225789388
71342-77-3 OR Phenol, 4,4'-(1-methylethylidene)bis[2,6-dibromo-, polymer with carbo	71342773
134237-50-6 OR (+/-)-α-Hexabromocyclododecane	134237506
147217-73-0 OR BDE-19	147217730

218304-36-0 OR BDE-24	218304360
446254-23-5 OR BDE-50	446254235
337513-82-3 OR BDE-57	337513823
446254-42-8 OR BDE-74	446254428
446254-50-8 OR BDE-81	446254508
446254-62-2 OR BDE-95	446254622
446254-67-7 OR BDE-103	446254677
446254-82-6 OR BDE-122	446254826
446254-87-1 OR BDE-129	446254871
116995-33-6 OR BDE-147	116995336
446255-07-8 OR BDE-152	446255078
407606-59-1 OR BDE-172	407606591
446255-24-9 OR BDE-178	446255249
63316-33-6 OR Benzenesulfonic acid, 3,3'-sulfonylbis-, potassium salt (1:2)	63316336
16923-95-8 OR Dipotassium hexafluorozirconate(2-)	16923958
60371-14-4 OR Bromkal 70	60371144
99717-56-3 OR 2-bromoallyl 2,4,6-tribromophenyl ethe	99717563
34432-82-1 OR Bis(2,3-dibromopropyl) hydrogen phosphate--ammonia (1/1)	34432821
34621-99-3 OR Tetrakis(1-chloropropan-2-yl) ethane-1,2-diyl bis(phosphate)	34621993
35656-01-0 OR Tris(2-bromo-4-methylphenyl) phosphate	35656010
38521-51-6 OR 1,2,3,4,5-Pentabromo-6-(bromomethyl)benzene	38521516
55818-96-7 OR Tetrakis(hydroxymethyl)phosphanium phosphate acetate (4/1/1)	55818967
79481-22-4 OR Tetrakis(hydroxymethyl)phosphanium naphthalene-2-sulfonate	79481224
11113-50-1 OR Crude natural boric acid	11113501
63747-58-0 OR Poly-(m-phenylene methylphosphonate)	63747580
214216-08-7 OR bis(2,3-dibromopropyl) 3,4,5,6-tetrabromobenzene-1,2-dicarboxylate	214216087
53587-44-3 OR Boric acid (H3BO3), compound with 1,3,5-triazine-2,4,6-triamine	53587443
2097144-45-9 OR Alkanes, C20-24, chloro	2097144459
191680-81-6 OR 1,3-Propanediamine, N1,N1'-1,2-ethanediylbis-, reaction products witl	191680816
91-76-9 OR 6-Phenyl-1,3,5-triazine-2,4-diamine	91769
53563-56-7 OR Diphenyl ether, dibromo-	53563567
10043-35-3 OR Boric acid (H3BO3) OR boric acid	10043353
60044-26-0 OR 3,3',4,4',5,5'-Hexabromobiphenyl	60044260
512-56-1 OR Trimethyl phosphate	512561
67888-97-5 OR 2,4,5,3',4'-Pentabromobiphenyl	67888975
115-86-6 OR Triphenyl phosphate	115866
79481-21-3 OR Phosphonium, tetrakis(hydroxymethyl)-, 1-naphthalenesulfonate (salt)	79481213
87-82-1 OR Hexabromobenzene	87821
7723-14-0 OR Phosphorus	7723140
37853-59-1 OR 1,2-Bis(2,4,6-tribromophenoxy)ethane	37853591
80-43-3 OR Dicumyl peroxide	80433
1241-94-7 OR 2-Ethylhexyl diphenyl phosphate OR 2-ethylhexyldiphenylphosphate	1241947
79-94-7 OR 3,3',5,5'-Tetrabromobisphenol A OR tetrabromobisphenol A	79947
79-27-6 OR 1,1,2,2-Tetrabromoethane	79276
298-07-7 OR Bis(2-ethylhexyl) phosphate OR di-2-(ethylhexyl)phosphoric acid	298077
25653-16-1 OR Tris(3,5-xylenyl)phosphate	25653161
77098-07-8 OR Tetrabromophthalic acid mixed esters with diethylene glycol and propy	77098078

125997-20-8 OR Phosphoric acid, mixed 3-bromo-2,2-dimethylpropyl and 2-bromoethy	125997208
7631-86-9 OR Silica OR Silicon Dioxide	7631869
7783-20-2 OR Ammonium sulfate	7783202
63449-39-8 OR Chlorinated paraffins OR chlorowax 500C	63449398
13701-59-2 OR Barium metaborate	13701592
12280-03-4 OR Disodium octaborate tetrahydrate	12280034
598-02-7 OR Diethyl hydrogen phosphate OR diethyl phosphate	598027
838-85-7 OR Diphenyl phosphate	838857
84-15-1 OR o-Terphenyl	84151
1770-80-5 OR Dibutyl chlorendate	1770805
615-58-7 OR 2,4-Dibromophenol	615587
7789-79-9 OR Calcium phosphinate	7789799
147217-77-4 OR 2,4-Dibromo-1-(3-bromophenoxy)benzene	147217774
92-86-4 OR 1,1'-Biphenyl, 4,4'-dibromo- OR 4,4'-dibromobiphenyl	92864
607-99-8 OR 2,4,6-Tribromoanisole	607998
1754-47-8 OR Phosphonic acid, P-phenyl-, dioctyl ester OR dioctylphenylphosphonate	1754478
2752-95-6 OR Butyl diphenyl phosphate	2752956
7580-37-2 OR Phosphonium, tetrakis(hydroxymethyl)-, acetate (salt) OR tetramethylol	7580372
52221-67-7 OR Phosphonium, tetrakis(hydroxymethyl)-, ethanedioate (2:1) (salt) OR te	52221677
53506-00-6 OR Propanenitrile, 3,3',3'',3'''-[(2,3,5,6-tetramethyl-1,4-phenylene)bis(metl	53506006
61090-89-9 OR 2,4,8,10-Tetraoxa-3,9-diphosphaspiro[5.5]undecane, 3,9-bis[3-bromo-2	61090899
446255-30-7 OR 1,2,3,5-Tetrabromo-4-(3,4,5-tribromophenoxy)benzene	446255307
115-87-7 OR Bis(p-tert-butylphenyl) phenyl phosphate	115877
109678-33-3 OR 1-Propanol, 3,3'-oxybis[2,2-bis(bromomethyl)-	109678333
2528-39-4 OR Phosphoric acid, trihexyl ester	2528394
57011-47-9 OR Pentabromophenyl benzoate	57011479
38463-82-0 OR 1,3,5-Tribromo-2-(2,3-dibromophenoxy)benzene	38463820
37853-61-5 OR Tetrabromobisphenol A dimethyl ether	37853615
108171-26-2 OR C10-12 chloroalkanes	108171262
13308-51-5 OR Boron phosphate (B(PO4))	13308515
135229-48-0 OR Brominated epoxy resin end-capped with tribromophenol	135229480
56974-60-8 OR Dimelamine monophosphate	56974608
35854-94-5 OR 2,2',4,4',6,6'-Hexabromodiphenyl Ether	35854945
337513-56-1 OR BDE-29	337513561
337513-54-9 OR BDE-38	337513549
446254-29-1 OR BDE-58	446254291
446254-34-8 OR BDE-63	446254348
446254-52-0 OR BDE-84	446254520
446254-57-5 OR 1,2,5-Tribromo-3-(2,4-dibromophenoxy)benzene	446254575
446254-72-4 OR BDE-109	446254724
446254-77-9 OR BDE-114	446254779
446254-94-0 OR BDE-136	446254940
446254-99-5 OR BDE-143	446254995
446255-13-6 OR BDE-162	446255136
446255-17-0 OR BDE-167	446255170
446255-34-1 OR BDE-193	446255341
66034-17-1 OR Diphosphoric acid, compd. with piperazine (1:1)	66034171

40039-93-8 OR 3,5,3',5'-Tetrabromobisphenol A, epichlorohydrin polymer	40039938
13767-32-3 OR Zinc molybdate(VI) (ZnMoO4)	13767323
12040-58-3 OR Calcium borate	12040583
12079-58-2 OR Bromine--methane (1/16)	12079582
67889-00-3 OR 2,3,4,5,2',3',4',5'-Octabromobiphenyl	67889003
59080-37-4 OR 2,2',5,5'-Tetrabromobiphenyl	59080374
26248-87-3 OR Tris(chloropropyl)phosphate	26248873
138257-18-8 OR (-)-beta-Hexabromocyclododecane	138257188
1951-97-9 OR Piperazine, phosphate (1:?)	1951979
27581-13-1 OR 2,3,4,5-Tetrabromobenzoic Acid	27581131
855993-01-0 OR (rel)-(1R,2S,3S,4S)-1,2,3,9-tetrabromo-1,2,3,4-tetrahydro-1,4-methano	855993010
135821-74-8 OR anti-Dechlorane Plus	135821748
75019-90-8 OR Tetrakis(hydroxymethyl)phosphonium p-toluenesulfonate	75019908
1195978-93-8 OR Benzene, ethenyl-, polymer with 1,3-butadiene, brominated	1195978938
155613-93-7 OR 1H-Indene, 2,3-dihydro-1,1,3-trimethyl-3-phenyl-, octabromo deriv.	155613937
119264-60-7 OR 2,2',3,3',4,5',6,6'-Octabromobiphenyl	119264607
115-28-6 OR Chlorendic acid	115286
99-30-9 OR Dicloran	99309
77-47-4 OR Hexachlorocyclopentadiene	77474
6161-81-5 OR Dioctyl phenyl phosphate	6161815
108-46-3 OR Resorcinol	108463
69278-61-1 OR 1,1'-Biphenyl, 2,2',3,3',4,4',5,6'-octabromo- OR octabromobiphenyl	69278611
791-28-6 OR Triphenylphosphine oxide	791286
32534-81-9 OR Pentabromodiphenyl ether	32534819
3066-70-4 OR 2,3-Dibromopropylmethacrylate OR poly(2,3-dibromopropylmethacrylat	3066704
115-27-5 OR Chlorendic anhydride	115275
115-77-5 OR Pentaerythritol	115775
92-06-8 OR 1,1':3',1''-Terphenyl	92068
2781-11-5 OR Diethyl (N,N-bis(2-hydroxyethyl)amino)methanephosphonate OR Fyrol 6	2781115
25327-89-3 OR Tetrabromobisphenol A diallyl ether	25327893
68527-01-5 OR Bromo chloro C12-30 a-alkenes	68527015
1303-96-4 OR Borax (B4Na2O7.10H2O) OR borax	1303964
1330-43-4 OR Sodium tetraborate (Na2B4O7) OR sodium borate	1330434
7646-85-7 OR Zinc chloride	7646857
21645-51-2 OR Aluminum hydroxide	21645512
813-76-3 OR diethylphosphinic acid	813763
85535-85-9 OR Cercelor S 52	85535859
1344-28-1 OR Alumina OR Aluminum Oxide	1344281
875-73-0 OR Tribromostyrene	875730
52434-59-0 OR 1,3,5-Triazine, 2,4,6-tris(2,3-dibromopropoxy)-	52434590
327185-11-5 OR Benzene, 1,2,3-tribromo-4-(2,3-dibromophenoxy)-	327185115
7782-42-5 OR Graphite	7782425
84852-53-9 OR 1,1'-Ethane-1,2-diylbis(pentabromobenzene) OR decabromodiphenyl et	84852539
995-32-4 OR Tetraethyl ethylenebisphosphonate	995324
36313-15-2 OR 2,3,4,5-Tetrabromophenol	36313152
814-29-9 OR Tributylphosphine oxide	814299
59789-51-4 OR 1H-Pyrrole-2,5-dione, 1-(2,4,6-tribromophenyl)-	59789514

68928-80-3 OR Diphenyl ether, heptabromo derivative	68928803
83242-23-3 OR 2-tert-Butylphenyl diphenyl phosphate OR tert-butylphenyl diphenyl ph	83242233
337513-53-8 OR 1,3-Dibromo-2-(3-bromophenoxy)benzene	337513538
405237-85-6 OR 1,2,3,4-Tetrabromo-5-(3,4-dibromophenoxy)benzene	405237856
106232-85-3 OR Alkanes, C18-20, chloro	106232853
4351-70-6 OR Phosphonic acid, P-[1-[[(2-chloroethoxy)(2-chloroethyl)phosphinyl]oxy]e	4351706
Medium-chain chlorinated paraffins (C14-17)	NOCAS_872423
68937-40-6 OR Triphenyl phosphates tert-butylated	68937406
84082-38-2 OR Alkanes, C10-21, chloro	84082382
10310-38-0 OR Ethylenebis[tris(2-cyanoethyl)phosphonium bromide]	10310380
15652-65-0 OR Tetrakis(hydroxymethyl)phosphonium tetraphenylborate-tetraacetate	15652650
18300-04-4 OR 1,2-Dibromo-4,5,6,7,8,8-hexachloro-2,3,3a,4,7,7a-hexahydro-4,7-meth	18300044
7784-22-7 OR Aluminum hypophosphite	7784227
134237-52-8 OR (+/-)-gamma-Hexabromocyclododecane	134237528
147217-72-9 OR BDE-6	147217729
337513-67-4 OR BDE-21	337513674
446254-21-3 OR BDE-45	446254213
446254-25-7 OR BDE-53	446254257
446254-39-3 OR BDE-70	446254393
446254-43-9 OR BDE-76	446254439
446254-64-4 OR BDE-97	446254644
373594-78-6 OR BDE-105	373594786
446254-84-8 OR BDE-124	446254848
446254-90-6 OR BDE-132	446254906
446255-04-5 OR BDE-149	446255045
446255-09-0 OR BDE-158	446255090
446255-22-7 OR BDE-175	446255227
442690-45-1 OR BDE-182	442690451
1093632-34-8 OR 1,3,5,7,9,11-Hexabromocyclododecane	1093632348
1047637-37-5 OR 2,2-Bis(chloromethyl)-1,3-propanediyl tetrakis(1-chloro-2-propanyl) I	1047637375
1401974-24-0 OR Alkanes, C22-30-branched and linear, chloro	1401974240
26446-73-1 OR Phosphoric acid, bis(methylphenyl) phenyl ester	26446731
13530-50-2 OR Aluminum phosphate (Al(H2PO4)3) OR aluminum phosphate	13530502
39686-78-7 OR Tetrakis(hydroxymethyl)phosphanium 2-hydroxypropanoate	39686787
39734-92-4 OR Tetrakis(hydroxymethyl)phosphanium 3-carboxy-3-hydroxypropanoate	39734924
77226-90-5 OR Carbonic acid, diphenyl ester, polymer with diphenyl P-methylphosphoi	77226905
114955-21-4 OR diethyl (4,6-dichloro-1,3,5-triazin-2-yl)phosphonate	114955214
57137-10-7 OR Poly(tribromostyrene)	57137107
71245-27-7 OR Dechloran 604	71245277
2097144-43-7 OR Alkanes, C20-28, chloro	2097144437
88497-56-7 OR Benzene, ethenyl-, homopolymer, brominated	88497567
13560-92-4 OR 1,4:5,8:9,10-Trimethanoanthracene, 1,2,3,4,5,6,7,8,12,12,13,13-dodeca	13560924
97553-43-0 OR Paraffins (petroleum), normal C>10, chloro	97553430
42429-89-0 OR 1,1':3',1''-Terphenyl, 2,2',2'',3,3'',4,4',4'',5,5',5'',6,6',6''-tetradecachloro-	42429890
36711-31-6 OR Bis(2,3-dibromopropyl) phosphate, magnesium salt OR bis(2,3-dibromo	36711316
60763-39-5 OR Isopropyl diphenyl phosphate	60763395
79-95-8 OR 2,2',6,6'-Tetrachlorobisphenol A OR tetrachlorodian	79958

67888-99-7 OR 2,3',4,4'5,5'-Hexabromobiphenyl	67888997
69278-62-2 OR 2,2',3,3',4,4',5,5',6-Nonabromo-1,1'-biphenyl	69278622
126-73-8 OR Tributyl phosphate	126738
63936-56-1 OR Nonabromodiphenyl ether OR 2,2',3,3',4,4',5,5',6-nonabromodiphenyl e	63936561
26444-49-5 OR Cresyl diphenyl phosphate OR diphenylcresyl phosphate	26444495
36355-01-8 OR Hexabromobiphenyl	36355018
29761-21-5 OR Isodecyl diphenyl phosphate OR santicizer 148	29761215
87-83-2 OR Pentabromotoluene OR 2,3,4,5,6-pentabromotoluene	87832
3290-92-4 OR Trimethylolpropane trimethacrylate	3290924
6145-73-9 OR Tris(2-chloropropyl) phosphate OR Fyrol PCF	6145739
65652-41-7 OR Bis(tert-butylphenyl)phenyl phosphate	65652417
85422-92-0 OR Chlorinated paraffin oils OR chloroparaffin 40G	85422920
5436-43-1 OR 2,2',4,4'-Tetrabromodiphenyl ether	5436431
21850-44-2 OR Tetrabromobisphenol A-bis(2,3-dibromopropyl ether)	21850442
1773-89-3 OR Chlorendate dimethyl	1773893
24655-84-3 OR Tetrakis(hydroxymethyl)phosphonium OR Pyroset TKP	24655843
3278-89-5 OR 1,3,5-Tribromo-2-(prop-2-en-1-yloxy)benzene	3278895
437701-79-6 OR BDE-207 OR 2,2',3,3',4,4',5,6,6'-nonabromodiphenyl ether	437701796
14657-64-8 OR 3-[Hydroxy(phenyl)phosphoryl]propanoic acid	14657648
678970-16-6 OR (+)-beta-Hexabromocyclododecane	678970166
1302-42-7 OR Sodium aluminate	1302427
9002-85-1 OR Polyvinylidene chloride	9002851
78-33-1 OR Tris(4-tert-butylphenyl) phosphate	78331
626-39-1 OR 1,3,5-Tribromobenzene	626391
207122-15-4 OR 2,2',4,4',5,6'-Hexabromodiphenyl ether	207122154
2528-38-3 OR Tripentyl phosphate	2528383
497107-13-8 OR 1,1'-[Oxybis(methylene)]bis(pentabromobenzene)	497107138
645-92-1 OR 1,3,5-Triazin-2(1H)-one, 4,6-diamino- OR ammeline	645921
36678-45-2 OR 2-Butene, 1,1,2,3,4,4-hexabromo-	36678452
37640-57-6 OR 1,3,5-Triazine-2,4,6(1H,3H,5H)-trione, compd. with 1,3,5-triazine-2,4,6-	37640576
103173-66-6 OR 1,1'-Oxybis(3,5-dibromobenzene)	103173666
446255-39-6 OR BDE-196	446255396
446254-96-2 OR 1,2,3,5-Tetrabromo-4-(2,4-dibromophenoxy)benzene	446254962
417727-71-0 OR 1,2,4-Tribromo-5-(3,5-dibromophenoxy)benzene	417727710
1084889-51-9 OR 4,5,6,7-Tetrabromo-1,1,3-trimethyl-3-(2,3,4,5-tetrabromophenyl)-2,3	1084889519
1852481-27-6 OR 2,3,4,5,6,8-Hexachlorodecane	1852481276
7415-86-3 OR 1,2-Benzenedicarboxylic acid, 1,2-bis(2,3-dibromopropyl) ester	7415863
40703-79-5 OR 4,7-Methano-1H-isoindole-1,3(2H)-dione, 5,6-dibromohexahydro-2-phe	40703795
85536-22-7 OR Alkanes, C12-14, chloro	85536227
63316-43-8 OR Potassium 3-(phenylsulfonyl)benzenesulfonate	63316438
446254-17-7 OR BDE-34	446254177
337513-68-5 OR BDE-41	337513685
446254-31-5 OR BDE-60	446254315
446254-36-0 OR BDE-65	446254360
446254-54-2 OR BDE-87	446254542
446254-59-7 OR BDE-92	446254597
446254-74-6 OR BDE-111	446254746

446254-79-1 OR BDE-117	446254791
243982-83-4 OR BDE-140	243982834
446255-01-2 OR BDE-145	446255012
446255-15-8 OR BDE-164	446255158
446255-18-1 OR BDE-169	446255181
446255-46-5 OR BDE-200	446255465
446255-42-1 OR BDE-198	446255421
63387-28-0 OR 1,2,3,4,5-Pentabromo-6-(2,3,4,5-tetrabromophenoxy)benzene	63387280
33798-02-6 OR Phenol, 4,4'-(1-methylethylidene)bis[2,6-dibromo-, 1,1'-diacetate	33798026
33908-66-6 OR Sodium hexahydroxyantimonate(1-)	33908666
2167063-57-0 OR 1,2,3-Tribromo-4-[(prop-2-en-1-yl)oxy]benzene	2167063570
36885-49-1 OR Ethenyl dihydrogen phosphate	36885491
56890-89-2 OR 1,2,3,4,7,7-Hexachloro-5-(2,4,6-tribromophenyl)bicyclo[2.2.1]hept-2-er	56890892
68333-79-9 OR Ammonium polyphosphates	68333799
72236-72-7 OR Bis(1,3-dichloropropan-2-yl) hydrogen phosphate	72236727
2097144-48-2 OR Octadecane, chloro derivs.	2097144482
68441-62-3 OR 2-Butyne-1,4-diol, polymer with 2-(chloromethyl)oxirane, brominated,	68441623
32844-27-2 OR Carbonic dichloride, polymer with 4,4'-(1-methylethylidene)bis[2,6-dibr	32844272
678970-15-5 OR (-)-alpha-Hexabromocyclododecane	678970155
61368-34-1 OR Tribromostyrene (mixed isomers)	61368341
125512-87-0 OR Hexabromohexane	125512870
19569-21-2 OR Huntite	19569212
71011-12-6 OR Alkanes, C12-13, chloro	71011126
85535-84-8 OR C10-13 chloro alkanes	85535848
148993-99-1 OR Benzene, ethenyl-, ar-bromo derivs., homopolymers	148993991
284685-45-6 OR Phosphinic acid, P,P-diethyl-, zinc salt (2:1)	284685456
119264-63-0 OR 2,2',3,3',4,5,5',6,6'-Nonobromobiphenyl	119264630
13765-95-2 OR Zirconium phosphate	13765952
1502-47-2 OR Melem	1502472
31710-32-4 OR p-Tetradecachloroterphenyl	31710324
96-69-5 OR 4,4'-Thiobis(6-tert-butyl-m-cresol) OR 4,4'-thiobis(6-tert-butyl-3-cresol)	96695
1330-78-5 OR Tris(methylphenyl) phosphate OR IMOL S-140	1330785
13472-45-2 OR Sodium tungsten oxide (Na2WO4) OR sodium tungstate(VI)	13472452
1309-64-4 OR Antimony trioxide	1309644
92-66-0 OR 4-Bromobiphenyl	92660
87-84-3 OR Pentabromochlorocyclohexane	87843
563-04-2 OR Tri-m-tolyl phosphate	563042
301-13-3 OR Tris(2-ethylhexyl) phosphite OR tri-(2-ethylhexyl)phosphite	301133
839-90-7 OR Tris(2-hydroxyethyl) isocyanurate	839907
3194-55-6 OR 1,2,5,6,9,10-Hexabromocyclododecane OR hexabromocyclododecane	3194556
68937-41-7 OR Triphenyl phosphates isopropylated	68937417
68631-49-2 OR 2,2',4,4',5,5'-Hexabromodiphenyl ether OR hexabrominated diphenyl e	68631492
4162-45-2 OR Tetrabromobisphenol A bis(2-hydroxyethyl) ether	4162452
32577-34-7 OR Benzene, pentabromo(2,3-dibromopropoxy)-	32577347
10025-91-9 OR Antimony trichloride	10025919
189084-59-1 OR 3,4-Dibromodiphenyl ether	189084591
546-93-0 OR Magnesium carbonate (1:1) OR magnesium carbonate	546930

1309-42-8 OR Magnesium hydroxide	1309428
1318-23-6 OR Boehmite OR aluminum oxide hydroxide	1318236
132-29-6 OR 2-Biphenylyl diphenyl phosphate	132296
182346-21-0 OR 2,2',3,4,4'-Pentabromodiphenyl ether OR pentabromodiphenyl ether	182346210
1837-91-8 OR Benzene hexabromide	1837918
189084-64-8 OR 2,2',4,4',6-Pentabromodiphenyl ether OR pentabromodiphenyl ether	189084648
41318-75-6 OR 2,4,4'-Tribromodiphenyl ether OR tribromodiphenyl ether 28	41318756
513-08-6 OR Tripropyl phosphate	513086
545-55-1 OR Tri(aziridin-1-yl)phosphine oxide OR Triethylenephosphoramide	545551
437701-78-5 OR 2,2',3,3',4,5,5',6,6'-nonabromodiphenyl ether	437701785
1017-56-7 OR 1,3,5-Triazine-2,4,6-triyltriiminotrimethanol	1017567
189084-57-9 OR 1,3-Dibromo-2-(2,4-dibromophenoxy)benzene	189084579
4090-51-1 OR 1,3,2-Dioxaphosphorinane, 2,2'-oxybis[5,5-dimethyl-, 2,2'-disulfide	4090511
70776-17-9 OR 1,3,5-Triazine-2,4,6-triamine, compd. with 3,9-dihydroxy-2,4,8,10-tetra	70776179
446254-80-4 OR 1,2,4-Tribromo-5-(3,4-dibromophenoxy)benzene	446254804
77102-82-0 OR PBB 077 OR 3,3',4,4'-tetrabromobiphenyl	77102820
446255-38-5 OR PBDE 195	446255385
446255-56-7 OR PBDE 205	446255567
82865-89-2 OR 2,2',3,3',4,4'-Hexabromo-1,1'-biphenyl	82865892
20217-01-0 OR 2,4-Dibromophenyl glycidyl ether	20217010
84776-07-8 OR Alkanes, C16-27, chloro	84776078
147217-75-2 OR 2,2',4-Tribromodiphenyl ether	147217752
12411-64-2 OR Tetraammonium octamolybdate OR ammonium molybdate	12411642
218768-84-4 OR Melapur 200	218768844
119264-62-9 OR 2,2',3,3',4,4',5,6,6'-Nonabromo-1,1'-biphenyl	119264629
446255-26-1 OR BDE-180	446255261
407606-55-7 OR BDE-18	407606557
446254-16-6 OR BDE-23	446254166
337513-55-0 OR BDE-48	337513550
446254-28-0 OR BDE-56	446254280
446254-41-7 OR BDE-73	446254417
446254-48-4 OR BDE-79	446254484
446254-66-6 OR BDE-102	446254666
446254-70-2 OR BDE-107	446254702
446254-86-0 OR BDE-127	446254860
446254-92-8 OR BDE-134	446254928
446255-06-7 OR BDE-151	446255067
446255-11-4 OR BDE-160	446255114
446255-23-8 OR BDE-177	446255238
446255-28-3 OR BDE-187	446255283
42597-49-9 OR 1,2-Benzenedicarboxylic acid, 3,4,5,6-tetrabromo-, 1-butyl ester	42597499
59794-15-9 OR Calcium borate silicate	59794159
36483-57-5 OR Tribromoneopentyl alcohol	36483575
12777-87-6 OR Sulfuric acid--methane (1/1)	12777876
13560-91-3 OR 1,2,3,4,5,6,7,8,10,10,11,11-Dodecachloro-4,4a,4b,5,8,8a,9,9a-octahydr	13560913
27104-30-9 OR Tetrakis(hydroxymethyl)phosphanium carbamimidate--hydrogen chlori	27104309
79596-31-9 OR 1~2~,1~3~,1~4~,1~5~,1~6~,2~3~,2~4~,3~2~,3~3~,3~4~,3~5~,3~6~-Dod	79596319

138257-19-9 OR (+)-alpha-Hexabromocyclododecane	138257199
70156-79-5 OR 1,1'-sulfonylbis(3,5-dibromo-4-methoxybenzene)	70156795
855992-98-2 OR (rel)-(1R,2R,3S,4S)-1,2,3,9-tetrabromo-1,2,3,4-tetrahydro-1,4-methan	855992982
135821-03-3 OR syn-Dechlorane Plus	135821033
5324-12-9 OR 2,3-Dibromopropylphosphate	5324129
73083-23-5 OR Phosphonium, tetrakis(hydroxymethyl)-, salt with cellulose carboxymet	73083235
171091-06-8 OR Benzene, ethenyl-, ar-bromo derivs., polymers with propene, graft	171091068
39569-21-6 OR 2,3,4,5-Tetrabromo-6-chlorotoluene	39569216
10043-52-4 OR Calcium chloride	10043524
868-85-9 OR Dimethyl phosphonate OR dimethyl hydrogen phosphite	868859
59536-65-1 OR Polybrominated biphenyls (PBB) OR Firemaster BP-6	59536651
124-64-1 OR Tetramethylolphosphonium chloride	124641
115-96-8 OR Tris(2-chloroethyl) phosphate OR tris(chloroethyl)phosphate	115968
126-72-7 OR Tris(2,3-dibromopropyl) phosphate OR tris(2,3-dibromopropyl)phosphate	126727
78-51-3 OR Tris(2-butoxyethyl) phosphate	78513
67888-96-4 OR 1,1'-Biphenyl, 2,2',4,5,5'-pentabromo-	67888964
7773-06-0 OR Ammonium sulfamate OR sulfamic acid	7773060
147217-78-5 OR 2',3,4-Tribromodiphenyl ether	147217785
2113-57-7 OR 3-Bromobiphenyl	2113577
26780-96-1 OR 1,2-Dihydro-2,2,4-trimethylquinoline, polymer OR poly(2,2,4-trimethyl-	26780961
813-78-5 OR Dimethyl phosphate	813785
13674-84-5 OR Tris(2-chloroisopropyl)phosphate OR tri-(2-chloroisopropyl)phosphate	13674845
33125-86-9 OR Phosphoric acid, 1,2-ethanediyl tetrakis(2-chloroethyl) ester	33125869
68920-70-7 OR Alkanes, C6-18, chloro	68920707
68952-33-0 OR Tar acids, cresylic, C8-rich, phosphates	68952330
542-02-9 OR 6-Methyl-1,3,5-triazine-2,4-diamine	542029
7722-76-1 OR Ammonium phosphate	7722761
7786-30-3 OR Magnesium chloride	7786303
117948-63-7 OR Benzene, 1,2,3,5-tetrabromo-4-(2,4,6-tribromophenoxy)-	117948637
42757-55-1 OR 1,1'-Sulfonylbis[3,5-dibromo-4-(2,3-dibromopropoxy)benzene]	42757551
3486-35-9 OR Zinc carbonate	3486359
12007-58-8 OR Diammonium tetraborate OR ammonium borate	12007588
147-82-0 OR 2,4,6-Tribromoaniline	147820
78-32-0 OR Tris(4-methylphenyl) phosphate OR triparacresyl phosphate	78320
35109-60-5 OR 1,3,5-Tribromo-2-(2,3-dibromopropoxy)benzene	35109605
366791-32-4 OR 1,2,3-Tribromo-5-(3,4-dibromophenoxy)benzene	366791324
147217-71-8 OR 1-Bromo-2-(4-bromophenoxy)benzene	147217718
3555-11-1 OR Allyl pentabromophenyl ether	3555111
12036-37-2 OR Tin zinc oxide (SnZnO3)	12036372
116995-32-5 OR 1,2,4,5-Tetrabromo-3-(2,4,6-tribromophenoxy)benzene	116995325
60044-25-9 OR PBB 053	60044259
26967-76-0 OR Tris(isopropylphenyl) phosphate	26967760
12058-66-1 OR Disodium stannate	12058661
12027-96-2 OR Tin zinc hydroxide (ZnSn(OH)6)	12027962
2528-36-1 OR Dibutyl phenyl phosphate OR dibutyl phenylphosphate	2528361
189084-66-0 OR 2,3',4,4',6-Pentabromodiphenyl Ether	189084660
147217-81-0 OR BDE-37	147217810

446254-19-9 OR BDE-43	446254199
446254-33-7 OR BDE-62	446254337
446254-38-2 OR 2,4-Dibromo-1-(3,5-dibromophenoxy)benzene	446254382
446254-51-9 OR BDE-83	446254519
446254-56-4 OR BDE-89	446254564
446254-71-3 OR BDE-108	446254713
446254-76-8 OR BDE-113	446254768
446254-93-9 OR BDE-135	446254939
446254-98-4 OR BDE-142	446254984
446255-12-5 OR BDE-161	446255125
189084-58-0 OR BDE-166	189084580
259087-35-9 OR BDE-189	259087359
67797-09-5 OR BDE-202	67797095
49690-94-0 OR Tribromodiphenyl ether	49690940
1417900-96-9 OR Alkanes, C21-34-branched and linear, chloro	1417900969
2788-11-6 OR Tris(2,4-dibromophenyl) phosphate	2788116
36065-30-2 OR 1,3,5-Tribromo-2-(2,3-dibromo-2-methylpropoxy)benzene	36065302
68955-41-9 OR 1-Bromo-4-chlorodecane	68955419
84282-27-9 OR 2-Bromoethyl 5-bromopentyl 2-chloroethyl phosphate	84282279
9003-08-1 OR 1,3,5-Triazine-2,4,6-triamine, polymer with formaldehyde	9003081
2097144-44-8 OR Slack Wax (petroleum), chloro	2097144448
94334-64-2 OR Carbonic dichloride, polymer with 4,4'-(1-methylethylidene)bis[2,6-dibr	94334642
1025956-65-3 OR 2,4,5,6,7-pentabromo-1,1,3-trimethyl-3-(2,4,6-tribromophenyl)-2,3-(	1025956653
55481-60-2 OR dimethyl 3,4,5,6-tetrabromobenzene-1,2-dicarboxylate	55481602
62588-94-7 OR Tetrakis(hydroxymethyl)phosphonium acetate-phosphate (1:1)	62588947
288260-42-4 OR Alkanes, C22-30, chloro	288260424
64176-42-7 OR Antiblaze 78	64176427
139189-30-3 OR Phosphoric acid, P,P'-1,3-phenylene P,P,P',P'-tetrakis(2,6-dimethylphe	139189303
383905-85-9 OR Polyphosphoric acids, compds. with piperazine	383905859
359406-89-6 OR Phosphonium, tetrakis(hydroxymethyl)-, chloride (1:1), reaction produ	359406896
119264-59-4 OR 1,1'-Biphenyl, 2,2',3,3',4,4',6,6'-octabromo-	119264594
14400-94-3 OR 2,3,4,6-Tetrabromophenol	14400943
5118-79-6 OR s-Triazine, 2,2'-O-phenylene-bis(4,6-diamino-	5118796
108-78-1 OR Melamine	108781
69278-60-0 OR 2,2',3,3',4,4',5-Heptabromobiphenyl	69278600
118-79-6 OR 2,4,6-Tribromophenol	118796
79755-43-4 OR 3,5-Dibromo-2-(2,4-dibromophenoxy)phenol	79755434
83929-69-5 OR 2,2',3,3',5,5',6,6'-Octabromo-4-phenoxy-1,1'-biphenyl	83929695
36483-60-0 OR Hexabromodiphenyl ether	36483600
32588-76-4 OR 1,2-Bis(tetrabromophthalimido)ethane	32588764
56803-37-3 OR tert-Butylphenyl diphenyl phosphate	56803373
28108-99-8 OR Isopropyl phenyl diphenyl phosphate	28108998
2234-13-1 OR 1,2,3,4,5,6,7,8-Octachloronaphthalene OR octachloronaphthalene	2234131
632-79-1 OR 4,5,6,7-Tetrabromo-1,3-Isobenzofurandione OR tetrabromophthalic anhy(	632791
1806-54-8 OR Trioctyl phosphate	1806548
92-94-4 OR 1,1':4',1''-Terphenyl	92944
7783-28-0 OR Diammonium hydrogen phosphate OR ammonium phosphate	7783280

1302-78-9 OR Bentonite	1302789
78-30-8 OR Tri-o-cresyl phosphate	78308
35948-25-5 OR 6H-Dibenzo[c,e][1,2]oxaphosphinine 6-oxide	35948255
41424-36-6 OR 1,3,5-Tribromo-2-methoxy-4-methylbenzene	41424366
155999-95-4 OR 2,4,6-Tribromodiphenyl ether	155999954
10124-31-9 OR Phosphoric acid, ammonium salt (1:x) OR ammonium phosphate	10124319
1314-60-9 OR Antimony pentoxide	1314609
16368-97-1 OR Bis(2-ethylhexyl) phenyl phosphate	16368971
52907-07-0 OR N,N'-(Ethylene)bis[4,5-dibromohexahydro-3,6-methanophthalimide]	52907070
182677-28-7 OR 1,1'-Oxybis(2,3,4-tribromobenzene)	182677287
189084-67-1 OR 1,2,3,4,5-Pentabromo-6-(2,4-dibromophenoxy)benzene	189084671
46438-88-4 OR 1,3-Dibromo-5-phenoxybenzene	46438884
632-58-6 OR Tetrachlorophthalic acid	632586
7760-50-1 OR Magnesium carbonate hydroxide (Mg5(CO3)4(OH)2)	7760501
53461-82-8 OR Diethylene glycol bis[bis(2-chloroethyl)phosphate]	53461828
337513-75-4 OR 1,4-Dibromo-2-(3-bromophenoxy)benzene	337513754
20566-35-2 OR 2-(2-Hydroxyethoxy)ethyl 2-hydroxypropyl 3,4,5,6-tetrabromophthalat	20566352
182677-30-1 OR 2,2',3,4,4',5'-Hexabromodiphenyl Ether	182677301
Long-chain chlorinated paraffins (C18-20)	NOCAS_872422
85535-86-0 OR C18-28 Chloroalkanes	85535860
97659-46-6 OR Alkanes, C10-26, chloro	97659466
1003300-73-9 OR Mixed esters of phosphoric acid with [1,1'-biphenyl]-4,4'-diol and phe	1003300739
15432-85-6 OR Sodium antimonate	15432856
28906-13-0 OR 2,2-Bis(3,5-dibromo-4-hydroxyphenyl)propane-phosgene copolymer	28906130
134237-51-7 OR (+/-)-beta-Hexabromocyclododecane	134237517
51452-87-0 OR BDE-4	51452870
147217-76-3 OR BDE-20	147217763
446254-20-2 OR BDE-44	446254202
446254-24-6 OR BDE-52	446254246
327185-09-1 OR BDE-69	327185091
189084-63-7 OR BDE-75	189084637
446254-63-3 OR BDE-96	446254633
446254-68-8 OR BDE-104	446254688
446254-83-7 OR BDE-123	446254837
446254-89-3 OR BDE-131	446254893
446255-03-4 OR BDE-148	446255034
446255-08-9 OR BDE-157	446255089
446255-21-6 OR BDE-174	446255216
446255-25-0 OR BDE-179	446255250
61262-53-1 OR Benzene, 1,1'-[1,2-ethanediylbis(oxy)]bis[2,3,4,5,6-pentabromo-	61262531
1332-07-6 OR Zinc borate	1332076
919-62-0 OR Tris(3-methylbutyl) phosphate	919620
28205-79-0 OR Ethanol, 2-chloro-, phosphate (3:1), homopolymer	28205790
55205-38-4 OR (Propane-2,2-diyl)bis(2,6-dibromo-4,1-phenylene) diprop-2-enoate	55205384
5940-69-2 OR Tetrakis(hydroxymethyl)phosphanium bromide	5940692
60044-24-8 OR 2,2',4,5-Tetrabromo-1,1'-biphenyl	60044248
68441-46-3 OR 1,3-Butadiene, homopolymer, brominated	68441463

68664-06-2 OR Diphenyl methylphosphonate--4,4'-(propane-2,2-diyl)diphenol (1/1)	68664062
2097144-47-1 OR Octacosane, chloro derivs.	2097144471
121839-52-9 OR 4,4'-(1-Methylethylidene)bisphenol tetrabromo deriv.	121839529
678970-17-7 OR (+)-gamma-Hexabromocyclododecane	678970177
168434-45-5 OR 2,4,6-tribromo-3-(tetrabromopentadecyl)-phenol	168434455
61288-13-9 OR Bromkal 80	61288139
93202-89-2 OR N-2,3-Dibromopropyl-4,5-dibromohexahydrophthalimide	93202892
55818-86-5 OR Fyrquel GT	55818865
184538-58-7 OR Phosphoric acid, triethyl ester, polymer with oxirane and phosphorus (	184538587
25495-98-1 OR Cyclodecane, hexabromo	25495981
36558-41-5 OR Bis(thiopyrophosphoric acid)	36558415
42429-88-9 OR 1,1':2',1''-Terphenyl, 2,2'',3,3',3'',4,4',4'',5,5',5'',6,6',6''-tetradecachloro-	42429889
80-05-7 OR Bisphenol A	80057
2385-85-5 OR Mirex	2385855
1313-27-5 OR Molybdenum trioxide	1313275
85-22-3 OR 2,3,4,5,6-Pentabromoethylbenzene	85223
67733-52-2 OR 1,1'-Biphenyl, 2,2',3,4,4',5,5'-heptabromo-	67733522
67888-98-6 OR 2,2',3,4,4',5-Hexabromobiphenyl	67888986
96-13-9 OR 2,3-Dibromopropanol	96139
78-43-3 OR Tris(2,3-dichloropropyl)phosphate	78433
108171-27-3 OR Chloro C22-26 alkanes	108171273
108-80-5 OR Cyanuric acid	108805
19660-16-3 OR 2,3-Dibromopropyl acrylate	19660163
78-38-6 OR Diethyl ethylphosphonate	78386
117-08-8 OR Tetrachlorophthalic anhydride	117088
78-31-9 OR Diphenyl 4-tolyl phosphate	78319
76649-15-5 OR (2-Chloro-1-methylethyl) bis(2-chloropropyl) phosphate	76649155
5412-25-9 OR Bis(2,3-dibromopropyl) hydrogen phosphate	5412259
13560-89-9 OR Dechlorane Plus	13560899
26040-51-7 OR Bis(2-ethylhexyl) tetrabromophthalate OR bis(2-ethylhexyl) 2,3,4,5-tetr	26040517
61788-76-9 OR Chloroalkanes	61788769
68527-02-6 OR Alkenes, C12-24, chloro	68527026
16919-27-0 OR Dipotassium hexafluorotitanate	16919270
1314-35-8 OR Tungsten trioxide OR tungsten oxide	1314358
1303-86-2 OR Boric oxide OR boron oxide	1303862
12179-04-3 OR Boron sodium oxide pentahydrate	12179043
1305-62-0 OR Calcium hydroxide	1305620
1314-13-2 OR Zinc oxide	1314132
14265-44-2 OR Phosphate OR Phosphates	14265442
5205-93-6 OR N-(3-Dimethylaminopropyl)methacrylamide OR N-(3-(dimethylamino)pro	5205936
1314-98-3 OR Zinc sulfide	1314983
1318-00-9 OR Vermiculite	1318009
10101-41-4 OR Calcium sulfate dihydrate OR Calcium Sulfate	10101414
93-52-7 OR 1,2-dibromo(phenyl)ethane OR 1,2-dibromo-1-phenylethane	93527
24173-07-7 OR 1,2,3,4-Tetrabromo-2,3-dimethylbutane	24173077
171977-44-9 OR 2,4-Dibromo-1-phenoxybenzene	171977449
115-89-9 OR Phosphoric acid, methyl diphenyl ester OR diphenyl methyl phosphate	115899

636-28-2 OR 1,2,4,5-Tetrabromobenzene	636282
15541-60-3 OR Diphosphoric acid, compd. with 1,3,5-triazine-2,4,6-triamine (1:?)	15541603
13560-90-2 OR 4,5,6,7,13,14,15,16,19,19,20,20-Dodecachloroheptacyclo[9.6.1.1~4,7~.	13560902
58495-09-3 OR 1,2,3,4,5-Pentabromo-6-(chloromethyl)benzene	58495093
7025-06-1 OR Benzene, bromophenoxy-	7025061
337513-72-1 OR BDE-203	337513721
29660-68-2 OR Phosphoric acid, dimethylphenyl diphenyl ester	29660682
98923-48-9 OR 4-Bromo-2-chlorobutyl 3-bromo-2,2-dimethylpropyl phosphate	98923489
407606-57-9 OR 1,3-Dibromo-5-(4-bromophenoxy)benzene	407606579
90075-91-5 OR Bis[(pentabromophenyl)methyl] benzene-1,4-dicarboxylate	90075915
59080-40-9 OR 2,2',4,4',5,5'-Hexabromobiphenyl OR 2,4,5,2',4',5'-hexabromobiphenyl	59080409
66710-97-2 OR Bis(p-acryloxyethoxy)tetrabromobisphenol A	66710972
85681-73-8 OR Chloroalkanes, C10-14	85681738
1271168-40-1 OR Melamine-(aluminium phosphate)	1271168401
138265-88-0 OR Zinc Borate 2335	138265880
26265-08-7 OR Bisphenol A-epichlorohydrin-2,2',6,6'-tetrabromobisphenol A copolyme	26265087
59447-57-3 OR Poly(pentabromobenzyl acrylate)	59447573
189084-60-4 OR BDE-32	189084604
337513-77-6 OR BDE-40	337513776
446254-30-4 OR BDE-59	446254304
446254-35-9 OR BDE-64	446254359
446254-53-1 OR BDE-86	446254531
446254-58-6 OR BDE-91	446254586
446254-73-5 OR BDE-110	446254735
446254-78-0 OR BDE-115	446254780
446254-95-1 OR BDE-137	446254951
446255-00-1 OR BDE-144	446255001
446255-14-7 OR BDE-163	446255147
53551-87-4 OR BDE-168	53551874
446255-43-2 OR BDE-199	446255432
860302-33-6 OR Firemaster 550	860302336
446254-88-2 OR 1,2,3-Tribromo-4-(2,3,5-tribromophenoxy)benzene	446254882
31611-84-4 OR 1-Pentanone, 2,3,4,5-tetrabromo-1,5-diphenyl-	31611844
13518-93-9 OR Diphosphoric acid--1,3,5-triazinane-2,4,6-triimine (1/2)	13518939
34571-16-9 OR 1,2,3,4,7,7-Hexachloro-5-(pentabromophenyl)bicyclo[2.2.1]hept-2-ene	34571169
512-82-3 OR Tetrakis(hydroxymethyl)phosphanium hydroxide	512823
64864-08-0 OR Sodium bis(2,3-dibromopropyl) phosphate	64864080
66519-18-4 OR potassium bis(2,3-dibromopropyl) phosphate	66519184
363626-50-0 OR Bis(polyoxyethylene) methylphosphonate	363626500
73082-49-2 OR Cellulose, 6-carboxy, ion (neg.), tetrakis(hydroxymethyl)phosphonium	73082492
181028-79-5 OR Phosphoric trichloride, reaction products with bisphenol A and phenol	181028795
61840-22-0 OR Antiblaze 19	61840220
11112-10-0 OR Antimonic acid, sodium salt	11112100
115245-07-3 OR 1,1'-Biphenyl, 2,4,5-tribromo-	115245073
119264-61-8 OR 2,2',3,4,4',5,6,6'-Octabromobiphenyl	119264618
53211-22-6 OR Phosphonium, tetrakis(hydroxymethyl)-, ethanedioate (1:1) (salt)	53211226
6326-72-3 OR Trimethylphosphoramide	6326723

69887-11-2 OR 2,2',3,3',4,5,5',6'-Octabromobiphenyl	69887112
101-55-3 OR p-Bromodiphenyl ether	101553
32536-52-0 OR Octabromodiphenyl ether	32536520
40088-47-9 OR Tetrabromodiphenyl ether	40088479
3322-93-8 OR 1,2-Dibromo-4-(1,2-dibromoethyl)cyclohexane	3322938
25637-99-4 OR Hexabromocyclododecane	25637994
75-95-6 OR Pentabromoethane	75956
78-40-0 OR Triethyl phosphate	78400
6294-34-4 OR Bis(2-chloroethyl) 2-chloroethylphosphonate	6294344
38051-10-4 OR Phosphoric acid, 2,2-bis(chloromethyl)-1,3-propanediyl tetrakis(2-chlor	38051104
41203-81-0 OR (5-Ethyl-2-methyl-1,3,2-dioxaphosphorinan-5-yl)methyl dimethyl phosp	41203810
12124-97-9 OR Ammonium bromide	12124979
126-71-6 OR Triisobutyl phosphate OR tri-isobutylphosphate	126716
7550-45-0 OR Titanium tetrachloride	7550450
13810-83-8 OR Tetrabromophthalic acid	13810838
58965-66-5 OR Perbromo-1,4-diphenoxybenzene	58965665
207122-16-5 OR 2,2',3,4,4',5',6-Heptabromodiphenyl ether	207122165
23488-38-2 OR 2,3,5,6-Tetrabromo-p-xylene	23488382
5945-33-5 OR Phosphoric acid, P,P'-[(1-methylethylidene)di-4,1-phenylene] P,P,P',P'-te	5945335
189084-65-9 OR 1,2,3,4,5-Pentabromo-6-phenoxybenzene	189084659
14852-17-6 OR 1,2-Ethanediamine, phosphate OR ethylenediamine	14852176
57583-54-7 OR Tetraphenyl m-phenylene bis(phosphate)	57583547
7436-90-0 OR Benzene, dibromoethenyl-	7436900
Benzene, dibromoethenyl-	31780264
115-88-8 OR Octyl diphenyl phosphate	115888
147217-74-1 OR 1,2-Dibromo-3-(2-bromophenoxy)benzene	147217741
169102-57-2 OR (1R,2S,5S,6S,9S,10R)-1,2,5,6,9,10-Hexabromocyclododecane	169102572
2502-15-0 OR Tris(4-isopropylphenyl) phosphate	2502150
106232-86-4 OR Alkanes, C22-40, chloro	106232864
1067-98-7 OR Tris(3-chloropropyl)phosphate OR Fyrol PCF	1067987
6749-73-1 OR Tris(1,3-dichloropropan-2-yl) phosphite	6749731
51936-55-1 OR 7,8-Dibromo-1,2,3,4,11,11-hexachloro-1,4,4a,5,6,7,8,9,10,10a-decahyd	51936551
189084-68-2 OR 2,3,3',4,4',5,6-Heptabromodiphenyl Ether	189084682
84776-06-7 OR Alkanes, C10-32, chloro	84776067
158725-44-1 OR 4,4'-(1-Methylethylidene)bis[2,6-dibromophenol] polymer with (chlor	158725441
20208-95-1 OR 1,3,5-Triazine-2,4,6-triamine monophosphate OR melamine	20208951
446255-19-2 OR BDE-171	446255192
93703-48-1 OR BDE-77	93703481
337513-66-3 OR BDE-9	337513663
446254-15-5 OR BDE-22	446254155
446254-22-4 OR BDE-46	446254224
446254-26-8 OR BDE-54	446254268
446254-40-6 OR BDE-72	446254406
446254-45-1 OR BDE-78	446254451
446254-65-5 OR BDE-101	446254655
446254-69-9 OR BDE-106	446254699

446254-85-9 OR BDE-125	446254859
446254-91-7 OR BDE-133	446254917
446255-05-6 OR BDE-150	446255056
446255-10-3 OR BDE-159	446255103
407606-61-5 OR BDE-176	407606615
446255-27-2 OR BDE-186	446255272
97416-84-7 OR 1,1'-(Isopropylidene)bis(3,5-dibromo-4-(2,3-dibromo-2-methylpropoxy)	97416847
1402738-52-6 OR Alkanes, C24-28, chloro	1402738526
29426-78-6 OR 4,4'-(propane-2,2-diyl)bis(2-bromophenol)	29426786
198840-65-2 OR Tetradecane, chloro derivs.	198840652
29716-44-7 OR Tris(chloroethyl) phosphate	29716447
893843-07-7 OR 4,5,6,7-tetrabromo-1,1,3-trimethyl-3-(2,3,4,6-tetrabromophenyl)-2,3-	893843077
26604-51-3 OR Tris(dichloropropyl) phosphate	26604513
31977-87-4 OR 1,1'-{[(2Z)-2,3-dibromobut-2-ene-1,4-diyl]bis(oxy)}bis(2,4,6-tribromobe	31977874
26657-83-0 OR pentabromocyclododecene	26657830
52734-85-7 OR Torflam	52734857
1372804-76-6 OR Alkanes, C14-16, chloro	1372804766
25151-36-4 OR Phosphonium, tetrakis(hydroxymethyl)-, formate (salt)	25151364
39568-99-5 OR 3,6-Bis(bromomethyl)-1,2,4,5-tetrabromobenzene	39568995
513-02-0 OR Triisopropyl phosphate	513020
3296-90-0 OR Pentaerythritol dibromide OR 2,2-bis(bromomethyl)-1,3-propanediol	3296900
1163-19-5 OR 1,1'-Oxybis[2,3,4,5,6-pentabromobenzene] OR decabromobiphenyl ethe	1163195
608-90-2 OR Benzene, pentabromo-	608902
608-71-9 OR Pentabromophenol	608719
84303-48-0 OR 1,1'-Biphenyl, 2,3',4,4',5',6-hexabromo-	84303480
2050-47-7 OR 4,4'-Dibromodiphenyl ether	2050477
1522-92-5 OR 3-Bromo-2,2-bis(bromomethyl)propanol	1522925
102-54-5 OR Ferrocene	102545
13674-87-8 OR Tris(1,3-dichloro-2-propyl) phosphate OR tris(1,3-dichloro-2-propyl)pho	13674878
25155-23-1 OR Trixylyl phosphate OR tri-xylenyl phosphate	25155231
3234-02-4 OR 2,3-Dibromo-2-butene-1,4-diol OR dibromobutenediol	3234024
68953-58-2 OR Quaternary ammonium compounds, bis(hydrogenated tallow alkyl)dim	68953582
125997-21-9 OR Phosphoric trichloride, polymer with 1,3-benzenediol, phenyl ester	125997219
60348-60-9 OR 2,2',4,4',5-Pentabromodiphenyl ether OR pentabromodiphenyl ether	60348609
1067-12-5 OR Tris(hydroxymethyl)phosphine oxide	1067125
20120-33-6 OR Dimethyl (3-((hydroxymethyl)amino)-3-oxopropyl)phosphonate	20120336
85446-17-9 OR 1,1'-Oxybis(2,3,4,5-tetrabromobenzene)	85446179
1314-56-3 OR Phosphorus pentoxide	1314563
58398-71-3 OR Calcium magnesium hydroxide oxide	58398713
115-98-0 OR Bis(2-chloroethyl) vinylphosphonate	115980
28109-00-4 OR Bis(isopropylphenyl) phenyl phosphate	28109004
183658-27-7 OR 2-Ethylhexyl 2,3,4,5-tetrabromobenzoate	183658277
189084-61-5 OR 2,3',4,4'-Tetrabromodiphenyl ether	189084615
49690-63-3 OR Tris(dibromophenyl) phosphate	49690633
52434-90-9 OR 1,3,5-Tris(2,3-dibromopropyl)-1,3,5-triazine-2,4,6(1H,3H,5H)-trione OR	52434909
5445-19-2 OR Methyl 2-bromohexanoate	5445192
59447-55-1 OR (Pentabromophenyl)methyl acrylate	59447551

6903-63-5 OR 1,1'-Oxybis(3-bromobenzene)	6903635
65075-08-3 OR 1,4-Dibromo-2-(4-bromophenoxy)benzene	65075083
83694-71-7 OR 1-Bromo-3-(4-bromophenoxy)benzene	83694717
1332-81-6 OR Antimony oxide (Sb2O4) OR antimony oxide	1332816
7347-19-5 OR Tribromophenoxyethyl acrylate	7347195
13654-09-6 OR 1,1'-Biphenyl, 2,2',3,3',4,4',5,5',6,6'-decabromo- OR decabromobipheny	13654096
22031-17-0 OR Phosphonium, tetrakis(hydroxymethyl)-, phosphate (3:1) (salt) OR tetra	22031170
41583-09-9 OR 1,3,5-Triazine-2,4,6-triamine, phosphate OR melamine	41583099
42595-45-9 OR Bis((5-ethyl-2-methyl-1,3,2-dioxaphosphorinan-5-yl)methyl) methyl pho	42595459
30178-92-8 OR 1,1,2,2-Tetrabromocyclododecane	30178928
66108-37-0 OR 2,2-Bis(bromomethyl)-3-chloropropyl bis[2-chloro-1-(chloromethyl)eth	66108370
7046-64-2 OR Tris(2,4,6-tribromophenyl) phosphate	7046642
59080-39-6 OR PBB 103	59080396
6876-00-2 OR PBDE 002	6876002
117964-21-3 OR BDE-197 OR 2,2',3,3',4,4',6,6'-octabromodiphenyl ether	117964213
446254-60-0 OR 1,2,4,5-Tetrabromo-3-(2-bromophenoxy)benzene	446254600
407578-53-4 OR 1,2,3,4,5-Pentabromo-6-(3,5-dibromophenoxy)benzene	407578534
405237-86-7 OR 1,2,3,4,5-Pentabromo-6-(2,5-dibromophenoxy)benzene	405237867
84303-46-8 OR 3,3',4,4',5-Pentabromo-1,1'-biphenyl	84303468
1271172-98-5 OR Diphosphoric acid, zinc salt, compd. with 1,3,5-triazine-2,4,6-triamine	1271172985
147768-39-6 OR 3,3'-[(2-Methylpropyl)phosphoryl]di(propan-1-ol)	147768396
68928-70-1 OR 2,2'-[(1-Methylethylidene)bis[(dibromo-4,1-phenylene)oxymethylene]]	68928701
69882-11-7 OR 2,4-(or 2,6)-Dibromophenol, homopolymer	69882117
189084-62-6 OR 2,3',4',6-Tetrabromodiphenyl Ether	189084626
243982-82-3 OR 2,2',4,5'-Tetrabromodiphenyl Ether	243982823
147217-80-9 OR BDE-35	147217809
446254-18-8 OR BDE-42	446254188
446254-32-6 OR BDE-61	446254326
446254-37-1 OR BDE-67	446254371
446254-55-3 OR BDE-88	446254553
446254-61-1 OR BDE-94	446254611
446254-75-7 OR BDE-112	446254757
446254-81-5 OR BDE-121	446254815
446254-97-3 OR BDE-141	446254973
446255-02-3 OR BDE-146	446255023
446255-16-9 OR BDE-165	446255169
327185-13-7 OR BDE-170	327185137
446255-50-1 OR BDE-201	446255501
446254-27-9 OR 1,2,3-Tribromo-4-(3-bromophenoxy)benzene	446254279
5445-17-0 OR Propanoic acid, 2-bromo-, methyl ester	5445170
25357-79-3 OR 1,2-Benzenedicarboxylic acid, 3,4,5,6-tetrabromo-, sodium salt (1:2)	25357793
61583-60-6 OR Molybdenum zinc oxide	61583606
31454-48-5 OR 1,3,5,7-Tetrabromocyclooctane	31454485
46355-07-1 OR Phenyl propan-2-yl hydrogen phosphate	46355071
Tribromotrichlorocyclohexane	30554735
Tetrabromo trichloromethyl benzene	198126862

Formula	#1	#2	#3	#4	#5	#6	#7	#8	#9
CBrClF2				1					
C3H12N6O3						1			
C2Br2F4						1			
CHBr3						1			
C2H3NaO2				1					
CF3I				1					
C2H6N4O						1			
C6H5BrO				1					
-				1					
C8H7Br									
CH4N2O				1					
-				1					
C6F12O				1					
C6H18O24P6				1					
-							1		
-									
CBrF3				1					
C3H3BrO				1					
C8H7Br									
C2H8O7P2									
S3Sb2						1			
-				1					
H4IN							1		
-				1					
C3H5BrO2						1			
-				1					
C2H3Br				1			1		
C8HF15O2				1					
HNaO3S						1	1		
C6H5BrO				1					
C4H7BrO2						1			
C2Br4						1			
BF4H4N						1			
C4H12IO4P							1		
C6H15NO3									
C12H10O				1					
C3H5BrO2						1			
-							1		
C4H11O3P						1			
Ca									
C6H5BrO									
C22H4Br14O4		1							
-			1						

CaMoO4	1	1							
C21H20Br4O4	1		1	1			1		
C6H8Cl2N3O4P	1								
C12H15Br2N3C	1			1			1	1	
C12H15Br4N3C	1			1			1	1	
-			1						
-					1				
AlH4NaO4			1					1	
-					1				
-		1							
C12H6Br4O2									
C6H12Br3O4P									
C12H2Br8O			1						
C12H8Cl2O									
ClH4N					1	1			
C3H9O3P	1	1	1			1			
C12H2Br8									
-						1			
C8H24O12P2S	1					1			
C24H51O4P			1	1		1		1	
C27H33O4P			1						
C12H4Br6						1			
C2H4N4O2			1						
C18H15O3P					1				
C6H12Cl3O3P	1					1			
-					1				
C9H18Cl3O4P	1								
C12H8Br2O			1						
-			1						
BaO4S			1						
C14H7NaO7S			1		1				
C21H20Br4O4	1		1	1	1		1		
C14H4Cl12O	1		1			1		1	
C12H6Br4O4S	1		1	1		1			
C8H12Br4	1	1	1	1	1		1	1	
B6O11Zn2			1					1	
C12H7Br3O			1						
C15H24Br9O4P	1	1	1	1	1	1	1	1	
C21H6Br9N3O3	1	1	1	1	1	1	1	1	
C6H4Br2O									
C5H13O3P		1	1		1			1	
C12H6Br4	1		1				1		
-						1			
C12H30AlO6P3		1	1						
-			1		1	1			
C12H18Br6	1		1				1		1
C12H7Br3O			1						

C12H7Br3O			1						
C12H6Br4O			1						
C12H6Br4O			1						
C12H6Br4O			1						
C12H6Br4O			1						
C12H5Br5O			1						
C12H5Br5O			1						
C12H5Br5O			1						
C12H4Br6O			1						
C12H4Br6O			1						
C12H4Br6O			1						
C12H3Br7O			1						
C12H3Br7O			1						
C12H8K2O8S3		1	1						
F6K2Zr					1	1			
-			1						
C9H6Br4O									1
C6H14Br4NO4P						1			
C14H28Cl4O8P2		1							
C21H18Br3O4P									
C7H2Br6	1		1	1	1				
C18H51O22P5						1			
C14H19O7PS						1			
-			1		1	1			
-		1	1						
C14H10Br8O4		1							
-			1		1				
-		1							
-	1	1	1						
C9H9N5									
C12H8Br2O			1						
BH3O3	1		1			1			
C12H4Br6	1		1				1		
C3H9O4P			1		1				
C12H5Br5			1			1			
C18H15O4P	1	1	1	1	1	1		1	
C14H19O7PS						1			
C6Br6	1		1	1		1	1	1	1
P		1	1			1			
C14H8Br6O2	1	1	1	1		1	1	1	1
C18H22O2								1	
C20H27O4P	1		1		1	1		1	
C15H12Br4O2	1	1	1	1	1	1	1	1	1
C2H2Br4	1				1				
C16H35O4P					1				
C24H27O4P			1						
-	1	1	1	1		1			

C9H18Br2ClO4P		1			1	1			
-		1	1						
H8N2O4S			1		1	1			
-	1	1	1		1	1	1	1	
B2BaO4					1	1			
B8H8Na2O17			1		1				
C4H11O4P			1						
C12H11O4P									
C18H14									
C17H20Cl6O4	1					1			
C6H4Br2O	1		1	1	1	1	1	1	1
CaO4P2			1					1	
C12H7Br3O			1						
C12H8Br2	1		1				1		
C7H5Br3O	1			1					
C22H39O3P									
C16H19O4P			1						
C6H15O6P						1			
C10H24O12P2						1			
C24H32N4O2P2					1				
C15H24Br6O8P	1					1			
C12H3Br7O			1						
C26H31O4P			1						
C10H18Br4O3	1	1				1		1	
C18H39O4P			1						
C13H5Br5O2	1					1			
C12H5Br5O			1						
C17H16Br4O2	1	1	1	1		1			
-				1		1	1		
BO4P			1					1	
-	1	1	1						
C6H15N12O4P					1				
C12H4Br6O			1						
C12H7Br3O			1						
C12H7Br3O			1						
C12H6Br4O			1						
C12H6Br4O			1						
C12H5Br5O			1						
C12H5Br5O			1						
C12H5Br5O			1						
C12H5Br5O			1						
C12H4Br6O			1						
C12H4Br6O			1						
C12H4Br6O			1						
C12H4Br6O			1						
C12H3Br7O			1						
C4H14N2O7P2		1	1						

-									
MoO4Zn	1								
-									
C16H64Br2									
C12H2Br8	1		1			1	1		
C12H6Br4	1		1				1		
-	1		1						
C12H18Br6	1						1		
-									
C7H2Br4O2	1								
C11H8Br4	1								
C18H12Cl12									
C11H19O7PS			1			1			
-		1						1	
-	1			1	1		1	1	
C12H2Br8									
C9H4Cl6O4	1		1			1			1
C6H4Cl2N2O2		1							
C5Cl6	1		1		1	1			
C22H39O4P									
C6H6O2			1						
C12H2Br8									
C18H15OP					1				
C12O	1	1	1	1		1			
C7H10Br2O2	1				1				
C9H2Cl6O3	1		1		1	1			
C5H12O4		1	1		1				
C18H14									
C9H22NO5P		1	1			1			
C21H20Br4O2	1	1	1	1	1	1	1		
-						1			
-					1	1			
-			1						
Cl2Zn						1			
AlH3O3	1	1	1	1	1	1		1	
C4H11O2P								1	
-	1	1	1	1	1	1	1	1	
-					1				
C8H5Br3	1								
C12H15Br6N3C	1					1			
C12H5Br5O			1						
-			1						
C14H4Br10	1	1	1	1	1		1	1	1
C10H24O6P2									
C6H2Br4O									
C12H27OP									
C10H4Br3NO2		1			1				

-		1	1			1			
C22H23O4P									
C12H7Br3O			1						
C12H4Br6O			1						
-		1				1	1		
C14H28Cl5O9P	1				1	1			
-	1								
-			1						
-				1		1	1		
C20H28Br2N6P2					1	1			
C36H40BO8P						1			
C10H6Br2Cl6	1					1			
AlH6O6P3			1						
C12H18Br6	1		1				1		1
C12H8Br2O			1						
C12H7Br3O			1						
C12H6Br4O			1						
C12H6Br4O			1						
C12H6Br4O			1						
C12H6Br4O			1						
C12H5Br5O			1						
C12H5Br5O			1						
C12H5Br5O			1						
C12H4Br6O			1						
C12H4Br6O			1						
C12H4Br6O			1						
C12H3Br7O			1						
C12H3Br7O			1						
C12H18Br6	1								
C17H32Cl6O8P2		1	1						
-							1		
-		1							
AlH6O12P3		1							
C7H17O7P						1			
C8H17O9P						1			
-		1	1						
C7H10Cl2N3O3	1								
-					1	1			
C13H4Br4Cl6			1						
-		1							
-	1	1	1		1				
-	1		1					1	
-				1		1	1		
C18Cl14									
C12H20Br8MgO8P2						1			
C15H17O4P					1				
C15H12Cl4O2	1		1			1			

C12H4Br6						1			
C12HBr9	1		1			1	1		
C12H27O4P		1	1	1	1			1	
-		1				1			
-		1	1		1	1		1	
(Br)y(Br)xC12H	1			1					
-	1		1		1	1			
C7H3Br5	1		1	1	1	1	1	1	1
C18H26O6					1				
C9H18Cl3O4P	1	1	1		1	1			
-			1						
-				1		1			
C12H6Br4O	1		1				1	1	
C21H20Br8O2	1	1	1	1	1	1	1	1	1
C11H8Cl6O4	1					1			
C4H12O4P	1								
C9H7Br3O	1	1	1	1		1	1		1
C12HBr9O			1						
C9H11O4P			1					1	
C12H18Br6	1						1		
AlNaO2			1		1	1		1	
-	1								
C30H39O4P		1	1						
C6H3Br3									
C12H4Br6O	1		1				1	1	
C15H33O4P			1						
C14H4Br10O	1		1	1					
C3H5N5O									
C4H2Br6		1							
C6H9N9O3		1	1			1		1	
C12H6Br4O			1						
C12H2Br8O			1						
C12H4Br6O			1						
C12H5Br5O			1						
C18H12Br8	1		1	1			1	1	
C10H16Cl6	1								
C14H14Br4O4	1	1				1			
C15H13Br2NO	1					1			
-				1		1			
C12H9KO5S2		1	1						
C12H7Br3O			1						
C12H6Br4O			1						
C12H6Br4O			1						
C12H6Br4O			1						
C12H5Br5O			1						
C12H5Br5O			1						
C12H5Br5O			1						

C12H5Br5O			1						
C12H4Br6O			1						
C12H4Br6O			1						
C12H4Br6O			1						
C12H4Br6O			1						
C12H2Br8O			1						
C12H2Br8O			1						
C12HBr9O			1						
C19H16Br4O4	1		1	1			1		
H6NaO6Sb	1								
C9H7Br3O	1			1					
C2H5O4P			1						
C13H5Br3Cl6									
-	1	1	1		1	1		1	
C6H11Cl4O4P									
-		1							
-					1			1	
-					1	1			
C12H18Br6	1						1		
-	1				1				
-		1							
C4CaMg3O12					1	1			
-	1	1		1		1	1		1
-	1			1		1	1		1
-	1				1				
-		1							
C12HBr9						1			
O16P4Zr3			1						
C6H6N10					1				
C18Cl14									
C22H30O2S					1				
-	1	1	1		1	1		1	
-					1	1			
O3Sb2	1	1	1		1	1		1	
C12H9Br	1		1				1		
C6H6Br5Cl	1	1				1			
C21H21O4P			1		1				
C24H51O3P					1				
C9H15N3O6		1							
C12H18Br6	1	1	1	1		1		1	1
-	1	1	1		1	1		1	
C12H4Br6O	1		1				1	1	
C19H20Br4O4	1	1	1	1	1	1	1		1
C9H5Br7O									
Cl3Sb					1				
C12H8Br2O			1						
CMgO3			1						

H2MgO2	1	1	1	1	1	1		1
AlHO2		1	1					1
C24H19O4P								
C12H5Br5O			1					
C6H6Br6	1		1			1		
C12H5Br5O	1		1				1	1
C12H7Br3O	1		1				1	1
C9H21O4P			1					1
C6H12N3OP			1			1		
C12HBr9O			1					
C6H12N6O3								
C12H6Br4O			1					
C10H20O5P2S2			1			1		
C11H22N12O8P2						1		
C12H5Br5O			1					
C12H6Br4	1		1				1	
C12H2Br8O			1					
C12H2Br8O			1					
C12H4Br6								
C9H8Br2O2	1					1		
-						1		
C12H7Br3O			1					
H16Mo8N4O26			1			1		
-			1			1		1
C12HBr9						1		
C12H3Br7O			1					
C12H7Br3O			1					
C12H7Br3O			1					
C12H6Br4O			1					
C12H6Br4O			1					
C12H6Br4O			1					
C12H6Br4O			1					
C12H5Br5O			1					
C12H5Br5O			1					
C12H5Br5O			1					
C12H4Br6O			1					
C12H4Br6O			1					
C12H4Br6O			1					
C12H3Br7O			1					
C12H3Br7O			1					
C12H10Br4O4	1							
-			1					
-	1	1	1	1		1		
CH6O4S		1						
C15H6Cl12								
C5H16ClN2O5P	1					1		
C18H2Br12								

C12H18Br6	1						1		
C14H10Br4O4S	1			1			1		
C11H8Br4	1								
C3H7Br2O4P			1						
-						1			
-					1	1			
C7H3Br4Cl	1		1	1					
CaCl2						1			
C2H7O3P	1					1			
-			1			1			
C4H12ClO4P	1	1			1	1			
C6H12Cl3O4P	1		1	1	1	1		1	1
C9H15Br6O4P	1	1	1	1		1	1	1	
C18H39O7P			1	1		1		1	
C12H5Br5	1		1			1	1		
H6N2O3S			1		1	1			
C12H7Br3O			1						
C12H9Br									
-					1				
C2H7O4P			1						
C9H18Cl3O4P	1	1	1	1	1	1		1	1
C10H20Cl4O8P	1					1			
-		1		1		1	1		
-			1						
C4H7N5									
H6NO4P			1					1	
Cl2Mg						1			
C12H3Br7O			1						
C18H14Br8O4S	1		1	1					
CO3Zn					1				
B4H8N2O7						1			
C6H4Br3N	1					1			
C21H21O4P			1						
C9H7Br5O	1		1	1		1	1		1
C12H5Br5O			1						
C12H8Br2O			1						
C9H5Br5O	1		1	1		1	1		
O3SnZn			1			1			
C12H3Br7O			1						
C12H6Br4	1								
-				1					
Na2O3Sn						1			
H6O6SnZn			1			1			
C14H23O4P			1						
C12H5Br5O			1						
C12H7Br3O			1						

C12H6Br4O			1						
C12H6Br4O			1						
C12H6Br4O			1						
C12H5Br5O			1						
C12H5Br5O			1						
C12H5Br5O			1						
C12H5Br5O			1						
C12H4Br6O			1						
C12H4Br6O			1						
C12H4Br6O			1						
C12H4Br6O			1						
C12H3Br7O			1						
C12H2Br8O			1						
-						1			
-							1		
C18H9Br6O4P									
C10H9Br5O									
C10H20BrCl									
C9H18Br2ClO4P									
-					1				
-		1							
-			1		1				
C18H12Br8	1						1	1	
C10H6Br4O4	1			1					
C10H29O14P3						1			
-		1							
-						1			
C38H40O8P2			1						
-		1							
-		1							
C12H2Br8									
C6H2Br4O									
C12H12N10									
C3H6N6	1	1	1		1	1		1	
C12H3Br7						1			
C6H3Br3O	1	1	1	1	1	1	1	1	1
C12H6Br4O2									
C18H6Br8O									
-		1				1			
C18H4Br8N2O4	1	1	1	1	1	1	1	1	1
-	1		1		1				
-			1		1				
C10Cl8									
C8Br4O3	1	1	1	1	1	1			1
C24H51O4P			1		1	1			
C18H14									
H9N2O4P					1	1			

-			1						
C21H21O4P	1		1	1					
C12H9O2P		1	1					1	
C8H7Br3O									
C12H7Br3O			1						
-					1	1			
O5Sb2	1					1			
C22H39O4P									
C20H20Br4N2C	1		1	1		1			
C12H4Br6O			1						
C12H3Br7O	1		1						
C12H8Br2O			1						
C8H2Cl4O4									
C4H2Mg5O14					1				
C12H24Cl4O9P	1				1				
C12H7Br3O			1						
C15H16Br4O7	1	1	1	1	1	1	1	1	
C12H4Br6O			1						
-	1	1							
-						1	1		
-				1		1	1		
-		1	1						
NaO3Sb	1				1	1			
-									
C12H18Br6	1		1				1		1
C12H8Br2O			1						
C12H7Br3O			1						
C12H6Br4O			1						
C12H6Br4O			1						
C12H6Br4O			1						
C12H6Br4O			1						
C12H5Br5O			1						
C12H5Br5O			1						
C12H5Br5O			1						
C12H4Br6O			1						
C12H4Br6O			1						
C12H4Br6O			1						
C12H3Br7O			1						
C12H3Br7O			1						
C14H4Br10O2	1	1			1	1			
B2O6Zn3	1	1	1		1	1			
C15H33O4P									
-					1	1			
C21H16Br4O4	1		1	1			1		
C4H12BrO4P						1			
C12H6Br4	1		1				1		
-					1	1			

C28H29O5P		1	1						
-		1							
-	1								
C12H18Br6	1						1		
-	1		1				1		
-						1			
C11H13Br4NO2		1							
-			1						
-		1	1						
C10H14Br6	1			1			1	1	
H4O5P2S2									
C8H7Br									
C15H16O2		1	1						
C10Cl12	1		1			1		1	
MoO3						1			
C8H5Br5	1	1	1	1		1	1	1	1
C12H3Br7	1		1			1	1		
C12H4Br6						1			
C3H6Br2O	1		1	1	1				
C9H15Cl6O4P	1		1			1			
-						1			
C3H3N3O3			1					1	
C6H8Br2O2	1					1			
C6H15O3P			1			1			
C8Cl4O3	1		1		1	1			
C19H17O4P									
C9H18Cl3O4P	1								
C6H11Br4O4P	1		1			1			
C18H12Cl12	1	1	1		1	1		1	1
C24H34Br4O4	1	1	1	1	1		1	1	1
-		1		1			1		
-		1							
F6K2Ti					1	1			
O3W									
B2O3	1				1				
B4H10Na2O12			1						
CaH2O2			1					1	
OZn	1		1			1			
O4P			1						
C9H18N2O					1				
SZn					1				
-			1						
CaH4O6S					1				
C8H8Br2	1		1		1				
C6H10Br4						1			
C12H8Br2O			1						
C13H13O4P					1				

C6H2Br4									
-		1	1						
C20H12Cl12									
C7H2Br5Cl	1		1	1					
C12H9BrO			1						
C12H2Br8O			1						
C20H19O4P									
C9H17Br2ClO4P					1				
C12H7Br3O			1						
C22H8Br10O4	1								
C12H4Br6	1		1			1	1		1
C25H24Br4O6	1		1	1		1	1		
-				1		1	1		
C6H14AlN12O10P3			1						
-	1								
-		1							
-	1	1	1			1			
C12H7Br3O			1						
C12H6Br4O			1						
C12H6Br4O			1						
C12H6Br4O			1						
C12H5Br5O			1						
C12H5Br5O			1						
C12H5Br5O			1						
C12H5Br5O			1						
C12H4Br6O			1						
C12H4Br6O			1						
C12H4Br6O			1						
C12H4Br6O			1						
C12H2Br8O			1						
-			1						
C12H4Br6O			1						
C17H14Br4O		1							
C6H16N12O7P2					1				
C13H3Br5Cl6	1		1	1			1	1	
C4H13O5P									
C6H10Br4NaO4P						1			
C6H10Br4KO4P						1			
-		1							
-						1			
-		1	1		1				
-	1					1			
Na3O4Sb					1				
C12H7Br3	1		1				1		
C12H2Br8									
C6H13O8P						1			
C3H12N3OP						1			

C12H2Br8									
C12H9BrO			1						
-	1	1	1	1	1	1			
-		1			1	1			
C8H12Br4	1	1	1	1		1	1	1	1
-	1	1	1	1	1	1		1	1
C2HBr5	1				1				
C6H15O4P	1	1	1			1		1	
C6H12Cl3O3P	1				1				
C13H24Cl6O8P	1	1	1	1	1			1	1
C9H20O6P2	1					1			
BrH4N			1		1	1			
C12H27O4P			1					1	
Cl4Ti									
C8H2Br4O4			1						
C18Br14O2	1		1	1	1	1	1	1	
C12H3Br7O	1		1				1	1	
C8H6Br4	1		1	1		1	1	1	
C39H34O8P2			1	1				1	
C12H5Br5O			1						
-		1	1		1				
C30H24O8P2			1		1	1		1	
C8H6Br2	1		1						
-	1			1	1	1	1	1	
C20H27O4P					1	1			
C12H7Br3O			1						
C12H18Br6	1						1		
C27H33O4P			1						
-						1			
C9H18Cl3O4P									
C9H15Cl6O3P	1					1			
C13H12Br2Cl6	1		1	1		1	1	1	1
C12H3Br7O			1						
-				1		1			
-									
C3H9N6O4P								1	
C12H3Br7O			1						
C12H6Br4O			1						
C12H8Br2O			1						
C12H7Br3O			1						
C12H6Br4O			1						
C12H6Br4O			1						
C12H6Br4O			1						
C12H6Br4O			1						
C12H5Br5O			1						
C12H5Br5O			1						

C12H5Br5O			1						
C12H4Br6O			1						
C12H4Br6O			1						
C12H4Br6O			1						
C12H3Br7O			1						
C12H3Br7O			1						
C23H24Br8O2		1	1					1	
-		1							
C15H14Br2O2									
-		1							
-	1								
C18H12Br8	1						1	1	
-									
C16H8Br8O2	1								
-			1						
-			1						
-		1							
C5H13O6P						1			
C8H4Br6	1					1			
C9H21O4P									
C5H10Br2O2	1		1	1		1	1	1	1
C12Br10O	1	1	1	1	1	1	1	1	
C6HBr5	1		1						
C6HBr5O	1		1	1	1	1	1	1	1
C12H4Br6						1			
C12H8Br2O			1						
C5H9Br3O	1		1	1			1	1	
C10H10Fe						1			
C9H15Cl6O4P	1	1	1	1	1	1		1	1
-			1			1			
C4H6Br2O2	1			1	1	1			
-		1	1						
-		1	1						
C12H5Br5O	1		1				1	1	
C3H9O4P	1								
C6H14NO5P	1	1			1	1			
C12H2Br8O	1		1						
O10P4			1						
CaH2MgO3			1					1	
C6H11Cl2O3P	1					1			
-									
C15H18Br4O2	1	1	1	1			1	1	1
C12H6Br4O			1						
C18H9Br6O4P	1					1			
C12H15Br6N3C	1	1	1	1			1	1	
C7H13BrO2	1				1				
C10H5Br5O2	1	1	1	1		1	1	1	

C12H8Br2O			1						
C12H7Br3O			1						
C12H8Br2O			1						
O4Sb2					1				
C11H9Br3O3	1								
C12Br10	1		1	1		1			
C12H36O16P4						1			
-			1					1	
C15H31O9P3	1				1	1			
C12H20Br4					1				
C11H18Br2Cl5(	1								1
C18H6Br9O4P	1					1			
C12H5Br5	1		1				1		
C12H9BrO			1						
C12H2Br8O			1						
C12H5Br5O			1						
C12H3Br7O			1						
C12H3Br7O			1						
C12H5Br5	1						1		
C6H14N12O7P2Zn			1						
C10H23O3P		1							
-	1	1	1						
-					1	1			
C12H6Br4O			1						
C12H6Br4O			1						
C12H7Br3O			1						
C12H6Br4O			1						
C12H6Br4O			1						
C12H6Br4O			1						
C12H5Br5O			1						
C12H5Br5O			1						
C12H5Br5O			1						
C12H5Br5O			1						
C12H4Br6O			1						
C12H4Br6O			1						
C12H4Br6O			1						
C12H3Br7O			1						
C12H2Br8O			1						
C12H6Br4O			1						
C4H7BrO2	1				1				
C8Br4Na2O4						1			
-	1								
		1							
C9H13O4P			1						
-									
-									

#10	#11	#12	#13	SUM all d	HIGH = 3	MOD = 2	LOV	QSUR SCOI
				1			0	0.15
				1			0	0.12
				1			0	0.14
				1			0	0.15
				1			0	0.28
				1			0	0.13
				1			0	0.74
		2		3			0	0.14
				1			1	#N/A
		1		1			0	
				1			0	0.88
				1			0	#N/A
				1			0	0.36
				1			0	0.52
				1			0	#N/A
			1	1			0	#N/A
				1			0	0.13
				1			0	0.13
		1		1			0	0.23
		1		1			0	
				1			0	0.76
				1			0	#N/A
				1			0	0.13
				1			0	#N/A
				1			0	0.75
				1			0	#N/A
1				3			0	0.10
				1			0	0.44
				2			0	0.59
		2		3			0	0.28
				1			0	0.36
				1			0	0.11
				1			0	0.20
				1			0	0.68
			1	1			0	0.12
				1			0	0.69
				1			0	0.44
				1			0	#N/A
				1			0	0.22
			1	1			0	0.75
		2		2			0	0.26
		1		2			2	0.97
				1			1	#N/A

				2	2	0.76
	1	2		7	3	0.92
				1	1	0.94
	1	2		7	3	0.97
	1	2		7	3	0.97
1		1		3	2	0.99
		1		2	2	#N/A
				2	2	0.93
				1	1	#N/A
				1	1	#N/A
		1		1	1	
		1		1	1	
		2		3	2	0.98
		1		1	1	
				2	2	0.13
		1	1	6	3	0.99
		1		1	1	0.97
		2		3	2	0.97
			1	3	2	0.68
1	1	3		9	3	0.95
		3		4	3	0.93
				1	1	0.97
				1	1	0.71
				1	1	0.78
		2		4	3	0.91
				1	1	#N/A
				1	1	0.98
		1		2	2	0.94
				1	1	0.52
				1	1	0.79
				2	2	0.06
	1	2		8	3	0.94
	1	3		8	3	0.44
	1	2		7	3	0.86
1	1	3	1	13	3	0.92
			1	3	2	0.84
				1	1	0.94
	1	3		12	3	0.99
1	1	3		13	3	0.96
		2		2	2	0.80
				4	3	0.97
		1		4	3	0.93
				1	1	#N/A
		1	1	4	3	0.81
		1	1	5	3	0.93
		2		6	3	0.92
				1	1	0.94

				1	1	0.98
				1	1	0.96
				1	1	0.98
				1	1	0.97
				1	1	0.94
				1	1	0.98
				1	1	0.98
				1	1	0.97
				1	1	0.98
				1	1	0.97
				1	1	0.98
				1	1	0.98
				1	1	0.98
				2	2	0.58
			1	3	2	0.20
				1	1	#N/A
		3		4	3	0.92
				1	1	0.99
				1	1	#N/A
		1		1	1	
	1	3		8	3	0.96
				1	1	0.68
				1	1	0.81
				3	2	0.91
				2	2	#N/A
				1	1	0.97
				2	2	#N/A
				1	1	#N/A
				3	2	#N/A
		2		2	2	0.88
				1	1	0.92
				3	2	0.91
		1		4	3	0.95
	1	3		6	3	0.96
				2	2	0.97
1	1	3		12	3	0.96
				1	1	0.81
	1	3		11	3	0.97
			1	4	3	#N/A
1	1	3	1	14	3	0.95
			1	2	2	0.65
		3		8	3	0.98
1	1	3	1	15	3	0.95
				2	2	0.60
1		2		4	3	0.86
		2		3	2	0.95
1		1		7	3	#N/A

	1		4	3	0.97
			2	2	0.37
			3	2	0.54
		1	8	3	0.53
		1	3	2	0.97
			2	2	0.97
	2		3	2	0.89
	2		2	2	
	1		1	1	0.84
	2		4	3	0.89
1	3	1	13	3	0.48
			2	2	0.88
			1	1	0.96
	2		5	3	0.54
	2		4	3	0.93
	1		1	1	
	2		3	2	0.99
		1	2	2	0.68
		1	2	2	0.68
			1	1	0.81
			2	2	0.98
	1		2	2	0.99
			1	1	0.98
	1		5	3	0.91
	2		3	2	0.96
		1	3	2	0.96
			1	1	0.98
1	3	1	10	3	0.97
			3	2	#N/A
			2	2	0.96
	1	1	5	3	#N/A
			1	1	0.90
			1	1	0.96
			1	1	0.97
			1	1	0.95
			1	1	0.97
			1	1	0.97
			1	1	0.98
			1	1	0.97
			1	1	0.99
			1	1	0.98
			1	1	0.98
			1	1	0.98
			1	1	0.98
			1	1	0.98
			1	1	0.98
	1		3	2	0.87

		1		1	1	
				1	1	0.36
			1	1	1	0.95
		1		1	1	
				4	3	0.98
		1		4	3	0.92
		2		4	3	0.96
				2	2	0.92
		1		1	1	#N/A
		1		2	2	0.86
		1		2	2	0.78
		3		3	2	0.68
				2	2	0.74
		1		3	2	#N/A
		2		7	3	0.79
		1		1	1	0.99
1	1	2	1	9	3	0.42
				1	1	0.43
1	1	2		8	3	0.94
		2		2	2	0.98
				1	1	0.17
		1		1	1	0.99
		2		3	2	0.95
1		2	1	9	3	0.97
				2	2	0.95
	1	2		7	3	0.91
		2		5	3	0.86
		1		1	1	0.84
1				4	3	0.93
1	1	3	1	13	3	0.95
1		1		3	2	#N/A
				2	2	0.95
				1	1	0.95
				1	1	0.51
1		1	1	10	3	0.84
		1		2	2	0.81
		1		9	3	#N/A
				1	1	0.81
				1	1	#N/A
		1	1	4	3	0.97
				1	1	0.97
1				2	2	#N/A
1	1	3	1	14	3	0.94
		2		2	2	0.99
		1		1	1	0.91
		1		1	1	
		1	1	4	3	0.89

1				4	3	0.98
		2		2	2	0.98
				1	1	0.97
		2		3	2	0.98
				3	2	#N/A
				3	2	0.98
	1			2	2	#N/A
				1	1	0.88
				3	2	#N/A
				2	2	0.66
				1	1	0.90
				2	2	0.82
				1	1	0.95
		2		6	3	0.92
				1	1	0.92
				1	1	0.97
				1	1	0.98
				1	1	0.98
				1	1	0.90
				1	1	0.97
				1	1	0.97
				1	1	0.97
				1	1	0.98
				1	1	0.98
				1	1	0.97
				1	1	0.99
				1	1	0.99
				1	1	0.99
				1	1	0.94
	1	2		5	3	0.99
				1	1	#N/A
1		1		3	2	0.95
				1	1	0.73
				1	1	0.68
				1	1	0.80
				2	2	#N/A
				1	1	0.96
		1	1	4	3	#N/A
		2		3	2	0.71
				1	1	#N/A
		1	1	6	3	0.19
	1	3		7	3	0.66
				3	2	#N/A
		1		1	1	0.99
				1	1	0.99
				1	1	0.96
		2		5	3	0.95

				1	1	0.98
		1		5	3	0.99
1	1	3	1	11	3	0.98
1		2		5	3	0.98
1		3	1	10	3	0.95
1				3	2	#N/A
1		2		7	3	0.94
	1	3	1	13	3	0.96
				1	1	0.31
1		2	1	9	3	0.98
1		2		4	3	0.98
				2	2	#N/A
		3		7	3	0.91
	1	3	1	14	3	0.98
				2	2	0.83
				1	1	0.68
1	1	3	1	13	3	0.92
		2		3	2	0.98
				2	2	0.80
		2		4	3	0.92
				4	3	0.83
				1	1	0.13
1		3		6	3	0.97
		2		2	2	
		3		7	3	0.98
		2		3	2	0.98
	1	2		6	3	0.91
		2		2	2	0.76
				1	1	0.83
		2		6	3	0.96
				1	1	0.94
		2		3	2	0.99
				1	1	0.99
				1	1	0.97
	1	3		9	3	0.92
				1	1	#N/A
1				4	3	0.97
				2	2	0.90
				2	2	#N/A
			1	3	2	0.66
				1	1	0.97
				1	1	0.97
				1	1	0.97
				1	1	0.98
				1	1	0.98
				1	1	0.98
				1	1	0.98

				1	1	0.97
				1	1	0.98
				1	1	0.99
				1	1	0.98
				1	1	0.94
		1		2	2	0.98
		2		3	2	0.98
		2		3	2	0.98
	1	2		7	3	0.94
				1	1	0.89
				2	2	0.89
				1	1	0.73
		1		1	1	0.79
1		1	1	9	3	0.76
		2		2	2	0.96
				1	1	#N/A
				2	2	0.37
		1		3	2	0.94
				2	2	0.92
				2	2	0.67
				1	1	0.99
				2	2	0.63
				6	3	#N/A
		1		6	3	#N/A
				2	2	#N/A
				1	1	0.95
		1		2	2	0.99
				1	1	0.90
				1	1	0.48
		1		1	1	0.99
				1	1	0.12
1	1	3	1	12	3	0.94
				2	2	0.57
1		1	1	9	3	0.93
		1		4	3	0.85
		2	1	6	3	0.99
		3		5	3	0.94
				1	1	0.61
				1	1	0.76
1	1	3		12	3	0.92
1		2		9	3	0.93
		3		7	3	0.97
1	1	3		13	3	0.96
		1		1	1	
				1	1	0.25
				1	1	0.76
				1	1	0.63

		1	1	9	3	0.75
				3	2	0.62
		1		1	1	
		3		4	3	0.97
				3	2	0.99
		3		7	3	0.96
		3		7	3	0.91
	1	3		6	3	0.97
			1	3	2	0.93
		2		3	2	0.98
			1	1	1	0.56
				1	1	0.91
		1		3	2	0.94
				1	1	0.95
				1	1	0.97
				3	2	0.73
		2		3	2	0.98
		2		3	2	0.98
		1		1	1	0.96
				2	2	0.41
				1	1	#N/A
		2		3	2	0.91
		1		3	2	0.12
		1	1	5	3	0.93
		1		2	2	0.99
		1		2	2	0.98
				1	1	0.94
				1	1	0.98
				1	1	0.97
				1	1	0.87
				1	1	0.97
				1	1	0.91
				1	1	0.97
				1	1	0.97
				1	1	0.94
				1	1	0.98
				1	1	0.98
				1	1	0.98
				1	1	0.98
				1	1	0.97
				1	1	0.89
				1	1	0.81
		1		6	3	0.94
1				2	2	0.20
		2		2	2	0.68
				2	2	0.30
		1		1	1	

		2		4	3	0.92
	1	2		6	3	0.85
		1		2	2	0.78
		3		3	2	0.68
				1	1	0.99
				1	1	#N/A
			1	3	2	#N/A
	1	3		7	3	0.96
				1	1	0.75
				2	2	0.84
				2	2	0.96
			1	5	3	0.68
1	1	3	1	13	3	0.97
	1	3	1	12	3	0.99
1	1	3	1	10	3	0.96
		1		5	3	0.97
			1	4	3	0.68
		1		2	2	0.87
		1		1	1	0.85
				1	1	0.05
		2		3	2	0.86
1	1	3	1	14	3	0.96
				2	2	0.97
				4	3	#N/A
				1	1	#N/A
		2		2	2	0.90
				2	2	0.48
				1	1	0.75
		2		3	2	0.99
	1	2		6	3	0.97
				1	1	0.63
				1	1	0.76
				2	2	0.93
		3		4	3	0.94
1	1	3		11	3	0.98
		2		3	2	0.94
		1		2	2	0.50
	1	3		9	3	0.97
		1	1	4	3	0.95
				1	1	0.98
				1	1	#N/A
		2	1	4	3	0.93
				1	1	0.97
		1	1	4	3	0.92
		2		3	2	0.99
		2		3	2	0.98
				1	1	0.47

				1	1	0.98
				1	1	0.99
				1	1	0.96
				1	1	0.98
				1	1	0.97
				1	1	0.97
				1	1	0.98
				1	1	0.98
				1	1	0.98
				1	1	0.99
				1	1	0.98
				1	1	0.98
		2		3	2	0.98
				1	1	0.87
				1	1	#N/A
		1		1	1	
		1		1	1	
		1		1	1	
		1		1	1	
				1	1	#N/A
				1	1	#N/A
		1		3	2	0.93
	1	2		6	3	0.96
				2	2	0.96
				1	1	0.68
				1	1	#N/A
				1	1	#N/A
	1	2		4	3	0.95
				1	1	#N/A
				1	1	0.49
		1		1	1	0.99
		1		1	1	0.94
		2		2	2	0.90
1		2	1	10	3	0.90
				1	1	0.98
	1	3	1	14	3	0.88
		1		1	1	
		1		1	1	
1				3	2	0.98
1	1	2	1	14	3	0.95
1		2		6	3	0.98
		2		4	3	0.95
		1		1	1	0.92
	1			8	3	0.93
				3	2	0.95
		1		1	1	0.84
				2	2	0.48

				1	1	#N/A
		3		6	3	0.94
	1	2	1	7	3	0.95
		1		1	1	
		1		2	2	0.96
				2	2	0.61
				2	2	0.93
		1		1	1	
		1	1	6	3	0.94
		1		2	2	0.97
				2	2	0.98
				1	1	0.95
			1	1	1	0.86
				1	1	0.81
				2	2	0.96
				1	1	0.94
1	1	2		12	3	0.93
		2		3	2	0.98
	1			3	2	#N/A
				2	2	#N/A
				3	2	#N/A
				2	2	#N/A
			1	4	3	0.95
		1		1	1	
		2		6	3	0.92
				1	1	0.60
				1	1	0.90
				1	1	0.93
				1	1	0.94
				1	1	0.98
				1	1	0.96
				1	1	0.98
				1	1	0.96
				1	1	0.97
				1	1	0.99
				1	1	0.98
				1	1	0.97
				1	1	0.98
				1	1	0.98
1		2	1	8	3	0.98
1			1	7	3	0.94
		1		1	1	
				2	2	#N/A
	1	2		7	3	0.96
				1	1	0.68
				3	2	0.97
		1		3	2	#N/A

				2	2	0.91
				1	1	#N/A
				1	1	#N/A
		2		4	3	0.92
	1	2		6	3	#N/A
				1	1	#N/A
				1	1	0.89
				1	1	#N/A
1				3	2	0.90
	1	2		7	3	0.90
			1	1	1	0.76
		1		1	1	0.99
				2	2	0.70
	1	2	1	8	3	0.95
				1	1	0.25
	1	3		12	3	0.94
		2		6	3	0.98
				1	1	0.98
				4	3	0.95
	1	2		6	3	0.99
				1	1	0.87
				2	2	0.69
				2	2	0.95
		1		3	2	0.99
	1	2		7	3	0.93
		1		1	1	0.95
				1	1	0.98
		2		5	3	0.99
1	1	3	1	13	3	0.68
1	1	3		13	3	0.96
				3	2	0.89
1		1		3	2	0.13
			1	3	2	0.20
			1	1	1	0.24
				2	2	0.70
				1	1	0.97
				2	2	0.72
				3	2	0.36
				1	1	0.89
				1	1	0.09
				1	1	0.71
				1	1	#N/A
				1	1	0.79
				3	2	0.97
				1	1	0.95
				1	1	0.92
				1	1	0.98

		1		1	1	
		1		3	2	0.93
		2		2	2	
	1	2		6	3	0.96
		1		2	2	0.85
		2		3	2	0.98
		2		2	2	0.94
				1	1	0.96
				1	1	0.91
		1		2	2	0.97
		1		6	3	0.97
	1	2		8	3	0.91
				3	2	#N/A
				1	1	0.96
				1	1	0.81
1				2	2	0.90
		1	1	6	3	0.91
				1	1	0.91
				1	1	0.90
				1	1	0.98
				1	1	0.97
				1	1	0.98
				1	1	0.97
				1	1	0.97
				1	1	0.99
				1	1	0.98
				1	1	0.99
				1	1	0.97
				1	1	0.98
		1		2	2	0.98
				1	1	#N/A
				1	1	0.98
				1	1	0.94
				1	1	0.90
	1	2		8	3	0.75
			1	1	1	0.68
				1	1	0.99
				1	1	0.99
				1	1	#N/A
				1	1	#N/A
		1		4	3	0.94
				2	2	#N/A
				1	1	0.97
				3	2	0.98
		1		1	1	0.99
				1	1	0.68
				1	1	0.89

		1		1	1	0.98
		1		2	2	0.37
1		2	1	10	3	0.98
1				4	3	0.97
	1	3	1	13	3	0.96
1		2	1	12	3	0.94
	1			3	2	0.34
1	1	3		10	3	0.97
				2	2	0.98
1	1	3		12	3	0.98
				2	2	0.97
		1		4	3	0.13
	1	3		6	3	0.95
			1	1	1	0.15
		1	1	3	2	0.86
1	1	2	1	12	3	0.98
		3		7	3	0.99
	1	3		10	3	0.75
	1	3		7	3	0.99
				1	1	0.98
				3	2	0.77
	1	3	1	9	3	0.96
		2		4	3	0.79
	1		1	8	3	#N/A
		2	1	5	3	0.98
				1	1	0.90
				2	2	0.92
	1	2		4	3	0.93
				1	1	#N/A
		2	1	3	2	0.96
				2	2	0.93
	1	3		11	3	0.82
		2		3	2	0.98
				2	2	#N/A
		1		1	1	
				1	1	0.90
				1	1	0.99
		2		3	2	0.47
				1	1	0.95
				1	1	0.87
				1	1	0.97
				1	1	0.94
				1	1	0.98
				1	1	0.94
				1	1	0.97
				1	1	0.98

				1	1	0.97
				1	1	0.98
				1	1	0.98
				1	1	0.98
				1	1	0.99
				1	1	0.98
		1		4	3	0.97
				1	1	#N/A
		1		1	1	
				1	1	#N/A
				1	1	0.97
	1	2		6	3	0.96
		1	1	2	2	0.96
		1		2	2	0.91
				1	1	0.94
				1	1	#N/A
				1	1	#N/A
				1	1	0.68
				2	2	0.95
		2		2	2	0.93
1	1	2	1	12	3	0.95
1		3	1	13	3	0.98
		3		5	3	0.97
1	1	3	1	14	3	0.94
				1	1	0.98
		2		3	2	0.33
	1	2		8	3	0.94
			1	2	2	0.17
1	1	3	1	14	3	0.98
1		2	1	6	3	0.95
				4	3	0.21
				2	2	#N/A
1		2		5	3	#N/A
		3		7	3	0.97
				1	1	0.93
				4	3	0.96
		2		4	3	0.98
				1	1	0.89
				2	2	0.75
				2	2	0.97
		2		2	2	0.95
1	1	3		12	3	0.96
		2		3	2	0.96
		1		3	2	0.95
1	1	3		11	3	0.97
				2	2	0.91
	1	3	1	12	3	0.91

				1	1	0.54
				1	1	0.91
				1	1	0.47
				1	1	0.93
				1	1	0.89
1		2	1	8	3	0.99
			1	2	2	0.68
		1		3	2	0.93
				3	2	0.97
				1	1	0.33
		2		4	3	0.99
				2	2	0.97
		1		4	3	0.98
				1	1	0.83
		2		3	2	0.99
				1	1	0.98
				1	1	0.98
				1	1	0.98
				2	2	0.95
				1	1	0.86
				1	1	0.83
		1	1	5	3	0.62
		1	1	4	3	0.48
		2		3	2	0.96
		2		3	2	0.97
				1	1	0.47
				1	1	0.96
				1	1	0.98
				1	1	0.97
				1	1	0.99
				1	1	0.98
				1	1	0.98
				1	1	0.98
				1	1	0.98
				1	1	0.97
				1	1	0.98
				1	1	0.98
		2		3	2	0.99
				1	1	0.97
				2	2	0.92
		1		2	2	0.86
				1	1	0.36
				1	1	0.94
	1	2		4	3	0.88
		2		2	2	#N/A
			1	1	1	#N/A

Comments_integrated	Oraganohalogen Flame	Organic non-halogenated I
fluorinated gaseous fire suppressant, not a flame retardant.		
physical-chemical property flag very low logKOW and very high water solubility. Many ubiqitic		
fail, this is a fire extinguisher not a flame retardant, also p-chem outside of range		
Physical-chemical properties, naturally occuring, used as intermediate, not a flame retardarda		
Flag for physical-chemical properties. High flash point, vapor pressure, and water solubiliy.		
This has 2 pubmed hits not as flame retardants. This is a fluorinated gaseous fire suppression a		
physical-chemical property flag very low logKOW and very high water solubility. Pharma uses,		
Naturally occuring. Highly reactive, only 1 bromine. Percent of mass is <50% p-chem propertie		
<50% Bromine, not likely FR		
Minor use as part of mixture in fire extinguishing agent. This is urine. Also can be added as syn		
Asbestos is not a flame retardant.		
This has 1 pubmed hit. No mention of Flame retardant in article, photolysis/atmospheric fate a		
flag for physical-chemical properties. Very low log KOW, high water solubility.		
PCBs are out of scope.		
PVC is a material type, not a flame retardant.		
Fluorinated gaseous fire suppressant/extinguisher. Do not think this is a flame retardant.		
Physical-chemical property flag. VP>10 and logKoW<1, may be a chemical intermediate		
only 1 data source, PUBCHEM has 4 hits for flame retardant in title BUT they are related to "Fl		
p-chem properties, too water soluble, likely not a FR.		
Appears on Table A7.1 “many of these substances appear not tobe flame retardants, but subst		
Physical form, not a flame retardant.		
Iodide flag, ineffective flame retardant.		
fiberglass is not a flame retardant		
flag for physical chemical properties, high flash point and vapor pressure.		
Dioxins are associated with and are breakdown products of flame retardants but are not flame		
fail- p/chem. Intermediate used to make other chemicals like flame retardants. Highly flamma		
This has 2 pubmed hits. Co-reported with flame retardants in article, PFOA is not a flame retar		
Appears on Table A7.1 “many of these substances appear not tobe flame retardants, but subst		
Flammable, naturally occuring not FR, <50% Br in total, Team of chemists this thinks this is not		
p-chem flag, likely intermediate, well-known precursorunintended presence in the product, if		
flag for physical chemcial properties, high flash point.		
Appears on Table A7.1 “many of these substances appear not tobe flame retardants, but subst		
Flag for iodide. Not effective as flame retardants. One of many possible tetrakis flame retarda		
flag for physical chemical properties, high water solubility, low log KoW.		
The source is pubmed hits associated with PBDEs. This chemical is combustable and has many		
p-chem flag, likely intermediate, well-known precursor unintended presence in the product, if		
flag-reactive. Highly soluble "water glass" mostly used as pH stabilizer		
flag for physical-chemical properties. High flash point and vapor pressure.		
Calcium on its own is highly reactive with water and air. Physical-chemical property flag. Other		
Natually occuring, <50% Br in total, highly reactive. Related to similar chemcials that are OFRs,		
	1	
	1	

	1	
	1	
	1	
	1	
	1	
polymeric		
polymeric		
		1
	1	
	1	
	1	
	1	
		1
	1	
		1
organic-non-halognated		1
		1
	1	
		1
		1
	1	
mixture		
	1	
	1	
polymeric		
	1	
	1	
	1	
OFR	1	
	1	
OFR	1	
OFR	1	
	1	
		1
	1	
	1	
inorganic		
polymeric		
	1	
	1	

	1	
	1	
	1	
	1	
	1	
	1	
	1	
	1	
	1	
	1	
	1	
	1	
	1	
inorganic		
	1	
	1	
	1	
	1	
	1	
	1	
		1
		1
inorganic		
		1
	1	
		1
	1	
		1
	1	
	1	
		1
	1	
organic-non-halognated		1
		1
OFR	1	
OFR	1	
		1
organic-non-halognated		1
OFR, old CAS number is 30496130 and wa	1	
	1	
non-halognated organic		1
		1

	1	
		1
inorganic		
Data sources 4 and 7 also reported old ger	1	
inorganic		
non-halognated organic		1
		1
		1
	1	
OFR	1	
	1	
	1	
	1	
		1
non-halognated organic		1
		1
		1
		1
	1	
	1	
		1
	1	
non-halognated organic		1
	1	
	1	
OFR	1	
	1	
		1
	1	
	1	
	1	
	1	
	1	
	1	
	1	
	1	
	1	
	1	
	1	
	1	
	1	
	1	

polymeric		
	1	
	1	
	1	
	1	
		1
	1	
	1	
	1	
		1
polymeric		
	1	
	1	
	1	
		1
	1	
		1
		1
	1	
		1
	1	
	1	
	1	
		1
		1
		1
OFR	1	
	1	
inorganic		
non-halognated organic		1
Chlorinated Paraffins Also mapped to DTX	1	
see supplemental NAS note	1	
	1	
	1	
		1
OFR	1	
		1
	1	
		1
	1	

	1	
		1
	1	
	1	
	1	
	1	
	1	
		1
	1	
This contains a halogen ion but not a carbon-halogen bond so it is		1
		1
	1	
	1	
	1	
	1	
	1	
	1	
	1	
	1	
	1	
	1	
	1	
	1	
	1	
	1	
	1	
	1	
	1	
	1	
	1	
		1
		1
		1
polymeric		
	1	
polymeric		
	1	
	1	
polymeric		
	1	
	1	
	1	
	1	
		1
	1	

	1	
	1	
organic-non-halognated		1
	1	
mixture of methylphenyl isomers; ill-defined substance		1
	1	
organic-non-halognated		1
OFR	1	
		1
	1	
		1
	1	
	1	
OFR	1	
	1	
		1
OFR	1	
	1	
		1
	1	
		1
		1
	1	
	1	
non-halognated organic		1
	1	
		1
	1	
		1
	1	
	1	
	1	
	1	
OFR	1	
Chlorinated Parrffin SCCP, see supplement	1	
	1	
	1	
	1	
	1	
	1	
	1	
	1	
	1	
	1	
	1	

	1	
	1	
	1	
	1	
	1	
	1	
	1	
	1	
	1	
This is an NAS chemical, previous CASRN w	1	
		1
	1	
Inorganic		
	1	
	1	
polymeric		
polymeric		
	1	
	1	
	1	
	1	
	1	
polymeric		
	1	
		1
	1	
		1
Ill-defined substance; mixture of isomeric tritolyl phosphates, usua		1
Inorganic		
	1	
	1	
		1
		1
		1
OFR	1	
organic-non-halognated		1
	1	
OFR	1	
	1	
	1	

Inorganic		
		1
	1	
	1	
	1	
	1	
		1
non-halognated organic		1
	1	
		1
	1	
		1
		1
	1	
	1	
	1	
	1	
	1	
	1	
	1	
	1	
inorganic		
		1
	1	
	1	
	1	
	1	
	1	
	1	
	1	
	1	
	1	
	1	
	1	
	1	
	1	
	1	
	1	
	1	
	1	
Ill-defined substance; [1522-92-5] Preferre<br>mixture	1	
	1	
This contains a Chloride ion but not a carbon-halogen bond so is n		1
	1	

	1	
	1	
	1	
	1	
	1	
cation/anion		1
polymeric		
	1	
		1
	1	
This contains a Chloride ion but not a carbon-halogen bond so it is		1
OFR	1	
OFR	1	
organic-non-halognated		1
	1	
inorganic		
	1	
	1	
polymeric		
non-halognated organic		1
OFR	1	
	1	
	1	
mixture		
		1
	1	
	1	
	1	
non-halognated organic		1
OFR	1	
	1	
	1	
OFR	1	
inorganic		
	1	
see supplemental NAS note	1	
non-halognated organic		1
inorganic		
		1
	1	
	1	

	1	
	1	
	1	
	1	
	1	
	1	
	1	
	1	
	1	
	1	
	1	
	1	
	1	
	1	
	1	
	1	
	1	
	1	
	1	
polymeric		
polymeric UVCB		
polymeric		
	1	
	1	
		1
	1	
mixture		
		1
		1
mixture, cation/anion Does not contain carbon-halogen bond		1
	1	
	1	
		1
organic-non-halognated		1
	1	
OFR	1	
	1	
	1	
	1	
OFR	1	
		1
		1
	1	
OFR	1	
non-halognated organic		1
		1

		1
		1
	1	
	1	
		1
	1	
	1	
	1	
	1	
	1	
	1	
	1	
OFR	1	
	1	
	1	
	1	
	1	
		1
inorganic		
polymeric		
	1	
	1	
	1	
	1	
	1	
	1	
	1	
	1	
	1	
	1	
	1	
	1	
	1	
	1	
	1	
	1	
Inorganic		
		1
polymeric		
	1	
This contains a Bromine ion but not a carbon-halogen bond so is n		1
	1	
polymeric		

		1
	1	
	1	
	1	
	1	
	1	
Containing triphenylphosphate, dicresyl xylenyl phosphate isomers		1
polymeric		
	1	
		1
	1	
		1
	1	
OFR	1	
	1	
	1	
	1	
	1	
	1	
		1
	1	
		1
	1	
		1
	1	
	1	
OFR	1	
OFR	1	
	1	
	1	
inorganic		
PUBCHEM has patents available that says this could be used as an		1
		1
	1	
	1	
	1	
		1

	1	
		1
	1	
	1	
	1	
	1	
		1
	1	
	1	
	1	
	1	
	1	
	1	
polymeric		
	1	
	1	
	1	
	1	
	1	
	1	
	1	
	1	
	1	
	1	
	1	
	1	
	1	
	1	
	1	
	1	
	1	
		1
OFR	1	
		1
	1	
	1	
		1
cation/anion		1
Handbook of Organophosphorus Chemistry, Robert Engel, 1992, CI		1
	1	
	1	
		1
		1

	1	
	1	
	1	
	1	
OFR	1	
OFR	1	
	1	
organic-non-halognated		1
	1	
OFR	1	
		1
		1
	1	
OFR	1	
	1	
OFR	1	
		1
	1	
non-halognated organic		1
organic-non-halognated		1
	1	
OFR, in the Dashboard, goes to 7436-90-0, PUBCHEM ID is 161726	1	
		1
	1	
	1	
non-halognated organic		1
	1	
	1	
	1	
OFR	1	
	1	
	1	
polymeric		
		1
	1	
	1	
	1	
	1	
	1	
	1	
	1	
	1	
	1	
	1	

	1	
	1	
	1	
	1	
	1	
	1	
	1	
	1	
	1	
	1	
	1	
	1	
	1	
	1	
	1	
	1	
		1
	1	
non-halognated organic		1
OFR	1	
OFR	1	
	1	
OFR	1	
	1	
	1	
OFR	1	
OFR	1	
mixture of isomeric xylene phosphates		1
	1	
polymeric		
	1	
		1
		1
	1	
	1	
see supplemental NAS note		1
OFR	1	
	1	
	1	
OFR	1	
	1	
OFR	1	

	1	
	1	
	1	
	1	
	1	
		1
		1
		1
	1	
	1	
	1	
	1	
	1	
	1	
	1	
	1	
	1	
	1	
		1
		1
polymeric		
polymeric		
	1	
	1	
	1	
	1	
	1	
	1	
	1	
	1	
	1	
	1	
	1	
	1	
	1	
	1	
	1	
	1	
	1	
	1	
	1	
		1
21447967	1	
23374640	1	

Polymeric Flame Retardan	Mixture OR Cation/Anion	Mixture OR Cation/Anion	Inorganic-Flame Retar
›us uses (mostly) buffer in cosmetics, but not a flame retardant use.			
nt.			
agent, remove.			
may be an intermediate, not a flame retardant use.			
s in range. Team of chemists note this is intermeidate/highly reactive flame retardant.			
1			1
ıergist but not a flame retardant on its own.			
article, not a flame retardant.			
lame retardanta AND intermediate" Team of chemists thinks this is not a flame retaradant			
:ances that are fire fighting agents, chemical intermediates or used forother purposes" If a chemical appe			
e retardants themselves			
ıble liquid/gas.			
rdant			
:ances that are fire fighting agents, chemical intermediates or used forother purposes" If a chemical appe			
t a flame retardant.			
present, Team of chemists thinks this is not a flame retardant			
:ances that are fire fighting agents, chemical intermediates or used forother purposes" If a chemical appe			
nts			
other non-flame retardant uses.			
present, Group thinks this is not a flame retardant			
r inorganic flame retardants contain calcium but calcium on its own is not a flame retardant			
but group thinks this is intermediate/highly reactive unlikely to be flame retardant.			
		1	

			1
1			
			1
1			
1			
			1
1		1	
1	1		
1			
			1
			1
			1
		1	
			1
1			

	1		1
			1
		1	
		1	1
	1		1
			1
			1
		1	
1			
			1
	1		1
			1
		1	

1

1
1

1

1 1

1
1
1

1

1

1

1

1

1

1 1

1

1

1

1

1

1

		1	
		1	
1			
		1	
1	1		
			1
		1	
			1
1			
1			
		1	
1			
		1	
		1	1

1

1

1

1

	1		1
			1
		1	
1			
1			
			1
1			
1			1
			1
			1
			1
			1
			1

1
1

1

1

1
1

1

1

1

	1		
1			
			1
	1		
			1
1			
		1	
1	1		
			1
			1
			1
			1
			1
			1
			1

		1	
1			
1			
1			
	1		1
		1	
1	1		
1			
	1		
			1

1

1
1

1

1
1
1
1
1
1

1

1

1

1

	1		
		1	
1			
		1	
	1		
1			
			1
		1	
			1
			1
			1
			1
			1
			1
			1
			1
			1

		1	
			1
			1
1		1	
		1	
	1		
		1	
		1	
	1		1
1			
			1
	1		

1

1

1

1

1

	1	
1		
	1	
	1	
		1
1		1
1		
		1
		1

1

1 1

1 1

1
1

1

Any Flame Retardant?	Manual remove_not FR?
	1
	1
	1
	1
	1
	1
	1
	1
11	1
	1
	1
	1
	1
	1
	1
	1
	1
	1
	1
	1
	1
	1
	1
	1
	1
	1
	1
	1
	1
	1
	1
	1
	1
	1
	1
	1
	1
	1
	1
	1
	1
1	0
1	0

1	0
1	0
1	0
1	0
1	0
1	0
1	0
1	0
1	0
11	0
1	0
1	0
1	0
1	0
1	0
1	0
1	0
1	0
1	0
1	0
1	0
1	0
1	0
1	0
1	0
1	0
1	0
1	0
1	0
1	0
1	0
1	0
1	0
1	0
1	0
1	0
1	0
1	0
1	0
1	0
1	0
1	0
1	0
1	0
1	0
1	0
1	0

1	0
1	0
1	0
1	0
1	0
1	0
1	0
1	0
1	0
1	0
1	0
1	0
1	0
1	0
1	0
1	0
1	0
11	0
1	0
1	0
1	0
11	0
1	0
1	0
1	0
1	0
11	0
1	0
1	0
1	0
1	0
1	0
1	0
1	0
1	0
1	0
11	0
1	0
1	0
1	0
1	0
1	0
1	0
1	0
1	0
1	0
	0

1	0
1	0
1	0
1	0
1	0
1	0
1	0
1	0
1	0
1	0
1	0
1	0
1	0
1	0
1	0
1	0
1	0
1	0
11	0
1	0
1	0
1	0
1	0
1	0
1	0
1	0
1	0
1	0
1	0
1	0
1	0
1	0
1	0
1	0
1	0
1	0
1	0
1	0
1	0
1	0
1	0
1	0
1	0
1	0
1	0
1	0
	0

1	0
1	0
1	0
1	0
1	0
1	0
1	0
1	0
11	0
1	0
1	0
1	0
11	0
1	0
1	0
1	0
1	0
1	0
1	0
1	0
1	0
1	0
1	0
1	0
1	0
1	0
1	0
1	0
1	0
1	0
1	0
1	0
1	0
1	0
1	0
1	0
1	0
1	0
1	0
1	0
1	0
1	0
1	0
1	0
1	0
1	0
1	0

1	0
1	0
1	0
1	0
1	0
1	0
1	0
11	0
1	0
11	0
1	0
1	0
1	0
1	0
1	0
1	0
1	0
1	0
1	0
1	0
1	0
1	0
1	0
1	0
1	0
1	0
1	0
1	0
1	0
1	0
1	0
1	0
1	0
1	0
1	0
1	0
1	0
1	0
1	0
1	0
1	0
1	0
1	0
1	0
11	0
1	0
1	0

1	0
1	0
1	0
1	0
1	0
1	0
1	0
1	0
1	0
1	0
1	0
1	0
1	0
1	0
1	0
1	0
1	0
1	0
1	0
1	0
1	0
1	0
1	0
1	0
1	0
1	0
1	0
1	0
1	0
1	0
1	0
1	0
1	0
1	0
1	0
1	0
1	0
1	0
1	0
1	0
1	0
1	0
1	0
1	0
1	0
1	0
1	0

1	0
1	0
1	0
1	0
1	0
1	0
1	0
1	0
1	0
1	0
1	0
1	0
1	0
1	0
1	0
1	0
1	0
1	0
1	0
1	0
1	0
1	0
1	0
1	0
1	0
11	0
1	0
1	0
1	0
1	0
1	0
1	0
1	0
1	0
1	0
1	0
1	0
1	0
1	0
1	0
1	0
1	0
1	0
1	0
1	0
1	0
1	0

1	0
1	0
1	0
1	0
1	0
1	0
1	0
1	0
1	0
1	0
1	0
1	0
1	0
1	0
1	0
1	0
1	0
1	0
1	0
1	0
1	0
1	0
1	0
11	0
1	0
1	0
1	0
1	0
1	0
1	0
1	0
1	0
1	0
1	0
1	0
1	0
1	0
1	0
1	0
1	0
1	0
1	0
1	0
1	0
1	0
1	0
1	0

1	0
1	0
1	0
1	0
1	0
1	0
1	0
1	0
1	0
1	0
1	0
1	0
1	0
1	0
1	0
1	0
1	0
1	0
1	0
1	0
1	0
1	0
1	0
1	0
1	0
1	0
1	0
1	0
1	0
1	0
1	0
1	0
1	0
1	0
1	0
1	0
1	0
1	0
1	0
1	0
1	0
1	0
1	0
1	0
1	0
1	0
1	0

1	0
1	0
1	0
1	0
1	0
1	0
1	0
1	0
1	0
1	0
1	0
1	0
1	0
1	0
1	0
1	0
1	0
1	0
1	0
1	0
1	0
1	0
1	0
1	0
11	0
1	0
1	0
1	0
11	0
1	0
1	0
1	0
1	0
1	0
1	0
1	0
1	0
1	0
1	0
1	0
1	0
1	0
1	0
1	0
1	0
1	0
1	0

1	0
1	0
1	0
1	0
1	0
1	0
1	0
1	0
1	0
1	0
1	0
1	0
1	0
1	0
1	0
1	0
1	0
1	0
1	0
1	0
1	0
11	0
1	0
1	0
1	0
1	0
1	0
1	0
1	0
1	0
1	0
1	0
1	0
1	0
1	0
1	0
1	0
1	0
1	0
1	0
1	0
1	0
1	0
1	0
1	0
1	0
1	0

1	0
1	0
1	0
1	0
1	0
1	0
1	0
1	0
1	0
1	0
1	0
1	0
1	0
1	0
1	0
1	0
1	0
1	0
1	0
1	0
1	0
1	0
1	0
1	0
1	0
1	0
1	0
1	0
1	0
1	0
1	0
1	0
1	0
1	0
1	0
1	0
1	0
1	0
1	0
1	0
1	0
1	0
1	0
1	0
1	0
1	0
1	0

1	0
1	0
1	0
1	0
1	0
1	0
1	0
1	0
1	0
1	0
1	0
1	0
1	0
1	0
1	0
1	0
1	0
1	0
1	0
1	0
1	0
1	0
1	0
1	0
1	0
1	0
1	0
1	0
1	0
1	0
1	0
1	0
1	0
1	0
1	0
1	0
1	0
1	0
1	0
11	0
1	0
1	0
1	0
1	0
1	0
1	0
1	0

1	0
1	0
1	0
1	0
1	0
1	0
1	0
1	0
1	0
1	0
1	0
1	0
1	0
1	0
1	0
1	0
1	0
1	0
1	0
1	0
1	0
1	0
1	0

1	0
1	0
1	0
1	0
1	0
1	0
1	0
1	0
1	0
1	0
1	0
1	0
1	0
1	0
1	0
1	0
1	0
1	0
1	0
1	0
1	0
1	0
1	0

1	0
1	0
1	0
1	0
1	0
1	0
1	0
1	0
1	0
1	0
1	0
1	0
1	0
1	0
1	0
1	0
1	0
1	0
1	0
1	0
1	0
1	0
1	0
1	0
1	0
1	0
1	0
1	0
1	0
1	0
1	0
11	0
1	0
1	0
1	0
1	0
1	0
1	0
1	0
1	0
1	0
1	0
1	0
1	0
1	0
1	0
1	0

1	0
1	0
1	0
1	0
1	0
1	0
11	0
1	0
1	0
1	0
1	0
1	0
1	0
1	0
1	0
1	0
1	0
1	0
1	0
111	0
1	0
1	0
1	0
1	0
1	0
1	0
1	0
1	0
1	0
1	0
1	0
1	0
1	0
1	0
1	0
1	0
1	0
1	0
1	0
1	0
1	0
1	0
1	0
1	0
1	0
1	0

Bergman Forward Search Chemicals

With links to documents

DSSToxID	Chemical Name
DTXSID1024627	1,2-Bis(2,4,6-tribromophenoxy)ethane
DTXSID1021952	Triphenyl phosphate
DTXSID1024128	Hexabromobenzene
DTXSID1026081	3,3',5,5'-Tetrabromobisphenol A
DTXSID4021391	Tris(methylphenyl) phosphate
DTXSID7027750	Dechlorane Plus
DTXSID5026259	Tris(2-chloroisopropyl)phosphate
DTXSID9026261	Tris(1,3-dichloro-2-propyl) phosphate
DTXSID5021411	Tris(2-chloroethyl) phosphate
DTXSID7021782	2,3,4,5,6-Pentabromoethylbenzene
DTXSID3025833	Pentabromotoluene
DTXSID6021959	2,4,6-Tribromophenol
DTXSID2024246	Pentabromodiphenyl ether
DTXSID2052732	1,1'-Ethane-1,2-diylbis(pentabromobenzene)
DTXSID70180181	Cyclodecane, hexabromo
DTXSID8024236	Octabromodiphenyl ether
DTXSID9052686	2-Ethylhexyl 2,3,4,5-tetrabromobenzoate
DTXSID7027887	Bis(2-ethylhexyl) tetrabromophthalate
DTXSID5021758	Tris(2-butoxyethyl) phosphate
DTXSID1025300	2-Ethylhexyl diphenyl phosphate
DTXSID3044918	1,3,5-Tribromo-2-(prop-2-en-1-yloxy)benzene
DTXSID3021986	Tributyl phosphate
DTXSID8024947	1,2-Dibromo-4-(1,2-dibromoethyl)cyclohexane
DTXSID0021414	Tris(2-ethylhexyl) phosphate
DTXSID80866227	7,8-Dibromo-1,2,3,4,11,11-hexachloro-1,4,4a,5,6,7,8,9,10,1
DTXSID3030056	2,2',4,4'-Tetrabromodiphenyl ether
DTXSID00904130	2-bromoallyl 2,4,6-tribromophenyl ethe
DTXSID4052689	2,2',4,4',6-Pentabromodiphenyl ether
DTXSID0052750	1,2,5,6-Tetrabromocyclooctane
DTXSID4030047	2,2',4,4',5,5'-Hexabromodiphenyl ether
DTXSID8052693	2,2',3,4,4',5',6-Heptabromodiphenyl ether
DTXSID4052712	Tripropyl phosphate
DTXSID4027527	1,2,5,6,9,10-Hexabromocyclododecane
DTXSID8052697	2,3,5,6-Tetrabromo-p-xylene
DTXSID9052719	(Pentabromophenyl)methyl acrylate
DTXSID50192686	2,3,4,5-Tetrabromo-6-chlorotoluene
DTXSID30858238	4,5,6,7-Tetrabromo-1,1,3-trimethyl-3-(2,3,4,5-tetrabromop
DTXSID00214802	Tris[2-(propan-2-yl)phenyl] phosphate
DTXSID1052290	2,4-Dibromophenol
DTXSID0058691	Tris(tribromoneopentyl)phosphate
DTXSID00959387	1,2,3,4,5-Pentabromo-6-(bromomethyl)benzene

DTXSID3051466	Tris(4-tert-butylphenyl) phosphate
DTXSID8069197	Tetraphenyl m-phenylene bis(phosphate)
DTXSID9022079	Pentabromophenol
DTXSID5063071	Allyl pentabromophenyl ether
DTXSID9020164	Pentaerythritol dibromide
DTXSID9024641	3-Bromo-2,2-bis(bromomethyl)propanol
DTXSID8073471	Benzene, dibromoethenyl-
DTXSID3052698	Tripentyl phosphate
DTXSID4025828	Pentabromochlorocyclohexane
DTXSID0060561	Phenol, 2,6-dibromo-
DTXSID80877030	BDE-77
DTXSID3027615	Tris(2-chloropropyl) phosphate
DTXSID201016652	Tris(chloropropyl)phosphate
DTXSID501016863	syn-Dechlorane Plus
DTXSID201016864	anti-Dechlorane Plus
DTXSID5052705	1,3,5-Tribromo-2-(2,3-dibromopropoxy)benzene
DTXSID9020376	1,1'-Oxybis[2,3,4,5,6-pentabromobenzene]
DTXSID8040698	Triisobutyl phosphate
DTXSID9030048	2,2',4,4',5-Pentabromodiphenyl ether
DTXSID3052692	2,2',4,4',5,6'-Hexabromodiphenyl ether
DTXSID4052710	2,4,4'-Tribromodiphenyl ether
DTXSID4052685	2,2',3,4,4'-Pentabromodiphenyl ether
DTXSID4038922	Tetrabromobisphenol A bis(2-hydroxyethyl) ether
DTXSID9052688	2,3',4,4'-Tetrabromodiphenyl ether
DTXSID9065572	1,1'-Biphenyl, 2,2',3,3',4,4',5,5',6,6'-decabromo-
DTXSID9074775	BDE-197
DTXSID20108830	1H-Indene, 2,3-dihydro-1,1,3-trimethyl-3-phenyl-, octabron
DTXSID7074749	BDE-203
DTXSID3074789	BDE-196
DTXSID0021173	Firemaster FF-1
DTXSID70218014	1,1'-Biphenyl, 2,2',3,4,4',5,5'-heptabromo-
DTXSID501016675	(+)-alpha-Hexabromocyclododecane
DTXSID30467127	(+)-beta-Hexabromocyclododecane
DTXSID701016671	(+)-gamma-Hexabromocyclododecane
DTXSID0075108	1,1'-Biphenyl, 3,3',5,5'-tetrabromo-
DTXSID9074771	PBB 103
DTXSID0052702	1,2,3,4,6,7,8,9,10,10,11,11-dodecachloro-1,4,4a,5a,6,9,9a,[illegible]
DTXSID30108225	1,4:5,8:9,10-Trimethanoanthracene, 1,2,3,4,5,6,7,8,12,12,1[illegible]
DTXSID7020895	Mirex
DTXSID40929084	1,2,3,4,5,6,7,8,10,10,11,11-Dodecachloro-4,4a,4b,5,8,8a,9,[illegible]
DTXSID301016700	Dechloran 604
DTXSID70675901	4,5,6,7,13,14,15,16,19,19,20,20-Dodecachloroheptacyclo[9
DTXSID8026228	Triethyl phosphate
DTXSID90209646	Benzene, pentabromo-
DTXSID5021413	Tris(2,3-dibromopropyl) phosphate
DTXSID6032192	Tri-o-cresyl phosphate
DTXSID3032129	Tetrabromobisphenol A-bis(2,3-dibromopropyl ether)

DTXSID5052676	Tris(4-methylphenyl) phosphate
DTXSID8028000	Phosphoric acid, 2,2-bis(chloromethyl)-1,3-propanediyl tetr
DTXSID4026216	Tri-m-tolyl phosphate
DTXSID9052713	1,3,5-Tris(2,3-dibromopropyl)-1,3,5-triazine-2,4,6(1H,3H,5H
DTXSID3024861	Cresyl diphenyl phosphate
DTXSID1021403	Trimethyl phosphate
DTXSID8052720	Phosphoric acid, P,P'-[(1-methylethylidene)di-4,1-phenylen
DTXSID2029327	Tetrabromobisphenol A diallyl ether
DTXSID0058695	2,4,6-Tris-(2,4,6-tribromophenoxy)-1,3,5-triazine
DTXSID10865889	Tetrabromobisphenol A dimethyl ether
DTXSID60864948	2-(2-Hydroxyethoxy)ethyl 2-hydroxypropyl 3,4,5,6-tetrabro
DTXSID3025465	Isodecyl diphenyl phosphate
DTXSID6024626	1,2-Bis(tetrabromophthalimido)ethane
DTXSID70956080	1,2,3,4,7,7-Hexachloro-5-(pentabromophenyl)bicyclo[2.2.1]
DTXSID6044563	6H-Dibenzo[c,e][1,2]oxaphosphinine 6-oxide
DTXSID0052708	4,4'-Sulphonylbis[2,6-dibromophenol]
DTXSID5044942	1,1'-Sulfonylbis[3,5-dibromo-4-(2,3-dibromopropoxy)benze
DTXSID8047507	Perbromo-1,4-diphenoxybenzene
DTXSID60108450	Phosphoric acid, P,P'-1,3-phenylene P,P,P',P'-tetrakis(2,6-di
DTXSID7023805	Tris(2,3-dichloropropyl)phosphate
DTXSID20873773	(+/-)-gamma-Hexabromocyclododecane
DTXSID00873771	(+/-)-α-Hexabromocyclododecane
DTXSID10274134	Tris(3,5-xylenyl)phosphate
DTXSID90199295	Triisopropyl phosphate
DTXSID60873772	(+/-)-beta-Hexabromocyclododecane
DTXSID70728087	1,2,3,4,5-Pentabromo-6-(chloromethyl)benzene
DTXSID60872265	2,2',3,4,4',5'-Hexabromodiphenyl Ether
DTXSID50873428	Dibutyl phenyl phosphate
DTXSID1062623	Butyl diphenyl phosphate
DTXSID901016677	4,5,6,7-tetrabromo-1,1,3-trimethyl-3-(2,3,4,6-tetrabromop
DTXSID3051543	4-Bromophenol
DTXSID0052700	2,2'-[(1-Methylethylidene)bis[(2,6-dibromo-4,1-phenylene)
DTXSID30885564	Phenol, 4,4'-(1-methylethylidene)bis[2,6-dibromo-, 1,1'-dia
DTXSID70867243	Bis(p-acryloxyethoxy)tetrabromobisphenol A
DTXSID30573656	1,1'-[Oxybis(methylene)]bis(pentabromobenzene)
DTXSID601016680	2,4,5,6,7-pentabromo-1,1,3-trimethyl-3-(2,4,6-tribromophe
DTXSID3021770	2,2',6,6'-Tetrachlorobisphenol A
DTXSID6020802	Melamine
DTXSID001016668	3,3',5,5'-Tetrabromobisphenol A bispropionate
DTXSID90963559	Phenyl propan-2-yl hydrogen phosphate
DTXSID60970533	(Propane-2,2-diyl)bis(2,6-dibromo-4,1-phenylene) diprop-2-
DTXSID001016694	1-(2,3-dibromopropyl)-3,5-di(prop-2-en-1-yl)-1,3,5-triazinar
DTXSID501016699	1,1'-sulfonylbis(3,5-dibromo-4-methoxybenzene)
DTXSID001016701	1,3-bis(2,3-dibromopropyl)-5-(prop-2-en-1-yl)-1,3,5-triazina
DTXSID40872703	2,2',4-Tribromodiphenyl ether
DTXSID90873922	2,3',4',6-Tetrabromodiphenyl Ether
DTXSID90873927	2,2',4,5'-Tetrabromodiphenyl Ether

DTXSID9070406	2,2-Bis(bromomethyl)-3-chloropropyl bis[2-chloro-1-(chlor
DTXSID9026267	Trixylyl phosphate
DTXSID2020688	Hexachlorocyclopentadiene
DTXSID2026941	Chlorendic anhydride
DTXSID2020268	Chlorendic acid
DTXSID7026102	Tetrachlorophthalic anhydride
DTXSID1044699	Diethyl hydrogen phosphate
DTXSID5025150	Dimethyl phosphate
DTXSID80862522	Tris(3-chloropropyl)phosphate
DTXSID80858783	Tris(4-isopropylphenyl) phosphate
DTXSID1074597	Phosphoric acid, trihexyl ester
DTXSID70275889	Bis(2,3-dibromopropyl) hydrogen phosphate
DTXSID3028269	Bis(tert-butylphenyl)phenyl phosphate
DTXSID701016683	2,4,6-tribromo-3-(tetrabromopentadecyl)-phenol
DTXSID20893653	2,2-Bis(chloromethyl)-1,3-propanediyl tetrakis(1-chloro-2-p
DTXSID9047548	1,1'-Oxybis(2,3,4,5-tetrabromobenzene)
DTXSID40556652	2,2',3,3',4,5,5',6,6'-nonabromodiphenyl ether
DTXSID30451985	BDE-207
DTXSID90879984	BDE-201
DTXSID00196255	2,2',3,4,4',5,6,6'-Octabromodiphenyl ether
DTXSID9029855	Phosphoric trichloride, polymer with 1,3-benzenediol, phen
DTXSID6024701	tert-Butylphenyl diphenyl phosphate
DTXSID30881107	1,2,3,4,5-Pentabromo-6-(2,3,4,5-tetrabromophenoxy)benz
DTXSID4028880	Triphenyl phosphates isopropylated
DTXSID1027134	Bis(2-ethylhexyl) phosphate
DTXSID8025383	Hexabromocyclododecane
DTXSID30992969	Bis(1,3-dichloropropan-2-yl) hydrogen phosphate
DTXSID50448655	Benzene, 1,2,3,5-tetrabromo-4-(2,4,6-tribromophenoxy)-
DTXSID1059072	1,1'-Biphenyl, 4,4'-dibromo-
DTXSID1048207	Diphenyl phosphate
DTXSID1020142	6-Phenyl-1,3,5-triazine-2,4-diamine
DTXSID8052641	2-Bromophenol
DTXSID2026943	Pentaerythritol
DTXSID8074287	Octyl diphenyl phosphate
DTXSID0026258	Tris(2-chloroethyl) phosphite
DTXSID5029186	6-Methyl-1,3,5-triazine-2,4-diamine
DTXSID9060449	Phenol, 3-bromo-
DTXSID3060950	1,3,5-Triazin-2(1H)-one, 4,6-diamino-
DTXSID2022121	Triphenylphosphine oxide
DTXSID2052738	Tetraethyl ethylenebisphosphonate
DTXSID1051802	Dibutyl chlorendate
DTXSID60199202	s-Triazine, 2,2'-O-phenylene-bis(4,6-diamino-
DTXSID20210629	Dioctyl phenyl phosphate
DTXSID6025484	Isopropyl phenyl diphenyl phosphate
DTXSID7075212	Phosphoric acid, dimethylphenyl diphenyl ester
DTXSID2074110	2-tert-Butylphenyl diphenyl phosphate
DTXSID9051957	Bis(isopropylphenyl) phenyl phosphate

DTXSID3024233	Nonabromodiphenyl ether
DTXSID50879985	BDE-202
DTXSID80872267	2,3,3',4,4',5,6-Heptabromodiphenyl Ether
DTXSID4074774	PBDE 195
DTXSID30879988	BDE-198
DTXSID4074776	PBDE 205
DTXSID50858795	Tris(isopropylphenyl) phosphate
DTXSID3068043	1,3,5-Triazine-2,4,6(1H,3H,5H)-trione, compd. with 1,3,5-tri
-	-
DTXSID60886428	Benzene, 1,1'-[1,2-ethanediylbis(oxy)]bis[2,3,4,5,6-pentabr
DTXSID1060558	2,4,6-Tribromoanisole
DTXSID3052307	1,3,5-Tribromobenzene
DTXSID9024015	4,4'-Dibromodiphenyl ether
DTXSID50873928	2,3',4,4',6-Pentabromodiphenyl Ether
DTXSID50573491	1,2,3-Tribromo-5-(3,4-dibromophenoxy)benzene
DTXSID20786910	1,2,3,4-Tetrabromo-5-(3,4-dibromophenoxy)benzene
DTXSID201009900	2,2',5,5'-Tetrabromobiphenyl
DTXSID50218113	1,1'-Biphenyl, 2,2',4,5,5'-pentabromo-
DTXSID70274039	Diphenyl 4-tolyl phosphate
DTXSID1049697	o-Terphenyl
DTXSID2029117	1,1':3',1''-Terphenyl
DTXSID4024640	4-Bromobiphenyl
DTXSID6029121	1,1':4',1''-Terphenyl
DTXSID20400487	diethylphosphinic acid
DTXSID5024639	3-Bromobiphenyl
DTXSID6025800	1,2,3,4,5,6,7,8-Octachloronaphthalene
DTXSID40185608	p-Tetradecachloroterphenyl
DTXSID70195215	1,1':2',1''-Terphenyl, 2,2'',3,3',3'',4,4',4'',5,5',5'',6,6',6''-tetra
DTXSID30195216	1,1':3',1''-Terphenyl, 2,2',2'',3,3'',4,4',4'',5,5',5'',6,6',6''-tetra
DTXSID30972327	1,2,3,4,7,7-Hexachloro-5-(2,4,6-tribromophenyl)bicyclo[2.2
DTXSID00207815	2,3,5,6,2',3',5',6'-Octabromobiphenyl
DTXSID20219339	1,1'-Biphenyl, 2,2',3,3',4,4',5,6'-octabromo-
DTXSID30219340	2,2',3,3',4,4',5,5',6-Nonabromo-1,1'-biphenyl
DTXSID80220168	2,2',3,3',4,5,5',6'-Octabromobiphenyl
DTXSID40858926	2,2',3,3',4,4'-Hexabromo-1,1'-biphenyl
DTXSID60152372	1,1'-Biphenyl, 2,2',3,3',4,4',6,6'-octabromo-
DTXSID20152373	2,2',3,3',4,5',6,6'-Octabromobiphenyl
DTXSID80152374	2,2',3,4,4',5,6,6'-Octabromobiphenyl
DTXSID40873533	2,2',3,3',4,4',5,6,6'-Nonabromo-1,1'-biphenyl
DTXSID40152375	2,2',3,3',4,5,5',6,6'-Nonobromobiphenyl
DTXSID5024348	2',3,4-Tribromodiphenyl ether
DTXSID70879982	BDE-199
DTXSID30879983	BDE-200
DTXSID80107228	Phosphoric trichloride, reaction products with bisphenol A a
DTXSID50950561	Tris(2,4-dibromophenyl) phosphate
DTXSID10923559	Bromine--methane (1/16)
DTXSID8035681	Ammonium bromide

DTXSID00164338	4,4',6,6'-Tetrabromo-2,2'-biphenyldiol
DTXSID90889689	1,2-Benzenedicarboxylic acid, 3,4,5,6-tetrabromo-, sodium
DTXSID60872801	2,2-Bis(3,5-dibromo-4-hydroxyphenyl)propane-phosgene co
DTXSID401012987	Carbonic dichloride, polymer with 4,4'-(1-methylethylidene
DTXSID50957474	1,3,5-Tribromo-2-(2,3-dibromo-2-methylpropoxy)benzene
DTXSID10892231	3,5,3',5'-Tetrabromobisphenol A, epichlorohydrin polymer
DTXSID9052711	Tris(dibromophenyl) phosphate
DTXSID20452860	1,3,5-Triazine, 2,4,6-tris(2,3-dibromopropoxy)-
DTXSID6052168	N,N'-(Ethylene)bis[4,5-dibromohexahydro-3,6-methanopht
DTXSID301016693	Poly(tribromostyrene)
DTXSID70872796	Poly(pentabromobenzyl acrylate)
DTXSID2069365	1H-Pyrrole-2,5-dione, 1-(2,4,6-tribromophenyl)-
DTXSID6098467	1,3-Butadiene, homopolymer, brominated
DTXSID90872793	2,2'-[(1-Methylethylidene)bis[(dibromo-4,1-phenylene)oxyr
DTXSID50988682	1-Bromo-4-chlorodecane
DTXSID90872798	2,4-(or 2,6)-Dibromophenol, homopolymer
DTXSID00872800	Phenol, 4,4'-(1-methylethylidene)bis[2,6-dibromo-, polyme
DTXSID1029049	Tetrabromophthalic acid mixed esters with diethylene glycc
DTXSID501000614	1~2~,1~3~,1~4~,1~5~,1~6~,2~3~,2~4~,3~2~,3~3~,3~4~,3~5
DTXSID60232825	2,2',3,3',5,5',6,6'-Octabromo-4-phenoxy-1,1'-biphenyl
DTXSID601004590	2-Bromoethyl 5-bromopentyl 2-chloroethyl phosphate
DTXSID30105330	Benzene, ethenyl-, homopolymer, brominated
DTXSID601015670	Carbonic dichloride, polymer with 4,4'-(1-methylethylidene
DTXSID80893715	1,1'-(Isopropylidene)bis(3,5-dibromo-4-(2,3-dibromo-2-met
DTXSID1074252	1-Propanol, 3,3'-oxybis[2,2-bis(bromomethyl)-
DTXSID1029095	Phosphoric acid, mixed 3-bromo-2,2-dimethylpropyl and 2-
DTXSID10872775	Brominated epoxy resin end-capped with tribromophenol
DTXSID001017111	End capped and partially end capped brominated epoxy olig
DTXSID80872782	4,4'-(1-Methylethylidene)bis[2,6-dibromophenol] polymer w
DTXSID20108653	Benzene, ethenyl-, polymer with 1,3-butadiene, brominated
DTXSID201016688	Piperazine, phosphate (1:?)
DTXSID7065912	Diphosphoric acid, compd. with 1,3,5-triazine-2,4,6-triamin
DTXSID901016689	Tris(dichloropropyl) phosphate
DTXSID9068328	1,3,5-Triazine-2,4,6-triamine, phosphate
DTXSID10886791	Diphosphoric acid, compd. with piperazine (1:1)
DTXSID40105260	C10-13 chloro alkanes
DTXSID2040313	Cercelor S 52
DTXSID20894685	Phosphoric acid, bis(methylphenyl) phenyl ester
DTXSID901016691	1,1'-{[(2Z)-2,3-dibromobut-2-ene-1,4-diyl]bis(oxy)}bis(2,4,6-
DTXSID40893054	Tribromoneopentyl alcohol
DTXSID2029458	Bromo chloro C12-30 a-alkenes
DTXSID7028695	Alkenes, C12-24, chloro
DTXSID001002372	Bis[(pentabromophenyl)methyl] 3,4,5,6-tetrabromobenzen
DTXSID70849024	Bis[(pentabromophenyl)methyl] benzene-1,4-dicarboxylate
DTXSID501016706	(rel)-(1R,2R,3S,4S)-1,2,3,9-tetrabromo-1,2,3,4-tetrahydro-1
DTXSID201016707	(rel)-(1R,2S,3S,4S)-1,2,3,9-tetrabromo-1,2,3,4-tetrahydro-1,
DTXSID60174332	3-Bromostyrene

DTXSID8043781	Tetrabromophthalic acid
DTXSID60162597	2,3,4,6-Tetrabromophenol
DTXSID201016690	2,3,4,5-Tetrabromobenzoic Acid
DTXSID20545726	2,3,4,5-Tetrabromophenol
DTXSID40872768	Tetraammonium octamolybdate
DTXSID0020494	Dimethyl methylphosphonate
DTXSID4049662	Magnesium hydroxide
DTXSID4023880	Antimony trioxide
DTXSID50872597	Tin zinc hydroxide (ZnSn(OH)6)
DTXSID5065196	Tin zinc oxide (SnZnO3)
DTXSID2036405	Aluminum hydroxide
DTXSID3097842	Ammonium polyphosphates
DTXSID40872788	Melapur 200
DTXSID00872789	Aluminum diethylphosphinate
DTXSID60919462	Tris(3-methylbutyl) phosphate
DTXSID60550652	1,1'-Oxybis(2,3,4-tribromobenzene)
DTXSID7025051	Diethyl ethylphosphonate
DTXSID40174330	4-Bromostyrene
DTXSID6023028	Etidronic acid
DTXSID4063290	1,3,2-Dioxaphosphorinane, 2,2'-oxybis[5,5-dimethyl-, 2,2'-d
DTXSID001016751	Octabromobiphenyl
DTXSID80904259	4,4'-(propane-2,2-diyl)bis(2-bromophenol)
DTXSID70858838	2,2',4,4',5,5'-Hexabromobiphenyl
DTXSID10208772	3,3',4,4',5,5'-Hexabromobiphenyl
DTXSID60229856	3,5-Dibromo-2-(2,4-dibromophenoxy)phenol
DTXSID2061149	Tributylphosphine oxide
DTXSID1061946	Phosphonic acid, P-phenyl-, dioctyl ester
DTXSID00181969	Ethanol, 2-bromo-, phosphate (3:1)
DTXSID40412148	Benzene, pentabromo(2,3-dibromopropoxy)-
DTXSID00957040	Tris(2-bromo-4-methylphenyl) phosphate
DTXSID6044939	1,3,5-Tribromo-2-methoxy-4-methylbenzene
DTXSID4051665	2-Biphenylyl diphenyl phosphate
DTXSID6051770	Bis(2-ethylhexyl) phenyl phosphate
DTXSID7073486	Benzene, bromophenoxy-
DTXSID00477016	Benzene, 1,3-dibromo-2-phenoxy-
DTXSID60477017	2,4,6-Tribromodiphenyl ether
DTXSID00199847	2,4-Dichlorodiphenyl ether
DTXSID40873972	BDE-180
DTXSID7060910	1,2,4,5-Tetrabromobenzene
DTXSID8023927	p-Bromodiphenyl ether
DTXSID50577712	1-Bromo-2-(4-bromophenoxy)benzene
DTXSID10704805	1,2,3,5-Tetrabromo-4-(3,4,5-tribromophenoxy)benzene

	Links:	[24016281]	[30223335]	[23185960]	[33466958]
**Query**	**CAS**	**A**	**B**	**C**	**D**
1,2-Bis(2,4,6-tribromophenoxy)etl	37853591	1	1	1	
Triphenyl phosphate	115866	1		1	
Hexabromobenzene	87821		1	1	
3,3',5,5'-Tetrabromobisphenol A	79947	1		1	
Tris(methylphenyl) phosphate	1330785	1		1	
Dechlorane Plus	13560899				1
Tris(2-chloroisopropyl)phosphate	13674845	1			
Tris(1,3-dichloro-2-propyl) phosph	13674878				
Tris(2-chloroethyl) phosphate	115968	1			
2,3,4,5,6-Pentabromoethylbenzer	85223		1		
Pentabromotoluene	87832		1		
2,4,6-Tribromophenol	118796				
Pentabromodiphenyl ether	32534819				
1,1'-Ethane-1,2-diylbis(pentabrom	84852539			1	
Cyclodecane, hexabromo	25495981				
Octabromodiphenyl ether	32536520				
2-Ethylhexyl 2,3,4,5-tetrabromobe	183658277	1	1	1	
Bis(2-ethylhexyl) tetrabromophtha	26040517	1		1	
Tris(2-butoxyethyl) phosphate	78513	1		1	
2-Ethylhexyl diphenyl phosphate	1241947	1		1	
1,3,5-Tribromo-2-(prop-2-en-1-ylc	3278895	1	1	1	
Tributyl phosphate	126738	1		1	
1,2-Dibromo-4-(1,2-dibromoethyl)	3322938		1	1	
Tris(2-ethylhexyl) phosphate	78422	1		1	
7,8-Dibromo-1,2,3,4,11,11-hexach	51936551	1	1	1	
2,2',4,4'-Tetrabromodiphenyl ethe	5436431	1	1	1	
2-bromoallyl 2,4,6-tribromopheny	99717563	1	1	1	
2,2',4,4',6-Pentabromodiphenyl et	189084648		1	1	
1,2,5,6-Tetrabromocyclooctane	3194578		1	1	
2,2',4,4',5,5'-Hexabromodiphenyl	68631492	1	1	1	
2,2',3,4,4',5',6-Heptabromodipher	207122165	1	1	1	
Tripropyl phosphate	513086			1	
1,2,5,6,9,10-Hexabromocyclodode	3194556			1	
2,3,5,6-Tetrabromo-p-xylene	23488382		1		
(Pentabromophenyl)methyl acryla	59447551				
2,3,4,5-Tetrabromo-6-chlorotolue	39569216		1		
4,5,6,7-Tetrabromo-1,1,3-trimeth	1084889519				
Tris[2-(propan-2-yl)phenyl] phospl	64532952				
2,4-Dibromophenol	615587				
Tris(tribromoneopentyl)phosphat	19186971				
1,2,3,4,5-Pentabromo-6-(bromom	38521516				

Tris(4-tert-butylphenyl) phosphate	78331				
Tetraphenyl m-phenylene bis(pho	57583547				
Pentabromophenol	608719				
Allyl pentabromophenyl ether	3555111				
Pentaerythritol dibromide	3296900				
3-Bromo-2,2-bis(bromomethyl)pr	1522925				
Benzene, dibromoethenyl-	7436900				
Tripentyl phosphate	2528383				
Pentabromochlorocyclohexane	87843				
Phenol, 2,6-dibromo-	608333				
BDE-77	93703481				
Tris(2-chloropropyl) phosphate	6145739				
Tris(chloropropyl)phosphate	26248873				
syn-Dechlorane Plus	135821033		1	1	1
anti-Dechlorane Plus	135821748		1	1	1
1,3,5-Tribromo-2-(2,3-dibromopr	35109605		1	1	
1,1'-Oxybis[2,3,4,5,6-pentabromo	1163195	1		1	
Triisobutyl phosphate	126716	1		1	
2,2',4,4',5-Pentabromodiphenyl et	60348609	1	1	1	
2,2',4,4',5,6'-Hexabromodiphenyl	207122154		1	1	
2,4,4'-Tribromodiphenyl ether	41318756	1	1	1	
2,2',3,4,4'-Pentabromodiphenyl et	182346210		1	1	
Tetrabromobisphenol A bis(2-hyd	4162452			1	
2,3',4,4'-Tetrabromodiphenyl ethe	189084615		1	1	
1,1'-Biphenyl, 2,2',3,3',4,4',5,5',6,6	13654096			1	
BDE-197	117964213			1	
1H-Indene, 2,3-dihydro-1,1,3-trim	155613937			1	
BDE-203	337513721	1		1	
BDE-196	446255396			1	
Firemaster FF-1	67774327			1	
1,1'-Biphenyl, 2,2',3,4,4',5,5'-hept	67733522			1	
(+)-alpha-Hexabromocyclododeca	138257199	1		1	
(+)-beta-Hexabromocyclododecan	678970166	1		1	
(+)-gamma-Hexabromocyclodode	678970177	1		1	
1,1'-Biphenyl, 3,3',5,5'-tetrabrom	16400503			1	
PBB 103	59080396			1	
1,2,3,4,6,7,8,9,10,10,11,11-dodec	31107445				1
1,4:5,8:9,10-Trimethanoanthrace	13560924				1
Mirex	2385855				1
1,2,3,4,5,6,7,8,10,10,11,11-Dodec	13560913				1
Dechloran 604	71245277				1
4,5,6,7,13,14,15,16,19,19,20,20-D	13560902				1
Triethyl phosphate	78400				
Benzene, pentabromo-	608902		1		
Tris(2,3-dibromopropyl) phosphat	126727				
Tri-o-cresyl phosphate	78308				
Tetrabromobisphenol A-bis(2,3-di	21850442				

Tris(4-methylphenyl) phosphate	78320	
Phosphoric acid, 2,2-bis(chlorome	38051104	
Tri-m-tolyl phosphate	563042	
1,3,5-Tris(2,3-dibromopropyl)-1,3,	52434909	
Cresyl diphenyl phosphate	26444495	
Trimethyl phosphate	512561	
Phosphoric acid, P,P'-[(1-methylet	5945335	
Tetrabromobisphenol A diallyl eth	25327893	
2,4,6-Tris-(2,4,6-tribromophenoxy	25713604	
Tetrabromobisphenol A dimethyl (	37853615	
2-(2-Hydroxyethoxy)ethyl 2-hydro	20566352	
Isodecyl diphenyl phosphate	29761215	
1,2-Bis(tetrabromophthalimido)et	32588764	
1,2,3,4,7,7-Hexachloro-5-(pentabr	34571169	
6H-Dibenzo[c,e][1,2]oxaphosphini	35948255	1
4,4'-Sulphonylbis[2,6-dibromophe	39635795	
1,1'-Sulfonylbis[3,5-dibromo-4-(2,:	42757551	
Perbromo-1,4-diphenoxybenzene	58965665	
Phosphoric acid, P,P'-1,3-phenyler	139189303	
Tris(2,3-dichloropropyl)phosphate	78433	
(+/-)-gamma-Hexabromocyclodod	134237528	
(+/-)-α-Hexabromocyclododecane	134237506	
Tris(3,5-xylenyl)phosphate	25653161	
Triisopropyl phosphate	513020	
(+/-)-beta-Hexabromocyclododeca	134237517	
1,2,3,4,5-Pentabromo-6-(chlorom	58495093	
2,2',3,4,4',5'-Hexabromodiphenyl	182677301	
Dibutyl phenyl phosphate	2528361	
Butyl diphenyl phosphate	2752956	
4,5,6,7-tetrabromo-1,1,3-trimethy	893843077	
4-Bromophenol	106412	
2,2'-[(1-Methylethylidene)bis[(2,6	3072842	
Phenol, 4,4'-(1-methylethylidene)l	33798026	
Bis(p-acryloxyethoxy)tetrabromob	66710972	
1,1'-[Oxybis(methylene)]bis(penta	497107138	
2,4,5,6,7-pentabromo-1,1,3-trime	1025956653	
2,2',6,6'-Tetrachlorobisphenol A	79958	
Melamine	108781	
3,3',5,5'-Tetrabromobisphenol A b	37419424	
Phenyl propan-2-yl hydrogen phos	46355071	
(Propane-2,2-diyl)bis(2,6-dibromo	55205384	
1-(2,3-dibromopropyl)-3,5-di(prop	57829897	
1,1'-sulfonylbis(3,5-dibromo-4-me	70156795	
1,3-bis(2,3-dibromopropyl)-5-(pro	75795163	
2,2',4-Tribromodiphenyl ether	147217752	
2,3',4',6-Tetrabromodiphenyl Ethe	189084626	
2,2',4,5'-Tetrabromodiphenyl Ethe	243982823	

2,2-Bis(bromomethyl)-3-chloropro	66108370	
Trixylyl phosphate	25155231	
Hexachlorocyclopentadiene	77474	
Chlorendic anhydride	115275	
Chlorendic acid	115286	
Tetrachlorophthalic anhydride	117088	
Diethyl hydrogen phosphate	598027	
Dimethyl phosphate	813785	
Tris(3-chloropropyl)phosphate	1067987	
Tris(4-isopropylphenyl) phosphate	2502150	
Phosphoric acid, trihexyl ester	2528394	
Bis(2,3-dibromopropyl) hydrogen	5412259	
Bis(tert-butylphenyl)phenyl phosp	65652417	
2,4,6-tribromo-3-(tetrabromopent	168434455	
2,2-Bis(chloromethyl)-1,3-propane	1047637375	
1,1'-Oxybis(2,3,4,5-tetrabromobe	85446179	
2,2',3,3',4,5,5',6,6'-nonabromodip	437701785	
BDE-207	437701796	
BDE-201	446255501	
2,2',3,4,4',5,6,6'-Octabromodiphe	446255545	
Phosphoric trichloride, polymer w	125997219	
tert-Butylphenyl diphenyl phospha	56803373	
1,2,3,4,5-Pentabromo-6-(2,3,4,5-t	63387280	1
Triphenyl phosphates isopropylate	68937417	
Bis(2-ethylhexyl) phosphate	298077	
Hexabromocyclododecane	25637994	
Bis(1,3-dichloropropan-2-yl) hydro	72236727	
Benzene, 1,2,3,5-tetrabromo-4-(2,	117948637	
1,1'-Biphenyl, 4,4'-dibromo-	92864	
Diphenyl phosphate	838857	
6-Phenyl-1,3,5-triazine-2,4-diamin	91769	
2-Bromophenol	95567	
Pentaerythritol	115775	
Octyl diphenyl phosphate	115888	
Tris(2-chloroethyl) phosphite	140089	
6-Methyl-1,3,5-triazine-2,4-diamir	542029	
Phenol, 3-bromo-	591208	
1,3,5-Triazin-2(1H)-one, 4,6-diami	645921	
Triphenylphosphine oxide	791286	
Tetraethyl ethylenebisphosphonat	995324	
Dibutyl chlorendate	1770805	
s-Triazine, 2,2'-O-phenylene-bis(4,	5118796	
Dioctyl phenyl phosphate	6161815	
Isopropyl phenyl diphenyl phospha	28108998	
Phosphoric acid, dimethylphenyl c	29660682	
2-tert-Butylphenyl diphenyl phosp	83242233	
Bis(isopropylphenyl) phenyl phosp	28109004	

Nonabromodiphenyl ether	63936561
BDE-202	67797095
2,3,3',4,4',5,6-Heptabromodiphen	189084682
PBDE 195	446255385
BDE-198	446255421
PBDE 205	446255567
Tris(isopropylphenyl) phosphate	26967760
1,3,5-Triazine-2,4,6(1H,3H,5H)-tric	37640576
-	30554735
Benzene, 1,1'-[1,2-ethanediylbis(o	61262531
2,4,6-Tribromoanisole	607998
1,3,5-Tribromobenzene	626391
4,4'-Dibromodiphenyl ether	2050477
2,3',4,4',6-Pentabromodiphenyl Et	189084660
1,2,3-Tribromo-5-(3,4-dibromoph	366791324
1,2,3,4-Tetrabromo-5-(3,4-dibrom	405237856
2,2',5,5'-Tetrabromobiphenyl	59080374
1,1'-Biphenyl, 2,2',4,5,5'-pentabro	67888964
Diphenyl 4-tolyl phosphate	78319
o-Terphenyl	84151
1,1':3',1''-Terphenyl	92068
4-Bromobiphenyl	92660
1,1':4',1''-Terphenyl	92944
diethylphosphinic acid	813763
3-Bromobiphenyl	2113577
1,2,3,4,5,6,7,8-Octachloronaphtha	2234131
p-Tetradecachloroterphenyl	31710324
1,1':2',1''-Terphenyl, 2,2'',3,3',3'',4	42429889
1,1':3',1''-Terphenyl, 2,2',2'',3,3'',4	42429890
1,2,3,4,7,7-Hexachloro-5-(2,4,6-tri	56890892
2,3,5,6,2',3',5',6'-Octabromobiphe	59080410
1,1'-Biphenyl, 2,2',3,3',4,4',5,6'-oc	69278611
2,2',3,3',4,4',5,5',6-Nonabromo-1,	69278622
2,2',3,3',4,5,5',6'-Octabromobiphe	69887112
2,2',3,3',4,4'-Hexabromo-1,1'-biph	82865892
1,1'-Biphenyl, 2,2',3,3',4,4',6,6'-oc	119264594
2,2',3,3',4,5',6,6'-Octabromobiphe	119264607
2,2',3,4,4',5,6,6'-Octabromobipher	119264618
2,2',3,3',4,4',5,6,6'-Nonabromo-1,	119264629
2,2',3,3',4,5,5',6,6'-Nonobromobip	119264630
2',3,4-Tribromodiphenyl ether	147217785
BDE-199	446255432
BDE-200	446255465
Phosphoric trichloride, reaction pr	181028795
Tris(2,4-dibromophenyl) phosphat	2788116
Bromine--methane (1/16)	12079582
Ammonium bromide	12124979

4,4',6,6'-Tetrabromo-2,2'-bipheny	14957654
1,2-Benzenedicarboxylic acid, 3,4,	25357793
2,2-Bis(3,5-dibromo-4-hydroxyphe	28906130
Carbonic dichloride, polymer with	32844272
1,3,5-Tribromo-2-(2,3-dibromo-2-	36065302
3,5,3',5'-Tetrabromobisphenol A, (	40039938
Tris(dibromophenyl) phosphate	49690633
1,3,5-Triazine, 2,4,6-tris(2,3-dibroi	52434590
N,N'-(Ethylene)bis[4,5-dibromohe:	52907070
Poly(tribromostyrene)	57137107
Poly(pentabromobenzyl acrylate)	59447573
1H-Pyrrole-2,5-dione, 1-(2,4,6-trib	59789514
1,3-Butadiene, homopolymer, bro	68441463
2,2'-[(1-Methylethylidene)bis[(dib	68928701
1-Bromo-4-chlorodecane	68955419
2,4-(or 2,6)-Dibromophenol, homo	69882117
Phenol, 4,4'-(1-methylethylidene)I	71342773
Tetrabromophthalic acid mixed es	77098078
1~2~,1~3~,1~4~,1~5~,1~6~,2~3~,2	79596319
2,2',3,3',5,5',6,6'-Octabromo-4-ph	83929695
2-Bromoethyl 5-bromopentyl 2-ch	84282279
Benzene, ethenyl-, homopolymer,	88497567
Carbonic dichloride, polymer with	94334642
1,1'-(Isopropylidene)bis(3,5-dibroi	97416847
1-Propanol, 3,3'-oxybis[2,2-bis(brc	109678333
Phosphoric acid, mixed 3-bromo-2	125997208
Brominated epoxy resin end-capp	135229480
End capped and partially end capp	139638587
4,4'-(1-Methylethylidene)bis[2,6-c	158725441
Benzene, ethenyl-, polymer with 1	1195978938
Piperazine, phosphate (1:?)	1951979
Diphosphoric acid, compd. with 1,	15541603
Tris(dichloropropyl) phosphate	26604513
1,3,5-Triazine-2,4,6-triamine, phos	41583099
Diphosphoric acid, compd. with pi	66034171
C10-13 chloro alkanes	85535848
Cercelor S 52	85535859
Phosphoric acid, bis(methylphenyl	26446731
1,1'-{[(2Z)-2,3-dibromobut-2-ene-1	31977874
Tribromoneopentyl alcohol	36483575
Bromo chloro C12-30 a-alkenes	68527015
Alkenes, C12-24, chloro	68527026
Bis[(pentabromophenyl)methyl] 3	82001216
Bis[(pentabromophenyl)methyl] b	90075915
(rel)-(1R,2R,3S,4S)-1,2,3,9-tetrabr(	855992982
(rel)-(1R,2S,3S,4S)-1,2,3,9-tetrabr(	855993010
3-Bromostyrene	2039863

Tetrabromophthalic acid	13810838
2,3,4,6-Tetrabromophenol	14400943
2,3,4,5-Tetrabromobenzoic Acid	27581131
2,3,4,5-Tetrabromophenol	36313152
Tetraammonium octamolybdate	12411642
Dimethyl methylphosphonate	756796
Magnesium hydroxide	1309428
Antimony trioxide	1309644
Tin zinc hydroxide (ZnSn(OH)6)	12027962
Tin zinc oxide (SnZnO3)	12036372
Aluminum hydroxide	21645512
Ammonium polyphosphates	68333799
Melapur 200	218768844
Aluminum diethylphosphinate	225789388
Tris(3-methylbutyl) phosphate	919620
1,1'-Oxybis(2,3,4-tribromobenzen	182677287
Diethyl ethylphosphonate	78386
4-Bromostyrene	2039829
Etidronic acid	2809214
1,3,2-Dioxaphosphorinane, 2,2'-ox	4090511
Octabromobiphenyl	27858077
4,4'-(propane-2,2-diyl)bis(2-brom	29426786
2,2',4,4',5,5'-Hexabromobiphenyl	59080409
3,3',4,4',5,5'-Hexabromobiphenyl	60044260
3,5-Dibromo-2-(2,4-dibromophen	79755434
Tributylphosphine oxide	814299
Phosphonic acid, P-phenyl-, diocty	1754478
Ethanol, 2-bromo-, phosphate (3:1	27568907
Benzene, pentabromo(2,3-dibrom	32577347
Tris(2-bromo-4-methylphenyl) pho	35656010
1,3,5-Tribromo-2-methoxy-4-meth	41424366
2-Biphenylyl diphenyl phosphate	132296
Bis(2-ethylhexyl) phenyl phosphat	16368971
Benzene, bromophenoxy-	7025061
Benzene, 1,3-dibromo-2-phenoxy-	51930042
2,4,6-Tribromodiphenyl ether	155999954
2,4-Dichlorodiphenyl ether	51892263
BDE-180	446255261
1,2,4,5-Tetrabromobenzene	636282
p-Bromodiphenyl ether	101553
1-Bromo-2-(4-bromophenoxy)ben	147217718
1,2,3,5-Tetrabromo-4-(3,4,5-tribro	446255307

link	link	link	27741446	link	33969458	31603658	31276863
E	F	G	H	I	J	K	L
1	1	1	1	1			
1	1		1		1	1	
1	1	1	1	1		1	
1	1		1	1	1	1	
	1		1		1		
1	1		1			1	
1	1		1		1	1	
1	1		1		1	1	
1	1		1		1	1	
1	1	1	1	1			
1	1	1	1	1			
1	1	1	1	1		1	
1	1	1	1				
1		1	1	1		1	
			1				
1			1				
1	1	1		1			
1	1	1		1			
1	1				1		
1	1				1	1	
1	1			1			
1	1					1	
1	1	1		1		1	
1	1						
1	1			1			
1	1	1			1	1	
1	1			1			
1	1	1				1	
1	1			1			
1	1	1				1	
1	1					1	
1	1						
1	1			1		1	
1	1			1			
1	1						
1	1						
1	1						
1	1						
1	1					1	
1	1						
1	1						

1	1						
1	1						
1	1	1					
1	1						
1	1						
1	1						
1	1						
1	1						
1	1			1			
1	1					1	
1	1					1	
1	1						
1	1						
							1
							1
1				1			
1		1				1	1
						1	
					1	1	
1						1	
1						1	
1						1	
1							
1						1	
1						1	
						1	
						1	1
							1
							1
							1
						1	
							1
1							
				1			
1					1	1	
1				1			

1			
1			
1			
1			
1			
1			
1			
1			
1			
	1		
1			
1		1	
1			
1			
1			
1			1
1			1
1			
1			1
1			1
1			1

			1
1			
1			
1			
		1	
		1	
1		1	
		1	
		1	
	1		
		1	
	1		
1		1	

1		
		1
		1
		1
		1
	1	
	1	
	1	
1		1
1		1
1		1
1		1

		1
		1
	1	
1		
1		
1		

27934115	32100996	27623734	28869101	30196219	27309668	27144674
**M**	**N**	**O**	**P**	**Q**	**R**	**S**
1		1				
	1	1		1	1	1
1						
1		1	1			
					1	1
	1	1		1	1	1
1	1	1		1	1	1
	1	1		1	1	1
1						
1						
			1			
1		1				
1		1				
1		1				
1	1			1	1	1
	1			1	1	1
1						
	1				1	1
1						
	1			1	1	1
1						
			1			
1						
1			1			
1						
			1			
	1					
1						
1						
1						
1						
				1	1	1
			1			
					1	1

	1				1	1
					1	1
			1			
1		1				
1		1				
1						
1						
					1	1
			1			
1			1			
			1			
1						
	1				1	1
1						
				1	1	1
	1			1	1	1

	1			1	1	1
					1	1
	1			1	1	1
1						
					1	1
					1	1
					1	1
					1	1
					1	1
					1	1
					1	1
		1	1			
		1	1			
					1	1
					1	1
		1	1			
1						

		1	1
1			
1			
		1	1
		1	1
	1		
		1	1
1			

	1
	1
1	
1	
1	

33026620	link	31081616	28556503	26099790	23724807	24493336	28034506	26742016
T	U	V	W	X	Y	Z	aa	bb
1	1	1				1	1	1
	1	1	1	1	1	1		1
1	1						1	1
1	1					1	1	1
	1						1	1
1	1	1					1	1
	1		1	1	1	1	1	1
	1		1		1	1	1	1
			1	1	1			1
1	1						1	1
1	1						1	1
1							1	1
							1	
1	1	1				1		1
1	1	1				1	1	1
	1	1				1	1	1
	1		1	1	1	1		1
	1			1		1	1	1
1	1					1	1	1
	1		1	1	1	1	1	1
1	1						1	1
	1		1	1	1	1		1
1	1					1	1	1
1								1
	1					1	1	
1							1	1
	1						1	1
1							1	1
1							1	
	1		1	1	1		1	1
							1	1
	1						1	1
1	1						1	1
	1						1	1
	1						1	
	1						1	
								1
	1	1						1
	1						1	1

	1							
		1						1
								1
							1	1
								1
							1	1
								1
	1							
							1	
							1	
	1					1	1	1
1		1						1
	1		1	1		1		1
1								1
1								1
1								
1								
								1
1								
								1
								1
								1
1	1							1
1	1							1
								1
	1		1	1	1			1
1	1							1
	1			1				1
				1		1		
	1	1						1

			1	1	1			
	1						1	1
				1				
							1	1
				1				1
	1				1			1
		1						1
								1
		1						1
1								1
								1
		1						1
	1							1
						1		1
								1
								1
		1						1
								1
								1
						1		
						1		
	1							
						1		
								1
1								
	1							
	1							
								1
								1
								1
								1
								1
		1						
								1
								1
								1
								1
								1
								1
1								
1								

1	1	
		1
		1
		1
		1
		1
		1
		1
		1
		1
		1
		1
	1	

1

1

1

1

							1
							1
							1
							1
							1
							1
							1
							1
							1
			1				
			1				
	1						
1							
1							
1							

24846325	26363216	link	26134984	24656972	32417508	26613357	link	28338271
cc	dd	ee	ff	gg	hh	ii	jj	kk
1		1	1	1	1	1	1	1
1	1					1	1	1
1		1	1	1	1	1	1	1
			1	1		1	1	1
	1					1	1	1
1		1				1	1	
1	1	1				1	1	1
1	1	1	1			1	1	1
1	1	1				1	1	1
1		1	1	1	1	1	1	1
1		1	1	1	1	1	1	1
		1		1	1	1	1	1
1		1	1	1	1	1	1	1
					1	1	1	
1		1	1		1	1	1	1
		1	1	1	1	1	1	1
1	1		1			1	1	1
1	1					1	1	1
1		1	1	1	1	1	1	1
1	1					1	1	1
1		1	1	1	1	1	1	1
	1					1	1	1
1			1	1	1	1	1	1
1								1
1			1		1		1	1
1								1
1			1	1	1	1	1	
1								
1								1
							1	1
		1	1			1		1
1			1	1	1	1	1	
1			1	1	1	1	1	1
			1	1	1	1		1
1			1		1	1	1	1
						1	1	
		1		1	1	1	1	1
					1	1	1	1
				1	1	1	1	

						1	1	1
	1						1	1
				1	1	1	1	
				1	1	1	1	
				1	1	1	1	
					1	1		
					1	1	1	
							1	
					1		1	
					1		1	
1								
							1	
			1				1	1
			1					1
1		1	1	1	1	1	1	1
1		1						1
	1						1	
1								
1								
1								1
1								
				1	1	1	1	1
		1						
			1			1		
								1
								1
1		1				1	1	
1		1				1	1	
		1				1		
		1						
							1	
	1					1	1	1
1			1		1	1	1	1
	1			1	1	1	1	1
1						1	1	
		1		1	1	1	1	1

1						1		
	1	1				1	1	1
1						1	1	
			1	1	1	1	1	1
	1						1	1
	1					1	1	
	1						1	1
		1		1	1	1	1	1
				1	1	1	1	1
				1	1	1	1	1
				1	1	1	1	1
	1					1	1	1
				1	1	1	1	1
		1		1	1	1	1	
	1							1
				1	1	1	1	1
				1	1	1	1	1
					1	1	1	1
	1						1	1
						1		
			1					
						1		1
						1	1	
					1	1	1	
								1
							1	
							1	
1			1			1		
					1		1	
					1	1		1
				1	1	1		
				1	1	1		
					1	1		
1			1			1		
							1	1
						1	1	
					1	1		
	1							1
					1	1		
					1	1		
					1	1		
					1	1		

	1		1
	1	1	
	1		
	1		
	1		
		1	
		1	
		1	
		1	
		1	
		1	1
1			
			1
	1		1
			1
	1		1
	1		
	1		
	1		
			1
		1	
		1	
		1	
		1	
		1	
		1	
		1	
		1	
		1	
		1	
		1	
		1	
		1	
		1	
		1	
		1	

					1	1
1					1	
			1			
			1			
						1
						1
						1

			1
			1
			1
			1
			1
			1
			1
			1
			1
			1
			1
			1
			1
			1
			1
			1
			1
			1
			1
			1
			1
			1
			1
			1
			1
			1
			1
			1
			1
			1
		1	
		1	
		1	
		1	
		1	
		1	
		1	
	1		
	1		
	1		
	1		
	1		
	1		
	1		
	1		
	1		
1			

					1
					1
					1
					1
		1			
	1				
	1				
	1				
	1				
	1				
	1				
	1				
	1				
	1				
1					
1					

29734094	25933174	25929991	29320633	link	32854002	25493574	33736204	28910084
ll	mm	nn	oo	pp	qq	rr	ss	tt
1	1		1	1		1		1
1	1	1		1	1			1
1	1		1	1		1	1	1
	1			1				
	1	1			1			
1				1				
1	1	1		1	1			1
1	1	1		1	1			1
1	1	1		1	1			1
1	1		1	1		1	1	1
1	1		1	1		1	1	1
	1			1				
1	1		1	1		1		1
	1			1				
1	1		1	1		1		1
1	1		1	1		1		1
1	1	1		1				1
1		1		1	1			1
1	1		1	1				1
1	1	1		1				1
1	1			1		1		1
1	1	1		1	1			1
1	1		1	1		1		1
1	1		1	1			1	1
1			1	1		1		1
1	1		1	1			1	1
1	1			1				1
1	1		1	1			1	1
1	1		1	1			1	1
	1	1		1				1
	1		1	1				
1	1		1	1		1	1	1
1	1		1	1				1
1				1		1	1	1
				1			1	
1				1	1			1
	1			1				
	1			1				
	1		1	1				

1				1				
	1			1				
	1			1				
	1			1				
	1			1				
	1			1				
	1			1				
		1		1				1
				1				
				1				
				1				
				1				
			1					1
			1					1
1	1		1	1		1		1
1	1		1	1			1	1
1	1	1		1				1
1	1		1	1			1	1
1	1		1	1			1	1
1	1		1	1			1	1
1			1	1				1
	1			1				
1			1	1				1
				1				
			1					
						1		
			1					
			1					
				1				
				1				
				1				
			1					
	1	1		1				
1			1	1		1	1	1
1	1			1				
1				1				1
	1			1				

1				1				1
	1			1	1			
1				1				1
	1			1				1
1	1				1			
	1			1				
	1			1				
	1			1				
	1			1				
	1			1				
	1			1				
				1				
	1			1				
	1			1				
	1			1				
	1			1				
				1				
				1				
	1			1				
	1			1				
							1	1
							1	1
1								1
		1		1				
							1	1
	1			1				
			1	1				
1				1				1
1				1				1
							1	
				1				
				1				
				1				
				1				
	1			1				
							1	
				1				
				1				
				1				
				1				
				1				
				1				
				1				
				1				
			1					
			1					
			1					

			1			
			1			
			1			
			1			
			1			
			1			
			1			
1			1			
	1		1			
			1			
			1			
			1			
			1			
		1				
		1				
		1				
		1				
		1				
1						

			1			1
				1		
			1			
			1			
			1			
			1			
			1			
			1			
			1			
			1			
			1			
			1			
			1			
			1			
			1			
			1			
			1			
			1			
			1			

	1		
	1		
	1		
	1		
	1		
	1		
			1
			1
		1	
		1	
		1	
		1	
		1	
		1	
		1	
		1	
		1	
		1	
		1	
		1	
		1	
		1	
		1	
		1	
		1	
		1	
		1	
		1	
		1	
		1	
	1		
	1		
	1		
1			

26725304	32460157	30448504	25240235	none	link	
uu	vv	ww	xx	yy	zz	SUM
1			1		1	33
	1	1				32
			1		1	30
1						24
					1	18
						16
	1	1			1	33
	1	1				32
	1	1			1	30
			1			27
			1			27
			1			19
						5
1			1			29
						6
						2
1					1	30
1			1		1	29
	1	1				29
	1				1	27
			1		1	27
	1	1				27
					1	26
	1	1				26
						25
1					1	21
					1	21
1					1	21
					1	20
1						19
1					1	18
					1	17
						14
						21
						19
						17
					1	14
						13
						13
						13
						12

				11
				11
				10
				10
				9
				8
				8
				7
				7
				7
				6
				4
				2
			1	12
			1	11
			1	24
1			1	21
	1			18
1			1	17
1				16
1				15
1				12
				10
				9
		1		5
				5
				4
				4
				3
				3
				2
				2
				2
				2
				1
				1
				11
				10
				5
				4
				4
				2
	1			17
				17
				16
				15
		1		14

	1			14
				14
		1		13
				13
	1			12
				11
				10
				10
				10
				10
				9
				9
				9
				9
				9
				8
				8
				8
				8
			1	7
1			1	7
1				7
				7
				7
1				6
				6
				6
				5
				5
				5
				5
				5
				5
				5
				5
				4
				4
				4
				4
				4
				4
				4
				4
				4
				4
				4
				4

4
4
3
3
3
3
3
3
3
3
3
3
3
3
3
3
3
3
3
3
3
3
3
3
3
3
3
3
2
2
2
2
2
2
2
2
2
2
2
2
2
2
2
2
2
2
2

2
2
2
2
2
2
2
2
2
2
2
2
2
2
2
2
1
1
1
1
1
1
1
1
1
1
1
1
1
1
1
1
1
1
1
1
1
1
1
1
1
1
1
1
1
1
1

1
1
1
1
1
1
1
1
1
1
1
1
1
1
1
1
1
1
1
1
1
1
1
1
1
1
1
1
1
1
1
1
1
1
1
1
1
1
1
1
1
1
1
1
1
1
1

1
1
1
1
1
1
1
1
1
1
1
1
1
1
1
1
1
1
1
1
1
1
1
1
1
1
1
1
1
1
1
1
1
1
1
1
1
1
1
1
1
1

Sample Queries

Category	Heading
Ecological	Algae
Epidemiology	Epi
Exposure	Dust exposure
Exposure	Food exposure
Exposure	Water exposure
Exposure	Occupational
Emergency/disaster	Emergency / disaster
Decontamination	Decontamination
Mechanism	Oxidative stress
Metabolism	Metabolism
Methods	Analytical chemistry
Methods	Statistical
Methods	In vitro
Mixtures	Mixtures
Medicine	Clinical trials
Medicine	Clinical trials in children
Medicine	Obesity
Toxicity	Genetox
Toxicity	Cancer
Toxicity	ReproTox
Toxicity	NeuroTox
Toxicity	DevTox
Toxicity	Skin sensitization
Toxicity	DNT
Use	Pharmaceutical
Use	Pesticide
Use	Cosmetics
Use	Explosive Agents
Use	Food
Use	Surface-acting
Use	Dye/coloring
Use	Fertilizer
Use	Solvents
Use	Flame retardant
Repro	Female repro
Repro	Male repro
Zebrafish	Zfish / neurotox
Zebrafish	Zfish / dev / behavior
Tests	Resp Sens Tests
Tests	Skin Sens Tests
Toxicity	Respiratory sensitisation
EmbDev	EmbDev

HeartDev	Heart Dev
HeartDevDefects	Heart Defect
HeartFlow	Heart Flow Dynamics
HeartDev	Heart Dev Dynamics
HeartCells	Heart Cells
HeartDev	Heart Dev Stages
Heart	System
HeartCellDefect	Heart Cell Defects
DevTransport	DevTransport
Justice	EnvJustice

Note: these are starting points … please expand and customize

Query (double-click to see how the query looks to PubMed)

algal bloom OR eutrophication OR algae OR Harmful algal bloom OR red tide
epidemiology
environmental exposure AND dust
environmental exposure AND food
environmental exposure AND (water OR groundwater OR drinking water)
(occupational exposure OR Air Pollutants, Occupational OR ((worker OR workers OR employee OR workplace OR i
(emergency OR emergencies OR spill OR spills OR disaster OR disasters or hurricane OR hurricanes OR flood OR
(decontamination OR Environmental Restoration and Remediation)
"oxidative stress" OR "free radicals" OR "reactive oxygen species" OR peroxides
(metabolism OR metabolite OR tissue distribution OR pharmacokinetics OR pharmacodynamics)
"Chemistry Techniques, Analytical" OR analytical chemistry
Statistics as Topic[mh] OR statistics OR statistical
In Vitro Techniques[mh] OR cell culture or "in vitro"
(Drug synergism[mh] OR cocarcinogenesis OR pesticide synergists[mh] OR mixture[tiab] OR mixtures[tiab] OR Dr
((clinical[Title/Abstract] AND trial[Title/Abstract]) OR clinical trial[Publication Type])
((children OR child OR infants) AND human) AND ((clinical[Title/Abstract] AND trial[Title/Abstract]) OR clinical trial
(obesity OR obese OR adipose OR overweight Or adipogenesis OR adipose tissue)
(dna/drug effects OR DNA Damage OR chromosome aberrations OR genotoxicity OR micronucleus OR DNA Repa
neoplasms or cancer OR carcinogen* OR precancerous
(reproduction AND (toxicity OR abnormal OR adverse effects))
(neurotoxicity OR (Nervous system diseases and chemically induced) OR ((neurons OR brain OR behavior) AND dru
((toxicity OR congenital abnormalities OR Prenatal Exposure Delayed Effects) AND (fetus OR embryo OR embryoni
("allergic" AND "contact" And dermatitis) OR Dermatitis, Allergic Contact[mh]
((Brain OR central nervous system OR "CNS" OR "neural tube" OR spinal cord OR spina bifida OR Nervous System I
"therapeutic use" OR "therapeutic use"[subheading]" OR pharmacologic actions[mh] OR drug therapy
pesticide OR insecticide OR rodenticide OR fungicide
cosmetics OR beauty
Explosive Agents OR explosive OR explosives
food OR diet OR beverage OR nutrition OR eating
Antifoaming OR Anti-foaming OR detergent OR detergents OR soap OR detergent OR surfactant
dye OR "coloring agent" OR pigment OR pigments
fertilizer OR fertilize
solvents OR solvent
flame retardant OR flame retardants OR fire suppression
(“female genitalia” OR “female gonad” OR “Female reproduction” OR “Granulosa cells” OR “Polycystic ovarian syn
(Aspermia OR Asthenozoospermia OR Azoospermia OR Blood-Testis Barrier OR Cryptorchidism OR Ejaculation OR
zebrafish AND neurotox*
zebrafish and (behavior or locomotor) and develop*
(nasal challenge OR bronchial challenge OR bronchial provocation test OR forced expiratory volume OR vital capac
(skin prick test OR Local Lymph Node Assay OR "LLNA"[tiab] OR skin tests OR Skin Irritancy Tests OR DPRA[tiab]OI
(Respiratory hypersensitivity OR respiratory sensitization OR Bronchial Hyperreactivity OR Respiration Disorders C
(embryo OR embryonic Or embryonal OR embryogenesis OR embryology OR fetus Or fetal OR larvae OR larval OI

(heart OR cardiac OR cardiogenesis OR cardiopoiesis OR atrium OR atrial OR ventricle OR chamber OR aortic OR a
((Heart defects, congenital[mh] OR congenital heart defects) OR   (heart AND (abnormalities OR pathologies OR pa
(Hemodynamics OR bidirectional blood flow OR unidirectional blood flow OR Mechanobiology OR Dorsal mesencl
(embryo OR embryonic Or embryonal OR embryogenesis OR embryology OR fetus Or fetal OR larvae OR larval OR
(((cardiac OR heart) AND (progenitor OR stem cell OR neural crest OR jelly OR fibroblasts OR endothelial OR myoc
((cardiac OR heart) AND (crescent OR tube OR loop OR looping OR maturation OR field)) OR (endocardial OR endo
(heart OR cardiac OR cardiogenesis OR cardiopoiesis OR atrium OR atrial OR ventricle OR chamber OR aortic OR a
((cardiac AND (progenitor OR stem cell OR neural crest OR jelly)) OR cardioprogenitors OR cardiomyocytes OR spi
(transport OR uptake OR storage OR metabolism) AND (pregnancy OR placenta OR placental)
(Environmental Justice OR (Social justice[tw] And environment) OR climate justice[tw] OR Urban justice[tw] OR (B

Comments
any relationship

industry) AND exposure))

needs work

ug Antagonism[mh] OR Drug Interactions[mh] OR drug agonism)

[Publication Type])

ir OR mutagenicity tests OR mutagens OR mutagenicity OR mutagenic or "gene mutation" OR "ames " OR "com

ug effects)
c development OR larva OR eggs)) or embryotoxicity

From DNT Robinson

From Nordic Working paper
From Nordic Working paper

city OR Bronchial Provocation Tests OR Peak Expiratory Flow Rate OR Respiratory Function Tests OR Diagnostic
R "KeratinoSens"[tiab] OR "LuSens"[tiab] OR "h-CLAT"[tiab])
R Respiratory Tract Diseases OR (Drug hypersensitivity AND lung) OR (irritants AND lung) OR (occupational dise
R chick OR Embryonic and Fetal Development[tw])

orta OR outflow OR pulmonary OR septum OR septal OR foramen ovale OR cushion OR sinus venosus OR trunc
athology OR teratogenesis)) OR (septum primus OR septum secundum OR septal defect OR septal defects OR (s
hymal protrusion OR Conductive system OR (heart AND chamber))
chick OR Embryonic and Fetal Development[tw]) AND (Hemodynamics OR bidirectional blood flow OR unidire
cytes)) OR cardioprogenitors OR cardiomyocytes OR spinal neural crest OR epicardial progenitor)
cardium OR myocardium OR myocardial OR epicardium OR epicardial OR pericardium OR pericardial OR Proepi
orta OR outflow OR pulmonary OR septum OR septal OR foramen ovale OR cushion OR sinus venosus OR trunc
nal neural crest OR epicardial progenitor) AND (hypoplasia OR hyperplasia OR proliferation Or hypertrophy OR

uilt environment AND (equity OR equality OR justice)) OR (Health Status Disparities AND environment))

Techniques, Respiratory System[tw] OR Respiratory Function Tests OR Pulmonary Ventilation OR Forced Expira

eases AND lung) OR ((asthma OR rhinitis) AND chemically induced) OR Respiration/drug effects OR Bronchocon

:us arteriosus OR patterning) AND (embryo OR embryonic Or embryonal OR embryogenesis OR embryology OF
;eptum AND defect*) OR (myocardial AND bridging) OR Myocardial Bridging[tw] OR Tetralogy of Fallot OR trar

ctional blood flow OR Mechanobiology OR Dorsal mesenchymal protrusion OR Conductive system OR (heart Al

atory Flow Rates OR Forced Expiratory Volume OR Maximal Voluntary Ventilation OR "forced vital capacity"[tia

nsposition of great vessels OR overriding aorta OR ventricular hypoplasia OR Interventricular septal defects O

R valvular septal defects OR Pericardial edema))

Read Me

This macro-enabled Excel file was created by running extracts fr[illegible]
LitDB database of MeSH terms extracted from PubMed. Articles [illegible]
Retardants annotated were selected, then for each of those rec[illegible]
names annotated as major topics were extracted. The resulting [illegible]
inserted into this tool and navigation steps were programmed. [illegible]
MeSH chemical names to DSSToxIDs were made. Notes that this [illegible]
always a one-to-one correspondence. The Preferred Name is the [illegible]
associated with the DSSToxID in the EPA Chemicals Dashboard.

To use, be sure to enable macros and content. Start with the Ov[illegible]
Browse and sort. The chemical names are hyperlinked to the M[illegible]
Double-click on an article count to see the article titles on the De[illegible]
PMIDs are hyperlinked to PubMed.

om the EPA
with Flame
ords, chemical
data was
Links from
s mapping is not
e name

verview sheet.
eSH Browser.
etail sheet.

Overview

Double-click on PMID Ct to see detail

DSSToxID	EPA Preferred Name
DTXSID4027527	1,2,5,6,9,10-Hexabromocyclododecane
DTXSID8025383	Hexabromocyclododecane
DTXSID1026081	3,3',5,5'-Tetrabromobisphenol A
DTXSID9020376	1,1'-Oxybis[2,3,4,5,6-pentabromobenzene]
DTXSID2024246	Pentabromodiphenyl ether
DTXSID4052685	2,2',3,4,4'-Pentabromodiphenyl ether
DTXSID4052689	2,2',4,4',6-Pentabromodiphenyl ether
DTXSID9030048	2,2',4,4',5-Pentabromodiphenyl ether
DTXSID3030056	2,2',4,4'-Tetrabromodiphenyl ether
DTXSID5024267	Polychlorinated biphenyls
DTXSID7027750	Dechlorane Plus
DTXSID5021413	Tris(2,3-dibromopropyl) phosphate
DTXSID1035238	Bromine
DTXSID1021952	Triphenyl phosphate
DTXSID9026261	Tris(1,3-dichloro-2-propyl) phosphate
DTXSID5021411	Tris(2-chloroethyl) phosphate
DTXSID8024947	1,2-Dibromo-4-(1,2-dibromoethyl)cyclohexane
DTXSID7020182	Bisphenol A
DTXSID5021758	Tris(2-butoxyethyl) phosphate
DTXSID1024627	1,2-Bis(2,4,6-tribromophenoxy)ethane
DTXSID3021986	Tributyl phosphate
DTXSID1024128	Hexabromobenzene
DTXSID3021770	2,2',6,6'-Tetrachlorobisphenol A
DTXSID2021315	2,3,7,8-Tetrachlorodibenzo-p-dioxin
DTXSID801017084	polychlorodibenzo-4-dioxin
DTXSID1024382	Phosphorus
DTXSID6021959	2,4,6-Tribromophenol
DTXSID8052693	2,2',3,4,4',5',6-Heptabromodiphenyl ether
DTXSID9021847	Diphenyl oxide
DTXSID9052686	2-Ethylhexyl 2,3,4,5-tetrabromobenzoate
DTXSID9052713	1,3,5-Tris(2,3-dibromopropyl)-1,3,5-triazine-2,4,6(1H
DTXSID5021174	Polybrominated biphenyls (PBB)
DTXSID70858838	2,2',4,4',5,5'-Hexabromobiphenyl
DTXSID9020164	Pentaerythritol dibromide
DTXSID201016652	Tris(chloropropyl)phosphate
DTXSID3027615	Tris(2-chloropropyl) phosphate
DTXSID8026228	Triethyl phosphate
DTXSID6020802	Melamine
DTXSID9068328	1,3,5-Triazine-2,4,6-triamine, phosphate
DTXSID2049634	Graphite
DTXSID40893054	Tribromoneopentyl alcohol
DTXSID5026415	Bromotrifluoromethane
DTXSID6030782	Bentonite

DTXSID7020637	Formaldehyde
DTXSID7021817	2,3-Dibromopropanol
DTXSID8020410	Dibenzo-p-dioxin
DTXSID90873927	2,2',4,5'-Tetrabromodiphenyl Ether
DTXSID0052750	1,2,5,6-Tetrabromocyclooctane
DTXSID10865889	Tetrabromobisphenol A dimethyl ether
DTXSID5020023	Acrolein
DTXSID70880073	Firemaster 550
DTXSID0049814	Vinyl acetate ethylene copolymer
DTXSID0052700	2,2'-[(1-Methylethylidene)bis[(2,6-dibromo-4,1-phe
DTXSID00955980	Bis(2,3-dibromopropyl) hydrogen phosphate--ammc
DTXSID1025300	2-Ethylhexyl diphenyl phosphate
DTXSID1029677	Silica
DTXSID1052290	2,4-Dibromophenol
DTXSID10873929	2,2',4,4',6,6'-Hexabromodiphenyl Ether
DTXSID2032180	2,2',4,4',5,5'-Hexachlorobiphenyl
DTXSID3020174	Bis(2,3-dibromopropyl) phosphate, magnesium salt
DTXSID3025833	Pentabromotoluene
DTXSID40889331	myo-Inositol hexakisphosphate
DTXSID5026259	Tris(2-chloroisopropyl)phosphate
DTXSID50931491	2-Bromopropenal
DTXSID7021782	2,3,4,5,6-Pentabromoethylbenzene
DTXSID7023805	Tris(2,3-dichloropropyl)phosphate
DTXSID70275889	Bis(2,3-dibromopropyl) hydrogen phosphate
DTXSID70983452	Sodium bis(2,3-dibromopropyl) phosphate
DTXSID8043781	Tetrabromophthalic acid
DTXSID8052641	2-Bromophenol
DTXSID901017087	pentabromocyclododecene
DTXSID9022079	Pentabromophenol
DTXSID90889689	1,2-Benzenedicarboxylic acid, 3,4,5,6-tetrabromo-, s
DTXSID001016751	Octabromobiphenyl
DTXSID0021173	Firemaster FF-1
DTXSID0021331	Tetrakis(hydroxymethyl)phosphonium sulfate
DTXSID0027147	Methane, bromochlorodifluoro-
DTXSID0027195	Hexadecane
DTXSID0060561	Phenol, 2,6-dibromo-
DTXSID00894087	Bromkal 70
DTXSID10214757	Ammelide
DTXSID1064746	Phosphonium, tetrakis(hydroxymethyl)-, acetate (sa
DTXSID1068720	Phosphonium, tetrakis(hydroxymethyl)-, ethanedioa
DTXSID10987252	2,3,4,5,2',3',4',5'-Octabromobiphenyl
DTXSID2026943	Pentaerythritol
DTXSID2062325	Trifluoroiodomethane
DTXSID20879972	BDE-175
DTXSID30451985	BDE-207
DTXSID3052692	2,2',4,4',5,6'-Hexabromodiphenyl ether
DTXSID3060950	1,3,5-Triazin-2(1H)-one, 4,6-diamino-

DTXSID4023880	Antimony trioxide
DTXSID4023888	Asbestos
DTXSID40556652	2,2',3,3',4,5,5',6,6'-nonabromodiphenyl ether
DTXSID5021330	Tetramethylolphosphonium chloride
DTXSID6029705	Diammonium hydrogen phosphate
DTXSID6032192	Tri-o-cresyl phosphate
DTXSID6049694	Policapram (Nylon 6)
DTXSID6050122	Phosphoric acid, ammonium salt (1:x)
DTXSID7024873	Cyanuric acid
DTXSID7035016	Zinc oxide
DTXSID7060910	1,2,4,5-Tetrabromobenzene
DTXSID8031865	Perfluorooctanoic acid
DTXSID9066752	Phosphonium, tetrakis(hydroxymethyl)-, phosphate
DTXSID1020273	Chlorine
DTXSID1021403	Trimethyl phosphate
DTXSID10218114	2,4,5,3',4'-Pentabromobiphenyl
DTXSID10228273	2,3,3',4,4',5-Hexabromobiphenyl
DTXSID1044699	Diethyl hydrogen phosphate
DTXSID1061073	Heptafluoropropyl iodide
DTXSID201009900	2,2',5,5'-Tetrabromobiphenyl
DTXSID2020268	Chlorendic acid
DTXSID2020686	Lindane
DTXSID2020688	Hexachlorocyclopentadiene
DTXSID2027545	Chlordene
DTXSID2029242	Diethyl (N,N-bis(2-hydroxyethyl)amino)methanepho
DTXSID2036405	Aluminum hydroxide
DTXSID3020205	Benzyl butyl phthalate
DTXSID3020679	Heptachlor
DTXSID3051543	4-Bromophenol
DTXSID3068043	1,3,5-Triazine-2,4,6(1H,3H,5H)-trione, compd. with [illegible]
DTXSID30958080	Ethenyl dihydrogen phosphate
DTXSID40160258	Zirconium phosphate
DTXSID4042381	Silicic acid
DTXSID4049662	Magnesium hydroxide
DTXSID4074932	Perfluoro-2-methyl-3-pentanone
DTXSID40872703	2,2',4-Tribromodiphenyl ether
DTXSID501017085	2,3-Dibromopropylphosphate
DTXSID5020100	Aroclor 1254
DTXSID5023875	Ammonium sulfamate
DTXSID5026918	1-Dodecanol
DTXSID50873428	Dibutyl phenyl phosphate
DTXSID50911718	N-(Hydroxymethyl)-3-[methoxy(methyl)phosphoryl]
DTXSID6020436	Dichlorodifluoromethane
DTXSID60229856	3,5-Dibromo-2-(2,4-dibromophenoxy)phenol
DTXSID6025010	1,3-Dichloro-2-propanol
DTXSID6026084	4,5,6,7-Tetrabromo-1,3-Isobenzofurandione
DTXSID6073524	2,3,7,8-Tetrabromodibenzo-p-dioxin

DTXSID6074754	1,1'-Biphenyl, 2,3,3',4,4',5,6-heptabromo-
DTXSID60894649	Cellulose, acetate butanoate
DTXSID7020267	Chlordane
DTXSID7020970	Nitrofen
DTXSID7025180	4,4'-Diphenylmethane diisocyanate
DTXSID7047724	Polytetrafluoroethylene
DTXSID8021432	Vinyl bromide
DTXSID8029656	Sulfur fluoride (SF6)
DTXSID901017118	Torflam
DTXSID9020827	Methoxychlor
DTXSID90879929	BDE-121

MeSH Chemical Name (hyperlinked)	PMID Ct
hexabromocyclododecane	283
hexabromocyclododecane	283
tetrabromobisphenol A	247
decabromobiphenyl ether	229
pentabromodiphenyl ether	154
pentabromodiphenyl ether	154
pentabromodiphenyl ether	154
pentabromodiphenyl ether	154
2,2',4,4'-tetrabromodiphenyl ether	127
Polychlorinated Biphenyls	103
dechlorane plus	94
tris(2,3-dibromopropyl)phosphate	60
Bromine	54
triphenyl phosphate	46
tris(1,3-dichloro-2-propyl)phosphate	41
tris(chloroethyl)phosphate	31
1,2-dibromo-4-(1,2-dibromoethyl)cyclohexane	21
bisphenol A	18
tris(2-butoxyethyl) phosphate	17
1,2-bis(2,4,6-tribromophenoxy)ethane	15
tributyl phosphate	14
hexabromobenzene	13
tetrachlorodian	13
Polychlorinated Dibenzodioxins	12
Polychlorinated Dibenzodioxins	12
Phosphorus	11
2,4,6-tribromophenol	11
2,2',3,4,4',5',6-heptabromodiphenyl ether	11
phenyl ether	11
2-ethylhexyl 2,3,4,5-tetrabromobenzoate	10
tris(2,3-dibromopropyl)isocyanurate	10
Firemaster BP-6	9
2,4,5,2',4',5'-hexabromobiphenyl	9
2,2-bis(bromomethyl)-1,3-propanediol	9
Fyrol PCF	8
Fyrol PCF	8
triethyl phosphate	8
melamine	7
melamine	7
Graphite	5
tribromoneopentyl alcohol	5
bromotrifluoromethane	5
Bentonite	5

Formaldehyde	5
2,3-dibromopropanol	5
dibenzo(1,4)dioxin	5
2,2',4,5'-tetrabromodiphenyl ether	5
1,2,5,6-tetrabromocyclooctane	4
tetrabromobisphenol A dimethyl ether	4
Acrolein	4
Firemaster 550	4
ethylenevinylacetate copolymer	3
tetrabromobisphenol A diglycidyl ether	3
bis(2,3-dibromopropyl)phosphate	3
2-ethylhexyldiphenylphosphate	3
Silicon Dioxide	3
2,4-dibromophenol	3
hexabromodiphenyl ether 154	3
2,4,5,2',4',5'-hexachlorobiphenyl	3
bis(2,3-dibromopropyl)phosphate	3
2,3,4,5,6-pentabromotoluene	3
Phytic Acid	3
tri-(2-chloroisopropyl)phosphate	3
2-bromoacrolein	3
2,3,4,5,6-pentabromoethylbenzene	3
tris(2,3-dichloropropyl)phosphate	3
bis(2,3-dibromopropyl)phosphate	3
bis(2,3-dibromopropyl)phosphate	3
tetrabromophthalic acid	3
2-bromophenol	3
pentabromocyclododecene	3
pentabromophenol	3
tetrabromophthalic acid	3
octabromobiphenyl	2
Firemaster FF-1	2
tetramethylolphosphonium chloride	2
bromochlorodifluoromethane	2
n-hexadecane	2
2,6-dibromophenol	2
Bromkal 70	2
ammelide	2
tetramethylolphosphonium chloride	2
tetramethylolphosphonium chloride	2
octabromobiphenyl	2
pentaerythritol	2
trifluoroiodomethane	2
2,2',4,4',5,6'-hexabromodiphenyl ether	2
2,2',3,3',4,4',5,6,6'-nonabromodiphenyl ether	2
2,2',4,4',5,6'-hexabromodiphenyl ether	2
ammeline	2

antimony trioxide	2
Asbestos	2
2,2',3,3',4,5,5',6,6'-nonabromodiphenyl ether	2
tetramethylolphosphonium chloride	2
ammonium phosphate	2
tri-o-cresyl phosphate	2
nylon 6	2
ammonium phosphate	2
cyanuric acid	2
Zinc Oxide	2
1,2,4,5-tetrabromobenzene	2
perfluorooctanoic acid	2
tetramethylolphosphonium chloride	2
Chlorine	1
trimethyl phosphate	1
2,4,5,3',4'-pentabromobiphenyl	1
2,3,3',4,4',5-hexabromobiphenyl	1
diethyl phosphate	1
1,1,2,2,3,3,3-heptafluoro-1-iodopropane	1
2,2',5,5'-tetrabromobiphenyl	1
chlorendic acid	1
Hexachlorocyclohexane	1
hexachlorocyclopentadiene	1
chlordene	1
Fyrol 6	1
Aluminum Hydroxide	1
butylbenzyl phthalate	1
Heptachlor	1
4-bromophenol	1
melamine cyanurate	1
vinyl phosphate	1
zirconium phosphate	1
Silicic Acid	1
Magnesium Hydroxide	1
perfluoro-2-methyl-3-pentanone	1
tribromodiphenyl ether 28	1
2,3-dibromopropylphosphate	1
Chlorodiphenyl (54% Chlorine)	1
sulfamic acid	1
Dodecanol	1
dibutyl phenylphosphate	1
pyrofix 2	1
dichlorodifluoromethane	1
3,5-dibromo-2-(2,4-dibromophenoxy)phenol	1
1,3-dichloro-2-propanol	1
tetrabromophthalic anhydride	1
2,3,7,8-tetrabromodibenzo-4-dioxin	1

2,3,3',4,4'5,6-heptabromobiphenyl	1
cellulose acetate-butyrate	1
Chlordan	1
nitrofen	1
4,4'-diphenylmethane diisocyanate	1
Polytetrafluoroethylene	1
vinyl bromide	1
Sulfur Hexafluoride	1
torflam	1
Methoxychlor	1
2,3',4,5',6-pentabromodiphenyl ether	1

Detail

DSSToxID	PREFERRED_NAME	MeSH Chemical Name (hyperlinked)
DTXSID001016751	Octabromobiphenyl	octabromobiphenyl
DTXSID001016751	Octabromobiphenyl	octabromobiphenyl
DTXSID0021173	Firemaster FF-1	Firemaster FF-1
DTXSID0021173	Firemaster FF-1	Firemaster FF-1
DTXSID0021331	Tetrakis(hydroxymethyl)phosphonium	tetramethylolphosphonium chloride
DTXSID0021331	Tetrakis(hydroxymethyl)phosphonium	tetramethylolphosphonium chloride
DTXSID0027147	Methane, bromochlorodifluoro-	bromochlorodifluoromethane
DTXSID0027147	Methane, bromochlorodifluoro-	bromochlorodifluoromethane
DTXSID0027195	Hexadecane	n-hexadecane
DTXSID0027195	Hexadecane	n-hexadecane
DTXSID0049814	Vinyl acetate ethylene copolymer	ethylenevinylacetate copolymer
DTXSID0049814	Vinyl acetate ethylene copolymer	ethylenevinylacetate copolymer
DTXSID0049814	Vinyl acetate ethylene copolymer	ethylenevinylacetate copolymer
DTXSID0052700	2,2'-[(1-Methylethylidene)bis[(2,6-dibr	tetrabromobisphenol A diglycidyl ether
DTXSID0052700	2,2'-[(1-Methylethylidene)bis[(2,6-dibr	tetrabromobisphenol A diglycidyl ether
DTXSID0052700	2,2'-[(1-Methylethylidene)bis[(2,6-dibr	tetrabromobisphenol A diglycidyl ether
DTXSID0052750	1,2,5,6-Tetrabromocyclooctane	1,2,5,6-tetrabromocyclooctane
DTXSID0052750	1,2,5,6-Tetrabromocyclooctane	1,2,5,6-tetrabromocyclooctane
DTXSID0052750	1,2,5,6-Tetrabromocyclooctane	1,2,5,6-tetrabromocyclooctane
DTXSID0052750	1,2,5,6-Tetrabromocyclooctane	1,2,5,6-tetrabromocyclooctane
DTXSID0060561	Phenol, 2,6-dibromo-	2,6-dibromophenol
DTXSID0060561	Phenol, 2,6-dibromo-	2,6-dibromophenol
DTXSID00894087	Bromkal 70	Bromkal 70
DTXSID00894087	Bromkal 70	Bromkal 70
DTXSID00955980	Bis(2,3-dibromopropyl) hydrogen phos	bis(2,3-dibromopropyl)phosphate
DTXSID00955980	Bis(2,3-dibromopropyl) hydrogen phos	bis(2,3-dibromopropyl)phosphate
DTXSID00955980	Bis(2,3-dibromopropyl) hydrogen phos	bis(2,3-dibromopropyl)phosphate
DTXSID1020273	Chlorine	Chlorine
DTXSID1021403	Trimethyl phosphate	trimethyl phosphate
DTXSID10214757	Ammelide	ammelide
DTXSID10214757	Ammelide	ammelide
DTXSID10218114	2,4,5,3',4'-Pentabromobiphenyl	2,4,5,3',4'-pentabromobiphenyl
DTXSID1021952	Triphenyl phosphate	triphenyl phosphate
DTXSID1021952	Triphenyl phosphate	triphenyl phosphate
DTXSID1021952	Triphenyl phosphate	triphenyl phosphate
DTXSID1021952	Triphenyl phosphate	triphenyl phosphate
DTXSID1021952	Triphenyl phosphate	triphenyl phosphate
DTXSID1021952	Triphenyl phosphate	triphenyl phosphate
DTXSID1021952	Triphenyl phosphate	triphenyl phosphate
DTXSID1021952	Triphenyl phosphate	triphenyl phosphate
DTXSID1021952	Triphenyl phosphate	triphenyl phosphate
DTXSID1021952	Triphenyl phosphate	triphenyl phosphate
DTXSID1021952	Triphenyl phosphate	triphenyl phosphate

DTXSID1021952	Triphenyl phosphate	triphenyl phosphate
DTXSID1021952	Triphenyl phosphate	triphenyl phosphate
DTXSID1021952	Triphenyl phosphate	triphenyl phosphate
DTXSID1021952	Triphenyl phosphate	triphenyl phosphate
DTXSID1021952	Triphenyl phosphate	triphenyl phosphate
DTXSID1021952	Triphenyl phosphate	triphenyl phosphate
DTXSID1021952	Triphenyl phosphate	triphenyl phosphate
DTXSID1021952	Triphenyl phosphate	triphenyl phosphate
DTXSID1021952	Triphenyl phosphate	triphenyl phosphate
DTXSID1021952	Triphenyl phosphate	triphenyl phosphate
DTXSID1021952	Triphenyl phosphate	triphenyl phosphate
DTXSID1021952	Triphenyl phosphate	triphenyl phosphate
DTXSID1021952	Triphenyl phosphate	triphenyl phosphate
DTXSID1021952	Triphenyl phosphate	triphenyl phosphate
DTXSID1021952	Triphenyl phosphate	triphenyl phosphate
DTXSID1021952	Triphenyl phosphate	triphenyl phosphate
DTXSID1021952	Triphenyl phosphate	triphenyl phosphate
DTXSID1021952	Triphenyl phosphate	triphenyl phosphate
DTXSID1021952	Triphenyl phosphate	triphenyl phosphate
DTXSID1021952	Triphenyl phosphate	triphenyl phosphate
DTXSID1021952	Triphenyl phosphate	triphenyl phosphate
DTXSID1021952	Triphenyl phosphate	triphenyl phosphate
DTXSID1021952	Triphenyl phosphate	triphenyl phosphate
DTXSID1021952	Triphenyl phosphate	triphenyl phosphate
DTXSID1021952	Triphenyl phosphate	triphenyl phosphate
DTXSID1021952	Triphenyl phosphate	triphenyl phosphate
DTXSID1021952	Triphenyl phosphate	triphenyl phosphate
DTXSID1021952	Triphenyl phosphate	triphenyl phosphate
DTXSID1021952	Triphenyl phosphate	triphenyl phosphate
DTXSID1021952	Triphenyl phosphate	triphenyl phosphate
DTXSID1021952	Triphenyl phosphate	triphenyl phosphate
DTXSID1021952	Triphenyl phosphate	triphenyl phosphate
DTXSID1021952	Triphenyl phosphate	triphenyl phosphate
DTXSID1021952	Triphenyl phosphate	triphenyl phosphate
DTXSID1021952	Triphenyl phosphate	triphenyl phosphate
DTXSID10228273	2,3,3',4,4',5-Hexabromobiphenyl	2,3,3',4,4',5-hexabromobiphenyl
DTXSID1024128	Hexabromobenzene	hexabromobenzene
DTXSID1024128	Hexabromobenzene	hexabromobenzene
DTXSID1024128	Hexabromobenzene	hexabromobenzene
DTXSID1024128	Hexabromobenzene	hexabromobenzene
DTXSID1024128	Hexabromobenzene	hexabromobenzene
DTXSID1024128	Hexabromobenzene	hexabromobenzene
DTXSID1024128	Hexabromobenzene	hexabromobenzene
DTXSID1024128	Hexabromobenzene	hexabromobenzene
DTXSID1024128	Hexabromobenzene	hexabromobenzene
DTXSID1024128	Hexabromobenzene	hexabromobenzene
DTXSID1024128	Hexabromobenzene	hexabromobenzene

DTXSID1024128	Hexabromobenzene	hexabromobenzene
DTXSID1024128	Hexabromobenzene	hexabromobenzene
DTXSID1024382	Phosphorus	Phosphorus
DTXSID1024382	Phosphorus	Phosphorus
DTXSID1024382	Phosphorus	Phosphorus
DTXSID1024382	Phosphorus	Phosphorus
DTXSID1024382	Phosphorus	Phosphorus
DTXSID1024382	Phosphorus	Phosphorus
DTXSID1024382	Phosphorus	Phosphorus
DTXSID1024382	Phosphorus	Phosphorus
DTXSID1024382	Phosphorus	Phosphorus
DTXSID1024382	Phosphorus	Phosphorus
DTXSID1024382	Phosphorus	Phosphorus
DTXSID1024627	1,2-Bis(2,4,6-tribromophenoxy)ethane	1,2-bis(2,4,6-tribromophenoxy)ethane
DTXSID1024627	1,2-Bis(2,4,6-tribromophenoxy)ethane	1,2-bis(2,4,6-tribromophenoxy)ethane
DTXSID1024627	1,2-Bis(2,4,6-tribromophenoxy)ethane	1,2-bis(2,4,6-tribromophenoxy)ethane
DTXSID1024627	1,2-Bis(2,4,6-tribromophenoxy)ethane	1,2-bis(2,4,6-tribromophenoxy)ethane
DTXSID1024627	1,2-Bis(2,4,6-tribromophenoxy)ethane	1,2-bis(2,4,6-tribromophenoxy)ethane
DTXSID1024627	1,2-Bis(2,4,6-tribromophenoxy)ethane	1,2-bis(2,4,6-tribromophenoxy)ethane
DTXSID1024627	1,2-Bis(2,4,6-tribromophenoxy)ethane	1,2-bis(2,4,6-tribromophenoxy)ethane
DTXSID1024627	1,2-Bis(2,4,6-tribromophenoxy)ethane	1,2-bis(2,4,6-tribromophenoxy)ethane
DTXSID1024627	1,2-Bis(2,4,6-tribromophenoxy)ethane	1,2-bis(2,4,6-tribromophenoxy)ethane
DTXSID1024627	1,2-Bis(2,4,6-tribromophenoxy)ethane	1,2-bis(2,4,6-tribromophenoxy)ethane
DTXSID1024627	1,2-Bis(2,4,6-tribromophenoxy)ethane	1,2-bis(2,4,6-tribromophenoxy)ethane
DTXSID1024627	1,2-Bis(2,4,6-tribromophenoxy)ethane	1,2-bis(2,4,6-tribromophenoxy)ethane
DTXSID1024627	1,2-Bis(2,4,6-tribromophenoxy)ethane	1,2-bis(2,4,6-tribromophenoxy)ethane
DTXSID1024627	1,2-Bis(2,4,6-tribromophenoxy)ethane	1,2-bis(2,4,6-tribromophenoxy)ethane
DTXSID1024627	1,2-Bis(2,4,6-tribromophenoxy)ethane	1,2-bis(2,4,6-tribromophenoxy)ethane
DTXSID1025300	2-Ethylhexyl diphenyl phosphate	2-ethylhexyldiphenylphosphate
DTXSID1025300	2-Ethylhexyl diphenyl phosphate	2-ethylhexyldiphenylphosphate
DTXSID1025300	2-Ethylhexyl diphenyl phosphate	2-ethylhexyldiphenylphosphate
DTXSID1026081	3,3',5,5'-Tetrabromobisphenol A	tetrabromobisphenol A
DTXSID1026081	3,3',5,5'-Tetrabromobisphenol A	tetrabromobisphenol A
DTXSID1026081	3,3',5,5'-Tetrabromobisphenol A	tetrabromobisphenol A
DTXSID1026081	3,3',5,5'-Tetrabromobisphenol A	tetrabromobisphenol A
DTXSID1026081	3,3',5,5'-Tetrabromobisphenol A	tetrabromobisphenol A
DTXSID1026081	3,3',5,5'-Tetrabromobisphenol A	tetrabromobisphenol A
DTXSID1026081	3,3',5,5'-Tetrabromobisphenol A	tetrabromobisphenol A
DTXSID1026081	3,3',5,5'-Tetrabromobisphenol A	tetrabromobisphenol A
DTXSID1026081	3,3',5,5'-Tetrabromobisphenol A	tetrabromobisphenol A
DTXSID1026081	3,3',5,5'-Tetrabromobisphenol A	tetrabromobisphenol A
DTXSID1026081	3,3',5,5'-Tetrabromobisphenol A	tetrabromobisphenol A
DTXSID1026081	3,3',5,5'-Tetrabromobisphenol A	tetrabromobisphenol A
DTXSID1026081	3,3',5,5'-Tetrabromobisphenol A	tetrabromobisphenol A
DTXSID1026081	3,3',5,5'-Tetrabromobisphenol A	tetrabromobisphenol A
DTXSID1026081	3,3',5,5'-Tetrabromobisphenol A	tetrabromobisphenol A
DTXSID1026081	3,3',5,5'-Tetrabromobisphenol A	tetrabromobisphenol A

DTXSID1026081	3,3',5,5'-Tetrabromobisphenol A	tetrabromobisphenol A
DTXSID1026081	3,3',5,5'-Tetrabromobisphenol A	tetrabromobisphenol A
DTXSID1026081	3,3',5,5'-Tetrabromobisphenol A	tetrabromobisphenol A
DTXSID1026081	3,3',5,5'-Tetrabromobisphenol A	tetrabromobisphenol A
DTXSID1026081	3,3',5,5'-Tetrabromobisphenol A	tetrabromobisphenol A
DTXSID1026081	3,3',5,5'-Tetrabromobisphenol A	tetrabromobisphenol A
DTXSID1026081	3,3',5,5'-Tetrabromobisphenol A	tetrabromobisphenol A
DTXSID1026081	3,3',5,5'-Tetrabromobisphenol A	tetrabromobisphenol A
DTXSID1026081	3,3',5,5'-Tetrabromobisphenol A	tetrabromobisphenol A
DTXSID1026081	3,3',5,5'-Tetrabromobisphenol A	tetrabromobisphenol A
DTXSID1026081	3,3',5,5'-Tetrabromobisphenol A	tetrabromobisphenol A
DTXSID1026081	3,3',5,5'-Tetrabromobisphenol A	tetrabromobisphenol A
DTXSID1026081	3,3',5,5'-Tetrabromobisphenol A	tetrabromobisphenol A
DTXSID1026081	3,3',5,5'-Tetrabromobisphenol A	tetrabromobisphenol A
DTXSID1026081	3,3',5,5'-Tetrabromobisphenol A	tetrabromobisphenol A
DTXSID1026081	3,3',5,5'-Tetrabromobisphenol A	tetrabromobisphenol A
DTXSID1026081	3,3',5,5'-Tetrabromobisphenol A	tetrabromobisphenol A
DTXSID1026081	3,3',5,5'-Tetrabromobisphenol A	tetrabromobisphenol A
DTXSID1026081	3,3',5,5'-Tetrabromobisphenol A	tetrabromobisphenol A
DTXSID1026081	3,3',5,5'-Tetrabromobisphenol A	tetrabromobisphenol A
DTXSID1026081	3,3',5,5'-Tetrabromobisphenol A	tetrabromobisphenol A
DTXSID1026081	3,3',5,5'-Tetrabromobisphenol A	tetrabromobisphenol A
DTXSID1026081	3,3',5,5'-Tetrabromobisphenol A	tetrabromobisphenol A
DTXSID1026081	3,3',5,5'-Tetrabromobisphenol A	tetrabromobisphenol A
DTXSID1026081	3,3',5,5'-Tetrabromobisphenol A	tetrabromobisphenol A
DTXSID1026081	3,3',5,5'-Tetrabromobisphenol A	tetrabromobisphenol A
DTXSID1026081	3,3',5,5'-Tetrabromobisphenol A	tetrabromobisphenol A
DTXSID1026081	3,3',5,5'-Tetrabromobisphenol A	tetrabromobisphenol A
DTXSID1026081	3,3',5,5'-Tetrabromobisphenol A	tetrabromobisphenol A
DTXSID1026081	3,3',5,5'-Tetrabromobisphenol A	tetrabromobisphenol A
DTXSID1026081	3,3',5,5'-Tetrabromobisphenol A	tetrabromobisphenol A
DTXSID1026081	3,3',5,5'-Tetrabromobisphenol A	tetrabromobisphenol A
DTXSID1026081	3,3',5,5'-Tetrabromobisphenol A	tetrabromobisphenol A
DTXSID1026081	3,3',5,5'-Tetrabromobisphenol A	tetrabromobisphenol A
DTXSID1026081	3,3',5,5'-Tetrabromobisphenol A	tetrabromobisphenol A
DTXSID1026081	3,3',5,5'-Tetrabromobisphenol A	tetrabromobisphenol A
DTXSID1026081	3,3',5,5'-Tetrabromobisphenol A	tetrabromobisphenol A
DTXSID1026081	3,3',5,5'-Tetrabromobisphenol A	tetrabromobisphenol A
DTXSID1026081	3,3',5,5'-Tetrabromobisphenol A	tetrabromobisphenol A
DTXSID1026081	3,3',5,5'-Tetrabromobisphenol A	tetrabromobisphenol A
DTXSID1026081	3,3',5,5'-Tetrabromobisphenol A	tetrabromobisphenol A
DTXSID1026081	3,3',5,5'-Tetrabromobisphenol A	tetrabromobisphenol A
DTXSID1026081	3,3',5,5'-Tetrabromobisphenol A	tetrabromobisphenol A
DTXSID1026081	3,3',5,5'-Tetrabromobisphenol A	tetrabromobisphenol A
DTXSID1026081	3,3',5,5'-Tetrabromobisphenol A	tetrabromobisphenol A
DTXSID1026081	3,3',5,5'-Tetrabromobisphenol A	tetrabromobisphenol A
DTXSID1026081	3,3',5,5'-Tetrabromobisphenol A	tetrabromobisphenol A

DTXSID1026081	3,3',5,5'-Tetrabromobisphenol A	tetrabromobisphenol A
DTXSID1026081	3,3',5,5'-Tetrabromobisphenol A	tetrabromobisphenol A
DTXSID1026081	3,3',5,5'-Tetrabromobisphenol A	tetrabromobisphenol A
DTXSID1026081	3,3',5,5'-Tetrabromobisphenol A	tetrabromobisphenol A
DTXSID1026081	3,3',5,5'-Tetrabromobisphenol A	tetrabromobisphenol A
DTXSID1026081	3,3',5,5'-Tetrabromobisphenol A	tetrabromobisphenol A
DTXSID1026081	3,3',5,5'-Tetrabromobisphenol A	tetrabromobisphenol A
DTXSID1026081	3,3',5,5'-Tetrabromobisphenol A	tetrabromobisphenol A
DTXSID1026081	3,3',5,5'-Tetrabromobisphenol A	tetrabromobisphenol A
DTXSID1026081	3,3',5,5'-Tetrabromobisphenol A	tetrabromobisphenol A
DTXSID1026081	3,3',5,5'-Tetrabromobisphenol A	tetrabromobisphenol A
DTXSID1026081	3,3',5,5'-Tetrabromobisphenol A	tetrabromobisphenol A
DTXSID1026081	3,3',5,5'-Tetrabromobisphenol A	tetrabromobisphenol A
DTXSID1026081	3,3',5,5'-Tetrabromobisphenol A	tetrabromobisphenol A
DTXSID1026081	3,3',5,5'-Tetrabromobisphenol A	tetrabromobisphenol A
DTXSID1026081	3,3',5,5'-Tetrabromobisphenol A	tetrabromobisphenol A
DTXSID1026081	3,3',5,5'-Tetrabromobisphenol A	tetrabromobisphenol A
DTXSID1026081	3,3',5,5'-Tetrabromobisphenol A	tetrabromobisphenol A
DTXSID1026081	3,3',5,5'-Tetrabromobisphenol A	tetrabromobisphenol A
DTXSID1026081	3,3',5,5'-Tetrabromobisphenol A	tetrabromobisphenol A
DTXSID1026081	3,3',5,5'-Tetrabromobisphenol A	tetrabromobisphenol A
DTXSID1026081	3,3',5,5'-Tetrabromobisphenol A	tetrabromobisphenol A
DTXSID1026081	3,3',5,5'-Tetrabromobisphenol A	tetrabromobisphenol A
DTXSID1026081	3,3',5,5'-Tetrabromobisphenol A	tetrabromobisphenol A
DTXSID1026081	3,3',5,5'-Tetrabromobisphenol A	tetrabromobisphenol A
DTXSID1026081	3,3',5,5'-Tetrabromobisphenol A	tetrabromobisphenol A
DTXSID1026081	3,3',5,5'-Tetrabromobisphenol A	tetrabromobisphenol A
DTXSID1026081	3,3',5,5'-Tetrabromobisphenol A	tetrabromobisphenol A
DTXSID1026081	3,3',5,5'-Tetrabromobisphenol A	tetrabromobisphenol A
DTXSID1026081	3,3',5,5'-Tetrabromobisphenol A	tetrabromobisphenol A
DTXSID1026081	3,3',5,5'-Tetrabromobisphenol A	tetrabromobisphenol A
DTXSID1026081	3,3',5,5'-Tetrabromobisphenol A	tetrabromobisphenol A
DTXSID1026081	3,3',5,5'-Tetrabromobisphenol A	tetrabromobisphenol A
DTXSID1026081	3,3',5,5'-Tetrabromobisphenol A	tetrabromobisphenol A
DTXSID1026081	3,3',5,5'-Tetrabromobisphenol A	tetrabromobisphenol A
DTXSID1026081	3,3',5,5'-Tetrabromobisphenol A	tetrabromobisphenol A
DTXSID1026081	3,3',5,5'-Tetrabromobisphenol A	tetrabromobisphenol A
DTXSID1026081	3,3',5,5'-Tetrabromobisphenol A	tetrabromobisphenol A
DTXSID1026081	3,3',5,5'-Tetrabromobisphenol A	tetrabromobisphenol A
DTXSID1026081	3,3',5,5'-Tetrabromobisphenol A	tetrabromobisphenol A
DTXSID1026081	3,3',5,5'-Tetrabromobisphenol A	tetrabromobisphenol A
DTXSID1026081	3,3',5,5'-Tetrabromobisphenol A	tetrabromobisphenol A
DTXSID1026081	3,3',5,5'-Tetrabromobisphenol A	tetrabromobisphenol A
DTXSID1026081	3,3',5,5'-Tetrabromobisphenol A	tetrabromobisphenol A
DTXSID1026081	3,3',5,5'-Tetrabromobisphenol A	tetrabromobisphenol A
DTXSID1026081	3,3',5,5'-Tetrabromobisphenol A	tetrabromobisphenol A
DTXSID1026081	3,3',5,5'-Tetrabromobisphenol A	tetrabromobisphenol A

DTXSID1026081	3,3',5,5'-Tetrabromobisphenol A	tetrabromobisphenol A
DTXSID1026081	3,3',5,5'-Tetrabromobisphenol A	tetrabromobisphenol A
DTXSID1026081	3,3',5,5'-Tetrabromobisphenol A	tetrabromobisphenol A
DTXSID1026081	3,3',5,5'-Tetrabromobisphenol A	tetrabromobisphenol A
DTXSID1026081	3,3',5,5'-Tetrabromobisphenol A	tetrabromobisphenol A
DTXSID1026081	3,3',5,5'-Tetrabromobisphenol A	tetrabromobisphenol A
DTXSID1026081	3,3',5,5'-Tetrabromobisphenol A	tetrabromobisphenol A
DTXSID1026081	3,3',5,5'-Tetrabromobisphenol A	tetrabromobisphenol A
DTXSID1026081	3,3',5,5'-Tetrabromobisphenol A	tetrabromobisphenol A
DTXSID1026081	3,3',5,5'-Tetrabromobisphenol A	tetrabromobisphenol A
DTXSID1026081	3,3',5,5'-Tetrabromobisphenol A	tetrabromobisphenol A
DTXSID1026081	3,3',5,5'-Tetrabromobisphenol A	tetrabromobisphenol A
DTXSID1026081	3,3',5,5'-Tetrabromobisphenol A	tetrabromobisphenol A
DTXSID1026081	3,3',5,5'-Tetrabromobisphenol A	tetrabromobisphenol A
DTXSID1026081	3,3',5,5'-Tetrabromobisphenol A	tetrabromobisphenol A
DTXSID1026081	3,3',5,5'-Tetrabromobisphenol A	tetrabromobisphenol A
DTXSID1026081	3,3',5,5'-Tetrabromobisphenol A	tetrabromobisphenol A
DTXSID1026081	3,3',5,5'-Tetrabromobisphenol A	tetrabromobisphenol A
DTXSID1026081	3,3',5,5'-Tetrabromobisphenol A	tetrabromobisphenol A
DTXSID1026081	3,3',5,5'-Tetrabromobisphenol A	tetrabromobisphenol A
DTXSID1026081	3,3',5,5'-Tetrabromobisphenol A	tetrabromobisphenol A
DTXSID1026081	3,3',5,5'-Tetrabromobisphenol A	tetrabromobisphenol A
DTXSID1026081	3,3',5,5'-Tetrabromobisphenol A	tetrabromobisphenol A
DTXSID1026081	3,3',5,5'-Tetrabromobisphenol A	tetrabromobisphenol A
DTXSID1026081	3,3',5,5'-Tetrabromobisphenol A	tetrabromobisphenol A
DTXSID1026081	3,3',5,5'-Tetrabromobisphenol A	tetrabromobisphenol A
DTXSID1026081	3,3',5,5'-Tetrabromobisphenol A	tetrabromobisphenol A
DTXSID1026081	3,3',5,5'-Tetrabromobisphenol A	tetrabromobisphenol A
DTXSID1026081	3,3',5,5'-Tetrabromobisphenol A	tetrabromobisphenol A
DTXSID1026081	3,3',5,5'-Tetrabromobisphenol A	tetrabromobisphenol A
DTXSID1026081	3,3',5,5'-Tetrabromobisphenol A	tetrabromobisphenol A
DTXSID1026081	3,3',5,5'-Tetrabromobisphenol A	tetrabromobisphenol A
DTXSID1026081	3,3',5,5'-Tetrabromobisphenol A	tetrabromobisphenol A
DTXSID1026081	3,3',5,5'-Tetrabromobisphenol A	tetrabromobisphenol A
DTXSID1026081	3,3',5,5'-Tetrabromobisphenol A	tetrabromobisphenol A
DTXSID1026081	3,3',5,5'-Tetrabromobisphenol A	tetrabromobisphenol A
DTXSID1026081	3,3',5,5'-Tetrabromobisphenol A	tetrabromobisphenol A
DTXSID1026081	3,3',5,5'-Tetrabromobisphenol A	tetrabromobisphenol A
DTXSID1026081	3,3',5,5'-Tetrabromobisphenol A	tetrabromobisphenol A
DTXSID1026081	3,3',5,5'-Tetrabromobisphenol A	tetrabromobisphenol A
DTXSID1026081	3,3',5,5'-Tetrabromobisphenol A	tetrabromobisphenol A
DTXSID1026081	3,3',5,5'-Tetrabromobisphenol A	tetrabromobisphenol A
DTXSID1026081	3,3',5,5'-Tetrabromobisphenol A	tetrabromobisphenol A
DTXSID1026081	3,3',5,5'-Tetrabromobisphenol A	tetrabromobisphenol A
DTXSID1026081	3,3',5,5'-Tetrabromobisphenol A	tetrabromobisphenol A
DTXSID1026081	3,3',5,5'-Tetrabromobisphenol A	tetrabromobisphenol A
DTXSID1026081	3,3',5,5'-Tetrabromobisphenol A	tetrabromobisphenol A

DTXSID1026081	3,3',5,5'-Tetrabromobisphenol A	tetrabromobisphenol A
DTXSID1026081	3,3',5,5'-Tetrabromobisphenol A	tetrabromobisphenol A
DTXSID1026081	3,3',5,5'-Tetrabromobisphenol A	tetrabromobisphenol A
DTXSID1026081	3,3',5,5'-Tetrabromobisphenol A	tetrabromobisphenol A
DTXSID1026081	3,3',5,5'-Tetrabromobisphenol A	tetrabromobisphenol A
DTXSID1026081	3,3',5,5'-Tetrabromobisphenol A	tetrabromobisphenol A
DTXSID1026081	3,3',5,5'-Tetrabromobisphenol A	tetrabromobisphenol A
DTXSID1026081	3,3',5,5'-Tetrabromobisphenol A	tetrabromobisphenol A
DTXSID1026081	3,3',5,5'-Tetrabromobisphenol A	tetrabromobisphenol A
DTXSID1026081	3,3',5,5'-Tetrabromobisphenol A	tetrabromobisphenol A
DTXSID1026081	3,3',5,5'-Tetrabromobisphenol A	tetrabromobisphenol A
DTXSID1026081	3,3',5,5'-Tetrabromobisphenol A	tetrabromobisphenol A
DTXSID1026081	3,3',5,5'-Tetrabromobisphenol A	tetrabromobisphenol A
DTXSID1026081	3,3',5,5'-Tetrabromobisphenol A	tetrabromobisphenol A
DTXSID1026081	3,3',5,5'-Tetrabromobisphenol A	tetrabromobisphenol A
DTXSID1026081	3,3',5,5'-Tetrabromobisphenol A	tetrabromobisphenol A
DTXSID1026081	3,3',5,5'-Tetrabromobisphenol A	tetrabromobisphenol A
DTXSID1026081	3,3',5,5'-Tetrabromobisphenol A	tetrabromobisphenol A
DTXSID1026081	3,3',5,5'-Tetrabromobisphenol A	tetrabromobisphenol A
DTXSID1026081	3,3',5,5'-Tetrabromobisphenol A	tetrabromobisphenol A
DTXSID1026081	3,3',5,5'-Tetrabromobisphenol A	tetrabromobisphenol A
DTXSID1026081	3,3',5,5'-Tetrabromobisphenol A	tetrabromobisphenol A
DTXSID1026081	3,3',5,5'-Tetrabromobisphenol A	tetrabromobisphenol A
DTXSID1026081	3,3',5,5'-Tetrabromobisphenol A	tetrabromobisphenol A
DTXSID1026081	3,3',5,5'-Tetrabromobisphenol A	tetrabromobisphenol A
DTXSID1026081	3,3',5,5'-Tetrabromobisphenol A	tetrabromobisphenol A
DTXSID1026081	3,3',5,5'-Tetrabromobisphenol A	tetrabromobisphenol A
DTXSID1026081	3,3',5,5'-Tetrabromobisphenol A	tetrabromobisphenol A
DTXSID1026081	3,3',5,5'-Tetrabromobisphenol A	tetrabromobisphenol A
DTXSID1026081	3,3',5,5'-Tetrabromobisphenol A	tetrabromobisphenol A
DTXSID1026081	3,3',5,5'-Tetrabromobisphenol A	tetrabromobisphenol A
DTXSID1026081	3,3',5,5'-Tetrabromobisphenol A	tetrabromobisphenol A
DTXSID1026081	3,3',5,5'-Tetrabromobisphenol A	tetrabromobisphenol A
DTXSID1026081	3,3',5,5'-Tetrabromobisphenol A	tetrabromobisphenol A
DTXSID1026081	3,3',5,5'-Tetrabromobisphenol A	tetrabromobisphenol A
DTXSID1026081	3,3',5,5'-Tetrabromobisphenol A	tetrabromobisphenol A
DTXSID1026081	3,3',5,5'-Tetrabromobisphenol A	tetrabromobisphenol A
DTXSID1026081	3,3',5,5'-Tetrabromobisphenol A	tetrabromobisphenol A
DTXSID1026081	3,3',5,5'-Tetrabromobisphenol A	tetrabromobisphenol A
DTXSID1026081	3,3',5,5'-Tetrabromobisphenol A	tetrabromobisphenol A
DTXSID1026081	3,3',5,5'-Tetrabromobisphenol A	tetrabromobisphenol A
DTXSID1026081	3,3',5,5'-Tetrabromobisphenol A	tetrabromobisphenol A
DTXSID1026081	3,3',5,5'-Tetrabromobisphenol A	tetrabromobisphenol A
DTXSID1026081	3,3',5,5'-Tetrabromobisphenol A	tetrabromobisphenol A
DTXSID1026081	3,3',5,5'-Tetrabromobisphenol A	tetrabromobisphenol A
DTXSID1026081	3,3',5,5'-Tetrabromobisphenol A	tetrabromobisphenol A
DTXSID1026081	3,3',5,5'-Tetrabromobisphenol A	tetrabromobisphenol A

DTXSID1026081	3,3',5,5'-Tetrabromobisphenol A	tetrabromobisphenol A
DTXSID1026081	3,3',5,5'-Tetrabromobisphenol A	tetrabromobisphenol A
DTXSID1026081	3,3',5,5'-Tetrabromobisphenol A	tetrabromobisphenol A
DTXSID1026081	3,3',5,5'-Tetrabromobisphenol A	tetrabromobisphenol A
DTXSID1026081	3,3',5,5'-Tetrabromobisphenol A	tetrabromobisphenol A
DTXSID1026081	3,3',5,5'-Tetrabromobisphenol A	tetrabromobisphenol A
DTXSID1026081	3,3',5,5'-Tetrabromobisphenol A	tetrabromobisphenol A
DTXSID1026081	3,3',5,5'-Tetrabromobisphenol A	tetrabromobisphenol A
DTXSID1026081	3,3',5,5'-Tetrabromobisphenol A	tetrabromobisphenol A
DTXSID1026081	3,3',5,5'-Tetrabromobisphenol A	tetrabromobisphenol A
DTXSID1026081	3,3',5,5'-Tetrabromobisphenol A	tetrabromobisphenol A
DTXSID1026081	3,3',5,5'-Tetrabromobisphenol A	tetrabromobisphenol A
DTXSID1026081	3,3',5,5'-Tetrabromobisphenol A	tetrabromobisphenol A
DTXSID1026081	3,3',5,5'-Tetrabromobisphenol A	tetrabromobisphenol A
DTXSID1026081	3,3',5,5'-Tetrabromobisphenol A	tetrabromobisphenol A
DTXSID1026081	3,3',5,5'-Tetrabromobisphenol A	tetrabromobisphenol A
DTXSID1026081	3,3',5,5'-Tetrabromobisphenol A	tetrabromobisphenol A
DTXSID1026081	3,3',5,5'-Tetrabromobisphenol A	tetrabromobisphenol A
DTXSID1026081	3,3',5,5'-Tetrabromobisphenol A	tetrabromobisphenol A
DTXSID1026081	3,3',5,5'-Tetrabromobisphenol A	tetrabromobisphenol A
DTXSID1026081	3,3',5,5'-Tetrabromobisphenol A	tetrabromobisphenol A
DTXSID1026081	3,3',5,5'-Tetrabromobisphenol A	tetrabromobisphenol A
DTXSID1026081	3,3',5,5'-Tetrabromobisphenol A	tetrabromobisphenol A
DTXSID1026081	3,3',5,5'-Tetrabromobisphenol A	tetrabromobisphenol A
DTXSID1026081	3,3',5,5'-Tetrabromobisphenol A	tetrabromobisphenol A
DTXSID1026081	3,3',5,5'-Tetrabromobisphenol A	tetrabromobisphenol A
DTXSID1026081	3,3',5,5'-Tetrabromobisphenol A	tetrabromobisphenol A
DTXSID1026081	3,3',5,5'-Tetrabromobisphenol A	tetrabromobisphenol A
DTXSID1026081	3,3',5,5'-Tetrabromobisphenol A	tetrabromobisphenol A
DTXSID1026081	3,3',5,5'-Tetrabromobisphenol A	tetrabromobisphenol A
DTXSID1026081	3,3',5,5'-Tetrabromobisphenol A	tetrabromobisphenol A
DTXSID1026081	3,3',5,5'-Tetrabromobisphenol A	tetrabromobisphenol A
DTXSID1026081	3,3',5,5'-Tetrabromobisphenol A	tetrabromobisphenol A
DTXSID1026081	3,3',5,5'-Tetrabromobisphenol A	tetrabromobisphenol A
DTXSID1026081	3,3',5,5'-Tetrabromobisphenol A	tetrabromobisphenol A
DTXSID1026081	3,3',5,5'-Tetrabromobisphenol A	tetrabromobisphenol A
DTXSID1026081	3,3',5,5'-Tetrabromobisphenol A	tetrabromobisphenol A
DTXSID1026081	3,3',5,5'-Tetrabromobisphenol A	tetrabromobisphenol A
DTXSID1026081	3,3',5,5'-Tetrabromobisphenol A	tetrabromobisphenol A
DTXSID1026081	3,3',5,5'-Tetrabromobisphenol A	tetrabromobisphenol A
DTXSID1026081	3,3',5,5'-Tetrabromobisphenol A	tetrabromobisphenol A
DTXSID1026081	3,3',5,5'-Tetrabromobisphenol A	tetrabromobisphenol A
DTXSID1026081	3,3',5,5'-Tetrabromobisphenol A	tetrabromobisphenol A
DTXSID1029677	Silica	Silicon Dioxide
DTXSID1029677	Silica	Silicon Dioxide
DTXSID1029677	Silica	Silicon Dioxide
DTXSID1035238	Bromine	Bromine

DTXSID1035238	Bromine	Bromine
DTXSID1035238	Bromine	Bromine
DTXSID1035238	Bromine	Bromine
DTXSID1035238	Bromine	Bromine
DTXSID1035238	Bromine	Bromine
DTXSID1035238	Bromine	Bromine
DTXSID1035238	Bromine	Bromine
DTXSID1035238	Bromine	Bromine
DTXSID1035238	Bromine	Bromine
DTXSID1035238	Bromine	Bromine
DTXSID1035238	Bromine	Bromine
DTXSID1035238	Bromine	Bromine
DTXSID1035238	Bromine	Bromine
DTXSID1035238	Bromine	Bromine
DTXSID1035238	Bromine	Bromine
DTXSID1035238	Bromine	Bromine
DTXSID1035238	Bromine	Bromine
DTXSID1035238	Bromine	Bromine
DTXSID1035238	Bromine	Bromine
DTXSID1035238	Bromine	Bromine
DTXSID1035238	Bromine	Bromine
DTXSID1035238	Bromine	Bromine
DTXSID1035238	Bromine	Bromine
DTXSID1035238	Bromine	Bromine
DTXSID1035238	Bromine	Bromine
DTXSID1035238	Bromine	Bromine
DTXSID1035238	Bromine	Bromine
DTXSID1035238	Bromine	Bromine
DTXSID1035238	Bromine	Bromine
DTXSID1035238	Bromine	Bromine
DTXSID1035238	Bromine	Bromine
DTXSID1035238	Bromine	Bromine
DTXSID1035238	Bromine	Bromine
DTXSID1035238	Bromine	Bromine
DTXSID1035238	Bromine	Bromine
DTXSID1035238	Bromine	Bromine
DTXSID1035238	Bromine	Bromine
DTXSID1035238	Bromine	Bromine
DTXSID1035238	Bromine	Bromine
DTXSID1035238	Bromine	Bromine
DTXSID1035238	Bromine	Bromine
DTXSID1035238	Bromine	Bromine
DTXSID1035238	Bromine	Bromine
DTXSID1035238	Bromine	Bromine
DTXSID1035238	Bromine	Bromine
DTXSID1035238	Bromine	Bromine
DTXSID1035238	Bromine	Bromine

DTXSID1035238	Bromine	Bromine
DTXSID1035238	Bromine	Bromine
DTXSID1035238	Bromine	Bromine
DTXSID1035238	Bromine	Bromine
DTXSID1035238	Bromine	Bromine
DTXSID1035238	Bromine	Bromine
DTXSID1044699	Diethyl hydrogen phosphate	diethyl phosphate
DTXSID1052290	2,4-Dibromophenol	2,4-dibromophenol
DTXSID1052290	2,4-Dibromophenol	2,4-dibromophenol
DTXSID1052290	2,4-Dibromophenol	2,4-dibromophenol
DTXSID1061073	Heptafluoropropyl iodide	1,1,2,2,3,3,3-heptafluoro-1-iodopropane
DTXSID1064746	Phosphonium, tetrakis(hydroxymethyl	tetramethylolphosphonium chloride
DTXSID1064746	Phosphonium, tetrakis(hydroxymethyl	tetramethylolphosphonium chloride
DTXSID1068720	Phosphonium, tetrakis(hydroxymethyl	tetramethylolphosphonium chloride
DTXSID1068720	Phosphonium, tetrakis(hydroxymethyl	tetramethylolphosphonium chloride
DTXSID10865889	Tetrabromobisphenol A dimethyl ethe	tetrabromobisphenol A dimethyl ether
DTXSID10865889	Tetrabromobisphenol A dimethyl ethe	tetrabromobisphenol A dimethyl ether
DTXSID10865889	Tetrabromobisphenol A dimethyl ethe	tetrabromobisphenol A dimethyl ether
DTXSID10865889	Tetrabromobisphenol A dimethyl ethe	tetrabromobisphenol A dimethyl ether
DTXSID10873929	2,2',4,4',6,6'-Hexabromodiphenyl Ethe	hexabromodiphenyl ether 154
DTXSID10873929	2,2',4,4',6,6'-Hexabromodiphenyl Ethe	hexabromodiphenyl ether 154
DTXSID10873929	2,2',4,4',6,6'-Hexabromodiphenyl Ethe	hexabromodiphenyl ether 154
DTXSID10987252	2,3,4,5,2',3',4',5'-Octabromobiphenyl	octabromobiphenyl
DTXSID10987252	2,3,4,5,2',3',4',5'-Octabromobiphenyl	octabromobiphenyl
DTXSID201009900	2,2',5,5'-Tetrabromobiphenyl	2,2',5,5'-tetrabromobiphenyl
DTXSID201016652	Tris(chloropropyl)phosphate	Fyrol PCF
DTXSID201016652	Tris(chloropropyl)phosphate	Fyrol PCF
DTXSID201016652	Tris(chloropropyl)phosphate	Fyrol PCF
DTXSID201016652	Tris(chloropropyl)phosphate	Fyrol PCF
DTXSID201016652	Tris(chloropropyl)phosphate	Fyrol PCF
DTXSID201016652	Tris(chloropropyl)phosphate	Fyrol PCF
DTXSID201016652	Tris(chloropropyl)phosphate	Fyrol PCF
DTXSID201016652	Tris(chloropropyl)phosphate	Fyrol PCF
DTXSID2020268	Chlorendic acid	chlorendic acid
DTXSID2020686	Lindane	Hexachlorocyclohexane
DTXSID2020688	Hexachlorocyclopentadiene	hexachlorocyclopentadiene
DTXSID2021315	2,3,7,8-Tetrachlorodibenzo-p-dioxin	Polychlorinated Dibenzodioxins
DTXSID2021315	2,3,7,8-Tetrachlorodibenzo-p-dioxin	Polychlorinated Dibenzodioxins
DTXSID2021315	2,3,7,8-Tetrachlorodibenzo-p-dioxin	Polychlorinated Dibenzodioxins
DTXSID2021315	2,3,7,8-Tetrachlorodibenzo-p-dioxin	Polychlorinated Dibenzodioxins
DTXSID2021315	2,3,7,8-Tetrachlorodibenzo-p-dioxin	Polychlorinated Dibenzodioxins
DTXSID2021315	2,3,7,8-Tetrachlorodibenzo-p-dioxin	Polychlorinated Dibenzodioxins
DTXSID2021315	2,3,7,8-Tetrachlorodibenzo-p-dioxin	Polychlorinated Dibenzodioxins
DTXSID2021315	2,3,7,8-Tetrachlorodibenzo-p-dioxin	Polychlorinated Dibenzodioxins
DTXSID2021315	2,3,7,8-Tetrachlorodibenzo-p-dioxin	Polychlorinated Dibenzodioxins
DTXSID2021315	2,3,7,8-Tetrachlorodibenzo-p-dioxin	Polychlorinated Dibenzodioxins
DTXSID2021315	2,3,7,8-Tetrachlorodibenzo-p-dioxin	Polychlorinated Dibenzodioxins

DTXSID2021315	2,3,7,8-Tetrachlorodibenzo-p-dioxin	Polychlorinated Dibenzodioxins
DTXSID2024246	Pentabromodiphenyl ether	pentabromodiphenyl ether
DTXSID2024246	Pentabromodiphenyl ether	pentabromodiphenyl ether
DTXSID2024246	Pentabromodiphenyl ether	pentabromodiphenyl ether
DTXSID2024246	Pentabromodiphenyl ether	pentabromodiphenyl ether
DTXSID2024246	Pentabromodiphenyl ether	pentabromodiphenyl ether
DTXSID2024246	Pentabromodiphenyl ether	pentabromodiphenyl ether
DTXSID2024246	Pentabromodiphenyl ether	pentabromodiphenyl ether
DTXSID2024246	Pentabromodiphenyl ether	pentabromodiphenyl ether
DTXSID2024246	Pentabromodiphenyl ether	pentabromodiphenyl ether
DTXSID2024246	Pentabromodiphenyl ether	pentabromodiphenyl ether
DTXSID2024246	Pentabromodiphenyl ether	pentabromodiphenyl ether
DTXSID2024246	Pentabromodiphenyl ether	pentabromodiphenyl ether
DTXSID2024246	Pentabromodiphenyl ether	pentabromodiphenyl ether
DTXSID2024246	Pentabromodiphenyl ether	pentabromodiphenyl ether
DTXSID2024246	Pentabromodiphenyl ether	pentabromodiphenyl ether
DTXSID2024246	Pentabromodiphenyl ether	pentabromodiphenyl ether
DTXSID2024246	Pentabromodiphenyl ether	pentabromodiphenyl ether
DTXSID2024246	Pentabromodiphenyl ether	pentabromodiphenyl ether
DTXSID2024246	Pentabromodiphenyl ether	pentabromodiphenyl ether
DTXSID2024246	Pentabromodiphenyl ether	pentabromodiphenyl ether
DTXSID2024246	Pentabromodiphenyl ether	pentabromodiphenyl ether
DTXSID2024246	Pentabromodiphenyl ether	pentabromodiphenyl ether
DTXSID2024246	Pentabromodiphenyl ether	pentabromodiphenyl ether
DTXSID2024246	Pentabromodiphenyl ether	pentabromodiphenyl ether
DTXSID2024246	Pentabromodiphenyl ether	pentabromodiphenyl ether
DTXSID2024246	Pentabromodiphenyl ether	pentabromodiphenyl ether
DTXSID2024246	Pentabromodiphenyl ether	pentabromodiphenyl ether
DTXSID2024246	Pentabromodiphenyl ether	pentabromodiphenyl ether
DTXSID2024246	Pentabromodiphenyl ether	pentabromodiphenyl ether
DTXSID2024246	Pentabromodiphenyl ether	pentabromodiphenyl ether
DTXSID2024246	Pentabromodiphenyl ether	pentabromodiphenyl ether
DTXSID2024246	Pentabromodiphenyl ether	pentabromodiphenyl ether
DTXSID2024246	Pentabromodiphenyl ether	pentabromodiphenyl ether
DTXSID2024246	Pentabromodiphenyl ether	pentabromodiphenyl ether
DTXSID2024246	Pentabromodiphenyl ether	pentabromodiphenyl ether
DTXSID2024246	Pentabromodiphenyl ether	pentabromodiphenyl ether
DTXSID2024246	Pentabromodiphenyl ether	pentabromodiphenyl ether
DTXSID2024246	Pentabromodiphenyl ether	pentabromodiphenyl ether
DTXSID2024246	Pentabromodiphenyl ether	pentabromodiphenyl ether
DTXSID2024246	Pentabromodiphenyl ether	pentabromodiphenyl ether
DTXSID2024246	Pentabromodiphenyl ether	pentabromodiphenyl ether
DTXSID2024246	Pentabromodiphenyl ether	pentabromodiphenyl ether
DTXSID2024246	Pentabromodiphenyl ether	pentabromodiphenyl ether
DTXSID2024246	Pentabromodiphenyl ether	pentabromodiphenyl ether
DTXSID2024246	Pentabromodiphenyl ether	pentabromodiphenyl ether
DTXSID2024246	Pentabromodiphenyl ether	pentabromodiphenyl ether

DTXSID2024246	Pentabromodiphenyl ether	pentabromodiphenyl ether
DTXSID2024246	Pentabromodiphenyl ether	pentabromodiphenyl ether
DTXSID2024246	Pentabromodiphenyl ether	pentabromodiphenyl ether
DTXSID2024246	Pentabromodiphenyl ether	pentabromodiphenyl ether
DTXSID2024246	Pentabromodiphenyl ether	pentabromodiphenyl ether
DTXSID2024246	Pentabromodiphenyl ether	pentabromodiphenyl ether
DTXSID2024246	Pentabromodiphenyl ether	pentabromodiphenyl ether
DTXSID2024246	Pentabromodiphenyl ether	pentabromodiphenyl ether
DTXSID2024246	Pentabromodiphenyl ether	pentabromodiphenyl ether
DTXSID2024246	Pentabromodiphenyl ether	pentabromodiphenyl ether
DTXSID2024246	Pentabromodiphenyl ether	pentabromodiphenyl ether
DTXSID2024246	Pentabromodiphenyl ether	pentabromodiphenyl ether
DTXSID2024246	Pentabromodiphenyl ether	pentabromodiphenyl ether
DTXSID2024246	Pentabromodiphenyl ether	pentabromodiphenyl ether
DTXSID2024246	Pentabromodiphenyl ether	pentabromodiphenyl ether
DTXSID2024246	Pentabromodiphenyl ether	pentabromodiphenyl ether
DTXSID2024246	Pentabromodiphenyl ether	pentabromodiphenyl ether
DTXSID2024246	Pentabromodiphenyl ether	pentabromodiphenyl ether
DTXSID2024246	Pentabromodiphenyl ether	pentabromodiphenyl ether
DTXSID2024246	Pentabromodiphenyl ether	pentabromodiphenyl ether
DTXSID2024246	Pentabromodiphenyl ether	pentabromodiphenyl ether
DTXSID2024246	Pentabromodiphenyl ether	pentabromodiphenyl ether
DTXSID2024246	Pentabromodiphenyl ether	pentabromodiphenyl ether
DTXSID2024246	Pentabromodiphenyl ether	pentabromodiphenyl ether
DTXSID2024246	Pentabromodiphenyl ether	pentabromodiphenyl ether
DTXSID2024246	Pentabromodiphenyl ether	pentabromodiphenyl ether
DTXSID2024246	Pentabromodiphenyl ether	pentabromodiphenyl ether
DTXSID2024246	Pentabromodiphenyl ether	pentabromodiphenyl ether
DTXSID2024246	Pentabromodiphenyl ether	pentabromodiphenyl ether
DTXSID2024246	Pentabromodiphenyl ether	pentabromodiphenyl ether
DTXSID2024246	Pentabromodiphenyl ether	pentabromodiphenyl ether
DTXSID2024246	Pentabromodiphenyl ether	pentabromodiphenyl ether
DTXSID2024246	Pentabromodiphenyl ether	pentabromodiphenyl ether
DTXSID2024246	Pentabromodiphenyl ether	pentabromodiphenyl ether
DTXSID2024246	Pentabromodiphenyl ether	pentabromodiphenyl ether
DTXSID2024246	Pentabromodiphenyl ether	pentabromodiphenyl ether
DTXSID2024246	Pentabromodiphenyl ether	pentabromodiphenyl ether
DTXSID2024246	Pentabromodiphenyl ether	pentabromodiphenyl ether
DTXSID2024246	Pentabromodiphenyl ether	pentabromodiphenyl ether
DTXSID2024246	Pentabromodiphenyl ether	pentabromodiphenyl ether
DTXSID2024246	Pentabromodiphenyl ether	pentabromodiphenyl ether
DTXSID2024246	Pentabromodiphenyl ether	pentabromodiphenyl ether
DTXSID2024246	Pentabromodiphenyl ether	pentabromodiphenyl ether
DTXSID2024246	Pentabromodiphenyl ether	pentabromodiphenyl ether
DTXSID2024246	Pentabromodiphenyl ether	pentabromodiphenyl ether
DTXSID2024246	Pentabromodiphenyl ether	pentabromodiphenyl ether
DTXSID2024246	Pentabromodiphenyl ether	pentabromodiphenyl ether

DTXSID2024246	Pentabromodiphenyl ether	pentabromodiphenyl ether
DTXSID2024246	Pentabromodiphenyl ether	pentabromodiphenyl ether
DTXSID2024246	Pentabromodiphenyl ether	pentabromodiphenyl ether
DTXSID2024246	Pentabromodiphenyl ether	pentabromodiphenyl ether
DTXSID2024246	Pentabromodiphenyl ether	pentabromodiphenyl ether
DTXSID2024246	Pentabromodiphenyl ether	pentabromodiphenyl ether
DTXSID2024246	Pentabromodiphenyl ether	pentabromodiphenyl ether
DTXSID2024246	Pentabromodiphenyl ether	pentabromodiphenyl ether
DTXSID2024246	Pentabromodiphenyl ether	pentabromodiphenyl ether
DTXSID2024246	Pentabromodiphenyl ether	pentabromodiphenyl ether
DTXSID2024246	Pentabromodiphenyl ether	pentabromodiphenyl ether
DTXSID2024246	Pentabromodiphenyl ether	pentabromodiphenyl ether
DTXSID2024246	Pentabromodiphenyl ether	pentabromodiphenyl ether
DTXSID2024246	Pentabromodiphenyl ether	pentabromodiphenyl ether
DTXSID2024246	Pentabromodiphenyl ether	pentabromodiphenyl ether
DTXSID2024246	Pentabromodiphenyl ether	pentabromodiphenyl ether
DTXSID2024246	Pentabromodiphenyl ether	pentabromodiphenyl ether
DTXSID2024246	Pentabromodiphenyl ether	pentabromodiphenyl ether
DTXSID2024246	Pentabromodiphenyl ether	pentabromodiphenyl ether
DTXSID2024246	Pentabromodiphenyl ether	pentabromodiphenyl ether
DTXSID2024246	Pentabromodiphenyl ether	pentabromodiphenyl ether
DTXSID2024246	Pentabromodiphenyl ether	pentabromodiphenyl ether
DTXSID2024246	Pentabromodiphenyl ether	pentabromodiphenyl ether
DTXSID2024246	Pentabromodiphenyl ether	pentabromodiphenyl ether
DTXSID2024246	Pentabromodiphenyl ether	pentabromodiphenyl ether
DTXSID2024246	Pentabromodiphenyl ether	pentabromodiphenyl ether
DTXSID2024246	Pentabromodiphenyl ether	pentabromodiphenyl ether
DTXSID2024246	Pentabromodiphenyl ether	pentabromodiphenyl ether
DTXSID2024246	Pentabromodiphenyl ether	pentabromodiphenyl ether
DTXSID2024246	Pentabromodiphenyl ether	pentabromodiphenyl ether
DTXSID2024246	Pentabromodiphenyl ether	pentabromodiphenyl ether
DTXSID2024246	Pentabromodiphenyl ether	pentabromodiphenyl ether
DTXSID2024246	Pentabromodiphenyl ether	pentabromodiphenyl ether
DTXSID2024246	Pentabromodiphenyl ether	pentabromodiphenyl ether
DTXSID2024246	Pentabromodiphenyl ether	pentabromodiphenyl ether
DTXSID2024246	Pentabromodiphenyl ether	pentabromodiphenyl ether
DTXSID2024246	Pentabromodiphenyl ether	pentabromodiphenyl ether
DTXSID2024246	Pentabromodiphenyl ether	pentabromodiphenyl ether
DTXSID2024246	Pentabromodiphenyl ether	pentabromodiphenyl ether
DTXSID2024246	Pentabromodiphenyl ether	pentabromodiphenyl ether
DTXSID2024246	Pentabromodiphenyl ether	pentabromodiphenyl ether
DTXSID2024246	Pentabromodiphenyl ether	pentabromodiphenyl ether
DTXSID2024246	Pentabromodiphenyl ether	pentabromodiphenyl ether
DTXSID2024246	Pentabromodiphenyl ether	pentabromodiphenyl ether
DTXSID2024246	Pentabromodiphenyl ether	pentabromodiphenyl ether
DTXSID2024246	Pentabromodiphenyl ether	pentabromodiphenyl ether
DTXSID2024246	Pentabromodiphenyl ether	pentabromodiphenyl ether

DTXSID2024246	Pentabromodiphenyl ether	pentabromodiphenyl ether
DTXSID2024246	Pentabromodiphenyl ether	pentabromodiphenyl ether
DTXSID2024246	Pentabromodiphenyl ether	pentabromodiphenyl ether
DTXSID2024246	Pentabromodiphenyl ether	pentabromodiphenyl ether
DTXSID2024246	Pentabromodiphenyl ether	pentabromodiphenyl ether
DTXSID2024246	Pentabromodiphenyl ether	pentabromodiphenyl ether
DTXSID2024246	Pentabromodiphenyl ether	pentabromodiphenyl ether
DTXSID2024246	Pentabromodiphenyl ether	pentabromodiphenyl ether
DTXSID2024246	Pentabromodiphenyl ether	pentabromodiphenyl ether
DTXSID2024246	Pentabromodiphenyl ether	pentabromodiphenyl ether
DTXSID2024246	Pentabromodiphenyl ether	pentabromodiphenyl ether
DTXSID2024246	Pentabromodiphenyl ether	pentabromodiphenyl ether
DTXSID2024246	Pentabromodiphenyl ether	pentabromodiphenyl ether
DTXSID2024246	Pentabromodiphenyl ether	pentabromodiphenyl ether
DTXSID2026943	Pentaerythritol	pentaerythritol
DTXSID2026943	Pentaerythritol	pentaerythritol
DTXSID2027545	Chlordene	chlordene
DTXSID2029242	Diethyl (N,N-bis(2-hydroxyethyl)amino	Fyrol 6
DTXSID2032180	2,2',4,4',5,5'-Hexachlorobiphenyl	2,4,5,2',4',5'-hexachlorobiphenyl
DTXSID2032180	2,2',4,4',5,5'-Hexachlorobiphenyl	2,4,5,2',4',5'-hexachlorobiphenyl
DTXSID2032180	2,2',4,4',5,5'-Hexachlorobiphenyl	2,4,5,2',4',5'-hexachlorobiphenyl
DTXSID2036405	Aluminum hydroxide	Aluminum Hydroxide
DTXSID2049634	Graphite	Graphite
DTXSID2049634	Graphite	Graphite
DTXSID2049634	Graphite	Graphite
DTXSID2049634	Graphite	Graphite
DTXSID2049634	Graphite	Graphite
DTXSID2062325	Trifluoroiodomethane	trifluoroiodomethane
DTXSID2062325	Trifluoroiodomethane	trifluoroiodomethane
DTXSID20879972	BDE-175	2,2',4,4',5,6'-hexabromodiphenyl ether
DTXSID20879972	BDE-175	2,2',4,4',5,6'-hexabromodiphenyl ether
DTXSID3020174	Bis(2,3-dibromopropyl) phosphate, ma	bis(2,3-dibromopropyl)phosphate
DTXSID3020174	Bis(2,3-dibromopropyl) phosphate, ma	bis(2,3-dibromopropyl)phosphate
DTXSID3020174	Bis(2,3-dibromopropyl) phosphate, ma	bis(2,3-dibromopropyl)phosphate
DTXSID3020205	Benzyl butyl phthalate	butylbenzyl phthalate
DTXSID3020679	Heptachlor	Heptachlor
DTXSID3021770	2,2',6,6'-Tetrachlorobisphenol A	tetrachlorodian
DTXSID3021770	2,2',6,6'-Tetrachlorobisphenol A	tetrachlorodian
DTXSID3021770	2,2',6,6'-Tetrachlorobisphenol A	tetrachlorodian
DTXSID3021770	2,2',6,6'-Tetrachlorobisphenol A	tetrachlorodian
DTXSID3021770	2,2',6,6'-Tetrachlorobisphenol A	tetrachlorodian
DTXSID3021770	2,2',6,6'-Tetrachlorobisphenol A	tetrachlorodian
DTXSID3021770	2,2',6,6'-Tetrachlorobisphenol A	tetrachlorodian
DTXSID3021770	2,2',6,6'-Tetrachlorobisphenol A	tetrachlorodian
DTXSID3021770	2,2',6,6'-Tetrachlorobisphenol A	tetrachlorodian
DTXSID3021770	2,2',6,6'-Tetrachlorobisphenol A	tetrachlorodian
DTXSID3021770	2,2',6,6'-Tetrachlorobisphenol A	tetrachlorodian

DTXSID3021770	2,2',6,6'-Tetrachlorobisphenol A	tetrachlorodian
DTXSID3021770	2,2',6,6'-Tetrachlorobisphenol A	tetrachlorodian
DTXSID3021986	Tributyl phosphate	tributyl phosphate
DTXSID3021986	Tributyl phosphate	tributyl phosphate
DTXSID3021986	Tributyl phosphate	tributyl phosphate
DTXSID3021986	Tributyl phosphate	tributyl phosphate
DTXSID3021986	Tributyl phosphate	tributyl phosphate
DTXSID3021986	Tributyl phosphate	tributyl phosphate
DTXSID3021986	Tributyl phosphate	tributyl phosphate
DTXSID3021986	Tributyl phosphate	tributyl phosphate
DTXSID3021986	Tributyl phosphate	tributyl phosphate
DTXSID3021986	Tributyl phosphate	tributyl phosphate
DTXSID3021986	Tributyl phosphate	tributyl phosphate
DTXSID3021986	Tributyl phosphate	tributyl phosphate
DTXSID3021986	Tributyl phosphate	tributyl phosphate
DTXSID3021986	Tributyl phosphate	tributyl phosphate
DTXSID3025833	Pentabromotoluene	2,3,4,5,6-pentabromotoluene
DTXSID3025833	Pentabromotoluene	2,3,4,5,6-pentabromotoluene
DTXSID3025833	Pentabromotoluene	2,3,4,5,6-pentabromotoluene
DTXSID3027615	Tris(2-chloropropyl) phosphate	Fyrol PCF
DTXSID3027615	Tris(2-chloropropyl) phosphate	Fyrol PCF
DTXSID3027615	Tris(2-chloropropyl) phosphate	Fyrol PCF
DTXSID3027615	Tris(2-chloropropyl) phosphate	Fyrol PCF
DTXSID3027615	Tris(2-chloropropyl) phosphate	Fyrol PCF
DTXSID3027615	Tris(2-chloropropyl) phosphate	Fyrol PCF
DTXSID3027615	Tris(2-chloropropyl) phosphate	Fyrol PCF
DTXSID3027615	Tris(2-chloropropyl) phosphate	Fyrol PCF
DTXSID3030056	2,2',4,4'-Tetrabromodiphenyl ether	2,2',4,4'-tetrabromodiphenyl ether
DTXSID3030056	2,2',4,4'-Tetrabromodiphenyl ether	2,2',4,4'-tetrabromodiphenyl ether
DTXSID3030056	2,2',4,4'-Tetrabromodiphenyl ether	2,2',4,4'-tetrabromodiphenyl ether
DTXSID3030056	2,2',4,4'-Tetrabromodiphenyl ether	2,2',4,4'-tetrabromodiphenyl ether
DTXSID3030056	2,2',4,4'-Tetrabromodiphenyl ether	2,2',4,4'-tetrabromodiphenyl ether
DTXSID3030056	2,2',4,4'-Tetrabromodiphenyl ether	2,2',4,4'-tetrabromodiphenyl ether
DTXSID3030056	2,2',4,4'-Tetrabromodiphenyl ether	2,2',4,4'-tetrabromodiphenyl ether
DTXSID3030056	2,2',4,4'-Tetrabromodiphenyl ether	2,2',4,4'-tetrabromodiphenyl ether
DTXSID3030056	2,2',4,4'-Tetrabromodiphenyl ether	2,2',4,4'-tetrabromodiphenyl ether
DTXSID3030056	2,2',4,4'-Tetrabromodiphenyl ether	2,2',4,4'-tetrabromodiphenyl ether
DTXSID3030056	2,2',4,4'-Tetrabromodiphenyl ether	2,2',4,4'-tetrabromodiphenyl ether
DTXSID3030056	2,2',4,4'-Tetrabromodiphenyl ether	2,2',4,4'-tetrabromodiphenyl ether
DTXSID3030056	2,2',4,4'-Tetrabromodiphenyl ether	2,2',4,4'-tetrabromodiphenyl ether
DTXSID3030056	2,2',4,4'-Tetrabromodiphenyl ether	2,2',4,4'-tetrabromodiphenyl ether
DTXSID3030056	2,2',4,4'-Tetrabromodiphenyl ether	2,2',4,4'-tetrabromodiphenyl ether
DTXSID3030056	2,2',4,4'-Tetrabromodiphenyl ether	2,2',4,4'-tetrabromodiphenyl ether
DTXSID3030056	2,2',4,4'-Tetrabromodiphenyl ether	2,2',4,4'-tetrabromodiphenyl ether
DTXSID3030056	2,2',4,4'-Tetrabromodiphenyl ether	2,2',4,4'-tetrabromodiphenyl ether
DTXSID3030056	2,2',4,4'-Tetrabromodiphenyl ether	2,2',4,4'-tetrabromodiphenyl ether

DTXSID3030056	2,2',4,4'-Tetrabromodiphenyl ether	2,2',4,4'-tetrabromodiphenyl ether
DTXSID3030056	2,2',4,4'-Tetrabromodiphenyl ether	2,2',4,4'-tetrabromodiphenyl ether
DTXSID3030056	2,2',4,4'-Tetrabromodiphenyl ether	2,2',4,4'-tetrabromodiphenyl ether
DTXSID3030056	2,2',4,4'-Tetrabromodiphenyl ether	2,2',4,4'-tetrabromodiphenyl ether
DTXSID3030056	2,2',4,4'-Tetrabromodiphenyl ether	2,2',4,4'-tetrabromodiphenyl ether
DTXSID3030056	2,2',4,4'-Tetrabromodiphenyl ether	2,2',4,4'-tetrabromodiphenyl ether
DTXSID3030056	2,2',4,4'-Tetrabromodiphenyl ether	2,2',4,4'-tetrabromodiphenyl ether
DTXSID3030056	2,2',4,4'-Tetrabromodiphenyl ether	2,2',4,4'-tetrabromodiphenyl ether
DTXSID3030056	2,2',4,4'-Tetrabromodiphenyl ether	2,2',4,4'-tetrabromodiphenyl ether
DTXSID3030056	2,2',4,4'-Tetrabromodiphenyl ether	2,2',4,4'-tetrabromodiphenyl ether
DTXSID3030056	2,2',4,4'-Tetrabromodiphenyl ether	2,2',4,4'-tetrabromodiphenyl ether
DTXSID3030056	2,2',4,4'-Tetrabromodiphenyl ether	2,2',4,4'-tetrabromodiphenyl ether
DTXSID3030056	2,2',4,4'-Tetrabromodiphenyl ether	2,2',4,4'-tetrabromodiphenyl ether
DTXSID3030056	2,2',4,4'-Tetrabromodiphenyl ether	2,2',4,4'-tetrabromodiphenyl ether
DTXSID3030056	2,2',4,4'-Tetrabromodiphenyl ether	2,2',4,4'-tetrabromodiphenyl ether
DTXSID3030056	2,2',4,4'-Tetrabromodiphenyl ether	2,2',4,4'-tetrabromodiphenyl ether
DTXSID3030056	2,2',4,4'-Tetrabromodiphenyl ether	2,2',4,4'-tetrabromodiphenyl ether
DTXSID3030056	2,2',4,4'-Tetrabromodiphenyl ether	2,2',4,4'-tetrabromodiphenyl ether
DTXSID3030056	2,2',4,4'-Tetrabromodiphenyl ether	2,2',4,4'-tetrabromodiphenyl ether
DTXSID3030056	2,2',4,4'-Tetrabromodiphenyl ether	2,2',4,4'-tetrabromodiphenyl ether
DTXSID3030056	2,2',4,4'-Tetrabromodiphenyl ether	2,2',4,4'-tetrabromodiphenyl ether
DTXSID3030056	2,2',4,4'-Tetrabromodiphenyl ether	2,2',4,4'-tetrabromodiphenyl ether
DTXSID3030056	2,2',4,4'-Tetrabromodiphenyl ether	2,2',4,4'-tetrabromodiphenyl ether
DTXSID3030056	2,2',4,4'-Tetrabromodiphenyl ether	2,2',4,4'-tetrabromodiphenyl ether
DTXSID3030056	2,2',4,4'-Tetrabromodiphenyl ether	2,2',4,4'-tetrabromodiphenyl ether
DTXSID3030056	2,2',4,4'-Tetrabromodiphenyl ether	2,2',4,4'-tetrabromodiphenyl ether
DTXSID3030056	2,2',4,4'-Tetrabromodiphenyl ether	2,2',4,4'-tetrabromodiphenyl ether
DTXSID3030056	2,2',4,4'-Tetrabromodiphenyl ether	2,2',4,4'-tetrabromodiphenyl ether
DTXSID3030056	2,2',4,4'-Tetrabromodiphenyl ether	2,2',4,4'-tetrabromodiphenyl ether
DTXSID3030056	2,2',4,4'-Tetrabromodiphenyl ether	2,2',4,4'-tetrabromodiphenyl ether
DTXSID3030056	2,2',4,4'-Tetrabromodiphenyl ether	2,2',4,4'-tetrabromodiphenyl ether
DTXSID3030056	2,2',4,4'-Tetrabromodiphenyl ether	2,2',4,4'-tetrabromodiphenyl ether
DTXSID3030056	2,2',4,4'-Tetrabromodiphenyl ether	2,2',4,4'-tetrabromodiphenyl ether
DTXSID3030056	2,2',4,4'-Tetrabromodiphenyl ether	2,2',4,4'-tetrabromodiphenyl ether
DTXSID3030056	2,2',4,4'-Tetrabromodiphenyl ether	2,2',4,4'-tetrabromodiphenyl ether
DTXSID3030056	2,2',4,4'-Tetrabromodiphenyl ether	2,2',4,4'-tetrabromodiphenyl ether
DTXSID3030056	2,2',4,4'-Tetrabromodiphenyl ether	2,2',4,4'-tetrabromodiphenyl ether
DTXSID3030056	2,2',4,4'-Tetrabromodiphenyl ether	2,2',4,4'-tetrabromodiphenyl ether
DTXSID3030056	2,2',4,4'-Tetrabromodiphenyl ether	2,2',4,4'-tetrabromodiphenyl ether
DTXSID3030056	2,2',4,4'-Tetrabromodiphenyl ether	2,2',4,4'-tetrabromodiphenyl ether
DTXSID3030056	2,2',4,4'-Tetrabromodiphenyl ether	2,2',4,4'-tetrabromodiphenyl ether
DTXSID3030056	2,2',4,4'-Tetrabromodiphenyl ether	2,2',4,4'-tetrabromodiphenyl ether
DTXSID3030056	2,2',4,4'-Tetrabromodiphenyl ether	2,2',4,4'-tetrabromodiphenyl ether
DTXSID3030056	2,2',4,4'-Tetrabromodiphenyl ether	2,2',4,4'-tetrabromodiphenyl ether
DTXSID3030056	2,2',4,4'-Tetrabromodiphenyl ether	2,2',4,4'-tetrabromodiphenyl ether
DTXSID3030056	2,2',4,4'-Tetrabromodiphenyl ether	2,2',4,4'-tetrabromodiphenyl ether
DTXSID3030056	2,2',4,4'-Tetrabromodiphenyl ether	2,2',4,4'-tetrabromodiphenyl ether

DTXSID3030056	2,2',4,4'-Tetrabromodiphenyl ether	2,2',4,4'-tetrabromodiphenyl ether
DTXSID3030056	2,2',4,4'-Tetrabromodiphenyl ether	2,2',4,4'-tetrabromodiphenyl ether
DTXSID3030056	2,2',4,4'-Tetrabromodiphenyl ether	2,2',4,4'-tetrabromodiphenyl ether
DTXSID3030056	2,2',4,4'-Tetrabromodiphenyl ether	2,2',4,4'-tetrabromodiphenyl ether
DTXSID3030056	2,2',4,4'-Tetrabromodiphenyl ether	2,2',4,4'-tetrabromodiphenyl ether
DTXSID3030056	2,2',4,4'-Tetrabromodiphenyl ether	2,2',4,4'-tetrabromodiphenyl ether
DTXSID3030056	2,2',4,4'-Tetrabromodiphenyl ether	2,2',4,4'-tetrabromodiphenyl ether
DTXSID3030056	2,2',4,4'-Tetrabromodiphenyl ether	2,2',4,4'-tetrabromodiphenyl ether
DTXSID3030056	2,2',4,4'-Tetrabromodiphenyl ether	2,2',4,4'-tetrabromodiphenyl ether
DTXSID3030056	2,2',4,4'-Tetrabromodiphenyl ether	2,2',4,4'-tetrabromodiphenyl ether
DTXSID3030056	2,2',4,4'-Tetrabromodiphenyl ether	2,2',4,4'-tetrabromodiphenyl ether
DTXSID3030056	2,2',4,4'-Tetrabromodiphenyl ether	2,2',4,4'-tetrabromodiphenyl ether
DTXSID3030056	2,2',4,4'-Tetrabromodiphenyl ether	2,2',4,4'-tetrabromodiphenyl ether
DTXSID3030056	2,2',4,4'-Tetrabromodiphenyl ether	2,2',4,4'-tetrabromodiphenyl ether
DTXSID3030056	2,2',4,4'-Tetrabromodiphenyl ether	2,2',4,4'-tetrabromodiphenyl ether
DTXSID3030056	2,2',4,4'-Tetrabromodiphenyl ether	2,2',4,4'-tetrabromodiphenyl ether
DTXSID3030056	2,2',4,4'-Tetrabromodiphenyl ether	2,2',4,4'-tetrabromodiphenyl ether
DTXSID3030056	2,2',4,4'-Tetrabromodiphenyl ether	2,2',4,4'-tetrabromodiphenyl ether
DTXSID3030056	2,2',4,4'-Tetrabromodiphenyl ether	2,2',4,4'-tetrabromodiphenyl ether
DTXSID3030056	2,2',4,4'-Tetrabromodiphenyl ether	2,2',4,4'-tetrabromodiphenyl ether
DTXSID3030056	2,2',4,4'-Tetrabromodiphenyl ether	2,2',4,4'-tetrabromodiphenyl ether
DTXSID3030056	2,2',4,4'-Tetrabromodiphenyl ether	2,2',4,4'-tetrabromodiphenyl ether
DTXSID3030056	2,2',4,4'-Tetrabromodiphenyl ether	2,2',4,4'-tetrabromodiphenyl ether
DTXSID3030056	2,2',4,4'-Tetrabromodiphenyl ether	2,2',4,4'-tetrabromodiphenyl ether
DTXSID3030056	2,2',4,4'-Tetrabromodiphenyl ether	2,2',4,4'-tetrabromodiphenyl ether
DTXSID3030056	2,2',4,4'-Tetrabromodiphenyl ether	2,2',4,4'-tetrabromodiphenyl ether
DTXSID3030056	2,2',4,4'-Tetrabromodiphenyl ether	2,2',4,4'-tetrabromodiphenyl ether
DTXSID3030056	2,2',4,4'-Tetrabromodiphenyl ether	2,2',4,4'-tetrabromodiphenyl ether
DTXSID3030056	2,2',4,4'-Tetrabromodiphenyl ether	2,2',4,4'-tetrabromodiphenyl ether
DTXSID3030056	2,2',4,4'-Tetrabromodiphenyl ether	2,2',4,4'-tetrabromodiphenyl ether
DTXSID3030056	2,2',4,4'-Tetrabromodiphenyl ether	2,2',4,4'-tetrabromodiphenyl ether
DTXSID3030056	2,2',4,4'-Tetrabromodiphenyl ether	2,2',4,4'-tetrabromodiphenyl ether
DTXSID3030056	2,2',4,4'-Tetrabromodiphenyl ether	2,2',4,4'-tetrabromodiphenyl ether
DTXSID3030056	2,2',4,4'-Tetrabromodiphenyl ether	2,2',4,4'-tetrabromodiphenyl ether
DTXSID3030056	2,2',4,4'-Tetrabromodiphenyl ether	2,2',4,4'-tetrabromodiphenyl ether
DTXSID3030056	2,2',4,4'-Tetrabromodiphenyl ether	2,2',4,4'-tetrabromodiphenyl ether
DTXSID3030056	2,2',4,4'-Tetrabromodiphenyl ether	2,2',4,4'-tetrabromodiphenyl ether
DTXSID3030056	2,2',4,4'-Tetrabromodiphenyl ether	2,2',4,4'-tetrabromodiphenyl ether
DTXSID3030056	2,2',4,4'-Tetrabromodiphenyl ether	2,2',4,4'-tetrabromodiphenyl ether
DTXSID3030056	2,2',4,4'-Tetrabromodiphenyl ether	2,2',4,4'-tetrabromodiphenyl ether
DTXSID3030056	2,2',4,4'-Tetrabromodiphenyl ether	2,2',4,4'-tetrabromodiphenyl ether
DTXSID3030056	2,2',4,4'-Tetrabromodiphenyl ether	2,2',4,4'-tetrabromodiphenyl ether
DTXSID3030056	2,2',4,4'-Tetrabromodiphenyl ether	2,2',4,4'-tetrabromodiphenyl ether
DTXSID3030056	2,2',4,4'-Tetrabromodiphenyl ether	2,2',4,4'-tetrabromodiphenyl ether
DTXSID3030056	2,2',4,4'-Tetrabromodiphenyl ether	2,2',4,4'-tetrabromodiphenyl ether
DTXSID3030056	2,2',4,4'-Tetrabromodiphenyl ether	2,2',4,4'-tetrabromodiphenyl ether
DTXSID3030056	2,2',4,4'-Tetrabromodiphenyl ether	2,2',4,4'-tetrabromodiphenyl ether

DTXSID3030056	2,2',4,4'-Tetrabromodiphenyl ether	2,2',4,4'-tetrabromodiphenyl ether
DTXSID3030056	2,2',4,4'-Tetrabromodiphenyl ether	2,2',4,4'-tetrabromodiphenyl ether
DTXSID3030056	2,2',4,4'-Tetrabromodiphenyl ether	2,2',4,4'-tetrabromodiphenyl ether
DTXSID3030056	2,2',4,4'-Tetrabromodiphenyl ether	2,2',4,4'-tetrabromodiphenyl ether
DTXSID3030056	2,2',4,4'-Tetrabromodiphenyl ether	2,2',4,4'-tetrabromodiphenyl ether
DTXSID3030056	2,2',4,4'-Tetrabromodiphenyl ether	2,2',4,4'-tetrabromodiphenyl ether
DTXSID3030056	2,2',4,4'-Tetrabromodiphenyl ether	2,2',4,4'-tetrabromodiphenyl ether
DTXSID3030056	2,2',4,4'-Tetrabromodiphenyl ether	2,2',4,4'-tetrabromodiphenyl ether
DTXSID3030056	2,2',4,4'-Tetrabromodiphenyl ether	2,2',4,4'-tetrabromodiphenyl ether
DTXSID3030056	2,2',4,4'-Tetrabromodiphenyl ether	2,2',4,4'-tetrabromodiphenyl ether
DTXSID3030056	2,2',4,4'-Tetrabromodiphenyl ether	2,2',4,4'-tetrabromodiphenyl ether
DTXSID3030056	2,2',4,4'-Tetrabromodiphenyl ether	2,2',4,4'-tetrabromodiphenyl ether
DTXSID3030056	2,2',4,4'-Tetrabromodiphenyl ether	2,2',4,4'-tetrabromodiphenyl ether
DTXSID30451985	BDE-207	2,2',3,3',4,4',5,6,6'-nonabromodiphenyl ether
DTXSID30451985	BDE-207	2,2',3,3',4,4',5,6,6'-nonabromodiphenyl ether
DTXSID3051543	4-Bromophenol	4-bromophenol
DTXSID3052692	2,2',4,4',5,6'-Hexabromodiphenyl ethe	2,2',4,4',5,6'-hexabromodiphenyl ether
DTXSID3052692	2,2',4,4',5,6'-Hexabromodiphenyl ethe	2,2',4,4',5,6'-hexabromodiphenyl ether
DTXSID3060950	1,3,5-Triazin-2(1H)-one, 4,6-diamino-	ammeline
DTXSID3060950	1,3,5-Triazin-2(1H)-one, 4,6-diamino-	ammeline
DTXSID3068043	1,3,5-Triazine-2,4,6(1H,3H,5H)-trione,	melamine cyanurate
DTXSID30958080	Ethenyl dihydrogen phosphate	vinyl phosphate
DTXSID40160258	Zirconium phosphate	zirconium phosphate
DTXSID4023880	Antimony trioxide	antimony trioxide
DTXSID4023880	Antimony trioxide	antimony trioxide
DTXSID4023888	Asbestos	Asbestos
DTXSID4023888	Asbestos	Asbestos
DTXSID4027527	1,2,5,6,9,10-Hexabromocyclododecan	hexabromocyclododecane
DTXSID4027527	1,2,5,6,9,10-Hexabromocyclododecan	hexabromocyclododecane
DTXSID4027527	1,2,5,6,9,10-Hexabromocyclododecan	hexabromocyclododecane
DTXSID4027527	1,2,5,6,9,10-Hexabromocyclododecan	hexabromocyclododecane
DTXSID4027527	1,2,5,6,9,10-Hexabromocyclododecan	hexabromocyclododecane
DTXSID4027527	1,2,5,6,9,10-Hexabromocyclododecan	hexabromocyclododecane
DTXSID4027527	1,2,5,6,9,10-Hexabromocyclododecan	hexabromocyclododecane
DTXSID4027527	1,2,5,6,9,10-Hexabromocyclododecan	hexabromocyclododecane
DTXSID4027527	1,2,5,6,9,10-Hexabromocyclododecan	hexabromocyclododecane
DTXSID4027527	1,2,5,6,9,10-Hexabromocyclododecan	hexabromocyclododecane
DTXSID4027527	1,2,5,6,9,10-Hexabromocyclododecan	hexabromocyclododecane
DTXSID4027527	1,2,5,6,9,10-Hexabromocyclododecan	hexabromocyclododecane
DTXSID4027527	1,2,5,6,9,10-Hexabromocyclododecan	hexabromocyclododecane
DTXSID4027527	1,2,5,6,9,10-Hexabromocyclododecan	hexabromocyclododecane
DTXSID4027527	1,2,5,6,9,10-Hexabromocyclododecan	hexabromocyclododecane
DTXSID4027527	1,2,5,6,9,10-Hexabromocyclododecan	hexabromocyclododecane
DTXSID4027527	1,2,5,6,9,10-Hexabromocyclododecan	hexabromocyclododecane
DTXSID4027527	1,2,5,6,9,10-Hexabromocyclododecan	hexabromocyclododecane
DTXSID4027527	1,2,5,6,9,10-Hexabromocyclododecan	hexabromocyclododecane
DTXSID4027527	1,2,5,6,9,10-Hexabromocyclododecan	hexabromocyclododecane

DTXSID4027527	1,2,5,6,9,10-Hexabromocyclododecane	hexabromocyclododecane
DTXSID4027527	1,2,5,6,9,10-Hexabromocyclododecane	hexabromocyclododecane
DTXSID4027527	1,2,5,6,9,10-Hexabromocyclododecane	hexabromocyclododecane
DTXSID4027527	1,2,5,6,9,10-Hexabromocyclododecane	hexabromocyclododecane
DTXSID4027527	1,2,5,6,9,10-Hexabromocyclododecane	hexabromocyclododecane
DTXSID4027527	1,2,5,6,9,10-Hexabromocyclododecane	hexabromocyclododecane
DTXSID4027527	1,2,5,6,9,10-Hexabromocyclododecane	hexabromocyclododecane
DTXSID4027527	1,2,5,6,9,10-Hexabromocyclododecane	hexabromocyclododecane
DTXSID4027527	1,2,5,6,9,10-Hexabromocyclododecane	hexabromocyclododecane
DTXSID4027527	1,2,5,6,9,10-Hexabromocyclododecane	hexabromocyclododecane
DTXSID4027527	1,2,5,6,9,10-Hexabromocyclododecane	hexabromocyclododecane
DTXSID4027527	1,2,5,6,9,10-Hexabromocyclododecane	hexabromocyclododecane
DTXSID4027527	1,2,5,6,9,10-Hexabromocyclododecane	hexabromocyclododecane
DTXSID4027527	1,2,5,6,9,10-Hexabromocyclododecane	hexabromocyclododecane
DTXSID4027527	1,2,5,6,9,10-Hexabromocyclododecane	hexabromocyclododecane
DTXSID4027527	1,2,5,6,9,10-Hexabromocyclododecane	hexabromocyclododecane
DTXSID4027527	1,2,5,6,9,10-Hexabromocyclododecane	hexabromocyclododecane
DTXSID4027527	1,2,5,6,9,10-Hexabromocyclododecane	hexabromocyclododecane
DTXSID4027527	1,2,5,6,9,10-Hexabromocyclododecane	hexabromocyclododecane
DTXSID4027527	1,2,5,6,9,10-Hexabromocyclododecane	hexabromocyclododecane
DTXSID4027527	1,2,5,6,9,10-Hexabromocyclododecane	hexabromocyclododecane
DTXSID4027527	1,2,5,6,9,10-Hexabromocyclododecane	hexabromocyclododecane
DTXSID4027527	1,2,5,6,9,10-Hexabromocyclododecane	hexabromocyclododecane
DTXSID4027527	1,2,5,6,9,10-Hexabromocyclododecane	hexabromocyclododecane
DTXSID4027527	1,2,5,6,9,10-Hexabromocyclododecane	hexabromocyclododecane
DTXSID4027527	1,2,5,6,9,10-Hexabromocyclododecane	hexabromocyclododecane
DTXSID4027527	1,2,5,6,9,10-Hexabromocyclododecane	hexabromocyclododecane
DTXSID4027527	1,2,5,6,9,10-Hexabromocyclododecane	hexabromocyclododecane
DTXSID4027527	1,2,5,6,9,10-Hexabromocyclododecane	hexabromocyclododecane
DTXSID4027527	1,2,5,6,9,10-Hexabromocyclododecane	hexabromocyclododecane
DTXSID4027527	1,2,5,6,9,10-Hexabromocyclododecane	hexabromocyclododecane
DTXSID4027527	1,2,5,6,9,10-Hexabromocyclododecane	hexabromocyclododecane
DTXSID4027527	1,2,5,6,9,10-Hexabromocyclododecane	hexabromocyclododecane
DTXSID4027527	1,2,5,6,9,10-Hexabromocyclododecane	hexabromocyclododecane
DTXSID4027527	1,2,5,6,9,10-Hexabromocyclododecane	hexabromocyclododecane
DTXSID4027527	1,2,5,6,9,10-Hexabromocyclododecane	hexabromocyclododecane
DTXSID4027527	1,2,5,6,9,10-Hexabromocyclododecane	hexabromocyclododecane
DTXSID4027527	1,2,5,6,9,10-Hexabromocyclododecane	hexabromocyclododecane
DTXSID4027527	1,2,5,6,9,10-Hexabromocyclododecane	hexabromocyclododecane
DTXSID4027527	1,2,5,6,9,10-Hexabromocyclododecane	hexabromocyclododecane
DTXSID4027527	1,2,5,6,9,10-Hexabromocyclododecane	hexabromocyclododecane
DTXSID4027527	1,2,5,6,9,10-Hexabromocyclododecane	hexabromocyclododecane
DTXSID4027527	1,2,5,6,9,10-Hexabromocyclododecane	hexabromocyclododecane
DTXSID4027527	1,2,5,6,9,10-Hexabromocyclododecane	hexabromocyclododecane
DTXSID4027527	1,2,5,6,9,10-Hexabromocyclododecane	hexabromocyclododecane
DTXSID4027527	1,2,5,6,9,10-Hexabromocyclododecane	hexabromocyclododecane
DTXSID4027527	1,2,5,6,9,10-Hexabromocyclododecane	hexabromocyclododecane

DTXSID4027527	1,2,5,6,9,10-Hexabromocyclododecan	hexabromocyclododecane
DTXSID4027527	1,2,5,6,9,10-Hexabromocyclododecan	hexabromocyclododecane
DTXSID4027527	1,2,5,6,9,10-Hexabromocyclododecan	hexabromocyclododecane
DTXSID4027527	1,2,5,6,9,10-Hexabromocyclododecan	hexabromocyclododecane
DTXSID4027527	1,2,5,6,9,10-Hexabromocyclododecan	hexabromocyclododecane
DTXSID4027527	1,2,5,6,9,10-Hexabromocyclododecan	hexabromocyclododecane
DTXSID4027527	1,2,5,6,9,10-Hexabromocyclododecan	hexabromocyclododecane
DTXSID4027527	1,2,5,6,9,10-Hexabromocyclododecan	hexabromocyclododecane
DTXSID4027527	1,2,5,6,9,10-Hexabromocyclododecan	hexabromocyclododecane
DTXSID4027527	1,2,5,6,9,10-Hexabromocyclododecan	hexabromocyclododecane
DTXSID4027527	1,2,5,6,9,10-Hexabromocyclododecan	hexabromocyclododecane
DTXSID4027527	1,2,5,6,9,10-Hexabromocyclododecan	hexabromocyclododecane
DTXSID4027527	1,2,5,6,9,10-Hexabromocyclododecan	hexabromocyclododecane
DTXSID4027527	1,2,5,6,9,10-Hexabromocyclododecan	hexabromocyclododecane
DTXSID4027527	1,2,5,6,9,10-Hexabromocyclododecan	hexabromocyclododecane
DTXSID4027527	1,2,5,6,9,10-Hexabromocyclododecan	hexabromocyclododecane
DTXSID4027527	1,2,5,6,9,10-Hexabromocyclododecan	hexabromocyclododecane
DTXSID4027527	1,2,5,6,9,10-Hexabromocyclododecan	hexabromocyclododecane
DTXSID4027527	1,2,5,6,9,10-Hexabromocyclododecan	hexabromocyclododecane
DTXSID4027527	1,2,5,6,9,10-Hexabromocyclododecan	hexabromocyclododecane
DTXSID4027527	1,2,5,6,9,10-Hexabromocyclododecan	hexabromocyclododecane
DTXSID4027527	1,2,5,6,9,10-Hexabromocyclododecan	hexabromocyclododecane
DTXSID4027527	1,2,5,6,9,10-Hexabromocyclododecan	hexabromocyclododecane
DTXSID4027527	1,2,5,6,9,10-Hexabromocyclododecan	hexabromocyclododecane
DTXSID4027527	1,2,5,6,9,10-Hexabromocyclododecan	hexabromocyclododecane
DTXSID4027527	1,2,5,6,9,10-Hexabromocyclododecan	hexabromocyclododecane
DTXSID4027527	1,2,5,6,9,10-Hexabromocyclododecan	hexabromocyclododecane
DTXSID4027527	1,2,5,6,9,10-Hexabromocyclododecan	hexabromocyclododecane
DTXSID4027527	1,2,5,6,9,10-Hexabromocyclododecan	hexabromocyclododecane
DTXSID4027527	1,2,5,6,9,10-Hexabromocyclododecan	hexabromocyclododecane
DTXSID4027527	1,2,5,6,9,10-Hexabromocyclododecan	hexabromocyclododecane
DTXSID4027527	1,2,5,6,9,10-Hexabromocyclododecan	hexabromocyclododecane
DTXSID4027527	1,2,5,6,9,10-Hexabromocyclododecan	hexabromocyclododecane
DTXSID4027527	1,2,5,6,9,10-Hexabromocyclododecan	hexabromocyclododecane
DTXSID4027527	1,2,5,6,9,10-Hexabromocyclododecan	hexabromocyclododecane
DTXSID4027527	1,2,5,6,9,10-Hexabromocyclododecan	hexabromocyclododecane
DTXSID4027527	1,2,5,6,9,10-Hexabromocyclododecan	hexabromocyclododecane
DTXSID4027527	1,2,5,6,9,10-Hexabromocyclododecan	hexabromocyclododecane
DTXSID4027527	1,2,5,6,9,10-Hexabromocyclododecan	hexabromocyclododecane
DTXSID4027527	1,2,5,6,9,10-Hexabromocyclododecan	hexabromocyclododecane
DTXSID4027527	1,2,5,6,9,10-Hexabromocyclododecan	hexabromocyclododecane
DTXSID4027527	1,2,5,6,9,10-Hexabromocyclododecan	hexabromocyclododecane
DTXSID4027527	1,2,5,6,9,10-Hexabromocyclododecan	hexabromocyclododecane
DTXSID4027527	1,2,5,6,9,10-Hexabromocyclododecan	hexabromocyclododecane
DTXSID4027527	1,2,5,6,9,10-Hexabromocyclododecan	hexabromocyclododecane
DTXSID4027527	1,2,5,6,9,10-Hexabromocyclododecan	hexabromocyclododecane
DTXSID4027527	1,2,5,6,9,10-Hexabromocyclododecan	hexabromocyclododecane

DTXSID4027527	1,2,5,6,9,10-Hexabromocyclododecan	hexabromocyclododecane
DTXSID4027527	1,2,5,6,9,10-Hexabromocyclododecan	hexabromocyclododecane
DTXSID4027527	1,2,5,6,9,10-Hexabromocyclododecan	hexabromocyclododecane
DTXSID4027527	1,2,5,6,9,10-Hexabromocyclododecan	hexabromocyclododecane
DTXSID4027527	1,2,5,6,9,10-Hexabromocyclododecan	hexabromocyclododecane
DTXSID4027527	1,2,5,6,9,10-Hexabromocyclododecan	hexabromocyclododecane
DTXSID4027527	1,2,5,6,9,10-Hexabromocyclododecan	hexabromocyclododecane
DTXSID4027527	1,2,5,6,9,10-Hexabromocyclododecan	hexabromocyclododecane
DTXSID4027527	1,2,5,6,9,10-Hexabromocyclododecan	hexabromocyclododecane
DTXSID4027527	1,2,5,6,9,10-Hexabromocyclododecan	hexabromocyclododecane
DTXSID4027527	1,2,5,6,9,10-Hexabromocyclododecan	hexabromocyclododecane
DTXSID4027527	1,2,5,6,9,10-Hexabromocyclododecan	hexabromocyclododecane
DTXSID4027527	1,2,5,6,9,10-Hexabromocyclododecan	hexabromocyclododecane
DTXSID4027527	1,2,5,6,9,10-Hexabromocyclododecan	hexabromocyclododecane
DTXSID4027527	1,2,5,6,9,10-Hexabromocyclododecan	hexabromocyclododecane
DTXSID4027527	1,2,5,6,9,10-Hexabromocyclododecan	hexabromocyclododecane
DTXSID4027527	1,2,5,6,9,10-Hexabromocyclododecan	hexabromocyclododecane
DTXSID4027527	1,2,5,6,9,10-Hexabromocyclododecan	hexabromocyclododecane
DTXSID4027527	1,2,5,6,9,10-Hexabromocyclododecan	hexabromocyclododecane
DTXSID4027527	1,2,5,6,9,10-Hexabromocyclododecan	hexabromocyclododecane
DTXSID4027527	1,2,5,6,9,10-Hexabromocyclododecan	hexabromocyclododecane
DTXSID4027527	1,2,5,6,9,10-Hexabromocyclododecan	hexabromocyclododecane
DTXSID4027527	1,2,5,6,9,10-Hexabromocyclododecan	hexabromocyclododecane
DTXSID4027527	1,2,5,6,9,10-Hexabromocyclododecan	hexabromocyclododecane
DTXSID4027527	1,2,5,6,9,10-Hexabromocyclododecan	hexabromocyclododecane
DTXSID4027527	1,2,5,6,9,10-Hexabromocyclododecan	hexabromocyclododecane
DTXSID4027527	1,2,5,6,9,10-Hexabromocyclododecan	hexabromocyclododecane
DTXSID4027527	1,2,5,6,9,10-Hexabromocyclododecan	hexabromocyclododecane
DTXSID4027527	1,2,5,6,9,10-Hexabromocyclododecan	hexabromocyclododecane
DTXSID4027527	1,2,5,6,9,10-Hexabromocyclododecan	hexabromocyclododecane
DTXSID4027527	1,2,5,6,9,10-Hexabromocyclododecan	hexabromocyclododecane
DTXSID4027527	1,2,5,6,9,10-Hexabromocyclododecan	hexabromocyclododecane
DTXSID4027527	1,2,5,6,9,10-Hexabromocyclododecan	hexabromocyclododecane
DTXSID4027527	1,2,5,6,9,10-Hexabromocyclododecan	hexabromocyclododecane
DTXSID4027527	1,2,5,6,9,10-Hexabromocyclododecan	hexabromocyclododecane
DTXSID4027527	1,2,5,6,9,10-Hexabromocyclododecan	hexabromocyclododecane
DTXSID4027527	1,2,5,6,9,10-Hexabromocyclododecan	hexabromocyclododecane
DTXSID4027527	1,2,5,6,9,10-Hexabromocyclododecan	hexabromocyclododecane
DTXSID4027527	1,2,5,6,9,10-Hexabromocyclododecan	hexabromocyclododecane
DTXSID4027527	1,2,5,6,9,10-Hexabromocyclododecan	hexabromocyclododecane
DTXSID4027527	1,2,5,6,9,10-Hexabromocyclododecan	hexabromocyclododecane
DTXSID4027527	1,2,5,6,9,10-Hexabromocyclododecan	hexabromocyclododecane
DTXSID4027527	1,2,5,6,9,10-Hexabromocyclododecan	hexabromocyclododecane
DTXSID4027527	1,2,5,6,9,10-Hexabromocyclododecan	hexabromocyclododecane
DTXSID4027527	1,2,5,6,9,10-Hexabromocyclododecan	hexabromocyclododecane
DTXSID4027527	1,2,5,6,9,10-Hexabromocyclododecan	hexabromocyclododecane
DTXSID4027527	1,2,5,6,9,10-Hexabromocyclododecan	hexabromocyclododecane

DTXSID4027527	1,2,5,6,9,10-Hexabromocyclododecan‹	hexabromocyclododecane
DTXSID4027527	1,2,5,6,9,10-Hexabromocyclododecan‹	hexabromocyclododecane
DTXSID4027527	1,2,5,6,9,10-Hexabromocyclododecan‹	hexabromocyclododecane
DTXSID4027527	1,2,5,6,9,10-Hexabromocyclododecan‹	hexabromocyclododecane
DTXSID4027527	1,2,5,6,9,10-Hexabromocyclododecan‹	hexabromocyclododecane
DTXSID4027527	1,2,5,6,9,10-Hexabromocyclododecan‹	hexabromocyclododecane
DTXSID4027527	1,2,5,6,9,10-Hexabromocyclododecan‹	hexabromocyclododecane
DTXSID4027527	1,2,5,6,9,10-Hexabromocyclododecan‹	hexabromocyclododecane
DTXSID4027527	1,2,5,6,9,10-Hexabromocyclododecan‹	hexabromocyclododecane
DTXSID4027527	1,2,5,6,9,10-Hexabromocyclododecan‹	hexabromocyclododecane
DTXSID4027527	1,2,5,6,9,10-Hexabromocyclododecan‹	hexabromocyclododecane
DTXSID4027527	1,2,5,6,9,10-Hexabromocyclododecan‹	hexabromocyclododecane
DTXSID4027527	1,2,5,6,9,10-Hexabromocyclododecan‹	hexabromocyclododecane
DTXSID4027527	1,2,5,6,9,10-Hexabromocyclododecan‹	hexabromocyclododecane
DTXSID4027527	1,2,5,6,9,10-Hexabromocyclododecan‹	hexabromocyclododecane
DTXSID4027527	1,2,5,6,9,10-Hexabromocyclododecan‹	hexabromocyclododecane
DTXSID4027527	1,2,5,6,9,10-Hexabromocyclododecan‹	hexabromocyclododecane
DTXSID4027527	1,2,5,6,9,10-Hexabromocyclododecan‹	hexabromocyclododecane
DTXSID4027527	1,2,5,6,9,10-Hexabromocyclododecan‹	hexabromocyclododecane
DTXSID4027527	1,2,5,6,9,10-Hexabromocyclododecan‹	hexabromocyclododecane
DTXSID4027527	1,2,5,6,9,10-Hexabromocyclododecan‹	hexabromocyclododecane
DTXSID4027527	1,2,5,6,9,10-Hexabromocyclododecan‹	hexabromocyclododecane
DTXSID4027527	1,2,5,6,9,10-Hexabromocyclododecan‹	hexabromocyclododecane
DTXSID4027527	1,2,5,6,9,10-Hexabromocyclododecan‹	hexabromocyclododecane
DTXSID4027527	1,2,5,6,9,10-Hexabromocyclododecan‹	hexabromocyclododecane
DTXSID4027527	1,2,5,6,9,10-Hexabromocyclododecan‹	hexabromocyclododecane
DTXSID4027527	1,2,5,6,9,10-Hexabromocyclododecan‹	hexabromocyclododecane
DTXSID4027527	1,2,5,6,9,10-Hexabromocyclododecan‹	hexabromocyclododecane
DTXSID4027527	1,2,5,6,9,10-Hexabromocyclododecan‹	hexabromocyclododecane
DTXSID4027527	1,2,5,6,9,10-Hexabromocyclododecan‹	hexabromocyclododecane
DTXSID4027527	1,2,5,6,9,10-Hexabromocyclododecan‹	hexabromocyclododecane
DTXSID4027527	1,2,5,6,9,10-Hexabromocyclododecan‹	hexabromocyclododecane
DTXSID4027527	1,2,5,6,9,10-Hexabromocyclododecan‹	hexabromocyclododecane
DTXSID4027527	1,2,5,6,9,10-Hexabromocyclododecan‹	hexabromocyclododecane
DTXSID4027527	1,2,5,6,9,10-Hexabromocyclododecan‹	hexabromocyclododecane
DTXSID4027527	1,2,5,6,9,10-Hexabromocyclododecan‹	hexabromocyclododecane
DTXSID4027527	1,2,5,6,9,10-Hexabromocyclododecan‹	hexabromocyclododecane
DTXSID4027527	1,2,5,6,9,10-Hexabromocyclododecan‹	hexabromocyclododecane
DTXSID4027527	1,2,5,6,9,10-Hexabromocyclododecan‹	hexabromocyclododecane
DTXSID4027527	1,2,5,6,9,10-Hexabromocyclododecan‹	hexabromocyclododecane
DTXSID4027527	1,2,5,6,9,10-Hexabromocyclododecan‹	hexabromocyclododecane
DTXSID4027527	1,2,5,6,9,10-Hexabromocyclododecan‹	hexabromocyclododecane
DTXSID4027527	1,2,5,6,9,10-Hexabromocyclododecan‹	hexabromocyclododecane
DTXSID4027527	1,2,5,6,9,10-Hexabromocyclododecan‹	hexabromocyclododecane
DTXSID4027527	1,2,5,6,9,10-Hexabromocyclododecan‹	hexabromocyclododecane
DTXSID4027527	1,2,5,6,9,10-Hexabromocyclododecan‹	hexabromocyclododecane
DTXSID4027527	1,2,5,6,9,10-Hexabromocyclododecan‹	hexabromocyclododecane

DTXSID4027527	1,2,5,6,9,10-Hexabromocyclododecane	hexabromocyclododecane
DTXSID4027527	1,2,5,6,9,10-Hexabromocyclododecane	hexabromocyclododecane
DTXSID4027527	1,2,5,6,9,10-Hexabromocyclododecane	hexabromocyclododecane
DTXSID4027527	1,2,5,6,9,10-Hexabromocyclododecane	hexabromocyclododecane
DTXSID4027527	1,2,5,6,9,10-Hexabromocyclododecane	hexabromocyclododecane
DTXSID4027527	1,2,5,6,9,10-Hexabromocyclododecane	hexabromocyclododecane
DTXSID4027527	1,2,5,6,9,10-Hexabromocyclododecane	hexabromocyclododecane
DTXSID4027527	1,2,5,6,9,10-Hexabromocyclododecane	hexabromocyclododecane
DTXSID4027527	1,2,5,6,9,10-Hexabromocyclododecane	hexabromocyclododecane
DTXSID4027527	1,2,5,6,9,10-Hexabromocyclododecane	hexabromocyclododecane
DTXSID4027527	1,2,5,6,9,10-Hexabromocyclododecane	hexabromocyclododecane
DTXSID4027527	1,2,5,6,9,10-Hexabromocyclododecane	hexabromocyclododecane
DTXSID4027527	1,2,5,6,9,10-Hexabromocyclododecane	hexabromocyclododecane
DTXSID4027527	1,2,5,6,9,10-Hexabromocyclododecane	hexabromocyclododecane
DTXSID4027527	1,2,5,6,9,10-Hexabromocyclododecane	hexabromocyclododecane
DTXSID4027527	1,2,5,6,9,10-Hexabromocyclododecane	hexabromocyclododecane
DTXSID4027527	1,2,5,6,9,10-Hexabromocyclododecane	hexabromocyclododecane
DTXSID4027527	1,2,5,6,9,10-Hexabromocyclododecane	hexabromocyclododecane
DTXSID4027527	1,2,5,6,9,10-Hexabromocyclododecane	hexabromocyclododecane
DTXSID4027527	1,2,5,6,9,10-Hexabromocyclododecane	hexabromocyclododecane
DTXSID4027527	1,2,5,6,9,10-Hexabromocyclododecane	hexabromocyclododecane
DTXSID4027527	1,2,5,6,9,10-Hexabromocyclododecane	hexabromocyclododecane
DTXSID4027527	1,2,5,6,9,10-Hexabromocyclododecane	hexabromocyclododecane
DTXSID4027527	1,2,5,6,9,10-Hexabromocyclododecane	hexabromocyclododecane
DTXSID4027527	1,2,5,6,9,10-Hexabromocyclododecane	hexabromocyclododecane
DTXSID4027527	1,2,5,6,9,10-Hexabromocyclododecane	hexabromocyclododecane
DTXSID4027527	1,2,5,6,9,10-Hexabromocyclododecane	hexabromocyclododecane
DTXSID4027527	1,2,5,6,9,10-Hexabromocyclododecane	hexabromocyclododecane
DTXSID4027527	1,2,5,6,9,10-Hexabromocyclododecane	hexabromocyclododecane
DTXSID4027527	1,2,5,6,9,10-Hexabromocyclododecane	hexabromocyclododecane
DTXSID4027527	1,2,5,6,9,10-Hexabromocyclododecane	hexabromocyclododecane
DTXSID4027527	1,2,5,6,9,10-Hexabromocyclododecane	hexabromocyclododecane
DTXSID4027527	1,2,5,6,9,10-Hexabromocyclododecane	hexabromocyclododecane
DTXSID4027527	1,2,5,6,9,10-Hexabromocyclododecane	hexabromocyclododecane
DTXSID4027527	1,2,5,6,9,10-Hexabromocyclododecane	hexabromocyclododecane
DTXSID4027527	1,2,5,6,9,10-Hexabromocyclododecane	hexabromocyclododecane
DTXSID4027527	1,2,5,6,9,10-Hexabromocyclododecane	hexabromocyclododecane
DTXSID4027527	1,2,5,6,9,10-Hexabromocyclododecane	hexabromocyclododecane
DTXSID4027527	1,2,5,6,9,10-Hexabromocyclododecane	hexabromocyclododecane
DTXSID4027527	1,2,5,6,9,10-Hexabromocyclododecane	hexabromocyclododecane
DTXSID4027527	1,2,5,6,9,10-Hexabromocyclododecane	hexabromocyclododecane
DTXSID4027527	1,2,5,6,9,10-Hexabromocyclododecane	hexabromocyclododecane
DTXSID4027527	1,2,5,6,9,10-Hexabromocyclododecane	hexabromocyclododecane
DTXSID4027527	1,2,5,6,9,10-Hexabromocyclododecane	hexabromocyclododecane
DTXSID4027527	1,2,5,6,9,10-Hexabromocyclododecane	hexabromocyclododecane
DTXSID4027527	1,2,5,6,9,10-Hexabromocyclododecane	hexabromocyclododecane
DTXSID4027527	1,2,5,6,9,10-Hexabromocyclododecane	hexabromocyclododecane

DTXSID4027527	1,2,5,6,9,10-Hexabromocyclododecan	hexabromocyclododecane
DTXSID4027527	1,2,5,6,9,10-Hexabromocyclododecan	hexabromocyclododecane
DTXSID4027527	1,2,5,6,9,10-Hexabromocyclododecan	hexabromocyclododecane
DTXSID4027527	1,2,5,6,9,10-Hexabromocyclododecan	hexabromocyclododecane
DTXSID4027527	1,2,5,6,9,10-Hexabromocyclododecan	hexabromocyclododecane
DTXSID4027527	1,2,5,6,9,10-Hexabromocyclododecan	hexabromocyclododecane
DTXSID4027527	1,2,5,6,9,10-Hexabromocyclododecan	hexabromocyclododecane
DTXSID4027527	1,2,5,6,9,10-Hexabromocyclododecan	hexabromocyclododecane
DTXSID4027527	1,2,5,6,9,10-Hexabromocyclododecan	hexabromocyclododecane
DTXSID4027527	1,2,5,6,9,10-Hexabromocyclododecan	hexabromocyclododecane
DTXSID4027527	1,2,5,6,9,10-Hexabromocyclododecan	hexabromocyclododecane
DTXSID4027527	1,2,5,6,9,10-Hexabromocyclododecan	hexabromocyclododecane
DTXSID4027527	1,2,5,6,9,10-Hexabromocyclododecan	hexabromocyclododecane
DTXSID4027527	1,2,5,6,9,10-Hexabromocyclododecan	hexabromocyclododecane
DTXSID4027527	1,2,5,6,9,10-Hexabromocyclododecan	hexabromocyclododecane
DTXSID4027527	1,2,5,6,9,10-Hexabromocyclododecan	hexabromocyclododecane
DTXSID4027527	1,2,5,6,9,10-Hexabromocyclododecan	hexabromocyclododecane
DTXSID4027527	1,2,5,6,9,10-Hexabromocyclododecan	hexabromocyclododecane
DTXSID4027527	1,2,5,6,9,10-Hexabromocyclododecan	hexabromocyclododecane
DTXSID4027527	1,2,5,6,9,10-Hexabromocyclododecan	hexabromocyclododecane
DTXSID4027527	1,2,5,6,9,10-Hexabromocyclododecan	hexabromocyclododecane
DTXSID4027527	1,2,5,6,9,10-Hexabromocyclododecan	hexabromocyclododecane
DTXSID4027527	1,2,5,6,9,10-Hexabromocyclododecan	hexabromocyclododecane
DTXSID4027527	1,2,5,6,9,10-Hexabromocyclododecan	hexabromocyclododecane
DTXSID4027527	1,2,5,6,9,10-Hexabromocyclododecan	hexabromocyclododecane
DTXSID4027527	1,2,5,6,9,10-Hexabromocyclododecan	hexabromocyclododecane
DTXSID4027527	1,2,5,6,9,10-Hexabromocyclododecan	hexabromocyclododecane
DTXSID4027527	1,2,5,6,9,10-Hexabromocyclododecan	hexabromocyclododecane
DTXSID4042381	Silicic acid	Silicic Acid
DTXSID4049662	Magnesium hydroxide	Magnesium Hydroxide
DTXSID4052685	2,2',3,4,4'-Pentabromodiphenyl ether	pentabromodiphenyl ether
DTXSID4052685	2,2',3,4,4'-Pentabromodiphenyl ether	pentabromodiphenyl ether
DTXSID4052685	2,2',3,4,4'-Pentabromodiphenyl ether	pentabromodiphenyl ether
DTXSID4052685	2,2',3,4,4'-Pentabromodiphenyl ether	pentabromodiphenyl ether
DTXSID4052685	2,2',3,4,4'-Pentabromodiphenyl ether	pentabromodiphenyl ether
DTXSID4052685	2,2',3,4,4'-Pentabromodiphenyl ether	pentabromodiphenyl ether
DTXSID4052685	2,2',3,4,4'-Pentabromodiphenyl ether	pentabromodiphenyl ether
DTXSID4052685	2,2',3,4,4'-Pentabromodiphenyl ether	pentabromodiphenyl ether
DTXSID4052685	2,2',3,4,4'-Pentabromodiphenyl ether	pentabromodiphenyl ether
DTXSID4052685	2,2',3,4,4'-Pentabromodiphenyl ether	pentabromodiphenyl ether
DTXSID4052685	2,2',3,4,4'-Pentabromodiphenyl ether	pentabromodiphenyl ether
DTXSID4052685	2,2',3,4,4'-Pentabromodiphenyl ether	pentabromodiphenyl ether
DTXSID4052685	2,2',3,4,4'-Pentabromodiphenyl ether	pentabromodiphenyl ether
DTXSID4052685	2,2',3,4,4'-Pentabromodiphenyl ether	pentabromodiphenyl ether
DTXSID4052685	2,2',3,4,4'-Pentabromodiphenyl ether	pentabromodiphenyl ether
DTXSID4052685	2,2',3,4,4'-Pentabromodiphenyl ether	pentabromodiphenyl ether
DTXSID4052685	2,2',3,4,4'-Pentabromodiphenyl ether	pentabromodiphenyl ether

DTXSID4052685	2,2',3,4,4'-Pentabromodiphenyl ether	pentabromodiphenyl ether
DTXSID4052685	2,2',3,4,4'-Pentabromodiphenyl ether	pentabromodiphenyl ether
DTXSID4052685	2,2',3,4,4'-Pentabromodiphenyl ether	pentabromodiphenyl ether
DTXSID4052685	2,2',3,4,4'-Pentabromodiphenyl ether	pentabromodiphenyl ether
DTXSID4052685	2,2',3,4,4'-Pentabromodiphenyl ether	pentabromodiphenyl ether
DTXSID4052685	2,2',3,4,4'-Pentabromodiphenyl ether	pentabromodiphenyl ether
DTXSID4052685	2,2',3,4,4'-Pentabromodiphenyl ether	pentabromodiphenyl ether
DTXSID4052685	2,2',3,4,4'-Pentabromodiphenyl ether	pentabromodiphenyl ether
DTXSID4052685	2,2',3,4,4'-Pentabromodiphenyl ether	pentabromodiphenyl ether
DTXSID4052685	2,2',3,4,4'-Pentabromodiphenyl ether	pentabromodiphenyl ether
DTXSID4052685	2,2',3,4,4'-Pentabromodiphenyl ether	pentabromodiphenyl ether
DTXSID4052685	2,2',3,4,4'-Pentabromodiphenyl ether	pentabromodiphenyl ether
DTXSID4052685	2,2',3,4,4'-Pentabromodiphenyl ether	pentabromodiphenyl ether
DTXSID4052685	2,2',3,4,4'-Pentabromodiphenyl ether	pentabromodiphenyl ether
DTXSID4052685	2,2',3,4,4'-Pentabromodiphenyl ether	pentabromodiphenyl ether
DTXSID4052685	2,2',3,4,4'-Pentabromodiphenyl ether	pentabromodiphenyl ether
DTXSID4052685	2,2',3,4,4'-Pentabromodiphenyl ether	pentabromodiphenyl ether
DTXSID4052685	2,2',3,4,4'-Pentabromodiphenyl ether	pentabromodiphenyl ether
DTXSID4052685	2,2',3,4,4'-Pentabromodiphenyl ether	pentabromodiphenyl ether
DTXSID4052685	2,2',3,4,4'-Pentabromodiphenyl ether	pentabromodiphenyl ether
DTXSID4052685	2,2',3,4,4'-Pentabromodiphenyl ether	pentabromodiphenyl ether
DTXSID4052685	2,2',3,4,4'-Pentabromodiphenyl ether	pentabromodiphenyl ether
DTXSID4052685	2,2',3,4,4'-Pentabromodiphenyl ether	pentabromodiphenyl ether
DTXSID4052685	2,2',3,4,4'-Pentabromodiphenyl ether	pentabromodiphenyl ether
DTXSID4052685	2,2',3,4,4'-Pentabromodiphenyl ether	pentabromodiphenyl ether
DTXSID4052685	2,2',3,4,4'-Pentabromodiphenyl ether	pentabromodiphenyl ether
DTXSID4052685	2,2',3,4,4'-Pentabromodiphenyl ether	pentabromodiphenyl ether
DTXSID4052685	2,2',3,4,4'-Pentabromodiphenyl ether	pentabromodiphenyl ether
DTXSID4052685	2,2',3,4,4'-Pentabromodiphenyl ether	pentabromodiphenyl ether
DTXSID4052685	2,2',3,4,4'-Pentabromodiphenyl ether	pentabromodiphenyl ether
DTXSID4052685	2,2',3,4,4'-Pentabromodiphenyl ether	pentabromodiphenyl ether
DTXSID4052685	2,2',3,4,4'-Pentabromodiphenyl ether	pentabromodiphenyl ether
DTXSID4052685	2,2',3,4,4'-Pentabromodiphenyl ether	pentabromodiphenyl ether
DTXSID4052685	2,2',3,4,4'-Pentabromodiphenyl ether	pentabromodiphenyl ether
DTXSID4052685	2,2',3,4,4'-Pentabromodiphenyl ether	pentabromodiphenyl ether
DTXSID4052685	2,2',3,4,4'-Pentabromodiphenyl ether	pentabromodiphenyl ether
DTXSID4052685	2,2',3,4,4'-Pentabromodiphenyl ether	pentabromodiphenyl ether
DTXSID4052685	2,2',3,4,4'-Pentabromodiphenyl ether	pentabromodiphenyl ether
DTXSID4052685	2,2',3,4,4'-Pentabromodiphenyl ether	pentabromodiphenyl ether
DTXSID4052685	2,2',3,4,4'-Pentabromodiphenyl ether	pentabromodiphenyl ether
DTXSID4052685	2,2',3,4,4'-Pentabromodiphenyl ether	pentabromodiphenyl ether
DTXSID4052685	2,2',3,4,4'-Pentabromodiphenyl ether	pentabromodiphenyl ether
DTXSID4052685	2,2',3,4,4'-Pentabromodiphenyl ether	pentabromodiphenyl ether
DTXSID4052685	2,2',3,4,4'-Pentabromodiphenyl ether	pentabromodiphenyl ether
DTXSID4052685	2,2',3,4,4'-Pentabromodiphenyl ether	pentabromodiphenyl ether
DTXSID4052685	2,2',3,4,4'-Pentabromodiphenyl ether	pentabromodiphenyl ether
DTXSID4052685	2,2',3,4,4'-Pentabromodiphenyl ether	pentabromodiphenyl ether

DTXSID4052685	2,2',3,4,4'-Pentabromodiphenyl ether	pentabromodiphenyl ether
DTXSID4052685	2,2',3,4,4'-Pentabromodiphenyl ether	pentabromodiphenyl ether
DTXSID4052685	2,2',3,4,4'-Pentabromodiphenyl ether	pentabromodiphenyl ether
DTXSID4052685	2,2',3,4,4'-Pentabromodiphenyl ether	pentabromodiphenyl ether
DTXSID4052685	2,2',3,4,4'-Pentabromodiphenyl ether	pentabromodiphenyl ether
DTXSID4052685	2,2',3,4,4'-Pentabromodiphenyl ether	pentabromodiphenyl ether
DTXSID4052685	2,2',3,4,4'-Pentabromodiphenyl ether	pentabromodiphenyl ether
DTXSID4052685	2,2',3,4,4'-Pentabromodiphenyl ether	pentabromodiphenyl ether
DTXSID4052685	2,2',3,4,4'-Pentabromodiphenyl ether	pentabromodiphenyl ether
DTXSID4052685	2,2',3,4,4'-Pentabromodiphenyl ether	pentabromodiphenyl ether
DTXSID4052685	2,2',3,4,4'-Pentabromodiphenyl ether	pentabromodiphenyl ether
DTXSID4052685	2,2',3,4,4'-Pentabromodiphenyl ether	pentabromodiphenyl ether
DTXSID4052685	2,2',3,4,4'-Pentabromodiphenyl ether	pentabromodiphenyl ether
DTXSID4052685	2,2',3,4,4'-Pentabromodiphenyl ether	pentabromodiphenyl ether
DTXSID4052685	2,2',3,4,4'-Pentabromodiphenyl ether	pentabromodiphenyl ether
DTXSID4052685	2,2',3,4,4'-Pentabromodiphenyl ether	pentabromodiphenyl ether
DTXSID4052685	2,2',3,4,4'-Pentabromodiphenyl ether	pentabromodiphenyl ether
DTXSID4052685	2,2',3,4,4'-Pentabromodiphenyl ether	pentabromodiphenyl ether
DTXSID4052685	2,2',3,4,4'-Pentabromodiphenyl ether	pentabromodiphenyl ether
DTXSID4052685	2,2',3,4,4'-Pentabromodiphenyl ether	pentabromodiphenyl ether
DTXSID4052685	2,2',3,4,4'-Pentabromodiphenyl ether	pentabromodiphenyl ether
DTXSID4052685	2,2',3,4,4'-Pentabromodiphenyl ether	pentabromodiphenyl ether
DTXSID4052685	2,2',3,4,4'-Pentabromodiphenyl ether	pentabromodiphenyl ether
DTXSID4052685	2,2',3,4,4'-Pentabromodiphenyl ether	pentabromodiphenyl ether
DTXSID4052685	2,2',3,4,4'-Pentabromodiphenyl ether	pentabromodiphenyl ether
DTXSID4052685	2,2',3,4,4'-Pentabromodiphenyl ether	pentabromodiphenyl ether
DTXSID4052685	2,2',3,4,4'-Pentabromodiphenyl ether	pentabromodiphenyl ether
DTXSID4052685	2,2',3,4,4'-Pentabromodiphenyl ether	pentabromodiphenyl ether
DTXSID4052685	2,2',3,4,4'-Pentabromodiphenyl ether	pentabromodiphenyl ether
DTXSID4052685	2,2',3,4,4'-Pentabromodiphenyl ether	pentabromodiphenyl ether
DTXSID4052685	2,2',3,4,4'-Pentabromodiphenyl ether	pentabromodiphenyl ether
DTXSID4052685	2,2',3,4,4'-Pentabromodiphenyl ether	pentabromodiphenyl ether
DTXSID4052685	2,2',3,4,4'-Pentabromodiphenyl ether	pentabromodiphenyl ether
DTXSID4052685	2,2',3,4,4'-Pentabromodiphenyl ether	pentabromodiphenyl ether
DTXSID4052685	2,2',3,4,4'-Pentabromodiphenyl ether	pentabromodiphenyl ether
DTXSID4052685	2,2',3,4,4'-Pentabromodiphenyl ether	pentabromodiphenyl ether
DTXSID4052685	2,2',3,4,4'-Pentabromodiphenyl ether	pentabromodiphenyl ether
DTXSID4052685	2,2',3,4,4'-Pentabromodiphenyl ether	pentabromodiphenyl ether
DTXSID4052685	2,2',3,4,4'-Pentabromodiphenyl ether	pentabromodiphenyl ether
DTXSID4052685	2,2',3,4,4'-Pentabromodiphenyl ether	pentabromodiphenyl ether
DTXSID4052685	2,2',3,4,4'-Pentabromodiphenyl ether	pentabromodiphenyl ether
DTXSID4052685	2,2',3,4,4'-Pentabromodiphenyl ether	pentabromodiphenyl ether
DTXSID4052685	2,2',3,4,4'-Pentabromodiphenyl ether	pentabromodiphenyl ether
DTXSID4052685	2,2',3,4,4'-Pentabromodiphenyl ether	pentabromodiphenyl ether
DTXSID4052685	2,2',3,4,4'-Pentabromodiphenyl ether	pentabromodiphenyl ether
DTXSID4052685	2,2',3,4,4'-Pentabromodiphenyl ether	pentabromodiphenyl ether
DTXSID4052685	2,2',3,4,4'-Pentabromodiphenyl ether	pentabromodiphenyl ether

DTXSID4052685	2,2',3,4,4'-Pentabromodiphenyl ether	pentabromodiphenyl ether
DTXSID4052685	2,2',3,4,4'-Pentabromodiphenyl ether	pentabromodiphenyl ether
DTXSID4052685	2,2',3,4,4'-Pentabromodiphenyl ether	pentabromodiphenyl ether
DTXSID4052685	2,2',3,4,4'-Pentabromodiphenyl ether	pentabromodiphenyl ether
DTXSID4052685	2,2',3,4,4'-Pentabromodiphenyl ether	pentabromodiphenyl ether
DTXSID4052685	2,2',3,4,4'-Pentabromodiphenyl ether	pentabromodiphenyl ether
DTXSID4052685	2,2',3,4,4'-Pentabromodiphenyl ether	pentabromodiphenyl ether
DTXSID4052685	2,2',3,4,4'-Pentabromodiphenyl ether	pentabromodiphenyl ether
DTXSID4052685	2,2',3,4,4'-Pentabromodiphenyl ether	pentabromodiphenyl ether
DTXSID4052685	2,2',3,4,4'-Pentabromodiphenyl ether	pentabromodiphenyl ether
DTXSID4052685	2,2',3,4,4'-Pentabromodiphenyl ether	pentabromodiphenyl ether
DTXSID4052685	2,2',3,4,4'-Pentabromodiphenyl ether	pentabromodiphenyl ether
DTXSID4052685	2,2',3,4,4'-Pentabromodiphenyl ether	pentabromodiphenyl ether
DTXSID4052685	2,2',3,4,4'-Pentabromodiphenyl ether	pentabromodiphenyl ether
DTXSID4052685	2,2',3,4,4'-Pentabromodiphenyl ether	pentabromodiphenyl ether
DTXSID4052685	2,2',3,4,4'-Pentabromodiphenyl ether	pentabromodiphenyl ether
DTXSID4052685	2,2',3,4,4'-Pentabromodiphenyl ether	pentabromodiphenyl ether
DTXSID4052685	2,2',3,4,4'-Pentabromodiphenyl ether	pentabromodiphenyl ether
DTXSID4052685	2,2',3,4,4'-Pentabromodiphenyl ether	pentabromodiphenyl ether
DTXSID4052685	2,2',3,4,4'-Pentabromodiphenyl ether	pentabromodiphenyl ether
DTXSID4052685	2,2',3,4,4'-Pentabromodiphenyl ether	pentabromodiphenyl ether
DTXSID4052685	2,2',3,4,4'-Pentabromodiphenyl ether	pentabromodiphenyl ether
DTXSID4052685	2,2',3,4,4'-Pentabromodiphenyl ether	pentabromodiphenyl ether
DTXSID4052685	2,2',3,4,4'-Pentabromodiphenyl ether	pentabromodiphenyl ether
DTXSID4052685	2,2',3,4,4'-Pentabromodiphenyl ether	pentabromodiphenyl ether
DTXSID4052685	2,2',3,4,4'-Pentabromodiphenyl ether	pentabromodiphenyl ether
DTXSID4052685	2,2',3,4,4'-Pentabromodiphenyl ether	pentabromodiphenyl ether
DTXSID4052685	2,2',3,4,4'-Pentabromodiphenyl ether	pentabromodiphenyl ether
DTXSID4052685	2,2',3,4,4'-Pentabromodiphenyl ether	pentabromodiphenyl ether
DTXSID4052685	2,2',3,4,4'-Pentabromodiphenyl ether	pentabromodiphenyl ether
DTXSID4052685	2,2',3,4,4'-Pentabromodiphenyl ether	pentabromodiphenyl ether
DTXSID4052685	2,2',3,4,4'-Pentabromodiphenyl ether	pentabromodiphenyl ether
DTXSID4052685	2,2',3,4,4'-Pentabromodiphenyl ether	pentabromodiphenyl ether
DTXSID4052685	2,2',3,4,4'-Pentabromodiphenyl ether	pentabromodiphenyl ether
DTXSID4052685	2,2',3,4,4'-Pentabromodiphenyl ether	pentabromodiphenyl ether
DTXSID4052685	2,2',3,4,4'-Pentabromodiphenyl ether	pentabromodiphenyl ether
DTXSID4052685	2,2',3,4,4'-Pentabromodiphenyl ether	pentabromodiphenyl ether
DTXSID4052685	2,2',3,4,4'-Pentabromodiphenyl ether	pentabromodiphenyl ether
DTXSID4052685	2,2',3,4,4'-Pentabromodiphenyl ether	pentabromodiphenyl ether
DTXSID4052685	2,2',3,4,4'-Pentabromodiphenyl ether	pentabromodiphenyl ether
DTXSID4052685	2,2',3,4,4'-Pentabromodiphenyl ether	pentabromodiphenyl ether
DTXSID4052685	2,2',3,4,4'-Pentabromodiphenyl ether	pentabromodiphenyl ether
DTXSID4052685	2,2',3,4,4'-Pentabromodiphenyl ether	pentabromodiphenyl ether
DTXSID4052689	2,2',4,4',6-Pentabromodiphenyl ether	pentabromodiphenyl ether
DTXSID4052689	2,2',4,4',6-Pentabromodiphenyl ether	pentabromodiphenyl ether
DTXSID4052689	2,2',4,4',6-Pentabromodiphenyl ether	pentabromodiphenyl ether
DTXSID4052689	2,2',4,4',6-Pentabromodiphenyl ether	pentabromodiphenyl ether

DTXSID4052689	2,2',4,4',6-Pentabromodiphenyl ether	pentabromodiphenyl ether
DTXSID4052689	2,2',4,4',6-Pentabromodiphenyl ether	pentabromodiphenyl ether
DTXSID4052689	2,2',4,4',6-Pentabromodiphenyl ether	pentabromodiphenyl ether
DTXSID4052689	2,2',4,4',6-Pentabromodiphenyl ether	pentabromodiphenyl ether
DTXSID4052689	2,2',4,4',6-Pentabromodiphenyl ether	pentabromodiphenyl ether
DTXSID4052689	2,2',4,4',6-Pentabromodiphenyl ether	pentabromodiphenyl ether
DTXSID4052689	2,2',4,4',6-Pentabromodiphenyl ether	pentabromodiphenyl ether
DTXSID4052689	2,2',4,4',6-Pentabromodiphenyl ether	pentabromodiphenyl ether
DTXSID4052689	2,2',4,4',6-Pentabromodiphenyl ether	pentabromodiphenyl ether
DTXSID4052689	2,2',4,4',6-Pentabromodiphenyl ether	pentabromodiphenyl ether
DTXSID4052689	2,2',4,4',6-Pentabromodiphenyl ether	pentabromodiphenyl ether
DTXSID4052689	2,2',4,4',6-Pentabromodiphenyl ether	pentabromodiphenyl ether
DTXSID4052689	2,2',4,4',6-Pentabromodiphenyl ether	pentabromodiphenyl ether
DTXSID4052689	2,2',4,4',6-Pentabromodiphenyl ether	pentabromodiphenyl ether
DTXSID4052689	2,2',4,4',6-Pentabromodiphenyl ether	pentabromodiphenyl ether
DTXSID4052689	2,2',4,4',6-Pentabromodiphenyl ether	pentabromodiphenyl ether
DTXSID4052689	2,2',4,4',6-Pentabromodiphenyl ether	pentabromodiphenyl ether
DTXSID4052689	2,2',4,4',6-Pentabromodiphenyl ether	pentabromodiphenyl ether
DTXSID4052689	2,2',4,4',6-Pentabromodiphenyl ether	pentabromodiphenyl ether
DTXSID4052689	2,2',4,4',6-Pentabromodiphenyl ether	pentabromodiphenyl ether
DTXSID4052689	2,2',4,4',6-Pentabromodiphenyl ether	pentabromodiphenyl ether
DTXSID4052689	2,2',4,4',6-Pentabromodiphenyl ether	pentabromodiphenyl ether
DTXSID4052689	2,2',4,4',6-Pentabromodiphenyl ether	pentabromodiphenyl ether
DTXSID4052689	2,2',4,4',6-Pentabromodiphenyl ether	pentabromodiphenyl ether
DTXSID4052689	2,2',4,4',6-Pentabromodiphenyl ether	pentabromodiphenyl ether
DTXSID4052689	2,2',4,4',6-Pentabromodiphenyl ether	pentabromodiphenyl ether
DTXSID4052689	2,2',4,4',6-Pentabromodiphenyl ether	pentabromodiphenyl ether
DTXSID4052689	2,2',4,4',6-Pentabromodiphenyl ether	pentabromodiphenyl ether
DTXSID4052689	2,2',4,4',6-Pentabromodiphenyl ether	pentabromodiphenyl ether
DTXSID4052689	2,2',4,4',6-Pentabromodiphenyl ether	pentabromodiphenyl ether
DTXSID4052689	2,2',4,4',6-Pentabromodiphenyl ether	pentabromodiphenyl ether
DTXSID4052689	2,2',4,4',6-Pentabromodiphenyl ether	pentabromodiphenyl ether
DTXSID4052689	2,2',4,4',6-Pentabromodiphenyl ether	pentabromodiphenyl ether
DTXSID4052689	2,2',4,4',6-Pentabromodiphenyl ether	pentabromodiphenyl ether
DTXSID4052689	2,2',4,4',6-Pentabromodiphenyl ether	pentabromodiphenyl ether
DTXSID4052689	2,2',4,4',6-Pentabromodiphenyl ether	pentabromodiphenyl ether
DTXSID4052689	2,2',4,4',6-Pentabromodiphenyl ether	pentabromodiphenyl ether
DTXSID4052689	2,2',4,4',6-Pentabromodiphenyl ether	pentabromodiphenyl ether
DTXSID4052689	2,2',4,4',6-Pentabromodiphenyl ether	pentabromodiphenyl ether
DTXSID4052689	2,2',4,4',6-Pentabromodiphenyl ether	pentabromodiphenyl ether
DTXSID4052689	2,2',4,4',6-Pentabromodiphenyl ether	pentabromodiphenyl ether
DTXSID4052689	2,2',4,4',6-Pentabromodiphenyl ether	pentabromodiphenyl ether
DTXSID4052689	2,2',4,4',6-Pentabromodiphenyl ether	pentabromodiphenyl ether
DTXSID4052689	2,2',4,4',6-Pentabromodiphenyl ether	pentabromodiphenyl ether
DTXSID4052689	2,2',4,4',6-Pentabromodiphenyl ether	pentabromodiphenyl ether
DTXSID4052689	2,2',4,4',6-Pentabromodiphenyl ether	pentabromodiphenyl ether
DTXSID4052689	2,2',4,4',6-Pentabromodiphenyl ether	pentabromodiphenyl ether

DTXSID4052689	2,2',4,4',6-Pentabromodiphenyl ether	pentabromodiphenyl ether
DTXSID4052689	2,2',4,4',6-Pentabromodiphenyl ether	pentabromodiphenyl ether
DTXSID4052689	2,2',4,4',6-Pentabromodiphenyl ether	pentabromodiphenyl ether
DTXSID4052689	2,2',4,4',6-Pentabromodiphenyl ether	pentabromodiphenyl ether
DTXSID4052689	2,2',4,4',6-Pentabromodiphenyl ether	pentabromodiphenyl ether
DTXSID4052689	2,2',4,4',6-Pentabromodiphenyl ether	pentabromodiphenyl ether
DTXSID4052689	2,2',4,4',6-Pentabromodiphenyl ether	pentabromodiphenyl ether
DTXSID4052689	2,2',4,4',6-Pentabromodiphenyl ether	pentabromodiphenyl ether
DTXSID4052689	2,2',4,4',6-Pentabromodiphenyl ether	pentabromodiphenyl ether
DTXSID4052689	2,2',4,4',6-Pentabromodiphenyl ether	pentabromodiphenyl ether
DTXSID4052689	2,2',4,4',6-Pentabromodiphenyl ether	pentabromodiphenyl ether
DTXSID4052689	2,2',4,4',6-Pentabromodiphenyl ether	pentabromodiphenyl ether
DTXSID4052689	2,2',4,4',6-Pentabromodiphenyl ether	pentabromodiphenyl ether
DTXSID4052689	2,2',4,4',6-Pentabromodiphenyl ether	pentabromodiphenyl ether
DTXSID4052689	2,2',4,4',6-Pentabromodiphenyl ether	pentabromodiphenyl ether
DTXSID4052689	2,2',4,4',6-Pentabromodiphenyl ether	pentabromodiphenyl ether
DTXSID4052689	2,2',4,4',6-Pentabromodiphenyl ether	pentabromodiphenyl ether
DTXSID4052689	2,2',4,4',6-Pentabromodiphenyl ether	pentabromodiphenyl ether
DTXSID4052689	2,2',4,4',6-Pentabromodiphenyl ether	pentabromodiphenyl ether
DTXSID4052689	2,2',4,4',6-Pentabromodiphenyl ether	pentabromodiphenyl ether
DTXSID4052689	2,2',4,4',6-Pentabromodiphenyl ether	pentabromodiphenyl ether
DTXSID4052689	2,2',4,4',6-Pentabromodiphenyl ether	pentabromodiphenyl ether
DTXSID4052689	2,2',4,4',6-Pentabromodiphenyl ether	pentabromodiphenyl ether
DTXSID4052689	2,2',4,4',6-Pentabromodiphenyl ether	pentabromodiphenyl ether
DTXSID4052689	2,2',4,4',6-Pentabromodiphenyl ether	pentabromodiphenyl ether
DTXSID4052689	2,2',4,4',6-Pentabromodiphenyl ether	pentabromodiphenyl ether
DTXSID4052689	2,2',4,4',6-Pentabromodiphenyl ether	pentabromodiphenyl ether
DTXSID4052689	2,2',4,4',6-Pentabromodiphenyl ether	pentabromodiphenyl ether
DTXSID4052689	2,2',4,4',6-Pentabromodiphenyl ether	pentabromodiphenyl ether
DTXSID4052689	2,2',4,4',6-Pentabromodiphenyl ether	pentabromodiphenyl ether
DTXSID4052689	2,2',4,4',6-Pentabromodiphenyl ether	pentabromodiphenyl ether
DTXSID4052689	2,2',4,4',6-Pentabromodiphenyl ether	pentabromodiphenyl ether
DTXSID4052689	2,2',4,4',6-Pentabromodiphenyl ether	pentabromodiphenyl ether
DTXSID4052689	2,2',4,4',6-Pentabromodiphenyl ether	pentabromodiphenyl ether
DTXSID4052689	2,2',4,4',6-Pentabromodiphenyl ether	pentabromodiphenyl ether
DTXSID4052689	2,2',4,4',6-Pentabromodiphenyl ether	pentabromodiphenyl ether
DTXSID4052689	2,2',4,4',6-Pentabromodiphenyl ether	pentabromodiphenyl ether
DTXSID4052689	2,2',4,4',6-Pentabromodiphenyl ether	pentabromodiphenyl ether
DTXSID4052689	2,2',4,4',6-Pentabromodiphenyl ether	pentabromodiphenyl ether
DTXSID4052689	2,2',4,4',6-Pentabromodiphenyl ether	pentabromodiphenyl ether
DTXSID4052689	2,2',4,4',6-Pentabromodiphenyl ether	pentabromodiphenyl ether
DTXSID4052689	2,2',4,4',6-Pentabromodiphenyl ether	pentabromodiphenyl ether
DTXSID4052689	2,2',4,4',6-Pentabromodiphenyl ether	pentabromodiphenyl ether
DTXSID4052689	2,2',4,4',6-Pentabromodiphenyl ether	pentabromodiphenyl ether
DTXSID4052689	2,2',4,4',6-Pentabromodiphenyl ether	pentabromodiphenyl ether
DTXSID4052689	2,2',4,4',6-Pentabromodiphenyl ether	pentabromodiphenyl ether
DTXSID4052689	2,2',4,4',6-Pentabromodiphenyl ether	pentabromodiphenyl ether

DTXSID4052689	2,2',4,4',6-Pentabromodiphenyl ether	pentabromodiphenyl ether
DTXSID4052689	2,2',4,4',6-Pentabromodiphenyl ether	pentabromodiphenyl ether
DTXSID4052689	2,2',4,4',6-Pentabromodiphenyl ether	pentabromodiphenyl ether
DTXSID4052689	2,2',4,4',6-Pentabromodiphenyl ether	pentabromodiphenyl ether
DTXSID4052689	2,2',4,4',6-Pentabromodiphenyl ether	pentabromodiphenyl ether
DTXSID4052689	2,2',4,4',6-Pentabromodiphenyl ether	pentabromodiphenyl ether
DTXSID4052689	2,2',4,4',6-Pentabromodiphenyl ether	pentabromodiphenyl ether
DTXSID4052689	2,2',4,4',6-Pentabromodiphenyl ether	pentabromodiphenyl ether
DTXSID4052689	2,2',4,4',6-Pentabromodiphenyl ether	pentabromodiphenyl ether
DTXSID4052689	2,2',4,4',6-Pentabromodiphenyl ether	pentabromodiphenyl ether
DTXSID4052689	2,2',4,4',6-Pentabromodiphenyl ether	pentabromodiphenyl ether
DTXSID4052689	2,2',4,4',6-Pentabromodiphenyl ether	pentabromodiphenyl ether
DTXSID4052689	2,2',4,4',6-Pentabromodiphenyl ether	pentabromodiphenyl ether
DTXSID4052689	2,2',4,4',6-Pentabromodiphenyl ether	pentabromodiphenyl ether
DTXSID4052689	2,2',4,4',6-Pentabromodiphenyl ether	pentabromodiphenyl ether
DTXSID4052689	2,2',4,4',6-Pentabromodiphenyl ether	pentabromodiphenyl ether
DTXSID4052689	2,2',4,4',6-Pentabromodiphenyl ether	pentabromodiphenyl ether
DTXSID4052689	2,2',4,4',6-Pentabromodiphenyl ether	pentabromodiphenyl ether
DTXSID4052689	2,2',4,4',6-Pentabromodiphenyl ether	pentabromodiphenyl ether
DTXSID4052689	2,2',4,4',6-Pentabromodiphenyl ether	pentabromodiphenyl ether
DTXSID4052689	2,2',4,4',6-Pentabromodiphenyl ether	pentabromodiphenyl ether
DTXSID4052689	2,2',4,4',6-Pentabromodiphenyl ether	pentabromodiphenyl ether
DTXSID4052689	2,2',4,4',6-Pentabromodiphenyl ether	pentabromodiphenyl ether
DTXSID4052689	2,2',4,4',6-Pentabromodiphenyl ether	pentabromodiphenyl ether
DTXSID4052689	2,2',4,4',6-Pentabromodiphenyl ether	pentabromodiphenyl ether
DTXSID4052689	2,2',4,4',6-Pentabromodiphenyl ether	pentabromodiphenyl ether
DTXSID4052689	2,2',4,4',6-Pentabromodiphenyl ether	pentabromodiphenyl ether
DTXSID4052689	2,2',4,4',6-Pentabromodiphenyl ether	pentabromodiphenyl ether
DTXSID4052689	2,2',4,4',6-Pentabromodiphenyl ether	pentabromodiphenyl ether
DTXSID4052689	2,2',4,4',6-Pentabromodiphenyl ether	pentabromodiphenyl ether
DTXSID4052689	2,2',4,4',6-Pentabromodiphenyl ether	pentabromodiphenyl ether
DTXSID4052689	2,2',4,4',6-Pentabromodiphenyl ether	pentabromodiphenyl ether
DTXSID4052689	2,2',4,4',6-Pentabromodiphenyl ether	pentabromodiphenyl ether
DTXSID4052689	2,2',4,4',6-Pentabromodiphenyl ether	pentabromodiphenyl ether
DTXSID4052689	2,2',4,4',6-Pentabromodiphenyl ether	pentabromodiphenyl ether
DTXSID4052689	2,2',4,4',6-Pentabromodiphenyl ether	pentabromodiphenyl ether
DTXSID4052689	2,2',4,4',6-Pentabromodiphenyl ether	pentabromodiphenyl ether
DTXSID4052689	2,2',4,4',6-Pentabromodiphenyl ether	pentabromodiphenyl ether
DTXSID4052689	2,2',4,4',6-Pentabromodiphenyl ether	pentabromodiphenyl ether
DTXSID4052689	2,2',4,4',6-Pentabromodiphenyl ether	pentabromodiphenyl ether
DTXSID4052689	2,2',4,4',6-Pentabromodiphenyl ether	pentabromodiphenyl ether
DTXSID4052689	2,2',4,4',6-Pentabromodiphenyl ether	pentabromodiphenyl ether
DTXSID4052689	2,2',4,4',6-Pentabromodiphenyl ether	pentabromodiphenyl ether
DTXSID4052689	2,2',4,4',6-Pentabromodiphenyl ether	pentabromodiphenyl ether
DTXSID4052689	2,2',4,4',6-Pentabromodiphenyl ether	pentabromodiphenyl ether
DTXSID4052689	2,2',4,4',6-Pentabromodiphenyl ether	pentabromodiphenyl ether
DTXSID4052689	2,2',4,4',6-Pentabromodiphenyl ether	pentabromodiphenyl ether

DTXSID4052689	2,2',4,4',6-Pentabromodiphenyl ether	pentabromodiphenyl ether
DTXSID4052689	2,2',4,4',6-Pentabromodiphenyl ether	pentabromodiphenyl ether
DTXSID4052689	2,2',4,4',6-Pentabromodiphenyl ether	pentabromodiphenyl ether
DTXSID4052689	2,2',4,4',6-Pentabromodiphenyl ether	pentabromodiphenyl ether
DTXSID4052689	2,2',4,4',6-Pentabromodiphenyl ether	pentabromodiphenyl ether
DTXSID4052689	2,2',4,4',6-Pentabromodiphenyl ether	pentabromodiphenyl ether
DTXSID4052689	2,2',4,4',6-Pentabromodiphenyl ether	pentabromodiphenyl ether
DTXSID4052689	2,2',4,4',6-Pentabromodiphenyl ether	pentabromodiphenyl ether
DTXSID4052689	2,2',4,4',6-Pentabromodiphenyl ether	pentabromodiphenyl ether
DTXSID40556652	2,2',3,3',4,5,5',6,6'-nonabromodipheny	2,2',3,3',4,5,5',6,6'-nonabromodiphenyl ether
DTXSID40556652	2,2',3,3',4,5,5',6,6'-nonabromodipheny	2,2',3,3',4,5,5',6,6'-nonabromodiphenyl ether
DTXSID4074932	Perfluoro-2-methyl-3-pentanone	perfluoro-2-methyl-3-pentanone
DTXSID40872703	2,2',4-Tribromodiphenyl ether	tribromodiphenyl ether 28
DTXSID40889331	myo-Inositol hexakisphosphate	Phytic Acid
DTXSID40889331	myo-Inositol hexakisphosphate	Phytic Acid
DTXSID40889331	myo-Inositol hexakisphosphate	Phytic Acid
DTXSID40893054	Tribromoneopentyl alcohol	tribromoneopentyl alcohol
DTXSID40893054	Tribromoneopentyl alcohol	tribromoneopentyl alcohol
DTXSID40893054	Tribromoneopentyl alcohol	tribromoneopentyl alcohol
DTXSID40893054	Tribromoneopentyl alcohol	tribromoneopentyl alcohol
DTXSID40893054	Tribromoneopentyl alcohol	tribromoneopentyl alcohol
DTXSID501017085	2,3-Dibromopropylphosphate	2,3-dibromopropylphosphate
DTXSID5020023	Acrolein	Acrolein
DTXSID5020023	Acrolein	Acrolein
DTXSID5020023	Acrolein	Acrolein
DTXSID5020023	Acrolein	Acrolein
DTXSID5020100	Aroclor 1254	Chlorodiphenyl (54% Chlorine)
DTXSID5021174	Polybrominated biphenyls (PBB)	Firemaster BP-6
DTXSID5021174	Polybrominated biphenyls (PBB)	Firemaster BP-6
DTXSID5021174	Polybrominated biphenyls (PBB)	Firemaster BP-6
DTXSID5021174	Polybrominated biphenyls (PBB)	Firemaster BP-6
DTXSID5021174	Polybrominated biphenyls (PBB)	Firemaster BP-6
DTXSID5021174	Polybrominated biphenyls (PBB)	Firemaster BP-6
DTXSID5021174	Polybrominated biphenyls (PBB)	Firemaster BP-6
DTXSID5021174	Polybrominated biphenyls (PBB)	Firemaster BP-6
DTXSID5021174	Polybrominated biphenyls (PBB)	Firemaster BP-6
DTXSID5021330	Tetramethylolphosphonium chloride	tetramethylolphosphonium chloride
DTXSID5021330	Tetramethylolphosphonium chloride	tetramethylolphosphonium chloride
DTXSID5021411	Tris(2-chloroethyl) phosphate	tris(chloroethyl)phosphate
DTXSID5021411	Tris(2-chloroethyl) phosphate	tris(chloroethyl)phosphate
DTXSID5021411	Tris(2-chloroethyl) phosphate	tris(chloroethyl)phosphate
DTXSID5021411	Tris(2-chloroethyl) phosphate	tris(chloroethyl)phosphate
DTXSID5021411	Tris(2-chloroethyl) phosphate	tris(chloroethyl)phosphate
DTXSID5021411	Tris(2-chloroethyl) phosphate	tris(chloroethyl)phosphate
DTXSID5021411	Tris(2-chloroethyl) phosphate	tris(chloroethyl)phosphate
DTXSID5021411	Tris(2-chloroethyl) phosphate	tris(chloroethyl)phosphate
DTXSID5021411	Tris(2-chloroethyl) phosphate	tris(chloroethyl)phosphate

DTXSID5021411	Tris(2-chloroethyl) phosphate	tris(chloroethyl)phosphate
DTXSID5021411	Tris(2-chloroethyl) phosphate	tris(chloroethyl)phosphate
DTXSID5021411	Tris(2-chloroethyl) phosphate	tris(chloroethyl)phosphate
DTXSID5021411	Tris(2-chloroethyl) phosphate	tris(chloroethyl)phosphate
DTXSID5021411	Tris(2-chloroethyl) phosphate	tris(chloroethyl)phosphate
DTXSID5021411	Tris(2-chloroethyl) phosphate	tris(chloroethyl)phosphate
DTXSID5021411	Tris(2-chloroethyl) phosphate	tris(chloroethyl)phosphate
DTXSID5021411	Tris(2-chloroethyl) phosphate	tris(chloroethyl)phosphate
DTXSID5021411	Tris(2-chloroethyl) phosphate	tris(chloroethyl)phosphate
DTXSID5021411	Tris(2-chloroethyl) phosphate	tris(chloroethyl)phosphate
DTXSID5021411	Tris(2-chloroethyl) phosphate	tris(chloroethyl)phosphate
DTXSID5021411	Tris(2-chloroethyl) phosphate	tris(chloroethyl)phosphate
DTXSID5021411	Tris(2-chloroethyl) phosphate	tris(chloroethyl)phosphate
DTXSID5021411	Tris(2-chloroethyl) phosphate	tris(chloroethyl)phosphate
DTXSID5021411	Tris(2-chloroethyl) phosphate	tris(chloroethyl)phosphate
DTXSID5021411	Tris(2-chloroethyl) phosphate	tris(chloroethyl)phosphate
DTXSID5021411	Tris(2-chloroethyl) phosphate	tris(chloroethyl)phosphate
DTXSID5021411	Tris(2-chloroethyl) phosphate	tris(chloroethyl)phosphate
DTXSID5021411	Tris(2-chloroethyl) phosphate	tris(chloroethyl)phosphate
DTXSID5021411	Tris(2-chloroethyl) phosphate	tris(chloroethyl)phosphate
DTXSID5021411	Tris(2-chloroethyl) phosphate	tris(chloroethyl)phosphate
DTXSID5021411	Tris(2-chloroethyl) phosphate	tris(chloroethyl)phosphate
DTXSID5021413	Tris(2,3-dibromopropyl) phosphate	tris(2,3-dibromopropyl)phosphate
DTXSID5021413	Tris(2,3-dibromopropyl) phosphate	tris(2,3-dibromopropyl)phosphate
DTXSID5021413	Tris(2,3-dibromopropyl) phosphate	tris(2,3-dibromopropyl)phosphate
DTXSID5021413	Tris(2,3-dibromopropyl) phosphate	tris(2,3-dibromopropyl)phosphate
DTXSID5021413	Tris(2,3-dibromopropyl) phosphate	tris(2,3-dibromopropyl)phosphate
DTXSID5021413	Tris(2,3-dibromopropyl) phosphate	tris(2,3-dibromopropyl)phosphate
DTXSID5021413	Tris(2,3-dibromopropyl) phosphate	tris(2,3-dibromopropyl)phosphate
DTXSID5021413	Tris(2,3-dibromopropyl) phosphate	tris(2,3-dibromopropyl)phosphate
DTXSID5021413	Tris(2,3-dibromopropyl) phosphate	tris(2,3-dibromopropyl)phosphate
DTXSID5021413	Tris(2,3-dibromopropyl) phosphate	tris(2,3-dibromopropyl)phosphate
DTXSID5021413	Tris(2,3-dibromopropyl) phosphate	tris(2,3-dibromopropyl)phosphate
DTXSID5021413	Tris(2,3-dibromopropyl) phosphate	tris(2,3-dibromopropyl)phosphate
DTXSID5021413	Tris(2,3-dibromopropyl) phosphate	tris(2,3-dibromopropyl)phosphate
DTXSID5021413	Tris(2,3-dibromopropyl) phosphate	tris(2,3-dibromopropyl)phosphate
DTXSID5021413	Tris(2,3-dibromopropyl) phosphate	tris(2,3-dibromopropyl)phosphate
DTXSID5021413	Tris(2,3-dibromopropyl) phosphate	tris(2,3-dibromopropyl)phosphate
DTXSID5021413	Tris(2,3-dibromopropyl) phosphate	tris(2,3-dibromopropyl)phosphate
DTXSID5021413	Tris(2,3-dibromopropyl) phosphate	tris(2,3-dibromopropyl)phosphate
DTXSID5021413	Tris(2,3-dibromopropyl) phosphate	tris(2,3-dibromopropyl)phosphate
DTXSID5021413	Tris(2,3-dibromopropyl) phosphate	tris(2,3-dibromopropyl)phosphate
DTXSID5021413	Tris(2,3-dibromopropyl) phosphate	tris(2,3-dibromopropyl)phosphate
DTXSID5021413	Tris(2,3-dibromopropyl) phosphate	tris(2,3-dibromopropyl)phosphate
DTXSID5021413	Tris(2,3-dibromopropyl) phosphate	tris(2,3-dibromopropyl)phosphate
DTXSID5021413	Tris(2,3-dibromopropyl) phosphate	tris(2,3-dibromopropyl)phosphate
DTXSID5021413	Tris(2,3-dibromopropyl) phosphate	tris(2,3-dibromopropyl)phosphate

DTXSID5021413	Tris(2,3-dibromopropyl) phosphate	tris(2,3-dibromopropyl)phosphate
DTXSID5021413	Tris(2,3-dibromopropyl) phosphate	tris(2,3-dibromopropyl)phosphate
DTXSID5021413	Tris(2,3-dibromopropyl) phosphate	tris(2,3-dibromopropyl)phosphate
DTXSID5021413	Tris(2,3-dibromopropyl) phosphate	tris(2,3-dibromopropyl)phosphate
DTXSID5021413	Tris(2,3-dibromopropyl) phosphate	tris(2,3-dibromopropyl)phosphate
DTXSID5021413	Tris(2,3-dibromopropyl) phosphate	tris(2,3-dibromopropyl)phosphate
DTXSID5021413	Tris(2,3-dibromopropyl) phosphate	tris(2,3-dibromopropyl)phosphate
DTXSID5021413	Tris(2,3-dibromopropyl) phosphate	tris(2,3-dibromopropyl)phosphate
DTXSID5021413	Tris(2,3-dibromopropyl) phosphate	tris(2,3-dibromopropyl)phosphate
DTXSID5021413	Tris(2,3-dibromopropyl) phosphate	tris(2,3-dibromopropyl)phosphate
DTXSID5021413	Tris(2,3-dibromopropyl) phosphate	tris(2,3-dibromopropyl)phosphate
DTXSID5021413	Tris(2,3-dibromopropyl) phosphate	tris(2,3-dibromopropyl)phosphate
DTXSID5021413	Tris(2,3-dibromopropyl) phosphate	tris(2,3-dibromopropyl)phosphate
DTXSID5021413	Tris(2,3-dibromopropyl) phosphate	tris(2,3-dibromopropyl)phosphate
DTXSID5021413	Tris(2,3-dibromopropyl) phosphate	tris(2,3-dibromopropyl)phosphate
DTXSID5021413	Tris(2,3-dibromopropyl) phosphate	tris(2,3-dibromopropyl)phosphate
DTXSID5021413	Tris(2,3-dibromopropyl) phosphate	tris(2,3-dibromopropyl)phosphate
DTXSID5021413	Tris(2,3-dibromopropyl) phosphate	tris(2,3-dibromopropyl)phosphate
DTXSID5021413	Tris(2,3-dibromopropyl) phosphate	tris(2,3-dibromopropyl)phosphate
DTXSID5021413	Tris(2,3-dibromopropyl) phosphate	tris(2,3-dibromopropyl)phosphate
DTXSID5021413	Tris(2,3-dibromopropyl) phosphate	tris(2,3-dibromopropyl)phosphate
DTXSID5021413	Tris(2,3-dibromopropyl) phosphate	tris(2,3-dibromopropyl)phosphate
DTXSID5021413	Tris(2,3-dibromopropyl) phosphate	tris(2,3-dibromopropyl)phosphate
DTXSID5021413	Tris(2,3-dibromopropyl) phosphate	tris(2,3-dibromopropyl)phosphate
DTXSID5021413	Tris(2,3-dibromopropyl) phosphate	tris(2,3-dibromopropyl)phosphate
DTXSID5021413	Tris(2,3-dibromopropyl) phosphate	tris(2,3-dibromopropyl)phosphate
DTXSID5021413	Tris(2,3-dibromopropyl) phosphate	tris(2,3-dibromopropyl)phosphate
DTXSID5021413	Tris(2,3-dibromopropyl) phosphate	tris(2,3-dibromopropyl)phosphate
DTXSID5021413	Tris(2,3-dibromopropyl) phosphate	tris(2,3-dibromopropyl)phosphate
DTXSID5021413	Tris(2,3-dibromopropyl) phosphate	tris(2,3-dibromopropyl)phosphate
DTXSID5021413	Tris(2,3-dibromopropyl) phosphate	tris(2,3-dibromopropyl)phosphate
DTXSID5021413	Tris(2,3-dibromopropyl) phosphate	tris(2,3-dibromopropyl)phosphate
DTXSID5021413	Tris(2,3-dibromopropyl) phosphate	tris(2,3-dibromopropyl)phosphate
DTXSID5021413	Tris(2,3-dibromopropyl) phosphate	tris(2,3-dibromopropyl)phosphate
DTXSID5021413	Tris(2,3-dibromopropyl) phosphate	tris(2,3-dibromopropyl)phosphate
DTXSID5021758	Tris(2-butoxyethyl) phosphate	tris(2-butoxyethyl) phosphate
DTXSID5021758	Tris(2-butoxyethyl) phosphate	tris(2-butoxyethyl) phosphate
DTXSID5021758	Tris(2-butoxyethyl) phosphate	tris(2-butoxyethyl) phosphate
DTXSID5021758	Tris(2-butoxyethyl) phosphate	tris(2-butoxyethyl) phosphate
DTXSID5021758	Tris(2-butoxyethyl) phosphate	tris(2-butoxyethyl) phosphate
DTXSID5021758	Tris(2-butoxyethyl) phosphate	tris(2-butoxyethyl) phosphate
DTXSID5021758	Tris(2-butoxyethyl) phosphate	tris(2-butoxyethyl) phosphate
DTXSID5021758	Tris(2-butoxyethyl) phosphate	tris(2-butoxyethyl) phosphate
DTXSID5021758	Tris(2-butoxyethyl) phosphate	tris(2-butoxyethyl) phosphate
DTXSID5021758	Tris(2-butoxyethyl) phosphate	tris(2-butoxyethyl) phosphate
DTXSID5021758	Tris(2-butoxyethyl) phosphate	tris(2-butoxyethyl) phosphate
DTXSID5021758	Tris(2-butoxyethyl) phosphate	tris(2-butoxyethyl) phosphate

DTXSID5021758	Tris(2-butoxyethyl) phosphate	tris(2-butoxyethyl) phosphate
DTXSID5021758	Tris(2-butoxyethyl) phosphate	tris(2-butoxyethyl) phosphate
DTXSID5021758	Tris(2-butoxyethyl) phosphate	tris(2-butoxyethyl) phosphate
DTXSID5021758	Tris(2-butoxyethyl) phosphate	tris(2-butoxyethyl) phosphate
DTXSID5021758	Tris(2-butoxyethyl) phosphate	tris(2-butoxyethyl) phosphate
DTXSID5023875	Ammonium sulfamate	sulfamic acid
DTXSID5024267	Polychlorinated biphenyls	Polychlorinated Biphenyls
DTXSID5024267	Polychlorinated biphenyls	Polychlorinated Biphenyls
DTXSID5024267	Polychlorinated biphenyls	Polychlorinated Biphenyls
DTXSID5024267	Polychlorinated biphenyls	Polychlorinated Biphenyls
DTXSID5024267	Polychlorinated biphenyls	Polychlorinated Biphenyls
DTXSID5024267	Polychlorinated biphenyls	Polychlorinated Biphenyls
DTXSID5024267	Polychlorinated biphenyls	Polychlorinated Biphenyls
DTXSID5024267	Polychlorinated biphenyls	Polychlorinated Biphenyls
DTXSID5024267	Polychlorinated biphenyls	Polychlorinated Biphenyls
DTXSID5024267	Polychlorinated biphenyls	Polychlorinated Biphenyls
DTXSID5024267	Polychlorinated biphenyls	Polychlorinated Biphenyls
DTXSID5024267	Polychlorinated biphenyls	Polychlorinated Biphenyls
DTXSID5024267	Polychlorinated biphenyls	Polychlorinated Biphenyls
DTXSID5024267	Polychlorinated biphenyls	Polychlorinated Biphenyls
DTXSID5024267	Polychlorinated biphenyls	Polychlorinated Biphenyls
DTXSID5024267	Polychlorinated biphenyls	Polychlorinated Biphenyls
DTXSID5024267	Polychlorinated biphenyls	Polychlorinated Biphenyls
DTXSID5024267	Polychlorinated biphenyls	Polychlorinated Biphenyls
DTXSID5024267	Polychlorinated biphenyls	Polychlorinated Biphenyls
DTXSID5024267	Polychlorinated biphenyls	Polychlorinated Biphenyls
DTXSID5024267	Polychlorinated biphenyls	Polychlorinated Biphenyls
DTXSID5024267	Polychlorinated biphenyls	Polychlorinated Biphenyls
DTXSID5024267	Polychlorinated biphenyls	Polychlorinated Biphenyls
DTXSID5024267	Polychlorinated biphenyls	Polychlorinated Biphenyls
DTXSID5024267	Polychlorinated biphenyls	Polychlorinated Biphenyls
DTXSID5024267	Polychlorinated biphenyls	Polychlorinated Biphenyls
DTXSID5024267	Polychlorinated biphenyls	Polychlorinated Biphenyls
DTXSID5024267	Polychlorinated biphenyls	Polychlorinated Biphenyls
DTXSID5024267	Polychlorinated biphenyls	Polychlorinated Biphenyls
DTXSID5024267	Polychlorinated biphenyls	Polychlorinated Biphenyls
DTXSID5024267	Polychlorinated biphenyls	Polychlorinated Biphenyls
DTXSID5024267	Polychlorinated biphenyls	Polychlorinated Biphenyls
DTXSID5024267	Polychlorinated biphenyls	Polychlorinated Biphenyls
DTXSID5024267	Polychlorinated biphenyls	Polychlorinated Biphenyls
DTXSID5024267	Polychlorinated biphenyls	Polychlorinated Biphenyls
DTXSID5024267	Polychlorinated biphenyls	Polychlorinated Biphenyls
DTXSID5024267	Polychlorinated biphenyls	Polychlorinated Biphenyls
DTXSID5024267	Polychlorinated biphenyls	Polychlorinated Biphenyls
DTXSID5024267	Polychlorinated biphenyls	Polychlorinated Biphenyls
DTXSID5024267	Polychlorinated biphenyls	Polychlorinated Biphenyls
DTXSID5024267	Polychlorinated biphenyls	Polychlorinated Biphenyls

DTXSID5024267	Polychlorinated biphenyls	Polychlorinated Biphenyls
DTXSID5024267	Polychlorinated biphenyls	Polychlorinated Biphenyls
DTXSID5024267	Polychlorinated biphenyls	Polychlorinated Biphenyls
DTXSID5024267	Polychlorinated biphenyls	Polychlorinated Biphenyls
DTXSID5024267	Polychlorinated biphenyls	Polychlorinated Biphenyls
DTXSID5024267	Polychlorinated biphenyls	Polychlorinated Biphenyls
DTXSID5024267	Polychlorinated biphenyls	Polychlorinated Biphenyls
DTXSID5024267	Polychlorinated biphenyls	Polychlorinated Biphenyls
DTXSID5024267	Polychlorinated biphenyls	Polychlorinated Biphenyls
DTXSID5024267	Polychlorinated biphenyls	Polychlorinated Biphenyls
DTXSID5024267	Polychlorinated biphenyls	Polychlorinated Biphenyls
DTXSID5024267	Polychlorinated biphenyls	Polychlorinated Biphenyls
DTXSID5024267	Polychlorinated biphenyls	Polychlorinated Biphenyls
DTXSID5024267	Polychlorinated biphenyls	Polychlorinated Biphenyls
DTXSID5024267	Polychlorinated biphenyls	Polychlorinated Biphenyls
DTXSID5024267	Polychlorinated biphenyls	Polychlorinated Biphenyls
DTXSID5024267	Polychlorinated biphenyls	Polychlorinated Biphenyls
DTXSID5024267	Polychlorinated biphenyls	Polychlorinated Biphenyls
DTXSID5024267	Polychlorinated biphenyls	Polychlorinated Biphenyls
DTXSID5024267	Polychlorinated biphenyls	Polychlorinated Biphenyls
DTXSID5024267	Polychlorinated biphenyls	Polychlorinated Biphenyls
DTXSID5024267	Polychlorinated biphenyls	Polychlorinated Biphenyls
DTXSID5024267	Polychlorinated biphenyls	Polychlorinated Biphenyls
DTXSID5024267	Polychlorinated biphenyls	Polychlorinated Biphenyls
DTXSID5024267	Polychlorinated biphenyls	Polychlorinated Biphenyls
DTXSID5024267	Polychlorinated biphenyls	Polychlorinated Biphenyls
DTXSID5024267	Polychlorinated biphenyls	Polychlorinated Biphenyls
DTXSID5024267	Polychlorinated biphenyls	Polychlorinated Biphenyls
DTXSID5024267	Polychlorinated biphenyls	Polychlorinated Biphenyls
DTXSID5024267	Polychlorinated biphenyls	Polychlorinated Biphenyls
DTXSID5024267	Polychlorinated biphenyls	Polychlorinated Biphenyls
DTXSID5024267	Polychlorinated biphenyls	Polychlorinated Biphenyls
DTXSID5024267	Polychlorinated biphenyls	Polychlorinated Biphenyls
DTXSID5024267	Polychlorinated biphenyls	Polychlorinated Biphenyls
DTXSID5024267	Polychlorinated biphenyls	Polychlorinated Biphenyls
DTXSID5024267	Polychlorinated biphenyls	Polychlorinated Biphenyls
DTXSID5024267	Polychlorinated biphenyls	Polychlorinated Biphenyls
DTXSID5024267	Polychlorinated biphenyls	Polychlorinated Biphenyls
DTXSID5024267	Polychlorinated biphenyls	Polychlorinated Biphenyls
DTXSID5024267	Polychlorinated biphenyls	Polychlorinated Biphenyls
DTXSID5024267	Polychlorinated biphenyls	Polychlorinated Biphenyls
DTXSID5024267	Polychlorinated biphenyls	Polychlorinated Biphenyls
DTXSID5024267	Polychlorinated biphenyls	Polychlorinated Biphenyls
DTXSID5024267	Polychlorinated biphenyls	Polychlorinated Biphenyls
DTXSID5024267	Polychlorinated biphenyls	Polychlorinated Biphenyls
DTXSID5024267	Polychlorinated biphenyls	Polychlorinated Biphenyls
DTXSID5024267	Polychlorinated biphenyls	Polychlorinated Biphenyls

DTXSID5024267	Polychlorinated biphenyls	Polychlorinated Biphenyls
DTXSID5024267	Polychlorinated biphenyls	Polychlorinated Biphenyls
DTXSID5024267	Polychlorinated biphenyls	Polychlorinated Biphenyls
DTXSID5024267	Polychlorinated biphenyls	Polychlorinated Biphenyls
DTXSID5024267	Polychlorinated biphenyls	Polychlorinated Biphenyls
DTXSID5024267	Polychlorinated biphenyls	Polychlorinated Biphenyls
DTXSID5024267	Polychlorinated biphenyls	Polychlorinated Biphenyls
DTXSID5024267	Polychlorinated biphenyls	Polychlorinated Biphenyls
DTXSID5024267	Polychlorinated biphenyls	Polychlorinated Biphenyls
DTXSID5024267	Polychlorinated biphenyls	Polychlorinated Biphenyls
DTXSID5024267	Polychlorinated biphenyls	Polychlorinated Biphenyls
DTXSID5024267	Polychlorinated biphenyls	Polychlorinated Biphenyls
DTXSID5024267	Polychlorinated biphenyls	Polychlorinated Biphenyls
DTXSID5024267	Polychlorinated biphenyls	Polychlorinated Biphenyls
DTXSID5024267	Polychlorinated biphenyls	Polychlorinated Biphenyls
DTXSID5026259	Tris(2-chloroisopropyl)phosphate	tri-(2-chloroisopropyl)phosphate
DTXSID5026259	Tris(2-chloroisopropyl)phosphate	tri-(2-chloroisopropyl)phosphate
DTXSID5026259	Tris(2-chloroisopropyl)phosphate	tri-(2-chloroisopropyl)phosphate
DTXSID5026415	Bromotrifluoromethane	bromotrifluoromethane
DTXSID5026415	Bromotrifluoromethane	bromotrifluoromethane
DTXSID5026415	Bromotrifluoromethane	bromotrifluoromethane
DTXSID5026415	Bromotrifluoromethane	bromotrifluoromethane
DTXSID5026415	Bromotrifluoromethane	bromotrifluoromethane
DTXSID5026918	1-Dodecanol	Dodecanol
DTXSID50873428	Dibutyl phenyl phosphate	dibutyl phenylphosphate
DTXSID50911718	N-(Hydroxymethyl)-3-[methoxy(methy	pyrofix 2
DTXSID50931491	2-Bromopropenal	2-bromoacrolein
DTXSID50931491	2-Bromopropenal	2-bromoacrolein
DTXSID50931491	2-Bromopropenal	2-bromoacrolein
DTXSID6020436	Dichlorodifluoromethane	dichlorodifluoromethane
DTXSID6020802	Melamine	melamine
DTXSID6020802	Melamine	melamine
DTXSID6020802	Melamine	melamine
DTXSID6020802	Melamine	melamine
DTXSID6020802	Melamine	melamine
DTXSID6020802	Melamine	melamine
DTXSID6020802	Melamine	melamine
DTXSID6021959	2,4,6-Tribromophenol	2,4,6-tribromophenol
DTXSID6021959	2,4,6-Tribromophenol	2,4,6-tribromophenol
DTXSID6021959	2,4,6-Tribromophenol	2,4,6-tribromophenol
DTXSID6021959	2,4,6-Tribromophenol	2,4,6-tribromophenol
DTXSID6021959	2,4,6-Tribromophenol	2,4,6-tribromophenol
DTXSID6021959	2,4,6-Tribromophenol	2,4,6-tribromophenol
DTXSID6021959	2,4,6-Tribromophenol	2,4,6-tribromophenol
DTXSID6021959	2,4,6-Tribromophenol	2,4,6-tribromophenol
DTXSID6021959	2,4,6-Tribromophenol	2,4,6-tribromophenol
DTXSID6021959	2,4,6-Tribromophenol	2,4,6-tribromophenol

DTXSID6021959	2,4,6-Tribromophenol	2,4,6-tribromophenol
DTXSID60229856	3,5-Dibromo-2-(2,4-dibromophenoxy)ı	3,5-dibromo-2-(2,4-dibromophenoxy)phenol
DTXSID6025010	1,3-Dichloro-2-propanol	1,3-dichloro-2-propanol
DTXSID6026084	4,5,6,7-Tetrabromo-1,3-Isobenzofuran	tetrabromophthalic anhydride
DTXSID6029705	Diammonium hydrogen phosphate	ammonium phosphate
DTXSID6029705	Diammonium hydrogen phosphate	ammonium phosphate
DTXSID6030782	Bentonite	Bentonite
DTXSID6030782	Bentonite	Bentonite
DTXSID6030782	Bentonite	Bentonite
DTXSID6030782	Bentonite	Bentonite
DTXSID6030782	Bentonite	Bentonite
DTXSID6032192	Tri-o-cresyl phosphate	tri-o-cresyl phosphate
DTXSID6032192	Tri-o-cresyl phosphate	tri-o-cresyl phosphate
DTXSID6049694	Policapram (Nylon 6)	nylon 6
DTXSID6049694	Policapram (Nylon 6)	nylon 6
DTXSID6050122	Phosphoric acid, ammonium salt (1:x)	ammonium phosphate
DTXSID6050122	Phosphoric acid, ammonium salt (1:x)	ammonium phosphate
DTXSID6073524	2,3,7,8-Tetrabromodibenzo-p-dioxin	2,3,7,8-tetrabromodibenzo-4-dioxin
DTXSID6074754	1,1'-Biphenyl, 2,3,3',4,4',5,6-heptabror	2,3,3',4,4'5,6-heptabromobiphenyl
DTXSID60894649	Cellulose, acetate butanoate	cellulose acetate-butyrate
DTXSID7020182	Bisphenol A	bisphenol A
DTXSID7020182	Bisphenol A	bisphenol A
DTXSID7020182	Bisphenol A	bisphenol A
DTXSID7020182	Bisphenol A	bisphenol A
DTXSID7020182	Bisphenol A	bisphenol A
DTXSID7020182	Bisphenol A	bisphenol A
DTXSID7020182	Bisphenol A	bisphenol A
DTXSID7020182	Bisphenol A	bisphenol A
DTXSID7020182	Bisphenol A	bisphenol A
DTXSID7020182	Bisphenol A	bisphenol A
DTXSID7020182	Bisphenol A	bisphenol A
DTXSID7020182	Bisphenol A	bisphenol A
DTXSID7020182	Bisphenol A	bisphenol A
DTXSID7020182	Bisphenol A	bisphenol A
DTXSID7020182	Bisphenol A	bisphenol A
DTXSID7020182	Bisphenol A	bisphenol A
DTXSID7020182	Bisphenol A	bisphenol A
DTXSID7020182	Bisphenol A	bisphenol A
DTXSID7020267	Chlordane	Chlordan
DTXSID7020637	Formaldehyde	Formaldehyde
DTXSID7020637	Formaldehyde	Formaldehyde
DTXSID7020637	Formaldehyde	Formaldehyde
DTXSID7020637	Formaldehyde	Formaldehyde
DTXSID7020637	Formaldehyde	Formaldehyde
DTXSID7020970	Nitrofen	nitrofen
DTXSID7021782	2,3,4,5,6-Pentabromoethylbenzene	2,3,4,5,6-pentabromoethylbenzene
DTXSID7021782	2,3,4,5,6-Pentabromoethylbenzene	2,3,4,5,6-pentabromoethylbenzene

DTXSID7021782	2,3,4,5,6-Pentabromoethylbenzene	2,3,4,5,6-pentabromoethylbenzene
DTXSID7021817	2,3-Dibromopropanol	2,3-dibromopropanol
DTXSID7021817	2,3-Dibromopropanol	2,3-dibromopropanol
DTXSID7021817	2,3-Dibromopropanol	2,3-dibromopropanol
DTXSID7021817	2,3-Dibromopropanol	2,3-dibromopropanol
DTXSID7021817	2,3-Dibromopropanol	2,3-dibromopropanol
DTXSID7023805	Tris(2,3-dichloropropyl)phosphate	tris(2,3-dichloropropyl)phosphate
DTXSID7023805	Tris(2,3-dichloropropyl)phosphate	tris(2,3-dichloropropyl)phosphate
DTXSID7023805	Tris(2,3-dichloropropyl)phosphate	tris(2,3-dichloropropyl)phosphate
DTXSID7024873	Cyanuric acid	cyanuric acid
DTXSID7024873	Cyanuric acid	cyanuric acid
DTXSID7025180	4,4'-Diphenylmethane diisocyanate	4,4'-diphenylmethane diisocyanate
DTXSID70275889	Bis(2,3-dibromopropyl) hydrogen phos	bis(2,3-dibromopropyl)phosphate
DTXSID70275889	Bis(2,3-dibromopropyl) hydrogen phos	bis(2,3-dibromopropyl)phosphate
DTXSID70275889	Bis(2,3-dibromopropyl) hydrogen phos	bis(2,3-dibromopropyl)phosphate
DTXSID7027750	Dechlorane Plus	dechlorane plus
DTXSID7027750	Dechlorane Plus	dechlorane plus
DTXSID7027750	Dechlorane Plus	dechlorane plus
DTXSID7027750	Dechlorane Plus	dechlorane plus
DTXSID7027750	Dechlorane Plus	dechlorane plus
DTXSID7027750	Dechlorane Plus	dechlorane plus
DTXSID7027750	Dechlorane Plus	dechlorane plus
DTXSID7027750	Dechlorane Plus	dechlorane plus
DTXSID7027750	Dechlorane Plus	dechlorane plus
DTXSID7027750	Dechlorane Plus	dechlorane plus
DTXSID7027750	Dechlorane Plus	dechlorane plus
DTXSID7027750	Dechlorane Plus	dechlorane plus
DTXSID7027750	Dechlorane Plus	dechlorane plus
DTXSID7027750	Dechlorane Plus	dechlorane plus
DTXSID7027750	Dechlorane Plus	dechlorane plus
DTXSID7027750	Dechlorane Plus	dechlorane plus
DTXSID7027750	Dechlorane Plus	dechlorane plus
DTXSID7027750	Dechlorane Plus	dechlorane plus
DTXSID7027750	Dechlorane Plus	dechlorane plus
DTXSID7027750	Dechlorane Plus	dechlorane plus
DTXSID7027750	Dechlorane Plus	dechlorane plus
DTXSID7027750	Dechlorane Plus	dechlorane plus
DTXSID7027750	Dechlorane Plus	dechlorane plus
DTXSID7027750	Dechlorane Plus	dechlorane plus
DTXSID7027750	Dechlorane Plus	dechlorane plus
DTXSID7027750	Dechlorane Plus	dechlorane plus
DTXSID7027750	Dechlorane Plus	dechlorane plus
DTXSID7027750	Dechlorane Plus	dechlorane plus
DTXSID7027750	Dechlorane Plus	dechlorane plus
DTXSID7027750	Dechlorane Plus	dechlorane plus
DTXSID7027750	Dechlorane Plus	dechlorane plus

DTXSID7027750	Dechlorane Plus	dechlorane plus
DTXSID7027750	Dechlorane Plus	dechlorane plus
DTXSID7027750	Dechlorane Plus	dechlorane plus
DTXSID7027750	Dechlorane Plus	dechlorane plus
DTXSID7027750	Dechlorane Plus	dechlorane plus
DTXSID7027750	Dechlorane Plus	dechlorane plus
DTXSID7027750	Dechlorane Plus	dechlorane plus
DTXSID7027750	Dechlorane Plus	dechlorane plus
DTXSID7027750	Dechlorane Plus	dechlorane plus
DTXSID7027750	Dechlorane Plus	dechlorane plus
DTXSID7027750	Dechlorane Plus	dechlorane plus
DTXSID7027750	Dechlorane Plus	dechlorane plus
DTXSID7027750	Dechlorane Plus	dechlorane plus
DTXSID7027750	Dechlorane Plus	dechlorane plus
DTXSID7027750	Dechlorane Plus	dechlorane plus
DTXSID7027750	Dechlorane Plus	dechlorane plus
DTXSID7027750	Dechlorane Plus	dechlorane plus
DTXSID7027750	Dechlorane Plus	dechlorane plus
DTXSID7027750	Dechlorane Plus	dechlorane plus
DTXSID7027750	Dechlorane Plus	dechlorane plus
DTXSID7027750	Dechlorane Plus	dechlorane plus
DTXSID7027750	Dechlorane Plus	dechlorane plus
DTXSID7027750	Dechlorane Plus	dechlorane plus
DTXSID7027750	Dechlorane Plus	dechlorane plus
DTXSID7027750	Dechlorane Plus	dechlorane plus
DTXSID7027750	Dechlorane Plus	dechlorane plus
DTXSID7027750	Dechlorane Plus	dechlorane plus
DTXSID7027750	Dechlorane Plus	dechlorane plus
DTXSID7027750	Dechlorane Plus	dechlorane plus
DTXSID7027750	Dechlorane Plus	dechlorane plus
DTXSID7027750	Dechlorane Plus	dechlorane plus
DTXSID7027750	Dechlorane Plus	dechlorane plus
DTXSID7027750	Dechlorane Plus	dechlorane plus
DTXSID7027750	Dechlorane Plus	dechlorane plus
DTXSID7027750	Dechlorane Plus	dechlorane plus
DTXSID7027750	Dechlorane Plus	dechlorane plus
DTXSID7027750	Dechlorane Plus	dechlorane plus
DTXSID7027750	Dechlorane Plus	dechlorane plus
DTXSID7027750	Dechlorane Plus	dechlorane plus
DTXSID7027750	Dechlorane Plus	dechlorane plus
DTXSID7027750	Dechlorane Plus	dechlorane plus
DTXSID7027750	Dechlorane Plus	dechlorane plus
DTXSID7027750	Dechlorane Plus	dechlorane plus
DTXSID7027750	Dechlorane Plus	dechlorane plus
DTXSID7027750	Dechlorane Plus	dechlorane plus
DTXSID7027750	Dechlorane Plus	dechlorane plus
DTXSID7027750	Dechlorane Plus	dechlorane plus

DTXSID7027750	Dechlorane Plus	dechlorane plus
DTXSID7027750	Dechlorane Plus	dechlorane plus
DTXSID7027750	Dechlorane Plus	dechlorane plus
DTXSID7027750	Dechlorane Plus	dechlorane plus
DTXSID7027750	Dechlorane Plus	dechlorane plus
DTXSID7027750	Dechlorane Plus	dechlorane plus
DTXSID7027750	Dechlorane Plus	dechlorane plus
DTXSID7027750	Dechlorane Plus	dechlorane plus
DTXSID7027750	Dechlorane Plus	dechlorane plus
DTXSID7027750	Dechlorane Plus	dechlorane plus
DTXSID7027750	Dechlorane Plus	dechlorane plus
DTXSID7027750	Dechlorane Plus	dechlorane plus
DTXSID7027750	Dechlorane Plus	dechlorane plus
DTXSID7027750	Dechlorane Plus	dechlorane plus
DTXSID7027750	Dechlorane Plus	dechlorane plus
DTXSID7035016	Zinc oxide	Zinc Oxide
DTXSID7035016	Zinc oxide	Zinc Oxide
DTXSID7047724	Polytetrafluoroethylene	Polytetrafluoroethylene
DTXSID7060910	1,2,4,5-Tetrabromobenzene	1,2,4,5-tetrabromobenzene
DTXSID7060910	1,2,4,5-Tetrabromobenzene	1,2,4,5-tetrabromobenzene
DTXSID70858838	2,2',4,4',5,5'-Hexabromobiphenyl	2,4,5,2',4',5'-hexabromobiphenyl
DTXSID70858838	2,2',4,4',5,5'-Hexabromobiphenyl	2,4,5,2',4',5'-hexabromobiphenyl
DTXSID70858838	2,2',4,4',5,5'-Hexabromobiphenyl	2,4,5,2',4',5'-hexabromobiphenyl
DTXSID70858838	2,2',4,4',5,5'-Hexabromobiphenyl	2,4,5,2',4',5'-hexabromobiphenyl
DTXSID70858838	2,2',4,4',5,5'-Hexabromobiphenyl	2,4,5,2',4',5'-hexabromobiphenyl
DTXSID70858838	2,2',4,4',5,5'-Hexabromobiphenyl	2,4,5,2',4',5'-hexabromobiphenyl
DTXSID70858838	2,2',4,4',5,5'-Hexabromobiphenyl	2,4,5,2',4',5'-hexabromobiphenyl
DTXSID70858838	2,2',4,4',5,5'-Hexabromobiphenyl	2,4,5,2',4',5'-hexabromobiphenyl
DTXSID70858838	2,2',4,4',5,5'-Hexabromobiphenyl	2,4,5,2',4',5'-hexabromobiphenyl
DTXSID70880073	Firemaster 550	Firemaster 550
DTXSID70880073	Firemaster 550	Firemaster 550
DTXSID70880073	Firemaster 550	Firemaster 550
DTXSID70880073	Firemaster 550	Firemaster 550
DTXSID70983452	Sodium bis(2,3-dibromopropyl) phospl	bis(2,3-dibromopropyl)phosphate
DTXSID70983452	Sodium bis(2,3-dibromopropyl) phospl	bis(2,3-dibromopropyl)phosphate
DTXSID70983452	Sodium bis(2,3-dibromopropyl) phospl	bis(2,3-dibromopropyl)phosphate
DTXSID801017084	polychlorodibenzo-4-dioxin	Polychlorinated Dibenzodioxins
DTXSID801017084	polychlorodibenzo-4-dioxin	Polychlorinated Dibenzodioxins
DTXSID801017084	polychlorodibenzo-4-dioxin	Polychlorinated Dibenzodioxins
DTXSID801017084	polychlorodibenzo-4-dioxin	Polychlorinated Dibenzodioxins
DTXSID801017084	polychlorodibenzo-4-dioxin	Polychlorinated Dibenzodioxins
DTXSID801017084	polychlorodibenzo-4-dioxin	Polychlorinated Dibenzodioxins
DTXSID801017084	polychlorodibenzo-4-dioxin	Polychlorinated Dibenzodioxins
DTXSID801017084	polychlorodibenzo-4-dioxin	Polychlorinated Dibenzodioxins
DTXSID801017084	polychlorodibenzo-4-dioxin	Polychlorinated Dibenzodioxins
DTXSID801017084	polychlorodibenzo-4-dioxin	Polychlorinated Dibenzodioxins
DTXSID801017084	polychlorodibenzo-4-dioxin	Polychlorinated Dibenzodioxins

DTXSID801017084	polychlorodibenzo-4-dioxin	Polychlorinated Dibenzodioxins
DTXSID8020410	Dibenzo-p-dioxin	dibenzo(1,4)dioxin
DTXSID8020410	Dibenzo-p-dioxin	dibenzo(1,4)dioxin
DTXSID8020410	Dibenzo-p-dioxin	dibenzo(1,4)dioxin
DTXSID8020410	Dibenzo-p-dioxin	dibenzo(1,4)dioxin
DTXSID8020410	Dibenzo-p-dioxin	dibenzo(1,4)dioxin
DTXSID8021432	Vinyl bromide	vinyl bromide
DTXSID8024947	1,2-Dibromo-4-(1,2-dibromoethyl)cycl	1,2-dibromo-4-(1,2-dibromoethyl)cyclohexane
DTXSID8024947	1,2-Dibromo-4-(1,2-dibromoethyl)cycl	1,2-dibromo-4-(1,2-dibromoethyl)cyclohexane
DTXSID8024947	1,2-Dibromo-4-(1,2-dibromoethyl)cycl	1,2-dibromo-4-(1,2-dibromoethyl)cyclohexane
DTXSID8024947	1,2-Dibromo-4-(1,2-dibromoethyl)cycl	1,2-dibromo-4-(1,2-dibromoethyl)cyclohexane
DTXSID8024947	1,2-Dibromo-4-(1,2-dibromoethyl)cycl	1,2-dibromo-4-(1,2-dibromoethyl)cyclohexane
DTXSID8024947	1,2-Dibromo-4-(1,2-dibromoethyl)cycl	1,2-dibromo-4-(1,2-dibromoethyl)cyclohexane
DTXSID8024947	1,2-Dibromo-4-(1,2-dibromoethyl)cycl	1,2-dibromo-4-(1,2-dibromoethyl)cyclohexane
DTXSID8024947	1,2-Dibromo-4-(1,2-dibromoethyl)cycl	1,2-dibromo-4-(1,2-dibromoethyl)cyclohexane
DTXSID8024947	1,2-Dibromo-4-(1,2-dibromoethyl)cycl	1,2-dibromo-4-(1,2-dibromoethyl)cyclohexane
DTXSID8024947	1,2-Dibromo-4-(1,2-dibromoethyl)cycl	1,2-dibromo-4-(1,2-dibromoethyl)cyclohexane
DTXSID8024947	1,2-Dibromo-4-(1,2-dibromoethyl)cycl	1,2-dibromo-4-(1,2-dibromoethyl)cyclohexane
DTXSID8024947	1,2-Dibromo-4-(1,2-dibromoethyl)cycl	1,2-dibromo-4-(1,2-dibromoethyl)cyclohexane
DTXSID8024947	1,2-Dibromo-4-(1,2-dibromoethyl)cycl	1,2-dibromo-4-(1,2-dibromoethyl)cyclohexane
DTXSID8024947	1,2-Dibromo-4-(1,2-dibromoethyl)cycl	1,2-dibromo-4-(1,2-dibromoethyl)cyclohexane
DTXSID8024947	1,2-Dibromo-4-(1,2-dibromoethyl)cycl	1,2-dibromo-4-(1,2-dibromoethyl)cyclohexane
DTXSID8024947	1,2-Dibromo-4-(1,2-dibromoethyl)cycl	1,2-dibromo-4-(1,2-dibromoethyl)cyclohexane
DTXSID8024947	1,2-Dibromo-4-(1,2-dibromoethyl)cycl	1,2-dibromo-4-(1,2-dibromoethyl)cyclohexane
DTXSID8024947	1,2-Dibromo-4-(1,2-dibromoethyl)cycl	1,2-dibromo-4-(1,2-dibromoethyl)cyclohexane
DTXSID8024947	1,2-Dibromo-4-(1,2-dibromoethyl)cycl	1,2-dibromo-4-(1,2-dibromoethyl)cyclohexane
DTXSID8024947	1,2-Dibromo-4-(1,2-dibromoethyl)cycl	1,2-dibromo-4-(1,2-dibromoethyl)cyclohexane
DTXSID8025383	Hexabromocyclododecane	hexabromocyclododecane
DTXSID8025383	Hexabromocyclododecane	hexabromocyclododecane
DTXSID8025383	Hexabromocyclododecane	hexabromocyclododecane
DTXSID8025383	Hexabromocyclododecane	hexabromocyclododecane
DTXSID8025383	Hexabromocyclododecane	hexabromocyclododecane
DTXSID8025383	Hexabromocyclododecane	hexabromocyclododecane
DTXSID8025383	Hexabromocyclododecane	hexabromocyclododecane
DTXSID8025383	Hexabromocyclododecane	hexabromocyclododecane
DTXSID8025383	Hexabromocyclododecane	hexabromocyclododecane
DTXSID8025383	Hexabromocyclododecane	hexabromocyclododecane
DTXSID8025383	Hexabromocyclododecane	hexabromocyclododecane
DTXSID8025383	Hexabromocyclododecane	hexabromocyclododecane
DTXSID8025383	Hexabromocyclododecane	hexabromocyclododecane
DTXSID8025383	Hexabromocyclododecane	hexabromocyclododecane
DTXSID8025383	Hexabromocyclododecane	hexabromocyclododecane
DTXSID8025383	Hexabromocyclododecane	hexabromocyclododecane
DTXSID8025383	Hexabromocyclododecane	hexabromocyclododecane
DTXSID8025383	Hexabromocyclododecane	hexabromocyclododecane
DTXSID8025383	Hexabromocyclododecane	hexabromocyclododecane

DTXSID8025383	Hexabromocyclododecane	hexabromocyclododecane
DTXSID8025383	Hexabromocyclododecane	hexabromocyclododecane
DTXSID8025383	Hexabromocyclododecane	hexabromocyclododecane
DTXSID8025383	Hexabromocyclododecane	hexabromocyclododecane
DTXSID8025383	Hexabromocyclododecane	hexabromocyclododecane
DTXSID8025383	Hexabromocyclododecane	hexabromocyclododecane
DTXSID8025383	Hexabromocyclododecane	hexabromocyclododecane
DTXSID8025383	Hexabromocyclododecane	hexabromocyclododecane
DTXSID8025383	Hexabromocyclododecane	hexabromocyclododecane
DTXSID8025383	Hexabromocyclododecane	hexabromocyclododecane
DTXSID8025383	Hexabromocyclododecane	hexabromocyclododecane
DTXSID8025383	Hexabromocyclododecane	hexabromocyclododecane
DTXSID8025383	Hexabromocyclododecane	hexabromocyclododecane
DTXSID8025383	Hexabromocyclododecane	hexabromocyclododecane
DTXSID8025383	Hexabromocyclododecane	hexabromocyclododecane
DTXSID8025383	Hexabromocyclododecane	hexabromocyclododecane
DTXSID8025383	Hexabromocyclododecane	hexabromocyclododecane
DTXSID8025383	Hexabromocyclododecane	hexabromocyclododecane
DTXSID8025383	Hexabromocyclododecane	hexabromocyclododecane
DTXSID8025383	Hexabromocyclododecane	hexabromocyclododecane
DTXSID8025383	Hexabromocyclododecane	hexabromocyclododecane
DTXSID8025383	Hexabromocyclododecane	hexabromocyclododecane
DTXSID8025383	Hexabromocyclododecane	hexabromocyclododecane
DTXSID8025383	Hexabromocyclododecane	hexabromocyclododecane
DTXSID8025383	Hexabromocyclododecane	hexabromocyclododecane
DTXSID8025383	Hexabromocyclododecane	hexabromocyclododecane
DTXSID8025383	Hexabromocyclododecane	hexabromocyclododecane
DTXSID8025383	Hexabromocyclododecane	hexabromocyclododecane
DTXSID8025383	Hexabromocyclododecane	hexabromocyclododecane
DTXSID8025383	Hexabromocyclododecane	hexabromocyclododecane
DTXSID8025383	Hexabromocyclododecane	hexabromocyclododecane
DTXSID8025383	Hexabromocyclododecane	hexabromocyclododecane
DTXSID8025383	Hexabromocyclododecane	hexabromocyclododecane
DTXSID8025383	Hexabromocyclododecane	hexabromocyclododecane
DTXSID8025383	Hexabromocyclododecane	hexabromocyclododecane
DTXSID8025383	Hexabromocyclododecane	hexabromocyclododecane
DTXSID8025383	Hexabromocyclododecane	hexabromocyclododecane
DTXSID8025383	Hexabromocyclododecane	hexabromocyclododecane
DTXSID8025383	Hexabromocyclododecane	hexabromocyclododecane
DTXSID8025383	Hexabromocyclododecane	hexabromocyclododecane
DTXSID8025383	Hexabromocyclododecane	hexabromocyclododecane
DTXSID8025383	Hexabromocyclododecane	hexabromocyclododecane
DTXSID8025383	Hexabromocyclododecane	hexabromocyclododecane
DTXSID8025383	Hexabromocyclododecane	hexabromocyclododecane
DTXSID8025383	Hexabromocyclododecane	hexabromocyclododecane
DTXSID8025383	Hexabromocyclododecane	hexabromocyclododecane
DTXSID8025383	Hexabromocyclododecane	hexabromocyclododecane

DTXSID8025383	Hexabromocyclododecane	hexabromocyclododecane
DTXSID8025383	Hexabromocyclododecane	hexabromocyclododecane
DTXSID8025383	Hexabromocyclododecane	hexabromocyclododecane
DTXSID8025383	Hexabromocyclododecane	hexabromocyclododecane
DTXSID8025383	Hexabromocyclododecane	hexabromocyclododecane
DTXSID8025383	Hexabromocyclododecane	hexabromocyclododecane
DTXSID8025383	Hexabromocyclododecane	hexabromocyclododecane
DTXSID8025383	Hexabromocyclododecane	hexabromocyclododecane
DTXSID8025383	Hexabromocyclododecane	hexabromocyclododecane
DTXSID8025383	Hexabromocyclododecane	hexabromocyclododecane
DTXSID8025383	Hexabromocyclododecane	hexabromocyclododecane
DTXSID8025383	Hexabromocyclododecane	hexabromocyclododecane
DTXSID8025383	Hexabromocyclododecane	hexabromocyclododecane
DTXSID8025383	Hexabromocyclododecane	hexabromocyclododecane
DTXSID8025383	Hexabromocyclododecane	hexabromocyclododecane
DTXSID8025383	Hexabromocyclododecane	hexabromocyclododecane
DTXSID8025383	Hexabromocyclododecane	hexabromocyclododecane
DTXSID8025383	Hexabromocyclododecane	hexabromocyclododecane
DTXSID8025383	Hexabromocyclododecane	hexabromocyclododecane
DTXSID8025383	Hexabromocyclododecane	hexabromocyclododecane
DTXSID8025383	Hexabromocyclododecane	hexabromocyclododecane
DTXSID8025383	Hexabromocyclododecane	hexabromocyclododecane
DTXSID8025383	Hexabromocyclododecane	hexabromocyclododecane
DTXSID8025383	Hexabromocyclododecane	hexabromocyclododecane
DTXSID8025383	Hexabromocyclododecane	hexabromocyclododecane
DTXSID8025383	Hexabromocyclododecane	hexabromocyclododecane
DTXSID8025383	Hexabromocyclododecane	hexabromocyclododecane
DTXSID8025383	Hexabromocyclododecane	hexabromocyclododecane
DTXSID8025383	Hexabromocyclododecane	hexabromocyclododecane
DTXSID8025383	Hexabromocyclododecane	hexabromocyclododecane
DTXSID8025383	Hexabromocyclododecane	hexabromocyclododecane
DTXSID8025383	Hexabromocyclododecane	hexabromocyclododecane
DTXSID8025383	Hexabromocyclododecane	hexabromocyclododecane
DTXSID8025383	Hexabromocyclododecane	hexabromocyclododecane
DTXSID8025383	Hexabromocyclododecane	hexabromocyclododecane
DTXSID8025383	Hexabromocyclododecane	hexabromocyclododecane
DTXSID8025383	Hexabromocyclododecane	hexabromocyclododecane
DTXSID8025383	Hexabromocyclododecane	hexabromocyclododecane
DTXSID8025383	Hexabromocyclododecane	hexabromocyclododecane
DTXSID8025383	Hexabromocyclododecane	hexabromocyclododecane
DTXSID8025383	Hexabromocyclododecane	hexabromocyclododecane
DTXSID8025383	Hexabromocyclododecane	hexabromocyclododecane
DTXSID8025383	Hexabromocyclododecane	hexabromocyclododecane
DTXSID8025383	Hexabromocyclododecane	hexabromocyclododecane
DTXSID8025383	Hexabromocyclododecane	hexabromocyclododecane
DTXSID8025383	Hexabromocyclododecane	hexabromocyclododecane
DTXSID8025383	Hexabromocyclododecane	hexabromocyclododecane

DTXSID8025383	Hexabromocyclododecane	hexabromocyclododecane
DTXSID8025383	Hexabromocyclododecane	hexabromocyclododecane
DTXSID8025383	Hexabromocyclododecane	hexabromocyclododecane
DTXSID8025383	Hexabromocyclododecane	hexabromocyclododecane
DTXSID8025383	Hexabromocyclododecane	hexabromocyclododecane
DTXSID8025383	Hexabromocyclododecane	hexabromocyclododecane
DTXSID8025383	Hexabromocyclododecane	hexabromocyclododecane
DTXSID8025383	Hexabromocyclododecane	hexabromocyclododecane
DTXSID8025383	Hexabromocyclododecane	hexabromocyclododecane
DTXSID8025383	Hexabromocyclododecane	hexabromocyclododecane
DTXSID8025383	Hexabromocyclododecane	hexabromocyclododecane
DTXSID8025383	Hexabromocyclododecane	hexabromocyclododecane
DTXSID8025383	Hexabromocyclododecane	hexabromocyclododecane
DTXSID8025383	Hexabromocyclododecane	hexabromocyclododecane
DTXSID8025383	Hexabromocyclododecane	hexabromocyclododecane
DTXSID8025383	Hexabromocyclododecane	hexabromocyclododecane
DTXSID8025383	Hexabromocyclododecane	hexabromocyclododecane
DTXSID8025383	Hexabromocyclododecane	hexabromocyclododecane
DTXSID8025383	Hexabromocyclododecane	hexabromocyclododecane
DTXSID8025383	Hexabromocyclododecane	hexabromocyclododecane
DTXSID8025383	Hexabromocyclododecane	hexabromocyclododecane
DTXSID8025383	Hexabromocyclododecane	hexabromocyclododecane
DTXSID8025383	Hexabromocyclododecane	hexabromocyclododecane
DTXSID8025383	Hexabromocyclododecane	hexabromocyclododecane
DTXSID8025383	Hexabromocyclododecane	hexabromocyclododecane
DTXSID8025383	Hexabromocyclododecane	hexabromocyclododecane
DTXSID8025383	Hexabromocyclododecane	hexabromocyclododecane
DTXSID8025383	Hexabromocyclododecane	hexabromocyclododecane
DTXSID8025383	Hexabromocyclododecane	hexabromocyclododecane
DTXSID8025383	Hexabromocyclododecane	hexabromocyclododecane
DTXSID8025383	Hexabromocyclododecane	hexabromocyclododecane
DTXSID8025383	Hexabromocyclododecane	hexabromocyclododecane
DTXSID8025383	Hexabromocyclododecane	hexabromocyclododecane
DTXSID8025383	Hexabromocyclododecane	hexabromocyclododecane
DTXSID8025383	Hexabromocyclododecane	hexabromocyclododecane
DTXSID8025383	Hexabromocyclododecane	hexabromocyclododecane
DTXSID8025383	Hexabromocyclododecane	hexabromocyclododecane
DTXSID8025383	Hexabromocyclododecane	hexabromocyclododecane
DTXSID8025383	Hexabromocyclododecane	hexabromocyclododecane
DTXSID8025383	Hexabromocyclododecane	hexabromocyclododecane
DTXSID8025383	Hexabromocyclododecane	hexabromocyclododecane
DTXSID8025383	Hexabromocyclododecane	hexabromocyclododecane
DTXSID8025383	Hexabromocyclododecane	hexabromocyclododecane
DTXSID8025383	Hexabromocyclododecane	hexabromocyclododecane
DTXSID8025383	Hexabromocyclododecane	hexabromocyclododecane
DTXSID8025383	Hexabromocyclododecane	hexabromocyclododecane
DTXSID8025383	Hexabromocyclododecane	hexabromocyclododecane

DTXSID8025383	Hexabromocyclododecane	hexabromocyclododecane
DTXSID8025383	Hexabromocyclododecane	hexabromocyclododecane
DTXSID8025383	Hexabromocyclododecane	hexabromocyclododecane
DTXSID8025383	Hexabromocyclododecane	hexabromocyclododecane
DTXSID8025383	Hexabromocyclododecane	hexabromocyclododecane
DTXSID8025383	Hexabromocyclododecane	hexabromocyclododecane
DTXSID8025383	Hexabromocyclododecane	hexabromocyclododecane
DTXSID8025383	Hexabromocyclododecane	hexabromocyclododecane
DTXSID8025383	Hexabromocyclododecane	hexabromocyclododecane
DTXSID8025383	Hexabromocyclododecane	hexabromocyclododecane
DTXSID8025383	Hexabromocyclododecane	hexabromocyclododecane
DTXSID8025383	Hexabromocyclododecane	hexabromocyclododecane
DTXSID8025383	Hexabromocyclododecane	hexabromocyclododecane
DTXSID8025383	Hexabromocyclododecane	hexabromocyclododecane
DTXSID8025383	Hexabromocyclododecane	hexabromocyclododecane
DTXSID8025383	Hexabromocyclododecane	hexabromocyclododecane
DTXSID8025383	Hexabromocyclododecane	hexabromocyclododecane
DTXSID8025383	Hexabromocyclododecane	hexabromocyclododecane
DTXSID8025383	Hexabromocyclododecane	hexabromocyclododecane
DTXSID8025383	Hexabromocyclododecane	hexabromocyclododecane
DTXSID8025383	Hexabromocyclododecane	hexabromocyclododecane
DTXSID8025383	Hexabromocyclododecane	hexabromocyclododecane
DTXSID8025383	Hexabromocyclododecane	hexabromocyclododecane
DTXSID8025383	Hexabromocyclododecane	hexabromocyclododecane
DTXSID8025383	Hexabromocyclododecane	hexabromocyclododecane
DTXSID8025383	Hexabromocyclododecane	hexabromocyclododecane
DTXSID8025383	Hexabromocyclododecane	hexabromocyclododecane
DTXSID8025383	Hexabromocyclododecane	hexabromocyclododecane
DTXSID8025383	Hexabromocyclododecane	hexabromocyclododecane
DTXSID8025383	Hexabromocyclododecane	hexabromocyclododecane
DTXSID8025383	Hexabromocyclododecane	hexabromocyclododecane
DTXSID8025383	Hexabromocyclododecane	hexabromocyclododecane
DTXSID8025383	Hexabromocyclododecane	hexabromocyclododecane
DTXSID8025383	Hexabromocyclododecane	hexabromocyclododecane
DTXSID8025383	Hexabromocyclododecane	hexabromocyclododecane
DTXSID8025383	Hexabromocyclododecane	hexabromocyclododecane
DTXSID8025383	Hexabromocyclododecane	hexabromocyclododecane
DTXSID8025383	Hexabromocyclododecane	hexabromocyclododecane
DTXSID8025383	Hexabromocyclododecane	hexabromocyclododecane
DTXSID8025383	Hexabromocyclododecane	hexabromocyclododecane
DTXSID8025383	Hexabromocyclododecane	hexabromocyclododecane
DTXSID8025383	Hexabromocyclododecane	hexabromocyclododecane
DTXSID8025383	Hexabromocyclododecane	hexabromocyclododecane
DTXSID8025383	Hexabromocyclododecane	hexabromocyclododecane
DTXSID8025383	Hexabromocyclododecane	hexabromocyclododecane
DTXSID8025383	Hexabromocyclododecane	hexabromocyclododecane
DTXSID8025383	Hexabromocyclododecane	hexabromocyclododecane

DTXSID8025383	Hexabromocyclododecane	hexabromocyclododecane
DTXSID8025383	Hexabromocyclododecane	hexabromocyclododecane
DTXSID8025383	Hexabromocyclododecane	hexabromocyclododecane
DTXSID8025383	Hexabromocyclododecane	hexabromocyclododecane
DTXSID8025383	Hexabromocyclododecane	hexabromocyclododecane
DTXSID8025383	Hexabromocyclododecane	hexabromocyclododecane
DTXSID8025383	Hexabromocyclododecane	hexabromocyclododecane
DTXSID8025383	Hexabromocyclododecane	hexabromocyclododecane
DTXSID8025383	Hexabromocyclododecane	hexabromocyclododecane
DTXSID8025383	Hexabromocyclododecane	hexabromocyclododecane
DTXSID8025383	Hexabromocyclododecane	hexabromocyclododecane
DTXSID8025383	Hexabromocyclododecane	hexabromocyclododecane
DTXSID8025383	Hexabromocyclododecane	hexabromocyclododecane
DTXSID8025383	Hexabromocyclododecane	hexabromocyclododecane
DTXSID8025383	Hexabromocyclododecane	hexabromocyclododecane
DTXSID8025383	Hexabromocyclododecane	hexabromocyclododecane
DTXSID8025383	Hexabromocyclododecane	hexabromocyclododecane
DTXSID8025383	Hexabromocyclododecane	hexabromocyclododecane
DTXSID8025383	Hexabromocyclododecane	hexabromocyclododecane
DTXSID8025383	Hexabromocyclododecane	hexabromocyclododecane
DTXSID8025383	Hexabromocyclododecane	hexabromocyclododecane
DTXSID8025383	Hexabromocyclododecane	hexabromocyclododecane
DTXSID8025383	Hexabromocyclododecane	hexabromocyclododecane
DTXSID8025383	Hexabromocyclododecane	hexabromocyclododecane
DTXSID8025383	Hexabromocyclododecane	hexabromocyclododecane
DTXSID8025383	Hexabromocyclododecane	hexabromocyclododecane
DTXSID8025383	Hexabromocyclododecane	hexabromocyclododecane
DTXSID8025383	Hexabromocyclododecane	hexabromocyclododecane
DTXSID8025383	Hexabromocyclododecane	hexabromocyclododecane
DTXSID8025383	Hexabromocyclododecane	hexabromocyclododecane
DTXSID8025383	Hexabromocyclododecane	hexabromocyclododecane
DTXSID8025383	Hexabromocyclododecane	hexabromocyclododecane
DTXSID8025383	Hexabromocyclododecane	hexabromocyclododecane
DTXSID8025383	Hexabromocyclododecane	hexabromocyclododecane
DTXSID8025383	Hexabromocyclododecane	hexabromocyclododecane
DTXSID8025383	Hexabromocyclododecane	hexabromocyclododecane
DTXSID8025383	Hexabromocyclododecane	hexabromocyclododecane
DTXSID8025383	Hexabromocyclododecane	hexabromocyclododecane
DTXSID8025383	Hexabromocyclododecane	hexabromocyclododecane
DTXSID8025383	Hexabromocyclododecane	hexabromocyclododecane
DTXSID8025383	Hexabromocyclododecane	hexabromocyclododecane
DTXSID8025383	Hexabromocyclododecane	hexabromocyclododecane
DTXSID8025383	Hexabromocyclododecane	hexabromocyclododecane
DTXSID8025383	Hexabromocyclododecane	hexabromocyclododecane
DTXSID8025383	Hexabromocyclododecane	hexabromocyclododecane
DTXSID8025383	Hexabromocyclododecane	hexabromocyclododecane
DTXSID8025383	Hexabromocyclododecane	hexabromocyclododecane

DTXSID8025383	Hexabromocyclododecane	hexabromocyclododecane
DTXSID8025383	Hexabromocyclododecane	hexabromocyclododecane
DTXSID8025383	Hexabromocyclododecane	hexabromocyclododecane
DTXSID8025383	Hexabromocyclododecane	hexabromocyclododecane
DTXSID8025383	Hexabromocyclododecane	hexabromocyclododecane
DTXSID8025383	Hexabromocyclododecane	hexabromocyclododecane
DTXSID8025383	Hexabromocyclododecane	hexabromocyclododecane
DTXSID8025383	Hexabromocyclododecane	hexabromocyclododecane
DTXSID8025383	Hexabromocyclododecane	hexabromocyclododecane
DTXSID8025383	Hexabromocyclododecane	hexabromocyclododecane
DTXSID8025383	Hexabromocyclododecane	hexabromocyclododecane
DTXSID8025383	Hexabromocyclododecane	hexabromocyclododecane
DTXSID8025383	Hexabromocyclododecane	hexabromocyclododecane
DTXSID8025383	Hexabromocyclododecane	hexabromocyclododecane
DTXSID8025383	Hexabromocyclododecane	hexabromocyclododecane
DTXSID8025383	Hexabromocyclododecane	hexabromocyclododecane
DTXSID8025383	Hexabromocyclododecane	hexabromocyclododecane
DTXSID8025383	Hexabromocyclododecane	hexabromocyclododecane
DTXSID8025383	Hexabromocyclododecane	hexabromocyclododecane
DTXSID8025383	Hexabromocyclododecane	hexabromocyclododecane
DTXSID8025383	Hexabromocyclododecane	hexabromocyclododecane
DTXSID8025383	Hexabromocyclododecane	hexabromocyclododecane
DTXSID8025383	Hexabromocyclododecane	hexabromocyclododecane
DTXSID8025383	Hexabromocyclododecane	hexabromocyclododecane
DTXSID8025383	Hexabromocyclododecane	hexabromocyclododecane
DTXSID8025383	Hexabromocyclododecane	hexabromocyclododecane
DTXSID8025383	Hexabromocyclododecane	hexabromocyclododecane
DTXSID8025383	Hexabromocyclododecane	hexabromocyclododecane
DTXSID8025383	Hexabromocyclododecane	hexabromocyclododecane
DTXSID8026228	Triethyl phosphate	triethyl phosphate
DTXSID8026228	Triethyl phosphate	triethyl phosphate
DTXSID8026228	Triethyl phosphate	triethyl phosphate
DTXSID8026228	Triethyl phosphate	triethyl phosphate
DTXSID8026228	Triethyl phosphate	triethyl phosphate
DTXSID8026228	Triethyl phosphate	triethyl phosphate
DTXSID8026228	Triethyl phosphate	triethyl phosphate
DTXSID8026228	Triethyl phosphate	triethyl phosphate
DTXSID8029656	Sulfur fluoride (SF6)	Sulfur Hexafluoride
DTXSID8031865	Perfluorooctanoic acid	perfluorooctanoic acid
DTXSID8031865	Perfluorooctanoic acid	perfluorooctanoic acid
DTXSID8043781	Tetrabromophthalic acid	tetrabromophthalic acid
DTXSID8043781	Tetrabromophthalic acid	tetrabromophthalic acid
DTXSID8043781	Tetrabromophthalic acid	tetrabromophthalic acid
DTXSID8052641	2-Bromophenol	2-bromophenol
DTXSID8052641	2-Bromophenol	2-bromophenol
DTXSID8052641	2-Bromophenol	2-bromophenol
DTXSID8052693	2,2',3,4,4',5',6-Heptabromodiphenyl et	2,2',3,4,4',5',6-heptabromodiphenyl ether

DTXSID8052693	2,2',3,4,4',5',6-Heptabromodiphenyl et	2,2',3,4,4',5',6-heptabromodiphenyl ether
DTXSID8052693	2,2',3,4,4',5',6-Heptabromodiphenyl et	2,2',3,4,4',5',6-heptabromodiphenyl ether
DTXSID8052693	2,2',3,4,4',5',6-Heptabromodiphenyl et	2,2',3,4,4',5',6-heptabromodiphenyl ether
DTXSID8052693	2,2',3,4,4',5',6-Heptabromodiphenyl et	2,2',3,4,4',5',6-heptabromodiphenyl ether
DTXSID8052693	2,2',3,4,4',5',6-Heptabromodiphenyl et	2,2',3,4,4',5',6-heptabromodiphenyl ether
DTXSID8052693	2,2',3,4,4',5',6-Heptabromodiphenyl et	2,2',3,4,4',5',6-heptabromodiphenyl ether
DTXSID8052693	2,2',3,4,4',5',6-Heptabromodiphenyl et	2,2',3,4,4',5',6-heptabromodiphenyl ether
DTXSID8052693	2,2',3,4,4',5',6-Heptabromodiphenyl et	2,2',3,4,4',5',6-heptabromodiphenyl ether
DTXSID8052693	2,2',3,4,4',5',6-Heptabromodiphenyl et	2,2',3,4,4',5',6-heptabromodiphenyl ether
DTXSID8052693	2,2',3,4,4',5',6-Heptabromodiphenyl et	2,2',3,4,4',5',6-heptabromodiphenyl ether
DTXSID901017087	pentabromocyclododecene	pentabromocyclododecene
DTXSID901017087	pentabromocyclododecene	pentabromocyclododecene
DTXSID901017087	pentabromocyclododecene	pentabromocyclododecene
DTXSID901017118	Torflam	torflam
DTXSID9020164	Pentaerythritol dibromide	2,2-bis(bromomethyl)-1,3-propanediol
DTXSID9020164	Pentaerythritol dibromide	2,2-bis(bromomethyl)-1,3-propanediol
DTXSID9020164	Pentaerythritol dibromide	2,2-bis(bromomethyl)-1,3-propanediol
DTXSID9020164	Pentaerythritol dibromide	2,2-bis(bromomethyl)-1,3-propanediol
DTXSID9020164	Pentaerythritol dibromide	2,2-bis(bromomethyl)-1,3-propanediol
DTXSID9020164	Pentaerythritol dibromide	2,2-bis(bromomethyl)-1,3-propanediol
DTXSID9020164	Pentaerythritol dibromide	2,2-bis(bromomethyl)-1,3-propanediol
DTXSID9020164	Pentaerythritol dibromide	2,2-bis(bromomethyl)-1,3-propanediol
DTXSID9020164	Pentaerythritol dibromide	2,2-bis(bromomethyl)-1,3-propanediol
DTXSID9020376	1,1'-Oxybis[2,3,4,5,6-pentabromobenz	decabromobiphenyl ether
DTXSID9020376	1,1'-Oxybis[2,3,4,5,6-pentabromobenz	decabromobiphenyl ether
DTXSID9020376	1,1'-Oxybis[2,3,4,5,6-pentabromobenz	decabromobiphenyl ether
DTXSID9020376	1,1'-Oxybis[2,3,4,5,6-pentabromobenz	decabromobiphenyl ether
DTXSID9020376	1,1'-Oxybis[2,3,4,5,6-pentabromobenz	decabromobiphenyl ether
DTXSID9020376	1,1'-Oxybis[2,3,4,5,6-pentabromobenz	decabromobiphenyl ether
DTXSID9020376	1,1'-Oxybis[2,3,4,5,6-pentabromobenz	decabromobiphenyl ether
DTXSID9020376	1,1'-Oxybis[2,3,4,5,6-pentabromobenz	decabromobiphenyl ether
DTXSID9020376	1,1'-Oxybis[2,3,4,5,6-pentabromobenz	decabromobiphenyl ether
DTXSID9020376	1,1'-Oxybis[2,3,4,5,6-pentabromobenz	decabromobiphenyl ether
DTXSID9020376	1,1'-Oxybis[2,3,4,5,6-pentabromobenz	decabromobiphenyl ether
DTXSID9020376	1,1'-Oxybis[2,3,4,5,6-pentabromobenz	decabromobiphenyl ether
DTXSID9020376	1,1'-Oxybis[2,3,4,5,6-pentabromobenz	decabromobiphenyl ether
DTXSID9020376	1,1'-Oxybis[2,3,4,5,6-pentabromobenz	decabromobiphenyl ether
DTXSID9020376	1,1'-Oxybis[2,3,4,5,6-pentabromobenz	decabromobiphenyl ether
DTXSID9020376	1,1'-Oxybis[2,3,4,5,6-pentabromobenz	decabromobiphenyl ether
DTXSID9020376	1,1'-Oxybis[2,3,4,5,6-pentabromobenz	decabromobiphenyl ether
DTXSID9020376	1,1'-Oxybis[2,3,4,5,6-pentabromobenz	decabromobiphenyl ether
DTXSID9020376	1,1'-Oxybis[2,3,4,5,6-pentabromobenz	decabromobiphenyl ether
DTXSID9020376	1,1'-Oxybis[2,3,4,5,6-pentabromobenz	decabromobiphenyl ether
DTXSID9020376	1,1'-Oxybis[2,3,4,5,6-pentabromobenz	decabromobiphenyl ether
DTXSID9020376	1,1'-Oxybis[2,3,4,5,6-pentabromobenz	decabromobiphenyl ether
DTXSID9020376	1,1'-Oxybis[2,3,4,5,6-pentabromobenz	decabromobiphenyl ether
DTXSID9020376	1,1'-Oxybis[2,3,4,5,6-pentabromobenz	decabromobiphenyl ether

DTXSID9020376	1,1'-Oxybis[2,3,4,5,6-pentabromobenz	decabromobiphenyl ether
DTXSID9020376	1,1'-Oxybis[2,3,4,5,6-pentabromobenz	decabromobiphenyl ether
DTXSID9020376	1,1'-Oxybis[2,3,4,5,6-pentabromobenz	decabromobiphenyl ether
DTXSID9020376	1,1'-Oxybis[2,3,4,5,6-pentabromobenz	decabromobiphenyl ether
DTXSID9020376	1,1'-Oxybis[2,3,4,5,6-pentabromobenz	decabromobiphenyl ether
DTXSID9020376	1,1'-Oxybis[2,3,4,5,6-pentabromobenz	decabromobiphenyl ether
DTXSID9020376	1,1'-Oxybis[2,3,4,5,6-pentabromobenz	decabromobiphenyl ether
DTXSID9020376	1,1'-Oxybis[2,3,4,5,6-pentabromobenz	decabromobiphenyl ether
DTXSID9020376	1,1'-Oxybis[2,3,4,5,6-pentabromobenz	decabromobiphenyl ether
DTXSID9020376	1,1'-Oxybis[2,3,4,5,6-pentabromobenz	decabromobiphenyl ether
DTXSID9020376	1,1'-Oxybis[2,3,4,5,6-pentabromobenz	decabromobiphenyl ether
DTXSID9020376	1,1'-Oxybis[2,3,4,5,6-pentabromobenz	decabromobiphenyl ether
DTXSID9020376	1,1'-Oxybis[2,3,4,5,6-pentabromobenz	decabromobiphenyl ether
DTXSID9020376	1,1'-Oxybis[2,3,4,5,6-pentabromobenz	decabromobiphenyl ether
DTXSID9020376	1,1'-Oxybis[2,3,4,5,6-pentabromobenz	decabromobiphenyl ether
DTXSID9020376	1,1'-Oxybis[2,3,4,5,6-pentabromobenz	decabromobiphenyl ether
DTXSID9020376	1,1'-Oxybis[2,3,4,5,6-pentabromobenz	decabromobiphenyl ether
DTXSID9020376	1,1'-Oxybis[2,3,4,5,6-pentabromobenz	decabromobiphenyl ether
DTXSID9020376	1,1'-Oxybis[2,3,4,5,6-pentabromobenz	decabromobiphenyl ether
DTXSID9020376	1,1'-Oxybis[2,3,4,5,6-pentabromobenz	decabromobiphenyl ether
DTXSID9020376	1,1'-Oxybis[2,3,4,5,6-pentabromobenz	decabromobiphenyl ether
DTXSID9020376	1,1'-Oxybis[2,3,4,5,6-pentabromobenz	decabromobiphenyl ether
DTXSID9020376	1,1'-Oxybis[2,3,4,5,6-pentabromobenz	decabromobiphenyl ether
DTXSID9020376	1,1'-Oxybis[2,3,4,5,6-pentabromobenz	decabromobiphenyl ether
DTXSID9020376	1,1'-Oxybis[2,3,4,5,6-pentabromobenz	decabromobiphenyl ether
DTXSID9020376	1,1'-Oxybis[2,3,4,5,6-pentabromobenz	decabromobiphenyl ether
DTXSID9020376	1,1'-Oxybis[2,3,4,5,6-pentabromobenz	decabromobiphenyl ether
DTXSID9020376	1,1'-Oxybis[2,3,4,5,6-pentabromobenz	decabromobiphenyl ether
DTXSID9020376	1,1'-Oxybis[2,3,4,5,6-pentabromobenz	decabromobiphenyl ether
DTXSID9020376	1,1'-Oxybis[2,3,4,5,6-pentabromobenz	decabromobiphenyl ether
DTXSID9020376	1,1'-Oxybis[2,3,4,5,6-pentabromobenz	decabromobiphenyl ether
DTXSID9020376	1,1'-Oxybis[2,3,4,5,6-pentabromobenz	decabromobiphenyl ether
DTXSID9020376	1,1'-Oxybis[2,3,4,5,6-pentabromobenz	decabromobiphenyl ether
DTXSID9020376	1,1'-Oxybis[2,3,4,5,6-pentabromobenz	decabromobiphenyl ether
DTXSID9020376	1,1'-Oxybis[2,3,4,5,6-pentabromobenz	decabromobiphenyl ether
DTXSID9020376	1,1'-Oxybis[2,3,4,5,6-pentabromobenz	decabromobiphenyl ether
DTXSID9020376	1,1'-Oxybis[2,3,4,5,6-pentabromobenz	decabromobiphenyl ether
DTXSID9020376	1,1'-Oxybis[2,3,4,5,6-pentabromobenz	decabromobiphenyl ether
DTXSID9020376	1,1'-Oxybis[2,3,4,5,6-pentabromobenz	decabromobiphenyl ether
DTXSID9020376	1,1'-Oxybis[2,3,4,5,6-pentabromobenz	decabromobiphenyl ether
DTXSID9020376	1,1'-Oxybis[2,3,4,5,6-pentabromobenz	decabromobiphenyl ether
DTXSID9020376	1,1'-Oxybis[2,3,4,5,6-pentabromobenz	decabromobiphenyl ether
DTXSID9020376	1,1'-Oxybis[2,3,4,5,6-pentabromobenz	decabromobiphenyl ether
DTXSID9020376	1,1'-Oxybis[2,3,4,5,6-pentabromobenz	decabromobiphenyl ether
DTXSID9020376	1,1'-Oxybis[2,3,4,5,6-pentabromobenz	decabromobiphenyl ether
DTXSID9020376	1,1'-Oxybis[2,3,4,5,6-pentabromobenz	decabromobiphenyl ether
DTXSID9020376	1,1'-Oxybis[2,3,4,5,6-pentabromobenz	decabromobiphenyl ether

DTXSID9020376	1,1'-Oxybis[2,3,4,5,6-pentabromobenz	decabromobiphenyl ether
DTXSID9020376	1,1'-Oxybis[2,3,4,5,6-pentabromobenz	decabromobiphenyl ether
DTXSID9020376	1,1'-Oxybis[2,3,4,5,6-pentabromobenz	decabromobiphenyl ether
DTXSID9020376	1,1'-Oxybis[2,3,4,5,6-pentabromobenz	decabromobiphenyl ether
DTXSID9020376	1,1'-Oxybis[2,3,4,5,6-pentabromobenz	decabromobiphenyl ether
DTXSID9020376	1,1'-Oxybis[2,3,4,5,6-pentabromobenz	decabromobiphenyl ether
DTXSID9020376	1,1'-Oxybis[2,3,4,5,6-pentabromobenz	decabromobiphenyl ether
DTXSID9020376	1,1'-Oxybis[2,3,4,5,6-pentabromobenz	decabromobiphenyl ether
DTXSID9020376	1,1'-Oxybis[2,3,4,5,6-pentabromobenz	decabromobiphenyl ether
DTXSID9020376	1,1'-Oxybis[2,3,4,5,6-pentabromobenz	decabromobiphenyl ether
DTXSID9020376	1,1'-Oxybis[2,3,4,5,6-pentabromobenz	decabromobiphenyl ether
DTXSID9020376	1,1'-Oxybis[2,3,4,5,6-pentabromobenz	decabromobiphenyl ether
DTXSID9020376	1,1'-Oxybis[2,3,4,5,6-pentabromobenz	decabromobiphenyl ether
DTXSID9020376	1,1'-Oxybis[2,3,4,5,6-pentabromobenz	decabromobiphenyl ether
DTXSID9020376	1,1'-Oxybis[2,3,4,5,6-pentabromobenz	decabromobiphenyl ether
DTXSID9020376	1,1'-Oxybis[2,3,4,5,6-pentabromobenz	decabromobiphenyl ether
DTXSID9020376	1,1'-Oxybis[2,3,4,5,6-pentabromobenz	decabromobiphenyl ether
DTXSID9020376	1,1'-Oxybis[2,3,4,5,6-pentabromobenz	decabromobiphenyl ether
DTXSID9020376	1,1'-Oxybis[2,3,4,5,6-pentabromobenz	decabromobiphenyl ether
DTXSID9020376	1,1'-Oxybis[2,3,4,5,6-pentabromobenz	decabromobiphenyl ether
DTXSID9020376	1,1'-Oxybis[2,3,4,5,6-pentabromobenz	decabromobiphenyl ether
DTXSID9020376	1,1'-Oxybis[2,3,4,5,6-pentabromobenz	decabromobiphenyl ether
DTXSID9020376	1,1'-Oxybis[2,3,4,5,6-pentabromobenz	decabromobiphenyl ether
DTXSID9020376	1,1'-Oxybis[2,3,4,5,6-pentabromobenz	decabromobiphenyl ether
DTXSID9020376	1,1'-Oxybis[2,3,4,5,6-pentabromobenz	decabromobiphenyl ether
DTXSID9020376	1,1'-Oxybis[2,3,4,5,6-pentabromobenz	decabromobiphenyl ether
DTXSID9020376	1,1'-Oxybis[2,3,4,5,6-pentabromobenz	decabromobiphenyl ether
DTXSID9020376	1,1'-Oxybis[2,3,4,5,6-pentabromobenz	decabromobiphenyl ether
DTXSID9020376	1,1'-Oxybis[2,3,4,5,6-pentabromobenz	decabromobiphenyl ether
DTXSID9020376	1,1'-Oxybis[2,3,4,5,6-pentabromobenz	decabromobiphenyl ether
DTXSID9020376	1,1'-Oxybis[2,3,4,5,6-pentabromobenz	decabromobiphenyl ether
DTXSID9020376	1,1'-Oxybis[2,3,4,5,6-pentabromobenz	decabromobiphenyl ether
DTXSID9020376	1,1'-Oxybis[2,3,4,5,6-pentabromobenz	decabromobiphenyl ether
DTXSID9020376	1,1'-Oxybis[2,3,4,5,6-pentabromobenz	decabromobiphenyl ether
DTXSID9020376	1,1'-Oxybis[2,3,4,5,6-pentabromobenz	decabromobiphenyl ether
DTXSID9020376	1,1'-Oxybis[2,3,4,5,6-pentabromobenz	decabromobiphenyl ether
DTXSID9020376	1,1'-Oxybis[2,3,4,5,6-pentabromobenz	decabromobiphenyl ether
DTXSID9020376	1,1'-Oxybis[2,3,4,5,6-pentabromobenz	decabromobiphenyl ether
DTXSID9020376	1,1'-Oxybis[2,3,4,5,6-pentabromobenz	decabromobiphenyl ether
DTXSID9020376	1,1'-Oxybis[2,3,4,5,6-pentabromobenz	decabromobiphenyl ether
DTXSID9020376	1,1'-Oxybis[2,3,4,5,6-pentabromobenz	decabromobiphenyl ether
DTXSID9020376	1,1'-Oxybis[2,3,4,5,6-pentabromobenz	decabromobiphenyl ether
DTXSID9020376	1,1'-Oxybis[2,3,4,5,6-pentabromobenz	decabromobiphenyl ether
DTXSID9020376	1,1'-Oxybis[2,3,4,5,6-pentabromobenz	decabromobiphenyl ether
DTXSID9020376	1,1'-Oxybis[2,3,4,5,6-pentabromobenz	decabromobiphenyl ether
DTXSID9020376	1,1'-Oxybis[2,3,4,5,6-pentabromobenz	decabromobiphenyl ether
DTXSID9020376	1,1'-Oxybis[2,3,4,5,6-pentabromobenz	decabromobiphenyl ether

DTXSID9020376	1,1'-Oxybis[2,3,4,5,6-pentabromobenz	decabromobiphenyl ether
DTXSID9020376	1,1'-Oxybis[2,3,4,5,6-pentabromobenz	decabromobiphenyl ether
DTXSID9020376	1,1'-Oxybis[2,3,4,5,6-pentabromobenz	decabromobiphenyl ether
DTXSID9020376	1,1'-Oxybis[2,3,4,5,6-pentabromobenz	decabromobiphenyl ether
DTXSID9020376	1,1'-Oxybis[2,3,4,5,6-pentabromobenz	decabromobiphenyl ether
DTXSID9020376	1,1'-Oxybis[2,3,4,5,6-pentabromobenz	decabromobiphenyl ether
DTXSID9020376	1,1'-Oxybis[2,3,4,5,6-pentabromobenz	decabromobiphenyl ether
DTXSID9020376	1,1'-Oxybis[2,3,4,5,6-pentabromobenz	decabromobiphenyl ether
DTXSID9020376	1,1'-Oxybis[2,3,4,5,6-pentabromobenz	decabromobiphenyl ether
DTXSID9020376	1,1'-Oxybis[2,3,4,5,6-pentabromobenz	decabromobiphenyl ether
DTXSID9020376	1,1'-Oxybis[2,3,4,5,6-pentabromobenz	decabromobiphenyl ether
DTXSID9020376	1,1'-Oxybis[2,3,4,5,6-pentabromobenz	decabromobiphenyl ether
DTXSID9020376	1,1'-Oxybis[2,3,4,5,6-pentabromobenz	decabromobiphenyl ether
DTXSID9020376	1,1'-Oxybis[2,3,4,5,6-pentabromobenz	decabromobiphenyl ether
DTXSID9020376	1,1'-Oxybis[2,3,4,5,6-pentabromobenz	decabromobiphenyl ether
DTXSID9020376	1,1'-Oxybis[2,3,4,5,6-pentabromobenz	decabromobiphenyl ether
DTXSID9020376	1,1'-Oxybis[2,3,4,5,6-pentabromobenz	decabromobiphenyl ether
DTXSID9020376	1,1'-Oxybis[2,3,4,5,6-pentabromobenz	decabromobiphenyl ether
DTXSID9020376	1,1'-Oxybis[2,3,4,5,6-pentabromobenz	decabromobiphenyl ether
DTXSID9020376	1,1'-Oxybis[2,3,4,5,6-pentabromobenz	decabromobiphenyl ether
DTXSID9020376	1,1'-Oxybis[2,3,4,5,6-pentabromobenz	decabromobiphenyl ether
DTXSID9020376	1,1'-Oxybis[2,3,4,5,6-pentabromobenz	decabromobiphenyl ether
DTXSID9020376	1,1'-Oxybis[2,3,4,5,6-pentabromobenz	decabromobiphenyl ether
DTXSID9020376	1,1'-Oxybis[2,3,4,5,6-pentabromobenz	decabromobiphenyl ether
DTXSID9020376	1,1'-Oxybis[2,3,4,5,6-pentabromobenz	decabromobiphenyl ether
DTXSID9020376	1,1'-Oxybis[2,3,4,5,6-pentabromobenz	decabromobiphenyl ether
DTXSID9020376	1,1'-Oxybis[2,3,4,5,6-pentabromobenz	decabromobiphenyl ether
DTXSID9020376	1,1'-Oxybis[2,3,4,5,6-pentabromobenz	decabromobiphenyl ether
DTXSID9020376	1,1'-Oxybis[2,3,4,5,6-pentabromobenz	decabromobiphenyl ether
DTXSID9020376	1,1'-Oxybis[2,3,4,5,6-pentabromobenz	decabromobiphenyl ether
DTXSID9020376	1,1'-Oxybis[2,3,4,5,6-pentabromobenz	decabromobiphenyl ether
DTXSID9020376	1,1'-Oxybis[2,3,4,5,6-pentabromobenz	decabromobiphenyl ether
DTXSID9020376	1,1'-Oxybis[2,3,4,5,6-pentabromobenz	decabromobiphenyl ether
DTXSID9020376	1,1'-Oxybis[2,3,4,5,6-pentabromobenz	decabromobiphenyl ether
DTXSID9020376	1,1'-Oxybis[2,3,4,5,6-pentabromobenz	decabromobiphenyl ether
DTXSID9020376	1,1'-Oxybis[2,3,4,5,6-pentabromobenz	decabromobiphenyl ether
DTXSID9020376	1,1'-Oxybis[2,3,4,5,6-pentabromobenz	decabromobiphenyl ether
DTXSID9020376	1,1'-Oxybis[2,3,4,5,6-pentabromobenz	decabromobiphenyl ether
DTXSID9020376	1,1'-Oxybis[2,3,4,5,6-pentabromobenz	decabromobiphenyl ether
DTXSID9020376	1,1'-Oxybis[2,3,4,5,6-pentabromobenz	decabromobiphenyl ether
DTXSID9020376	1,1'-Oxybis[2,3,4,5,6-pentabromobenz	decabromobiphenyl ether
DTXSID9020376	1,1'-Oxybis[2,3,4,5,6-pentabromobenz	decabromobiphenyl ether
DTXSID9020376	1,1'-Oxybis[2,3,4,5,6-pentabromobenz	decabromobiphenyl ether
DTXSID9020376	1,1'-Oxybis[2,3,4,5,6-pentabromobenz	decabromobiphenyl ether
DTXSID9020376	1,1'-Oxybis[2,3,4,5,6-pentabromobenz	decabromobiphenyl ether
DTXSID9020376	1,1'-Oxybis[2,3,4,5,6-pentabromobenz	decabromobiphenyl ether
DTXSID9020376	1,1'-Oxybis[2,3,4,5,6-pentabromobenz	decabromobiphenyl ether

DTXSID9020376	1,1'-Oxybis[2,3,4,5,6-pentabromobenz	decabromobiphenyl ether
DTXSID9020376	1,1'-Oxybis[2,3,4,5,6-pentabromobenz	decabromobiphenyl ether
DTXSID9020376	1,1'-Oxybis[2,3,4,5,6-pentabromobenz	decabromobiphenyl ether
DTXSID9020376	1,1'-Oxybis[2,3,4,5,6-pentabromobenz	decabromobiphenyl ether
DTXSID9020376	1,1'-Oxybis[2,3,4,5,6-pentabromobenz	decabromobiphenyl ether
DTXSID9020376	1,1'-Oxybis[2,3,4,5,6-pentabromobenz	decabromobiphenyl ether
DTXSID9020376	1,1'-Oxybis[2,3,4,5,6-pentabromobenz	decabromobiphenyl ether
DTXSID9020376	1,1'-Oxybis[2,3,4,5,6-pentabromobenz	decabromobiphenyl ether
DTXSID9020376	1,1'-Oxybis[2,3,4,5,6-pentabromobenz	decabromobiphenyl ether
DTXSID9020376	1,1'-Oxybis[2,3,4,5,6-pentabromobenz	decabromobiphenyl ether
DTXSID9020376	1,1'-Oxybis[2,3,4,5,6-pentabromobenz	decabromobiphenyl ether
DTXSID9020376	1,1'-Oxybis[2,3,4,5,6-pentabromobenz	decabromobiphenyl ether
DTXSID9020376	1,1'-Oxybis[2,3,4,5,6-pentabromobenz	decabromobiphenyl ether
DTXSID9020376	1,1'-Oxybis[2,3,4,5,6-pentabromobenz	decabromobiphenyl ether
DTXSID9020376	1,1'-Oxybis[2,3,4,5,6-pentabromobenz	decabromobiphenyl ether
DTXSID9020376	1,1'-Oxybis[2,3,4,5,6-pentabromobenz	decabromobiphenyl ether
DTXSID9020376	1,1'-Oxybis[2,3,4,5,6-pentabromobenz	decabromobiphenyl ether
DTXSID9020376	1,1'-Oxybis[2,3,4,5,6-pentabromobenz	decabromobiphenyl ether
DTXSID9020376	1,1'-Oxybis[2,3,4,5,6-pentabromobenz	decabromobiphenyl ether
DTXSID9020376	1,1'-Oxybis[2,3,4,5,6-pentabromobenz	decabromobiphenyl ether
DTXSID9020376	1,1'-Oxybis[2,3,4,5,6-pentabromobenz	decabromobiphenyl ether
DTXSID9020376	1,1'-Oxybis[2,3,4,5,6-pentabromobenz	decabromobiphenyl ether
DTXSID9020376	1,1'-Oxybis[2,3,4,5,6-pentabromobenz	decabromobiphenyl ether
DTXSID9020376	1,1'-Oxybis[2,3,4,5,6-pentabromobenz	decabromobiphenyl ether
DTXSID9020376	1,1'-Oxybis[2,3,4,5,6-pentabromobenz	decabromobiphenyl ether
DTXSID9020376	1,1'-Oxybis[2,3,4,5,6-pentabromobenz	decabromobiphenyl ether
DTXSID9020376	1,1'-Oxybis[2,3,4,5,6-pentabromobenz	decabromobiphenyl ether
DTXSID9020376	1,1'-Oxybis[2,3,4,5,6-pentabromobenz	decabromobiphenyl ether
DTXSID9020376	1,1'-Oxybis[2,3,4,5,6-pentabromobenz	decabromobiphenyl ether
DTXSID9020376	1,1'-Oxybis[2,3,4,5,6-pentabromobenz	decabromobiphenyl ether
DTXSID9020376	1,1'-Oxybis[2,3,4,5,6-pentabromobenz	decabromobiphenyl ether
DTXSID9020376	1,1'-Oxybis[2,3,4,5,6-pentabromobenz	decabromobiphenyl ether
DTXSID9020376	1,1'-Oxybis[2,3,4,5,6-pentabromobenz	decabromobiphenyl ether
DTXSID9020376	1,1'-Oxybis[2,3,4,5,6-pentabromobenz	decabromobiphenyl ether
DTXSID9020376	1,1'-Oxybis[2,3,4,5,6-pentabromobenz	decabromobiphenyl ether
DTXSID9020376	1,1'-Oxybis[2,3,4,5,6-pentabromobenz	decabromobiphenyl ether
DTXSID9020376	1,1'-Oxybis[2,3,4,5,6-pentabromobenz	decabromobiphenyl ether
DTXSID9020376	1,1'-Oxybis[2,3,4,5,6-pentabromobenz	decabromobiphenyl ether
DTXSID9020376	1,1'-Oxybis[2,3,4,5,6-pentabromobenz	decabromobiphenyl ether
DTXSID9020376	1,1'-Oxybis[2,3,4,5,6-pentabromobenz	decabromobiphenyl ether
DTXSID9020376	1,1'-Oxybis[2,3,4,5,6-pentabromobenz	decabromobiphenyl ether
DTXSID9020376	1,1'-Oxybis[2,3,4,5,6-pentabromobenz	decabromobiphenyl ether
DTXSID9020376	1,1'-Oxybis[2,3,4,5,6-pentabromobenz	decabromobiphenyl ether
DTXSID9020376	1,1'-Oxybis[2,3,4,5,6-pentabromobenz	decabromobiphenyl ether
DTXSID9020376	1,1'-Oxybis[2,3,4,5,6-pentabromobenz	decabromobiphenyl ether
DTXSID9020376	1,1'-Oxybis[2,3,4,5,6-pentabromobenz	decabromobiphenyl ether
DTXSID9020376	1,1'-Oxybis[2,3,4,5,6-pentabromobenz	decabromobiphenyl ether

DTXSID9020376	1,1'-Oxybis[2,3,4,5,6-pentabromobenz	decabromobiphenyl ether
DTXSID9020376	1,1'-Oxybis[2,3,4,5,6-pentabromobenz	decabromobiphenyl ether
DTXSID9020376	1,1'-Oxybis[2,3,4,5,6-pentabromobenz	decabromobiphenyl ether
DTXSID9020376	1,1'-Oxybis[2,3,4,5,6-pentabromobenz	decabromobiphenyl ether
DTXSID9020376	1,1'-Oxybis[2,3,4,5,6-pentabromobenz	decabromobiphenyl ether
DTXSID9020376	1,1'-Oxybis[2,3,4,5,6-pentabromobenz	decabromobiphenyl ether
DTXSID9020376	1,1'-Oxybis[2,3,4,5,6-pentabromobenz	decabromobiphenyl ether
DTXSID9020376	1,1'-Oxybis[2,3,4,5,6-pentabromobenz	decabromobiphenyl ether
DTXSID9020376	1,1'-Oxybis[2,3,4,5,6-pentabromobenz	decabromobiphenyl ether
DTXSID9020376	1,1'-Oxybis[2,3,4,5,6-pentabromobenz	decabromobiphenyl ether
DTXSID9020376	1,1'-Oxybis[2,3,4,5,6-pentabromobenz	decabromobiphenyl ether
DTXSID9020376	1,1'-Oxybis[2,3,4,5,6-pentabromobenz	decabromobiphenyl ether
DTXSID9020376	1,1'-Oxybis[2,3,4,5,6-pentabromobenz	decabromobiphenyl ether
DTXSID9020376	1,1'-Oxybis[2,3,4,5,6-pentabromobenz	decabromobiphenyl ether
DTXSID9020376	1,1'-Oxybis[2,3,4,5,6-pentabromobenz	decabromobiphenyl ether
DTXSID9020376	1,1'-Oxybis[2,3,4,5,6-pentabromobenz	decabromobiphenyl ether
DTXSID9020376	1,1'-Oxybis[2,3,4,5,6-pentabromobenz	decabromobiphenyl ether
DTXSID9020827	Methoxychlor	Methoxychlor
DTXSID9021847	Diphenyl oxide	phenyl ether
DTXSID9021847	Diphenyl oxide	phenyl ether
DTXSID9021847	Diphenyl oxide	phenyl ether
DTXSID9021847	Diphenyl oxide	phenyl ether
DTXSID9021847	Diphenyl oxide	phenyl ether
DTXSID9021847	Diphenyl oxide	phenyl ether
DTXSID9021847	Diphenyl oxide	phenyl ether
DTXSID9021847	Diphenyl oxide	phenyl ether
DTXSID9021847	Diphenyl oxide	phenyl ether
DTXSID9021847	Diphenyl oxide	phenyl ether
DTXSID9021847	Diphenyl oxide	phenyl ether
DTXSID9022079	Pentabromophenol	pentabromophenol
DTXSID9022079	Pentabromophenol	pentabromophenol
DTXSID9022079	Pentabromophenol	pentabromophenol
DTXSID9026261	Tris(1,3-dichloro-2-propyl) phosphate	tris(1,3-dichloro-2-propyl)phosphate
DTXSID9026261	Tris(1,3-dichloro-2-propyl) phosphate	tris(1,3-dichloro-2-propyl)phosphate
DTXSID9026261	Tris(1,3-dichloro-2-propyl) phosphate	tris(1,3-dichloro-2-propyl)phosphate
DTXSID9026261	Tris(1,3-dichloro-2-propyl) phosphate	tris(1,3-dichloro-2-propyl)phosphate
DTXSID9026261	Tris(1,3-dichloro-2-propyl) phosphate	tris(1,3-dichloro-2-propyl)phosphate
DTXSID9026261	Tris(1,3-dichloro-2-propyl) phosphate	tris(1,3-dichloro-2-propyl)phosphate
DTXSID9026261	Tris(1,3-dichloro-2-propyl) phosphate	tris(1,3-dichloro-2-propyl)phosphate
DTXSID9026261	Tris(1,3-dichloro-2-propyl) phosphate	tris(1,3-dichloro-2-propyl)phosphate
DTXSID9026261	Tris(1,3-dichloro-2-propyl) phosphate	tris(1,3-dichloro-2-propyl)phosphate
DTXSID9026261	Tris(1,3-dichloro-2-propyl) phosphate	tris(1,3-dichloro-2-propyl)phosphate
DTXSID9026261	Tris(1,3-dichloro-2-propyl) phosphate	tris(1,3-dichloro-2-propyl)phosphate
DTXSID9026261	Tris(1,3-dichloro-2-propyl) phosphate	tris(1,3-dichloro-2-propyl)phosphate
DTXSID9026261	Tris(1,3-dichloro-2-propyl) phosphate	tris(1,3-dichloro-2-propyl)phosphate
DTXSID9026261	Tris(1,3-dichloro-2-propyl) phosphate	tris(1,3-dichloro-2-propyl)phosphate
DTXSID9026261	Tris(1,3-dichloro-2-propyl) phosphate	tris(1,3-dichloro-2-propyl)phosphate

DTXSID9026261	Tris(1,3-dichloro-2-propyl) phosphate	tris(1,3-dichloro-2-propyl)phosphate
DTXSID9026261	Tris(1,3-dichloro-2-propyl) phosphate	tris(1,3-dichloro-2-propyl)phosphate
DTXSID9026261	Tris(1,3-dichloro-2-propyl) phosphate	tris(1,3-dichloro-2-propyl)phosphate
DTXSID9026261	Tris(1,3-dichloro-2-propyl) phosphate	tris(1,3-dichloro-2-propyl)phosphate
DTXSID9026261	Tris(1,3-dichloro-2-propyl) phosphate	tris(1,3-dichloro-2-propyl)phosphate
DTXSID9026261	Tris(1,3-dichloro-2-propyl) phosphate	tris(1,3-dichloro-2-propyl)phosphate
DTXSID9026261	Tris(1,3-dichloro-2-propyl) phosphate	tris(1,3-dichloro-2-propyl)phosphate
DTXSID9026261	Tris(1,3-dichloro-2-propyl) phosphate	tris(1,3-dichloro-2-propyl)phosphate
DTXSID9026261	Tris(1,3-dichloro-2-propyl) phosphate	tris(1,3-dichloro-2-propyl)phosphate
DTXSID9026261	Tris(1,3-dichloro-2-propyl) phosphate	tris(1,3-dichloro-2-propyl)phosphate
DTXSID9026261	Tris(1,3-dichloro-2-propyl) phosphate	tris(1,3-dichloro-2-propyl)phosphate
DTXSID9026261	Tris(1,3-dichloro-2-propyl) phosphate	tris(1,3-dichloro-2-propyl)phosphate
DTXSID9026261	Tris(1,3-dichloro-2-propyl) phosphate	tris(1,3-dichloro-2-propyl)phosphate
DTXSID9026261	Tris(1,3-dichloro-2-propyl) phosphate	tris(1,3-dichloro-2-propyl)phosphate
DTXSID9026261	Tris(1,3-dichloro-2-propyl) phosphate	tris(1,3-dichloro-2-propyl)phosphate
DTXSID9026261	Tris(1,3-dichloro-2-propyl) phosphate	tris(1,3-dichloro-2-propyl)phosphate
DTXSID9026261	Tris(1,3-dichloro-2-propyl) phosphate	tris(1,3-dichloro-2-propyl)phosphate
DTXSID9026261	Tris(1,3-dichloro-2-propyl) phosphate	tris(1,3-dichloro-2-propyl)phosphate
DTXSID9026261	Tris(1,3-dichloro-2-propyl) phosphate	tris(1,3-dichloro-2-propyl)phosphate
DTXSID9026261	Tris(1,3-dichloro-2-propyl) phosphate	tris(1,3-dichloro-2-propyl)phosphate
DTXSID9026261	Tris(1,3-dichloro-2-propyl) phosphate	tris(1,3-dichloro-2-propyl)phosphate
DTXSID9026261	Tris(1,3-dichloro-2-propyl) phosphate	tris(1,3-dichloro-2-propyl)phosphate
DTXSID9026261	Tris(1,3-dichloro-2-propyl) phosphate	tris(1,3-dichloro-2-propyl)phosphate
DTXSID9026261	Tris(1,3-dichloro-2-propyl) phosphate	tris(1,3-dichloro-2-propyl)phosphate
DTXSID9026261	Tris(1,3-dichloro-2-propyl) phosphate	tris(1,3-dichloro-2-propyl)phosphate
DTXSID9026261	Tris(1,3-dichloro-2-propyl) phosphate	tris(1,3-dichloro-2-propyl)phosphate
DTXSID9030048	2,2',4,4',5-Pentabromodiphenyl ether	pentabromodiphenyl ether
DTXSID9030048	2,2',4,4',5-Pentabromodiphenyl ether	pentabromodiphenyl ether
DTXSID9030048	2,2',4,4',5-Pentabromodiphenyl ether	pentabromodiphenyl ether
DTXSID9030048	2,2',4,4',5-Pentabromodiphenyl ether	pentabromodiphenyl ether
DTXSID9030048	2,2',4,4',5-Pentabromodiphenyl ether	pentabromodiphenyl ether
DTXSID9030048	2,2',4,4',5-Pentabromodiphenyl ether	pentabromodiphenyl ether
DTXSID9030048	2,2',4,4',5-Pentabromodiphenyl ether	pentabromodiphenyl ether
DTXSID9030048	2,2',4,4',5-Pentabromodiphenyl ether	pentabromodiphenyl ether
DTXSID9030048	2,2',4,4',5-Pentabromodiphenyl ether	pentabromodiphenyl ether
DTXSID9030048	2,2',4,4',5-Pentabromodiphenyl ether	pentabromodiphenyl ether
DTXSID9030048	2,2',4,4',5-Pentabromodiphenyl ether	pentabromodiphenyl ether
DTXSID9030048	2,2',4,4',5-Pentabromodiphenyl ether	pentabromodiphenyl ether
DTXSID9030048	2,2',4,4',5-Pentabromodiphenyl ether	pentabromodiphenyl ether
DTXSID9030048	2,2',4,4',5-Pentabromodiphenyl ether	pentabromodiphenyl ether
DTXSID9030048	2,2',4,4',5-Pentabromodiphenyl ether	pentabromodiphenyl ether
DTXSID9030048	2,2',4,4',5-Pentabromodiphenyl ether	pentabromodiphenyl ether
DTXSID9030048	2,2',4,4',5-Pentabromodiphenyl ether	pentabromodiphenyl ether
DTXSID9030048	2,2',4,4',5-Pentabromodiphenyl ether	pentabromodiphenyl ether
DTXSID9030048	2,2',4,4',5-Pentabromodiphenyl ether	pentabromodiphenyl ether
DTXSID9030048	2,2',4,4',5-Pentabromodiphenyl ether	pentabromodiphenyl ether
DTXSID9030048	2,2',4,4',5-Pentabromodiphenyl ether	pentabromodiphenyl ether

DTXSID9030048	2,2',4,4',5-Pentabromodiphenyl ether	pentabromodiphenyl ether
DTXSID9030048	2,2',4,4',5-Pentabromodiphenyl ether	pentabromodiphenyl ether
DTXSID9030048	2,2',4,4',5-Pentabromodiphenyl ether	pentabromodiphenyl ether
DTXSID9030048	2,2',4,4',5-Pentabromodiphenyl ether	pentabromodiphenyl ether
DTXSID9030048	2,2',4,4',5-Pentabromodiphenyl ether	pentabromodiphenyl ether
DTXSID9030048	2,2',4,4',5-Pentabromodiphenyl ether	pentabromodiphenyl ether
DTXSID9030048	2,2',4,4',5-Pentabromodiphenyl ether	pentabromodiphenyl ether
DTXSID9030048	2,2',4,4',5-Pentabromodiphenyl ether	pentabromodiphenyl ether
DTXSID9030048	2,2',4,4',5-Pentabromodiphenyl ether	pentabromodiphenyl ether
DTXSID9030048	2,2',4,4',5-Pentabromodiphenyl ether	pentabromodiphenyl ether
DTXSID9030048	2,2',4,4',5-Pentabromodiphenyl ether	pentabromodiphenyl ether
DTXSID9030048	2,2',4,4',5-Pentabromodiphenyl ether	pentabromodiphenyl ether
DTXSID9030048	2,2',4,4',5-Pentabromodiphenyl ether	pentabromodiphenyl ether
DTXSID9030048	2,2',4,4',5-Pentabromodiphenyl ether	pentabromodiphenyl ether
DTXSID9030048	2,2',4,4',5-Pentabromodiphenyl ether	pentabromodiphenyl ether
DTXSID9030048	2,2',4,4',5-Pentabromodiphenyl ether	pentabromodiphenyl ether
DTXSID9030048	2,2',4,4',5-Pentabromodiphenyl ether	pentabromodiphenyl ether
DTXSID9030048	2,2',4,4',5-Pentabromodiphenyl ether	pentabromodiphenyl ether
DTXSID9030048	2,2',4,4',5-Pentabromodiphenyl ether	pentabromodiphenyl ether
DTXSID9030048	2,2',4,4',5-Pentabromodiphenyl ether	pentabromodiphenyl ether
DTXSID9030048	2,2',4,4',5-Pentabromodiphenyl ether	pentabromodiphenyl ether
DTXSID9030048	2,2',4,4',5-Pentabromodiphenyl ether	pentabromodiphenyl ether
DTXSID9030048	2,2',4,4',5-Pentabromodiphenyl ether	pentabromodiphenyl ether
DTXSID9030048	2,2',4,4',5-Pentabromodiphenyl ether	pentabromodiphenyl ether
DTXSID9030048	2,2',4,4',5-Pentabromodiphenyl ether	pentabromodiphenyl ether
DTXSID9030048	2,2',4,4',5-Pentabromodiphenyl ether	pentabromodiphenyl ether
DTXSID9030048	2,2',4,4',5-Pentabromodiphenyl ether	pentabromodiphenyl ether
DTXSID9030048	2,2',4,4',5-Pentabromodiphenyl ether	pentabromodiphenyl ether
DTXSID9030048	2,2',4,4',5-Pentabromodiphenyl ether	pentabromodiphenyl ether
DTXSID9030048	2,2',4,4',5-Pentabromodiphenyl ether	pentabromodiphenyl ether
DTXSID9030048	2,2',4,4',5-Pentabromodiphenyl ether	pentabromodiphenyl ether
DTXSID9030048	2,2',4,4',5-Pentabromodiphenyl ether	pentabromodiphenyl ether
DTXSID9030048	2,2',4,4',5-Pentabromodiphenyl ether	pentabromodiphenyl ether
DTXSID9030048	2,2',4,4',5-Pentabromodiphenyl ether	pentabromodiphenyl ether
DTXSID9030048	2,2',4,4',5-Pentabromodiphenyl ether	pentabromodiphenyl ether
DTXSID9030048	2,2',4,4',5-Pentabromodiphenyl ether	pentabromodiphenyl ether
DTXSID9030048	2,2',4,4',5-Pentabromodiphenyl ether	pentabromodiphenyl ether
DTXSID9030048	2,2',4,4',5-Pentabromodiphenyl ether	pentabromodiphenyl ether
DTXSID9030048	2,2',4,4',5-Pentabromodiphenyl ether	pentabromodiphenyl ether
DTXSID9030048	2,2',4,4',5-Pentabromodiphenyl ether	pentabromodiphenyl ether
DTXSID9030048	2,2',4,4',5-Pentabromodiphenyl ether	pentabromodiphenyl ether
DTXSID9030048	2,2',4,4',5-Pentabromodiphenyl ether	pentabromodiphenyl ether
DTXSID9030048	2,2',4,4',5-Pentabromodiphenyl ether	pentabromodiphenyl ether
DTXSID9030048	2,2',4,4',5-Pentabromodiphenyl ether	pentabromodiphenyl ether
DTXSID9030048	2,2',4,4',5-Pentabromodiphenyl ether	pentabromodiphenyl ether
DTXSID9030048	2,2',4,4',5-Pentabromodiphenyl ether	pentabromodiphenyl ether
DTXSID9030048	2,2',4,4',5-Pentabromodiphenyl ether	pentabromodiphenyl ether

DTXSID9030048	2,2',4,4',5-Pentabromodiphenyl ether	pentabromodiphenyl ether
DTXSID9030048	2,2',4,4',5-Pentabromodiphenyl ether	pentabromodiphenyl ether
DTXSID9030048	2,2',4,4',5-Pentabromodiphenyl ether	pentabromodiphenyl ether
DTXSID9030048	2,2',4,4',5-Pentabromodiphenyl ether	pentabromodiphenyl ether
DTXSID9030048	2,2',4,4',5-Pentabromodiphenyl ether	pentabromodiphenyl ether
DTXSID9030048	2,2',4,4',5-Pentabromodiphenyl ether	pentabromodiphenyl ether
DTXSID9030048	2,2',4,4',5-Pentabromodiphenyl ether	pentabromodiphenyl ether
DTXSID9030048	2,2',4,4',5-Pentabromodiphenyl ether	pentabromodiphenyl ether
DTXSID9030048	2,2',4,4',5-Pentabromodiphenyl ether	pentabromodiphenyl ether
DTXSID9030048	2,2',4,4',5-Pentabromodiphenyl ether	pentabromodiphenyl ether
DTXSID9030048	2,2',4,4',5-Pentabromodiphenyl ether	pentabromodiphenyl ether
DTXSID9030048	2,2',4,4',5-Pentabromodiphenyl ether	pentabromodiphenyl ether
DTXSID9030048	2,2',4,4',5-Pentabromodiphenyl ether	pentabromodiphenyl ether
DTXSID9030048	2,2',4,4',5-Pentabromodiphenyl ether	pentabromodiphenyl ether
DTXSID9030048	2,2',4,4',5-Pentabromodiphenyl ether	pentabromodiphenyl ether
DTXSID9030048	2,2',4,4',5-Pentabromodiphenyl ether	pentabromodiphenyl ether
DTXSID9030048	2,2',4,4',5-Pentabromodiphenyl ether	pentabromodiphenyl ether
DTXSID9030048	2,2',4,4',5-Pentabromodiphenyl ether	pentabromodiphenyl ether
DTXSID9030048	2,2',4,4',5-Pentabromodiphenyl ether	pentabromodiphenyl ether
DTXSID9030048	2,2',4,4',5-Pentabromodiphenyl ether	pentabromodiphenyl ether
DTXSID9030048	2,2',4,4',5-Pentabromodiphenyl ether	pentabromodiphenyl ether
DTXSID9030048	2,2',4,4',5-Pentabromodiphenyl ether	pentabromodiphenyl ether
DTXSID9030048	2,2',4,4',5-Pentabromodiphenyl ether	pentabromodiphenyl ether
DTXSID9030048	2,2',4,4',5-Pentabromodiphenyl ether	pentabromodiphenyl ether
DTXSID9030048	2,2',4,4',5-Pentabromodiphenyl ether	pentabromodiphenyl ether
DTXSID9030048	2,2',4,4',5-Pentabromodiphenyl ether	pentabromodiphenyl ether
DTXSID9030048	2,2',4,4',5-Pentabromodiphenyl ether	pentabromodiphenyl ether
DTXSID9030048	2,2',4,4',5-Pentabromodiphenyl ether	pentabromodiphenyl ether
DTXSID9030048	2,2',4,4',5-Pentabromodiphenyl ether	pentabromodiphenyl ether
DTXSID9030048	2,2',4,4',5-Pentabromodiphenyl ether	pentabromodiphenyl ether
DTXSID9030048	2,2',4,4',5-Pentabromodiphenyl ether	pentabromodiphenyl ether
DTXSID9030048	2,2',4,4',5-Pentabromodiphenyl ether	pentabromodiphenyl ether
DTXSID9030048	2,2',4,4',5-Pentabromodiphenyl ether	pentabromodiphenyl ether
DTXSID9030048	2,2',4,4',5-Pentabromodiphenyl ether	pentabromodiphenyl ether
DTXSID9030048	2,2',4,4',5-Pentabromodiphenyl ether	pentabromodiphenyl ether
DTXSID9030048	2,2',4,4',5-Pentabromodiphenyl ether	pentabromodiphenyl ether
DTXSID9030048	2,2',4,4',5-Pentabromodiphenyl ether	pentabromodiphenyl ether
DTXSID9030048	2,2',4,4',5-Pentabromodiphenyl ether	pentabromodiphenyl ether
DTXSID9030048	2,2',4,4',5-Pentabromodiphenyl ether	pentabromodiphenyl ether
DTXSID9030048	2,2',4,4',5-Pentabromodiphenyl ether	pentabromodiphenyl ether
DTXSID9030048	2,2',4,4',5-Pentabromodiphenyl ether	pentabromodiphenyl ether
DTXSID9030048	2,2',4,4',5-Pentabromodiphenyl ether	pentabromodiphenyl ether
DTXSID9030048	2,2',4,4',5-Pentabromodiphenyl ether	pentabromodiphenyl ether
DTXSID9030048	2,2',4,4',5-Pentabromodiphenyl ether	pentabromodiphenyl ether
DTXSID9030048	2,2',4,4',5-Pentabromodiphenyl ether	pentabromodiphenyl ether
DTXSID9030048	2,2',4,4',5-Pentabromodiphenyl ether	pentabromodiphenyl ether
DTXSID9030048	2,2',4,4',5-Pentabromodiphenyl ether	pentabromodiphenyl ether

DTXSID9030048	2,2',4,4',5-Pentabromodiphenyl ether	pentabromodiphenyl ether
DTXSID9030048	2,2',4,4',5-Pentabromodiphenyl ether	pentabromodiphenyl ether
DTXSID9030048	2,2',4,4',5-Pentabromodiphenyl ether	pentabromodiphenyl ether
DTXSID9030048	2,2',4,4',5-Pentabromodiphenyl ether	pentabromodiphenyl ether
DTXSID9030048	2,2',4,4',5-Pentabromodiphenyl ether	pentabromodiphenyl ether
DTXSID9030048	2,2',4,4',5-Pentabromodiphenyl ether	pentabromodiphenyl ether
DTXSID9030048	2,2',4,4',5-Pentabromodiphenyl ether	pentabromodiphenyl ether
DTXSID9030048	2,2',4,4',5-Pentabromodiphenyl ether	pentabromodiphenyl ether
DTXSID9030048	2,2',4,4',5-Pentabromodiphenyl ether	pentabromodiphenyl ether
DTXSID9030048	2,2',4,4',5-Pentabromodiphenyl ether	pentabromodiphenyl ether
DTXSID9030048	2,2',4,4',5-Pentabromodiphenyl ether	pentabromodiphenyl ether
DTXSID9030048	2,2',4,4',5-Pentabromodiphenyl ether	pentabromodiphenyl ether
DTXSID9030048	2,2',4,4',5-Pentabromodiphenyl ether	pentabromodiphenyl ether
DTXSID9030048	2,2',4,4',5-Pentabromodiphenyl ether	pentabromodiphenyl ether
DTXSID9030048	2,2',4,4',5-Pentabromodiphenyl ether	pentabromodiphenyl ether
DTXSID9030048	2,2',4,4',5-Pentabromodiphenyl ether	pentabromodiphenyl ether
DTXSID9030048	2,2',4,4',5-Pentabromodiphenyl ether	pentabromodiphenyl ether
DTXSID9030048	2,2',4,4',5-Pentabromodiphenyl ether	pentabromodiphenyl ether
DTXSID9030048	2,2',4,4',5-Pentabromodiphenyl ether	pentabromodiphenyl ether
DTXSID9030048	2,2',4,4',5-Pentabromodiphenyl ether	pentabromodiphenyl ether
DTXSID9030048	2,2',4,4',5-Pentabromodiphenyl ether	pentabromodiphenyl ether
DTXSID9030048	2,2',4,4',5-Pentabromodiphenyl ether	pentabromodiphenyl ether
DTXSID9030048	2,2',4,4',5-Pentabromodiphenyl ether	pentabromodiphenyl ether
DTXSID9030048	2,2',4,4',5-Pentabromodiphenyl ether	pentabromodiphenyl ether
DTXSID9030048	2,2',4,4',5-Pentabromodiphenyl ether	pentabromodiphenyl ether
DTXSID9030048	2,2',4,4',5-Pentabromodiphenyl ether	pentabromodiphenyl ether
DTXSID9030048	2,2',4,4',5-Pentabromodiphenyl ether	pentabromodiphenyl ether
DTXSID9030048	2,2',4,4',5-Pentabromodiphenyl ether	pentabromodiphenyl ether
DTXSID9030048	2,2',4,4',5-Pentabromodiphenyl ether	pentabromodiphenyl ether
DTXSID9030048	2,2',4,4',5-Pentabromodiphenyl ether	pentabromodiphenyl ether
DTXSID9030048	2,2',4,4',5-Pentabromodiphenyl ether	pentabromodiphenyl ether
DTXSID9030048	2,2',4,4',5-Pentabromodiphenyl ether	pentabromodiphenyl ether
DTXSID9030048	2,2',4,4',5-Pentabromodiphenyl ether	pentabromodiphenyl ether
DTXSID9030048	2,2',4,4',5-Pentabromodiphenyl ether	pentabromodiphenyl ether
DTXSID9030048	2,2',4,4',5-Pentabromodiphenyl ether	pentabromodiphenyl ether
DTXSID9030048	2,2',4,4',5-Pentabromodiphenyl ether	pentabromodiphenyl ether
DTXSID9030048	2,2',4,4',5-Pentabromodiphenyl ether	pentabromodiphenyl ether
DTXSID9030048	2,2',4,4',5-Pentabromodiphenyl ether	pentabromodiphenyl ether
DTXSID9030048	2,2',4,4',5-Pentabromodiphenyl ether	pentabromodiphenyl ether
DTXSID9052686	2-Ethylhexyl 2,3,4,5-tetrabromobenzoa	2-ethylhexyl 2,3,4,5-tetrabromobenzoate
DTXSID9052686	2-Ethylhexyl 2,3,4,5-tetrabromobenzoa	2-ethylhexyl 2,3,4,5-tetrabromobenzoate
DTXSID9052686	2-Ethylhexyl 2,3,4,5-tetrabromobenzoa	2-ethylhexyl 2,3,4,5-tetrabromobenzoate
DTXSID9052686	2-Ethylhexyl 2,3,4,5-tetrabromobenzoa	2-ethylhexyl 2,3,4,5-tetrabromobenzoate
DTXSID9052686	2-Ethylhexyl 2,3,4,5-tetrabromobenzoa	2-ethylhexyl 2,3,4,5-tetrabromobenzoate
DTXSID9052686	2-Ethylhexyl 2,3,4,5-tetrabromobenzoa	2-ethylhexyl 2,3,4,5-tetrabromobenzoate
DTXSID9052686	2-Ethylhexyl 2,3,4,5-tetrabromobenzoa	2-ethylhexyl 2,3,4,5-tetrabromobenzoate
DTXSID9052686	2-Ethylhexyl 2,3,4,5-tetrabromobenzoa	2-ethylhexyl 2,3,4,5-tetrabromobenzoate

DTXSID9052686	2-Ethylhexyl 2,3,4,5-tetrabromobenzoa	2-ethylhexyl 2,3,4,5-tetrabromobenzoate
DTXSID9052686	2-Ethylhexyl 2,3,4,5-tetrabromobenzoa	2-ethylhexyl 2,3,4,5-tetrabromobenzoate
DTXSID9052713	1,3,5-Tris(2,3-dibromopropyl)-1,3,5-tri	tris(2,3-dibromopropyl)isocyanurate
DTXSID9052713	1,3,5-Tris(2,3-dibromopropyl)-1,3,5-tri	tris(2,3-dibromopropyl)isocyanurate
DTXSID9052713	1,3,5-Tris(2,3-dibromopropyl)-1,3,5-tri	tris(2,3-dibromopropyl)isocyanurate
DTXSID9052713	1,3,5-Tris(2,3-dibromopropyl)-1,3,5-tri	tris(2,3-dibromopropyl)isocyanurate
DTXSID9052713	1,3,5-Tris(2,3-dibromopropyl)-1,3,5-tri	tris(2,3-dibromopropyl)isocyanurate
DTXSID9052713	1,3,5-Tris(2,3-dibromopropyl)-1,3,5-tri	tris(2,3-dibromopropyl)isocyanurate
DTXSID9052713	1,3,5-Tris(2,3-dibromopropyl)-1,3,5-tri	tris(2,3-dibromopropyl)isocyanurate
DTXSID9052713	1,3,5-Tris(2,3-dibromopropyl)-1,3,5-tri	tris(2,3-dibromopropyl)isocyanurate
DTXSID9052713	1,3,5-Tris(2,3-dibromopropyl)-1,3,5-tri	tris(2,3-dibromopropyl)isocyanurate
DTXSID9052713	1,3,5-Tris(2,3-dibromopropyl)-1,3,5-tri	tris(2,3-dibromopropyl)isocyanurate
DTXSID9066752	Phosphonium, tetrakis(hydroxymethyl	tetramethylolphosphonium chloride
DTXSID9066752	Phosphonium, tetrakis(hydroxymethyl	tetramethylolphosphonium chloride
DTXSID9068328	1,3,5-Triazine-2,4,6-triamine, phospha	melamine
DTXSID9068328	1,3,5-Triazine-2,4,6-triamine, phospha	melamine
DTXSID9068328	1,3,5-Triazine-2,4,6-triamine, phospha	melamine
DTXSID9068328	1,3,5-Triazine-2,4,6-triamine, phospha	melamine
DTXSID9068328	1,3,5-Triazine-2,4,6-triamine, phospha	melamine
DTXSID9068328	1,3,5-Triazine-2,4,6-triamine, phospha	melamine
DTXSID9068328	1,3,5-Triazine-2,4,6-triamine, phospha	melamine
DTXSID90873927	2,2',4,5'-Tetrabromodiphenyl Ether	2,2',4,5'-tetrabromodiphenyl ether
DTXSID90873927	2,2',4,5'-Tetrabromodiphenyl Ether	2,2',4,5'-tetrabromodiphenyl ether
DTXSID90873927	2,2',4,5'-Tetrabromodiphenyl Ether	2,2',4,5'-tetrabromodiphenyl ether
DTXSID90873927	2,2',4,5'-Tetrabromodiphenyl Ether	2,2',4,5'-tetrabromodiphenyl ether
DTXSID90873927	2,2',4,5'-Tetrabromodiphenyl Ether	2,2',4,5'-tetrabromodiphenyl ether
DTXSID90879929	BDE-121	2,3',4,5',6-pentabromodiphenyl ether
DTXSID90889689	1,2-Benzenedicarboxylic acid, 3,4,5,6-t	tetrabromophthalic acid
DTXSID90889689	1,2-Benzenedicarboxylic acid, 3,4,5,6-t	tetrabromophthalic acid
DTXSID90889689	1,2-Benzenedicarboxylic acid, 3,4,5,6-t	tetrabromophthalic acid

PMID	Noise?	Pub Yr
11999799	1	2002
15805045	1	2005
6259446	1	1980
6309596	1	1982
23863426	1	2013
7003047	1	1980
8516773	1	1993
9598296	1	1998
22177723	2	2012
20554381	1	2010
21859130	1	2011
22693627	1	2012
30769043	1	2019
6325086	1	1984
24945793	1	2014
23746984	1	2013
25646719	1	2015
32758930	1	2020
19124146	1	2009
24064184	1	2013
24792882	1	2014
27018518	1	2016
17257644	1	2007
20447676	1	2010
6508849	1	1984
8441997	1	1993
3356864	1	1988
29783866	1	2018
30312655	1	2018
29567446	1	2018
19618683	1	2009
6289491	1	1982
28282624	2	2017
26889657	2	2016
26874059	2	2016
11766124	2	2001
11393700	2	2001
29353672	2	2018
31377596	2	2019
31351298	2	2019
31756765	2	2020
25646720	1	2015
28187300	1	2017

28163246	1	2017
28162782	1	2017
28115951	1	2016
27336727	1	2016
26898711	1	2016
28763939	1	2017
26551726	1	2015
27457671	1	2016
25350880	1	2014
24316320	1	2014
23461877	1	2013
22284495	1	2012
19848166	1	2009
26865342	1	2016
31646362	1	2020
32454264	1	2020
32402876	1	2020
26926265	1	2016
31693950	1	2020
31421579	1	2019
3131991	1	1988
31229880	1	2019
31226563	1	2019
31132918	1	2019
29758525	1	2018
31035246	1	2019
29529285	1	2018
30308812	1	2019
30312655	1	2018
30352349	1	2019
30508752	1	2019
30660026	1	2019
30857102	1	2018
30981098	1	2019
29466770	1	2018
6258818	1	1981
23804377	2	2014
27060640	1	2016
12222772	1	2002
15683155	1	2004
26780041	1	2016
26942686	1	2016
27203466	1	2016
21140013	1	2011
30228060	1	2018
30530183	1	2019
3228297	1	1988

9575663	1	1997
16201203	1	2005
30659251	2	2019
31627395	1	2019
21030912	1	2010
23218381	1	2013
24844797	1	2014
26300366	1	2015
27455714	1	2016
31208794	1	2019
31683861	1	2019
31947576	1	2020
32797829	1	2020
8097687	1	1993
31260929	1	2019
31035249	1	2019
26780041	1	2016
26354040	1	2015
25340709	1	2014
23181569	1	2012
22752998	1	2012
15884337	1	2005
29289282	1	2017
28249227	1	2017
28413084	1	2017
14659938	1	2004
11351713	1	2001
27602544	1	2016
25448284	2	2015
3377680	1	1988
30660026	1	2019
22351617	3	2013
32041069	3	2020
24596333	2	2015
26732374	2	2017
26117064	2	2015
25957833	2	2015
21561829	2	2011
22236948	2	2012
20042203	2	2010
22410623	2	2012
28523590	2	2018
16678281	2	2006
12505372	2	2003
16473389	1	2006
24777328	1	2014
25402269	1	2014

25341744	1	2014
25260812	1	2015
25223798	1	2015
25194328	1	2014
25172293	1	2014
25068372	1	2014
24945793	1	2014
24830628	1	2014
19603665	1	2009
24792882	1	2014
25818463	1	2015
24709479	1	2014
24687216	1	2014
24599331	1	2014
24594840	1	2014
24468340	1	2014
24352537	1	2014
24345234	1	2014
24342050	1	2014
24798896	1	2014
26410710	1	2016
27105166	1	2016
27018518	1	2016
26828025	1	2016
26800486	1	2016
26743646	1	2016
26725304	1	2016
26721346	1	2016
26718265	1	2016
25599136	1	2015
26490922	1	2016
25754048	1	2015
26387765	1	2015
26359264	1	2016
26302867	1	2016
26257357	1	2015
26232142	1	2015
16337636	1	2006
25893623	1	2015
24060738	1	2014
26716882	1	2016
21051070	1	2011
24295083	1	2014
22086976	1	2012
21885366	1	2011
21820030	1	2011
21723583	1	2011

21623697	1	2011
21457653	1	2011
21397395	1	2011
21116783	1	2010
20964361	1	2010
20728951	1	2010
20601118	1	2010
20582854	1	2010
20512725	1	2010
20202021	1	2010
20077216	1	2009
20077215	1	2009
20074668	1	2010
21208641	1	2011
23158692	1	2012
27239688	1	2016
23921179	1	2013
23792388	1	2013
23791110	1	2013
23769323	1	2013
23746984	1	2013
23665846	1	2013
23607346	1	2013
23500394	1	2013
22213441	1	2012
23256593	1	2013
24209347	1	2013
23084060	1	2012
22998345	1	2012
22946165	1	2012
22877936	1	2012
22825637	1	2012
22683481	1	2012
22677475	1	2013
22591914	1	2012
22417763	1	2012
23291335	1	2013
30557707	1	2019
29774514	1	2018
31129320	1	2019
31035249	1	2019
31026748	1	2019
30951941	1	2019
30835936	1	2019
30769291	1	2019
30763833	1	2019
30625682	1	2019

31209756	1	2019
30579172	1	2019
31272044	1	2019
30537814	1	2019
30481582	1	2019
30460434	1	2019
30336317	1	2019
30261454	1	2018
30153641	1	2018
30098271	1	2018
29980040	1	2018
29890436	1	2018
27164878	1	2016
30593853	1	2019
31817344	1	2019
6325086	1	1984
32933740	1	2020
32731020	1	2020
32402876	1	2020
32314580	1	2020
32229371	1	2020
32061989	1	2020
32051360	1	2020
31146158	1	2019
31923865	1	2020
29671227	1	2018
31812044	1	2020
31744693	1	2020
31704326	1	2020
31545206	1	2019
31541695	1	2019
31442778	1	2020
31351299	1	2019
31326446	1	2019
31299510	1	2019
31283198	1	2019
32018154	1	2020
27914987	1	2017
29843015	1	2018
28550799	1	2017
28499262	1	2017
28440637	1	2017
28395268	1	2017
28372837	1	2017
28324129	1	2017
28301301	1	2017
28622663	1	2017

28211328	1	2017
28688304	1	2017
27745959	1	2017
27745666	1	2016
27718046	1	2017
27693315	1	2016
27592553	1	2016
27350038	1	2016
27325014	1	2016
27297965	1	2016
27266836	1	2016
25846749	1	2016
28300664	1	2017
29102057	1	2017
29608295	1	2018
29572047	1	2018
29441599	1	2018
29428767	1	2018
29407835	1	2018
29407805	1	2018
29406119	1	2018
29356862	1	2018
29355782	1	2018
28564613	1	2017
29142177	1	2017
27234832	1	2016
29091796	1	2018
29087783	1	2018
28992476	1	2018
28919410	1	2017
28892110	1	2017
28851516	1	2017
28821000	1	2017
28811233	1	2017
28762732	1	2018
28715756	1	2017
29304458	1	2018
11878385	1	2002
15456914	1	2005
16481339	1	2006
15575264	1	2004
15680168	1	2005
17507125	1	2007
17475208	1	2007
17386623	1	2007
17254629	1	2007
17234645	1	2007

16750213	1	2006
16738895	1	2007
16406053	1	2006
16708282	1	2006
16198432	1	2005
11510556	1	2001
25924207	1	2015
11055266	1	2000
19603640	1	2009
11999794	1	2002
12054637	1	2002
14669869	1	2003
15093670	1	2004
15276329	1	2004
15284916	1	2004
16716481	1	2006
18350892	1	2008
19577631	1	2009
19524246	1	2009
19477007	1	2009
19463927	1	2009
19457453	1	2009
19450923	1	2009
18976794	1	2008
18790516	1	2008
18767142	1	2009
18760795	1	2009
18754475	1	2008
11999610	1	2002
18472134	1	2008
18342900	1	2008
18329244	1	2008
18295390	1	2008
18246587	1	2008
18155746	1	2008
18037156	1	2008
17963815	1	2008
17784851	1	2007
17620216	1	2007
17582672	1	2007
15949835	1	2006
16038199	1	2005
18569008	1	2008
20554381	1	2010
2545544	1	1989
30189763	1	2018
22236948	2	2012

23597996	1	2013
21604552	1	2011
21608493	1	2011
21783321	1	2011
23201697	1	2013
20839863	1	2010
22575175	1	2012
20230020	1	2010
19921842	1	2009
19700605	1	2009
19450923	1	2009
19350916	1	2009
19054543	1	2009
25217713	1	2014
17022393	1	2006
27281541	1	2016
12742101	1	2003
18762297	1	2009
31875573	1	2020
31797015	1	2020
31170651	1	2019
30849572	1	2019
29030119	1	2018
28903489	1	2017
28570972	1	2017
26748263	1	2016
28458244	1	2017
25244143	1	2014
27262547	1	2016
17910257	1	2007
26733012	1	2016
26123348	1	2015
26037108	1	2015
25882416	1	2015
25820830	1	2015
25600687	1	2015
28521663	1	2017
16814793	1	2006
10023042	1	1999
11510556	1	2001
16294855	1	2005
11820272	1	2002
12222772	1	2002
15224744	1	2004
16468388	1	2006
16814310	1	2006
16601080	1	2006

16916037	1	2006
16999102	1	2006
18200884	1	2007
16201203	1	2005
18589991	1	2008
18558431	1	2008
31272008	3	2019
25817024	1	2015
29126092	1	2018
23786809	1	2013
9598296	1	1998
23863426	1	2013
7003047	1	1980
23863426	1	2013
7003047	1	1980
20728951	1	2010
28324129	1	2017
29304458	1	2018
29843015	1	2018
24317224	1	2014
27060917	1	2016
21767471	1	2011
15805045	1	2005
11999799	1	2002
6259446	1	1980
26521218	2	2016
30060005	2	2019
11521497	2	2001
27336727	1	2016
29758525	1	2018
29143341	1	2018
22268003	1	2012
7345172	1	1981
21089823	1	2004
28521164	1	2017
23820075	1	2013
21863101	2	2011
21284993	2	2011
18779939	1	2009
16448790	1	2006
15352443	1	2004
29954136	1	2018
10739072	1	2000
28069368	1	2017
32443226	1	2020
22595527	1	2012
31518918	1	2020

24680724	1	2014
18406082	3	2008
16782212	2	2006
25488487	2	2015
21772022	2	2011
24184330	2	2014
17215027	2	2007
19631710	2	2009
15917144	1	2005
15958660	1	2005
16107549	1	2005
16107551	1	2005
16038199	1	2005
16830531	1	2006
15519725	1	2005
15622945	1	2004
15805046	1	2005
15687051	1	2005
15805045	1	2005
16614824	1	2006
16919340	1	2006
15506173	1	2004
12065060	1	2002
16959125	1	2006
16117131	1	2005
16120752	1	2005
16580039	1	2006
16198357	1	2005
16451863	1	2006
16192069	1	2005
12151641	1	2002
12387364	1	2002
12408642	1	2002
12380070	1	2002
10417362	1	1999
11999799	1	2002
11961221	1	2002
11961222	1	2002
11918013	1	2002
15124519	1	2004
15145223	1	2004
15180056	1	2004
15212250	1	2004
15488571	1	2004
12875373	1	2003
14552008	1	2003
12621885	1	2002

14620800	1	2003
14968842	1	2004
14987867	1	2004
27294297	1	2016
27262786	1	2016
22875421	1	2012
27128973	1	2016
27485910	1	2016
27060352	1	2016
27023809	1	2016
28466692	1	2017
23721586	1	2013
23294302	1	2013
28419962	1	2017
23186002	1	2012
23185960	1	2012
21113805	1	2011
21820030	1	2011
31362383	1	2019
21762987	1	2011
26254212	1	2015
23797055	1	2013
23916505	1	2013
22866812	1	2012
22760441	1	2013
22387749	1	2012
22024237	1	2012
22321537	1	2012
22214215	1	2012
22079498	1	2012
28644858	1	2017
9067487	1	1997
31270770	1	2019
31253486	1	2019
31207422	1	2019
31071566	1	2019
31026633	1	2019
31499316	1	2020
31701034	1	2019
8134923	1	1994
32682128	1	2020
32699379	1	2020
7886253	1	1995
32251567	1	2019
29428609	1	2018
30041130	1	2018
30014366	1	2018

29618764	1	2019
29289024	1	2018
28741109	1	2017
30830665	1	2019
30903904	1	2019
30882909	1	2019
30849572	1	2019
30713057	1	2019
30676604	1	2019
30073326	1	2018
18453545	1	2008
18313081	1	2008
18779939	1	2009
18246212	1	2008
18472139	1	2008
18243468	1	2008
18198302	1	2007
18035459	1	2008
18656495	1	2008
18550172	1	2008
18538377	1	2008
18522114	1	2008
18482827	1	2008
17982161	1	2008
17208275	1	2007
17257644	1	2007
17239511	1	2007
17312594	1	2007
17120553	1	2006
17010488	1	2007
16980691	1	2006
18589991	1	2008
17961649	1	2008
17938745	1	2007
17482735	1	2008
17396627	1	2007
17324954	1	2007
17765970	1	2008
20056574	1	2010
20020105	1	2010
20008168	1	2010
20821627	1	2010
24451063	1	2014
20884388	1	2010
20540532	1	2010
20671024	1	2010
20664651	1	2011

20549633	1	2011
20063890	1	2010
20488850	1	2011
20329534	1	2010
19045936	1	2009
19211133	1	2009
19145591	1	2009
19068229	1	2009
18800493	1	2008
18834613	1	2008
19420013	1	2009
18978342	1	2009
19954255	1	2010
19945756	1	2010
28884596	1	2017
30999658	1	2019
21133428	2	2011
3351977	1	1988
24680724	1	2014
28764132	1	2017
20947653	1	2011
16051090	1	2005
27312627	2	2016
32505289	1	2020
31229880	1	2019
25820830	1	2015
29117109	1	2017
10562696	1	1999
9598296	1	1998
21122891	1	2011
21546348	1	2011
8441997	1	1993
6508849	1	1984
3356864	1	1988
25488487	2	2015
22351617	3	2013
21561829	2	2011
28811233	1	2017
29857228	1	2018
19524246	1	2009
19457453	1	2009
12054637	1	2002
24345234	1	2014
30557707	1	2019
24121629	1	2013
31326446	1	2019
11878385	1	2002

21885366	1	2011
28572609	1	2017
32220724	2	2020
23250727	2	2013
30870749	2	2019
28866882	2	2017
32113093	2	2020
27018022	2	2016
32662595	1	2020
32556397	1	2020
24905208	1	2014
30352349	1	2019
29758525	1	2018
29653306	1	2018
27391075	1	2017
27336727	1	2016
27060640	1	2016
30530183	1	2019
21140013	1	2011
11521497	2	2001
30060005	2	2019
26521218	2	2016
27336727	1	2016
29143341	1	2018
29758525	1	2018
7345172	1	1981
22268003	1	2012
26640238	2	2016
30802654	2	2019
29634796	2	2018
23954424	2	2013
16777707	2	2006
19365754	2	2009
19720130	2	2010
26026498	2	2015
26098022	2	2015
25305463	2	2014
25462306	2	2015
25488487	2	2015
29407791	1	2018
29366955	1	2018
29289024	1	2018
28935585	1	2018
27990605	1	2017
28851516	1	2017
28843142	1	2017
29428609	1	2018

29407835	1	2018
28807763	1	2017
28764132	1	2017
28712647	1	2017
28525835	1	2017
27750173	1	2017
28131476	1	2017
27884471	1	2017
30963189	1	2019
27600263	1	2016
27588576	1	2016
28189027	1	2017
31152904	1	2019
27450237	1	2016
32559690	1	2020
32302906	1	2020
32236703	1	2020
32101552	1	2020
31791515	1	2020
31437728	1	2019
30763833	1	2019
31207422	1	2019
29625299	1	2018
30743844	1	2018
30641371	1	2019
30223335	1	2018
30202865	1	2019
30071320	1	2018
30037399	1	2018
29859315	1	2018
31326446	1	2019
22818847	1	2012
21314472	1	2011
21673328	1	2011
21912957	1	2012
22111525	1	2012
22208742	1	2012
22280972	1	2012
22467012	1	2012
21210679	1	2011
22710639	1	2012
20947653	1	2011
22847182	1	2012
22964426	1	2012
23147837	1	2013
23249762	1	2013
23334565	1	2013

23564589	1	2013
23564643	1	2013
26808241	1	2016
22476648	1	2012
18455754	1	2008
15509665	1	2005
16407092	1	2006
17010488	1	2007
17257644	1	2007
17324954	1	2007
17482735	1	2008
18035459	1	2008
21312244	1	2011
18186105	1	2008
24080000	1	2014
19174868	1	2008
20362048	1	2010
20429633	1	2010
20549613	1	2011
20650676	1	2010
20737425	1	2011
18186101	1	2008
26840066	1	2016
23816362	1	2013
25656793	1	2015
25659769	1	2015
25697951	1	2015
25732806	1	2015
25797475	1	2015
25544014	1	2015
26745384	1	2016
25542760	1	2015
26845362	1	2016
27023809	1	2016
27060352	1	2016
27060917	1	2016
27198750	1	2016
27256741	1	2016
27291303	1	2016
27343407	1	2016
24680724	1	2014
24792125	1	2014
24148424	1	2013
24157584	1	2013
24177579	1	2013
24218150	1	2014
24225586	1	2013

24246239	1	2014
24400732	1	2014
24451063	1	2014
25654621	1	2015
24559133	1	2014
27429067	1	2016
25272197	1	2014
25283367	1	2015
25387207	1	2014
25450909	1	2015
25533936	1	2015
24497080	1	2014
24513925	1	2014
26325685	1	2015
16297962	1	2006
28391934	1	2017
21546348	1	2011
21122891	1	2011
29567446	1	2018
19618683	1	2009
25867591	1	2015
22524051	1	2012
7010442	1	1980
17910257	1	2007
11878750	1	2002
25744309	1	2015
1529912	1	1992
19631710	2	2009
19056936	2	2009
20633923	2	2010
15585367	2	2005
18511058	2	2008
17014861	2	2006
27915102	2	2017
28523590	2	2018
26732374	2	2017
28951040	2	2018
23347875	2	2013
26117064	2	2015
19577631	1	2009
19344952	1	2009
19356805	1	2009
19269019	1	2009
19368196	1	2009
19477007	1	2009
19142559	1	2009
19584526	1	2009

19551757	1	2009
18800504	1	2008
18569008	1	2008
20573431	1	2010
18644697	1	2008
18657291	1	2008
18853794	1	2008
19111915	1	2009
19118610	1	2009
19124143	1	2009
18767142	1	2009
20564801	1	2010
20575536	1	2010
20580407	1	2010
20562218	1	2010
20821623	1	2010
20825970	1	2010
20875598	1	2010
20582854	1	2010
20064778	1	2010
19665796	1	2009
19723599	1	2009
19782412	1	2009
19815253	1	2010
20083292	1	2010
20096439	1	2010
20200218	1	2010
20378152	1	2010
19938002	1	2010
15865183	1	2005
15871216	1	2005
15871242	1	2005
16157171	1	2005
16198357	1	2005
16325980	1	2006
16442148	1	2006
16504358	1	2006
16614824	1	2006
20875653	1	2011
12820203	1	2003
14559253	1	2004
14620797	1	2003
15180056	1	2004
15694478	1	2005
15276329	1	2004
15519725	1	2005
15575264	1	2004

16986806	1	2006
16830527	1	2006
18191442	1	2008
18246212	1	2008
18246585	1	2008
18262388	1	2008
18186105	1	2008
18284125	1	2008
18284147	1	2008
18466951	1	2008
18514256	1	2008
17510934	1	2007
18534652	1	2008
17291651	1	2007
17346772	1	2007
17363034	1	2007
18037156	1	2008
18076970	1	2008
18174948	1	2007
18186101	1	2008
17419640	1	2007
27792943	1	2017
28214749	1	2017
28109902	1	2017
28057373	1	2017
28365503	1	2017
27745666	1	2016
27707600	1	2017
27647291	1	2017
27494656	1	2016
27814246	1	2017
28752240	1	2017
28688304	1	2017
28624943	1	2017
28525835	1	2017
28342374	1	2017
28521164	1	2017
27343943	1	2016
28477811	1	2017
28477480	1	2017
28440626	1	2017
26320833	1	2015
27414104	1	2016
26492426	1	2016
26490922	1	2016
26423281	1	2015
26412400	1	2016

26583291	1	2016
26312743	1	2015
26302867	1	2016
26232142	1	2015
26219072	1	2015
26174355	1	2015
26348671	1	2015
27297965	1	2016
27064612	1	2016
26999514	1	2016
26876804	1	2016
26843139	1	2016
26554601	1	2016
26841293	1	2016
28821000	1	2017
26795019	1	2016
26745295	1	2016
26725304	1	2016
26718265	1	2016
28768159	1	2017
31299510	1	2019
31279191	1	2019
31202941	1	2019
31202932	1	2019
31200309	1	2019
31394478	1	2019
31165938	1	2019
31146158	1	2019
31133265	1	2019
31082459	1	2019
30933770	1	2019
30594549	1	2019
31812044	1	2020
7920564	1	1993
32763577	1	2021
32758951	1	2020
32758930	1	2020
32601871	1	2020
30377961	1	2018
31804807	1	2020
31563749	1	2019
31449896	1	2019
31446271	1	2019
31404810	1	2019
31896015	1	2020
29356862	1	2018
29501037	1	2018

29459351	1	2018
29428767	1	2018
29421112	1	2018
29407835	1	2018
30496982	1	2019
29087783	1	2018
28965058	1	2017
28892110	1	2017
28494839	1	2017
29404869	1	2018
29939731	1	2018
30360547	1	2018
30228060	1	2018
30146881	1	2018
30077002	1	2018
29572047	1	2018
29929234	1	2018
29890578	1	2018
29761355	1	2018
29721597	1	2018
29713751	1	2018
29980040	1	2018
22360937	1	2012
22591914	1	2012
22571713	1	2012
22520970	1	2012
22500441	1	2011
22445922	1	2012
21938514	1	2012
22663562	1	2012
22321537	1	2012
22213441	1	2012
22212125	1	2012
22208754	1	2012
22172654	1	2012
23665846	1	2013
22415764	1	2012
22963262	1	2012
23500394	1	2013
23137556	1	2013
23062943	1	2013
23013539	1	2012
22647707	1	2012
22898824	1	2012
22818088	1	2013
22759507	1	2012
22748388	1	2012

22698370	1	2012
22998345	1	2012
21381397	1	2010
21300394	1	2011
21216435	1	2011
21208641	1	2011
26123348	1	2015
21131049	1	2011
28495472	1	2017
21122893	1	2011
21122892	1	2011
21111446	1	2011
21111444	1	2011
21067980	1	2010
21051070	1	2011
20971499	1	2010
20967578	1	2011
21898552	1	2011
21863854	1	2011
21808974	1	2012
21749232	1	2011
21724224	1	2011
21705717	1	2011
21623506	1	2011
21396716	1	2011
21596419	1	2011
21473616	1	2011
21470682	1	2011
21457653	1	2011
21441408	1	2011
21440498	1	2011
25059130	1	2014
25038592	1	2014
24995466	1	2014
24962054	1	2014
24879366	1	2014
25341744	1	2014
24756669	1	2014
25447410	1	2015
24745557	1	2014
24680724	1	2014
24670387	1	2014
24630246	1	2014
24599331	1	2014
24582366	1	2014
25703155	1	2015
25924207	1	2015

25770469	1	2015
25079089	1	2014
26073293	1	2015
25673522	1	2015
25668286	1	2015
25649405	1	2015
25618363	1	2015
25461030	1	2015
25703626	1	2015
24246239	1	2014
24209347	1	2013
24189551	1	2014
24061053	1	2014
24060738	1	2014
24056914	1	2013
24007617	1	2013
23987577	1	2013
23903933	1	2013
23831754	1	2013
23738364	1	2013
23733921	1	2013
24486972	1	2014
26111846	1	2015
23712119	1	2013
24290101	1	2014
24484690	1	2014
24291159	1	2014
24398136	1	2014
2545544	1	1989
30769043	1	2019
18406082	3	2008
16782212	2	2006
21772022	2	2011
17215027	2	2007
25488487	2	2015
24184330	2	2014
19631710	2	2009
15917144	1	2005
15958660	1	2005
16038199	1	2005
16107549	1	2005
16107551	1	2005
15506173	1	2004
15687051	1	2005
15805045	1	2005
15519725	1	2005
15805046	1	2005

15622945	1	2004
16614824	1	2006
16830531	1	2006
16919340	1	2006
16117131	1	2005
16120752	1	2005
16580039	1	2006
16192069	1	2005
16198357	1	2005
16451863	1	2006
12065060	1	2002
12151641	1	2002
12380070	1	2002
12387364	1	2002
12408642	1	2002
16959125	1	2006
10417362	1	1999
11999799	1	2002
11918013	1	2002
11961221	1	2002
11961222	1	2002
12621885	1	2002
15180056	1	2004
15124519	1	2004
15145223	1	2004
14987867	1	2004
15212250	1	2004
15488571	1	2004
12875373	1	2003
14620800	1	2003
14968842	1	2004
14552008	1	2003
27294297	1	2016
27262786	1	2016
20821627	1	2010
27060352	1	2016
27023809	1	2016
27128973	1	2016
28419962	1	2017
28466692	1	2017
23721586	1	2013
23294302	1	2013
23186002	1	2012
23185960	1	2012
27485910	1	2016
21820030	1	2011
22875421	1	2012

21113805	1	2011
22024237	1	2012
26254212	1	2015
23797055	1	2013
23916505	1	2013
22321537	1	2012
22866812	1	2012
22760441	1	2013
22387749	1	2012
22214215	1	2012
22079498	1	2012
8134923	1	1994
9067487	1	1997
31270770	1	2019
31207422	1	2019
31071566	1	2019
31026633	1	2019
31253486	1	2019
31362383	1	2019
31499316	1	2020
31701034	1	2019
32251567	1	2019
32682128	1	2020
32699379	1	2020
7886253	1	1995
30903904	1	2019
30014366	1	2018
29618764	1	2019
29428609	1	2018
29289024	1	2018
28741109	1	2017
28644858	1	2017
30882909	1	2019
30849572	1	2019
30041130	1	2018
30830665	1	2019
30713057	1	2019
30676604	1	2019
30073326	1	2018
18453545	1	2008
18313081	1	2008
18243468	1	2008
18198302	1	2007
18035459	1	2008
18246212	1	2008
18538377	1	2008
18656495	1	2008

18589991	1	2008
21762987	1	2011
18550172	1	2008
18522114	1	2008
18482827	1	2008
18472139	1	2008
17257644	1	2007
17239511	1	2007
17208275	1	2007
17120553	1	2006
17010488	1	2007
16980691	1	2006
17961649	1	2008
17938745	1	2007
17765970	1	2008
17982161	1	2008
17482735	1	2008
17396627	1	2007
17324954	1	2007
17312594	1	2007
20056574	1	2010
20020105	1	2010
20008168	1	2010
19954255	1	2010
18779939	1	2009
20884388	1	2010
20671024	1	2010
20664651	1	2011
20549633	1	2011
20540532	1	2010
20063890	1	2010
24451063	1	2014
20488850	1	2011
20329534	1	2010
19420013	1	2009
19211133	1	2009
19145591	1	2009
19068229	1	2009
19045936	1	2009
18978342	1	2009
18800493	1	2008
19945756	1	2010
18834613	1	2008
18406082	3	2008
16782212	2	2006
21772022	2	2011
24184330	2	2014

17215027	2	2007
25488487	2	2015
19631710	2	2009
15958660	1	2005
16038199	1	2005
15519725	1	2005
15805046	1	2005
16107549	1	2005
16107551	1	2005
16117131	1	2005
15622945	1	2004
15687051	1	2005
15917144	1	2005
15805045	1	2005
16919340	1	2006
16614824	1	2006
16830531	1	2006
16959125	1	2006
16198357	1	2005
16120752	1	2005
16192069	1	2005
16451863	1	2006
16580039	1	2006
12387364	1	2002
12065060	1	2002
12380070	1	2002
12151641	1	2002
12408642	1	2002
12621885	1	2002
11961221	1	2002
10417362	1	1999
11918013	1	2002
11961222	1	2002
11999799	1	2002
15145223	1	2004
15180056	1	2004
15124519	1	2004
15212250	1	2004
15488571	1	2004
15506173	1	2004
12875373	1	2003
14552008	1	2003
14620800	1	2003
14968842	1	2004
14987867	1	2004
27294297	1	2016
16980691	1	2006

27262786	1	2016
27128973	1	2016
27060352	1	2016
27023809	1	2016
23185960	1	2012
23797055	1	2013
23721586	1	2013
27485910	1	2016
28466692	1	2017
23186002	1	2012
28419962	1	2017
23294302	1	2013
22024237	1	2012
21820030	1	2011
23916505	1	2013
21762987	1	2011
22875421	1	2012
22866812	1	2012
22760441	1	2013
22387749	1	2012
22321537	1	2012
22214215	1	2012
22079498	1	2012
9067487	1	1997
31362383	1	2019
31270770	1	2019
28644858	1	2017
8134923	1	1994
31253486	1	2019
31207422	1	2019
31071566	1	2019
32682128	1	2020
31499316	1	2020
31701034	1	2019
32251567	1	2019
32699379	1	2020
7886253	1	1995
30041130	1	2018
30014366	1	2018
30073326	1	2018
29428609	1	2018
29289024	1	2018
28741109	1	2017
29618764	1	2019
30903904	1	2019
30882909	1	2019
31026633	1	2019

30830665	1	2019
30713057	1	2019
30676604	1	2019
30849572	1	2019
18453545	1	2008
18313081	1	2008
18246212	1	2008
18243468	1	2008
18198302	1	2007
18779939	1	2009
18656495	1	2008
18472139	1	2008
18550172	1	2008
18538377	1	2008
18522114	1	2008
18482827	1	2008
18035459	1	2008
17257644	1	2007
17239511	1	2007
17208275	1	2007
17120553	1	2006
17010488	1	2007
17765970	1	2008
17982161	1	2008
17961649	1	2008
17938745	1	2007
17312594	1	2007
17482735	1	2008
17396627	1	2007
17324954	1	2007
20884388	1	2010
20063890	1	2010
20056574	1	2010
20020105	1	2010
20329534	1	2010
18589991	1	2008
21113805	1	2011
26254212	1	2015
20821627	1	2010
20671024	1	2010
20664651	1	2011
20540532	1	2010
20488850	1	2011
20008168	1	2010
20549633	1	2011
24451063	1	2014
19420013	1	2009

19211133	1	2009
19045936	1	2009
19145591	1	2009
19068229	1	2009
18800493	1	2008
18834613	1	2008
18978342	1	2009
19954255	1	2010
19945756	1	2010
25113210	2	2014
16297962	1	2006
16323766	1	2005
30228060	1	2018
22897635	1	2012
31415853	1	2019
32241447	1	2020
22445833	2	2012
27183339	1	2016
25723316	1	2015
23786809	1	2013
19205903	1	2009
9714806	1	1998
6288786	3	1982
7718306	2	1994
9714806	1	1998
6375666	1	1984
15475174	1	2004
6305904	1	1983
6275573	1	1981
6277044	1	1981
6305905	1	1983
6309595	1	1982
6309596	1	1982
7968941	1	1994
6267321	1	1981
6258818	1	1981
23863426	1	2013
7003047	1	1980
11521497	2	2001
26378621	2	2016
27866252	2	2017
7891285	1	1994
28772138	1	2017
10476442	1	1999
1676650	1	1991
1676651	1	1991
1765216	1	1991

21419471	1	2011
2197455	1	1990
22578591	1	2012
27019041	1	2016
9114364	1	1997
9465270	1	1998
28886428	1	2017
32760963	1	2020
29175403	1	2018
29407800	1	2018
29518649	1	2018
29758525	1	2018
29945461	1	2018
30420131	1	2018
30935230	1	2019
32460054	1	2020
29143341	1	2018
8314462	1	1993
27391075	1	2017
6675772	1	1983
6891932	1	1982
7345172	1	1981
6288786	3	1982
7718306	2	1994
8242859	2	1993
22553152	2	2012
3325760	2	1987
26618261	2	2016
7453132	1	1981
7308025	1	1980
7394328	1	1980
7454466	1	1980
7466817	1	1980
8020156	1	1994
8441997	1	1993
8441998	1	1993
8566475	1	1995
521417	1	1979
3883695	1	1985
3611594	1	1987
353552	1	1978
397179	1	1979
6933906	1	1980
6375666	1	1984
642035	1	1978
6508849	1	1984
6686285	1	1983

684422	1	1978
6845365	1	1983
7066551	1	1982
6891932	1	1982
7262733	1	1981
6991918	1	1980
7046380	1	1981
23919627	1	2013
7064152	1	1982
3356864	1	1988
7071866	1	1982
7185616	1	1982
722202	1	1978
688215	1	1978
25637911	1	2015
23601895	1	2013
23523854	1	2013
22818088	1	2013
24905208	1	2014
21956155	1	2012
21863116	1	2011
24316320	1	2014
593298	1	1977
25242413	1	2014
21105245	1	2004
25855008	1	2015
3230254	1	1988
29024780	1	2018
286830	1	1979
25063913	1	2014
26030692	1	2015
1568270	1	1992
10476477	1	1999
15323032	1	2002
263284	1	1977
28956089	2	2018
28866882	2	2017
26123237	2	2015
26889657	2	2016
29407800	1	2018
27131816	1	2016
31226563	1	2019
29304461	1	2018
28772162	1	2017
28711770	1	2017
27207485	1	2017
27019041	1	2016

24998971	1	2014
25063913	1	2014
25808963	1	2016
26709203	1	2016
28054199	1	2017
7405831	1	1980
30583161	3	2019
17240498	3	2007
18653208	3	2008
25058892	2	2014
24576782	2	2014
23768977	2	2013
23245899	2	2013
21284993	2	2011
20704217	2	2010
21543539	2	2011
22092870	2	2012
30316155	2	2019
25488487	2	2015
28745305	2	2017
26897409	2	2016
27521295	2	2016
28806690	2	2017
28273531	2	2017
16325312	2	2006
20579684	2	2010
20129604	2	2010
19806727	2	2009
19780864	2	2010
19031922	2	2008
18677994	2	2008
27068391	1	2016
22095624	1	2012
20671024	1	2010
24680724	1	2014
24132598	1	2014
24054131	1	2013
23742027	1	2013
23554012	1	2013
23400821	1	2013
23228866	1	2013
23178840	1	2013
25912262	1	2015
20875653	1	2011
209992	1	1978
26735736	1	2016
21220147	1	2011

22560183	1	2012
21256534	1	2011
22208746	1	2012
21429558	1	2011
21470682	1	2011
21807586	1	2011
25981316	1	2015
21245014	1	2011
9067487	1	1997
32927526	1	2020
32783844	1	2020
32758951	1	2020
32629271	1	2020
32220776	1	2020
31653098	1	2019
31633338	1	2019
31454572	1	2019
31441649	1	2019
31271988	1	2019
31154115	1	2019
30761900	1	2019
26344820	1	2015
27351149	1	2016
27431696	1	2016
28502627	1	2017
30384584	1	2018
28764132	1	2017
29573718	1	2018
29642697	1	2018
29954136	1	2018
20947653	1	2011
15261986	1	2004
17120553	1	2006
16980691	1	2006
16603288	1	2006
16291560	1	2006
16091135	1	2005
15954222	1	2005
17448678	1	2007
15827307	1	2005
12523420	1	2002
14552008	1	2003
12875375	1	2003
12151641	1	2002
21033435	1	2010
12026978	1	2002
11999785	1	2002

11642440	1	2001
15862396	1	2005
20116831	1	2010
17533817	1	2007
20441148	1	2010
201769	1	1977
20171977	1	2010
20049217	1	2009
19800686	1	2010
18186102	1	2008
18719292	1	2008
18400299	1	2008
18213478	1	2008
19700605	1	2009
17905497	1	2008
23629516	1	2013
26449156	1	2015
1781444	1	1991
11057687	1	2000
2697205	1	1989
17107857	1	2006
1345555	1	1992
10880155	1	2000
22520970	1	2012
11766124	2	2001
3823038	1	1986
7718306	2	1994
9714806	1	1998
6375666	1	1984
17107857	1	2006
18157843	1	2007
19618685	1	2009
29567446	1	2018
28884596	1	2017
26076621	1	2015
19618684	1	2009
19618683	1	2009
30768125	2	2019
16708282	1	2006
31491624	1	2019
29572047	1	2018
29427708	1	2018
25817024	1	2015
27350038	1	2016
27613672	1	2017
27884139	1	2016
28413084	1	2017

29126092	1	2018
22111525	1	2012
21956155	1	2012
339076	1	1977
24880550	1	2014
23459988	1	2013
31227162	2	2019
18842055	1	2008
29682898	1	2018
30332755	1	2018
30676017	1	2019
3919466	2	1985
31154180	1	2019
25867591	1	2015
21859130	1	2011
23459988	1	2013
24880550	1	2014
1987653	1	1991
6258818	1	1981
21859130	1	2011
22351617	3	2013
30622082	3	2019
28597690	2	2018
26521218	2	2016
21622942	2	2011
29306804	1	2018
11878385	1	2002
31543489	1	2019
31394373	1	2019
30557707	1	2019
16708282	1	2006
29102866	1	2018
24261654	1	2013
24089703	1	2013
20728951	1	2010
27350038	1	2016
19524246	1	2009
19457453	1	2009
21133428	2	2011
12653288	3	2003
2545544	1	1989
8022244	1	1994
2972527	1	1988
17507217	1	2008
15585367	2	2005
15884337	1	2005
19174868	1	2008

21140013	1	2011
21089851	1	2004
3687742	1	1987
17396210	1	2007
11100410	1	2000
684422	1	1978
7245200	2	1981
7466368	1	1981
6117442	1	1981
29567446	1	2018
19618683	1	2009
17507217	1	2008
6508849	1	1984
3356864	1	1988
8441997	1	1993
29709809	2	2018
21133428	2	2011
21376363	1	2011
21561692	1	2011
21539933	1	2011
21434636	1	2011
29659266	1	2018
21351765	1	2011
21222481	1	2011
21529947	1	2011
22695692	1	2012
21705082	1	2011
29625318	1	2018
29149662	1	2018
29102191	1	2018
29078185	1	2018
22060819	1	2011
22695503	1	2012
21621844	1	2011
21105700	1	2010
18754476	1	2008
19108864	1	2009
19994895	1	2010
20000543	1	2009
20025285	1	2010
20303139	1	2010
20621327	1	2010
20630636	1	2010
20673687	1	2011
21047104	1	2010
29727946	1	2018
21122888	1	2011

21128659	1	2011
30321706	1	2019
30223335	1	2018
30159835	1	2018
30086519	1	2018
30077101	1	2018
22913883	1	2012
20959615	1	2010
24594740	1	2014
23801340	1	2013
23919519	1	2013
24021719	1	2013
25585867	1	2015
25542638	1	2015
25463724	1	2015
25463253	1	2015
22899248	1	2012
24568839	1	2014
25950133	1	2015
25113208	1	2014
25010345	1	2014
18350871	1	2008
24997969	1	2014
23500052	1	2013
24751489	1	2014
28441609	1	2017
24737022	1	2015
24433788	1	2014
28214714	1	2017
22727895	1	2012
22913625	1	2012
22982220	1	2012
23202648	1	2013
23245762	1	2013
23246747	1	2013
23416272	1	2013
23427074	1	2013
25666277	1	2015
28397063	1	2018
23542572	1	2013
27608428	1	2016
27572532	1	2016
26829245	1	2016
26760718	1	2016
26735721	1	2016
26572321	1	2015
28954370	1	2018

22698370	1	2012
23440862	1	2013
31865567	1	2020
30595018	1	2019
30708317	1	2019
32805504	1	2020
31276863	1	2019
31141740	1	2019
32209501	1	2020
18350866	1	2008
23745424	1	2013
16572773	1	2006
18075076	1	2007
18284131	1	2008
31260929	1	2019
23218312	1	2013
24892782	1	2014
8022244	1	1994
15683155	1	2004
16201203	1	2005
6255052	1	1980
220618	1	1979
32444626	1	2020
27474862	1	2016
23361180	1	2013
15212250	1	2004
15352443	1	2004
17920655	1	2008
22002787	1	2012
24865613	1	2014
26239867	1	2015
30981098	1	2019
24823833	1	2014
8441997	1	1993
6508849	1	1984
3356864	1	1988
21284993	2	2011
21863101	2	2011
18779939	1	2009
16448790	1	2006
10739072	1	2000
15352443	1	2004
31518918	1	2020
29954136	1	2018
28069368	1	2017
24680724	1	2014
22595527	1	2012

32443226	1	2020
28511039	1	2017
28413084	1	2017
30269281	1	2018
31518918	1	2020
18186102	1	2008
21089981	1	2004
17149866	2	2006
27473015	1	2016
27932249	1	2017
26729308	1	2016
28189029	1	2017
24726521	1	2014
21216340	1	2011
24375616	1	2014
23903759	1	2013
22909217	1	2012
22775271	1	2012
21028802	1	2010
18471860	1	2008
18284160	1	2008
26013366	1	2015
28917944	1	2017
30528700	1	2019
30597775	1	2019
31696622	1	2020
20049203	1	2009
28846412	1	2017
19631710	2	2009
19056936	2	2009
20633923	2	2010
17014861	2	2006
15585367	2	2005
18511058	2	2008
27915102	2	2017
28523590	2	2018
26732374	2	2017
28951040	2	2018
23347875	2	2013
26117064	2	2015
19368196	1	2009
19344952	1	2009
19269019	1	2009
19356805	1	2009
19477007	1	2009
19551757	1	2009
19577631	1	2009

19111915	1	2009
19584526	1	2009
18644697	1	2008
18657291	1	2008
18767142	1	2009
19118610	1	2009
18800504	1	2008
19142559	1	2009
18853794	1	2008
19124143	1	2009
20875598	1	2010
20573431	1	2010
20575536	1	2010
20580407	1	2010
20582854	1	2010
20562218	1	2010
20821623	1	2010
20825970	1	2010
18569008	1	2008
20875653	1	2011
19723599	1	2009
19782412	1	2009
19815253	1	2010
19938002	1	2010
20564801	1	2010
20064778	1	2010
19665796	1	2009
20083292	1	2010
20096439	1	2010
20200218	1	2010
20378152	1	2010
16325980	1	2006
15871216	1	2005
15871242	1	2005
16157171	1	2005
17291651	1	2007
15694478	1	2005
16442148	1	2006
16504358	1	2006
16614824	1	2006
16830527	1	2006
16198357	1	2005
15276329	1	2004
25447410	1	2015
12820203	1	2003
14559253	1	2004
14620797	1	2003

15865183	1	2005
15519725	1	2005
15575264	1	2004
15180056	1	2004
18284147	1	2008
18246212	1	2008
18246585	1	2008
18262388	1	2008
18186105	1	2008
18466951	1	2008
18514256	1	2008
18534652	1	2008
18284125	1	2008
17346772	1	2007
17363034	1	2007
17419640	1	2007
18191442	1	2008
17510934	1	2007
16986806	1	2006
18037156	1	2008
18076970	1	2008
18174948	1	2007
18186101	1	2008
27414104	1	2016
28342374	1	2017
28214749	1	2017
28109902	1	2017
28057373	1	2017
28365503	1	2017
27814246	1	2017
27792943	1	2017
27745666	1	2016
27707600	1	2017
27647291	1	2017
27494656	1	2016
26174355	1	2015
28521164	1	2017
28768159	1	2017
28752240	1	2017
28688304	1	2017
28624943	1	2017
28525835	1	2017
25341744	1	2014
28495472	1	2017
20967578	1	2011
28494839	1	2017
28477811	1	2017

28477480	1	2017
28440626	1	2017
26554601	1	2016
26492426	1	2016
26490922	1	2016
26423281	1	2015
26412400	1	2016
26583291	1	2016
26348671	1	2015
26320833	1	2015
26312743	1	2015
26302867	1	2016
26232142	1	2015
26219072	1	2015
26841293	1	2016
27343943	1	2016
27297965	1	2016
27064612	1	2016
26999514	1	2016
26876804	1	2016
26795019	1	2016
26745295	1	2016
26725304	1	2016
26718265	1	2016
26843139	1	2016
31165938	1	2019
31299510	1	2019
31279191	1	2019
31202941	1	2019
31202932	1	2019
31404810	1	2019
31146158	1	2019
31133265	1	2019
31082459	1	2019
30933770	1	2019
30594549	1	2019
31200309	1	2019
7920564	1	1993
32763577	1	2021
32758951	1	2020
32758930	1	2020
32601871	1	2020
31394478	1	2019
31896015	1	2020
31812044	1	2020
31804807	1	2020
31563749	1	2019

31449896	1	2019
31446271	1	2019
29501037	1	2018
29459351	1	2018
29428767	1	2018
29421112	1	2018
29407835	1	2018
29404869	1	2018
29713751	1	2018
29356862	1	2018
29087783	1	2018
28965058	1	2017
28892110	1	2017
28821000	1	2017
30377961	1	2018
30360547	1	2018
30228060	1	2018
30146881	1	2018
30077002	1	2018
29572047	1	2018
29980040	1	2018
29939731	1	2018
29929234	1	2018
29890578	1	2018
29761355	1	2018
29721597	1	2018
30496982	1	2019
22647707	1	2012
22591914	1	2012
22571713	1	2012
22520970	1	2012
22500441	1	2011
22445922	1	2012
22415764	1	2012
22360937	1	2012
22321537	1	2012
22213441	1	2012
22212125	1	2012
22208754	1	2012
23665846	1	2013
23500394	1	2013
23137556	1	2013
23062943	1	2013
23013539	1	2012
22998345	1	2012
22663562	1	2012
22963262	1	2012

22898824	1	2012
22818088	1	2013
22759507	1	2012
22748388	1	2012
22698370	1	2012
21938514	1	2012
21122892	1	2011
21381397	1	2010
21300394	1	2011
21216435	1	2011
22172654	1	2012
21440498	1	2011
21111446	1	2011
21111444	1	2011
21067980	1	2010
21051070	1	2011
20971499	1	2010
21122893	1	2011
21623506	1	2011
21898552	1	2011
21863854	1	2011
21808974	1	2012
21749232	1	2011
21724224	1	2011
21396716	1	2011
21596419	1	2011
21473616	1	2011
21470682	1	2011
21457653	1	2011
21441408	1	2011
21208641	1	2011
21705717	1	2011
24756669	1	2014
24582366	1	2014
25079089	1	2014
25059130	1	2014
25038592	1	2014
24995466	1	2014
24962054	1	2014
24745557	1	2014
24680724	1	2014
24670387	1	2014
24630246	1	2014
24599331	1	2014
24879366	1	2014
21131049	1	2011
23712119	1	2013

26123348	1	2015
26111846	1	2015
26073293	1	2015
25924207	1	2015
25770469	1	2015
25703626	1	2015
25703155	1	2015
25673522	1	2015
25668286	1	2015
25649405	1	2015
25618363	1	2015
25461030	1	2015
24007617	1	2013
24246239	1	2014
24209347	1	2013
24189551	1	2014
24061053	1	2014
24060738	1	2014
24290101	1	2014
23738364	1	2013
23987577	1	2013
23903933	1	2013
23831754	1	2013
23733921	1	2013
24056914	1	2013
24398136	1	2014
24486972	1	2014
24291159	1	2014
24484690	1	2014
23250727	2	2013
30352349	1	2019
29653306	1	2018
24998971	1	2014
32556397	1	2020
31693950	1	2020
12608925	1	2003
31646362	1	2020
2891872	1	1987
20876037	1	2011
24209347	1	2013
25804200	1	2016
27380226	1	2016
339076	1	1977
12935756	2	2003
14717166	1	2003
18996562	1	2009
25113210	2	2014

25450909	1	2015
25387207	1	2014
24497080	1	2014
17010488	1	2007
24148424	1	2013
22818847	1	2012
21762987	1	2011
21122891	1	2011
27588576	1	2016
19233527	1	2009
17419640	1	2007
31896015	1	2020
19344952	1	2009
3823038	1	1986
2792593	1	1989
9437797	1	1997
9114320	1	1997
28719877	1	2017
2731663	1	1989
22019925	1	2011
21089814	1	2004
20189218	1	2010
31071560	1	2019
25113210	2	2014
24220201	2	2013
28597690	2	2018
25462306	2	2015
26117064	2	2015
26448514	2	2016
26676538	2	2018
29339338	2	2018
30743236	2	2019
18646259	2	2008
23899252	2	2013
19660780	2	2009
14660178	2	2003
23199337	1	2013
22921657	1	2013
23038006	1	2012
23262324	1	2013
23376525	1	2013
23520876	1	2012
23542572	1	2013
23738364	1	2013
21901812	1	2014
21766320	1	2011
21762987	1	2011

21925710	1	2011
2197464	1	1990
24534697	1	2014
24497080	1	2014
24737022	1	2015
25193018	1	2014
25172293	1	2014
24368294	1	2014
24327299	1	2014
24317228	1	2014
24317224	1	2014
23839511	1	2013
23745424	1	2013
24148424	1	2013
24038512	1	2013
23999552	1	2013
25062546	1	2015
25043594	1	2015
24848787	1	2014
24880601	1	2014
31437728	1	2019
22759507	1	2012
23940625	1	2013
23798137	1	2013
24261654	1	2013
27694023	1	2017
28772138	1	2017
26845362	1	2016
26852209	1	2016
26942686	1	2016
27023809	1	2016
27060352	1	2016
27060640	1	2016
27090441	1	2017
27213241	1	2016
27256741	1	2016
27336952	1	2016
26829245	1	2016
27639615	1	2017
26743646	1	2016
28104350	1	2017
28464995	1	2017
28477480	1	2017
28525835	1	2017
28572024	1	2017
28651164	1	2017
28688304	1	2017

28734253	1	2017
28756010	1	2017
31995770	1	2020
27588576	1	2016
25687576	1	2015
25194327	1	2014
25194328	1	2014
25217713	1	2014
25222814	1	2014
25387207	1	2014
25450909	1	2015
25454222	1	2015
25460770	1	2015
25544014	1	2015
25655576	1	2015
26840066	1	2016
25681704	1	2015
28803340	1	2017
25784301	1	2015
26332257	1	2016
26393637	1	2015
26453821	1	2015
26490922	1	2016
26517991	1	2016
26602377	1	2016
26655676	1	2016
25661400	1	2015
3719120	1	1986
28764964	1	2017
30963189	1	2019
30981938	1	2019
31132478	1	2019
31141741	1	2019
31232338	1	2019
31237594	1	2019
31326446	1	2019
31520908	1	2019
32682128	1	2020
32682134	1	2020
30844666	1	2019
3694703	1	1987
30802833	1	2019
7234684	1	1981
9011064	1	1997
22178224	1	2012
22208742	1	2012
22265595	1	2012

22321537	1	2012
22509600	1	2012
22595527	1	2012
22619941	1	2011
22624401	1	2012
22694774	1	2012
3440151	1	1987
29964531	1	2017
28841423	1	2017
28863388	1	2017
28880749	1	2017
28884398	1	2018
29052980	1	2017
29151189	1	2018
29366955	1	2018
29407791	1	2018
29407835	1	2018
29428609	1	2018
30861468	1	2019
29625318	1	2018
22710639	1	2012
30014366	1	2018
30037399	1	2018
30149342	1	2018
30228060	1	2018
30321706	1	2019
30404998	1	2018
30468867	1	2019
30578573	1	2019
30597220	1	2019
30676604	1	2019
29625299	1	2018
20033485	1	2010
20020105	1	2010
19874087	1	2009
19682725	1	2009
19535737	1	2009
19449387	1	2010
19360447	1	2009
17707457	1	2007
18922559	1	2008
20100737	1	2010
18599211	1	2009
18589991	1	2008
18522114	1	2008
18495385	1	2008
18472139	1	2008

18385696	1	2008
18367248	1	2008
18174948	1	2007
17961649	1	2008
17716730	1	2008
18977512	1	2009
20821515	1	2010
21669248	1	2011
21626651	1	2013
21546348	1	2011
21437940	1	2011
21396716	1	2011
21345361	1	2011
21269970	1	2011
21193230	1	2011
21183262	1	2011
21182867	1	2011
20049210	1	2009
20851178	1	2011
20077200	1	2009
24992563	1	2014
20675151	1	2010
20609476	1	2011
20581093	1	2010
20557935	1	2010
20546880	1	2010
20429633	1	2010
20146463	1	2010
20117822	1	2010
19249332	1	2009
20851758	1	2011
15115890	1	2004
16758706	1	2006
16713668	1	2006
16640311	1	2006
16530311	1	2006
16527328	1	2006
16406053	1	2006
16297962	1	2006
15871227	1	2005
15805045	1	2005
15773480	1	2005
16793588	1	2006
15352454	1	2004
12022634	1	2002
14740727	1	2004
14740725	1	2004

12915714	1	2003
17482735	1	2008
11999799	1	2002
19395166	1	2009
11696400	1	2001
10901679	1	1999
10476413	1	1999
25016100	1	2014
15359078	1	2004
17438789	1	2007
17030062	1	2007
17239511	1	2007
11999795	1	2002
17010488	1	2007
17267017	1	2007
17481732	1	2008
17324954	1	2007
22351617	3	2013
26676538	2	2018
12850099	1	2003
20419825	1	2010
17997208	1	2008
17169381	1	2007
12850100	1	2003
12850096	1	2003
12850095	1	2003
12850094	1	2003
12850093	1	2003
16603288	1	2006
8581332	1	1995
32402876	1	2020
27350038	1	2016
29149642	2	2018
24717766	2	2014
26552522	2	2016
29149662	1	2018
7394328	1	1980
30927662	1	2019
29605586	1	2018
29783866	1	2018
30508752	1	2019
30685670	1	2019
30842366	1	2019
31398778	1	2019
31411769	1	2019
32222518	1	2020
3596894	1	1987

3883695	1	1985
7332819	1	1981
22268003	1	2012
7222047	1	1981
23629516	1	2013
11696400	1	2001
19848114	1	2009
21255595	1	2011
23635554	1	2013
28898785	1	2017
25343780	1	2015
25461749	1	2015
25826601	1	2015
26300399	1	2015
26344674	1	2015
28495519	1	2017
28851516	1	2017
23017583	1	2012
28641197	1	2017
26512412	1	2015
28181390	1	2017
26551726	1	2015
27574916	1	2016
26743178	1	2016
26709203	1	2016
27646168	1	2017
18406082	3	2008
16782212	2	2006
24184330	2	2014
21772022	2	2011
17215027	2	2007
25488487	2	2015
19631710	2	2009
15958660	1	2005
16038199	1	2005
15917144	1	2005
16107549	1	2005
16107551	1	2005
15687051	1	2005
15519725	1	2005
15622945	1	2004
15805045	1	2005
15805046	1	2005
16614824	1	2006
16580039	1	2006
16919340	1	2006
16959125	1	2006

16830531	1	2006
16120752	1	2005
16192069	1	2005
16198357	1	2005
16451863	1	2006
16117131	1	2005
12065060	1	2002
12151641	1	2002
12380070	1	2002
11999799	1	2002
12387364	1	2002
12408642	1	2002
20884388	1	2010
10417362	1	1999
11918013	1	2002
11961221	1	2002
11961222	1	2002
12621885	1	2002
15145223	1	2004
15212250	1	2004
15488571	1	2004
15180056	1	2004
14620800	1	2003
15506173	1	2004
12875373	1	2003
14552008	1	2003
15124519	1	2004
14968842	1	2004
14987867	1	2004
27128973	1	2016
27294297	1	2016
27262786	1	2016
27060352	1	2016
27023809	1	2016
28466692	1	2017
23721586	1	2013
23294302	1	2013
27485910	1	2016
23186002	1	2012
23185960	1	2012
28419962	1	2017
22875421	1	2012
21820030	1	2011
21113805	1	2011
23797055	1	2013
23916505	1	2013
22866812	1	2012

22760441	1	2013
22024237	1	2012
22321537	1	2012
22214215	1	2012
22079498	1	2012
22387749	1	2012
31253486	1	2019
9067487	1	1997
31362383	1	2019
31270770	1	2019
31207422	1	2019
31071566	1	2019
31499316	1	2020
31701034	1	2019
32251567	1	2019
8134923	1	1994
32682128	1	2020
32699379	1	2020
7886253	1	1995
31026633	1	2019
30041130	1	2018
30014366	1	2018
29618764	1	2019
30073326	1	2018
29428609	1	2018
29289024	1	2018
28741109	1	2017
21762987	1	2011
28644858	1	2017
30903904	1	2019
30882909	1	2019
30849572	1	2019
30830665	1	2019
30713057	1	2019
30676604	1	2019
18246212	1	2008
18453545	1	2008
18313081	1	2008
18243468	1	2008
18198302	1	2007
18035459	1	2008
18779939	1	2009
18656495	1	2008
18589991	1	2008
18472139	1	2008
18538377	1	2008
18522114	1	2008

18482827	1	2008
18550172	1	2008
17257644	1	2007
17239511	1	2007
17312594	1	2007
17208275	1	2007
17120553	1	2006
17010488	1	2007
16980691	1	2006
17982161	1	2008
17961649	1	2008
17938745	1	2007
17765970	1	2008
17482735	1	2008
17396627	1	2007
17324954	1	2007
20063890	1	2010
20056574	1	2010
20020105	1	2010
24451063	1	2014
26254212	1	2015
20008168	1	2010
20821627	1	2010
20671024	1	2010
20664651	1	2011
20549633	1	2011
20540532	1	2010
20488850	1	2011
20329534	1	2010
19420013	1	2009
19211133	1	2009
19145591	1	2009
19068229	1	2009
19045936	1	2009
18978342	1	2009
18800493	1	2008
18834613	1	2008
19954255	1	2010
19945756	1	2010
22575079	1	2012
30035332	1	2018
28249227	1	2017
27732871	1	2016
27613714	1	2016
27506419	1	2016
26780041	1	2016
24064184	1	2013

30981098	1	2019
22889877	1	2012
19534117	1	2009
27647291	1	2017
26558450	1	2015
25059183	1	2014
25042629	1	2015
24576575	1	2014
20875598	1	2010
22663562	1	2012
21961643	1	2011
24599331	1	2014
7003047	1	1980
23863426	1	2013
19618683	1	2009
29567446	1	2018
28884596	1	2017
18157843	1	2007
26076621	1	2015
19618685	1	2009
19618684	1	2009
29407835	1	2018
26745384	1	2016
25299509	1	2015
21951712	1	2012
31152904	1	2019
24892782	1	2014
27380226	1	2016
25804200	1	2016
339076	1	1977

Title

The toxicology of the three commercial polybrominated diphenyl oxide (ether) flame retardants.
Polybrominated diphenyl ethers (PBDEs) in U.S. computers and domestic carpet vacuuming: possible sources of hu
GC-EC analysis of polybrominated biphenyl constituents of Firemaster FF-1 using tetrabromobiphenyl as an interna
Reconstitution of some biochemical and toxicological effects of commercial mixtures of polybrominated biphenyls
Synthesis of tetrakis (hydroxymethyl) phosphonium chloride by high-concentration phosphine in industrial off-gas.
The mutagenic evaluation of tetrakis (hydroxymethyl) phosphonium sulfate using a combined testing protocol app
Metabolism and pharmacokinetics of selected halon replacement candidates.
Cardiac sensitization testing of the halon replacement candidates trifluoroiodomethane (CF3I) and 1,1,2,2,3,3,3-he
Measurements and predictions of hexadecane/air partition coefficients for 387 environmentally relevant compour
Preparation and characterization of flame retardant n-hexadecane/silicon dioxide composites as thermal energy st
Effect of cellulose acetate butyrate microencapsulated ammonium polyphosphate on the flame retardancy, mecha
Effect of rare Earth ions on the properties of composites composed of ethylene vinyl acetate copolymer and layere
Development of metal hydroxide nanoparticles from eggshell waste and seawater and their application as flame re
Patch testing with brominated epoxy resins.
Study on simultaneous recycling of EAF dust and plastic waste containing TBBPA.
Distribution of copper, silver and gold during thermal treatment with brominated flame retardants.
Effects of the brominated flame retardant TBCO on fecundity and profiles of transcripts of the HPGL-axis in Japane
Cytotoxicity of hexabromocyclododecane, 1,2-dibromo-4-(1,2-dibromoethyl) cyclohexane and 1,2,5,6-tetrabromo
Structural characterization and thermal stabilities of the isomers of the brominated flame retardant 1,2,5,6-tetrab
In vitro endocrine disruption and TCDD-like effects of three novel brominated flame retardants: TBPH, TBB, &
Formation of brominated pollutants during the pyrolysis and combustion of tetrabromobisphenol A at different te
Mechanistic and kinetic investigation on OH-initiated oxidation of tetrabromobisphenol A.
Plasma PBDE and thyroxine levels in rats exposed to Bromkal or BDE-47.
Toxicity of Bromkal 70-5DE, a technical mixture of polybrominated diphenyl ethers, following 28 d of oral exposure
Metabolism in vitro of tris(2,3-dibromopropyl)-phosphate: oxidative debromination and bis(2,3-dibromopropyl)ph
Metabolic activation of tris(2,3-dibromopropyl)phosphate to reactive intermediates. I. Covalent binding and reacti
Comparative studies on nephrotoxic effects of tris (2,3-dibromopropyl) phosphate and bis (2,3-dibromopropyl) ph
Effect of the flame retardant tris (1,3-dichloro-2-propyl) phosphate (TDCPP) on Na+-K+-ATPase and Cl- transport in
Toxicity profiling of flame retardants in zebrafish embryos using a battery of assays for developmental toxicity, neu
Melamine and its derivatives in dog and cat urine: An exposure assessment study.
Simultaneous determination of melamine, ammelide, ammeline, and cyanuric acid in milk and milk products by ga
Liver microsomal enzyme induction and toxicity studies with 2,4,5,3',4'-pentabromobiphenyl.
Immunotoxicity of organophosphate flame retardants TPHP and TDCIPP on murine dendritic cells in vitro.
Biotransformation of three phosphate flame retardants and plasticizers in primary human hepatocytes: untargetec
Determination of glucuronide conjugates of hydroxyl triphenyl phosphate (OH-TPHP) metabolites in human urine
A subchronic toxicity study of Phosflex 51B in Sprague-Dawley rats.
Development of a microporous membrane liquid-liquid extractor for organophosphate esters in human blood plas
Dermal uptake and percutaneous penetration of organophosphate esters in a human skin ex vivo model.
In vitro metabolic activation of triphenyl phosphate leading to the formation of glutathione conjugates by rat liver
Early life exposure to triphenyl phosphate: Effects on thyroid function, growth, and resting metabolic rate of Japan
Uptake, Deposition, and Metabolism of Triphenyl Phosphate in Embryonated Eggs and Chicks of Japanese Quail (C
Thyroid disruption by triphenyl phosphate, an organophosphate flame retardant, in zebrafish (Danio rerio) embryo
Electrospun template directed molecularly imprinted nanofibers incorporated with BiOI nanoflake arrays as photo

Triphenyl phosphate enhances adipogenic differentiation, glucose uptake and lipolysis via endocrine and noradren
Associations between urinary diphenyl phosphate and thyroid function.
Levels of Urinary Metabolites of Organophosphate Flame Retardants, TDCIPP, and TPHP, in Pregnant Women in Sh
The cytotoxicity of organophosphate flame retardants on HepG2, A549 and Caco-2 cells.
TPhP exposure disturbs carbohydrate metabolism, lipid metabolism, and the DNA damage repair system in zebrafi
Demographic and dietary risk factors in relation to urinary metabolites of organophosphate flame retardants in toc
High Exposure to Organophosphate Flame Retardants in Infants: Associations with Baby Products.
Atmospheric chemical reactions of alternatives of polybrominated diphenyl ethers initiated by OH: A case study or
Rapid in vitro metabolism of the flame retardant triphenyl phosphate and effects on cytotoxicity and mRNA expres
Urinary metabolites of organophosphate flame retardants and their variability in pregnant women.
Urinary metabolites of organophosphate flame retardants: temporal variability and correlations with house dust c
Multi-residue method for the determination of brominated and organophosphate flame retardants in indoor dust.
Detection of organophosphate flame retardants in furniture foam and U.S. house dust.
Long-term exposure to triphenylphosphate alters hormone balance and HPG, HPI, and HPT gene expression in zeb
Organophosphate Flame Retardants in Soils of Zhejiang Province, China: Levels, Distribution, Sources, and Exposur
Effects of triphenyl phosphate on ciliate protozoa Tetrahymena thermophila following acute exposure and sub-chr
Mitochondrial-related effects of pentabromophenol, tetrabromobisphenol A, and triphenyl phosphate on murine
Hair and Nails as Noninvasive Biomarkers of Human Exposure to Brominated and Organophosphate Flame Retarda
Organophosphate di- and tri-esters in indoor and outdoor dust from China and its implications for human exposur
Enhanced degradation of triphenyl phosphate (TPHP) in bioelectrochemical systems: Kinetics, pathway and degrad
Tricresyl phosphate and triphenyl phosphate are toxic to cultured human, monkey and dog cells.
Combinatorial immune and stress response, cytoskeleton and signal transduction effects of graphene and tripheny
Organophosphate flame retardants (OPFRs) induce genotoxicity in vivo: A survey on apoptosis, DNA methylation, I
Toxicity of Flame Retardant Isopropylated Triphenyl Phosphate: Liver, Adrenal, and Metabolic Effects.
Concentrations and variability of organophosphate esters, halogenated flame retardants, and polybrominated dipl
Acute exposure to triphenyl phosphate (TPhP) disturbs ocular development and muscular organization in zebrafish
Disruption of Nuclear Receptor Signaling Alters Triphenyl Phosphate-Induced Cardiotoxicity in Zebrafish Embryos.
Uptake and toxic effects of triphenyl phosphate on freshwater microalgae Chlorella vulgaris and Scenedesmus obli
Toxicity profiling of flame retardants in zebrafish embryos using a battery of assays for developmental toxicity, neu
Occurrence of organic phosphates in particulate matter of the vehicle exhausts and outdoor environment - A case
Effects of tris(1,3-dichloro-2-propyl) phosphate (TDCPP) and triphenyl phosphate (TPP) on sex-dependent alteratio
Urinary biomarkers for assessment of human exposure to monomeric aryl phosphate flame retardants.
Bioremediation of triphenyl phosphate by Brevibacillus brevis: Degradation characteristics and role of cytochrome
First insight into human extrahepatic metabolism of flame retardants: Biotransformation of EH-TBB and Firemaster
Neonatal triphenyl phosphate and its metabolite diphenyl phosphate exposure induce sex- and dose-dependent m
Potent induction of rat liver microsomal, drug-metabolizing enzymes by 2,3,3',4,4',5-hexabromobiphenyl, a compo
Biochemical biomarkers in liver and gill tissues of freshwater fish Carassius auratus following in vivo exposure to he
Concentrations and distributions of polybrominated diphenyl ethers and novel brominated flame retardants in tre
Debromination of hexabromobenzene by its co-grinding with CaO.
Effect of repeated administration of hexabromobenzene and 1,2,4,5-tetrabromobenzene on the levels of selected
Non-PBDE halogenated flame retardants in Canadian indoor house dust: sampling, analysis, and occurrence.
Polybrominated diphenyl ethers (PBDEs) and alternative brominated flame retardants (aBFRs) in sediments from f
Characterization of anthropogenic impacts in a large urban center by examining the spatial distribution of halogen
Presence and partitioning properties of the flame retardants pentabromotoluene, pentabromoethylbenzene and h
Legacy and alternative brominated flame retardants in outdoor dust and pine needles in mainland China: Spatial tr
Novel brominated flame retardant (NBFR) concentrations and spatial distributions in global fishmeal.
Tissue distribution and excretion of hexabromobenzene and its debrominated metabolites in the rat.

The distribution and excretion of hexabromobenzene after a single administration in rat.
The effect of selected aromatic bromine derivatives on the activity of glutathione peroxidase and transferase.
Microbial consumption of organophosphate esters in seawater under phosphorus limited conditions.
Phosphorus-Containing Flame Retardants from Biobased Chemicals and Their Application in Polyesters and Epoxy [illegible]
Synthesis and application in polypropylene of a novel of phosphorus-containing intumescent flame retardant.
Conferring flame retardancy on cotton using novel halogen-free flame retardant bifunctional monomers: synthesis [illegible]
Potential estrogenic effects of phosphorus-containing flame retardants.
Intumescent flame retardant-derived P,N co-doped porous carbon as an efficient electrocatalyst for the oxygen rec[illegible]
Synthesis of a Novel Phosphorus-Containing Flame Retardant Curing Agent and Its Application in Epoxy Resins.
Determination of human metabolites of chlorinated phosphorous flame retardants in wastewater by N-tert-butyld[illegible]
Flame Retardant Epoxy Composites on the Road of Innovation: An Analysis with Flame Retardancy Index for Future [illegible]
Flame Retardancy of Wood Fiber Materials Using Phosphorus-Modified Wheat Starch.
Optimization and validation of automated solid-phase microextraction arrow technique for determination of phos[illegible]
Disposition of the flame retardant 1,2-bis(2,4,6-tribromophenoxy)ethane in rats following administration in the die[illegible]
Spatial distribution and hazard of halogenated flame retardants and polychlorinated biphenyls to common kingfish[illegible]
In ovo exposure to brominated flame retardants Part II: Assessment of effects of TBBPA-BDBPE and BTBPE on hatc[illegible]
Non-PBDE halogenated flame retardants in Canadian indoor house dust: sampling, analysis, and occurrence.
Novel analytical approach for brominated flame retardants based on the use of gas chromatography-atmospheric [illegible]
Thermal decomposition of 1,2-bis(2,4,6-tribromophenoxy)ethane (BTBPE), a novel brominated flame retardant.
Tribromophenoxy flame retardants in the Great Lakes atmosphere.
Bioaccumulation of polybrominated diphenyl ethers, decabromodiphenyl ethane, and 1,2-bis(2,4,6-tribromophen[illegible]
Novel flame retardants, 1,2-bis(2,4,6-tribromophenoxy)ethane and 2,3,4,5,6-pentabromoethylbenzene, in United [illegible]
Oxidation reactivity of 1,2-bis(2,4,6-tribromophenoxy)ethane (BTBPE) by Compound I model of cytochrome P450s[illegible]
Effects of food-borne exposure of juvenile rainbow trout (Oncorhynchus mykiss) to emerging brominated flame re[illegible]
Brominated dioxins/furans and hydroxylated polybrominated diphenyl ethers: Occurrences in commercial 1,2-bis([illegible]
Metabolism, tissue disposition, and excretion of 1,2-bis(2,4,6-tribromophenoxy)ethane (BTBPE) in male Sprague-D[illegible]
Flame retardants in indoor air at an electronics recycling plant and at other work environments.
Concentrations, gas-particle distributions, and source indicator analysis of brominated flame retardants in air at To[illegible]
In vitro metabolism of 2-ethylhexyldiphenyl phosphate (EHDPHP) by human liver microsomes.
Isolation and identification of metabolites of 2-ethylhexyl diphenyl phosphate in rats.
Urinary biomarkers for assessment of human exposure to monomeric aryl phosphate flame retardants.
Toxicity assessment and vitellogenin expression in zebrafish (Danio rerio) embryos and larvae acutely exposed to b[illegible]
Enhanced adsorption of tetrabromobisphenol a (TBBPA) on cosmetic-derived plastic microbeads and combined eff[illegible]
Protective effects of puerarin against tetrabromobisphenol a-induced apoptosis and cardiac developmental toxicit[illegible]
Dermal bioaccessibility of flame retardants from indoor dust and the influence of topically applied cosmetics.
Responses of growth inhibition and antioxidant gene expression in earthworms (Eisenia fetida) exposed to tetrabr[illegible]
Simultaneous detection of multiple bioactive pollutants using a multiparametric biochip for water quality monitori[illegible]
Peroxisome proliferator-activated receptor ? is a target for halogenated analogs of bisphenol A.
Degradation of brominated flame retardant in computer housing plastic by supercritical fluids.
Free and bound polybrominated diphenyl ethers and tetrabromobisphenol A in freshwater sediments.
Transformation of tetrabromobisphenol A in the presence of different solvents and metals.
Solvent effects on quantitative analysis of brominated flame retardants with Soxhlet extraction.
Exposure to tetrabromobisphenol-A alters TH-associated gene expression and tadpole metamorphosis in the Pacif[illegible]
The flame retardants tetrabromobisphenol A and tetrabromobisphenol A-bisallylether suppress the induction of ir[illegible]
Biotransformation of the flame retardant tetrabromo-bisphenol A by human and rat sub-cellular liver fractions.
Occurrences and inventories of heavy metals and brominated flame retardants in wastes from printed circuit boar[illegible]
Degradation and metabolism of tetrabromobisphenol A (TBBPA) in submerged soil and soil-plant systems.

Brominated flame retardants, tetrabromobisphenol A and hexabromocyclododecane, activate mitogen-activated p
Deep sequencing of the scallop Chlamys farreri transcriptome response to tetrabromobisphenol A (TBBPA) stress.
Modulation of estradiol synthesis and aromatase activity in human choriocarcinoma JEG-3 cells exposed to tetrabr
Exploring the interactions of decabrominateddiphenyl ether and tetrabromobisphenol A with human serum album
Toxicogenomic analysis of the ability of brominated flame retardants TBBPA and BDE-209 to disrupt thyroid hormo
Heterologous antigen selection of camelid heavy chain single domain antibodies against tetrabromobisphenol A.
Study on simultaneous recycling of EAF dust and plastic waste containing TBBPA.
Quest for the binding mode of tetrabromobisphenol A with Calf thymus DNA.
Reaction of tetrabromobisphenol A (TBBPA) with manganese dioxide: kinetics, products, and pathways.
Formation of brominated pollutants during the pyrolysis and combustion of tetrabromobisphenol A at different te
Tetrabromobisphenol A (TBBPA): Possible modes of action of toxicity and carcinogenicity in rodents.
Tetrabromobisphenol A contamination and emission in printed circuit board production and implications for huma
Biotransformation of the flame retardant tetrabromobisphenol-A (TBBPA) by freshwater microalgae.
Levels of flame retardants HBCD, TBBPA and TBC in surface soils from an industrialized region of East China.
Kinetics of tetrabromobisphenol A (TBBPA) reactions with H?SO?, HNO? and HCl: implication for hydrometallurgy o
Biotin-streptavidin enzyme-linked immunosorbent assay for detecting Tetrabromobisphenol A in electronic waste.
Mammalian toxicology and human exposures to the flame retardant 2,2',6,6'-tetrabromo-4,4'-isopropylidenediphe
Comparison of the oxidation products produced by tetrahalobisphenol A flame retardants as a result of potassium
Development of carbon nanotubes/CoFe2O4 magnetic hybrid material for removal of tetrabromobisphenol A and
The oxidation of tetrabromobisphenol A by potassium monopersulfate with an iron(III)-phthalocyanine-tetrasulfor
Tetrabromobisphenol A and heavy metal exposure via dust ingestion in an e-waste recycling region in Southeast Cl
Fate and metabolism of the brominated flame retardant tetrabromobisphenol A (TBBPA) in rice cell suspension cu
Mechanistic and kinetic investigation on OH-initiated oxidation of tetrabromobisphenol A.
A high dose mode of action for tetrabromobisphenol A-induced uterine adenocarcinomas in Wistar Han rats: A crit
A review of status of tetrabromobisphenol A (TBBPA) in China.
Determination and human exposure assessment of polybrominated diphenyl ethers and tetrabromobisphenol A ir
Brominated flame retardants (BFRs): A review on environmental contamination in China.
TBBPA causes neurotoxic and the apoptotic responses in cultured mouse hippocampal neurons in vitro.
Brominated flame retardants, hexabromocyclododecane and tetrabromobisphenol A, affect proinflammatory prot
Evidence of waste electrical and electronic equipment (WEEE) relevant substances in polymeric food-contact articl
Levels and distributions of polybrominated diphenyl ethers, hexabromocyclododecane, and tetrabromobisphenol
Enhanced transformation of tetrabromobisphenol a by nitrifiers in nitrifying activated sludge.
Estimation of tetrabromobisphenol A (TBBPA) percutaneous uptake in humans using the parallelogram method.
Sodium persulfate-assisted mechanochemical degradation of tetrabromobisphenol A: Efficacy, products and pathw
Hexabromocyclododecane and tetrabromobisphenol A alter secretion of interferon gamma (IFN-?) from human im
A screening assay for thyroid hormone signaling disruption based on thyroid hormone-response gene expression a
Evaluation of 3D-human skin equivalents for assessment of human dermal absorption of some brominated flame r
Determination of the flame retardant tetrabromobisphenol A in air samples by liquid chromatography-mass spectr
Exposure of Chlamys farreri to tetrabromobisphenol A: accumulation and multibiomarker responses.
Tetrabromobisphenol A and hexabromocyclododecane flame retardants in infant-mother paired serum samples, a
Absorption and excretion of Tetrabromobisphenol A in male Wistar rats following subchronic dermal exposure.
Fate of tetrabromobisphenol A and hexabromocyclododecane brominated flame retardants in soil and uptake by p
Oxidation of flame retardant tetrabromobisphenol a by aqueous permanganate: reaction kinetics, brominated pro
Parallel biotransformation of tetrabromobisphenol A in Xenopus laevis and mammals: Xenopus as a model for end
Warm reception? Halogenated BPA flame retardants and PPAR? activation.
Differences in neonatal neurotoxicity of brominated flame retardants, PBDE 99 and TBBPA, in mice.
Comparative toxicity of a brominated flame retardant (tetrabromobisphenol A) on microalgae with single and mul

Tetrabromobisphenol A decreases cell-surface proteins involved in human natural killer (NK) cell-dependent target
Assessing oestrogenic effects of brominated flame retardants hexabromocyclododecane and tetrabromobispheno
Efficient degradation of tetrabromobisphenol A by heterostructured Ag/Bi5Nb3O15 material under the simulated
A rapid method to determine tetrabromobisphenol a in rat serum and urine by liquid chromatography-tandem ma
High-sensitivity method for determination of tetrabromobisphenol-S and tetrabromobisphenol-A derivative flame
Embryonic exposure to tetrabromobisphenol A and its metabolites, bisphenol A and tetrabromobisphenol A dime
Ecotoxicity of a brominated flame retardant (tetrabromobisphenol A) and its derivatives to aquatic organisms.
The potential of selected brominated flame retardants to affect neurological development.
Biodegradation of tetrachlorobisphenol-A in river sediment and the microbial community changes.
Characterization of an enrichment culture debrominating tetrabromobisphenol A and optimization of its activity u
Nephrotoxic potential and toxicokinetics of tetrabromobisphenol A in rat for risk assessment.
Risk assessment of tetrabromobisphenol A on cyclooxygenase-2 expression via MAP kinase/NF-kappaB/AP-1 signa
Effects of tetrabromobisphenol A, a brominated flame retardant, on the immune response to respiratory syncytial
Brominated flame retardants in dust from UK cars--within-vehicle spatial variability, evidence for degradation and
Mechanochemical degradation of tetrabromobisphenol A: performance, products and pathway.
TBBPA exposure during a sensitive developmental window produces neurobehavioral changes in larval zebrafish.
Fate of lead oxide during thermal treatment with tetrabromobisphenol A.
Quantitation of tetrabromobisphenol-A from dust sampled on consumer electronics by dispersed liquid-liquid mic
Reductive debromination of tetrabromobisphenol A by Pd/Fe bimetallic catalysts.
A humin-dependent Dehalobacter species is involved in reductive debromination of tetrabromobisphenol A.
Distribution of copper, silver and gold during thermal treatment with brominated flame retardants.
Diasteroisomer and enantiomer-specific profiles of hexabromocyclododecane and tetrabromobisphenol A in an ac
Development of a monoclonal antibody-based enzyme-linked immunosorbent assay for tetrabromobisphenol A.
Levels of tetrabromobisphenol A, hexabromocyclododecanes and polybrominated diphenyl ethers in human milk
Vapor pressure of three brominated flame retardants determined by using the Knudsen effusion method.
Transformation and removal of tetrabromobisphenol A from water in the presence of natural organic matter via la
The determination of perfluoroalkyl substances, brominated flame retardants and their metabolites in human bre
A highly sensitive and selective immunoassay for the detection of tetrabromobisphenol A in soil and sediment.
Predictors of tetrabromobisphenol-A (TBBP-A) and hexabromocyclododecanes (HBCD) in milk from Boston mothe
[Tissue distribution and bioconcentration factors of tetrabromobisphenol A in five fishes in Lake Chaohu].
Removal of tetrabromobisphenol A by conventional activated sludge, submerged membrane and membrane aerat
Tetrabromobisphenol A: tissue distribution in fish, and seasonal variation in water and sediment of Lake Chaohu, C
Development of freshwater aquatic life criteria for tetrabromobisphenol A in China.
Effect of tetrabrombisphenol A on induction of apoptosis in the testes and changes in expression of selected testic
Simultaneous extraction and determination of HBCD isomers and TBBPA by ASE and LC-MSMS in fish.
Metabolism of polybrominated diphenyl ethers and tetrabromobisphenol A by fish liver subcellular fractions in vit
Leaching characteristics of heavy metals and brominated flame retardants from waste printed circuit boards.
Metabolic perturbation, proliferation and reactive oxygen species jointly contribute to cytotoxicity of human brea
Summary of historical terrestrial toxicity data for the brominated flame retardant tetrabromobisphenol A (TBBPA):
Embryoid body-based RNA-seq analyses reveal a potential TBBPA multifaceted developmental toxicity.
In ovo exposure to brominated flame retardants Part II: Assessment of effects of TBBPA-BDBPE and BTBPE on hatc
In ovo exposure to brominated flame retardants Part I: Assessment of effects of TBBPA-BDBPE on survival, morpho
Regulation of TBBPA-induced oxidative stress on mitochondrial apoptosis in L02?cells through the Nrf2 signaling p
Transcriptomic analyses of human bronchial epithelial cells BEAS-2B exposed to brominated flame retardant (tetra
Tetrabromobisphenol A-induced depolarization of rat cerebellar granule cells: ex vivo and in vitro studies.
Molecular mechanisms and tissue targets of brominated flame retardants, BDE-47 and TBBPA, in embryo-larval life
Acetate promotes microbial reductive debromination of tetrabromobisphenol A during the startup phase of anaer

Aerobic cometabolism of tetrabromobisphenol A by marine bacterial consortia.
A DFT/TDDFT study on the mechanisms of direct and indirect photodegradation of tetrabromobisphenol A in wate
Tetrabromobisphenol A (TBBPA) inhibits denitrification via regulating carbon metabolism to decrease electron don
Ultrasensitive Determination of Tetrabromobisphenol A by Covalent Organic Framework Based Solid Phase Microe
Dermal disposition of Tetrabromobisphenol A Bis(2,3-dibromopropyl) ether (TBBPA-BDBPE) using rat and human s
Effects of tetrabromobisphenol A on maize (Zea mays L.) physiological indexes, soil enzyme activity, and soil microl
In vitro assessment of eryptotic potential of tetrabromobisphenol A and other bromophenolic flame retardants.
Transformation/degradation of tetrabromobisphenol A and its derivatives: A review of the metabolism and metab
Tetrabromobisphenol A alters soil microbial community via selective antibacterial activity.
Effects of novel brominated flame retardant TBBPA on human airway epithelial cell (A549) in vitro and proteome p
Occurrence of tetrabromobisphenol a (TBBPA) and hexabromocyclododecane (HBCD) in soil and road dust in Chon
In vitro effects of brominated flame retardants, selected metals and their mixtures on ethoxyresorufin-O-deethyla
A simulation research on the natural degradation process of tetrabromobisphenol A in soil under the atmospheric
Biomonitoring Equivalents (BEs) for tetrabromobisphenol A.
Oxidation of Flame Retardant Tetrabromobisphenol A by a Biocatalytic Nanofiber of Chloroperoxidase.
Patch testing with brominated epoxy resins.
Tetrabromobisphenol A (TBBPA): A controversial environmental pollutant.
Tetrabromobisphenol A, terabromobisphenol S and other bromophenolic flame retardants cause cytotoxic effects
Mitochondrial-related effects of pentabromophenol, tetrabromobisphenol A, and triphenyl phosphate on murine
Chemical Structure-Related Adipogenic Effects of Tetrabromobisphenol A and Its Analogues on 3T3-L1 Preadipocyt
A typical derivative and byproduct of tetrabromobisphenol A: Development of novel high-throughput immunoassa
Photodecomposition properties of brominated flame retardants (BFRs).
Enhanced Degradation of Organic Pollutants with Microwave-induced Plasma-in-liquid (MPL): Case of Flame Retar
Bioaccumulation and translocation of tetrabromobisphenol A and hexabromocyclododecanes in mangrove plants
Development of an analytical method for simultaneously determining TBBPA and HBCDs in various foods.
Review of historical aquatic toxicity and bioconcentration data for the brominated flame retardant tetrabromobisp
Tetrabromobisphenol A and hexabromocyclododecane isomers in breast milk from the general population in Beijir
Trans-generational effect of neurotoxicity and related stress response in Caenorhabditis elegans exposed to tetrab
A rapid and simple fluorescence enzyme-linked immunosorbent assay for tetrabromobisphenol A in soil samples b
Environmentally relevant doses of tetrabromobisphenol A (TBBPA) cause immunotoxicity in murine macrophages.
Sex-specific behavioral effects following developmental exposure to tetrabromobisphenol A (TBBPA) in Wistar rats
Tetrabromobisphenol A inhibits carboxylesterase activity of marine organisms from different trophic levels.
Tetrabromobisphenol A: Disposition, kinetics and toxicity in animals and humans.
Toxicogenomic analyses of the effects of BDE-47/209, TBBPA/S and TCBPA on early neural development with a hur
The effects of prosperity indices and land use indicators of an urban conurbation on the occurrence of hexabromo
Glycosylation of Tetrabromobisphenol A in Pumpkin.
TBBPA regulates calcium-mediated lysosomal exocytosis and thereby promotes invasion and migration in hepatoce
Tetrabromobisphenol A activates the hepatic interferon pathway in rats.
Biotransformation of tetrabromobisphenol A dimethyl ether back to tetrabromobisphenol A in whole pumpkin pla
In vivo assessment of dermal adhesion, penetration, and bioavailability of tetrabromobisphenol A.
Fate and O-methylating detoxification of Tetrabromobisphenol A (TBBPA) in two earthworms (Metaphire guillelmi
Identification of Emerging Brominated Chemicals as the Transformation Products of Tetrabromobisphenol A (TBBP
Effects of the earthworm Metaphire guillelmi on the mineralization, metabolism, and bound-residue formation of
Determination of tetrabromobisphenol-A/S and their main derivatives in water samples by high performance liqui
Detection of tetrabromobisphenol A and its mono- and dimethyl derivatives in fish, sediment and suspended parti
Tetrabromobisphenol A induces cellular damages in pancreatic ?-cells in vitro.
Sensitive immunoassay for simultaneous determination of tetrabromobisphenol A bis(2-hydroxyethyl) ether and te

Excretion characteristics of tetrabromobisphenol-A in Wistar rats following mouth and nose inhalation exposure.
Dietary exposure assessment of Chinese population to tetrabromobisphenol-A, hexabromocyclododecane and dec
Anaerobic co-metabolic biodegradation of tetrabromobisphenol A using a bioelectrochemical system.
Hexabromocyclododecane and tetrabromobisphenol A in sediments and paddy soils from Liaohe River Basin, Chin
The Role of Ca2+ Imbalance in the Induction of Acute Oxidative Stress and Cytotoxicity in Cultured Rat Cerebellar G
Evaluation of tetrabromobisphenol A effects on human glucocorticoid and androgen receptors: A comparison of re
Tetrabromobisphenol-A induces apoptotic death of auditory cells and hearing loss.
Emission patterns and risk assessment of polybrominated diphenyl ethers and bromophenols in water and sedime
A review of the environmental distribution, fate, and control of tetrabromobisphenol A released from sources.
Tetrabromobisphenol A and hexabromocyclododecane alter secretion of IL-1? from human immune cells.
Environmental occurrence, analysis and human exposure to the flame retardant tetrabromobisphenol-A (TBBP-A)-
TBBPA induces developmental toxicity, oxidative stress, and apoptosis in embryos and zebrafish larvae (Danio reric
Gene expression changes in immune response pathways following oral administration of tetrabromobisphenol A (T
Thin-layer chromatography coupled with high performance liquid chromatography for determining tetrabromobisp
TBBPA and Its Alternatives Disturb the Early Stages of Neural Development by Interfering with the NOTCH and WN
Contamination trends and factors affecting the transfer of hexabromocyclododecane diastereomers, tetrabromobi
Derivation of a no-significant-risk-level for tetrabromobisphenol A based on a threshold non-mutagenic cancer mo
Legacy and emerging brominated flame retardants in China: A review on food and human milk contamination, hur
Occurrence of polybrominated diphenylethers, hexabromocyclododecanes, bromophenols and tetrabromobispher
Tetrabromobisphenol A caused neurodevelopmental toxicity via disrupting thyroid hormones in zebrafish larvae.
Intranasal administration of tetrabromobisphenol A bis(2-hydroxyethyl ether) induces neurobehavioral changes in
Flame retardants, hexabromocyclododecane (HCBD) and tetrabromobisphenol a (TBBPA), alter secretion of tumor
An ultrasensitive competitive immunosensor using silica nanoparticles as an enzyme carrier for simultaneous impe
High dose tetrabromobisphenol A impairs hippocampal neurogenesis and memory retention.
Perinatal exposure to tetrabromobisphenol A (TBBPA), a brominated flame retardant, exacerbated the pneumonia
Aquatic bioaccumulation and trophic transfer of tetrabromobisphenol-A flame retardant introduced from a typical
TBBPA disposition and kinetics in pregnant and nursing Wistar Han IGS rats.
Development and validation of a quantitative UHPLC-MS/MS method for selected brominated flame retardants in
Pharmacokinetics and effects of tetrabromobisphenol a (TBBPA) to early life stages of zebrafish (Danio rerio).
Tetrabromobisphenol A disturbs zinc homeostasis in cultured cerebellar granule cells: A dual role in neurotoxicity.
Hexabromocyclododecane and tetrabromobisphenol A in tree bark from different functional areas of Shanghai, Ch
Brominated and organophosphate flame retardants target different neurodevelopmental stages, characterized wit
Simultaneous liquid chromatography-tandem mass spectrometry analysis of brominated flame retardants (tetrabr
Stimulation of ovarian cell proliferation by tetrabromobisphenol A but not tetrachlorobisphenol A through G prote
Plasmonic ELISA Based on Nanospherical Brush-Induced Signal Amplification for the Ultrasensitive Naked-Eye Simu
The occurrence and spatial-temporal distribution of tetrabromobisphenol A in the coastal intertidal zone of Qingda
Transformation of tetrabromobisphenol A by Rhodococcus jostii RHA1: Effects of heavy metals.
Anaerobic biotransformation of tetrabromobisphenol A, tetrachlorobisphenol A, and bisphenol A in estuarine sedi
The effect of a brominated flame retardant, tetrabromobisphenol-A, on free radical formation in human neutrophi
Toxicokinetics of tetrabromobisphenol a in humans and rats after oral administration.
Distribution and fate of HBCD and TBBPA brominated flame retardants in North Sea estuaries and aquatic food we
Anti-thyroid hormonal activity of tetrabromobisphenol A, a flame retardant, and related compounds: Affinity to th
Effects of housing condition on experimental outcome in a reproduction toxicity study.
The interaction of the brominated flame retardant: tetrabromobisphenol A with phospholipid membranes.
Trace determination of the flame retardant tetrabromobisphenol A in the atmosphere by gas chromatography-mas
Cytotoxicity of TBBPA and effects on proliferation, cell cycle and MAPK pathways in mammalian cells.
The effects of dose, route, and repeated dosing on the disposition and kinetics of tetrabromobisphenol A in male F

Catalytic destruction of brominated aromatic compounds studied in a catalyst microbed coupled to gas chromatog
Toxicity of tetrabromobisphenol A (TBBPA) in zebrafish (Danio rerio) in a partial life-cycle test.
Toxicity of three halogenated flame retardants to nitrifying bacteria, red clover (Trifolium pratense), and a soil inve
Biodegradability of tetrabromobisphenol A and tribromophenol by activated sludge.
Effects of the brominated flame retardant tetrabromobisphenol-A (TBBPA) on cell signaling and function of Mytilus
Brominated flame retardants in laboratory air.
Concentrations of Polybrominated Diphenyl Ethers, Hexabromocyclododecanes and Tetrabromobisphenol-A in Bre
Metabolism, excretion and distribution of the flame retardant tetrabromobisphenol-A in conventional and bile-duc
Dietary exposure assessment of Chinese adults and nursing infants to tetrabromobisphenol-A and hexabromocyclo
Exposure to polybrominated diphenyl ethers and tetrabromobisphenol A among computer technicians.
Thyroid hormonal activity of the flame retardants tetrabromobisphenol A and tetrachlorobisphenol A.
Enrichment of a microbial culture capable of reductive debromination of the flame retardant tetrabromobispheno
Unexpected nephrotoxicity induced by tetrabromobisphenol A in newborn rats.
Effects of the brominated flame retardants hexabromocyclododecane (HBCDD), and tetrabromobisphenol A (TBBP
LC-ESI-MS-MS method for the analysis of tetrabromobisphenol A in sediment and sewage sludge.
Effects of tetrabromobisphenol A, brominated flame retardant, in ICR mice after prenatal and postnatal exposure.
Oxidation of flame retardant tetrabromobisphenol A by singlet oxygen.
Developmental toxicity of brominated flame retardants, tetrabromobisphenol A and 1,2,5,6,9,10-hexabromocyclo
Determination of tetrabromobisphenol-A, tetrachlorobisphenol-A and bisphenol-A in soil by ultrasonic assisted ext
Proteomic studies in zebrafish liver cells exposed to the brominated flame retardants HBCD and TBBPA.
Neurobehavioral effects of tetrabromobisphenol A, a brominated flame retardant, in mice.
Anti-thyroid hormone activity of bisphenol A, tetrabromobisphenol A and tetrachlorobisphenol A in an improved r
Zn dust mediated reductive debromination of tetrabromobisphenol A (TBBPA).
Molecular targets of TBBPA in zebrafish analysed through integration of genomic and proteomic approaches.
Exposure assessment of French women and their newborns to tetrabromobisphenol-A: occurrence measurements
Assessing the toxicity of TBBPA and HBCD by zebrafish embryo toxicity assay and biomarker analysis.
Analytical and environmental aspects of the flame retardant tetrabromobisphenol-A and its derivatives.
Microbial O-methylation of the flame retardant tetrabromobisphenol-A.
Thermal formation of PBDD/F from tetrabromobisphenol A--a comparison of polymer linked TBBP A with its additi
Levels and trends of HBCD and BDEs in the European and Asian environments, with some information for other BF
EPR studies of in vivo radical production by 3,3',5,5'-tetrabromobisphenol A (TBBPA) in the Sprague-Dawley rat.
Tetrabromobisphenol A (TBBPA), induces cell death in TM4 Sertoli cells by modulating Ca2+ transport proteins and
Exposure to tetrabromobisphenol A (TBBPA) in Wistar rats: neurobehavioral effects in offspring from a one-genera
Determination of brominated flame retardants and brominated dioxins in fish collected from three regions of Japa
Pyrolysis of tetrabromobisphenol-A containing paper laminated printed circuit boards.
Tetrabromobisphenol A (TBBPA) and hexabromocyclododecanes (HBCDs) in tissues of humans, dolphins, and shark
Bioaccumulation and physiological effects of tetrabromobisphenol A in coontail Ceratophyllum demersum L.
The widely utilized brominated flame retardant tetrabromobisphenol A (TBBPA) is a potent inhibitor of the SERCA
Biotransformation and cytotoxicity of a brominated flame retardant, tetrabromobisphenol A, and its analogues in r
Absorption, distribution, metabolism and excretion of intravenously and orally administered tetrabromobisphenol
Evaluation of soil flushing potential for clean-up of desert soil contaminated by industrial wastewater.
Probing new approaches using atmospheric pressure photo ionization for the analysis of brominated flame retarda
Determination of brominated flame retardants in food by LC-MS/MS: diastereoisomer-specific hexabromocyclodo
Preparation and characterization of flame retardant n-hexadecane/silicon dioxide composites as thermal energy st
[Status of the upper respiratory tract and skin of workers manufacturing artificial mineral wool].
Preparation and characterisation of flame retardant encapsulated with functionalised silica-based shell.
Degradation of brominated flame retardant in computer housing plastic by supercritical fluids.

High throughput sample preparation in combination with gas chromatography coupled to triple quadrupole tander
[Comparison of bacterial diversity of polluted and unpolluted sediment by brominated flame retardant].
Study of the transference rules for bromine in waste printed circuit boards during microwave-induced pyrolysis.
Brominated and organophosphate flame retardants in selected consumer products on the Japanese market in 200
Environmental occurrence of emerging and legacy brominated flame retardants near suspected sources in Norway
Deposition history of brominated flame retardant compounds in an ice core from Holtedahlfonna, Svalbard, Norwa
Removal of brominated flame retardant from electrical and electronic waste plastic by solvothermal technique.
Modification and calibration of a passive air sampler for monitoring vapor and particulate phase brominated flame
Current-use brominated flame retardants in water, sediment, and fish from English lakes.
Effects of methyl mercury in combination with polychlorinated biphenyls and brominated flame retardants on the
Zn dust mediated reductive debromination of tetrabromobisphenol A (TBBPA).
Existence state of bromine as an indicator of the source of brominated flame retardants in indoor dust.
Alkaline reforming of brominated fire-retardant plastics: fate of bromine and antimony.
Complete debromination of decabromodiphenyl ether using the integration of Dehalococcoides sp. strain CBDB1 a
Dynamic modeling of food-chain accumulation of brominated flame retardants in fish from the Ebro River Basin, S
Improving the accuracy of hand-held X-ray fluorescence spectrometers as a tool for monitoring brominated flame
The effect of brominated flame retardants on neurotransmitter uptake into rat brain synaptosomes and vesicles.
Methods for the determination of phenolic brominated flame retardants, and by-products, formulation intermedia
Leaching behavior of Sb and Br from E-waste flame retardant plastics.
Single sample preparation for brominated flame retardants in fish and shellfish with dual detection: GC-MS/MS (PI
Evaluation of Direct Analysis in Real Time - High Resolution Mass Spectrometry (DART-HRMS) for WEEE specific sul
Species and habitat-dependent accumulation and biomagnification of brominated flame retardants and PBDE met
WEEE plastic sorting for bromine essential to enforce EU regulation.
From the Cover: Exposure to an Environmentally Relevant Mixture of Brominated Flame Retardants Decreased p-?
Bromine in plastic consumer products - Evidence for the widespread recycling of electronic waste.
Biodegradation of brominated and organophosphorus flame retardants.
Cadmium, lead and bromine in beached microplastics.
An efficient and fast analytical procedure for the bromine determination in waste electrical and electronic equipm
Reductive transformation of hexabromocyclododecane (HBCD) by FeS.
Pyrolysis of waste electrical and electronic equipment: effect of antinomy trioxide on the pyrolysis of styrenic poly
Bromine content and brominated flame retardants in food and animal feed from the UK.
Rapid identification of polystyrene foam wastes containing hexabromocyclododecane or its alternative polymeric b
Fingerprint analysis of brominated flame retardants and Dechloranes in North Sea sediments.
Determination of bromine and tin compounds in plastics using laser ablation inductively coupled plasma mass spe
Magnetic solid-phase extraction of brominated flame retardants from environmental waters with graphene-doped
High-resolution mass spectrometry provides novel insights into products of human metabolism of organophospha
Towards a generic procedure for the detection of relevant contaminants from waste electric and electronic equipn
New approach based on solid-phase microextraction to estimate polydimethylsiloxane fibre coating-water distribu
Brominated flame retardants induce intragenic recombination in mammalian cells.
Brominated flame retardants in laboratory air.
Temporal development of brominated flame retardants in peregrine Falcon (Falco peregrinus) eggs from South Gre
Pressurised hot water extraction coupled on-line with liquid chromatography-gas chromatography for the determi
Debromination of hexabromobenzene by its co-grinding with CaO.
Photochemical decomposition of 15 polybrominated diphenyl ether congeners in methanol/water.
Brominated flame retardants in polar bears (Ursus maritimus) from Alaska, the Canadian Arctic, East Greenland, ar
Simple approach for the determination of brominated flame retardants in environmental solid samples based on s
In vitro profiling of the endocrine-disrupting potency of brominated flame retardants.

Bioaccumulation and trophic transfer of some brominated flame retardants in a Lake Winnipeg (Canada) food web
Accumulation of organochlorines and brominated flame retardants in the eggs and nestlings of great tits, Parus ma
Temporal trends (1986-2004) of organochlorines and brominated flame retardants in tawny owl eggs from norther
The effect of selected aromatic bromine derivatives on the activity of glutathione peroxidase and transferase.
Linking PBDEs in house dust to consumer products using X-ray fluorescence.
Concentrations of brominated flame retardants in dust from United Kingdom cars, homes, and offices: causes of va
Quantification of 16 urinary biomarkers of exposure to flame retardants, plasticizers, and organophosphate insecti
Urinary bromophenol glucuronide and sulfate conjugates: Potential human exposure molecular markers for polybr
Environmental concentrations and toxicology of 2,4,6-tribromophenol (TBP).
Catalytic degradation of brominated flame retardants by copper oxide nanoparticles.
Cardiac sensitization testing of the halon replacement candidates trifluoroiodomethane (CF3I) and 1,1,2,2,3,3,3-he
Synthesis of tetrakis (hydroxymethyl) phosphonium chloride by high-concentration phosphine in industrial off-gas.
The mutagenic evaluation of tetrakis (hydroxymethyl) phosphonium sulfate using a combined testing protocol app
Synthesis of tetrakis (hydroxymethyl) phosphonium chloride by high-concentration phosphine in industrial off-gas.
The mutagenic evaluation of tetrakis (hydroxymethyl) phosphonium sulfate using a combined testing protocol app
Embryonic exposure to tetrabromobisphenol A and its metabolites, bisphenol A and tetrabromobisphenol A dimet
Detection of tetrabromobisphenol A and its mono- and dimethyl derivatives in fish, sediment and suspended parti
Transformation of tetrabromobisphenol A by Rhodococcus jostii RHA1: Effects of heavy metals.
Biotransformation of tetrabromobisphenol A dimethyl ether back to tetrabromobisphenol A in whole pumpkin pla
Dietary exposure of American kestrels (Falco sparverius) to decabromodiphenyl ether (BDE-209) flame retardant: u
Evaluation of Polybrominated Diphenyl Ether Toxicity on HepG2 Cells - Hexabrominated Congener (BDE-154) Is Les
Hepatic in vitro toxicity assessment of PBDE congeners BDE47, BDE153 and BDE154 in Atlantic salmon (Salmo sala
Polybrominated diphenyl ethers (PBDEs) in U.S. computers and domestic carpet vacuuming: possible sources of hu
The toxicology of the three commercial polybrominated diphenyl oxide (ether) flame retardants.
GC-EC analysis of polybrominated biphenyl constituents of Firemaster FF-1 using tetrabromobiphenyl as an interna
Oxidation of organic contaminants by manganese oxide geomedia for passive urban stormwater treatment system
Development and Validation of an Analytical Method to Quantitate Tris(chloroisopropyl)phosphate in Rat and Mou
Chlorinated ethyl and isopropyl phosphoric acid triesters in the indoor environment--an inter-laboratory exposure
The cytotoxicity of organophosphate flame retardants on HepG2, A549 and Caco-2 cells.
Concentrations and variability of organophosphate esters, halogenated flame retardants, and polybrominated dipl
A toxicogenomics approach to screen chlorinated flame retardants tris(2-chloroethyl) phosphate and tris(2-chloroi
Effects of tris(1,3-dichloro-2-propyl) phosphate and tris(1-chloropropyl) phosphate on cytotoxicity and mRNA expr
Neurotoxicity of two organophosphorus ester flame retardants in hens.
Chlorendic acid.
Biotransformation of hexabromocyclododecanes with hexachlorocyclohexane-transforming Sphingobium chinhate
Sublethal effects of the flame retardant intermediate hexachlorocyclopentadiene (HCCPD) on the gene transcriptio
2,3,7,8-Tetrachlorodibenzo-p-dioxin.
Carry-over of dietary organochlorine pesticides, PCDD/Fs, PCBs, and brominated flame retardants to Atlantic salmo
Desorption and bioavailability of spiked pentabromo diphenyl ether and tetrachlorodibenzo(p)dioxin in contamina
Antagonism of TCDD-induced ethoxyresorufin-O-deethylation activity by polybrominated diphenyl ethers (PBDEs)
Brominated organic contaminants in the liver and egg of the common cormorants (Phalacrocorax carbo) from Japa
Exposure to Environmental Contaminants and Lung Function in Adolescents-Is There a Link?
Psychological effects upon exposure to polyhalogenated dibenzodioxins and dibenzofurans.
Polychlorinated dibenzo-p-dioxins, dibenzofurans, and flame retardants in northern gannet (Morus bassanus) eggs
Detection of high PBDD/Fs levels and dioxin-like activity in toys using a combination of GC-HRMS, rat-based and hu
Assessment of characteristic distribution of PCDD/Fs and BFRs in sludge generated at municipal and industrial was
Facile synthesis of bromo- and mixed bromo/chloro dibenzo-p-dioxins and [14C]-labeled 1,3,7,8-tetrabromodibenz

Cross-omics gene and protein expression profiling in juvenile female mice highlights disruption of calcium and zinc
Steroid secretion following exposure of ovarian follicular cells to single congeners and defined mixture of polybron
A multilevel approach to predict toxicity in copepod populations: assessment of growth, genetics, and population s
Semivolatile organic compounds in homes: strategies for efficient and systematic exposure measurement based or
Effects of BDE-85 on the oxidative status and nerve conduction in rodents.
Penta- and octa-bromodiphenyl ethers promote proinflammatory protein expression in human bronchial epithelia
Physicochemical properties of selected polybrominated diphenyl ethers and extension of the UNIFAC model to bro
Technical pentabromodiphenyl ether and hexabromocyclododecane as activators of the pregnane-X-receptor (PXR
Ultrastructural changes observed in rat ovaries following in utero and lactational exposure to low doses of a polyb
A commercial mixture of the brominated flame retardant pentabrominated diphenyl ether (DE-71) induces respira
Differential expression of CYP1A, 2B, and 3A genes in the F344 rat following exposure to a polybrominated dipheny
Brief postnatal PBDE exposure alters learning and the cholinergic modulation of attention in rats.
Probing new approaches using atmospheric pressure photo ionization for the analysis of brominated flame retarda
Brominated flame retardants in tree bark from North America.
Brominated flame retardants in Alburnus alburnus from Cinca River Basin (Spain).
Exposure to flame retardants: nursing concern.
Effects of pentabrominated diphenyl ether (PBDE-99) on vitamin status in domestic duck (Anas platyrhynchos) hat
Developmental exposure to low dose PBDE 99: effects on male fertility and neurobehavior in rat offspring.
Polybrominated diphenyl ethers (PBDEs) in U.S. computers and domestic carpet vacuuming: possible sources of hu
Neurotoxicity of the pentabrominated diphenyl ether mixture, DE-71, and hexabromocyclododecane (HBCD) in rat
In vivo and in vitro Ah-receptor activation by commercial and fractionated pentabromodiphenylether using zebrafi
Salmon flame retardant research raises new questions.
Tissue disposition, excretion and metabolism of 2,2',4,4',5-pentabromodiphenyl ether (BDE-99) in the male Spragu
Comparison of analytical strategies for the chromatographic and mass spectrometric measurement of brominated
Effects of selected polybrominated diphenyl ether flame retardants on lake trout (Salvelinus namaycush).
Exposure to polybrominated diphenyl ethers (PBDEs): changes in thyroid, vitamin A, glutathione homeostasis, and
Polybrominated diphenyl ether (PBDE)-induced alterations in vitamin A and thyroid hormone concentrations in the
New multiresidue analytical method dedicated to trace level measurement of brominated flame retardants in hum
Proteomic evaluation of neonatal exposure to 2,2 ,4,4 ,5-pentabromodiphenyl ether.
Dietary accumulation efficiencies and biotransformation of polybrominated diphenyl ethers in farmed Atlantic saln
Differential effects of polybrominated diphenyl ethers and polychlorinated biphenyls on [3H]arachidonic acid relea
Effects of perinatal exposure to a polybrominated diphenyl ether (PBDE 99) on mouse neurobehavioural developm
The expression of CYP1A, vitellogenin and zona radiata proteins in Atlantic salmon (Salmo salar) after oral dosing w
Levels of polybrominated diphenyl ether (PBDE) flame retardants in animals representing different trophic levels o
Flame retardant exposure: polybrominated diphenyl ethers in blood from Swedish workers.
The toxicology of the three commercial polybrominated diphenyl oxide (ether) flame retardants.
A brominated flame retardant, 2,2',4,4',5-pentabromodiphenyl ether: uptake, retention, and induction of neurobe
Neonatal exposure to the brominated flame retardant 2,2',4,4',5-pentabromodiphenyl ether causes altered suscep
PBDE information overlooked?
Preventing fires, igniting questions.
Some polybrominated diphenyl ether (PBDE) flame retardants with wide environmental distribution inhibit TCDD-i
Occurrence and bioavailability of polybrominated diphenyl ethers and hexabromocyclododecane in sediment and
Temporal trends and spatial distributions of brominated flame retardants in archived fishes from the Great Lakes.
Impact of fermented brown rice with Aspergillus oryzae (FEBRA) intake and concentrations of polybrominated dipl
Clarifications on PBDE flame retardants.
Occurrence of polychlorinated biphenyls and polybrominated diphenyl ethers in green mussels (Perna viridis) from
[Flame retardants--use and hazards for human].

U.S. PBDE milestones.
Deca PBDE flame retardant gets around.
Screening of halogenated aromatic compounds in some raw material lots for an aluminium recycling plant.
Developmental neurotoxicity of polybrominated diphenyl ethers mixture de71 in Sprague-Dawley rats.
Propelling plastics into the circular economy - weeding out the toxics first.
Geographical distribution of non-PBDE-brominated flame retardants in mussels from Asian coastal waters.
Effects of the Commercial Flame Retardant Mixture DE-71 on Cytokine Production by Human Immune Cells.
Exposure to polybrominated diphenyl ethers and female reproductive function: A study in the production area of S
Comparative Study of Genotoxicity Induced by Six Different PBDEs.
The environmental fate of polybrominated diphenyl ethers (PBDEs) in western Taiwan and coastal waters: evaluati
DNA Methylation Changes in Tbx3 in a Mouse Model Exposed to Polybrominated Diphenyl Ethers.
Effect of smoking and caffeine consumption on polybrominated diphenyl ethers (PBDE) and polybrominated biphe
Polybrominated diphenyl ethers alter hepatic phosphoenolpyruvate carboxykinase enzyme kinetics in male Wistar
Temporal trends of PBDEs and emerging flame retardants in belugas from the St. Lawrence Estuary (Canada) and c
Novel and high volume use flame retardants in US couches reflective of the 2005 PentaBDE phase out.
After the PBDE phase-out: a broad suite of flame retardants in repeat house dust samples from California.
Porphyrogenic effect of pentabromodiphenyl ether after repeated administration to rats.
Differences in neonatal neurotoxicity of brominated flame retardants, PBDE 99 and TBBPA, in mice.
Epigenetic Effects of Polybrominated Diphenyl Ethers on Human Health.
Trophic level determines levels of brominated flame-retardants in coastal herring gulls.
More signs of neurotoxicity of surfactants and flame retardants - Neonatal PFOS and PBDE 99 cause transcriptiona
Associations between PBDEs in office air, dust, and surface wipes.
Alterations to the circuitry of the frontal cortex following exposure to the polybrominated diphenyl ether mixture,
Prenatal transfer of polybrominated diphenyl ethers (PBDEs) results in developmental neurotoxicity in zebrafish la
Associations between serum levels of polybrominated diphenyl ether (PBDE) flame retardants and environmental
Effects of chronic exposure to an environmentally relevant mixture of brominated flame retardants on the reprodu
Acute postnatal exposure to the pentaBDE commercial mixture DE-71 at 5 or 15 mg/kg/day does not produce lear
Tissue-specific accumulation of polybrominated diphenyl ethers (PBDEs) including Deca-BDE and hexabromocycloc
Immune function in female B(6)C(3)F(1) mice is modulated by DE-71, a commercial polybrominated diphenyl ethe
Detection of polybrominated biphenyl ethers (PBDEs) in pediatric hair as a tool for determining in utero exposure.
The flame retardant DE-71 (a mixture of polybrominated diphenyl ethers) inhibits human differentiated thyroid ce
Lack of effects of some individual polybrominated diphenyl ether (PBDE) and polychlorinated biphenyl (PCB) conge
The occurrence of polybrominated diphenyl ether (PBDE) contamination in soil, water/sediment, and air.
Fetal exposure to polybrominated diphenyl ethers and the risk of hypospadias: focus on the congeners involved.
Brominated flame retardants in animal derived foods in the Netherlands between 2009 and 2014.
Polybrominated diphenyl ethers (PBDEs) in chicken eggs and cow milk around municipal dumpsites in Abuja, Niger
Decreasing but still high levels of halogenated flame retardants in wetland birds in central Spain.
Concentrations and loadings of organophosphate and replacement brominated flame retardants in house dust fro
Single-cell RNA-sequencing analysis of estrogen- and endocrine-disrupting chemical-induced reorganization of mo
Immunologic and endocrine effects of the flame-retardant pentabromodiphenyl ether (DE-71) in C57BL/6J mice.
A noninvasive environmental monitoring tool for brominated flame-retardants (BFRs) assisted by conservation det
Racial/ethnic and geographic differences in polybrominated diphenyl ether (PBDE) levels across maternal, placenta
Polybrominated biphenyl and diphenylether flame retardants: analysis, toxicity, and environmental occurrence.
[Reproductive toxicity of PBDE in males: Advances in studies].
Concentrations of legacy and novel brominated flame retardants in indoor dust in Melbourne, Australia: An assess
Dietary exposure to a binary mixture of polybrominated diphenyl ethers alters innate immunity and disease susce
Distribution of polybrominated diphenyl ethers in the atmosphere of the Pearl River Delta region, South China.

Temporal trends and developmental patterns of plasma polybrominated diphenyl ether concentrations over a 15-y
Polybrominated diphenyl ethers (PBDEs) and hydroxylated PBDE metabolites (OH-PBDEs): A six-year temporal trer
Multi-analyte method development for analysis of brominated flame retardants (BFRs) and PBDE metabolites in hu
Pine needles as biomonitors of polybrominated diphenyl ethers and emerging flame retardants in the atmosphere
The brominated flame retardant PBDE 99 promotes adipogenesis via regulating mitotic clonal expansion and PPAR
A preliminary study on prenatal polybrominated diphenyl ether serum concentrations and intrinsic functional netv
Species and habitat-dependent accumulation and biomagnification of brominated flame retardants and PBDE met
Prenatal polybrominated diphenyl ethers exposure and anogenital distance in boys from a Shanghai birth cohort.
Occurrence, levels and profiles of brominated flame retardants in daily-use consumer products on the Chinese ma
Association of In Utero Exposure to Polybrominated Diphenyl Ethers With the Risk of Hypospadias.
DE-71-induced apoptosis involving intracellular calcium and the Bax-mitochondria-caspase protease pathway in hu
Flame retardants (PBDEs) in marine turtles, dugongs and seafood from Queensland, Australia.
Desorption and bioavailability of spiked pentabromo diphenyl ether and tetrachlorodibenzo(p)dioxin in contamina
Brominated flame retardants in the environment of Asia-Pacific: an overview of spatial and temporal trends.
Electrolytic debromination of PBDEs in DE-83 technical decabromodiphenyl ether.
A 28-day oral dose toxicity study enhanced to detect endocrine effects of a purified technical pentabromodipheny
Polybrominated diphenyl ethers as endocrine disruptors of adipocyte metabolism.
Spatial trends of polybrominated diphenyl ethers in avian species: utilization of stored samples in the Environment
Neurotoxicity of a polybrominated diphenyl ether mixture (DE-71) in mouse neurons and astrocytes is modulated
Exposure to brominated flame retardant PBDE-99 affects cytoskeletal protein expression in the neonatal mouse ce
Bioaccumulation behaviour of polybrominated diphenyl ethers (PBDEs) in a Canadian Arctic marine food web.
Measurement of polybrominated diphenyl ethers on hand wipes: estimating exposure from hand-to-mouth contac
Detection of PBDE effects on mRNA expression in chicken (Gallus domesticus) neuronal cells using real-time RT-PC
Coexposure of neonatal mice to a flame retardant PBDE 99 (2,2',4,4',5-pentabromodiphenyl ether) and methyl me
Polybrominated diphenyl ether in sewage sludge in Germany.
Plasma PBDE and thyroxine levels in rats exposed to Bromkal or BDE-47.
Accumulation, tissue-specific distribution and debromination of decabromodiphenyl ether (BDE 209) in European
PBDEs in circuit boards.
Brominated flame retardants and halogenated phenolic compounds in North American west coast bald eaglet (Hal
Congener distribution of polybrominated diphenyl ethers in feral carp (Cyprinus carpio) from the Llobregat River, S
Polybrominated diphenyl ethers, a group of brominated flame retardants, can interact with polychlorinated bipher
Linking PBDEs in house dust to consumer products using X-ray fluorescence.
Decabromodiphenyl ether (deca-BDE) commercial mixture components, and other PBDEs, in airborne particles at
Flame retardants in placenta and breast milk and cryptorchidism in newborn boys.
Geographical distribution and accumulation features of PBDEs in human breast milk from Indonesia.
The risk of PBDEs in dust.
The flame retardants, polybrominated diphenyl ethers, are pregnane X receptor activators.
Variation, levels and profiles of organochlorines and brominated flame retardants in great tit (Parus major) eggs fr
Individual characteristics associated with PBDE levels in U.S. human milk samples.
Toxicity of penta- and decabromodiphenyl ethers after repeated administration to rats: a comparative study.
Reductive debromination of polybrominated diphenyl ethers by anaerobic bacteria from soils and sediments.
Multi-generational effects of polybrominated diphenylethers exposure: embryonic exposure of male American kes
Toxicity assessment of air-delivered particle-bound polybrominated diphenyl ethers.
Thyroid insult: flame retardants linked to alterations in pregnant women's TSH levels.
Polybrominated diphenyl ether (PBDE) levels in peregrine falcon (Falco peregrinus) eggs from California correlate w
Polychlorinated biphenyls and polybrominated diphenyl ethers alter striatal dopamine neurochemistry in synaptos
PBDE exposure from food in Ireland: optimising data exploitation in probabilistic exposure modelling.

Brominated diphenyl ether (BDE) levels in liver, adipose, and milk from adult and juvenile rats exposed by gavage t
Flame retardants have different effects at high and low doses.
The effect of short-term intoxication of rats with pentabromodiphenyl ether (in mixture mimic commercial produc
[Preliminary study of PBDE levels in house dust and human exposure to PBDEs via dust ingestion].
Toxicity of polybrominated diphenyl ethers (DE-71) in chicken (Gallus gallus), mallard (Anas platyrhynchos), and An
Polybrominated diphenyl ether (PBDE) concentrations in house dust are related to hormone levels in men.
The effects of marginal maternal vitamin A status on penta-brominated diphenyl ether mixture-induced alterations
Chronic postnatal DE-71 exposure: effects on learning, attention and thyroxine levels.
Spending time in vehicles can increase PBDE exposure.
Levels and congener specific profiles of PBDEs in human breast milk from China: implication on exposure sources a
Reproductive and developmental toxicity of a pentabrominated diphenyl ether mixture, DE-71, to ranch mink (Mu
Effects of perinatal PBDE exposure on hepatic phase I, phase II, phase III, and deiodinase 1 gene expression involve
In vitro neurotoxicity of PBDE-99: immediate and concentration-dependent effects on protein expression in cerebr
Exposure to DE-71 alters thyroid hormone levels and gene transcription in the hypothalamic-pituitary-thyroid axis
3D-map modelling for the melting points prediction of intumescent flame-retardant coatings.
The Efficiency of Biobased Carbonization Agent and Intumescent Flame Retardant on Flame Retardancy of Biopoly
Dechloranes 602, 603, 604, Dechlorane Plus, and Chlordene Plus, a newly detected analogue, in tributary sedimen
Hepatic changes in rats following subchronic administration of FYROL 6, an organophosphorus ester flame retardan
Cross-omics gene and protein expression profiling in juvenile female mice highlights disruption of calcium and zinc
Use of a simple pharmacokinetic model to study the impact of breast-feeding on infant and toddler body burdens
Toxic effect of PBDE-47 on thyroid development, learning, and memory, and the interaction between PBDE-47 and
Some of the properties of flame retardant medium density fiberboard made from rubberwood and recycled conta
Graphene oxide/cellulose aerogels nanocomposite: Preparation, pyrolysis, and application for electromagnetic inte
Preparation and application of graphene oxide-based surface molecularly imprinted polymer for monolithic fiber a
Combinatorial immune and stress response, cytoskeleton and signal transduction effects of graphene and tripheny
Magnetic solid-phase extraction of brominated flame retardants from environmental waters with graphene-doped
Lignin-Modified Carbon Nanotube/Graphene Hybrid Coating as Efficient Flame Retardant.
Reproductive toxicity screen of trifluoroiodomethane (CF(3)I) in Sprague-Dawley rats.
Cardiac sensitization testing of the halon replacement candidates trifluoroiodomethane (CF3I) and 1,1,2,2,3,3,3-he
Is BDE-175 an important enough component of commercial octabromodiphenyl ether mixtures to be listed in Ann
Accumulation and debromination of decabromodiphenyl ether (BDE-209) in juvenile fathead minnows (Pimephale
Metabolic activation of tris(2,3-dibromopropyl)phosphate to reactive intermediates. I. Covalent binding and reacti
Metabolism in vitro of tris(2,3-dibromopropyl)-phosphate: oxidative debromination and bis(2,3-dibromopropyl)ph
Comparative studies on nephrotoxic effects of tris (2,3-dibromopropyl) phosphate and bis (2,3-dibromopropyl) ph
Semivolatile organic compounds in homes: strategies for efficient and systematic exposure measurement based on
Toxicity assessment and vitellogenin expression in zebrafish (Danio rerio) embryos and larvae acutely exposed to b
Peroxisome proliferator-activated receptor ? is a target for halogenated analogs of bisphenol A.
Stimulation of ovarian cell proliferation by tetrabromobisphenol A but not tetrachlorobisphenol A through G prote
Oxidative stress and cytotoxicity induced by tetrachlorobisphenol A in Saccharomyces cerevisiae cells.
Determination of tetrabromobisphenol-A, tetrachlorobisphenol-A and bisphenol-A in soil by ultrasonic assisted ext
Anti-thyroid hormone activity of bisphenol A, tetrabromobisphenol A and tetrachlorobisphenol A in an improved r
Thyroid hormonal activity of the flame retardants tetrabromobisphenol A and tetrachlorobisphenol A.
Comparison of the oxidation products produced by tetrahalobisphenol A flame retardants as a result of potassium
Metabolic perturbation, proliferation and reactive oxygen species jointly contribute to cytotoxicity of human breas
Reductive dechlorination of tetrachlorobisphenol A by Pd/Fe bimetallic catalysts.
Toxicogenomic analyses of the effects of BDE-47/209, TBBPA/S and TCBPA on early neural development with a hur
Anaerobic biotransformation of tetrabromobisphenol A, tetrachlorobisphenol A, and bisphenol A in estuarine sedi

Warm reception? Halogenated BPA flame retardants and PPAR? activation.
The Toxic Effects of Tetrachlorobisphenol A in Saccharomyces cerevisiae Cells via Metabolic Interference.
Assessment of organophosphate flame retardants in Mediterranean Boops boops and their relationship to anthro[illegible]
Uptake and translocation of organophosphates and other emerging contaminants in food and forage crops.
Toxicokinetic patterns, metabolites formation and distribution in various tissues of the Chinese rare minnow (Gobi[illegible]
Organophosphorus Flame Retardants and Plasticizers in Building and Decoration Materials and Their Potential Bur[illegible]
Distribution, source apportionment and ecological risks of organophosphate esters in surface sediments from the [illegible]
Developmental exposure of zebrafish larvae to organophosphate flame retardants causes neurotoxicity.
Comparative assessment of neurotoxicity impacts induced by alkyl tri-n-butyl phosphate and aromatic tricresyl ph[illegible]
Distribution Pattern of Organophosphate Esters in Particle-Size Fractions of Urban Topsoils Under Different Land-U[illegible]
Comparative body compartment composition and in ovo transfer of organophosphate flame retardants in North A[illegible]
Occurrence of organic phosphates in particulate matter of the vehicle exhausts and outdoor environment - A case [illegible]
Concentrations and variability of organophosphate esters, halogenated flame retardants, and polybrominated dip[illegible]
Tissue-specific distribution and bioaccumulation potential of organophosphate flame retardants in crucian carp.
Organophosphate ester flame retardant concentrations and distributions in serum from inhabitants of Shandong, [illegible]
The cytotoxicity of organophosphate flame retardants on HepG2, A549 and Caco-2 cells.
Concentrations and distributions of polybrominated diphenyl ethers and novel brominated flame retardants in tre[illegible]
Novel brominated flame retardant (NBFR) concentrations and spatial distributions in global fishmeal.
Presence and partitioning properties of the flame retardants pentabromotoluene, pentabromoethylbenzene and h[illegible]
Chlorinated ethyl and isopropyl phosphoric acid triesters in the indoor environment--an inter-laboratory exposure [illegible]
Development and Validation of an Analytical Method to Quantitate Tris(chloroisopropyl)phosphate in Rat and Mou[illegible]
Oxidation of organic contaminants by manganese oxide geomedia for passive urban stormwater treatment system[illegible]
The cytotoxicity of organophosphate flame retardants on HepG2, A549 and Caco-2 cells.
A toxicogenomics approach to screen chlorinated flame retardants tris(2-chloroethyl) phosphate and tris(2-chloroi[illegible]
Concentrations and variability of organophosphate esters, halogenated flame retardants, and polybrominated dip[illegible]
Neurotoxicity of two organophosphorus ester flame retardants in hens.
Effects of tris(1,3-dichloro-2-propyl) phosphate and tris(1-chloropropyl) phosphate on cytotoxicity and mRNA expr[illegible]
Role of glutamate receptors in tetrabrominated diphenyl ether (BDE-47) neurotoxicity in mouse cerebellar granule[illegible]
Isoliquiritigenin as an antioxidant phytochemical ameliorates the developmental anomalies of zebrafish induced b[illegible]
Glutathione-Ascorbate Cycle Is an Early Warning Indicator of Toxicity of BDE-47 in Mangroves.
Proteomic and metabolomic analysis of earthworm Eisenia fetida exposed to different concentrations of 2,2',4,4'-t[illegible]
Absorption of [14C]-tetrabromodiphenyl ether (TeBDE) through human and rat skin in vitro.
Lethal and sublethal effects of simvastatin, irgarol, and PBDE-47 on the estuarine fish, Fundulus heteroclitus.
Low concentrations of the brominated flame retardants BDE-47 and BDE-99 induce synergistic oxidative stress-me[illegible]
Protective effect of (±)?-tocopherol on brominated diphenyl ether-47-stimulated prostaglandin pathways in huma[illegible]
Multiyear Measurements of Flame Retardants and Organochlorine Pesticides in Air in Canada's Western Sub-Arcti[illegible]
Protective effect of nuclear factor E2-related factor 2 on inflammatory cytokine response to brominated diphenyl [illegible]
In vitro immune toxicity of polybrominated diphenyl ethers on murine peritoneal macrophages: apoptosis and im[illegible]
Semivolatile organic compounds in homes: strategies for efficient and systematic exposure measurement based o[illegible]
Studies on the interaction of BDE-47 and BDE-209 with acetylcholinesterase (AChE) based on the neurotoxicity thr[illegible]
PBDEs and novel brominated flame retardants in road dust from northern Vietnam: Levels, congener profiles, emi[illegible]
Polybrominated diphenyl ethers (PBDEs) and hydroxylated PBDE metabolites (OH-PBDEs): A six-year temporal tre[illegible]
Developmental exposure to low concentrations of two brominated flame retardants, BDE-47 and BDE-99, causes l[illegible]
Profiling of Selected Functional Metabolites in the Central Nervous System of Marine Medaka (Oryzias melastigma[illegible]
Brominated and organophosphate flame retardants target different neurodevelopmental stages, characterized wit[illegible]
Legacy and alternative halogenated flame retardants in human milk in Europe: Implications for children's health.
Concentrations of legacy and novel brominated flame retardants in indoor dust in Melbourne, Australia: An asses[illegible]

Occurrence of polybrominated diphenylethers, hexabromocyclododecanes, bromophenols and tetrabromobispher
Effects of 2,2',4,4'-tetrabromodiphenyl ether on neurobehavior and memory change and bcl-2, c-fos, grin1b and lir
Use of a simple pharmacokinetic model to study the impact of breast-feeding on infant and toddler body burdens
Perinatal exposure to 2,2',4'4' -Tetrabromodiphenyl ether induces testicular toxicity in adult rats.
Emerging and legacy flame retardants in UK human milk and food suggest slow response to restrictions on use of F
Serum levels of brominated flame retardants (BFRs: PBDE, HBCD) and influence of dietary factors in a population-b
Spatial and temporal trends of alternative flame retardants and polybrominated diphenyl ethers in ringed seals (Ph
Polybrominated diphenyl ethers (PBDES) and hexa-brominated biphenyls (Hexa-BBs) in fresh foods ingested in Taiw
Spatial Distribution of Organophosphorus and Brominated Flame Retardants in Surface Water, Sediment, Groundw
Applicability of Gas Chromatography (GC) Coupled to Triple-Quadrupole (QqQ) Tandem Mass Spectrometry (MS/M
Widespread polybrominated diphenyl ether (PBDE) contamination of urban soils in Melbourne, Australia.
The flame retardant 2,2',4,4'-Tetrabromodiphenyl ether enhances the expression of corticotropin-releasing hormo
PBDEs in cod (Gadus morhua) liver products (1972-2017): Occurrence and human exposure.
A brominated flame retardant 2,2',4,4' tetrabrominated diphenyl ether (BDE-47) leads to lipogenesis in the copepo
2,2',4,4'-tetrabromodiphenyl ether (BDE-47) induces wide metabolic changes including attenuated mitochondrial f
Debromination of polybrominated diphenyl ethers (PBDEs) by palladized zerovalent zinc particles: Influence factor
Accumulation and Transformation of 2,2',4,4'-Tetrabrominated Diphenyl Ether (BDE47) by the Earthworm Metaphi
Transcriptomic profiling of PBDE-exposed HepaRG cells unveils critical lncRNA- PCG pairs involved in intermediary r
"Waste"-ing away: Presence of Cu ions influences microbial degradation kinetics and metabolite formation of the p
Toxicological, gene expression and histopathological evaluations of environmentally realistic concentrations of poly
Molecular mechanisms and tissue targets of brominated flame retardants, BDE-47 and TBBPA, in embryo-larval life
Brominated flame retardants in animal derived foods in the Netherlands between 2009 and 2014.
Spatiotemporal variability of polybrominated diphenyl ether concentration in atmospheric fine particles in Shenzh
The brominated flame retardant BDE 47 upregulates purine metabolism and mitochondrial respiration to promote
Biomineralization of 2'2'4'4'-Tetrabromodiphenyl ether in a Pseudomonas putida and Fe/Pd nanoparticles integrat
New brominated flame retardants and dechlorane plus in the Arctic: Local sources and bioaccumulation potential
Genomic Profiling of BDE-47 Effects on Human Placental Cytotrophoblasts.
Waterborne exposure to low concentrations of BDE-47 impedes early vascular development in zebrafish embryos/
Seasonal variations of polybrominated flame retardants bound to car dust under Mediterranean climate.
Embryo-larval BDE-47 exposure causes decreased pathogen resistance in adult male fathead minnows (Pimephale
Toxicogenomic analyses of the effects of BDE-47/209, TBBPA/S and TCBPA on early neural development with a hur
Toxic effects of two brominated flame retardants BDE-47 and BDE-183 on the survival and protein expression of th
Neurochemical changes following a single dose of polybrominated diphenyl ether 47 in mice.
Comparative oxidative metabolism of BDE-47 and BDE-99 by rat hepatic microsomes.
Acute toxicity of polybrominated diphenyl ethers (PBDEs) for turbot (Psetta maxima) early life stages (ELS).
Toxicogenomic mechanisms of 6-HO-BDE-47, 6-MeO-BDE-47, and BDE-47 in E. coli.
Levels of dechlorane plus and polybrominated diphenylethers in human milk in two Canadian cities.
Cyto-genotoxic effects induced by three brominated diphenyl ether congeners on the freshwater mussel Dreissena
Spatial learning and memory deficit of low level polybrominated diphenyl ethers-47 in male adult rat is modulated
Improved measurements of partition coefficients for polybrominated diphenyl ethers.
Evaluation of DNA damage induced by 2 polybrominated diphenyl ether flame retardants (BDE-47 and BDE-209) in
Toxic effect of PBDE-47 on thyroid development, learning, and memory, and the interaction between PBDE-47 and
Assessment of the levels of polybrominated diphenyl ethers in blood samples from Guadalajara, Jalisco, Mexico.
Genotoxicity and development effects of brominated flame retardant PBDEs and UV-exposed PBDEs on grass shrin
Experimental exposure of eggs to polybrominated diphenyl ethers BDE-47 and BDE-99 in red-eared sliders (Trache
Biotransformation of BDE-47 to potentially toxic metabolites is predominantly mediated by human CYP2B6.
Effects of dietary exposure to brominated flame retardant BDE-47 on thyroid condition, gonadal development and

Study of the toxic effects of flame retardant PBDE-47 on the clam Chamelea gallina (Linnaeus, 1758).
A hydroxylated metabolite of flame-retardant PBDE-47 decreases the survival, proliferation, and neuronal differen
Hazards of low dose flame-retardants (BDE-47 and BDE-32): Influence on transcriptome regulation and cell death i
Polybrominated diphenyl ethers disrupt molting in neonatal Daphnia magna.
Toxicity and physiological effects of brominated flame retardant PBDE-47 on two life stages of grass shrimp, Palaer
Toxicokinetics of BDE 47 in female mice: effect of dose, route of exposure, and time.
Disposition of BDE 47 in developing mice.
Congener distribution of polybrominated diphenyl ethers in feral carp (Cyprinus carpio) from the Llobregat River, S
Plasma PBDE and thyroxine levels in rats exposed to Bromkal or BDE-47.
The flame retardants, polybrominated diphenyl ethers, are pregnane X receptor activators.
Geographical distribution and accumulation features of PBDEs in human breast milk from Indonesia.
Spatial trends of polybrominated diphenyl ethers in avian species: utilization of stored samples in the Environment
A dynamic multimedia environmental and bioaccumulation model for brominated flame retardants in Lake Huron
Temporal trends of polybrominated diphenyl ethers and hexabromocyclododecane in milk from Stockholm mothe
Locomotor activity changes on zebrafish larvae with different 2,2',4,4'-tetrabromodiphenyl ether (PBDE-47) embry
Polymeric brominated flame retardants: are they a relevant source of emerging brominated aromatic compounds
Determination of environmentally relevant exposure concentrations of polybrominated diphenyl ethers for in vitro
Mixture of dominant PBDE congeners (BDE-47, -99, -100 and -209) at levels noted in human blood dramatically en
The cytotoxic effects of synthetic 6-hydroxylated and 6-methoxylated polybrominated diphenyl ether 47 (BDE47).
Vibrational spectroscopic investigation and DFT studies on 2,2',4,4'-tetrabromodiphenyl ether.
Does 2,2',4,4'-tetrabromodiphenyl ether interact directly with thyroid receptor?
Consumption of fish from a contaminated lake strongly affects the concentrations of polybrominated diphenyl eth
Observation-Based Assessment of PBDE Loads in Arctic Ocean Waters.
Histopathological effects of 2,2',4,4'-tetrabromodiphenyl ether (BDE-47) in the gills, intestine and liver of turbot (P
2,2',4,4'-Tetrabromodiphenyl ether disrupts spermatogenesis, impairs mitochondrial function and induces apoptos
Effect of omega-3 fatty acid oxidation products on the cellular and mitochondrial toxicity of BDE 47.
Multiple biomarkers of the cytotoxicity induced by BDE-47 in human embryonic kidney cells.
Changes in antitoxic defense systems of the freshwater amphipod Gammarus pulex exposed to BDE-47 and BDE-9
The brominated flame retardant BDE-47 causes oxidative stress and apoptotic cell death in vitro and in vivo in mic
Declines in polybrominated diphenyl ether contamination of San Francisco Bay following production phase-outs ar
Evaluation of hepatic biotransformation of polybrominated diphenyl ethers in the polar bear (Ursus maritimus).
Does exposure to flame retardants increase the risk for preterm birth?
Occurrence and levels of polybrominated diphenyl ethers in surface sediments from the Yellow River Estuary, Chin
The environmental fate of polybrominated diphenyl ethers (PBDEs) in western Taiwan and coastal waters: evaluati
Comparative Study of Genotoxicity Induced by Six Different PBDEs.
Evaluation of Polybrominated Diphenyl Ether Toxicity on HepG2 Cells - Hexabrominated Congener (BDE-154) Is Les
Effects of Polybrominated Diphenyl Ethers on Rat and Human 11?-Hydroxysteroid Dehydrogenase 1 and 2 Activitie
[Research progress of health effect of polybrominated diphenyl ethers].
Environmental exposure to BDE47 is associated with increased diabetes prevalence: Evidence from community-ba
BDE-99, but not BDE-47, is a transient aryl hydrocarbon receptor agonist in zebrafish liver cells.
Cross-omics gene and protein expression profiling in juvenile female mice highlights disruption of calcium and zinc
Gender-specific metabolic responses in gonad of mussel Mytilus galloprovincialis to 2,2',4,4'-tetrabromodiphenyl e
An efficient GC-IDMS method for determination of PBDEs and PBB in plastic materials.
Modeling human off-site aerosol exposures to polybrominated flame retardants emitted during the land applicatio
Effects of perchlorate on BDE-47-induced alteration thyroid hormone and gene expression of in the hypothalamus
Flame retardant BDE-47 effectively activates nuclear receptor CAR in human primary hepatocytes.
Transformation of 2,2',4,4'-tetrabromodiphenyl ether under UV irradiation: potential sources of the secondary pol

Brominated flame retardant concentrations in sera from the Canadian Health Measures Survey (CHMS) from 2007
Polybrominated diphenyl ethers and alternative flame retardants in air and precipitation samples from the northe
Toxicity assessment of air-delivered particle-bound polybrominated diphenyl ethers.
Phytotoxicity of brominated diphenyl ether-47 (BDE-47) and its hydroxylated and methoxylated analogues (6-OH-E
Transcriptional and epigenetic mechanisms underlying enhanced in vitro adipocyte differentiation by the bromina
Pharmacokinetic bias analysis of the epidemiological associations between serum polybrominated diphenyl ether
Patterns and trends in brominated flame retardants in bald eagle nestlings from the upper midwestern United Sta
Effects of the bioaccumulative polybrominated diphenyl ether flame retardant congener BDE-47 on growth, devel
Hair as a biomarker of systemic exposure to polybrominated diphenyl ethers.
Polybrominated diphenyl ethers (PBDEs) in the indoor dust in China: levels, spatial distribution and human exposu
Epigenetic effects of low perinatal doses of flame retardant BDE-47 on mitochondrial and nuclear genes in rat offs
Investigation of polybrominated diphenyl ethers in old consumer products in India.
Exposure to polybrominated diphenyl ethers and male reproductive function in Greenland, Poland and Ukraine.
Facilitated Leaching of Additive-Derived PBDEs from Plastic by Seabirds' Stomach Oil and Accumulation in Tissues.
Methods for synthesis of nonabromodiphenyl ethers and a chloro-nonabromodiphenyl ether.
Enhanced debromination of 4-bromophenol by the UV/sulfite process: Efficiency and mechanism.
Accumulation and debromination of decabromodiphenyl ether (BDE-209) in juvenile fathead minnows (Pimephale
Is BDE-175 an important enough component of commercial octabromodiphenyl ether mixtures to be listed in Ann
Melamine and its derivatives in dog and cat urine: An exposure assessment study.
Simultaneous determination of melamine, ammelide, ammeline, and cyanuric acid in milk and milk products by ga
Aluminum hypophosphite microencapsulated to improve its safety and application to flame retardant polyamide 6
Surface nanomodification of cotton fiber for flame retardant application.
[Use of zirconium phosphate as a fire-proof material in dental casting].
Pyrolysis of waste electrical and electronic equipment: effect of antinomy trioxide on the pyrolysis of styrenic poly
Biomonitoring of a worker population exposed to low antimony trioxide levels.
Asbestos between science and myth. A 6,000-year story.
Exposure to airborne asbestos associated with simulated cable installation above a suspended ceiling.
Technical pentabromodiphenyl ether and hexabromocyclododecane as activators of the pregnane-X-receptor (PXR
Hexabromocyclododecane inhibits depolarization-induced increase in intracellular calcium levels and neurotransm
Effects of metals on the transformation of hexabromocyclododecane (HBCD) in solvents: implications for solvent-b
Modulation at a cellular level of the thyroid hormone receptor-mediated gene expression by 1,2,5,6,9,10-hexabro
Analysis of brominated flame retardants in styrenic polymers. Comparison of the extraction efficiency of ultrasoni
Analysis of hexabromocyclododecane diastereomers and enantiomers by liquid chromatography/tandem mass spe
Transformation of hexabromocyclododecane in contaminated soil in association with microbial diversity.
Solvent effects on quantitative analysis of brominated flame retardants with Soxhlet extraction.
Dermal bioaccessibility of flame retardants from indoor dust and the influence of topically applied cosmetics.
Antioxidant gene expression and metabolic responses of earthworms (Eisenia fetida) after exposure to various co
Acute effects of hexabromocyclododecane on Leydig cell cyclic nucleotide signaling and steroidogenesis in vitro.
Responses of growth inhibition and antioxidant gene expression in earthworms (Eisenia fetida) exposed to tetrabr
Developmental toxicity of brominated flame retardants, tetrabromobisphenol A and 1,2,5,6,9,10-hexabromocyclo
Personal exposure to HBCDs and its degradation products via ingestion of indoor dust.
Hexabromocyclododecane-induced developmental toxicity and apoptosis in zebrafish embryos.
Bioaccumulation of polybrominated diphenyl ethers and hexabromocyclododecane in the northwest Atlantic mari
Hexabromocyclododecane in human breast milk: levels and enantiomeric patterns.
Proteomic studies in zebrafish liver cells exposed to the brominated flame retardants HBCD and TBBPA.
Brominated flame retardants in fish of Lake Geneva (Switzerland).
Isolation of Pseudomonas sp. strain HB01 which degrades the persistent brominated flame retardant gamma-hexa

Hexabromocyclododecane decreases the lytic function and ATP levels of human natural killer cells.
Temporal trends, congener patterns, and sources of octa-, nona-, and decabromodiphenyl ethers (PBDE) and hexal
Determination of brominated flame retardants in food by LC-MS/MS: diastereoisomer-specific hexabromocyclodo
Brominated flame retardants and perfluorinated chemicals, two groups of persistent contaminants in Belgian hum
Cytotoxicity evaluation of three pairs of hexabromocyclododecane (HBCD) enantiomers on Hep G2 cell.
Transfer of brominated flame retardants from components into dust inside television cabinets.
Sediment record and atmospheric deposition of brominated flame retardants and organochlorine compounds in La
Effects of the brominated flame retardant hexabromocyclododecane (HBCD) on dopamine-dependent behavior ar
Endocrine effects of hexabromocyclododecane (HBCD) in a one-generation reproduction study in Wistar rats.
Determination of flame-retardant hexabromocyclododecane diastereomers in textiles.
Assessing the toxicity of TBBPA and HBCD by zebrafish embryo toxicity assay and biomarker analysis.
New perspective on the determination of flame retardants in sewage sludge by using ultrahigh pressure liquid chro
Trophodynamics of hexabromocyclododecanes and several other non-PBDE brominated flame retardants in a fresh
Thermally-induced transformation of hexabromocyclo dodecanes and isobutoxypenta bromocyclododecanes in fla
Toxicokinetics of the flame retardant hexabromocyclododecane gamma: effect of dose, timing, route, repeated ex
Bioavailability of hexabromocyclododecane to the polychaete Hediste diversicolor: exposure through sediment an
Hexabromocyclododecane determination in seafood samples collected from Japanese coastal areas.
Simultaneous determination of hexabromocyclododecanes and tris (2,3-dibromopropyl) isocyanurate using LC-AP(
The potential of selected brominated flame retardants to affect neurological development.
Polybrominated diphenyl ethers (PBDEs) and hexabromocyclodecane (HBCD) in composite U.S. food samples.
Biomagnification of anthropogenic and naturally-produced organobrominated compounds in a marine food web fr
Spatial diastereomer patterns of hexabromocyclododecane (HBCD) in a Norwegian fjord.
Accumulation and disposition of hexabromocyclododecane (HBCD) in juvenile rainbow trout (Oncorhynchus mykis
Brominated flame retardants in the Arctic environment--trends and new candidates.
Isobutoxypentabromocyclododecanes (iBPBCDs): a new class of polybrominated compounds.
Spatial distribution of hexabromocyclododecanes (HBCDs), polybrominated diphenyl ethers (PBDEs) and organoch
Pipping success, isomer-specific accumulation, and hepatic mRNA expression in chicken embryos exposed to HBCD
Altered thyroxine metabolism in rainbow trout (Oncorhynchus mykiss) exposed to hexabromocyclododecane (HBC
Hexabromocyclododecane decreases tumor-cell-binding capacity and cell-surface protein expression of human nat
Determination of bromophenols as dioxin precursors in combustion gases of fire retarded extruded polystyrene by
More clues to HBCD isomer mystery.
Levels of hexabromocyclododecane in harbor porpoises and common dolphins from western European seas, with
Structure elucidation of hexabromocyclododecanes--a class of compounds with a complex stereochemistry.
New multiresidue analytical method dedicated to trace level measurement of brominated flame retardants in hum
Subacute effects of the brominated flame retardants hexabromocyclododecane and tetrabromobisphenol A on he
Distribution of hexabromocyclododecane in Detroit River suspended sediments.
Distribution and transportability of hexabromocyclododecane (HBCD) in the Asia-Pacific region using skipjack tuna
Neurotoxicity of the pentabrominated diphenyl ether mixture, DE-71, and hexabromocyclododecane (HBCD) in rat
Characterization of polychlorinated biphenyls and brominated flame retardants in sediments from riverine and coa
Congener-specific analysis of hexabromocyclododecane by high-performance liquid chromatography/electrospray
The environmental occurrence of hexabromocyclododecane in Sweden.
More flame-proofed fish.
Occurrence and bioavailability of polybrominated diphenyl ethers and hexabromocyclododecane in sediment and
A chemical and toxicological profile of Dutch North Sea surface sediments.
Effects of the brominated flame retardants hexabromocyclododecane (HBCDD), and tetrabromobisphenol A (TBBP
Brominated flame retardants in Alburnus alburnus from Cinca River Basin (Spain).
Distribution and fate of HBCD and TBBPA brominated flame retardants in North Sea estuaries and aquatic food we

Biomagnification of polybrominated diphenyl ether and hexabromocyclododecane flame retardants in the polar b
Hexabromocyclododecanes (HBCDs) in the environment and humans: a review.
Regio- and stereoselective isomerization of hexabromocyclododecanes (HBCDs): kinetics and mechanism of beta-
Brominated flame retardants in the environment of Asia-Pacific: an overview of spatial and temporal trends.
Brominated flame retardants in fish and shellfish - levels and contribution of fish consumption to dietary exposure
Two-generation reproductive toxicity study of the flame retardant hexabromocyclododecane in rats.
Temporal trends of polybrominated diphenyl ethers and hexabromocyclododecane in milk from Stockholm mothe
More flame retardants found in house dust.
Hexabromocyclododecanes in indoor dust from Canada, the United Kingdom, and the United States.
Polybrominated diphenyl ethers and HBCD in bird eggs of South Africa.
Maternal transfer of brominated flame retardants in zebrafish (Danio rerio).
Factors influencing enantiomeric fractions of hexabromocyclododecane measured using liquid chromatography/ta
Subacute effects of hexabromocyclododecane (HBCD) on hepatic gene expression profiles in rats.
Spatial distribution and vertical profile of polybrominated diphenyl ethers and hexabromocyclododecanes in sedi
Solid-state conformations and absolute configurations of (+) and (-) alpha-, beta-, and gamma-hexabromocyclodo
Synthesis of the two minor isomers, delta- and epsilon-1,2,5,6,9,10-hexabromocyclododecane, present in comme
Tetrabromobisphenol A (TBBPA) and hexabromocyclododecanes (HBCDs) in tissues of humans, dolphins, and shar
Time trend of hexabromocyclododecane in the breast milk of Japanese women.
Brominated flame retardants in North-East Atlantic marine ecosystems.
Consumption of fish from a contaminated lake strongly affects the concentrations of polybrominated diphenyl eth
Detection of hexabromocyclododecane and its metabolite pentabromocyclododecene in chicken egg and fish fron
Demographic and temporal trends of hexabromocyclododecanes (HBCDD) in an Australian population.
Impairment in the mesohippocampal dopamine circuit following exposure to the brominated flame retardant, HB
Deriving freshwater safety thresholds for hexabromocyclododecane and comparison of toxicity of brominated flan
Effects of the amendment of biochars and carbon nanotubes on the bioavailability of hexabromocyclododecanes (
Complete catalytic debromination of hexabromocyclododecane using a silica-supported palladium catalyst in alkal
Hexabromocyclododecane and tetrabromobisphenol A in sediments and paddy soils from Liaohe River Basin, Chin
Transfer of hexabromocyclododecane flame retardant isomers from captive American kestrel eggs to feathers and
Levels and distribution of tris-(2,3-dibromopropyl) isocyanurate and hexabromocyclododecanes in surface sedime
Characterization of brominated flame retardants in construction and demolition waste components: HBCD and PB
Serum Metabolomic Profiles in Neonatal Mice following Oral Brominated Flame Retardant Exposures to Hexabron
Preliminary screening of polybrominated diphenyl ethers (PBDEs), hexabromocyclododecane (HBCDD) and tetrabr
Dietary exposure assessment of Chinese population to tetrabromobisphenol-A, hexabromocyclododecane and de
Hexabromocyclododecane diastereomers in fish and suspended particulate matter from selected European waters
Emerging and legacy flame retardants in UK human milk and food suggest slow response to restrictions on use of
Accumulation of ?-hexabromocyclododecane (?-HBCDD) in tissues of fast- and slow-growing broilers (Gallus dome
Biotransformation of hexabromocyclododecanes with hexachlorocyclohexane-transforming Sphingobium chinhate
Does the source migration pathway of HBCDs to household dust influence their bio-accessibility?
Determination of hexabromocyclododecanes in sediments from the Haihe River in China by an optimized HPLC-M
A national survey of tetrabromobisphenol-A, hexabromocyclododecane and decabrominated diphenyl ether in hu
Alteration of Diastereoisomeric and Enantiomeric Profiles of Hexabromocyclododecanes (HBCDs) in Adult Chicken
Transcriptomic and metabolomic approaches to investigate the molecular responses of human cell lines exposed t
Indoor pollutant hexabromocyclododecane enhances house dust mite-induced activation of human monocyte-de
Discrimination of hexabromocyclododecane from new polymeric brominated flame retardant in polystyrene foam
Levels and distributions of polybrominated diphenyl ethers, hexabromocyclododecane, and tetrabromobisphenol
Environmental risks of HBCDD from construction and demolition waste: a contemporary and future issue.
Brominated flame retardants - Exposure and risk assessment for the general population.

Leaching behaviour of hexabromocyclododecane from treated curtains.
Hexabromocyclododecane flame retardant in Antarctica: Research stations as sources.
Hexabromocyclododecane and tetrabromobisphenol A alter secretion of interferon gamma (IFN-?) from human im
Evaluation of 3D-human skin equivalents for assessment of human dermal absorption of some brominated flame r
Hexabromocyclododecane affects benthic-pelagic coupling in an experimental ecosystem.
Concentration of novel brominated flame retardants and HBCD in leachates and sediments from selected municipa
Short-term effects of a perinatal exposure to the HBCDD ?-isomer in rats: Assessment of early motor and sensory d
Tetrabromobisphenol A and hexabromocyclododecane alter secretion of IL-1? from human immune cells.
Brominated flame retardant emissions from the open burning of five plastic wastes and implications for environme
Long-term emissions of hexabromocyclododecane as a chemical of concern in products in China.
New Insights into the Cytotoxic Mechanism of Hexabromocyclododecane from a Metabolomic Approach.
Emerging halogenated flame retardants and hexabromocyclododecanes in food samples from an e-waste processi
Potential of gas chromatography-atmospheric pressure chemical ionization-tandem mass spectrometry for screeni
Hexabromocyclododecanes (HBCDDs) in surface soils from coastal cities in North China: Correlation between diast
Simultaneous liquid chromatography-tandem mass spectrometry analysis of brominated flame retardants (tetrabr
Hexabromocyclododecane (HBCD) induced changes in the liver proteome of eu- and hypothyroid female rats.
Direct contact between dust and HBCD-treated fabrics is an important pathway of source-to-dust transfer.
Brominated flame retardants (BFRs): A review on environmental contamination in China.
Brominated flame retardants, hexabromocyclododecane and tetrabromobisphenol A, affect proinflammatory prot
Hens can ingest extruded polystyrene in rearing buildings and lay eggs contaminated with hexabromocyclododeca
The effects of prosperity indices and land use indicators of an urban conurbation on the occurrence of hexabromo
Treatability of hexabromocyclododecane using Pd/Fe nanoparticles in the soil-plant system: Effects of humic acids.
Hexabromocyclododecane (HBCD): A case study applying tiered testing for human health risk assessment.
Distribution and diastereoisomeric profiles of hexabromocyclododecanes in air, water, soil, and sediment samples
Diastereoisomer-specific neurotoxicity of hexabromocyclododecane in human SH-SY5Y neuroblastoma cells.
Distribution, diastereomer-specific accumulation and associated health risks of hexabromocyclododecanes (HBCDs
Inhomogeneity of sediment samples in analysis of hexabromocyclododecane.
Bioaccumulation and translocation of tetrabromobisphenol A and hexabromocyclododecanes in mangrove plants f
Temporal-spatial distribution and diastereoisomer pattern of hexabromocyclododecane in the vicinity of a chemica
Rat strain response differences upon exposure to technical or alpha hexabromocyclododecane.
Dietary exposure and risk assessment of exposure to hexabromocyclododecanes in a Taiwan population.
Hepatic transcriptional dose-response analysis of male and female Fischer rats exposed to hexabromocyclododeca
Tetrabromobisphenol A and hexabromocyclododecane isomers in breast milk from the general population in Beijin
[Dermatological evaluation of a flame retardant, hexabromocyclododecane (HBCD) on guinea pig by using the prin
Analysis of polybrominated diphenyl ethers, hexabromocyclododecanes, and legacy and emerging phosphorus flar
Coastal biomonitoring survey on persistent organic pollutants using oysters (Saccostrea mordax) from Okinawa, Ja
Cytotoxicity of hexabromocyclododecane, 1,2-dibromo-4-(1,2-dibromoethyl) cyclohexane and 1,2,5,6-tetrabromoc
Hexabromocyclododecane in riverine and estuarine sediments from Osaka, Japan: spatial distribution and concent
Hexabromocyclododecane: concentrations and isomer profiles from sources to environmental sinks.
Fate of Hexabromocyclododecane (HBCD), A Common Flame Retardant, In Polystyrene-Degrading Mealworms: Ele
The enrichment and purification of hexabromocyclododecanes and its effects on thyroid in zebrafish.
Evaluating hexabromocyclododecane (HBCD) toxicokinetics in humans and rodents by physiologically based pharm
The HBCDs biodegradation using a Pseudomonas strain and its application in soil phytoremediation.
Serum measures of hexabromocyclododecane (HBCDD) and polybrominated diphenyl ethers (PBDEs) in reproducti
Biodegradation of hexabromocyclododecane by Rhodopseudomonas palustris YSC3 strain: A free-living nitrogen-fi
Flame retardants, hexabromocyclododecane (HCBD) and tetrabromobisphenol a (TBBPA), alter secretion of tumor
Atmospheric concentrations of hexabromocyclododecane (HBCDD) diastereomers in the Great Lakes region.

Hexabromocyclododecane in polystyrene packaging: A downside of recycling?
Legacy and emerging brominated flame retardants in China: A review on food and human milk contamination, hur
Hexabromocyclododecanes (HBCDs) in fish: Evidence of recent HBCD input into the coastal environment.
Occurrence of polybrominated diphenylethers, hexabromocyclododecanes, bromophenols and tetrabromobisphe
Dietary exposure to brominated flame retardants and risk of type 2 diabetes in the French E3N cohort.
Development and validation of a quantitative UHPLC-MS/MS method for selected brominated flame retardants in
Liver volatolomics to reveal poultry exposure to ?-hexabromocyclododecane (HBCD).
Hexabromocyclododecane and tetrabromobisphenol A in tree bark from different functional areas of Shanghai, Ch
Hexabromocyclododecane-induced Genotoxicity in Cultured Human Breast Cells through DNA Damage.
Bioconcentration and effects of hexabromocyclododecane exposure in crucian carp (Carassius auratus).
Stereoisomer-Specific Trophodynamics of the Chiral Brominated Flame Retardants HBCD and TBECH in a Marine Fo
Concentrations, Distributions, and Risk Assessment of HBCD in Sediment in the Weihe River Basin in Northwest Ch
Legacy and alternative brominated flame retardants in outdoor dust and pine needles in mainland China: Spatial tr
Elucidating the Variability in the Hexabromocyclododecane Diastereomer Profile in the Global Environment.
Factors influencing risk assessments of brominated flame-retardants; evidence based on seafood from the North E
Contamination trends and factors affecting the transfer of hexabromocyclododecane diastereomers, tetrabromobi
Biomagnification of Hexabromocyclododecane (HBCD) in a coastal ecosystem near a large producer in China: Hum
Hexabromocyclododecanes in breast milk from residents in Shenzhen, China: Implications for infant exposure.
Application of triolein-embedded cellulose acetate membrane (TECAM) passive sampler to study phase distributio
Brominated Flame Retardants in Sediments of Four Coastal Lagoons of Yucatan, Mexico.
Bioaccumulation and Distribution of Hexabromocyclododecane Isomers in Duck Tissues.
Occurrence of tetrabromobisphenol a (TBBPA) and hexabromocyclododecane (HBCD) in soil and road dust in Chon
Developmental toxicity evaluation of three hexabromocyclododecane diastereoisomers on zebrafish embryos.
Simultaneous extraction and determination of HBCD isomers and TBBPA by ASE and LC-MSMS in fish.
In situ accumulation of HBCD, PBDEs, and several alternative flame-retardants in the bivalve (Corbicula fluminea) a
2,5,6,9,10-Pentabromocyclododecanols (PBCDOHs): a new class of HBCD transformation products.
[Determination of three brominated flame retardants in human serum using solid-phase extraction coupled with u
Levels, isomer profiles and chiral signatures of particle-bound hexabromocyclododecanes in ambient air around Sh
Monitoring of hexabromocyclododecane diastereomers in fish from European freshwaters and estuaries.
Tris(2,3-dibromopropyl) isocyanurate, hexabromocyclododecanes, and polybrominated diphenyl ethers in mollusk
Tissue-specific accumulation of polybrominated diphenyl ethers (PBDEs) including Deca-BDE and hexabromocycloc
Vapor pressure of three brominated flame retardants determined by using the Knudsen effusion method.
Comparisons of polybrominated diphenyl ether and hexabromocyclododecane concentrations in dust collected wi
Tri-decabrominated diphenyl ethers and hexabromocyclododecane in indoor air and dust from Stockholm microer
A review of the analysis of novel brominated flame retardants.
Diasteroisomer and enantiomer-specific profiles of hexabromocyclododecane and tetrabromobisphenol A in an ac
Transient aberration of neuronal development in the hippocampal dentate gyrus after developmental exposure to
Diastereoisomer- and enantiomer-specific accumulation, depuration, and bioisomerization of hexabromocyclodod
Levels of tetrabromobisphenol A, hexabromocyclododecanes and polybrominated diphenyl ethers in human milk f
Three decades (1983-2010) of contaminant trends in East Greenland polar bears (Ursus maritimus). Part 2: bromin
Hexabromocyclododecanes in surface sediments and a sediment core from Rivers and Harbor in the northern Chir
Co-release of hexabromocyclododecane (HBCD) and Nano- and microparticles from thermal cutting of polystyrene
Hexabromocyclododecane (HBCD) stereoisomers in U.S. food from Dallas, Texas.
Levels and distribution of hexabromocyclododecane (HBCD) in environmental samples near manufacturing facilitie
Spatial distribution and inter-year variation of hexabromocyclododecane (HBCD) and tris-(2,3-dibromopropyl) isoc
Policy relevant results from an expert elicitation on the human health risks of decabromodiphenyl ether (decaBDE
Brominated flame retardants in the Australian population: 1993-2009.

Detection of Dechlorane Plus and brominated flame retardants in marketed fish in Japan.
Predictors of tetrabromobisphenol-A (TBBP-A) and hexabromocyclododecanes (HBCD) in milk from Boston mother
[Effects of perinatal exposure to the brominated flame-retardant hexabromocyclododecane (HBCD) on the develo
Crystal structure of ?-isobutoxypentabromo-cyclododecanes, kinetics and selectivity of their isomerization during
Temporal trend (1988-2008) of hexabromocyclododecane enantiomers in herring gull eggs from the German coast
Brominated flame retardants in dust from UK cars--within-vehicle spatial variability, evidence for degradation and
Rapid identification of polystyrene foam wastes containing hexabromocyclododecane or its alternative polymeric
Flame retardants and legacy contaminants in polar bears from Alaska, Canada, East Greenland and Svalbard, 2005-
Matrix-specific distribution and diastereomeric profiles of hexabromocyclododecane (HBCD) in a multimedia envir
Diastereoisomer- and enantiomer-specific determination of hexabromocyclododecane in fish oil for food and feed
Determinations of hexabromocyclododecane (HBCD) isomers in channel catfish, crayfish, hen eggs and fish feeds f
Occurrence, sources, and inventory of hexabromocyclododecanes (HBCDs) in soils from Chongming Island, the Yar
Hexabromocyclododecanes (HBCDs) in marine fishes along the Chinese coastline.
Quantitative determination of the diastereoisomers of hexabromocyclododecane in human plasma using liquid ch
Fate of tetrabromobisphenol A and hexabromocyclododecane brominated flame retardants in soil and uptake by
Levels of brominated flame retardants and methoxylated polybrominated diphenyl ethers in eggs of white-tailed s
1,2,5,6,9,10-?Hexabromocyclododecane (HBCD) impairs thyroid hormone-induced dendrite arborization of Purkin
Reproductive changes in American kestrels (Falco sparverius) in relation to exposure to technical hexabromocyclo
Do temporal and geographical patterns of HBCD and PBDE flame retardants in U.S. fish reflect evolving industrial u
Kinetic study of ?-hexabromocyclododecane orally given to laying hens (Gallus domesticus). "Transfer of HBCD in la
Toxicokinetics and carry-over model of ?-hexabromocyclododecane (HBCD) from feed to consumption-sized Atlant
Preparation and X-ray structural characterization of further stereoisomers of 1,2,5,6,9,10-hexabromocyclododecan
Differences in tissue distribution of HBCD alpha and gamma between adult and developing mice.
Brominated flame retardants in Canadian chicken egg yolks.
Temporal trends of polybrominated diphenyl ethers and hexabromocyclododecane in Swedish Peregrine Falcon (F
Dietary intake of hexabromocyclododecane diastereoisomers (?-, ?-, and ?-HBCD) in the Belgian adult population.
Hexabromocyclododecane enantiomers: microsomal degradation and patterns of hydroxylated metabolites.
Human exposure to PCBs, PBDEs and HBCDs in Ghana: Temporal variation, sources of exposure and estimation of
Assessing oestrogenic effects of brominated flame retardants hexabromocyclododecane and tetrabromobispheno
Toxicokinetics of the flame retardant hexabromocyclododecane alpha: effect of dose, timing, route, repeated expo
Biomonitoring-based risk assessment for hexabromocyclododecane (HBCD).
Determination of hexabromocyclododecane by flowing atmospheric pressure afterglow mass spectrometry.
Occurrence of additive brominated flame retardants in aquatic organisms from Tai Lake and Yangtze River in Easte
Prenatal exposure to the brominated flame retardant hexabromocyclododecane (HBCD) impairs measures of susta
The transformation of hexabromocyclododecane using zerovalent iron nanoparticle aggregates.
The brominated flame retardants, PBDEs and HBCD, in Canadian human milk samples collected from 1992 to 2005
Brominated flame retardants, tetrabromobisphenol A and hexabromocyclododecane, activate mitogen-activated
Evaluation of spatial distribution and accumulation of novel brominated flame retardants, HBCD and PBDEs in an I
HBCDD-induced sustained reduction in mitochondrial membrane potential, ATP and steroidogenesis in peripubert
Brominated flame retardant exposure of aircraft personnel.
Cross-omics gene and protein expression profiling in juvenile female mice highlights disruption of calcium and zinc
Exposure to an environmentally relevant mixture of brominated flame retardants affects fetal development in Spr
Hexabromocyclododecane in polystyrene based consumer products: an evidence of unregulated use.
Levels of flame retardants HBCD, TBBPA and TBC in surface soils from an industrialized region of East China.
Destruction behavior of hexabromocyclododecanes during incineration of solid waste containing expanded and ex
Uptake, distribution, depletion, and in ovo transfer of isomers of hexabromocyclododecane flame retardant in die
Concentrations of Polybrominated Diphenyl Ethers, Hexabromocyclododecanes and Tetrabromobisphenol-A in Bre

Study of novel pure culture HBCD-1, effectively degrading Hexabromocyclododecane, isolated from an anaerobic r
PBDE, HBCD, and novel brominated flame retardant contamination in sediments from Lake Maggiore (Northern Ita
Selective damage to dopaminergic transporters following exposure to the brominated flame retardant, HBCDD.
Hexabromocyclododecane Flame Retardant Isomers in Sediments from Detroit River and Lake Erie of the Laurentia
Emerging flame retardants, PBDEs, and HBCDDs in indoor and outdoor media in Stockholm, Sweden.
Destruction of the flame retardant hexabromocyclododecane in a full-scale municipal solid waste incinerator.
Review of hexabromocyclododecane (HBCD) with a focus on legislation and recent publications concerning toxicok
Enrichment of hexabromocyclododecanes in coastal sediments near aquaculture areas and a wastewater treatmen
Effects of benthos, temperature, and dose on the fate of hexabromocyclododecane in experimental coastal ecosys
Brominated flame retardant concentrations in sera from the Canadian Health Measures Survey (CHMS) from 2007
The determination of perfluoroalkyl substances, brominated flame retardants and their metabolites in human brea
The sarcoplasmic-endoplasmic reticulum Ca(2+)-ATPase (SERCA) is the likely molecular target for the acute toxicity
Hexabromocyclododecane concentrations in Canadian human fetal liver and placental tissues.
Tetrabromobisphenol A and hexabromocyclododecane flame retardants in infant-mother paired serum samples, a
Emission behavior of hexabromocyclododecanes and polybrominated diphenyl ethers from flame-retardant-treate
Hexabromocyclododecanes in limnic and marine organisms and terrestrial plants from Tianjin, China: diastereome
Polybrominated diphenyl ethers (PBDEs) and hexabromocyclododecane (HBCD) in seven different marine bird spe
Effects of low-level hexabromocyclododecane (HBCD) exposure on cardiac development in zebrafish embryos.
A two-dimensional HPLC separation for the enantioselective determination of hexabromocyclododecane (HBCD) is
Photolysis of brominated flame retardants in textiles exposed to natural sunlight.
The fate of ?-hexabromocyclododecane in female C57BL/6 mice.
Levels and trends of PBDEs and HBCDs in the global environment: status at the end of 2012.
Temporal trends in classical and alternative flame retardants in bird eggs from Doñana Natural Space and surrounc
Occurrence of brominated flame retardants and perfluoroalkyl substances in fish from the Czech aquatic ecosyster
Hexabromocyclododecanes in crucian carp and sediment from the major rivers in Korea.
New perspectives on diastereoselective determination of hexabromocyclododecane traces in fish by ultra high per
Oligomeric proanthocyanidins alleviate hexabromocyclododecane-induced cytotoxicity in HepG2 cells through reg
Impaired lipid and glucose homeostasis in hexabromocyclododecane-exposed mice fed a high-fat diet.
[Status of the upper respiratory tract and skin of workers manufacturing artificial mineral wool].
Development of metal hydroxide nanoparticles from eggshell waste and seawater and their application as flame re
Steroid secretion following exposure of ovarian follicular cells to single congeners and defined mixture of polybron
A multilevel approach to predict toxicity in copepod populations: assessment of growth, genetics, and population s
Effects of BDE-85 on the oxidative status and nerve conduction in rodents.
Physicochemical properties of selected polybrominated diphenyl ethers and extension of the UNIFAC model to bro
Semivolatile organic compounds in homes: strategies for efficient and systematic exposure measurement based or
Penta- and octa-bromodiphenyl ethers promote proinflammatory protein expression in human bronchial epithelia
Technical pentabromodiphenyl ether and hexabromocyclododecane as activators of the pregnane-X-receptor (PXR
Ultrastructural changes observed in rat ovaries following in utero and lactational exposure to low doses of a polybr
A commercial mixture of the brominated flame retardant pentabrominated diphenyl ether (DE-71) induces respira
Probing new approaches using atmospheric pressure photo ionization for the analysis of brominated flame retarda
Differential expression of CYP1A, 2B, and 3A genes in the F344 rat following exposure to a polybrominated diphen
Brief postnatal PBDE exposure alters learning and the cholinergic modulation of attention in rats.
Salmon flame retardant research raises new questions.
Developmental exposure to low dose PBDE 99: effects on male fertility and neurobehavior in rat offspring.
Polybrominated diphenyl ethers (PBDEs) in U.S. computers and domestic carpet vacuuming: possible sources of hu
Brominated flame retardants in Alburnus alburnus from Cinca River Basin (Spain).
Effects of pentabrominated diphenyl ether (PBDE-99) on vitamin status in domestic duck (Anas platyrhynchos) hat

Exposure to flame retardants: nursing concern.
Neurotoxicity of the pentabrominated diphenyl ether mixture, DE-71, and hexabromocyclododecane (HBCD) in rat
Brominated flame retardants in tree bark from North America.
In vivo and in vitro Ah-receptor activation by commercial and fractionated pentabromodiphenylether using zebrafi
Effects of selected polybrominated diphenyl ether flame retardants on lake trout (Salvelinus namaycush).
Exposure to polybrominated diphenyl ethers (PBDEs): changes in thyroid, vitamin A, glutathione homeostasis, and
Polybrominated diphenyl ether (PBDE)-induced alterations in vitamin A and thyroid hormone concentrations in the
Dietary accumulation efficiencies and biotransformation of polybrominated diphenyl ethers in farmed Atlantic saln
New multiresidue analytical method dedicated to trace level measurement of brominated flame retardants in hum
Proteomic evaluation of neonatal exposure to 2,2 ,4,4 ,5-pentabromodiphenyl ether.
Tissue disposition, excretion and metabolism of 2,2',4,4',5-pentabromodiphenyl ether (BDE-99) in the male Spragu
Differential effects of polybrominated diphenyl ethers and polychlorinated biphenyls on [3H]arachidonic acid relea
Levels of polybrominated diphenyl ether (PBDE) flame retardants in animals representing different trophic levels o
Effects of perinatal exposure to a polybrominated diphenyl ether (PBDE 99) on mouse neurobehavioural developm
The expression of CYP1A, vitellogenin and zona radiata proteins in Atlantic salmon (Salmo salar) after oral dosing v
Comparison of analytical strategies for the chromatographic and mass spectrometric measurement of brominated
Flame retardant exposure: polybrominated diphenyl ethers in blood from Swedish workers.
The toxicology of the three commercial polybrominated diphenyl oxide (ether) flame retardants.
PBDE information overlooked?
A brominated flame retardant, 2,2',4,4',5-pentabromodiphenyl ether: uptake, retention, and induction of neurobe
Neonatal exposure to the brominated flame retardant 2,2',4,4',5-pentabromodiphenyl ether causes altered suscep
[Flame retardants--use and hazards for human].
Occurrence and bioavailability of polybrominated diphenyl ethers and hexabromocyclododecane in sediment and
Preventing fires, igniting questions.
Some polybrominated diphenyl ether (PBDE) flame retardants with wide environmental distribution inhibit TCDD-i
Screening of halogenated aromatic compounds in some raw material lots for an aluminium recycling plant.
Temporal trends and spatial distributions of brominated flame retardants in archived fishes from the Great Lakes.
Impact of fermented brown rice with Aspergillus oryzae (FEBRA) intake and concentrations of polybrominated dipl
Clarifications on PBDE flame retardants.
U.S. PBDE milestones.
Deca PBDE flame retardant gets around.
Occurrence of polychlorinated biphenyls and polybrominated diphenyl ethers in green mussels (Perna viridis) from
Developmental neurotoxicity of polybrominated diphenyl ethers mixture de71 in Sprague-Dawley rats.
Propelling plastics into the circular economy - weeding out the toxics first.
Multi-generational effects of polybrominated diphenylethers exposure: embryonic exposure of male American kes
Comparative Study of Genotoxicity Induced by Six Different PBDEs.
The environmental fate of polybrominated diphenyl ethers (PBDEs) in western Taiwan and coastal waters: evaluati
Effects of the Commercial Flame Retardant Mixture DE-71 on Cytokine Production by Human Immune Cells.
Temporal trends of PBDEs and emerging flame retardants in belugas from the St. Lawrence Estuary (Canada) and c
DNA Methylation Changes in Tbx3 in a Mouse Model Exposed to Polybrominated Diphenyl Ethers.
Effect of smoking and caffeine consumption on polybrominated diphenyl ethers (PBDE) and polybrominated biphe
Polybrominated diphenyl ethers alter hepatic phosphoenolpyruvate carboxykinase enzyme kinetics in male Wistar
Novel and high volume use flame retardants in US couches reflective of the 2005 PentaBDE phase out.
After the PBDE phase-out: a broad suite of flame retardants in repeat house dust samples from California.
Exposure to polybrominated diphenyl ethers and female reproductive function: A study in the production area of S
Differences in neonatal neurotoxicity of brominated flame retardants, PBDE 99 and TBBPA, in mice.
Geographical distribution of non-PBDE-brominated flame retardants in mussels from Asian coastal waters.

Porphyrogenic effect of pentabromodiphenyl ether after repeated administration to rats.
Acute postnatal exposure to the pentaBDE commercial mixture DE-71 at 5 or 15 mg/kg/day does not produce lear
More signs of neurotoxicity of surfactants and flame retardants - Neonatal PFOS and PBDE 99 cause transcriptiona
Associations between PBDEs in office air, dust, and surface wipes.
Alterations to the circuitry of the frontal cortex following exposure to the polybrominated diphenyl ether mixture,
Tissue-specific accumulation of polybrominated diphenyl ethers (PBDEs) including Deca-BDE and hexabromocycloc
Prenatal transfer of polybrominated diphenyl ethers (PBDEs) results in developmental neurotoxicity in zebrafish la
Associations between serum levels of polybrominated diphenyl ether (PBDE) flame retardants and environmental
Effects of chronic exposure to an environmentally relevant mixture of brominated flame retardants on the reprodu
Immune function in female B(6)C(3)F(1) mice is modulated by DE-71, a commercial polybrominated diphenyl ethe
Detection of polybrominated biphenyl ethers (PBDEs) in pediatric hair as a tool for determining in utero exposure.
Immunologic and endocrine effects of the flame-retardant pentabromodiphenyl ether (DE-71) in C57BL/6J mice.
Lack of effects of some individual polybrominated diphenyl ether (PBDE) and polychlorinated biphenyl (PCB) conge
The occurrence of polybrominated diphenyl ether (PBDE) contamination in soil, water/sediment, and air.
Brominated flame retardants in animal derived foods in the Netherlands between 2009 and 2014.
Polybrominated diphenyl ethers (PBDEs) in chicken eggs and cow milk around municipal dumpsites in Abuja, Niger
Decreasing but still high levels of halogenated flame retardants in wetland birds in central Spain.
Fetal exposure to polybrominated diphenyl ethers and the risk of hypospadias: focus on the congeners involved.
Epigenetic Effects of Polybrominated Diphenyl Ethers on Human Health.
Concentrations and loadings of organophosphate and replacement brominated flame retardants in house dust fro
Single-cell RNA-sequencing analysis of estrogen- and endocrine-disrupting chemical-induced reorganization of mou
[Reproductive toxicity of PBDE in males: Advances in studies].
A noninvasive environmental monitoring tool for brominated flame-retardants (BFRs) assisted by conservation det
Racial/ethnic and geographic differences in polybrominated diphenyl ether (PBDE) levels across maternal, placenta
Polybrominated biphenyl and diphenylether flame retardants: analysis, toxicity, and environmental occurrence.
The brominated flame retardant PBDE 99 promotes adipogenesis via regulating mitotic clonal expansion and PPAR
Distribution of polybrominated diphenyl ethers in the atmosphere of the Pearl River Delta region, South China.
Temporal trends and developmental patterns of plasma polybrominated diphenyl ether concentrations over a 15-y
Concentrations of legacy and novel brominated flame retardants in indoor dust in Melbourne, Australia: An assess
Polybrominated diphenyl ethers (PBDEs) and hydroxylated PBDE metabolites (OH-PBDEs): A six-year temporal tren
Multi-analyte method development for analysis of brominated flame retardants (BFRs) and PBDE metabolites in hu
The flame retardant DE-71 (a mixture of polybrominated diphenyl ethers) inhibits human differentiated thyroid ce
A preliminary study on prenatal polybrominated diphenyl ether serum concentrations and intrinsic functional netw
Species and habitat-dependent accumulation and biomagnification of brominated flame retardants and PBDE met
Dietary exposure to a binary mixture of polybrominated diphenyl ethers alters innate immunity and disease susce
Pine needles as biomonitors of polybrominated diphenyl ethers and emerging flame retardants in the atmosphere
Prenatal polybrominated diphenyl ethers exposure and anogenital distance in boys from a Shanghai birth cohort.
Occurrence, levels and profiles of brominated flame retardants in daily-use consumer products on the Chinese ma
Association of In Utero Exposure to Polybrominated Diphenyl Ethers With the Risk of Hypospadias.
DE-71-induced apoptosis involving intracellular calcium and the Bax-mitochondria-caspase protease pathway in hu
Flame retardants (PBDEs) in marine turtles, dugongs and seafood from Queensland, Australia.
A 28-day oral dose toxicity study enhanced to detect endocrine effects of a purified technical pentabromodipheny
Polybrominated diphenyl ethers as endocrine disruptors of adipocyte metabolism.
Spatial trends of polybrominated diphenyl ethers in avian species: utilization of stored samples in the Environment
Brominated flame retardants in the environment of Asia-Pacific: an overview of spatial and temporal trends.
Bioaccumulation behaviour of polybrominated diphenyl ethers (PBDEs) in a Canadian Arctic marine food web.
Neurotoxicity of a polybrominated diphenyl ether mixture (DE-71) in mouse neurons and astrocytes is modulated

Linking PBDEs in house dust to consumer products using X-ray fluorescence.
Trophic level determines levels of brominated flame-retardants in coastal herring gulls.
Exposure to brominated flame retardant PBDE-99 affects cytoskeletal protein expression in the neonatal mouse ce
Measurement of polybrominated diphenyl ethers on hand wipes: estimating exposure from hand-to-mouth conta
Detection of PBDE effects on mRNA expression in chicken (Gallus domesticus) neuronal cells using real-time RT-PC
Electrolytic debromination of PBDEs in DE-83 technical decabromodiphenyl ether.
Plasma PBDE and thyroxine levels in rats exposed to Bromkal or BDE-47.
Accumulation, tissue-specific distribution and debromination of decabromodiphenyl ether (BDE 209) in European
Polybrominated diphenyl ether in sewage sludge in Germany.
Brominated flame retardants and halogenated phenolic compounds in North American west coast bald eaglet (Hal
Congener distribution of polybrominated diphenyl ethers in feral carp (Cyprinus carpio) from the Llobregat River, S
Polybrominated diphenyl ethers, a group of brominated flame retardants, can interact with polychlorinated bipher
Decabromodiphenyl ether (deca-BDE) commercial mixture components, and other PBDEs, in airborne particles at
Flame retardants in placenta and breast milk and cryptorchidism in newborn boys.
Variation, levels and profiles of organochlorines and brominated flame retardants in great tit (Parus major) eggs fr
Coexposure of neonatal mice to a flame retardant PBDE 99 (2,2',4,4',5-pentabromodiphenyl ether) and methyl me
Geographical distribution and accumulation features of PBDEs in human breast milk from Indonesia.
The risk of PBDEs in dust.
The flame retardants, polybrominated diphenyl ethers, are pregnane X receptor activators.
PBDEs in circuit boards.
Individual characteristics associated with PBDE levels in U.S. human milk samples.
Toxicity of penta- and decabromodiphenyl ethers after repeated administration to rats: a comparative study.
Reductive debromination of polybrominated diphenyl ethers by anaerobic bacteria from soils and sediments.
In vitro neurotoxicity of PBDE-99: immediate and concentration-dependent effects on protein expression in cerebr
Desorption and bioavailability of spiked pentabromo diphenyl ether and tetrachlorodibenzo(p)dioxin in contamina
Thyroid insult: flame retardants linked to alterations in pregnant women's TSH levels.
Polychlorinated biphenyls and polybrominated diphenyl ethers alter striatal dopamine neurochemistry in synaptos
PBDE exposure from food in Ireland: optimising data exploitation in probabilistic exposure modelling.
Brominated diphenyl ether (BDE) levels in liver, adipose, and milk from adult and juvenile rats exposed by gavage t
Polybrominated diphenyl ether (PBDE) levels in peregrine falcon (Falco peregrinus) eggs from California correlate v
Flame retardants have different effects at high and low doses.
Toxicity assessment of air-delivered particle-bound polybrominated diphenyl ethers.
The effect of short-term intoxication of rats with pentabromodiphenyl ether (in mixture mimic commercial produc
[Preliminary study of PBDE levels in house dust and human exposure to PBDEs via dust ingestion].
Reproductive and developmental toxicity of a pentabrominated diphenyl ether mixture, DE-71, to ranch mink (Mu
Polybrominated diphenyl ether (PBDE) concentrations in house dust are related to hormone levels in men.
The effects of marginal maternal vitamin A status on penta-brominated diphenyl ether mixture-induced alteration
Chronic postnatal DE-71 exposure: effects on learning, attention and thyroxine levels.
Toxicity of polybrominated diphenyl ethers (DE-71) in chicken (Gallus gallus), mallard (Anas platyrhynchos), and An
Effects of perinatal PBDE exposure on hepatic phase I, phase II, phase III, and deiodinase 1 gene expression involve
Spending time in vehicles can increase PBDE exposure.
Exposure to DE-71 alters thyroid hormone levels and gene transcription in the hypothalamic-pituitary-thyroid axis
Levels and congener specific profiles of PBDEs in human breast milk from China: implication on exposure sources a
Steroid secretion following exposure of ovarian follicular cells to single congeners and defined mixture of polybron
A multilevel approach to predict toxicity in copepod populations: assessment of growth, genetics, and population
Effects of BDE-85 on the oxidative status and nerve conduction in rodents.
Penta- and octa-bromodiphenyl ethers promote proinflammatory protein expression in human bronchial epithelia

Physicochemical properties of selected polybrominated diphenyl ethers and extension of the UNIFAC model to brc
Semivolatile organic compounds in homes: strategies for efficient and systematic exposure measurement based or
Technical pentabromodiphenyl ether and hexabromocyclododecane as activators of the pregnane-X-receptor (PXR
A commercial mixture of the brominated flame retardant pentabrominated diphenyl ether (DE-71) induces respira
Probing new approaches using atmospheric pressure photo ionization for the analysis of brominated flame retarda
Brominated flame retardants in Alburnus alburnus from Cinca River Basin (Spain).
Effects of pentabrominated diphenyl ether (PBDE-99) on vitamin status in domestic duck (Anas platyrhynchos) hat
Differential expression of CYP1A, 2B, and 3A genes in the F344 rat following exposure to a polybrominated diphen
Brief postnatal PBDE exposure alters learning and the cholinergic modulation of attention in rats.
Effects of selected polybrominated diphenyl ether flame retardants on lake trout (Salvelinus namaycush).
Exposure to flame retardants: nursing concern.
Developmental exposure to low dose PBDE 99: effects on male fertility and neurobehavior in rat offspring.
Ultrastructural changes observed in rat ovaries following in utero and lactational exposure to low doses of a polyb
Polybrominated diphenyl ethers (PBDEs) in U.S. computers and domestic carpet vacuuming: possible sources of hu
In vivo and in vitro Ah-receptor activation by commercial and fractionated pentabromodiphenylether using zebrafi
Neurotoxicity of the pentabrominated diphenyl ether mixture, DE-71, and hexabromocyclododecane (HBCD) in rat
Brominated flame retardants in tree bark from North America.
Comparison of analytical strategies for the chromatographic and mass spectrometric measurement of brominated
New multiresidue analytical method dedicated to trace level measurement of brominated flame retardants in hum
Exposure to polybrominated diphenyl ethers (PBDEs): changes in thyroid, vitamin A, glutathione homeostasis, and
Dietary accumulation efficiencies and biotransformation of polybrominated diphenyl ethers in farmed Atlantic salr
Proteomic evaluation of neonatal exposure to 2,2 ,4,4 ,5-pentabromodiphenyl ether.
Polybrominated diphenyl ether (PBDE)-induced alterations in vitamin A and thyroid hormone concentrations in the
Effects of perinatal exposure to a polybrominated diphenyl ether (PBDE 99) on mouse neurobehavioural developm
Tissue disposition, excretion and metabolism of 2,2',4,4',5-pentabromodiphenyl ether (BDE-99) in the male Spragu
Levels of polybrominated diphenyl ether (PBDE) flame retardants in animals representing different trophic levels o
Differential effects of polybrominated diphenyl ethers and polychlorinated biphenyls on [3H]arachidonic acid relea
The expression of CYP1A, vitellogenin and zona radiata proteins in Atlantic salmon (Salmo salar) after oral dosing w
[Flame retardants--use and hazards for human].
A brominated flame retardant, 2,2',4,4',5-pentabromodiphenyl ether: uptake, retention, and induction of neurobe
Flame retardant exposure: polybrominated diphenyl ethers in blood from Swedish workers.
PBDE information overlooked?
Neonatal exposure to the brominated flame retardant 2,2',4,4',5-pentabromodiphenyl ether causes altered suscep
The toxicology of the three commercial polybrominated diphenyl oxide (ether) flame retardants.
Some polybrominated diphenyl ether (PBDE) flame retardants with wide environmental distribution inhibit TCDD-i
Occurrence and bioavailability of polybrominated diphenyl ethers and hexabromocyclododecane in sediment and
Preventing fires, igniting questions.
Temporal trends and spatial distributions of brominated flame retardants in archived fishes from the Great Lakes.
Impact of fermented brown rice with Aspergillus oryzae (FEBRA) intake and concentrations of polybrominated dipl
Salmon flame retardant research raises new questions.
Clarifications on PBDE flame retardants.
Occurrence of polychlorinated biphenyls and polybrominated diphenyl ethers in green mussels (Perna viridis) from
U.S. PBDE milestones.
Deca PBDE flame retardant gets around.
Screening of halogenated aromatic compounds in some raw material lots for an aluminium recycling plant.
Developmental neurotoxicity of polybrominated diphenyl ethers mixture de71 in Sprague-Dawley rats.
Polybrominated diphenyl ethers, a group of brominated flame retardants, can interact with polychlorinated bipher

Propelling plastics into the circular economy - weeding out the toxics first.
Effects of the Commercial Flame Retardant Mixture DE-71 on Cytokine Production by Human Immune Cells.
Comparative Study of Genotoxicity Induced by Six Different PBDEs.
The environmental fate of polybrominated diphenyl ethers (PBDEs) in western Taiwan and coastal waters: evaluati
After the PBDE phase-out: a broad suite of flame retardants in repeat house dust samples from California.
Associations between PBDEs in office air, dust, and surface wipes.
Effect of smoking and caffeine consumption on polybrominated diphenyl ethers (PBDE) and polybrominated biphe
Exposure to polybrominated diphenyl ethers and female reproductive function: A study in the production area of S
DNA Methylation Changes in Tbx3 in a Mouse Model Exposed to Polybrominated Diphenyl Ethers.
Novel and high volume use flame retardants in US couches reflective of the 2005 PentaBDE phase out.
Temporal trends of PBDEs and emerging flame retardants in belugas from the St. Lawrence Estuary (Canada) and c
Polybrominated diphenyl ethers alter hepatic phosphoenolpyruvate carboxykinase enzyme kinetics in male Wistar
Acute postnatal exposure to the pentaBDE commercial mixture DE-71 at 5 or 15 mg/kg/day does not produce lear
Differences in neonatal neurotoxicity of brominated flame retardants, PBDE 99 and TBBPA, in mice.
Alterations to the circuitry of the frontal cortex following exposure to the polybrominated diphenyl ether mixture,
Trophic level determines levels of brominated flame-retardants in coastal herring gulls.
Geographical distribution of non-PBDE-brominated flame retardants in mussels from Asian coastal waters.
Prenatal transfer of polybrominated diphenyl ethers (PBDEs) results in developmental neurotoxicity in zebrafish la
Associations between serum levels of polybrominated diphenyl ether (PBDE) flame retardants and environmental
Effects of chronic exposure to an environmentally relevant mixture of brominated flame retardants on the reprodu
Tissue-specific accumulation of polybrominated diphenyl ethers (PBDEs) including Deca-BDE and hexabromocycloc
Immune function in female B(6)C(3)F(1) mice is modulated by DE-71, a commercial polybrominated diphenyl ethe
Detection of polybrominated biphenyl ethers (PBDEs) in pediatric hair as a tool for determining in utero exposure.
Lack of effects of some individual polybrominated diphenyl ether (PBDE) and polychlorinated biphenyl (PCB) conge
Epigenetic Effects of Polybrominated Diphenyl Ethers on Human Health.
The occurrence of polybrominated diphenyl ether (PBDE) contamination in soil, water/sediment, and air.
The flame retardant DE-71 (a mixture of polybrominated diphenyl ethers) inhibits human differentiated thyroid ce
Immunologic and endocrine effects of the flame-retardant pentabromodiphenyl ether (DE-71) in C57BL/6J mice.
Fetal exposure to polybrominated diphenyl ethers and the risk of hypospadias: focus on the congeners involved.
Brominated flame retardants in animal derived foods in the Netherlands between 2009 and 2014.
Polybrominated diphenyl ethers (PBDEs) in chicken eggs and cow milk around municipal dumpsites in Abuja, Niger
A noninvasive environmental monitoring tool for brominated flame-retardants (BFRs) assisted by conservation det
Concentrations and loadings of organophosphate and replacement brominated flame retardants in house dust fro
Single-cell RNA-sequencing analysis of estrogen- and endocrine-disrupting chemical-induced reorganization of mou
[Reproductive toxicity of PBDE in males: Advances in studies].
Racial/ethnic and geographic differences in polybrominated diphenyl ether (PBDE) levels across maternal, placenta
Polybrominated biphenyl and diphenylether flame retardants: analysis, toxicity, and environmental occurrence.
Dietary exposure to a binary mixture of polybrominated diphenyl ethers alters innate immunity and disease suscep
Distribution of polybrominated diphenyl ethers in the atmosphere of the Pearl River Delta region, South China.
Association of In Utero Exposure to Polybrominated Diphenyl Ethers With the Risk of Hypospadias.
Concentrations of legacy and novel brominated flame retardants in indoor dust in Melbourne, Australia: An assess
Polybrominated diphenyl ethers (PBDEs) and hydroxylated PBDE metabolites (OH-PBDEs): A six-year temporal tren
Multi-analyte method development for analysis of brominated flame retardants (BFRs) and PBDE metabolites in hu
Temporal trends and developmental patterns of plasma polybrominated diphenyl ether concentrations over a 15-y
The brominated flame retardant PBDE 99 promotes adipogenesis via regulating mitotic clonal expansion and PPAR
A preliminary study on prenatal polybrominated diphenyl ether serum concentrations and intrinsic functional netw
Decreasing but still high levels of halogenated flame retardants in wetland birds in central Spain.

Pine needles as biomonitors of polybrominated diphenyl ethers and emerging flame retardants in the atmosphere
Prenatal polybrominated diphenyl ethers exposure and anogenital distance in boys from a Shanghai birth cohort.
Occurrence, levels and profiles of brominated flame retardants in daily-use consumer products on the Chinese ma
Species and habitat-dependent accumulation and biomagnification of brominated flame retardants and PBDE met
DE-71-induced apoptosis involving intracellular calcium and the Bax-mitochondria-caspase protease pathway in hu
Flame retardants (PBDEs) in marine turtles, dugongs and seafood from Queensland, Australia.
Brominated flame retardants in the environment of Asia-Pacific: an overview of spatial and temporal trends.
A 28-day oral dose toxicity study enhanced to detect endocrine effects of a purified technical pentabromodipheny
Polybrominated diphenyl ethers as endocrine disruptors of adipocyte metabolism.
Desorption and bioavailability of spiked pentabromo diphenyl ether and tetrachlorodibenzo(p)dioxin in contamina
Neurotoxicity of a polybrominated diphenyl ether mixture (DE-71) in mouse neurons and astrocytes is modulated
Electrolytic debromination of PBDEs in DE-83 technical decabromodiphenyl ether.
Exposure to brominated flame retardant PBDE-99 affects cytoskeletal protein expression in the neonatal mouse ce
Bioaccumulation behaviour of polybrominated diphenyl ethers (PBDEs) in a Canadian Arctic marine food web.
Measurement of polybrominated diphenyl ethers on hand wipes: estimating exposure from hand-to-mouth conta
Detection of PBDE effects on mRNA expression in chicken (Gallus domesticus) neuronal cells using real-time RT-PC
Spatial trends of polybrominated diphenyl ethers in avian species: utilization of stored samples in the Environment
Plasma PBDE and thyroxine levels in rats exposed to Bromkal or BDE-47.
Accumulation, tissue-specific distribution and debromination of decabromodiphenyl ether (BDE 209) in European
Polybrominated diphenyl ether in sewage sludge in Germany.
Brominated flame retardants and halogenated phenolic compounds in North American west coast bald eaglet (Hal
Congener distribution of polybrominated diphenyl ethers in feral carp (Cyprinus carpio) from the Llobregat River, S
Variation, levels and profiles of organochlorines and brominated flame retardants in great tit (Parus major) eggs fr
Coexposure of neonatal mice to a flame retardant PBDE 99 (2,2',4,4',5-pentabromodiphenyl ether) and methyl me
Decabromodiphenyl ether (deca-BDE) commercial mixture components, and other PBDEs, in airborne particles at
Flame retardants in placenta and breast milk and cryptorchidism in newborn boys.
PBDEs in circuit boards.
Geographical distribution and accumulation features of PBDEs in human breast milk from Indonesia.
The risk of PBDEs in dust.
The flame retardants, polybrominated diphenyl ethers, are pregnane X receptor activators.
Thyroid insult: flame retardants linked to alterations in pregnant women's TSH levels.
Flame retardants have different effects at high and low doses.
Individual characteristics associated with PBDE levels in U.S. human milk samples.
Toxicity of penta- and decabromodiphenyl ethers after repeated administration to rats: a comparative study.
[Preliminary study of PBDE levels in house dust and human exposure to PBDEs via dust ingestion].
Linking PBDEs in house dust to consumer products using X-ray fluorescence.
Porphyrogenic effect of pentabromodiphenyl ether after repeated administration to rats.
More signs of neurotoxicity of surfactants and flame retardants - Neonatal PFOS and PBDE 99 cause transcriptiona
Multi-generational effects of polybrominated diphenylethers exposure: embryonic exposure of male American kes
Polychlorinated biphenyls and polybrominated diphenyl ethers alter striatal dopamine neurochemistry in synaptos
PBDE exposure from food in Ireland: optimising data exploitation in probabilistic exposure modelling.
Polybrominated diphenyl ether (PBDE) levels in peregrine falcon (Falco peregrinus) eggs from California correlate
The effect of short-term intoxication of rats with pentabromodiphenyl ether (in mixture mimic commercial produc
Reductive debromination of polybrominated diphenyl ethers by anaerobic bacteria from soils and sediments.
Brominated diphenyl ether (BDE) levels in liver, adipose, and milk from adult and juvenile rats exposed by gavage t
Toxicity assessment of air-delivered particle-bound polybrominated diphenyl ethers.
Reproductive and developmental toxicity of a pentabrominated diphenyl ether mixture, DE-71, to ranch mink (Mu

Polybrominated diphenyl ether (PBDE) concentrations in house dust are related to hormone levels in men.
Toxicity of polybrominated diphenyl ethers (DE-71) in chicken (Gallus gallus), mallard (Anas platyrhynchos), and An
The effects of marginal maternal vitamin A status on penta-brominated diphenyl ether mixture-induced alteration
Chronic postnatal DE-71 exposure: effects on learning, attention and thyroxine levels.
Spending time in vehicles can increase PBDE exposure.
Levels and congener specific profiles of PBDEs in human breast milk from China: implication on exposure sources a
Effects of perinatal PBDE exposure on hepatic phase I, phase II, phase III, and deiodinase 1 gene expression involve
In vitro neurotoxicity of PBDE-99: immediate and concentration-dependent effects on protein expression in cerebr
Exposure to DE-71 alters thyroid hormone levels and gene transcription in the hypothalamic-pituitary-thyroid axis
Tea saponin enhanced biodegradation of decabromodiphenyl ether by Brevibacillus brevis.
Methods for synthesis of nonabromodiphenyl ethers and a chloro-nonabromodiphenyl ether.
Photolysis study of perfluoro-2-methyl-3-pentanone under natural sunlight conditions.
Legacy and alternative brominated flame retardants in outdoor dust and pine needles in mainland China: Spatial tr
Intumescent multilayer nanocoating, made with renewable polyelectrolytes, for flame-retardant cotton.
Eco-friendly flame retardant coating deposited on cotton fabrics from bio-based chitosan, phytic acid and divalent
Fully bio-based coating from chitosan and phytate for fire-safety and antibacterial cotton fabrics.
Experimental and modeling analysis of coupled non-Fickian transport and sorption in natural soils.
Microbial degradation of the brominated flame retardant TBNPA by groundwater bacteria: laboratory and field stu
Application of dual carbon-bromine isotope analysis for investigating abiotic transformations of tribromoneopenty
Catalytic degradation of brominated flame retardants by copper oxide nanoparticles.
Aerobic biodegradation of the brominated flame retardants, dibromoneopentyl glycol and tribromoneopentyl alco
Lack of mammalian mutagenicity of the potent bacterial mutagen tris(2,3-dibromopropyl) phosphate and its meta
2-Haloacrylic acids as indicators of mutagenic 2-haloacrolein intermediates in mammalian metabolism of selected
Metabolism and genotoxicity of the halogenated alkyl compound tris(2,3-dibromopropyl)phosphate.
Lack of mammalian mutagenicity of the potent bacterial mutagen tris(2,3-dibromopropyl) phosphate and its meta
Activation mechanism of tris(2,3-dibromopropyl)phosphate to the potent mutagen, 2-bromoacrolein.
Differential in vitro neurotoxicity of the flame retardant PBDE-99 and of the PCB Aroclor 1254 in human astrocytor
Effects of polybrominated biphenyls on the concentration and clearance of steroids from blood.
Aryl hydrocarbon hydroxylase (AHH) induction by polybrominated biphenyls (PBBs): enchancement by photolysis.
Tissue distribution and elimination kinetics of polybrominated biphenyls (PBB) from rat tissue.
Effects of polybrominated biphenyls on the excretion of steroids.
Toxicity and microsomal enzyme induction effects of several polybrominated biphenyls of Firemaster.
Reconstitution of some biochemical and toxicological effects of commercial mixtures of polybrominated biphenyls
Developmental neurotoxicity of polybrominated biphenyls.
Alterations in hepatic mixed-function oxidase activity of rainbow trout after acute treatment with polybrominated
Potent induction of rat liver microsomal, drug-metabolizing enzymes by 2,3,3',4,4',5-hexabromobiphenyl, a compo
Synthesis of tetrakis (hydroxymethyl) phosphonium chloride by high-concentration phosphine in industrial off-gas.
The mutagenic evaluation of tetrakis (hydroxymethyl) phosphonium sulfate using a combined testing protocol app
Chlorinated ethyl and isopropyl phosphoric acid triesters in the indoor environment--an inter-laboratory exposure
Tris(2-chloroethyl)phosphate-induced cell growth arrest via attenuation of SIRT1-independent PI3K/Akt/mTOR pat
Identification of alkaline phosphatase genes for utilizing a flame retardant, tris(2-chloroethyl) phosphate, in Sphing
Tris(2-chloroethyl) phosphate pharmacokinetics in the Fischer 344 rat: a comparison of conventional methods and
Exposure to flame retardant chemicals and occurrence and severity of papillary thyroid cancer: A case-control stuc
Tris(2-chloroethyl) phosphate.
Brain distribution and fate of tris(2-chloroethyl) phosphate in Fischer 344 rats.
Metabolism of tris(2-chloroethyl) phosphate in rats and mice.
Metabolism of the flame retardant plasticizer tris(2-chloroethyl)phosphate by human and rat liver preparations.

Reaction of tris(2-chloroethyl)phosphate with reduced sulfur species.
Tris(2-chloroethyl) phosphate.
Complete detoxification of tris(2-chloroethyl) phosphate by two bacterial strains: Sphingobium sp. strain TCM1 an
Differential modulation of neuro- and interrenal steroidogenesis of juvenile salmon by the organophosphates - tris
Reproductive toxicology. Tris(2-chloroethyl)phosphate.
Tris(2-chloroethyl)phosphate increases ambulatory activity in mice: pharmacological analyses of its neurochemical
Tris (2-chloroethyl) phosphate induces senescence-like phenotype of hepatocytes via the p21Waf1/Cip1-Rb pathw
Tris(2-chloroethyl) phosphate, a pervasive flame retardant: critical perspective on its emissions into the environme
Measuring and modeling surface sorption dynamics of organophosphate flame retardants on impervious surfaces.
Novel aspects of uptake patterns, metabolite formation and toxicological responses in Salmon exposed to the orga
1H-nuclear magnetic resonance metabolomics revealing the intrinsic relationships between neurochemical alterat
Concentrations and variability of organophosphate esters, halogenated flame retardants, and polybrominated dipl
Effects of tris(1-chloro-2-propyl)phosphate and tris(2-chloroethyl)phosphate on cell viability and morphological ch
Urinary metabolites of organophosphate flame retardants in China: Health risk from tris(2-chloroethyl) phosphate
DNA damage and methylation induced by organophosphate flame retardants: Tris(2-chloroethyl) phosphate and tr
Toxicity of tris(2-chloroethyl) phosphate in Daphnia magna after lifetime exposure: Changes in growth, reproductic
A toxicogenomics approach to screen chlorinated flame retardants tris(2-chloroethyl) phosphate and tris(2-chloroi
Toxicity and carcinogenicity of chronic exposure to tris(2-chloroethyl)phosphate.
Organophosphate ester flame retardant concentrations and distributions in serum from inhabitants of Shandong, (
[Effect of oral administration of tris(2-chloroethyl) phosphate to pregnant rats on prenatal and postnatal developm
In vivo and in vitro biological effects of the flame retardants tris(2,3-dibromopropyl) phosphate and tris(2-chloreth
Neurotoxicity of two organophosphorus ester flame retardants in hens.
2-Haloacrylic acids as indicators of mutagenic 2-haloacrolein intermediates in mammalian metabolism of selected
Metabolism and genotoxicity of the halogenated alkyl compound tris(2,3-dibromopropyl)phosphate.
Increased mutagenicity of 1,2-dibromo-3-chloropropane and tris(2,3-dibromopropyl)phosphate in Salmonella TA1
Levels and distributions of organophosphate flame retardants and plasticizers in sediment from Taihu Lake, China.
Synthesis and mutagenicity of selectively methylated analogs of tris(2,3-dibromopropyl)phosphate and 1,2-dibrom
Occurrence, distribution and seasonal variation of organophosphate flame retardants and plasticizers in urban sur
Pathogenesis of toxic and neoplastic renal lesions induced by the flame retardant tris (2,3-dibromopropyl)phospha
Irreversible macromolecular binding of the flame retardant tris-(2, 3-dibromopropyl)phosphate in vitro and in vivo
Diester metabolites of the flame retardant chemicals, tris(1,3-dichloro-2-propyl)phosphate and tris(2,3-dibromopr
Anti-Triser--where are you?
Nephrotoxicity and hepatotoxicity of tris(2,3-dibromopropyl)phosphate in the rat.
Genotoxicity of the flame retardant tris(2,3-dibromopropyl)phosphate in the rat and Drosophila: effects of deuteri
Metabolic activation of tris(2,3-dibromopropyl)phosphate to reactive intermediates. I. Covalent binding and reacti
Metabolic activation of tris(2,3-dibromopropyl)phosphate to reactive intermediates. II. Covalent binding, reactive
Deuterium isotope effect on the metabolism of the flame retardant tris(2,3-dibromopropyl) phosphate in the isola
Quantitative determination of tris-(2,3-dibromopropyl) phosphate by densitometry of thin layer chromatograms.
Comparative genotoxicity and nephrotoxicity studies of the two halogenated flame retardants tris(1,3-dichloro-2-p
Nephrotoxic effect of tris(2,3-dibromopropyl)phosphate on rat urinary metabolites: assessment from 13C-NMR sp
Mutagenicity of derivatives of the flame retardant tris(2,3-dibromopropyl) phosphate: halogenated propanols.
Tris(2,3-dibromopropyl) phosphate.
Nephrotoxicity of the flame retardant tris(2,3-dibromopropyl)phosphate.
Activation mechanism of tris(2,3-dibromopropyl)phosphate to the potent mutagen, 2-bromoacrolein.
Sister chromatid exchanges and growth inhibition induced by the flame retardant tris(2,3-dipromopropyl) phospha
Metabolism in vitro of tris(2,3-dibromopropyl)-phosphate: oxidative debromination and bis(2,3-dibromopropyl)ph
Comparative genotoxicity studies of the flame retardant tris(2,3-dibromopropyl)phosphate and possible metabolit

Children absorb tris-BP flame retardant from sleepwear: urine contains the mutagenic metabolite, 2,3-dibromopro
Metabolism and disposition of the flame retardant tris(2,3-dibromopropyl)phosphate in the rat.
Biodegradation of 14C-tris(2,3-dibromopropyl) phosphate in a laboratory activated sludge system.
In vivo and in vitro biological effects of the flame retardants tris(2,3-dibromopropyl) phosphate and tris(2-chloreth
Rat teratology study of orally administered tris-(2,3-dibromopropyl) phosphate.
A new mutagenic and genotoxic response of the flame retardant tris(2,3-dibromopropyl)phosphate. Activation by
Metabolic activation of bromopropyl compounds.
Toxicogenomic responses of zebrafish embryos/larvae to tris(1,3-dichloro-2-propyl) phosphate (TDCPP) reveal pos
Nephrotoxicity of the flame retardant, tris(2,3-dibromopropyl) phosphate, and its metabolites.
Comparative studies on nephrotoxic effects of tris (2,3-dibromopropyl) phosphate and bis (2,3-dibromopropyl) ph
Metabolism, distribution, and excretion of the flame retardant, tris(2,3-dibromopropyl) phosphate (tris-bp) in the
Species differences in kidney toxicity and metabolic activation of tris(2,3-dibromopropyl)phosphate.
Percutaneous absorption of radiolabeled TRIS from flame-retarded fabric.
Mouse skin carcinogenicity tests of the flame retardants tris(2,3-dibromopropyl)phosphate, tetrakis(hydroxymeth
Developmental exposure to the organophosphorus flame retardant tris(1,3-dichloro-2-propyl) phosphate: estroge
Detection and distribution of Tris(2-chloroethyl) phosphate on the East Antarctic ice sheet.
Predictors of tris(1,3-dichloro-2-propyl) phosphate metabolite in the urine of office workers.
Spatial distribution and inter-year variation of hexabromocyclododecane (HBCD) and tris-(2,3-dibromopropyl) isoc
Comparative body compartment composition and in ovo transfer of organophosphate flame retardants in North A
Complete detoxification of tris(1,3-dichloro-2-propyl) phosphate by mixed two bacteria, Sphingobium sp. strain TC
Tris(2,3-dibromopropyl) phosphate.
Urinary metabolites of organophosphate flame retardants and their variability in pregnant women.
The flame retardant tris (2, 3-dibromopropyl) phosphate: alteration of human cellular DNA.
Time-dependent effects of the flame retardant tris(1,3-dichloro-2-propyl) phosphate (TDCPP) on mRNA expression
Tris(2,3-dibromopropyl) phosphate.
Chronic toxicity evaluation of the flame retardant tris (2-butoxyethyl) phosphate (TBOEP) using Daphnia magna tra
Tris (2,3-dibromopropyl) phosphate nephrotoxicity in the rat: histological and biochemical changes in renal compo
Toxicogenomics of the flame retardant tris (2-butoxyethyl) phosphate in HepG2 cells using RNA-seq.
Renal carcinogenic and nephrotoxic effects of the flame retardant tris(2,3-dibromopropyl) phosphate in F344 rats a
Organophosphate flame retardants and organosiloxanes in predatory freshwater fish from locations across Canada
Kinetics of thermal and photo-initiated release of tris (1,3-dichloro-2-propyl) phosphate (TDCP) flame retardant fro
Organ-specific DNA damage of tris(2,3-dibromopropyl)-phosphate and its diester metabolite in the rat.
Tris(2,3-dibromopropyl) phosphate.
Tris(2,3-dibromopropyl) phosphate.
Flame-resistant sleepwear: have the bird-watchers gone ape?
Toxicokinetic of tris(2-butoxyethyl) phosphate (TBOEP) in humans following single oral administration.
Organophosphorus Flame Retardants and Plasticizers in Building and Decoration Materials and Their Potential Burd
Wastewater analysis of Census day samples to investigate per capita input of organophosphorus flame retardants a
Biotransformation of three phosphate flame retardants and plasticizers in primary human hepatocytes: untargetec
Novel aspects of uptake patterns, metabolite formation and toxicological responses in Salmon exposed to the orga
Effects of tris(2-butoxyethyl) phosphate exposure on endocrine systems and reproduction of zebrafish (Danio rerio
Organophosphate flame retardants (OPFRs) induce genotoxicity in vivo: A survey on apoptosis, DNA methylation, I
Legacy and emerging organophosph?rus flame retardants in car dust from Greece: Implications for human exposur
Acute exposure to tris (2-butoxyethyl) phosphate (TBOEP) affects growth and development of embryo-larval zebra
Multigenerational effects evaluation of the flame retardant tris(2-butoxyethyl) phosphate (TBOEP) using Daphnia r
In ovo tris(2-butoxyethyl) phosphate concentrations significantly decrease in late incubation after a single exposure
Differential modulation of neuro- and interrenal steroidogenesis of juvenile salmon by the organophosphates - tris

Tris(2-butoxyethyl)phosphate and triethyl phosphate alter embryonic development, hepatic mRNA expression, thy
Organophosphate flame retardants and organosiloxanes in predatory freshwater fish from locations across Canada
Effects of TBEP on the induction of oxidative stress and endocrine disruption in Tm3 Leydig cells.
The Flame-Retardant Tris(1,3-dichloro-2-propyl) Phosphate Represses Androgen Signaling in Human Prostate Canc
Organophosphate flame retardants in the indoor air and dust in cars in Japan.
Determination of ammonium sulfamate in air using ion chromatography.
Plasma concentrations of organohalogenated contaminants in white-tailed eagle nestlings - The role of age and die
Spatial distribution and accumulation of brominated flame retardants, polychlorinated biphenyls and organochlori
Levels and temporal trends of chlorinated pesticides, polychlorinated biphenyls and brominated flame retardants i
Performance and storage integrity of dried blood spots for PCB, BFR and pesticide measurements.
Occurrence of brominated flame retardants (BFRs), organochlorine pesticides (OCPs), and polychlorinated bipheny
Biomagnification of persistent chlorinated and brominated contaminants in food web components of the Yellow Se
Multi-class, multi-residue analysis of pesticides, polychlorinated biphenyls, polycyclic aromatic hydrocarbons, poly
Carry-over of dietary organochlorine pesticides, PCDD/Fs, PCBs, and brominated flame retardants to Atlantic salmo
Evaluation of tree bark as a passive atmospheric sampler for flame retardants, PCBs, and organochlorine pesticides
The OBELIX project: early life exposure to endocrine disruptors and obesity.
Assessment of human exposure to indoor organic contaminants via dust ingestion in Pakistan.
Legacy and emerging contaminants in coastal surface sediments around Hainan Island in South China.
Semivolatile organic compounds in homes: strategies for efficient and systematic exposure measurement based on
Preparation and performance features of wristband samplers and considerations for chemical exposure assessmen
Dioxins, PCBs, chlorinated pesticides and brominated flame retardants in free-range chicken eggs from peri-urban
Country-specific chemical signatures of persistent organic pollutants (POPs) in breast milk of French, Danish and Fi
Occurrence and distribution of halogenated flame retardants in an urban watershed: Comparison to polychlorinate
A rapid analytical method to quantify complex organohalogen contaminant mixtures in large samples of high lipid
Tissue-specific accumulation and lactational transfer of polychlorinated biphenyls, chlorinated pesticides, and bror
Flame retardants and organochlorine pollutants in bald eagle plasma from the Great Lakes region.
Body burdens of brominated flame retardants and other persistent organo-halogenated compounds and their des
Brominated flame retardants, polychlorinated biphenyls, and organochlorine pesticides in bird eggs from the Yello
Country-specific chemical signatures of persistent environmental compounds in breast milk.
Comment on "Brominated flame retardants, polychlorinated biphenyls, and organochlorine pesticides in captive gi
Brominated flame retardants, polychlorinated biphenyls, and organochlorine pesticides in captive giant panda (ailu
Maternal levels of endocrine disruptors, polybrominated diphenyl ethers, in early pregnancy are not associated wi
Transplacental transfer of polychlorinated biphenyls and polybrominated diphenyl ethers in arctic beluga whales (D
Polychlorinated biphenyls and polybrominated diphenyl ethers alter striatal dopamine neurochemistry in synaptos
Cross-omics gene and protein expression profiling in juvenile female mice highlights disruption of calcium and zinc
Polychlorinated biphenyls, polybrominated diphenyl ethers, and phthalates in roach from the Seine River basin (Fr
Characterization of polychlorinated biphenyls and brominated flame retardants in sludge, sediment and fish from r
Polybrominated diphenyl ethers (PBDEs) and indicator polychlorinated biphenyls (PCBs) in foods from China: levels
Using the kingfisher (Alcedo atthis) as a bioindicator of PCBs and PBDEs in the dinghushan biosphere reserve, Chin
Metabolic transformation shapes polychlorinated biphenyl and polybrominated diphenyl ether patterns in beluga v
Contamination of indoor dust and air by polychlorinated biphenyls and brominated flame retardants and relevance
Accumulation of brominated flame retardants and polychlorinated biphenyls in human breast milk and scalp hair f
Novel flame retardants (N-FRs), polybrominated diphenyl ethers (PBDEs) and dioxin-like polychlorinated biphenyls
Characterization of polychlorinated biphenyls and brominated flame retardants in sediments from riverine and coa
Induction of monooxygenation in rainbow trout by polybrominated biphenyls: a comparative study.
Polybrominated diphenyl ethers and polychlorinated biphenyls in dust from cars, homes, and offices in Lagos, Nige
Polybrominated diphenyl ethers (PBDEs) and indicator polychlorinated biphenyls (PCBs) in marine fish from four ar

PBDE flame retardants and PCBs in migrating Steller sea lions (Eumetopias jubatus) in the Strait of Georgia, British
PBDE and PCB contamination of eels from the Gironde estuary: from glass eels to silver eels.
Brominated flame retardants and polychlorinated biphenyls in human breast milk from several locations in India: p
Characterization of polychlorinated biphenyls and brominated flame retardants in surface soils from Surabaya, Ind
Human exposure to PCBs, PBDEs and HBCDs in Ghana: Temporal variation, sources of exposure and estimation of
Neurotoxicity of PBDEs on the developing nervous system.
Chicago's Sanitary and Ship Canal sediment: Polycyclic aromatic hydrocarbons, polychlorinated biphenyls, bromina
Neurotoxicity of brominated flame retardants: (in)direct effects of parent and hydroxylated polybrominated diphe
Lack of effects of some individual polybrominated diphenyl ether (PBDE) and polychlorinated biphenyl (PCB) conge
Assessment of the quality of European silver eels and tentative approach to trace the origin of contaminants - A Eu
Spatial and seasonal occurrence of semi-volatile organic compounds (SVOCs) in fish influenced by snowmelt and m
Coastal biomonitoring survey on persistent organic pollutants using oysters (Saccostrea mordax) from Okinawa, Ja
Changes in thyroid hormone related proteins and gene expression induced by polychlorinated biphenyls and halog
Uptake of halogenated organic compounds (HOCs) into peanut and corn during the whole life cycle grown in an ag
Evaluation of the Burdening on the Czech Population by Brominated Flame Retardants.
Halogenated Organic Pollutant Residuals in Human Bared and Clothing-Covered Skin Areas: Source Differentiation
Disruption of thyroid hormone regulated proteins and gene expression by polychlorinated biphenyls, polybromina
C12-30 ?-Bromo-Chloro "Alkenes": Characterization of a Poorly Identified Flame Retardant and Potential Environm
Higher health risk resulted from dermal exposure to PCBs than HFRs and the influence of haze.
Optimization of suspect and non-target analytical methods using GC/TOF for prioritization of emerging contamina
Legacy and Currently Used Organic Contaminants in Human Hair and Hand Wipes of Female E-Waste Dismantling
Baseline survey of marine sediments collected from the State of Kuwait: PAHs, PCBs, brominated flame retardants
Brominated, chlorinated and phosphate organic contaminants in house dust from Portugal.
Seasonality and indoor/outdoor relationships of flame retardants and PCBs in residential air.
Distribution of PBDEs, HBCDs and PCBs in the Brisbane River estuary sediment.
Human Sex Hormone Disrupting Effects of New Flame Retardants and Their Interactions with Polychlorinated Biph
Use of a simple pharmacokinetic model to study the impact of breast-feeding on infant and toddler body burdens
The influence of microplastics and halogenated contaminants in feed on toxicokinetics and gene expression in Eur
Bioaccumulation of Persistent Halogenated Organic Pollutants in Insects: Common Alterations to the Pollutant Pat
Exposure to Environmental Contaminants and Lung Function in Adolescents-Is There a Link?
Toxic effect of PBDE-47 on thyroid development, learning, and memory, and the interaction between PBDE-47 and
Comparative effects of technical toxaphene, 2,5-dichloro-3-biphenylol and octabromodiphenylether on cell viabilit
Brominated flame retardants and halogenated phenolic compounds in North American west coast bald eaglet (Hal
Polybrominated diphenyl ethers, a group of brominated flame retardants, can interact with polychlorinated bipher
Polychlorinated biphenyls and polybrominated diphenyl ethers in the North American atmosphere.
Molecular mechanisms involved in the toxic effects of polychlorinated biphenyls (PCBs) and brominated flame reta
Menstrual function among women exposed to polybrominated biphenyls: a follow-up prevalence study.
Polybrominated diphenyl ethers in the sediments of the Great Lakes. 2. Lakes Michigan and Huron.
Endometriosis among women exposed to polybrominated biphenyls.
Health risks associated with polychlorinated biphenyls.
Latitudinal fractionation of polybrominated diphenyl ethers and polychlorinated biphenyls in frogs (Rana temporar
Occurrence of polychlorinated biphenyls and polybrominated diphenyl ethers in green mussels (Perna viridis) from
PBDEs poised to overtake PCBs in popular fish.
Differential effects of polybrominated diphenyl ethers and polychlorinated biphenyls on [3H]arachidonic acid relea
[Study of between persistent organic pollutants (POPs) content in children vein blood and thyroid stimulating horn
PBCDD and PBCDF from incineration of waste-containing brominated flame retardants.
First world-wide interlaboratory study on polybrominated diphenylethers (PBDEs).

Brominated flame retardants in serum from U.S. blood donors.
Selected chlorobornanes, polychlorinated naphthalenes and brominated flame retardants in Bjørnøya (Bear Island
Accumulation of polychlorinated biphenyls and brominated flame retardants in breast milk from women living in V
E-waste creates hot spots for POPs.
Dust from U.K. primary school classrooms and daycare centers: the significance of dust as a pathway of exposure c
Biochemical and cytogenetic effects in rats caused by short-term ingestion of Aroclor 1254 or Firemaster BP6.
Changes in mitogen-activated protein kinase in cerebellar granule neurons by polybrominated diphenyl ethers and
Prenatal exposure to organohalogens, including brominated flame retardants, influences motor, cognitive, and beh
Relationships between organohalogen contaminants and blood plasma clinical-chemical parameters in chicks of th
Brominated and chlorinated dioxins, PCBs and brominated flame retardants in Scottish shellfish: methodology, occ
Perinatal exposure to brominated flame retardants and polychlorinated biphenyls in Japan.
Brominated flame retardants and polychlorinated biphenyls in fish from the river Scheldt, Belgium.
Polybrominated diphenyl ethers (PBDEs) and polychlorinated biphenyls (PCBs) in 0+ juvenile cyprinids and sedimen
Effects of methyl mercury in combination with polychlorinated biphenyls and brominated flame retardants on the
Bioaccumulation of persistent organic pollutants in female common dolphins (Delphinus delphis) and harbour porp
In Ovo effects of two organophosphate flame retardants--TCPP and TDCPP--on pipping success, development, mRN
Compositional Analysis of Commercial Oligomeric Organophosphorus Flame Retardants Used as Alternatives for P
Tri (2-chloroisopropyl) phosphate--an unexpected organochlorine contaminant in some charcoal air-sampling sorb
Effects of halone 1301 on Lepidium sativum, Petunia hybrida and Phaseolus vulgaris.
[Literature review of the toxicology of the fire-extinguishing agents halon 1301 and 1211 and their decomposition
Grand rounds: outbreak of hematologic abnormalities in a community of people exposed to leakage of fire extingu
Health effects of Halon 1301 exposure.
Setting safe acute exposure limits for halon replacement chemicals using physiologically based pharmacokinetic m
2,5,6,9,10-Pentabromocyclododecanols (PBCDOHs): a new class of HBCD transformation products.
A subchronic toxicity study of Phosflex 51B in Sprague-Dawley rats.
[Hygienic study and evaluation of textile materials with reduced combustibility with reference to the use of the ne
Metabolism and genotoxicity of the halogenated alkyl compound tris(2,3-dibromopropyl)phosphate.
Lack of mammalian mutagenicity of the potent bacterial mutagen tris(2,3-dibromopropyl) phosphate and its meta
Activation mechanism of tris(2,3-dibromopropyl)phosphate to the potent mutagen, 2-bromoacrolein.
Grand rounds: outbreak of hematologic abnormalities in a community of people exposed to leakage of fire extingu
Environmentally friendly flame retardants. A detailed solid-state NMR study of melamine orthophosphate.
Assessment on dietary melamine exposure from tainted infant formula.
Melamine and its derivatives in dog and cat urine: An exposure assessment study.
3D-map modelling for the melting points prediction of intumescent flame-retardant coatings.
Water-based chitosan/melamine polyphosphate multilayer nanocoating that extinguishes fire on polyester-cotton
A survey on occurrence of melamine and its analogues in tainted infant formula in China.
Simultaneous determination of melamine, ammelide, ammeline, and cyanuric acid in milk and milk products by ga
2,4,6-Tribromophenol Disposition and Kinetics in Rodents: Effects of Dose, Route, Sex, and Species.
Biodegradability of tetrabromobisphenol A and tribromophenol by activated sludge.
Biodegradation of typical BFRs 2,4,6-tribromophenol by an indigenous strain Bacillus sp. GZT isolated from e-waste
Contamination trends and factors affecting the transfer of hexabromocyclododecane diastereomers, tetrabromobi
Tribromophenol affects the metabolism, proliferation, migration and multidrug resistance transporters activity of r
Urinary bromophenol glucuronide and sulfate conjugates: Potential human exposure molecular markers for polybr
Emission patterns and risk assessment of polybrominated diphenyl ethers and bromophenols in water and sedime
The microbial degradation of 2,4,6-tribromophenol (TBP) in water/sediments interface: Investigating bioaugmenta
Brominated flame retardants in placental tissues: associations with infant sex and thyroid hormone endpoints.
Brominated dioxins/furans and hydroxylated polybrominated diphenyl ethers: Occurrences in commercial 1,2-bis(2

Environmental concentrations and toxicology of 2,4,6-tribromophenol (TBP).
Toxicogenomic mechanisms of 6-HO-BDE-47, 6-MeO-BDE-47, and BDE-47 in E. coli.
Complete detoxification of tris(1,3-dichloro-2-propyl) phosphate by mixed two bacteria, Sphingobium sp. strain TC
Nonmutagenicity of tetrabromophthalic anhydride and tetrabromophthalic acid in the ames Salmonella/microsom
Toxicity of PHOS-CHEK LC-95A and 259F fire retardants to ocean- and stream-type Chinook salmon and their poter
Construction of flame retardant nanocoating on ramie fabric via layer-by-layer assembly of carbon nanotube and a
Highly efficient flame-retardant and low-smoke-toxicity poly(vinyl alcohol)/alginate/ montmorillonite composite ae
Morphological and thermal properties of cellulose-montmorillonite nanocomposites.
Crumpling and Unfolding of Montmorillonite Hybrid Nanocoatings as Stretchable Flame-Retardant Skin.
Thermal Degradation Characteristic and Flame Retardancy of Polylactide-Based Nanobiocomposites.
Simultaneously Improved Flame Retardance and Ceramifiable Properties of Polymer-Based Composites via the For
Metabolism and disposition of the flame retardant plasticizer, tri-p-cresyl phosphate, in the rat.
Biodegradation of tricresyl phosphate isomers by Brevibacillus brevis: Degradation pathway and metabolic mechar
Aluminum hypophosphite microencapsulated to improve its safety and application to flame retardant polyamide 6
Effect of cellulose acetate butyrate microencapsulated ammonium polyphosphate on the flame retardancy, mecha
Construction of flame retardant nanocoating on ramie fabric via layer-by-layer assembly of carbon nanotube and a
Toxicity of PHOS-CHEK LC-95A and 259F fire retardants to ocean- and stream-type Chinook salmon and their poter
Teratogenic effects of 2,3,7,8-tetrabromodibenzo-p-dioxin and three polybrominated dibenzofurans in C57BL/6N r
Potent induction of rat liver microsomal, drug-metabolizing enzymes by 2,3,3',4,4',5-hexabromobiphenyl, a compo
Effect of cellulose acetate butyrate microencapsulated ammonium polyphosphate on the flame retardancy, mecha
Toxicity assessment and vitellogenin expression in zebrafish (Danio rerio) embryos and larvae acutely exposed to b
Health risks of chemicals in consumer products: A review.
DNA methylation and copy number variation analyses of human embryonic stem cell-derived neuroprogenitors aft
Oxidation of organic contaminants by manganese oxide geomedia for passive urban stormwater treatment system
Characterization of novel ligands of ER?, Er?, and PPAR?: the case of halogenated bisphenol A and their conjugatec
Phosphorus flame retardants and Bisphenol A in indoor dust and PM2.5 in kindergartens and primary schools in H
Anaerobic biotransformation of tetrabromobisphenol A, tetrachlorobisphenol A, and bisphenol A in estuarine sedi
Occurrence of bisphenol-A and its brominated derivatives in tributary and estuary of Xiaoqing River adjacent to Bo
Organophosphate flame retardants and bisphenol A in children's urine in Hong Kong: has the burden been underes
Metabolic perturbation, proliferation and reactive oxygen species jointly contribute to cytotoxicity of human breas
Biodegradability of tetrabromobisphenol A and tribromophenol by activated sludge.
Concentrations of trace metals, phthalates, bisphenol A and flame-retardants in toys and other children's products
Dust measurement of two organophosphorus flame retardants, resorcinol bis(diphenylphosphate) (RBDPP) and bis
Inhibition of thyroid hormone sulfotransferase activity by brominated flame retardants and halogenated phenolics
Embryonic exposure to tetrabromobisphenol A and its metabolites, bisphenol A and tetrabromobisphenol A dimet
Emission patterns and risk assessment of polybrominated diphenyl ethers and bromophenols in water and sedime
Determination of tetrabromobisphenol-A, tetrachlorobisphenol-A and bisphenol-A in soil by ultrasonic assisted ext
Anti-thyroid hormone activity of bisphenol A, tetrabromobisphenol A and tetrachlorobisphenol A in an improved r
Dechloranes 602, 603, 604, Dechlorane Plus, and Chlordene Plus, a newly detected analogue, in tributary sedimen
Manufacture of flame retardant foaming board from waste papers reinforced with phenol-formaldehyde resin.
[Status of the upper respiratory tract and skin of workers manufacturing artificial mineral wool].
The effect of blood on laser-resistant endotracheal tube combustion.
Contact reaction to flame retardant.
Fire-retardant-treated low-formaldehyde-emission particleboard made from recycled wood-waste.
Modulation at a cellular level of the thyroid hormone receptor-mediated gene expression by 1,2,5,6,9,10-hexabro
Novel flame retardants, 1,2-bis(2,4,6-tribromophenoxy)ethane and 2,3,4,5,6-pentabromoethylbenzene, in United
Polymeric brominated flame retardants: are they a relevant source of emerging brominated aromatic compounds

Presence and partitioning properties of the flame retardants pentabromotoluene, pentabromoethylbenzene and h
2,3-Dibromo-1-propanol.
Determination of 2,3-dibromopropanol in air.
Automated solid phase extraction and quantitative measurement of 2,3-dibromo-1-propanol in urine using gas chr
2,3-Dibromopropan-1-ol.
Children absorb tris-BP flame retardant from sleepwear: urine contains the mutagenic metabolite, 2,3-dibromopro
The effects of orthophenylphenol, tris(2,3-dichloropropyl) phosphate, and cyclophosphamide on the immune syst
Tris(dichloropropyl)phosphate, a mutagenic flame retardant: frequent cocurrence in human seminal plasma.
Disposition of the flame retardant, tris(1,3-dichloro-2-propyl) phosphate, in the rat.
Melamine and its derivatives in dog and cat urine: An exposure assessment study.
Simultaneous determination of melamine, ammelide, ammeline, and cyanuric acid in milk and milk products by ga
Fire-retardant-treated low-formaldehyde-emission particleboard made from recycled wood-waste.
Metabolism in vitro of tris(2,3-dibromopropyl)-phosphate: oxidative debromination and bis(2,3-dibromopropyl)ph
Comparative studies on nephrotoxic effects of tris (2,3-dibromopropyl) phosphate and bis (2,3-dibromopropyl) ph
Metabolic activation of tris(2,3-dibromopropyl)phosphate to reactive intermediates. I. Covalent binding and reacti
Enrichment and physiological responses of dechlorane plus on juvenile marine macroalgae (Ulva pertusa).
Dechloranes 602, 603, 604, Dechlorane Plus, and Chlordene Plus, a newly detected analogue, in tributary sedimen
Levels and distribution of Dechlorane Plus in coastal sediments of the Yellow Sea, North China.
Particle-bound Dechlorane Plus and polybrominated diphenyl ethers in ambient air around Shanghai, China.
The effects of Dechlorane Plus on toxicity and mRNA expression in chicken embryos: a comparison of in vitro and i
Historic trends of dechloranes 602, 603, 604, dechlorane plus and other norbornene derivatives and their bioaccu
Dechlorinated Analogues of Dechlorane Plus.
Concentration and bioaccumulation of dechlorane compounds in coastal environment of northern China.
Dechlorane plus and related compounds in peregrine falcon (Falco peregrinus) eggs from Canada and Spain.
Dechlorane plus and possible degradation products in white stork eggs from Spain.
Analytical method for the determination of halogenated norbornene flame retardants in environmental and biota
Biota-sediment accumulation factors for Dechlorane Plus in bottom fish from an electronic waste recycling site, So
Halogenated flame retardants in building and decoration materials in China: Implications for human exposure via i
In ovo transformation of two emerging flame retardants in Japanese quail (Coturnix japonica).
Stereoselective bioaccumulation of syn- and anti-Dechlorane plus isomers in different tissues of common carp (Cy
Development and optimization of gas chromatography coupled to high resolution mass spectrometry based meth
Dechlorane plus in the atmosphere and precipitation near the Great Lakes.
Dechlorane Plus and related compounds in aquatic and terrestrial biota: a review.
Sources and environmental behavior of dechlorane plus--a review.
Dechlorane Plus in human hair from an e-waste recycling area in South China: comparison with dust.
Examination of isomer specific bioaccumulation parameters and potential in vivo hepatic metabolites of syn- and a
Isomers of Dechlorane Plus flame retardant in the eggs of herring gulls (Larus argentatus) from the Laurentian Gre
Isomer-specific bioaccumulation and trophic transfer of Dechlorane Plus in the freshwater food web from a highly
Determination of Dechlorane Plus in serum from electronics dismantling workers in South China.
Compounds structurally related to Dechlorane Plus in sediment and biota from Lake Ontario (Canada).
Detection of Dechlorane Plus in fish from urban-industrial rivers.
Dechlorane Plus pollution and inventory in soil of Huai'an City, China.
Levels and isomer profiles of Dechlorane Plus in the surface soils from e-waste recycling areas and industrial areas
Dechlorane plus in multimedia in northeastern Chinese urban region.
Large-scale distribution of dechlorane plus in air and seawater from the Arctic to Antarctica.
Levels, occurrence and human exposure to novel brominated flame retardants (NBFRs) and Dechlorane Plus (DP) i
Concentrations and sources of Dechlorane Plus in sewage sludge.

Bioaccumulation of several brominated flame retardants and dechlorane plus in waterbirds from an e-waste recyc
Is the urban-adapted ring-billed gull a biovector for flame retardants?
New brominated flame retardants and dechlorane plus in the Arctic: Local sources and bioaccumulation potential
Distributions, influencing factors, and risk assessment of Dechlorane Plus and related compounds in surficial water
Amplification effect of haze on human exposure to halogenated flame retardants in atmospheric particulate matte
Dechlorane Plus flame retardant in a contaminated frog species: Biomagnification and isomer-specific transfer fro
Tissue distribution, maternal transfer, and age-related accumulation of dechloranes in Chinese sturgeon.
Oral repeat dose and reproductive toxicity of the chlorinated flame retardant Dechlorane Plus.
Investigating Dechlorane Plus (DP) distribution and isomer specific adsorption behavior in size fractionated marine
Levels and profiles of Dechlorane Plus in a major E-waste dismantling area in China.
Assessment on the occupational exposure of manufacturing workers to Dechlorane Plus through blood and hair ar
Alternative flame retardants, Dechlorane Plus and BDEs in the blubber of harbour porpoises (Phocoena phocoena)
Potential genotoxicity and risk assessment of a chlorinated flame retardant, Dechlorane Plus.
Dechlorane Plus in paired hair and serum samples from e-waste workers: correlation and differences.
Novel brominated flame retardants and dechlorane plus in Greenland air and biota.
Trophic magnification of chlorinated flame retardants and their dechlorinated analogs in a fresh water food web.
Dechlorane Plus in eggs of two gull species (Larus michahellis and Larus audouinii) from the southwestern Mediter
Isomers of Dechlorane Plus in an aquatic environment in a highly industrialized area in Southern China: spatial and
Occurrence, behavior and human health risk assessment of dechlorane plus and related compounds in indoor dust
Sources, gastrointestinal absorption and stereo-selective and tissue-specific accumulation of Dechlorane Plus (DP)
Distribution patterns of brominated, chlorinated, and phosphorus flame retardants with particle size in indoor and
Dechlorane plus and other flame retardants in tree bark from the northeastern United States.
Kinetics of stereoselective enrichment of Dechlorane Plus in Ulva Pertusa.
Sex-dependent accumulation and maternal transfer of Dechlorane Plus flame retardant in fish from an electronic v
Short-term effects of Dechlorane Plus on the earthworm Eisenia fetida determined by a systems biology approach
Dechlorane Plus induces oxidative stress and decreases cyclooxygenase activity in the blue mussel.
Dechlorane Plus and decabromodiphenyl ether in atmospheric particles of northeast Asian cities.
Gastrointestinal absorption, dynamic tissue-specific accumulation, and isomer composition of dechlorane plus and
Current halogenated flame retardant concentrations in serum from residents of Shandong Province, China, and te
Species- and tissue-specific accumulation of Dechlorane Plus in three terrestrial passerine bird species from the Pe
Responses of mouse liver to dechlorane plus exposure by integrative transcriptomic and metabonomic studies.
Dechlorane Plus in serum from e-waste recycling workers: influence of gender and potential isomer-specific metab
Dechlorane Plus and its dechlorinated analogs from an e-waste recycling center in maternal serum and breast milk
Accumulation pattern of Dechlorane Plus and associated biological effects on rats after 90 d of exposure.
Dechlorane Plus flame retardant in kingfishers (Alcedo atthis) from an electronic waste recycling site and a referen
Dechlorane Plus flame retardant in terrestrial raptors from northern China.
Dechlorane plus monoadducts in a Lake Ontario (Canada) food web and biotransformation by lake trout (Salvelinu
Accumulation of Dechlorane Plus flame retardant in terrestrial passerines from a nature reserve in South China: th
Brominated flame retardants and dechlorane plus on a remote high mountain of the eastern Tibetan Plateau: impl
In vitro biotransformation of decabromodiphenyl ether (BDE-209) and Dechlorane Plus flame retardants: a case st
Measurement and health risk assessment of PM2.5, flame retardants, carbonyls and black carbon in indoor and ou
Distribution and region-specific sources of Dechlorane Plus in marine sediments from the coastal East China Sea.
Evaluation of the Genotoxic and Physiological Effects of Decabromodiphenyl Ether (BDE-209) and Dechlorane Plus
Sources and environmental behaviors of Dechlorane Plus and related compounds - A review.
Toxicological responses following short-term exposure through gavage feeding or water-borne exposure to Dechlo
Evidence for Anaerobic Dechlorination of Dechlorane Plus in Sewage Sludge.
Brominated flame retardant (BFRs) and Dechlorane Plus (DP) in paired human serum and segmented hair.

Detection of Dechlorane Plus and brominated flame retardants in marketed fish in Japan.
Accumulation and effects of 90-day oral exposure to Dechlorane Plus in quail (Coturnix coturnix).
Uptake, depuration, bioaccumulation, and selective enrichment of dechlorane plus in common carp (Cyprinus carp
Validation and Application of a 3-Step Sequential Extraction Method to Investigate the Fraction Transformation of (
Comparative study of dechlorane plus (DP) in adult chickens and developing embryos: Stereo-selective bioaccumu
Effects of dechlorane plus on intestinal barrier function and intestinal microbiota of Cyprinus carpio L.
Assessment of Dechlorane Plus and related compounds in foodstuffs and estimates of daily intake from Lebanese
Correlations between dechlorane plus concentrations in paired hair and indoor dust samples and differences betw
Toxic effects of dechlorane plus on the common carp (Cyprinus carpio) embryonic development.
New data on a widely used flame retardant.
[Levels and sources of decabromodiphenyl ether and dechlorane plus in Xining and Tianjun, Qinghai Province, Chir
Dechlorane plus, a chlorinated flame retardant, in the Great Lakes.
Detection of dechlorane plus in residential indoor dust in the city of Ottawa, Canada.
Dechlorane plus levels in sediment of the lower Great Lakes.
Spatial distribution and hazard of halogenated flame retardants and polychlorinated biphenyls to common kingfish
Flame retardancy and UV protection of cotton based fabrics using nano ZnO and polycarboxylic acids.
Highly sensitive and selective photoelectrochemical biosensor platform for polybrominated diphenyl ether detecti
The effect of blood on laser-resistant endotracheal tube combustion.
Effect of repeated administration of hexabromobenzene and 1,2,4,5-tetrabromobenzene on the levels of selected
The effect of selected aromatic bromine derivatives on the activity of glutathione peroxidase and transferase.
The in vitro metabolism of 2,2',4,4',5,5'-hexabromobiphenyl.
Effect of congeners of polybrominated biphenyls on hatchability of chicken eggs. I. 2,2',4,4',5,5'-Hexabromobiphen
Detrimental effects of flame retardant, PBB153, exposure on sperm and future generations.
Exposure to PBB-153 and Digit Ratio.
Occurrence of polybrominated diphenyl ethers (PBDEs) and 2,2',4,4',5,5'-hexabromobiphenyl (BB-153) in water sa
Temporal trends and spatial distributions of brominated flame retardants in archived fishes from the Great Lakes.
Brominated organic contaminants in the liver and egg of the common cormorants (Phalacrocorax carbo) from Japa
Anaerobic transformation of polybrominated biphenyls with the goal of identifying unknown hexabromobiphenyls
Occurrence of selected polybrominated diphenyl ethers and 2,2',4,4',5,5'-hexabromobiphenyl (BB-153) in sewage
Mono-substituted isopropylated triaryl phosphate, a major component of Firemaster 550, is an AHR agonist that e
Neurotoxicity of FireMaster 550® in zebrafish (Danio rerio): Chronic developmental and acute adolescent exposure
First insight into human extrahepatic metabolism of flame retardants: Biotransformation of EH-TBB and Firemaste
Urinary tetrabromobenzoic acid (TBBA) as a biomarker of exposure to the flame retardant mixture Firemaster® 55
Metabolic activation of tris(2,3-dibromopropyl)phosphate to reactive intermediates. I. Covalent binding and reacti
Metabolism in vitro of tris(2,3-dibromopropyl)-phosphate: oxidative debromination and bis(2,3-dibromopropyl)ph
Comparative studies on nephrotoxic effects of tris (2,3-dibromopropyl) phosphate and bis (2,3-dibromopropyl) ph
Carry-over of dietary organochlorine pesticides, PCDD/Fs, PCBs, and brominated flame retardants to Atlantic salm
2,3,7,8-Tetrachlorodibenzo-p-dioxin.
Desorption and bioavailability of spiked pentabromo diphenyl ether and tetrachlorodibenzo(p)dioxin in contamina
Antagonism of TCDD-induced ethoxyresorufin-O-deethylation activity by polybrominated diphenyl ethers (PBDEs)
Psychological effects upon exposure to polyhalogenated dibenzodioxins and dibenzofurans.
Brominated organic contaminants in the liver and egg of the common cormorants (Phalacrocorax carbo) from Japa
Facile synthesis of bromo- and mixed bromo/chloro dibenzo-p-dioxins and [14C]-labeled 1,3,7,8-tetrabromodibenz
Exposure to Environmental Contaminants and Lung Function in Adolescents-Is There a Link?
Polychlorinated dibenzo-p-dioxins, dibenzofurans, and flame retardants in northern gannet (Morus bassanus) eggs
Cross-omics gene and protein expression profiling in juvenile female mice highlights disruption of calcium and zinc
Assessment of characteristic distribution of PCDD/Fs and BFRs in sludge generated at municipal and industrial was

Detection of high PBDD/Fs levels and dioxin-like activity in toys using a combination of GC-HRMS, rat-based and hu
Occurrence, composition, source, and regional distribution of halogenated flame retardants and polybrominated c
Brominated dioxins/furans and hydroxylated polybrominated diphenyl ethers: Occurrences in commercial 1,2-bis(2
Emissions, environmental levels, sources, formation pathways, and analysis of polybrominated dibenzo-p-dioxins a
Facile synthesis of bromo- and mixed bromo/chloro dibenzo-p-dioxins and [14C]-labeled 1,3,7,8-tetrabromodibenz
Brominated and chlorinated dioxins, PCBs and brominated flame retardants in Scottish shellfish: methodology, occ
Vinyl bromide.
Identification of the brominated flame retardant 1,2-dibromo-4-(1,2-dibromoethyl)cyclohexane as an androgen ag
TBECH, 1,2-dibromo-4-(1,2 dibromoethyl) cyclohexane, alters androgen receptor regulation in response to mutatic
Toxicologic effects of 28-day dietary exposure to the flame retardant 1,2-dibromo-4-(1,2-dibromoethyl)-cyclohexar
The Effects of the Organic Flame-Retardant 1,2-Dibromo-4-(1,2-dibromoethyl) Cyclohexane (TBECH) on Androgen S
Disruption of thyroxine and sex hormones by 1,2-dibromo-4-(1,2-dibromoethyl)cyclohexane (DBE-DBCH) in Americ
1,2-Dibromo-4-(1,2-dibromoethyl)-cyclohexane and tris(methylphenyl) phosphate cause significant effects on deve
Toxicokinetics of tetrabromoethylcyclohexane (TBECH) in juvenile brown trout (Salmo trutta) and effects on plasma
1,2-Dibromo-4-(1,2 dibromoethyl) cyclohexane (TBECH)-mediated steroid hormone receptor activation and gene r
An assessment of in ovo toxicity of the flame retardant 1,2-dibromo-4-(1,2-dibromoethyl) cyclohexane (TBECH) in
Alpha and beta isomers of tetrabromoethylcyclohexane (TBECH) flame retardant: depletion and metabolite format
The flame retardant ?-1,2-dibromo-4-(1,2-dibromoethyl)cyclohexane: fate, fertility, and reproductive success in Am
Accumulation of polybrominated diphenyl ethers, hexabromobenzene, and 1,2-dibromo-4-(1,2-dibromoethyl)cyclo
Structure characterization and thermal stabilities of the isomers of the brominated flame retardant 1,2-dibromo-4
Identilication of the novel cycloaliphatic brominated flame retardant 1,2-dibromo-4-(1,2-dibromoethyl)cyclohexan
Exposure to the androgenic brominated flame retardant 1,2-dibromo-4-(1,2-dibromoethyl)-cyclohexane alters rep
Tetrabromoethylcyclohexane affects gonadal differentiation and development in the frog Pelophylax nigromaculat
Evaluation of development, locomotor behavior, oxidative stress, immune responses and apoptosis in developing z
Is the current-use flame retardant, DBE-DBCH, a potential obesogen? Effects on body mass, fat content and associa
Tetrabromoethylcyclohexane (TBECH) exhibits immunotoxicity in murine macrophages.
Diastereomers of the brominated flame retardant 1,2-dibromo-4-(1,2 dibromoethyl)cyclohexane induce androgen
Biotransformation of the Flame Retardant 1,2-Dibromo-4-(1,2-dibromoethyl)cyclohexane (TBECH) in Vitro by Hum
Technical pentabromodiphenyl ether and hexabromocyclododecane as activators of the pregnane-X-receptor (PXR
Hexabromocyclododecane inhibits depolarization-induced increase in intracellular calcium levels and neurotransm
Effects of metals on the transformation of hexabromocyclododecane (HBCD) in solvents: implications for solvent-b
Analysis of hexabromocyclododecane diastereomers and enantiomers by liquid chromatography/tandem mass spe
Modulation at a cellular level of the thyroid hormone receptor-mediated gene expression by 1,2,5,6,9,10-hexabror
Analysis of brominated flame retardants in styrenic polymers. Comparison of the extraction efficiency of ultrasonic
Transformation of hexabromocyclododecane in contaminated soil in association with microbial diversity.
Solvent effects on quantitative analysis of brominated flame retardants with Soxhlet extraction.
Dermal bioaccessibility of flame retardants from indoor dust and the influence of topically applied cosmetics.
Antioxidant gene expression and metabolic responses of earthworms (Eisenia fetida) after exposure to various con
Acute effects of hexabromocyclododecane on Leydig cell cyclic nucleotide signaling and steroidogenesis in vitro.
Responses of growth inhibition and antioxidant gene expression in earthworms (Eisenia fetida) exposed to tetrabro
Hexabromocyclododecane in human breast milk: levels and enantiomeric patterns.
Personal exposure to HBCDs and its degradation products via ingestion of indoor dust.
Bioaccumulation of polybrominated diphenyl ethers and hexabromocyclododecane in the northwest Atlantic mari
Hexabromocyclododecane-induced developmental toxicity and apoptosis in zebrafish embryos.
Proteomic studies in zebrafish liver cells exposed to the brominated flame retardants HBCD and TBBPA.
Hexabromocyclododecane decreases the lytic function and ATP levels of human natural killer cells.
Developmental toxicity of brominated flame retardants, tetrabromobisphenol A and 1,2,5,6,9,10-hexabromocyclo

Effects of the brominated flame retardant hexabromocyclododecane (HBCD) on dopamine-dependent behavior ar
Isolation of Pseudomonas sp. strain HB01 which degrades the persistent brominated flame retardant gamma-hexa
Cytotoxicity evaluation of three pairs of hexabromocyclododecane (HBCD) enantiomers on Hep G2 cell.
Transfer of brominated flame retardants from components into dust inside television cabinets.
Assessing the toxicity of TBBPA and HBCD by zebrafish embryo toxicity assay and biomarker analysis.
Endocrine effects of hexabromocyclododecane (HBCD) in a one-generation reproduction study in Wistar rats.
Temporal trends, congener patterns, and sources of octa-, nona-, and decabromodiphenyl ethers (PBDE) and hexal
Brominated flame retardants in fish of Lake Geneva (Switzerland).
Sediment record and atmospheric deposition of brominated flame retardants and organochlorine compounds in La
Determination of flame-retardant hexabromocyclododecane diastereomers in textiles.
Simultaneous determination of hexabromocyclododecanes and tris (2,3-dibromopropyl) isocyanurate using LC-AP(
Brominated flame retardants and perfluorinated chemicals, two groups of persistent contaminants in Belgian hum
Trophodynamics of hexabromocyclododecanes and several other non-PBDE brominated flame retardants in a fresl
Thermally-induced transformation of hexabromocyclo dodecanes and isobutoxypenta bromocyclododecanes in fla
The potential of selected brominated flame retardants to affect neurological development.
Toxicokinetics of the flame retardant hexabromocyclododecane gamma: effect of dose, timing, route, repeated ex
Bioavailability of hexabromocyclododecane to the polychaete Hediste diversicolor: exposure through sediment an
Hexabromocyclododecane determination in seafood samples collected from Japanese coastal areas.
Determination of brominated flame retardants in food by LC-MS/MS: diastereoisomer-specific hexabromocyclodo
Characterization of polychlorinated biphenyls and brominated flame retardants in sediments from riverine and coa
Spatial diastereomer patterns of hexabromocyclododecane (HBCD) in a Norwegian fjord.
Accumulation and disposition of hexabromocyclododecane (HBCD) in juvenile rainbow trout (Oncorhynchus mykis
Brominated flame retardants in the Arctic environment--trends and new candidates.
Hexabromocyclododecane decreases tumor-cell-binding capacity and cell-surface protein expression of human na
New perspective on the determination of flame retardants in sewage sludge by using ultrahigh pressure liquid chro
Polybrominated diphenyl ethers (PBDEs) and hexabromocyclodecane (HBCD) in composite U.S. food samples.
Biomagnification of anthropogenic and naturally-produced organobrominated compounds in a marine food web fr
Isobutoxypentabromocyclododecanes (iBPBCDs): a new class of polybrominated compounds.
Spatial distribution of hexabromocyclododecanes (HBCDs), polybrominated diphenyl ethers (PBDEs) and organoch
Pipping success, isomer-specific accumulation, and hepatic mRNA expression in chicken embryos exposed to HBCD
Altered thyroxine metabolism in rainbow trout (Oncorhynchus mykiss) exposed to hexabromocyclododecane (HBC
Subacute effects of the brominated flame retardants hexabromocyclododecane and tetrabromobisphenol A on he
More clues to HBCD isomer mystery.
Levels of hexabromocyclododecane in harbor porpoises and common dolphins from western European seas, with
Structure elucidation of hexabromocyclododecanes--a class of compounds with a complex stereochemistry.
Spatial distribution and vertical profile of polybrominated diphenyl ethers and hexabromocyclododecanes in sedim
A chemical and toxicological profile of Dutch North Sea surface sediments.
Distribution of hexabromocyclododecane in Detroit River suspended sediments.
Distribution and transportability of hexabromocyclododecane (HBCD) in the Asia-Pacific region using skipjack tuna
Neurotoxicity of the pentabrominated diphenyl ether mixture, DE-71, and hexabromocyclododecane (HBCD) in rat
Hexabromocyclododecanes (HBCDs) in the environment and humans: a review.
New multiresidue analytical method dedicated to trace level measurement of brominated flame retardants in hum
Effects of the brominated flame retardants hexabromocyclododecane (HBCDD), and tetrabromobisphenol A (TBBP
HBCDD-induced sustained reduction in mitochondrial membrane potential, ATP and steroidogenesis in peripuberta
Congener-specific analysis of hexabromocyclododecane by high-performance liquid chromatography/electrospray
The environmental occurrence of hexabromocyclododecane in Sweden.
More flame-proofed fish.

Determination of bromophenols as dioxin precursors in combustion gases of fire retarded extruded polystyrene by
Brominated flame retardants in Alburnus alburnus from Cinca River Basin (Spain).
Distribution and fate of HBCD and TBBPA brominated flame retardants in North Sea estuaries and aquatic food we
Occurrence and bioavailability of polybrominated diphenyl ethers and hexabromocyclododecane in sediment and
Hexabromocyclododecanes in indoor dust from Canada, the United Kingdom, and the United States.
Brominated flame retardants in the environment of Asia-Pacific: an overview of spatial and temporal trends.
Brominated flame retardants in fish and shellfish - levels and contribution of fish consumption to dietary exposure
Two-generation reproductive toxicity study of the flame retardant hexabromocyclododecane in rats.
Temporal trends of polybrominated diphenyl ethers and hexabromocyclododecane in milk from Stockholm mothe
Polybrominated diphenyl ethers and HBCD in bird eggs of South Africa.
Maternal transfer of brominated flame retardants in zebrafish (Danio rerio).
Subacute effects of hexabromocyclododecane (HBCD) on hepatic gene expression profiles in rats.
More flame retardants found in house dust.
Solid-state conformations and absolute configurations of (+) and (-) alpha-, beta-, and gamma-hexabromocyclodoc
Synthesis of the two minor isomers, delta- and epsilon-1,2,5,6,9,10-hexabromocyclododecane, present in commer
Detection of hexabromocyclododecane and its metabolite pentabromocyclododecene in chicken egg and fish from
Regio- and stereoselective isomerization of hexabromocyclododecanes (HBCDs): kinetics and mechanism of beta-H
Factors influencing enantiomeric fractions of hexabromocyclododecane measured using liquid chromatography/ta
Biomagnification of polybrominated diphenyl ether and hexabromocyclododecane flame retardants in the polar b
Tetrabromobisphenol A (TBBPA) and hexabromocyclododecanes (HBCDs) in tissues of humans, dolphins, and shark
Time trend of hexabromocyclododecane in the breast milk of Japanese women.
Brominated flame retardants in North-East Atlantic marine ecosystems.
Consumption of fish from a contaminated lake strongly affects the concentrations of polybrominated diphenyl eth
Indoor pollutant hexabromocyclododecane enhances house dust mite-induced activation of human monocyte-der
Accumulation of ?-hexabromocyclododecane (?-HBCDD) in tissues of fast- and slow-growing broilers (Gallus dome
Impairment in the mesohippocampal dopamine circuit following exposure to the brominated flame retardant, HB(
Deriving freshwater safety thresholds for hexabromocyclododecane and comparison of toxicity of brominated flan
Effects of the amendment of biochars and carbon nanotubes on the bioavailability of hexabromocyclododecanes (
Complete catalytic debromination of hexabromocyclododecane using a silica-supported palladium catalyst in alkali
Serum Metabolomic Profiles in Neonatal Mice following Oral Brominated Flame Retardant Exposures to Hexabrom
Demographic and temporal trends of hexabromocyclododecanes (HBCDD) in an Australian population.
Hexabromocyclododecane and tetrabromobisphenol A in sediments and paddy soils from Liaohe River Basin, Chin
Transfer of hexabromocyclododecane flame retardant isomers from captive American kestrel eggs to feathers and
Levels and distribution of tris-(2,3-dibromopropyl) isocyanurate and hexabromocyclododecanes in surface sedimer
Characterization of brominated flame retardants in construction and demolition waste components: HBCD and PB
Concentration of novel brominated flame retardants and HBCD in leachates and sediments from selected municipa
Biotransformation of hexabromocyclododecanes with hexachlorocyclohexane-transforming Sphingobium chinhate
Hens can ingest extruded polystyrene in rearing buildings and lay eggs contaminated with hexabromocyclododeca
Preliminary screening of polybrominated diphenyl ethers (PBDEs), hexabromocyclododecane (HBCDD) and tetrabr
Dietary exposure assessment of Chinese population to tetrabromobisphenol-A, hexabromocyclododecane and dec
Hexabromocyclododecane diastereomers in fish and suspended particulate matter from selected European waters
Emerging and legacy flame retardants in UK human milk and food suggest slow response to restrictions on use of F
Brominated flame retardants, tetrabromobisphenol A and hexabromocyclododecane, activate mitogen-activated p
Matrix-specific distribution and diastereomeric profiles of hexabromocyclododecane (HBCD) in a multimedia envir
1,2,5,6,9,10-?Hexabromocyclododecane (HBCD) impairs thyroid hormone-induced dendrite arborization of Purkin
Hexabromocyclododecane-induced Genotoxicity in Cultured Human Breast Cells through DNA Damage.
Determination of hexabromocyclododecanes in sediments from the Haihe River in China by an optimized HPLC-MS

A national survey of tetrabromobisphenol-A, hexabromocyclododecane and decabrominated diphenyl ether in hu
Alteration of Diastereoisomeric and Enantiomeric Profiles of Hexabromocyclododecanes (HBCDs) in Adult Chicken
Potential of gas chromatography-atmospheric pressure chemical ionization-tandem mass spectrometry for screeni
Discrimination of hexabromocyclododecane from new polymeric brominated flame retardant in polystyrene foam
Levels and distributions of polybrominated diphenyl ethers, hexabromocyclododecane, and tetrabromobisphenol
Environmental risks of HBCDD from construction and demolition waste: a contemporary and future issue.
Brominated flame retardants - Exposure and risk assessment for the general population.
Leaching behaviour of hexabromocyclododecane from treated curtains.
Short-term effects of a perinatal exposure to the HBCDD ?-isomer in rats: Assessment of early motor and sensory
Transcriptomic and metabolomic approaches to investigate the molecular responses of human cell lines exposed t
Hexabromocyclododecane flame retardant in Antarctica: Research stations as sources.
Hexabromocyclododecane and tetrabromobisphenol A alter secretion of interferon gamma (IFN-?) from human im
Evaluation of 3D-human skin equivalents for assessment of human dermal absorption of some brominated flame
Hexabromocyclododecane affects benthic-pelagic coupling in an experimental ecosystem.
Hexabromocyclododecanes (HBCDDs) in surface soils from coastal cities in North China: Correlation between diast
Does the source migration pathway of HBCDs to household dust influence their bio-accessibility?
Tetrabromobisphenol A and hexabromocyclododecane alter secretion of IL-1? from human immune cells.
Brominated flame retardant emissions from the open burning of five plastic wastes and implications for environm
Long-term emissions of hexabromocyclododecane as a chemical of concern in products in China.
New Insights into the Cytotoxic Mechanism of Hexabromocyclododecane from a Metabolomic Approach.
Hexabromocyclododecane (HBCD) induced changes in the liver proteome of eu- and hypothyroid female rats.
Direct contact between dust and HBCD-treated fabrics is an important pathway of source-to-dust transfer.
Brominated flame retardants (BFRs): A review on environmental contamination in China.
Brominated flame retardants, hexabromocyclododecane and tetrabromobisphenol A, affect proinflammatory prot
Emerging halogenated flame retardants and hexabromocyclododecanes in food samples from an e-waste processi
Inhomogeneity of sediment samples in analysis of hexabromocyclododecane.
The effects of prosperity indices and land use indicators of an urban conurbation on the occurrence of hexabromo
Treatability of hexabromocyclododecane using Pd/Fe nanoparticles in the soil-plant system: Effects of humic acids.
Hexabromocyclododecane (HBCD): A case study applying tiered testing for human health risk assessment.
Distribution and diastereoisomeric profiles of hexabromocyclododecanes in air, water, soil, and sediment samples
Serum measures of hexabromocyclododecane (HBCDD) and polybrominated diphenyl ethers (PBDEs) in reproducti
Bioaccumulation and translocation of tetrabromobisphenol A and hexabromocyclododecanes in mangrove plants
Temporal-spatial distribution and diastereoisomer pattern of hexabromocyclododecane in the vicinity of a chemica
Rat strain response differences upon exposure to technical or alpha hexabromocyclododecane.
Dietary exposure and risk assessment of exposure to hexabromocyclododecanes in a Taiwan population.
Hepatic transcriptional dose-response analysis of male and female Fischer rats exposed to hexabromocyclododeca
Diastereoisomer-specific neurotoxicity of hexabromocyclododecane in human SH-SY5Y neuroblastoma cells.
[Dermatological evaluation of a flame retardant, hexabromocyclododecane (HBCD) on guinea pig by using the prin
Analysis of polybrominated diphenyl ethers, hexabromocyclododecanes, and legacy and emerging phosphorus fla
Coastal biomonitoring survey on persistent organic pollutants using oysters (Saccostrea mordax) from Okinawa, Ja
Cytotoxicity of hexabromocyclododecane, 1,2-dibromo-4-(1,2-dibromoethyl) cyclohexane and 1,2,5,6-tetrabromo
Hexabromocyclododecane in riverine and estuarine sediments from Osaka, Japan: spatial distribution and concent
Distribution, diastereomer-specific accumulation and associated health risks of hexabromocyclododecanes (HBCD
Biodegradation of hexabromocyclododecane by Rhodopseudomonas palustris YSC3 strain: A free-living nitrogen-fi
Tetrabromobisphenol A and hexabromocyclododecane isomers in breast milk from the general population in Beiji
Fate of Hexabromocyclododecane (HBCD), A Common Flame Retardant, In Polystyrene-Degrading Mealworms: Ele
The enrichment and purification of hexabromocyclododecanes and its effects on thyroid in zebrafish.

Evaluating hexabromocyclododecane (HBCD) toxicokinetics in humans and rodents by physiologically based pharm
The HBCDs biodegradation using a Pseudomonas strain and its application in soil phytoremediation.
Atmospheric concentrations of hexabromocyclododecane (HBCDD) diastereomers in the Great Lakes region.
Hexabromocyclododecane in polystyrene packaging: A downside of recycling?
Legacy and emerging brominated flame retardants in China: A review on food and human milk contamination, hur
Hexabromocyclododecanes (HBCDs) in fish: Evidence of recent HBCD input into the coastal environment.
Occurrence of polybrominated diphenylethers, hexabromocyclododecanes, bromophenols and tetrabromobispher
Bioconcentration and effects of hexabromocyclododecane exposure in crucian carp (Carassius auratus).
Bioaccumulation and Distribution of Hexabromocyclododecane Isomers in Duck Tissues.
Flame retardants, hexabromocyclododecane (HCBD) and tetrabromobisphenol a (TBBPA), alter secretion of tumor
Development and validation of a quantitative UHPLC-MS/MS method for selected brominated flame retardants in
Liver volatolomics to reveal poultry exposure to ?-hexabromocyclododecane (HBCD).
Hexabromocyclododecane and tetrabromobisphenol A in tree bark from different functional areas of Shanghai, Ch
Simultaneous liquid chromatography-tandem mass spectrometry analysis of brominated flame retardants (tetrabr
Hexabromocyclododecane: concentrations and isomer profiles from sources to environmental sinks.
Concentrations, Distributions, and Risk Assessment of HBCD in Sediment in the Weihe River Basin in Northwest Ch
Legacy and alternative brominated flame retardants in outdoor dust and pine needles in mainland China: Spatial tr
Elucidating the Variability in the Hexabromocyclododecane Diastereomer Profile in the Global Environment.
Factors influencing risk assessments of brominated flame-retardants; evidence based on seafood from the North E
Contamination trends and factors affecting the transfer of hexabromocyclododecane diastereomers, tetrabromobi
Occurrence of tetrabromobisphenol a (TBBPA) and hexabromocyclododecane (HBCD) in soil and road dust in Chon
Stereoisomer-Specific Trophodynamics of the Chiral Brominated Flame Retardants HBCD and TBECH in a Marine Fo
Biomagnification of Hexabromocyclododecane (HBCD) in a coastal ecosystem near a large producer in China: Hum
Hexabromocyclododecanes in breast milk from residents in Shenzhen, China: Implications for infant exposure.
Application of triolein-embedded cellulose acetate membrane (TECAM) passive sampler to study phase distributio
Brominated Flame Retardants in Sediments of Four Coastal Lagoons of Yucatan, Mexico.
Dietary exposure to brominated flame retardants and risk of type 2 diabetes in the French E3N cohort.
Hexabromocyclododecane (HBCD) stereoisomers in U.S. food from Dallas, Texas.
Simultaneous extraction and determination of HBCD isomers and TBBPA by ASE and LC-MSMS in fish.
In situ accumulation of HBCD, PBDEs, and several alternative flame-retardants in the bivalve (Corbicula fluminea) a
2,5,6,9,10-Pentabromocyclododecanols (PBCDOHs): a new class of HBCD transformation products.
[Determination of three brominated flame retardants in human serum using solid-phase extraction coupled with u
Levels, isomer profiles and chiral signatures of particle-bound hexabromocyclododecanes in ambient air around Sh
Transient aberration of neuronal development in the hippocampal dentate gyrus after developmental exposure to
Developmental toxicity evaluation of three hexabromocyclododecane diastereoisomers on zebrafish embryos.
Tissue-specific accumulation of polybrominated diphenyl ethers (PBDEs) including Deca-BDE and hexabromocycloc
Vapor pressure of three brominated flame retardants determined by using the Knudsen effusion method.
Comparisons of polybrominated diphenyl ether and hexabromocyclododecane concentrations in dust collected wi
Tri-decabrominated diphenyl ethers and hexabromocyclododecane in indoor air and dust from Stockholm microer
Diasteroisomer and enantiomer-specific profiles of hexabromocyclododecane and tetrabromobisphenol A in an ac
Levels of tetrabromobisphenol A, hexabromocyclododecanes and polybrominated diphenyl ethers in human milk f
Three decades (1983-2010) of contaminant trends in East Greenland polar bears (Ursus maritimus). Part 2: bromin
Hexabromocyclododecanes in surface sediments and a sediment core from Rivers and Harbor in the northern Chir
Co-release of hexabromocyclododecane (HBCD) and Nano- and microparticles from thermal cutting of polystyrene
Predictors of tetrabromobisphenol-A (TBBP-A) and hexabromocyclododecanes (HBCD) in milk from Boston mother
Tris(2,3-dibromopropyl) isocyanurate, hexabromocyclododecanes, and polybrominated diphenyl ethers in mollusk
Diastereoisomer- and enantiomer-specific accumulation, depuration, and bioisomerization of hexabromocyclodod

Levels and distribution of hexabromocyclododecane (HBCD) in environmental samples near manufacturing faciliti
Spatial distribution and inter-year variation of hexabromocyclododecane (HBCD) and tris-(2,3-dibromopropyl) isoc
Policy relevant results from an expert elicitation on the human health risks of decabromodiphenyl ether (decaBDE
Brominated flame retardants in the Australian population: 1993-2009.
Detection of Dechlorane Plus and brominated flame retardants in marketed fish in Japan.
Monitoring of hexabromocyclododecane diastereomers in fish from European freshwaters and estuaries.
Determinations of hexabromocyclododecane (HBCD) isomers in channel catfish, crayfish, hen eggs and fish feeds f
[Effects of perinatal exposure to the brominated flame-retardant hexabromocyclododecane (HBCD) on the develo
Crystal structure of ?-isobutoxypentabromo-cyclododecanes, kinetics and selectivity of their isomerization during
Temporal trend (1988-2008) of hexabromocyclododecane enantiomers in herring gull eggs from the German coas
A review of the analysis of novel brominated flame retardants.
Biomonitoring-based risk assessment for hexabromocyclododecane (HBCD).
Occurrence, sources, and inventory of hexabromocyclododecanes (HBCDs) in soils from Chongming Island, the Yar
Hexabromocyclododecanes (HBCDs) in marine fishes along the Chinese coastline.
Quantitative determination of the diastereoisomers of hexabromocyclododecane in human plasma using liquid ch
Fate of tetrabromobisphenol A and hexabromocyclododecane brominated flame retardants in soil and uptake by
Levels of brominated flame retardants and methoxylated polybrominated diphenyl ethers in eggs of white-tailed s
Diastereoisomer- and enantiomer-specific determination of hexabromocyclododecane in fish oil for food and feed
Brominated flame retardants in Canadian chicken egg yolks.
Reproductive changes in American kestrels (Falco sparverius) in relation to exposure to technical hexabromocycloc
Do temporal and geographical patterns of HBCD and PBDE flame retardants in U.S. fish reflect evolving industrial u
Kinetic study of ?-hexabromocyclododecane orally given to laying hens (Gallus domesticus). "Transfer of HBCD in l
Toxicokinetics and carry-over model of ?-hexabromocyclododecane (HBCD) from feed to consumption-sized Atlant
Preparation and X-ray structural characterization of further stereoisomers of 1,2,5,6,9,10-hexabromocyclododeca
Temporal trends of polybrominated diphenyl ethers and hexabromocyclododecane in Swedish Peregrine Falcon (F
Dietary intake of hexabromocyclododecane diastereoisomers (?-, ?-, and ?-HBCD) in the Belgian adult population.
Hexabromocyclododecane enantiomers: microsomal degradation and patterns of hydroxylated metabolites.
Human exposure to PCBs, PBDEs and HBCDs in Ghana: Temporal variation, sources of exposure and estimation of
Assessing oestrogenic effects of brominated flame retardants hexabromocyclododecane and tetrabromobispheno
Toxicokinetics of the flame retardant hexabromocyclododecane alpha: effect of dose, timing, route, repeated exp
Brominated flame retardants in dust from UK cars--within-vehicle spatial variability, evidence for degradation and
Differences in tissue distribution of HBCD alpha and gamma between adult and developing mice.
Evaluation of spatial distribution and accumulation of novel brominated flame retardants, HBCD and PBDEs in an I
Destruction behavior of hexabromocyclododecanes during incineration of solid waste containing expanded and ex
PBDE, HBCD, and novel brominated flame retardant contamination in sediments from Lake Maggiore (Northern Ita
Determination of hexabromocyclododecane by flowing atmospheric pressure afterglow mass spectrometry.
Occurrence of additive brominated flame retardants in aquatic organisms from Tai Lake and Yangtze River in Easte
Prenatal exposure to the brominated flame retardant hexabromocyclododecane (HBCD) impairs measures of sust
The transformation of hexabromocyclododecane using zerovalent iron nanoparticle aggregates.
Brominated flame retardant exposure of aircraft personnel.
Cross-omics gene and protein expression profiling in juvenile female mice highlights disruption of calcium and zinc
Exposure to an environmentally relevant mixture of brominated flame retardants affects fetal development in Spr
Hexabromocyclododecane in polystyrene based consumer products: an evidence of unregulated use.
Levels of flame retardants HBCD, TBBPA and TBC in surface soils from an industrialized region of East China.
The brominated flame retardants, PBDEs and HBCD, in Canadian human milk samples collected from 1992 to 2005
Flame retardants and legacy contaminants in polar bears from Alaska, Canada, East Greenland and Svalbard, 2005-
Occurrence of brominated flame retardants and perfluoroalkyl substances in fish from the Czech aquatic ecosyste

Rapid identification of polystyrene foam wastes containing hexabromocyclododecane or its alternative polymeric b
Temporal trends in classical and alternative flame retardants in bird eggs from Doñana Natural Space and surround
Selective damage to dopaminergic transporters following exposure to the brominated flame retardant, HBCDD.
Concentrations of Polybrominated Diphenyl Ethers, Hexabromocyclododecanes and Tetrabromobisphenol-A in Bre
Study of novel pure culture HBCD-1, effectively degrading Hexabromocyclododecane, isolated from an anaerobic r
Effects of benthos, temperature, and dose on the fate of hexabromocyclododecane in experimental coastal ecosys
Uptake, distribution, depletion, and in ovo transfer of isomers of hexabromocyclododecane flame retardant in diet
Hexabromocyclododecane Flame Retardant Isomers in Sediments from Detroit River and Lake Erie of the Laurentia
Emerging flame retardants, PBDEs, and HBCDDs in indoor and outdoor media in Stockholm, Sweden.
Destruction of the flame retardant hexabromocyclododecane in a full-scale municipal solid waste incinerator.
Review of hexabromocyclododecane (HBCD) with a focus on legislation and recent publications concerning toxicok
Enrichment of hexabromocyclododecanes in coastal sediments near aquaculture areas and a wastewater treatmen
Hexabromocyclododecanes in limnic and marine organisms and terrestrial plants from Tianjin, China: diastereome
Brominated flame retardant concentrations in sera from the Canadian Health Measures Survey (CHMS) from 2007
The determination of perfluoroalkyl substances, brominated flame retardants and their metabolites in human brea
The sarcoplasmic-endoplasmic reticulum Ca(2+)-ATPase (SERCA) is the likely molecular target for the acute toxicity
Hexabromocyclododecane concentrations in Canadian human fetal liver and placental tissues.
Tetrabromobisphenol A and hexabromocyclododecane flame retardants in infant-mother paired serum samples, a
Hexabromocyclododecanes in crucian carp and sediment from the major rivers in Korea.
Photolysis of brominated flame retardants in textiles exposed to natural sunlight.
Polybrominated diphenyl ethers (PBDEs) and hexabromocyclododecane (HBCD) in seven different marine bird spec
Effects of low-level hexabromocyclododecane (HBCD) exposure on cardiac development in zebrafish embryos.
A two-dimensional HPLC separation for the enantioselective determination of hexabromocyclododecane (HBCD) is
The fate of ?-hexabromocyclododecane in female C57BL/6 mice.
Emission behavior of hexabromocyclododecanes and polybrominated diphenyl ethers from flame-retardant-treate
Impaired lipid and glucose homeostasis in hexabromocyclododecane-exposed mice fed a high-fat diet.
Levels and trends of PBDEs and HBCDs in the global environment: status at the end of 2012.
Oligomeric proanthocyanidins alleviate hexabromocyclododecane-induced cytotoxicity in HepG2 cells through reg
New perspectives on diastereoselective determination of hexabromocyclododecane traces in fish by ultra high per
Uptake and translocation of organophosphates and other emerging contaminants in food and forage crops.
Occurrence of organic phosphates in particulate matter of the vehicle exhausts and outdoor environment - A case
Tissue-specific distribution and bioaccumulation potential of organophosphate flame retardants in crucian carp.
Tris(2-butoxyethyl)phosphate and triethyl phosphate alter embryonic development, hepatic mRNA expression, thy
Distribution Pattern of Organophosphate Esters in Particle-Size Fractions of Urban Topsoils Under Different Land-U
Organophosphate di- and tri-esters in indoor and outdoor dust from China and its implications for human exposure
Flame retardants in the indoor environment -- Part II: release of VOCs (triethylphosphate and halogenated degrada
Organophosphate Flame Retardants in Soils of Zhejiang Province, China: Levels, Distribution, Sources, and Exposur
[Toxicokinetic aspect of the potential use of sulfur hexafluoride in sealed quarters].
Sharing unexpected biomarker results with study participants.
The determination of perfluoroalkyl substances, brominated flame retardants and their metabolites in human brea
Quantification of tetrabromo benzoic acid and tetrabromo phthalic acid in rats exposed to the flame retardant Uni
Comparison of in vitro hormone activities of novel flame retardants TBB, TBPH and their metabolites TBBA and TB
Nonmutagenicity of tetrabromophthalic anhydride and tetrabromophthalic acid in the ames Salmonella/microsom
Decomposition of 2-bromophenol in NaOH solution at high temperature.
Mechanisms of dioxin formation from the high-temperature pyrolysis of 2-bromophenol.
Synthesis of polybrominated diphenyl ethers via symmetrical tetra- and hexabrominated diphenyliodonium salts.
Tea saponin enhanced biodegradation of decabromodiphenyl ether by Brevibacillus brevis.

Polybrominated diphenyl ethers (PBDEs) in the indoor dust in China: levels, spatial distribution and human exposu
Hair as a biomarker of systemic exposure to polybrominated diphenyl ethers.
Investigation of polybrominated diphenyl ethers in old consumer products in India.
Congener distribution of polybrominated diphenyl ethers in feral carp (Cyprinus carpio) from the Llobregat River, S
An efficient GC-IDMS method for determination of PBDEs and PBB in plastic materials.
Toxic effects of two brominated flame retardants BDE-47 and BDE-183 on the survival and protein expression of th
Trophic level determines levels of brominated flame-retardants in coastal herring gulls.
Is BDE-175 an important enough component of commercial octabromodiphenyl ether mixtures to be listed in Ann
Widespread polybrominated diphenyl ether (PBDE) contamination of urban soils in Melbourne, Australia.
Levels and distribution of polybrominated diphenyl ethers (PBDEs) in the freshwater environment surrounding a P
Detection of hexabromocyclododecane and its metabolite pentabromocyclododecene in chicken egg and fish from
Biodegradation of hexabromocyclododecane by Rhodopseudomonas palustris YSC3 strain: A free-living nitrogen-fi
Personal exposure to HBCDs and its degradation products via ingestion of indoor dust.
[Hygienic study and evaluation of textile materials with reduced combustibility with reference to the use of the ne
Reproductive toxicity of 2,2-bis(bromomethyl)-1,3-propanediol in a continuous breeding protocol in Swiss (CD-1) r
Carcinogenic activity of the flame retardant, 2,2-bis(bromomethyl)-1,3-propanediol in rodents, and comparison wi
Reproductive toxicology. 2,2-bis(bromomethyl)-1,3-propanediol.
Functional genomic assessment of 2, 2-bis (bromomethyl)-1, 3-propanediol induced cytotoxicity in a single-gene k
Kidney and urinary bladder lesions in F344/N rats and B6C3F1 mice after 13 weeks of 2,2-bis(bromomethyl)-1,3-pr
Induction of DNA damage in human urothelial cells by the brominated flame retardant 2,2-bis(bromomethyl)-1,3-
2,2-bis(Bromomethyl)-1,3-propanediol (technical grade).
Chemical degradation of 2,2-bis(bromomethyl)propan-1,3-diol (DBNPG) in alkaline conditions.
Oxidative debromination of 2,2-bis(bromomethyl)-1,3-propanediol by UV/persulfate process and corresponding fo
Tea saponin enhanced biodegradation of decabromodiphenyl ether by Brevibacillus brevis.
Effect of cadmium ion on biodegradation of decabromodiphenyl ether (BDE-209) by Pseudomonas aeruginosa.
DNA methylation and copy number variation analyses of human embryonic stem cell-derived neuroprogenitors aft
In vitro immune toxicity of polybrominated diphenyl ethers on murine peritoneal macrophages: apoptosis and imr
Responses of growth inhibition and antioxidant gene expression in earthworms (Eisenia fetida) exposed to tetrabr
Effect of decabromodiphenyl ether (BDE-209) on a soil-biota system: Role of earthworms and ryegrass.
Oxidative stress and renal toxicity after subacute exposure to decabrominated diphenyl ether in Wistar rats.
The phytotoxicities of decabromodiphenyl ether (BDE-209) to different rice cultivars (Oryza sativa L.).
The effects of decabromodiphenyl ether on glycolipid metabolism and related signaling pathways in mice.
Dispersive liquid-liquid microextraction followed by reversed phase HPLC for the determination of decabrominate
Low level exposure to the flame retardant BDE-209 reduces thyroid hormone levels and disrupts thyroid signaling
Important role of reaction field in photodegradation of deca-bromodiphenyl ether: theoretical and experimental i
Bioavailability and half-life of decabromodiphenyl ether (BDE-209) in rat.
Efficient oxidative debromination of decabromodiphenyl ether by TiO2-mediated photocatalysis in aqueous enviro
Decabromodiphenyl ether in indoor dust from different microenvironments in a university in the Philippines.
Postnatal exposure to low-dose decabromodiphenyl ether adversely affects mouse testes by increasing thyrosine
Determination of PBDEs, HBB, PBEB, DBDPE, HBCD, TBBPA and related compounds in sewage sludge from Catalon
Estimating European historical production, consumption and atmospheric emissions of decabromodiphenyl ether.
Characterizing the optimal operation of photocatalytic degradation of BDE-209 by nano-sized TiO2.
In vitro biotransformation of decabromodiphenyl ether (BDE-209) and Dechlorane Plus flame retardants: a case st
Photolysis of brominated flame retardants in textiles exposed to natural sunlight.
Effects of decabromodiphenyl ether (BDE-209) on mRNA transcription of thyroid hormone pathway and spermato
Vapor pressure of solid polybrominated diphenyl ethers determined via Knudsen effusion method.
Trophic level determines levels of brominated flame-retardants in coastal herring gulls.

Decabromodiphenyl ether (BDE-209) enters the food web of the River Po and is metabolically debrominated in res
Decabromodiphenyl oxide.
Advanced UV/H?O? oxidation of deca-bromo diphenyl ether in sediments.
Investigation of polybrominated diphenyl ethers in old consumer products in India.
Dechlorane Plus and decabromodiphenyl ether in atmospheric particles of northeast Asian cities.
Neurodevelopmental effects of decabromodiphenyl ether (BDE-209) in APOE transgenic mice.
Toxicogenomic analysis of the ability of brominated flame retardants TBBPA and BDE-209 to disrupt thyroid hormo
Polybrominated diphenyl ethers (PBDEs), hexabromocyclododecane (HBCD) and "novel" brominated flame retarda
What do the data show? Knowledge map development for comprehensive environmental assessment.
Effects of BDE-209 contaminated sediments on zebrafish development and potential implications to human health
Dietary exposure of American kestrels (Falco sparverius) to decabromodiphenyl ether (BDE-209) flame retardant: u
Oxidative stress biomarkers in freshwater fish Carassius auratus exposed to decabromodiphenyl ether and ethane,
[Levels and sources of decabromodiphenyl ether and dechlorane plus in Xining and Tianjun, Qinghai Province, Chir
An efficient GC-IDMS method for determination of PBDEs and PBB in plastic materials.
Bioaccumulation kinetics of polybrominated diphenyl ethers and decabromodiphenyl ethane from field-collected s
Long term effects of murine postnatal exposure to decabromodiphenyl ether (BDE-209) on learning and memory a
Polybrominated diphenyl ethers listed as Stockholm Convention POPs, other brominated flame retardants and hea
Formation of polybrominated dibenzofurans (PBDFs) after heating of a salmon sample spiked with decabromodiph
Organophosphate and halogenated flame retardants in atmospheric particles from a European Arctic site.
Biodegradation of decabromodiphenyl ether (BDE-209) by white-rot fungus Phlebia lindtneri.
Toxicological, gene expression and histopathological evaluations of environmentally realistic concentrations of poly
Policy relevant results from an expert elicitation on the human health risks of decabromodiphenyl ether (decaBDE
Microbial electricity generation enhances decabromodiphenyl ether (BDE-209) degradation.
[Biodegradation of decabromodiphenyl ether by intracellular enzyme obtained from Pseudomonas aeruginosa].
Dust measurement of two organophosphorus flame retardants, resorcinol bis(diphenylphosphate) (RBDPP) and bis
Bioaccumulation and bound-residue formation of 14C-decabromodiphenyl ether in an earthworm-soil system.
Exposure to flame retardant chemicals and occurrence and severity of papillary thyroid cancer: A case-control stuc
Occurrence and levels of polybrominated diphenyl ethers in surface sediments from the Yellow River Estuary, Chin
Aerobic degradation of BDE-209 by Enterococcus casseliflavus: Isolation, identification and cell changes during deg
Polybrominated diphenyl ethers (PBDEs) and alternative brominated flame retardants (aBFRs) in sediments from fo
The environmental fate of polybrominated diphenyl ethers (PBDEs) in western Taiwan and coastal waters: evaluati
Comparative Study of Genotoxicity Induced by Six Different PBDEs.
Concentrations and distributions of polybrominated diphenyl ethers and novel brominated flame retardants in tre
The protective effects of insulin-like growth factor-1 on neurochemical phenotypes of dorsal root ganglion neurons
Distribution pattern of legacy and "novel" brominated flame retardants in different particle size fractions of indoor
[Research progress of health effect of polybrominated diphenyl ethers].
Relationships between polybrominated diphenyl ethers and transcription and activity of type 1 deiodinase in a gul
Evaluation of the Genotoxic and Physiological Effects of Decabromodiphenyl Ether (BDE-209) and Dechlorane Plus
Occurrence of PBDEs and alternative halogenated flame retardants in sewage sludge from the industrial city of Gu
Determination and human exposure assessment of polybrominated diphenyl ethers and tetrabromobisphenol A ir
Neonatal exposure to BDE 209 impaired learning and memory, decreased expression of hippocampal core SNAREs
In vivo reporter gene mutation and micronucleus assays in gpt delta mice treated with a flame retardant decabron
A national survey of tetrabromobisphenol-A, hexabromocyclododecane and decabrominated diphenyl ether in hur
Emerging and legacy flame retardants in UK human milk and food suggest slow response to restrictions on use of P
Maternal exposure to polybrominated diphenyl ether (BDE-209) during lactation affects germ cell survival with alte
Serum levels of decabromodiphenyl ether (BDE-209) in women from different European countries and possible rel
Dietary exposure assessment of Chinese population to tetrabromobisphenol-A, hexabromocyclododecane and dec

Polybrominated diphenyl ether flame retardant concentrations in faeces from young children in Queensland, Austr
Brominated and organophosphorus flame retardants in body wipes and house dust, and an estimation of house du
Trends of production, consumption and environmental emissions of Decabromodiphenyl ether in mainland China.
Widespread polybrominated diphenyl ether (PBDE) contamination of urban soils in Melbourne, Australia.
Simulating long-term occupational exposure to decabrominated diphenyl ether using C57BL/6 mice: biodistributio
Developmental exposure to the polybrominated diphenyl ether PBDE 209: Neurobehavioural and neuroprotein an
Exploring the interactions of decabrominateddiphenyl ether and tetrabromobisphenol A with human serum album
Complete debromination of decabromodiphenyl ether using the integration of Dehalococcoides sp. strain CBDB1 a
Photolytic degradation products of two highly brominated flame retardants cause cytotoxicity and mRNA expressi
Hair as a biomarker of systemic exposure to polybrominated diphenyl ethers.
Polybrominated diphenyl ethers (PBDEs) in the indoor dust in China: levels, spatial distribution and human exposu
A first European scale multimedia fate modelling of BDE-209 from 1970 to 2020.
Uptake and transport mechanisms of decabromodiphenyl ether (BDE-209) by rice (Oryza sativa).
Declines in polybrominated diphenyl ether contamination of San Francisco Bay following production phase-outs ar
Brominated flame retardants in Korean river sediments, including changes in polybrominated diphenyl ether conce
Observation-Based Assessment of PBDE Loads in Arctic Ocean Waters.
Metabolic pathways of decabromodiphenyl ether (BDE209) in rainbow trout (Oncorhynchus mykiss) via intraperito
Emission characteristics of PBDEs during flame-retardant plastics extruding process: field investigation and laborat
Bioremediation of wastewaters with decabromodiphenyl ether by anaerobic granular sludge.
Probing the debromination of the flame retardant decabromodiphenyl ether in sediments of a boreal lake.
Biodegradation of Decabromodiphenyl Ether (BDE-209) by Crude Enzyme Extract from Pseudomonas aeruginosa.
Review on the occurrence and profiles of polybrominated diphenyl ethers in the Philippines.
Levels and distributions of polybrominated diphenyl ethers, hexabromocyclododecane, and tetrabromobisphenol
Isolation and characterization of two novel psychrotrophic decabromodiphenyl ether-degrading bacteria from rive
Effect of polybrominated diphenyl ether (BDE-209) on testicular steroidogenesis and spermatogenesis through alte
Children's exposure to polybrominated diphenyl ethers (PBDEs) through mouthing toys.
Spatial distribution of old and emerging flame retardants in Chinese forest soils: sources, trends and processes.
Confirmation of the presence of the flame retardant decabromobiphenyl ether in river sediment from Osaka, Japa
Motor deficits, impaired response inhibition, and blunted response to methylphenidate following neonatal exposu
Spatial Distribution of Organophosphorus and Brominated Flame Retardants in Surface Water, Sediment, Groundw
Stocks, flows and emissions of DBDPE in China and its international distribution through products and waste.
Inhibition of progesterone biosynthesis induced by deca-brominated diphenyl ether (BDE-209) in mouse Leydig tu
Occupational exposure to polybrominated diphenyl ethers or decabromodiphenyl ethane during chemical manufa
Polybrominated diphenyl ethers and alternative halogenated flame retardants in mollusks from the Chinese Bohai
Mass balance study of brominated flame retardants in female captive peregrine falcons.
Toxicogenomic analyses of the effects of BDE-47/209, TBBPA/S and TCBPA on early neural development with a hu
BDE-209 induces male reproductive toxicity via cell cycle arrest and apoptosis mediated by DNA damage response
A noninvasive environmental monitoring tool for brominated flame-retardants (BFRs) assisted by conservation det
Oral exposure to BDE-209 modulates metastatic spread of melanoma in C57BL/6 mice inoculated with B16-F10 ce
A comparison of the thyroid disruption induced by decabrominated diphenyl ethers (BDE-209) and decabromodipl
Disposition of decabromobiphenyl ether in rats dosed intravenously or by feeding.
Cardiovascular toxicity of decabrominated diphenyl ethers (BDE-209) and decabromodiphenyl ethane (DBDPE) in r
Workplace environmental exposure level guide: decabromodiphenyl oxide.
Determination of decabromobiphenyl ether in water and sediment samples by gas chromatography with electron
Behavioral effects of oral subacute exposure to BDE-209 in young adult mice: a preliminary study.
Levels of dechlorane plus and polybrominated diphenylethers in human milk in two Canadian cities.
Synergistic effect of microscale zerovalent iron particles combined with anaerobic sludges on the degradation of de

Tissue-specific accumulation of polybrominated diphenyl ethers (PBDEs) including Deca-BDE and hexabromocycloc
[Horizontal and vertical distribution of polybrominated diphenyl ethers (PBDEs) in river sediment from a typical ele
Assessment of characteristic distribution of PCDD/Fs and BFRs in sludge generated at municipal and industrial wast
[Pollution characterization and source apportionment of polybrominated diphenyl ethers in autumn air of Xi'an].
[Effect of heavy metals on degradation of BDE-209 by white-rot fungus].
Photocatalytic debromination of preloaded decabromodiphenyl ether on the TiO(2) surface in aqueous system.
Formation of brominated dibenzofurans from the photolysis of flame retardant decabromobiphenyl ether in hexar
[Remediation of Decabromodiphenyl Ether Contaminated Sediment Through Plant Roots Enhanced by Exogenous
Solid surface-mediated photochemical transformation of decabromodiphenyl ether (BDE-209) in aqueous solution
Children's exposure to brominated flame retardants in indoor environments - A review.
Exposure to decabromodiphenyl ether (BDE-209) produces mitochondrial dysfunction in rat liver and cell death.
Developmental exposure of decabromodiphenyl ether impairs subventricular zone neurogenesis and morphology
Decabromodiphenyl Ether (DecaBDE) in Electrical and Electronic Equipment in Japan: Stock, Emission, and Substitu
The environmental pollutant BDE-209 regulates NO/cGMP signaling through activation of NMDA receptors in neur
PBDEs and novel brominated flame retardants in road dust from northern Vietnam: Levels, congener profiles, emis
Studies on the interaction of BDE-47 and BDE-209 with acetylcholinesterase (AChE) based on the neurotoxicity thr
Occurrence of polybrominated diphenylethers, hexabromocyclododecanes, bromophenols and tetrabromobispher
Concentrations of legacy and novel brominated flame retardants in indoor dust in Melbourne, Australia: An assess
Bioaccumulation of decabromodiphenyl ether affects the antioxidant system in the clam Mactra veneriformis.
Halogenated flame retardants in building and decoration materials in China: Implications for human exposure via i
Evaluation of DNA damage induced by 2 polybrominated diphenyl ether flame retardants (BDE-47 and BDE-209) in
Distribution of polybrominated diphenyl ethers in the atmosphere of the Pearl River Delta region, South China.
Seasonal variations of polybrominated flame retardants bound to car dust under Mediterranean climate.
Disruption of thyroid hormone levels by decabrominated diphenyl ethers (BDE-209) in occupational workers from
Legacy and alternative brominated flame retardants in outdoor dust and pine needles in mainland China: Spatial tr
Is the urban-adapted ring-billed gull a biovector for flame retardants?
Effect of decabrominated diphenyl ether exposure on spatial learning and memory, the expression and phosphory
Decabromodiphenyl ether exacerbates hyperglycemia in diet-induced obese mice.
Temporal trends of decabromodiphenyl ether and emerging brominated flame retardants in dust, air and window
MicroRNA-21 attenuates BDE-209-induced lipid accumulation in THP-1 macrophages by downregulating Toll-like re
Occurrence, levels and profiles of brominated flame retardants in daily-use consumer products on the Chinese ma
Spatiotemporal variability of polybrominated diphenyl ether concentration in atmospheric fine particles in Shenzh
Bioaccumulation and biotransformation of decabromodiphenyl ether and effects on daily growth in juvenile lake w
Toxicity of penta- and decabromodiphenyl ethers after repeated administration to rats: a comparative study.
Toxicology and human health assessment of decabromodiphenyl ether.
Patterns and concentration levels of polybrominated diphenyl ethers (PBDEs) in placental tissue of women in Denr
Effects of decabrominated diphenyl ether (PBDE 209) exposure at different developmental periods on synaptic pla
In vivo and in vitro toxicity of decabromodiphenyl ethane, a flame retardant.
Identification and quantification of products formed via photolysis of decabromodiphenyl ether.
Effect of Tween 80 and beta-cyclodextrin on degradation of decabromodiphenyl ether (BDE-209) by White rot fung
An international survey of decabromodiphenyl ethane (deBDethane) and decabromodiphenyl ether (decaBDE) in s
Comment on: Effects of decabrominated diphenyl ether (PBDE 209) exposure at different developmental periods c
Determination of decabromodiphenyl ether in backcoated textile preparation.
Linking PBDEs in house dust to consumer products using X-ray fluorescence.
Measurement of polybrominated diphenyl ethers on hand wipes: estimating exposure from hand-to-mouth contac
A 28-day oral dose toxicity study in Wistar rats enhanced to detect endocrine effects of decabromodiphenyl ether
Electrolytic debromination of PBDEs in DE-83 technical decabromodiphenyl ether.

EPA feels heat over flame retardant.
Developmental neurotoxicity: when research succeeds through inappropriate statistics.
Brominated flame retardants in North-East Atlantic marine ecosystems.
Decabromodiphenyl ether (deca-BDE) commercial mixture components, and other PBDEs, in airborne particles at
Polybrominated diphenyl ethers (PBDEs) in sediment by salinity and land-use type from Australia.
Mass balance of decabromodiphenyl ethane and decabromodiphenyl ether in a WWTP.
Bioavailability of decabromodiphenyl ether to the marine polychaete Nereis virens.
Sample characterization: a priori to evaluating absorption, distribution, and metabolism.
Developmental exposure to decabrominated diphenyl ether (BDE-209): effects on sperm oxidative stress and chro
Accumulation and debromination of decabromodiphenyl ether (BDE-209) in juvenile fathead minnows (Pimephale
Comparative hepatic microsomal biotransformation of selected PBDEs, including decabromodiphenyl ether, and de
Temporal trends of polybrominated diphenyl ethers and hexabromocyclododecane in Swedish Peregrine Falcon (Fa
Tissue distribution of decabrominated diphenyl ether (BDE-209) and its metabolites in sucking rat pups after prena
[Mouse sperm DNA damage induced by exogenous BDE-209].
Predominance of BDE-209 and other higher brominated diphenyl ethers in eggs of white stork (Ciconia ciconia) col
Behavior of decabromodiphenyl ether (BDE-209) in soil: effects of rhizosphere and mycorrhizal colonization of ryeg
Is decabromodiphenyl ether (BDE-209) a developmental neurotoxicant?
Behavioral changes in aging but not young mice after neonatal exposure to the polybrominated flame retardant de
Characterization of maternal transfer of decabromodiphenyl ether (BDE-209) administered to pregnant Sprague-D
Effects of gestational exposure to decabromodiphenyl ether on reproductive parameters, thyroid hormone levels,
Health risk characterization for resident inhalation exposure to particle-bound halogenated flame retardants in a t
Time-of-flight secondary ion mass spectrometry imaging demonstrates the specific localization of deca-bromo-dip
Understanding the mismatch between the demands of risk assessment and practice of scientists--the case of Deca
Effects of dose, administration route, and/or vehicle on decabromodiphenyl ether concentrations in plasma of mat
A global review of polybrominated diphenyl ether flame retardant contamination in birds.
Protective effect of N-acetylcysteine against BDE-209-induced neurotoxicity in primary cultured neonatal rat hippo
Mixture of dominant PBDE congeners (BDE-47, -99, -100 and -209) at levels noted in human blood dramatically en
Levels and potential sources of decabromodiphenyl ethane (DBDPE) and decabromodiphenyl ether (DecaBDE) in la
Reaction of decabrominated diphenyl ether by zerovalent iron nanoparticles.
Neurodevelopmental effects of decabromodiphenyl ether (BDE-209) and implications for the reference dose.
Impaired oligodendroglial development by decabromodiphenyl ether in rat offspring after maternal exposure from
Re: Viberg H, et al. Neurobehavioral derangements in adult mice receiving decabrominated diphenyl ether (PBDE 2
Ah receptor agonists in UV-exposed toluene solutions of decabromodiphenyl ether (decaBDE) and in soils contami
Postnatal exposure of the male mouse to 2,2',3,3',4,4',5,5',6,6'-decabrominated diphenyl ether: decreased epididy
Risk assessment for children exposed to decabromodiphenyl (oxide) ether (Deca) in the United States.
Levels and distribution of polybrominated diphenyl ethers in various tissues of birds of prey.
Concentrations of decabromodiphenyl ether in air from Southern Ontario: implications for particle-bound transpor
Toxicity of three halogenated flame retardants to nitrifying bacteria, red clover (Trifolium pratense), and a soil inve
Methods for synthesis of nonabromodiphenyl ethers and a chloro-nonabromodiphenyl ether.
Occupational exposure to commercial decabromodiphenyl ether in workers manufacturing or handling flame-retar
Polybrominated diphenyl ethers (PBDEs) in U.S. computers and domestic carpet vacuuming: possible sources of hu
Anaerobic degradation of decabromodiphenyl ether.
[Impact of PBDE-209 exposure during pregnancy and lactation on immune function of offspring rats].
Solar photodecomposition of decabromodiphenyl ether: products and quantum yield.
Prenatal oral (gavage) developmental toxicity study of decabromodiphenyl oxide in rats.
Photolytic debromination of decabromodiphenyl ether (BDE 209).
Debromination of the flame retardant decabromodiphenyl ether by juvenile carp (Cyprinus carpio) following dieta

Neurobehavioral derangements in adult mice receiving decabrominated diphenyl ether (PBDE 209) during a define
Geographical distribution and accumulation features of PBDEs in human breast milk from Indonesia.
The toxicology of the three commercial polybrominated diphenyl oxide (ether) flame retardants.
Wet air co-oxidation of decabromodiphenyl ether (BDE209) and tetrahydrofuran.
In vitro dermal absorption of flame retardant chemicals.
Qualitative determination of 10,10'-oxybisphenoxarsine and decabromodiphenyl ether in plastics.
Decabromodiphenyl oxide.
A multi-biomarker risk assessment of the impact of brominated flame retardant-decabromodiphenyl ether (BDE2C
Neurobehavioral effects of decabromodiphenyl ether (Deca) in neonatal mice.
Accumulation, whole-body depletion, and debromination of decabromodiphenyl ether in male sprague-dawley rat
Changes in spontaneous behaviour and altered response to nicotine in the adult rat, after neonatal exposure to th
Accumulation, tissue-specific distribution and debromination of decabromodiphenyl ether (BDE 209) in European
A comparison of the properties of the major commercial PBDPO/PBDE product to those of major PBB and PCB prc
Congener distribution of polybrominated diphenyl ethers in feral carp (Cyprinus carpio) from the Llobregat River, S
Degradation pathways of decabromodiphenyl ether during hydrothermal treatment.
Disposition and metabolic profiling of [14C]-decabromodiphenyl ether in pregnant Wistar rats.
The flame retardants, polybrominated diphenyl ethers, are pregnane X receptor activators.
Toxicity assessment and vitellogenin expression in zebrafish (Danio rerio) embryos and larvae acutely exposed to b
Oxidative stress and renal toxicity after subacute exposure to decabrominated diphenyl ether in Wistar rats.
A review on human exposure to brominated flame retardants--particularly polybrominated diphenyl ethers.
Functional disorder of primary immunity responding to respiratory syncytial virus infection in offspring mice expos
Levels and congener profiles of polybrominated diphenyl ethers (PBDEs) in Zebra mussels (D. polymorpha) from La
Polybrominated diphenyl ether compounds in ringed, bearded, spotted, and ribbon seals from the Alaskan Bering
Toxic effects of brominated flame retardants in man and in wildlife.
BFR-governmental testing programme.
Polybrominated diphenyl ether flame retardants in the North American environment.
Levels and trends of polybrominated diphenylethers and other brominated flame retardants in wildlife.
Determination of brominated flame retardants, with emphasis on polybrominated diphenyl ethers (PBDEs) in envi
Polychlorinated biphenyls and polybrominated diphenyl ethers in the North American atmosphere.
Investigations on acute hepato- and nephrotoxicity of pentabromophenol.
Mitochondrial-related effects of pentabromophenol, tetrabromobisphenol A, and triphenyl phosphate on murine
Emission patterns and risk assessment of polybrominated diphenyl ethers and bromophenols in water and sedime
Oxidative stress, cell cycle arrest, DNA damage and apoptosis in adult zebrafish (Danio rerio) induced by tris(1,3-di
Toxicity of TDCPP and TCEP on PC12 cell: changes in CAMKII, GAP43, tubulin and NF-H gene and protein levels.
Targeting neurotrophic factors and their receptors, but not cholinesterase or neurotransmitter, in the neurotoxicity
In ovo transformation of two emerging flame retardants in Japanese quail (Coturnix japonica).
Diester metabolites of the flame retardant chemicals, tris(1,3-dichloro-2-propyl)phosphate and tris(2,3-dibromopr
Analytical human biomonitoring method for the identification and quantification of the metabolite BDCPP originat
A protective role of autophagy in TDCIPP-induced developmental neurotoxicity in zebrafish larvae.
Effect of the flame retardant tris (1,3-dichloro-2-propyl) phosphate (TDCPP) on Na+-K+-ATPase and Cl- transport in
Effects of tris(1,3-dichloro-2-propyl) phosphate (TDCPP) and triphenyl phosphate (TPP) on sex-dependent alteratio
Exposure to tris(1,3-dichloro-2-propyl) phosphate (TDCPP) induces vascular toxicity through Nrf2-VEGF pathway in
Tris (1,3-dichloro-2-propyl) phosphate treatment induces DNA damage, cell cycle arrest and apoptosis in murine R
Tris(1,3-dichloro-2-propyl)phosphate (TDCIPP) disrupts zebrafish tail fin development.
Tris(1,3-dichloro-2-propyl) phosphate disturbs mouse embryonic development by inducing apoptosis and abnorma
Toxic effect and mechanism of tris (1,3-dichloro-2-propyl)phosphate (TDCPP) on the marine alga Phaeodactylum tı
Mass spectrometric determination of tris(1,3-dichloro-2-propyl)-phosphate (TDCP) using NCI-technique.

Comparative genotoxicity and nephrotoxicity studies of the two halogenated flame retardants tris(1,3-dichloro-2-p
[Effect of oral administration of tris(1,3-dichloroisopropyl)phosphate to pregnant rats on prenatal and postnatal de
Effects of tris(1,3-dichloro-2-propyl) phosphate and tris(1-chloropropyl) phosphate on cytotoxicity and mRNA expr
The metabolism and disposition of tris(1,3-dichloro-2-propyl) phosphate (Fyrol FR-2) in the rat.
In Ovo effects of two organophosphate flame retardants--TCPP and TDCPP--on pipping success, development, mRI
In vitro dermal absorption of flame retardant chemicals.
Discontinued pajama flame retardant detected in baby products and house dust.
Is the PentaBDE replacement, tris (1,3-dichloro-2-propyl) phosphate (TDCPP), a developmental neurotoxicant? Stu
Exposure to TDCPP appears widespread.
Tris(1,3-dichloro-2-propyl) phosphate disrupts axonal growth, cholinergic system and motor behavior in early life z
Monitoring indoor exposure to organophosphate flame retardants: hand wipes and house dust.
Bioconcentration, metabolism and neurotoxicity of the organophorous flame retardant 1,3-dichloro 2-propyl phos
Bioconcentration and transfer of the organophorous flame retardant 1,3-dichloro-2-propyl phosphate causes thyro
Neurotoxicological and thyroid evaluations of rats developmentally exposed to tris(1,3-dichloro-2-propyl)phospha
Developmental exposure to organophosphate flame retardants causes behavioral effects in larval and adult zebraf
The involvement of autophagy and cytoskeletal regulation in TDCIPP-induced SH-SY5Y cell differentiation.
Brominated and organophosphate flame retardants target different neurodevelopmental stages, characterized wit
Early zebrafish embryogenesis is susceptible to developmental TDCPP exposure.
Effects of organophosphorus flame retardant TDCPP on normal human corneal epithelial cells: Implications for hur
Environmentally Relevant Concentrations of the Flame Retardant Tris(1,3-dichloro-2-propyl) Phosphate Inhibit Gro
Tris (1,3-dichloro-2-propyl) phosphate induces toxicity by stimulating CaMK2 in PC12 cells.
High Exposure to Organophosphate Flame Retardants in Infants: Associations with Baby Products.
Tris(1,3-dichloro-2-propyl)phosphate Induces Genome-Wide Hypomethylation within Early Zebrafish Embryos.
Acute Exposure to Tris(1,3-dichloro-2-propyl) Phosphate (TDCIPP) Causes Hepatic Inflammation and Leads to Hepa
The Flame-Retardant Tris(1,3-dichloro-2-propyl) Phosphate Represses Androgen Signaling in Human Prostate Canc
Parental transfer of tris(1,3-dichloro-2-propyl) phosphate and transgenerational inhibition of growth of zebrafish e
Steroid secretion following exposure of ovarian follicular cells to single congeners and defined mixture of polybron
A multilevel approach to predict toxicity in copepod populations: assessment of growth, genetics, and population s
Penta- and octa-bromodiphenyl ethers promote proinflammatory protein expression in human bronchial epithelia
Effects of BDE-85 on the oxidative status and nerve conduction in rodents.
Physicochemical properties of selected polybrominated diphenyl ethers and extension of the UNIFAC model to bro
Semivolatile organic compounds in homes: strategies for efficient and systematic exposure measurement based or
Technical pentabromodiphenyl ether and hexabromocyclododecane as activators of the pregnane-X-receptor (PXR
A commercial mixture of the brominated flame retardant pentabrominated diphenyl ether (DE-71) induces respira
Probing new approaches using atmospheric pressure photo ionization for the analysis of brominated flame retarda
Ultrastructural changes observed in rat ovaries following in utero and lactational exposure to low doses of a polyb
Differential expression of CYP1A, 2B, and 3A genes in the F344 rat following exposure to a polybrominated dipheny
Brief postnatal PBDE exposure alters learning and the cholinergic modulation of attention in rats.
Developmental exposure to low dose PBDE 99: effects on male fertility and neurobehavior in rat offspring.
Brominated flame retardants in Alburnus alburnus from Cinca River Basin (Spain).
Exposure to flame retardants: nursing concern.
Polybrominated diphenyl ethers (PBDEs) in U.S. computers and domestic carpet vacuuming: possible sources of hu
Effects of pentabrominated diphenyl ether (PBDE-99) on vitamin status in domestic duck (Anas platyrhynchos) hat
Neurotoxicity of the pentabrominated diphenyl ether mixture, DE-71, and hexabromocyclododecane (HBCD) in rat
Polybrominated diphenyl ether (PBDE)-induced alterations in vitamin A and thyroid hormone concentrations in the
In vivo and in vitro Ah-receptor activation by commercial and fractionated pentabromodiphenylether using zebrafi
Comparison of analytical strategies for the chromatographic and mass spectrometric measurement of brominated

Brominated flame retardants in tree bark from North America.
Exposure to polybrominated diphenyl ethers (PBDEs): changes in thyroid, vitamin A, glutathione homeostasis, and
Dietary accumulation efficiencies and biotransformation of polybrominated diphenyl ethers in farmed Atlantic salr
New multiresidue analytical method dedicated to trace level measurement of brominated flame retardants in hum
Proteomic evaluation of neonatal exposure to 2,2 ,4,4 ,5-pentabromodiphenyl ether.
Effects of selected polybrominated diphenyl ether flame retardants on lake trout (Salvelinus namaycush).
Tissue disposition, excretion and metabolism of 2,2',4,4',5-pentabromodiphenyl ether (BDE-99) in the male Spragu
Differential effects of polybrominated diphenyl ethers and polychlorinated biphenyls on [3H]arachidonic acid relea
Levels of polybrominated diphenyl ether (PBDE) flame retardants in animals representing different trophic levels o
The toxicology of the three commercial polybrominated diphenyl oxide (ether) flame retardants.
Effects of perinatal exposure to a polybrominated diphenyl ether (PBDE 99) on mouse neurobehavioural developm
The expression of CYP1A, vitellogenin and zona radiata proteins in Atlantic salmon (Salmo salar) after oral dosing w
Thyroid insult: flame retardants linked to alterations in pregnant women's TSH levels.
Flame retardant exposure: polybrominated diphenyl ethers in blood from Swedish workers.
PBDE information overlooked?
A brominated flame retardant, 2,2',4,4',5-pentabromodiphenyl ether: uptake, retention, and induction of neurobe
Neonatal exposure to the brominated flame retardant 2,2',4,4',5-pentabromodiphenyl ether causes altered suscep
[Flame retardants--use and hazards for human].
Some polybrominated diphenyl ether (PBDE) flame retardants with wide environmental distribution inhibit TCDD-i
Temporal trends and spatial distributions of brominated flame retardants in archived fishes from the Great Lakes.
Impact of fermented brown rice with Aspergillus oryzae (FEBRA) intake and concentrations of polybrominated dipl
Occurrence and bioavailability of polybrominated diphenyl ethers and hexabromocyclododecane in sediment and
U.S. PBDE milestones.
Salmon flame retardant research raises new questions.
Clarifications on PBDE flame retardants.
Occurrence of polychlorinated biphenyls and polybrominated diphenyl ethers in green mussels (Perna viridis) from
Preventing fires, igniting questions.
Deca PBDE flame retardant gets around.
Screening of halogenated aromatic compounds in some raw material lots for an aluminium recycling plant.
Effects of the Commercial Flame Retardant Mixture DE-71 on Cytokine Production by Human Immune Cells.
Developmental neurotoxicity of polybrominated diphenyl ethers mixture de71 in Sprague-Dawley rats.
Propelling plastics into the circular economy - weeding out the toxics first.
Comparative Study of Genotoxicity Induced by Six Different PBDEs.
The environmental fate of polybrominated diphenyl ethers (PBDEs) in western Taiwan and coastal waters: evaluati
DNA Methylation Changes in Tbx3 in a Mouse Model Exposed to Polybrominated Diphenyl Ethers.
Effect of smoking and caffeine consumption on polybrominated diphenyl ethers (PBDE) and polybrominated biphe
Polybrominated diphenyl ethers alter hepatic phosphoenolpyruvate carboxykinase enzyme kinetics in male Wistar
Exposure to polybrominated diphenyl ethers and female reproductive function: A study in the production area of S
Novel and high volume use flame retardants in US couches reflective of the 2005 PentaBDE phase out.
After the PBDE phase-out: a broad suite of flame retardants in repeat house dust samples from California.
Temporal trends of PBDEs and emerging flame retardants in belugas from the St. Lawrence Estuary (Canada) and c
Geographical distribution of non-PBDE-brominated flame retardants in mussels from Asian coastal waters.
Differences in neonatal neurotoxicity of brominated flame retardants, PBDE 99 and TBBPA, in mice.
Porphyrogenic effect of pentabromodiphenyl ether after repeated administration to rats.
Associations between PBDEs in office air, dust, and surface wipes.
Alterations to the circuitry of the frontal cortex following exposure to the polybrominated diphenyl ether mixture,
Prenatal transfer of polybrominated diphenyl ethers (PBDEs) results in developmental neurotoxicity in zebrafish lar

Associations between serum levels of polybrominated diphenyl ether (PBDE) flame retardants and environmental
Acute postnatal exposure to the pentaBDE commercial mixture DE-71 at 5 or 15 mg/kg/day does not produce lear
Tissue-specific accumulation of polybrominated diphenyl ethers (PBDEs) including Deca-BDE and hexabromocycloc
Immune function in female B(6)C(3)F(1) mice is modulated by DE-71, a commercial polybrominated diphenyl ethe
Detection of polybrominated biphenyl ethers (PBDEs) in pediatric hair as a tool for determining in utero exposure.
Effects of chronic exposure to an environmentally relevant mixture of brominated flame retardants on the reprodu
Fetal exposure to polybrominated diphenyl ethers and the risk of hypospadias: focus on the congeners involved.
Lack of effects of some individual polybrominated diphenyl ether (PBDE) and polychlorinated biphenyl (PCB) conge
Epigenetic Effects of Polybrominated Diphenyl Ethers on Human Health.
The occurrence of polybrominated diphenyl ether (PBDE) contamination in soil, water/sediment, and air.
Brominated flame retardants in animal derived foods in the Netherlands between 2009 and 2014.
Polybrominated diphenyl ethers (PBDEs) in chicken eggs and cow milk around municipal dumpsites in Abuja, Niger
Concentrations and loadings of organophosphate and replacement brominated flame retardants in house dust fro
Single-cell RNA-sequencing analysis of estrogen- and endocrine-disrupting chemical-induced reorganization of mou
[Reproductive toxicity of PBDE in males: Advances in studies].
Immunologic and endocrine effects of the flame-retardant pentabromodiphenyl ether (DE-71) in C57BL/6J mice.
A noninvasive environmental monitoring tool for brominated flame-retardants (BFRs) assisted by conservation det
Racial/ethnic and geographic differences in polybrominated diphenyl ether (PBDE) levels across maternal, placenta
Polybrominated biphenyl and diphenylether flame retardants: analysis, toxicity, and environmental occurrence.
Decreasing but still high levels of halogenated flame retardants in wetland birds in central Spain.
Dietary exposure to a binary mixture of polybrominated diphenyl ethers alters innate immunity and disease suscep
Distribution of polybrominated diphenyl ethers in the atmosphere of the Pearl River Delta region, South China.
Temporal trends and developmental patterns of plasma polybrominated diphenyl ether concentrations over a 15-y
Association of In Utero Exposure to Polybrominated Diphenyl Ethers With the Risk of Hypospadias.
Concentrations of legacy and novel brominated flame retardants in indoor dust in Melbourne, Australia: An assess
Polybrominated diphenyl ethers (PBDEs) and hydroxylated PBDE metabolites (OH-PBDEs): A six-year temporal tren
Multi-analyte method development for analysis of brominated flame retardants (BFRs) and PBDE metabolites in hu
Trophic level determines levels of brominated flame-retardants in coastal herring gulls.
The flame retardant DE-71 (a mixture of polybrominated diphenyl ethers) inhibits human differentiated thyroid ce
The brominated flame retardant PBDE 99 promotes adipogenesis via regulating mitotic clonal expansion and PPAR
A preliminary study on prenatal polybrominated diphenyl ether serum concentrations and intrinsic functional netw
Species and habitat-dependent accumulation and biomagnification of brominated flame retardants and PBDE met
Pine needles as biomonitors of polybrominated diphenyl ethers and emerging flame retardants in the atmosphere
Prenatal polybrominated diphenyl ethers exposure and anogenital distance in boys from a Shanghai birth cohort.
Occurrence, levels and profiles of brominated flame retardants in daily-use consumer products on the Chinese ma
Brominated flame retardants in the environment of Asia-Pacific: an overview of spatial and temporal trends.
DE-71-induced apoptosis involving intracellular calcium and the Bax-mitochondria-caspase protease pathway in hu
Flame retardants (PBDEs) in marine turtles, dugongs and seafood from Queensland, Australia.
A 28-day oral dose toxicity study enhanced to detect endocrine effects of a purified technical pentabromodipheny
Polybrominated diphenyl ethers as endocrine disruptors of adipocyte metabolism.
Spatial trends of polybrominated diphenyl ethers in avian species: utilization of stored samples in the Environment
Desorption and bioavailability of spiked pentabromo diphenyl ether and tetrachlorodibenzo(p)dioxin in contamina
Neurotoxicity of a polybrominated diphenyl ether mixture (DE-71) in mouse neurons and astrocytes is modulated
Linking PBDEs in house dust to consumer products using X-ray fluorescence.
Electrolytic debromination of PBDEs in DE-83 technical decabromodiphenyl ether.
Bioaccumulation behaviour of polybrominated diphenyl ethers (PBDEs) in a Canadian Arctic marine food web.
Measurement of polybrominated diphenyl ethers on hand wipes: estimating exposure from hand-to-mouth conta

Detection of PBDE effects on mRNA expression in chicken (Gallus domesticus) neuronal cells using real-time RT-PC
Exposure to brominated flame retardant PBDE-99 affects cytoskeletal protein expression in the neonatal mouse ce
Plasma PBDE and thyroxine levels in rats exposed to Bromkal or BDE-47.
Accumulation, tissue-specific distribution and debromination of decabromodiphenyl ether (BDE 209) in European
PBDEs in circuit boards.
Polybrominated diphenyl ether in sewage sludge in Germany.
Brominated flame retardants and halogenated phenolic compounds in North American west coast bald eaglet (Hal
Congener distribution of polybrominated diphenyl ethers in feral carp (Cyprinus carpio) from the Llobregat River, S
Polybrominated diphenyl ethers, a group of brominated flame retardants, can interact with polychlorinated bipher
Coexposure of neonatal mice to a flame retardant PBDE 99 (2,2',4,4',5-pentabromodiphenyl ether) and methyl me
Decabromodiphenyl ether (deca-BDE) commercial mixture components, and other PBDEs, in airborne particles at
Flame retardants in placenta and breast milk and cryptorchidism in newborn boys.
Variation, levels and profiles of organochlorines and brominated flame retardants in great tit (Parus major) eggs fr
Geographical distribution and accumulation features of PBDEs in human breast milk from Indonesia.
The risk of PBDEs in dust.
The flame retardants, polybrominated diphenyl ethers, are pregnane X receptor activators.
Flame retardants have different effects at high and low doses.
Individual characteristics associated with PBDE levels in U.S. human milk samples.
Toxicity of penta- and decabromodiphenyl ethers after repeated administration to rats: a comparative study.
Toxicity assessment of air-delivered particle-bound polybrominated diphenyl ethers.
More signs of neurotoxicity of surfactants and flame retardants - Neonatal PFOS and PBDE 99 cause transcriptiona
Reductive debromination of polybrominated diphenyl ethers by anaerobic bacteria from soils and sediments.
Multi-generational effects of polybrominated diphenylethers exposure: embryonic exposure of male American kes
Polychlorinated biphenyls and polybrominated diphenyl ethers alter striatal dopamine neurochemistry in synaptos
PBDE exposure from food in Ireland: optimising data exploitation in probabilistic exposure modelling.
Brominated diphenyl ether (BDE) levels in liver, adipose, and milk from adult and juvenile rats exposed by gavage t
Polybrominated diphenyl ether (PBDE) levels in peregrine falcon (Falco peregrinus) eggs from California correlate v
The effect of short-term intoxication of rats with pentabromodiphenyl ether (in mixture mimic commercial produc
[Preliminary study of PBDE levels in house dust and human exposure to PBDEs via dust ingestion].
Reproductive and developmental toxicity of a pentabrominated diphenyl ether mixture, DE-71, to ranch mink (Mu
Polybrominated diphenyl ether (PBDE) concentrations in house dust are related to hormone levels in men.
The effects of marginal maternal vitamin A status on penta-brominated diphenyl ether mixture-induced alteration
Chronic postnatal DE-71 exposure: effects on learning, attention and thyroxine levels.
Toxicity of polybrominated diphenyl ethers (DE-71) in chicken (Gallus gallus), mallard (Anas platyrhynchos), and An
Effects of perinatal PBDE exposure on hepatic phase I, phase II, phase III, and deiodinase 1 gene expression involve
Spending time in vehicles can increase PBDE exposure.
Levels and congener specific profiles of PBDEs in human breast milk from China: implication on exposure sources a
In vitro neurotoxicity of PBDE-99: immediate and concentration-dependent effects on protein expression in cerebr
Exposure to DE-71 alters thyroid hormone levels and gene transcription in the hypothalamic-pituitary-thyroid axis
In vitro metabolism of the brominated flame retardants 2-ethylhexyl-2,3,4,5-tetrabromobenzoate (TBB) and bis(2-
Sex-specific responses in neuroanatomy of hatchling American kestrels in response to embryonic exposure to the
Effects of food-borne exposure of juvenile rainbow trout (Oncorhynchus mykiss) to emerging brominated flame re
Estimation of human percutaneous bioavailability for two novel brominated flame retardants, 2-ethylhexyl 2,3,4,5
Disposition of the Emerging Brominated Flame Retardant, 2-Ethylhexyl 2,3,4,5-Tetrabromobenzoate, in Female SD
New brominated flame retardants and their metabolites as activators of the pregnane X receptor.
Non-PBDE halogenated flame retardants in Canadian indoor house dust: sampling, analysis, and occurrence.
In vitro endocrine disruption and TCDD-like effects of three novel brominated flame retardants: TBPH, TBB, &

First insight into human extrahepatic metabolism of flame retardants: Biotransformation of EH-TBB and Firemaste
Species specific differences in the in vitro metabolism of the flame retardant mixture, Firemaster® BZ-54.
Identification and evaluation of a novel heterocyclic brominated flame retardant tris(2,3-dibromopropyl) isocyanu
Levels and distribution of tris-(2,3-dibromopropyl) isocyanurate and hexabromocyclododecanes in surface sedime
Tris-(2,3-dibromopropyl) isocyanurate induces depression-like behaviors and neurotoxicity by oxidative damage ar
Indirect competitive enzyme-linked immunosorbent assay of tris-(2,3-dibromopropyl) isocyanurate with monoclor
Toxicity of new emerging pollutant tris-(2,3-dibromopropyl) isocyanurate on BALB/c mice.
Influence of tris(2,3-dibromopropyl) isocyanurate on the expression of photosynthesis genes of Nannochloropsis s
Simultaneous determination of hexabromocyclododecanes and tris (2,3-dibromopropyl) isocyanurate using LC-AP
Tris(2,3-dibromopropyl) isocyanurate, hexabromocyclododecanes, and polybrominated diphenyl ethers in mollusk
Impaired gas bladder inflation in zebrafish exposed to a novel heterocyclic brominated flame retardant tris(2,3-dib
Levels of flame retardants HBCD, TBBPA and TBC in surface soils from an industrialized region of East China.
The mutagenic evaluation of tetrakis (hydroxymethyl) phosphonium sulfate using a combined testing protocol app
Synthesis of tetrakis (hydroxymethyl) phosphonium chloride by high-concentration phosphine in industrial off-gas.
Simultaneous determination of melamine, ammelide, ammeline, and cyanuric acid in milk and milk products by ga
Melamine and its derivatives in dog and cat urine: An exposure assessment study.
3D-map modelling for the melting points prediction of intumescent flame-retardant coatings.
Environmentally friendly flame retardants. A detailed solid-state NMR study of melamine orthophosphate.
Water-based chitosan/melamine polyphosphate multilayer nanocoating that extinguishes fire on polyester-cotton
Assessment on dietary melamine exposure from tainted infant formula.
A survey on occurrence of melamine and its analogues in tainted infant formula in China.
Occurrence of polybrominated diphenylethers, hexabromocyclododecanes, bromophenols and tetrabromobisphe
Evaluation of hepatic biotransformation of polybrominated diphenyl ethers in the polar bear (Ursus maritimus).
Toxicity of brominated flame retardants, BDE-47 and BDE-99 stems from impaired mitochondrial bioenergetics.
BDE 49 and developmental toxicity in zebrafish.
PBDEs in cod (Gadus morhua) liver products (1972-2017): Occurrence and human exposure.
Highly sensitive and selective photoelectrochemical biosensor platform for polybrominated diphenyl ether detecti
Comparison of in vitro hormone activities of novel flame retardants TBB, TBPH and their metabolites TBBA and TB
Quantification of tetrabromo benzoic acid and tetrabromo phthalic acid in rats exposed to the flame retardant Uni
Nonmutagenicity of tetrabromophthalic anhydride and tetrabromophthalic acid in the ames Salmonella/microsom

anical, electrical, and thermal properties of intumescent flame-retardant ethylene-vinyl acetate copolymer/mic

limethylsilyl-N-methyltrifluoroacetamide-derivatization and gas chromatography-high resolution mass spectror

pressure chemical ionization-tandem mass spectrometry with emphasis in highly brominated congeners.

oxy) ethane flame retardants in kingfishers (Alcedo atthis) from an electronic waste-recycling site in South Chir

2,4,6-tribromophenoxy)ethane (BTBPE) and 2,4,6-tribromophenol, and formation during synthesis of BTBPE.

monopersulfate oxidation with an iron(III)-tetrakis(p-sulfonatophenyl)porphyrin in the presence of humic acid

retardants in great lakes herring gull eggs by liquid chromatography-atmospheric pressure photoionization-tar
thyl ether disrupts normal zebrafish (Danio rerio) development and matrix metalloproteinase expression.

quatic environment in a highly industrialized area, South China: vertical profile, phase partition, and bioaccumu

ıgqing, western China, with emphasis on diastereoisomer profiles, particle size distribution, and human exposu

ays and systematic investigation of their distributions in Taizhou, an e-waste recycling area in eastern China.

ng, China: Contamination levels, temporal trends, nursing infant's daily intake, and risk assessment.

etrabromobisphenol A mono(hydroxyethyl) ether: An effective and reliable strategy to estimate the typical tetı

edimetric detection of tetrabromobisphenol A bis(2-hydroxyethyl) ether and tetrabromobisphenol A mono(hyc

m mass spectrometry (GC-MS/MS): a smart procedure for (ultra)trace analysis of brominated flame retardants

uptake of glutamate in rat brain synaptosomes: a mathematical approach for the study of mixtures.

[illegible]-Cateninser675 Expression and Its Interaction With E-Cadherin in the Mammary Glands of Lactating Rats.

olvent extraction and solid-phase microextraction followed by gas chromatography-tandem mass spectrometr

thyl ether disrupts normal zebrafish (Danio rerio) development and matrix metalloproteinase expression.

monopersulfate oxidation with an iron(III)-tetrakis(p-sulfonatophenyl)porphyrin in the presence of humic acid

ocypris rarus) exposed to tri(2?butoxyethyl) phosphate (TBOEP) and tri-n-butyl phosphate (TNBP).

MS) for Polybrominated Diphenyl Ether (PBDE) and Emerging Brominated Flame Retardant (BFR) Determination

mys scripta elegans) and snapping turtles (Chelydra serpentina) and possible species-specific differences in del

tiation of primary cultured adult neural stem cells and interferes with signaling of ERK5 MAP kinase and neuro

ng, China: Contamination levels, temporal trends, nursing infant's daily intake, and risk assessment.

pan: Geographical distribution and polystyrene foam as a potential source of hexabromocyclododecanes.

ıgqing, western China, with emphasis on diastereoisomer profiles, particle size distribution, and human exposu

ıltra-performance liquid chromatography-tandem mass spectrometry and gas chromatography-mass spectrom

ıuatic environment in a highly industrialized area, South China: vertical profile, phase partition, and bioaccumu

ions and neurobehavioral and neuropathological abnormalities in rats exposed to tris(2-chloroethyl)phosphate

e via injection, with no evidence of effects on hatching success or latent effects on growth or reproduction in z

brominated diphenyl ethers and novel flame retardants in fish using fast, low-pressure gas chromatography-tar

minated flame retardants in hooded seals (Cistophora cristata) from the Gulf of St. Lawrence: applications for n

pan: Geographical distribution and polystyrene foam as a potential source of hexabromocyclododecanes.

uptake of glutamate in rat brain synaptosomes: a mathematical approach for the study of mixtures.
poises (Phocoena phocoena) from western European seas: geographical trends, causal factors and effects on re

2,4,6-tribromophenoxy)ethane (BTBPE) and 2,4,6-tribromophenol, and formation during synthesis of BTBPE.

anical, electrical, and thermal properties of intumescent flame-retardant ethylene-vinyl acetate copolymer/mic

anical, electrical, and thermal properties of intumescent flame-retardant ethylene-vinyl acetate copolymer/mic

thyl ether disrupts normal zebrafish (Danio rerio) development and matrix metalloproteinase expression.

od for the sensitive determination of Dechlorane plus and related norbornene-based flame retardants in food

on using the quantum dots sensitized three-dimensional, macroporous ZnO nanosheet photoelectrode.

2,4,6-tribromophenoxy)ethane (BTBPE) and 2,4,6-tribromophenol, and formation during synthesis of BTBPE.

receptor activation in the hepg2 hepatocellular carcinoma cell line and the lncap prostate cancer cell line.

pan: Geographical distribution and polystyrene foam as a potential source of hexabromocyclododecanes.

ng, China: Contamination levels, temporal trends, nursing infant's daily intake, and risk assessment.

ıgqing, western China, with emphasis on diastereoisomer profiles, particle size distribution, and human exposu

ıltra-performance liquid chromatography-tandem mass spectrometry and gas chromatography-mass spectrom

ıuatic environment in a highly industrialized area, South China: vertical profile, phase partition, and bioaccumu

ered testicular glucose homeostasis and oxidative status through down-regulation of Cx43 and p27Kip1 in prep

flame retardants bis(2-ethylhexyl)-2,3,4,5-tetrabromophthalate and 2-ethylhexyl-2,3,4,5-tetrabromobenzoate.

on using the quantum dots sensitized three-dimensional, macroporous ZnO nanosheet photoelectrode.

croencapsulated ammonium polyphosphate/polyamide-6 blends.

rabromobisphenol A derivative and byproduct in aquatic environments.

:roencapsulated ammonium polyphosphate/polyamide-6 blends.

:roencapsulated ammonium polyphosphate/polyamide-6 blends.
